# Characteristics of *Mycobacterium tuberculosis* PtpA interaction and activity on the alpha subunit of human mitochondrial trifunctional protein, a key enzyme of lipid metabolism

**DOI:** 10.3389/fcimb.2023.1095060

**Published:** 2023-06-22

**Authors:** Mariana Margenat, Gabriela Betancour, Vivian Irving, Alicia Costábile, Tania García-Cedrés, María Magdalena Portela, Federico Carrión, Fernando E. Herrera, Andrea Villarino

**Affiliations:** ^1^ Instituto de Biología, Sección Bioquímica, Facultad de Ciencias-Universidad de la República, Montevideo, Uruguay; ^2^ Instituto de Biología, Facultad de Ciencias-Universidad de la República, Montevideo, Uruguay; ^3^ Unidad de Bioquímica y Proteómica Analíticas, Institut Pasteur de Montevideo and Instituto de Investigaciones Biológicas Clemente Estable, Montevideo, Uruguay; ^4^ Laboratorio de Inmunovirología, Institut Pasteur de Montevideo, Montevideo, Uruguay; ^5^ Departamento de Física, Facultad de Bioquímica y Ciencias Biológicas-Universidad Nacional del Litoral – CONICET, Santa Fe, Argentina

**Keywords:** *Mycobacterium tuberculosis*, tyrosine phosphatase, PtpA, human mitochondrial trifunctional protein, TFP, ECHA, lipid metabolism, Jak

## Abstract

During *Mycobacterium tuberculosis (Mtb)* infection, the virulence factor PtpA belonging to the protein tyrosine phosphatase family is delivered into the cytosol of the macrophage. PtpA interacts with numerous eukaryotic proteins modulating phagosome maturation, innate immune response, apoptosis, and potentially host-lipid metabolism, as previously reported by our group. *In vitro*, the human trifunctional protein enzyme (*h*TFP) is a *bona fide* PtpA substrate, a key enzyme of mitochondrial β-oxidation of long-chain fatty acids, containing two alpha and two beta subunits arranged in a tetramer structure. Interestingly, it has been described that the alpha subunit of *h*TFP (ECHA, *h*TFPα) is no longer detected in mitochondria during macrophage infection with the virulent *Mtb* H37Rv. To better understand if PtpA could be the bacterial factor responsible for this effect, in the present work, we studied in-depth the PtpA activity and interaction with *h*TFP_α_. With this aim, we performed docking and *in vitro* dephosphorylation assays defining the P-Tyr-271 as the potential target of mycobacterial PtpA, a residue located in the helix-10 of *h*TFP_α_, previously described as relevant for its mitochondrial membrane localization and activity. Phylogenetic analysis showed that Tyr-271 is absent in TFP_α_ of bacteria and is present in more complex eukaryotic organisms. These results suggest that this residue is a specific PtpA target, and its phosphorylation state is a way of regulating its subcellular localization. We also showed that phosphorylation of Tyr-271 can be catalyzed by Jak kinase. In addition, we found by molecular dynamics that PtpA and *h*TFP_α_ form a stable protein complex through the PtpA active site, and we determined the dissociation equilibrium constant. Finally, a detailed study of PtpA interaction with ubiquitin, a reported PtpA activator, showed that additional factors are required to explain a ubiquitin-mediated activation of PtpA. Altogether, our results provide further evidence supporting that PtpA could be the bacterial factor that dephosphorylates *h*TFP_α_ during infection, potentially affecting its mitochondrial localization or β-oxidation activity.

## Introduction

Phosphatases and kinases are essential players in signal transduction, modulating enzyme activities, protein-protein interactions, and subcellular localization, among other processes. Our group is interested in contributing to a deeper understanding of the eukaryotic pathways modulated by the *Mycobacterium tuberculosis (Mtb)* phosphatase PtpA (Chiaradia et al., 2008; Mascarello et al., 2010; Margenat et al., 2015). This enzyme, which belongs to the Protein Tyrosine Phosphatase (PTP) family, is delivered into the macrophage during infection, acting as a critical virulence factor (Bach et al., 2008; Mascarello et al., 2010). PtpA was detected in the cytoplasm and the nucleus of mycobacteria-infected macrophages, despite lacking an export signal sequence. It has been suggested that the bacterial SecA2 and ESX/type VII export systems are the candidates responsible for PtpA export (Sullivan et al., 2012; Wong et al., 2013; Wang et al., 2016; Wang et al., 2017). The *Mtb* PtpA-deletion mutant strain showed reduced survival in infected human THP-1 derived macrophages and in mouse SPF C57BL/6, and expression of PtpA neutralizing antibodies and inhibitors simulated this effect (Bach et al., 2008; Mascarello et al., 2010; Wang et al., 2015). In addition, a *Mtb* mutant (*Mtb*Dmms) lacking the genes encoding PtpA, PtpB, and the acid phosphatase, SapM12, displayed a significantly reduced ability to infect and grow inside human THP-1 macrophages. Moreover, no bacilli were recovered in the spleens and lungs of guinea pigs ten weeks following infection with this mutant, suggesting an important role of these phosphatases in the colonization of these organs (Chauhan et al., 2013).

PtpA is a member of the Low-Molecular-Weight PTP (LMW-PTP) family (Denu et al., 1996) and shows 37% of sequence identity and high structural similarity with the *h*ACP1 (UniProt code P24666 isoforms 1 and 2) (Alonso et al., 2004; Madhurantakam et al., 2005). PtpA is a secreted protein during infection; thus, it constitutes an ideal target for drug design (Bach et al., 2008; Sullivan et al., 2012; Wong et al., 2013; Wang et al., 2017) as the drugs would not need to cross the mycobacterial envelope, a barrier that explains much of the resistance of *Mtb* to antibiotics (Chiaradia et al., 2017; Abrahams and Besra, 2020). Numerous groups, including ours, have identified PtpA inhibitors (Mascarello et al., 2010; Silva and Tabernero, 2010; Wong et al., 2013; Mascarello et al., 2016). However, the candidates targeted the PtpA active site, which is highly conserved within PTPs including human ones (Denu et al., 1996; Madhurantakam et al., 2005). Thus, identifying less conserved secondary sites continues to be a challenge. During infection, the action of PtpA seems to depend in part of its phosphatase activity (Wang et al., 2017, 2016; 2020). PtpA interacts with numerous eukaryotic proteins modulating several cell signaling pathways such as phagosome maturation, innate immune response, apoptosis, and host lipid metabolism. Dephosphorylation of VPS33B (Vacuolar Protein Sorting 33B) by PtpA seems to exclude host vacuolar-H1-ATPase from phagosomes, leading to inhibition of phagosome acidification and maturation (Bach et al., 2008; Wong et al., 2011). Also, the presence of PtpA correlates with a decrease in the production of pro-inflammatory cytokines (TNF*α*, IL-1β, and IL-12) during macrophage infection (Wang et al., 2015). In addition, GSK3*α* (Glycogen Synthase Kinase 3, alpha subunit) dephosphorylation by PtpA avoids kinase activation promoting an anti-apoptotic pathway, supporting pathogen survival within host macrophage (Poirier et al., 2014). On the other hand, some reports connect PtpA with the degradation pathways induced by ubiquitin (Wang et al., 2017, 2015; 2016; 2020) showing that it acts as an activator of PtpA (using the artificial substrate pNPP or other reported substrates as VPS33B, Jnk and p38) (Wang et al., 2015). Finally, our group identified the human trifunctional enzyme *h*TFP, a key enzyme in the β-oxidation of long-chain fatty acids, as a bona fide PtpA substrate (Eaton et al., 2000; Margenat et al., 2015). Like most proteins with a role in the mitochondria, TFP is synthesized in the cytosol and then translocated to this organelle (Bykov et al., 2020). In the mitochondria, TFP plays a central role in the β-oxidation of long-chain fatty acids, catalyzing three of the four stages of this pathway (Eaton et al., 2000). Recently, the crystallographic structure of *h*TFP has been resolved, showing that it is a α_2_β_2_-heterotetramer in which helix-10 of the alpha subunit (*h*TFP_α_) appears to be important for anchoring to the inner mitochondrial membrane and for its activity (Xia et al., 2019). Our group has a particular interest in the alpha subunit *h*TFP_α_ because during five independent assays of substrate trapping it was the only PtpA-substrate candidate isolated in the five replicates and identified with the best Mascot score (Margenat et al., 2015). In addition, *h*TFP_α_ was no longer detected in the mitochondria of macrophages infected with the virulent *Mtb* H37Rv, where its expression was strongly modulated (more than a 10-fold mRNA decrease) (Jamwal et al., 2013). To better understand if PtpA could be the bacterial factor responsible for this effect, in the present work, we identified by *in vitro* and *in silico* approaches the characteristics of the PtpA-*h*TFP_α_ interaction, the *h*TFP_α_ Tyr phosphorylation/dephosphorylation and evaluated the potential regulation of PtpA phosphatase activity by ubiquitin.

## Results and discussion

### The p-Tyr271 of *h*TFP_α_ is the potential target of mycobacterial PtpA

**Figure 1:**
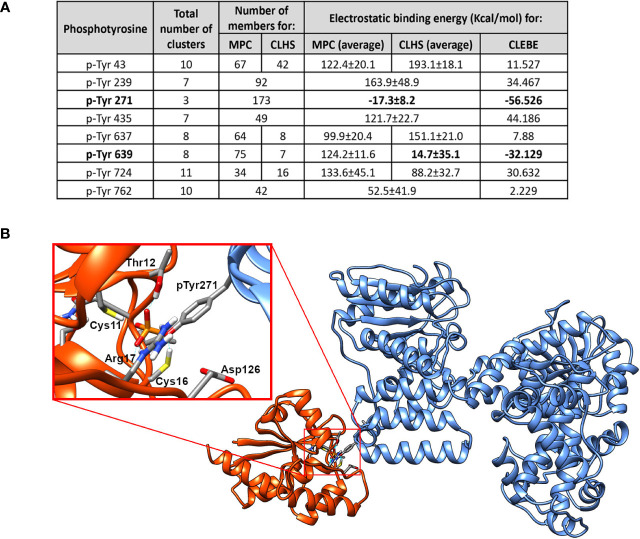
The P-Tyr271 of *h*TFP_α_ is the potential target of mycobacterial PtpA. **(A)** Table showing the results of the docking assays between mycobacterial PtpA and *h*TFP_α_. The total number of clusters obtained for each p-Tyr evaluated, the number of members, and the electrostatic binding energy are indicated. MPC: Most Populated Cluster; CLHS: Cluster with the Lowest Haddock Score, CLEBE: Complex with the Lowest Electrostatic Binding Energy. In the case where the MPC is the same as the CLHS, both columns are merged into one. **(B)** Molecular structure of the best model of the PtpA/*h*TFP_α_ complex obtained after docking assays. In the enlarged inset, the most critical residues of the PtpA (orange) binding region and *h*TFP_α_ (blue) are represented as sticks. These include the catalytic residues of PtpA (Cys11, Thr12, Cys16, Arg17, Asp126) and the p-Tyr 271 of *h*TFP_α_.

We have previously reported that recombinant PtpA (rPtpA) interacts and dephosphorylates *h*TFP_α_ immunopurified from macrophages and that its dephosphorylation is dependent on the PtpA dose (Margenat et al., 2015). To identify the phosphotyrosine potentially dephosphorylated by PtpA, we performed *in silico* docking studies, using the crystal structure of *h*TFP_α_ (extracted from PDB 6DV2) and of *Mtb* PtpA (PDB 1U2P) (Madhurantakam et al., 2005; Xia et al., 2019). The reported structure of the *h*TFP (Xia et al., 2019) showed that, as its bacterial homolog (*P. fragi*, PDB 1WDK) (Ishikawa et al., 2004), the biological unit was a *α*_2_ *β*_2_-heterotetramer. Nevertheless, *h*TFP presents significant primary and secondary structure differences compared to its bacterial counterparts, some of them in the *α* subunit, described as relevant for its association with the inner mitochondrial membrane (Fould et al., 2010; Xia et al., 2019). To drive the docking process, we selected the PtpA active site as we previously showed by SPR assays that the PtpA-*h*TFP interaction involves the active site of PtpA (Margenat et al., 2015). For *h*TFP_α_, we selected all tyrosine residues located at the surface (Tyr43, Tyr239, Tyr271, Tyr435, Tyr637, Tyr639, Tyr724, and Tyr762), and we phosphorylated them *in silico*. The inset table (**Figure 1A**) shows the different numbers of complexes (PtpA/*h*TFP_α_) and clusters obtained for each p-Tyr evaluated in the docking assays. The first four best representative complexes from each cluster were selected, counting 256 possible solutions. It is worth observing that a p-Tyr, with a lower number of clusters, indicates that the binding mode is well-defined and better than another with a greater number of clusters. Additionally, the clusters with lower electrostatic interaction energies (represented by HADDOCK scores) or with a higher number of members are preferable. Considering this, the interaction through the p-Tyr271 or p-Tyr639 of *h*TFP_α_ and the active site of PtpA results in at least one complex with negative electrostatic interaction energy. However, only the cluster of *h*TFP_α_ phosphorylated at the Tyr271 residue has a negative average electrostatic interaction energy. In this case, all complexes within this cluster have negative electrostatic interaction energy, and this cluster represents the one with the least energy and the most populated. The situation is different for the results of *h*TFP_α_ interaction through p-Tyr639. In this case, we found that the cluster with a higher number of members differs from the one with lower energy. Only one complex in the last has negative electrostatic interaction energy. The best PtpA-*h*TFP_α_ complex, involving Tyr271, is shown in **Figure 1B**. In this protein complex, it is possible to observe that the p-Tyr-271 fits in the PtpA active site, and there are no steric clashes between both proteins. The p-Tyr271 of *h*TFP_α_ is directly interacting with the catalytic Asp126 and the Cys11 and Cys16 of PtpA, as it should be for the enzymatic reaction to occur (Madhurantakam et al., 2008).

In addition, we evaluated if there exists a common sequence or structure pattern among the reported PtpA targets. For the mycobacterial substrate of PtpA, the ATP synthase subunit alpha (ATPA), the p-Tyr targeted by PtpA still needs to be elucidated (Chatterjee et al., 2019). For the eukaryotic substrates, GSK3α and VPS33B, only indirect evidence of which Tyr could be dephosphorylated by PtpA has been reported (Bach et al., 2008; Poirier et al., 2014). When considering these residues (Tyr279 of GSK3α, Tyr133 or Tyr382 of VPS33B) and the available alphafold structures models of these substrates, we did not find any sequence pattern around those residues (**Figure S1A**) or a conserved structural motif of interaction between PtpA and the reported substrates. One reason that could explain this result is that the p-Tyr of GSK3α and VPS33B were not identified using the same approach as our study, where an unbiased *in silico* analysis was applied to determine the p-Tyr candidates dephosphorylated by PtpA. Therefore, we cannot rule out that other Tyr of GSK3α and VPS33B could be the target of PtpA. Another reason is that alphafold models of GSK3α, VPS33B, and ATPA did not consider the phosphorylated state of the residues. Therefore, the orientation of the side chain of these residues might not be the correct one in the alphafold-modeled structure. Overall, we think that an unbiased *in silico* study, as carried out for PtpA with the *h*TFP_α_ as substrate, is needed for the other reported substrates to be sure of which Tyr is potentially dephosphorylated by PtpA and to evaluate correctly if a conserved recognition motif exists.

### The Tyr271 of *h*TFP_α_ is absent in bacteria and is conserved in mammals and located in a helix described as relevant to its localization and activity

**Figure 2:**
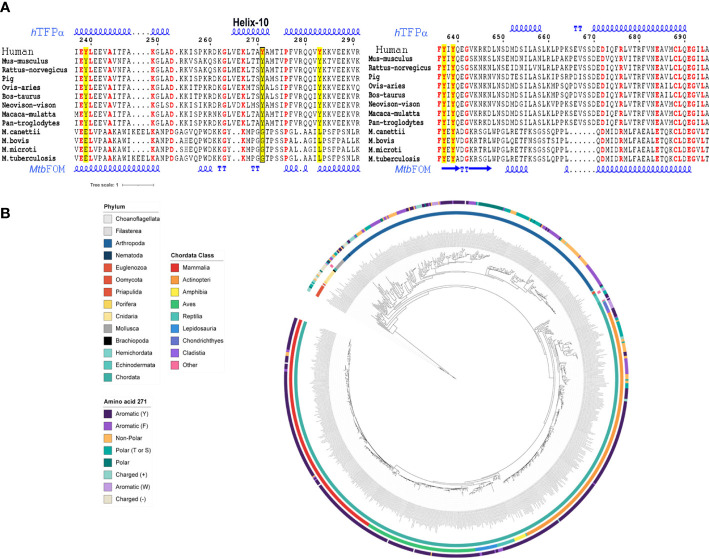
The Tyr271 of *h*TFP_α_ is conserved in several eukaryotic protein homologs and part of the helix-10, absent in bacterial homologs. **(A)** Multiple sequence alignment of *h*TFP_α_ homologous, representative of mammals and bacterial TB complex. The Tyr271 (left alignment) and Tyr639 (right alignment) conservation are shown. The complete alignments are shown in **Figures S1**–**S3**. The tyrosines of *h*TFP_α_ are in red and highlighted in yellow. The box indicates the Y271 residue placed in the helix-10 of the *h*TFP_α_. The alignment was obtained using the MUSCLE server (Edgar, 2004), and the figure was done using the ESPrit 3.0 server (Robert and Gouet, 2014). The secondary protein structure of *h*TFP_α_ and the *Mtb* FOM are also shown, based on the pdb structures 6DV2 (Xia et al., 2019) and 4B3H (Venkatesan and Wierenga, 2013), respectively. **(B)** Eukaryotic TFP_α_ phylogenetic analysis. Circular representation of a maximum likelihood tree of TFP_α_ of eukaryotes ranging from euglenozoos to vertebrates. The tree is midpoint rooted. Inner circle: phylum colored according to the legend. Middle circle: Chordata class colored according to the legend. Outer circle: amino acid present in each gene at the position aligned to human Tyr-271, colored according to the legend. For details, see **Figure S3**.

As it has been proved that mycobacterial PtpA introduced into the cytosol of the infected cells acts principally on eukaryotic proteins, this enzyme is expected to dephosphorylate a specific tyrosine residue of *h*TFP_α_, absent or not phosphorylated in the bacterial homologs. Thus, we evaluate the conservation of the Tyr271 and Tyr639 in bacterial TFP_α_ and eukaryotic homologs (**Figure 2A**, see **Figures S1**–**S3** for the complete alignments). While Tyr639 is in the HACD catalytic domain and is conserved in mammals and bacteria, Tyr271 seems specific to the mammal’s group, and it is part of the helix-10 of *h*TFP_α_ which is absent in bacterial TFP homologs (**Figures 2A, B**). Interestingly, this helix-10 was described as one of the helices relevant for the activity and mitochondrial localization of the *h*TFP (Xia et al., 2019). Then, we analyzed in detail the conservation of Tyr271 in eukaryotes employing blastp searches of the *h*TFP against the NCBI RefSeq protein database and a maximum likelihood phylogenetic analysis (**Figure 2B**). In chordates, Tyr271 is mainly conserved in mammals, birds, reptiles, and amphibians (**Figures 2B**, **S3**). In fish, there are two major clades; one contains 146 genes with a high degree of Tyr271 conservation, and the other with 57 genes without conservation of Tyr271. A closer look at this non-conserved clade indicates that these genes are, in most cases (53/57), different isoforms of a gene containing a conserved Tyr271. Fishes are generally polyploid (Rajkov et al., 2014), and additional copies of duplicated genes are free to change regarding one of the copies remaining functional. On the contrary, there is no conservation of Tyr271 in non-chordates (like arthropods, mollusks, or cnidaria), suggesting that the preservation of the Tyr271 is a characteristic of more complex organisms. These results allowed us to hypothesize a potential role of the Tyr271 residue in the regulation of *h*TFP_α_ subcellular localization/activity, supported by the fact that the subcellular localization of *h*TFP_α_ was modulated during TB infection, where this subunit was no longer detected in the mitochondria of macrophages infected with the virulent *Mtb* H37Rv (Jamwal et al., 2013).

### p-Tyr271 of *h*TFP_α_ is detected after phosphorylation by Jak kinase

**Figure 3:**
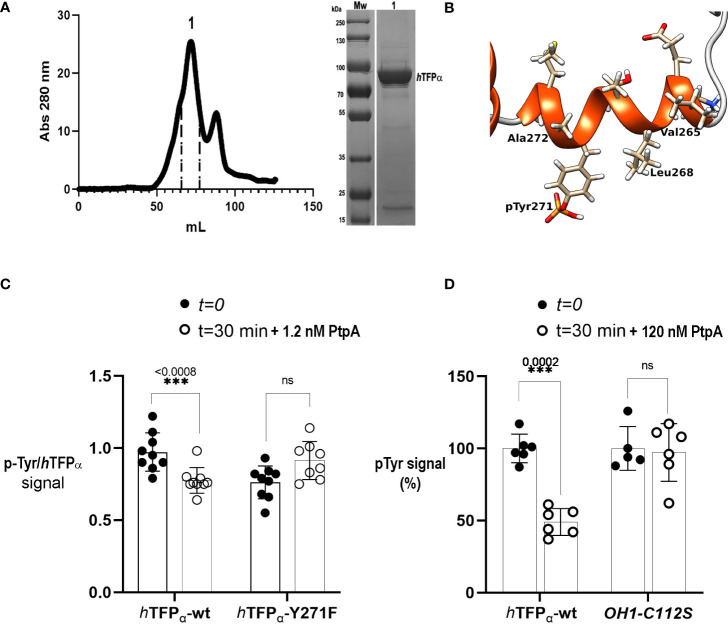
Mycobacterial PtpA activity and interaction with recombinant *hTFP*_α_. **(A)** SEC profiles of recombinant *h*TFP_α_ purification on Superdex 200 column. SEC was performed in an AKTA Basic system (GE Healthcare), injecting a sample volume of 5 mL in a Superdex 200 16/60 preparative grade column (GE Healthcare). Elution was carried out with two-bed column volumes of the equilibration buffer, and fractions containing the recombinant protein were pooled (fractions indicated in peak 1, elution volume 72 mL). The apparent molecular weight was calculated using the following equation *K*_*av*_= −0.3872 (log*M*w) + 2.2662 corresponding to the previous calibration curve of the column using SEC molecular weight (SIGMA). **(B)** Cartoon representation of the helix-10 of the *h*TFP_α_ containing the p-Tyr 271 phosphorylated by Jak. The residues of the helix are represented as sticks. The figure was generated with UCSF Chimera [Pettersen EF, 2004 et al.]. **(C)** Evaluation of dephosphorylation of *h*TFP_α_-wt and *h*TFP_α_-Y271F by PtpA. The graph shows the mean of p-Tyr/*h*TFP_α_ ratios ± SD of three independent experiments including three technical replicates. The asterisks indicate the p-value obtained after an unpaired t-test. Equal amounts of the recombinant *h*TFP_α_ and *h*TFP_α_ -Y271F previously phosphorylated by Jak were incubated with 1.2 nM of PtpA for 30 min at 37°C in solution. After this time, spots of 5 μL of the reaction were applied in a nitrocellulose membrane by triplicates. Dephosphorylation was evaluated by a Dot Blot assay with an anti-p-Tyr antibody and anti *h*TFP_α_ antibody to determine the ratio between p-Tyr and *h*TFP_α_ chemiluminescent signals, quantified using ImageJ (Schneider et al., 2012). **Figure S11** shows a representative dot blot used in the analysis. **(D)** Evaluation of PtpA specificity using *h*TFP_α_-wt and rOH1-C112S as substrates. The graph shows the obtained p-Tyr signal (expressed as %) after incubation with PtpA. Equal amounts of *h*TFP_α_ or rOH1-C112S previously phosphorylated by Jak were incubated with 120 nM of PtpA (corresponding to a E:S molar ratio of 1:50) for 30 min at 37°C. Then, spots of 5 μL of the reaction were applied in a nitrocellulose membrane by triplicates to perform a Dot Blot assay using an anti-p-Tyr antibody. Chemiluminescent signals were quantified using ImageJ (Schneider et al., 2012). The average p-Tyr signal at t=0 was considered as 100%. After a two-way ANOVA a significant difference (***) was detected between both groups (*h*TFP_α_ or rOH1-C112S). The asterisks represent the p-value obtained after an unpaired t-test between t=0 and t=30 minutes, and ns: not statistically significant. **Figure S12** shows a representative dot blot used in the analysis.

The next step was to show if Tyr271 of *h*TFP_α_ could exist in its phosphorylated state. The *h*TFP_α_ contains several post-translational modifications (PTMs) identified after large-scale MS studies and noted in the PhosphoSitePlus (Hornbeck et al., 2012). Among them, nine Tyr residues of *h*TFP_α_ were reported as phosphorylated, but the Tyr271 is not included (**Figure S7**). The functional significance of p-Tyr and the kinase involved has not been elucidated yet. In this context, to improve the identification of the p-residues of *h*TFP_α_, we prepared a batch of immunopurified *h*TFP from a macrophage phosphoprotein-enriched extract, as previously described (Margenat et al., 2015). We verified in this sample the presence of *h*TFP_α_ by MS (**Figure S4A**). However, the amounts of immunopurified *h*TFP were insufficient to detect the p-Tyr by MS and evaluate PtpA-mediated p-Tyr271 dephosphorylation. Phosphorylation generally represents a lower percentage of the total protein and could be lost during MS fragmentation (Mann et al., 2002; Dephoure et al., 2013) or by the action of endogenous phosphatases during the protein extract preparation, despite the precautions taken to inhibit them. This explanation is supported by the fact that Tyr271 is exposed to the solvent, as seen in its crystallographic structure (**Figure 1B**). Thus, to obtain enough material, we decided to produce and purify the recombinant *h*TFP_α_ based on previous evidence of its successful production in a soluble form in *E. coli* (Fould et al., 2010). After IMAC, SEC, and removal of the His-tag, 2.5 mg of soluble *h*TFP_α_/gr of *E. coli* pellet was obtained. As illustrated on the SEC chromatogram, we observed the main peak with a shoulder aspect (**Figure 3A**). A pool of the eluting fractions corresponding to this peak was analyzed by SDS-PAGE and MS, showing that the principal band is the *h*TFP_α,_ and the less intense band corresponds to an *E. coli* chaperone contaminant (**Figure S4B**). Then, taking into account that it is not known which eukaryotic kinase is involved in its phosphorylation, we decided to evaluate the Tyr phosphorylation by Janus kinase (Jak). This enzyme is an intracellular kinase involved in cytokine-mediated signaling, for which inhibitors of its activity were proposed for host-directed therapy of TB as an adjunct to standard TB chemotherapy (Dorhoi, 2015; Schwartz et al., 2017). Thus, we incubated the recombinant *h*TFP_α_ with catalytic amounts of the Jak kinase domain, using an E:S molar ratio of 1:3000 to avoid non-specific phosphorylation. For the recombinant *h*TFP_α_ non-treated with Jak, after SDS-PAGE and WB using an anti-p-Tyr antibody, we detected a signal of Tyr phosphorylation of *h*TFP_α_, and by MS, we identified Tyr499 as phosphorylated (**Figure S6**, already reported in PhosphoSitePlus). This result is explained because the recombinant *h*TFP_α_ was expressed in an *E. coli* strain with a tyrosine kinase/phosphatase system (Vincent et al., 1999). Then, for the recombinant *h*TFP_α_ pre-treated with Jak and ATP, we observed additional phosphorylation, detected by the electrophoretic shift of the protein in the SDS-PAGE (Villarino et al., 2005) as well as by WB and MS analysis (**Figures S5**, **S6**). Using this strategy, we identified two new sites of phosphorylation of *h*TFP_α_, the p-Tyr271 of helix-10 (**Figure 3B**) and the p-Tyr43 of the predicted signal peptide; and the p-Tyr435 of HACD already reported in the PhosphoSitePlus. This result is relevant because until now, the phosphorylation sites Tyr43 and Tyr271 and the potential kinase involved in *h*TFP_α_ phosphorylation were not described. **Figure S7** summarizes all the PTMs of *h*TFP_α_ reported up to now.

### PtpA specifically dephosphorylates the recombinant *h*TFP_α_

First, we characterized the recombinant protein *h*TFP_α_ by SEC. We determined its apparent protein molecular weight noting that *h*TFP_α_ eluted as a protein dimer (179 kDa) of two alpha subunits (**Figure 3A**). We suggest that this dimer is stabilized by hydrophobic regions of the alpha subunits, which in the biological unit of *h*TFP (*α*_2_ *β*_2_-heterotetramer) interact with the beta subunits (Xia et al., 2019). We also produced with a high degree of purity and yield, the recombinant *h*TFP_α_-Y271F mutant, which, as the *h*TFP_α_-wt protein, is a dimer in solution (**Figure S8**). For the PtpA activity assay both *h*TFP_α_ and *h*TFP_α_-Y271F were previously phosphorylated by Jak. The recombinant PtpA-wt and the inactive mutant PtpA-C11S were also produced as described previously (Margenat et al., 2015), and characterized by SEC as monomeric proteins (**Figure S9**). Then, we evaluated PtpA activity using a method previously reported (Najarro et al., 2001), based on the incubation of the phosphatase with the protein substrate previously separated by SDS-PAGE, transferred and immobilized to a membrane in a non-covalent way. After immobilization, the *h*TFP_α_ substrate was expected to recover part of its secondary structure, because the denaturing agent was removed. Using this method we verified the p-Tyr phosphatase activity of PtpA (**Figure S10A-D**). However, this strategy was not appropriate to evaluate the substrate specificity of PtpA, because the substrate did not preserve its global structure needed to a correct enzyme-substrate recognition. In this context, we observed that PtpA dephosphorylated p-Tyr residues no matter which immobilized protein was used (TFP or the viral protein OH1-C112S) (**Figure S10E**). On the other hand, carrying out the activity assay in solution presents difficulties, as a detergent is needed to maintain the recombinant *h*TFP_α_ protein in solution, since this protein is described as associated to the mitochondrial membrane (Fould et al., 2010; Liang et al., 2018; Xia et al., 2019). High concentrations of detergent inhibit PtpA and interfere with the commonly used Malachite Green reagent in the Pi detection. Thus, it was necessary to define the optimal conditions of the reaction to preserve the *h*TFP and PtpA stability during the incubation at 37°C (see methodology). In this assay we used catalytic amounts of PtpA (1.2 nM) corresponding to an E:S molar ratio of 1:5000, and observed a significant PtpA dephosphorylation of *h*TFP_α_-wt but not of *h*TFP_α_-Y271F (**Figure 3C**). This result can be explained by the absence of p-Y271 in the mutant, suggesting that this residue is the preferred target of PtpA *in vitro*. When the assay was performed with a higher concentration of PtpA (12 nM, E:S molar ratio of 1:500) the dephosphorylation of *h*TFP_α_-Y271F started to be detected (**Figure S11**). In this condition, it is not possible to avoid dephosphorylation of other p-Tyr residues that probably do not represent specific targets of PtpA. Then, to confirm the dephosphorylation site/s of the recombinant *h*TFP_α_ we performed nanoLC-MS/MS analyses. Nevertheless, despite the addition of phosphopeptide enrichment steps, we could not confidently detect phosphorylation sites in *h*TFP_α_ to perform a quantitative analysis of dephosphorylation by PtpA. More long-term research efforts to improve and optimize the detection of phosphosites in this protein are needed. Finally, to evaluate PtpA specificity, we used the recombinant *h*TFP_α_ and the inactive mutant of the viral protein OH1 (OH1-C112S) as a substrate (Segovia et al., 2017), previously phosphorylated by Jak kinase. As shown in **Figure 3D** we observed a significant decrease of the p-Tyr signal when *h*TFP_α_ was used as a substrate but not using OH1-C112S (120 nM of PtpA, E:S molar ratio of 1:50), demonstrating the specificity of PtpA for *h*TFP_α_.

### *In silico mycobacterial* PtpA and *h*TFP_α_ form a stable complex that involves PtpA active site

**Figure 4:**
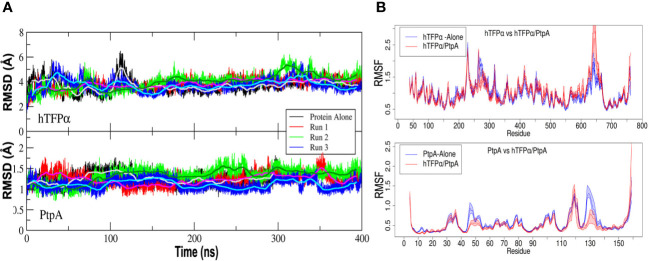
Mycobacterial PtpA and *h*TFP_α_ form a stable complex that involves PtpA active site. **(A)** Graphical representation of the Root Mean Square Deviation (RMSD) along time (nanoseconds) for three parallel molecular dynamics simulations of *h*TFP_α_ (up) and PtpA (down) proteins alone in solution, and forming part of the interacting complex (run1, run 2 and 3). **(B)** Graphical representation of the Root Mean Square Fluctuation (RMSF) of the backbone atoms along the trajectory (average over each residue) for *h*TFP_α_ (up) and PtpA (down) proteins alone in solution (blue curve) and the three simulations together (red curve). The RMSF values were calculated over a moving window of 10 ns with a step of 2 ns during the last 200 ns of simulation. Each curve is plotted from the percentile 25 to the percentile 75 of the obtained values, with the median at the center. For the calculation of the statistical values, only one trajectory was taken into account for the proteins alone, while for the complex, three trajectories were taken into account.

We have already observed by SPR studies that PtpA interacts through the active site with *h*TFP immunopurified from macrophage (Margenat et al., 2015). In the present study, we performed additional SPR studies to determine the affinity constant of the interaction of the complex, whose value was 0.31 μM (**Figure S13**), a value similar to that reported for other phosphatases and their substrates (Czikora et al., 2011). Previously to this assay, we verified that 21% of the immunopurified *h*TFP is phosphorylated on Tyr, using an anti-p-Tyr-Ab (**Figure S10A**). In addition, we performed molecular dynamics simulations (MD) to determine features of the interaction between PtpA and *h*TFP_α_, using the best complex obtained by docking described above. Three systems were considered for MD simulations in solution: PtpA, *h*TFP_α_, and the PtpA-*h*TFP_α_ complex. For the last, three sets of independent simulations (with different initial random Maxwell-Boltzmann velocities) were performed. According to the Root Mean Square Distance (RMSD) of the backbone atoms along the nanoseconds of simulation, both proteins in the complex maintained their structure stable, as they are alone in solution (**Figure 4A**). This result suggests that the complex is stable and remains formed over the time analyzed. Concerning the Root Mean Square Fluctuation (RMSF) values, we observed that *h*TFP_α_ fluctuation in the complex is similar (on average) to the protein alone. In contrast, PtpA fluctuations in the complex are reduced compared to when the protein alone is evaluated (**Figure 4B**). The last suggests a change in PtpA behavior when the complex is formed. Interestingly, the regions corresponding to the active site of PtpA have a more considerable relative decrease in their fluctuation when the complex is formed. For instance, the region that corresponds to the D-loop (Asp126) of PtpA is stabilized upon the PtpA-*h*TFP_*α*_ complex formation, probably reflecting the conformational change that allows the catalytic Asp to adopt the correct position during catalysis (Madhurantakam et al., 2008; Hobiger and Friedrich, 2015).

### Additional factors are required to explain an ubiquitin-mediated activation of PtpA

**Figure 5:**
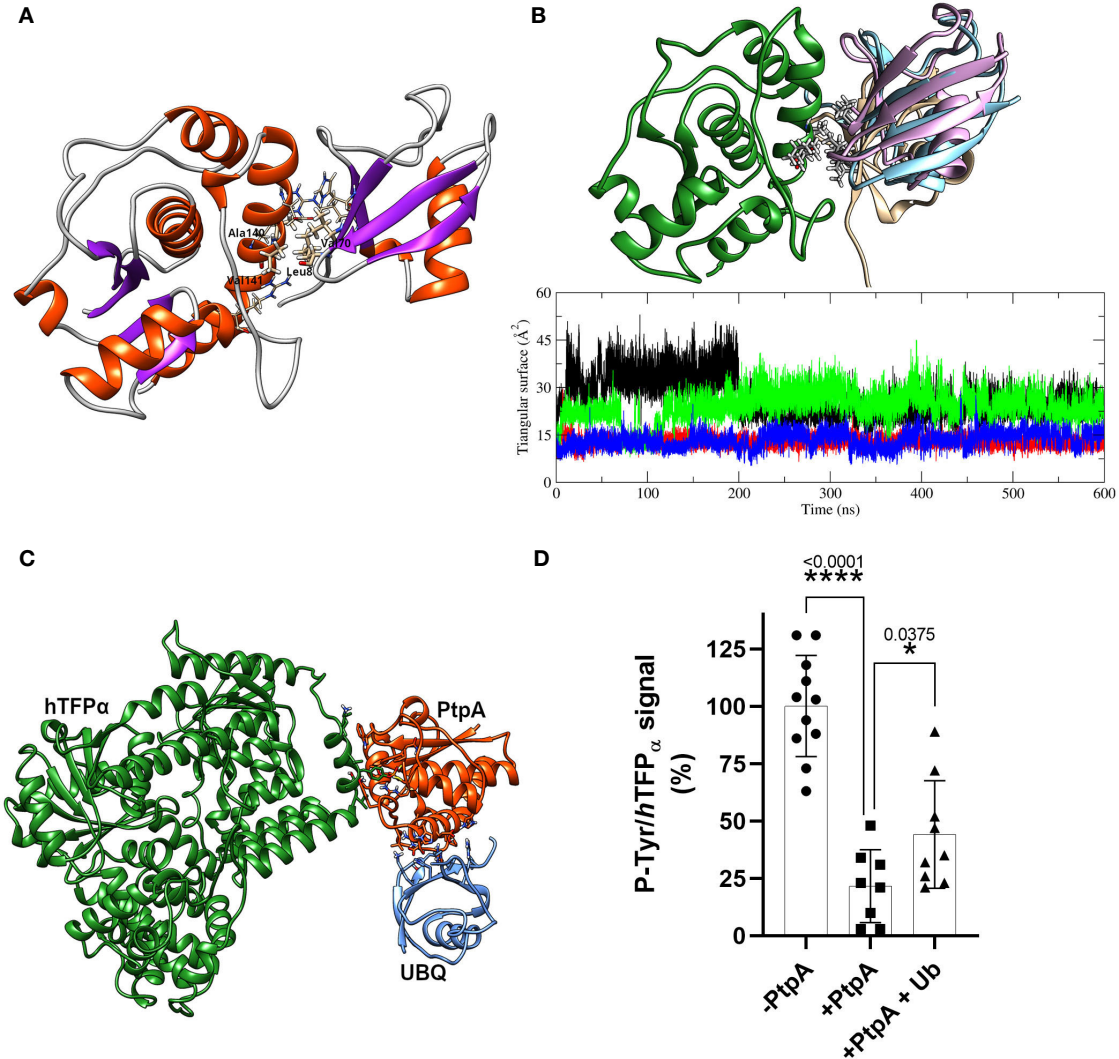
PtpA-ubiquitin interaction assays and evaluation of the ubiquitin effect on phosphatase activity. **(A)** Best PtpA/Ub complex structure obtained after the protein/protein docking protocol. The residues involved in the hydrophobic core are labeled and plotted with thicker bonds, while the residues involved in the h-bonds interactions are plotted with thinner bonds. **(B)** The upper structure represents the final configuration of the three MD runs for ubiquitin molecules (1-pink, 2- brown, and 3-light blue) with respect to PtpA (green). For the PtpA MD, only one conformation is shown since the changes in its conformation were negligible between the three runs. The graphical inset represents the triangular area of the PtpA binding site (defined in the text) along the trajectory of the simulation of PtpA in solution (black curve) and in the three simulations of the complex PtpA/Ub (1-red, 2-green, and 3-blue). **(C)** Hypothetical complex of the *h*TFP_α_/PtpA/Ub. The image shows that there are no steric interferences between proteins suggesting that the complex could form. **(D)** Dephosphorylation of recombinant *h*TFP_α_ in the presence or absence of ubiquitin. Equal amounts of the recombinant *h*TFP_α_ were resolved by SDS-PAGE, transferred to PVDF membrane and blocked, and treated at 37°C for 30 min with a buffer containing 0 μM PtpA-wt (-PtpA), 1.5 μM PtpA (+PtpA), and 1.5 μM PtpA preincubated with ubiquitin for 15 min (+PtpA and Ub). Dephosphorylation was evaluated with the anti-p-Tyr antibody. The same membranes were re-probed with anti *h*TFP_α_ antibody to determine the ratio between p-Tyr and *h*TFP_α_ chemiluminescent signals (expressed as %). Error bars represent experimental variability detected between three independent experiments, and the asterisks are the p-value obtained after an unpaired t-test.

As described in the introduction, some reports connect PtpA with the degradation pathways induced by ubiquitin. It has been reported that PtpA binds non-covalently host ubiquitin *via* a previously unknown ubiquitin-interacting motif-like (UIML) and that this interaction activates PtpA to dephosphorylate the kinase Jnk and the p38 MAPK, which leads to the suppression of innate immunity (Wang et al., 2015). In this context, we decided to characterize the ubiquitin:PtpA interaction by molecular docking and MD and perform kinetic studies to evaluate the proposed modulation of PtpA activity. The interacting configuration between ubiquitin and PtpA was determined using the HADDOCK web server (Van Zundert et al., 2016). For that, the residues from the UIML region of PtpA and the residues of ubiquitin proposed as relevant for the interaction (His 68, Ile 44, and Leu 8) were selected (Wang et al., 2015). The best result was obtained from the cluster with the lowest HADDOCK score and also the most populated one. The interacting region has a hydrophobic core (HC) composed of residues from the UIML region of PtpA (Ala 140 and Val 141) and ubiquitin residues (Leu 8 and Val 70) (indicated in **Figure 5A**), flanked by hydrogen bond interacting residues, Glu 143 (PtpA) with Arg 42 (ubiquitin), Glu 137 (PtpA) with His 68 (ubiquitin), and Arg 111 (PtpA) interacting with the backbone oxygen of Leu 8 (ubiquitin). To test the stability of the complex, we performed three parallel MD simulations using as a starting point the interacting complex described above. The MD results show that the HC is stable and remains conserved in all three simulations while the surrounding hydrogen bonds form, break down, and change over time. In particular, the final configuration of the complex is different in the simulations, as shown in **Figure 5B**. In the first MD, the ubiquitin rotates around the HC and forms different H-bonds in comparison with the starting point during the MD simulation. In the second case, the interactions between both proteins are stable and the same as at the beginning of the MD. Finally, in the third MD the ubiquitin moves and rotates over the HC, changing all H-bonds interaction in comparison with the initial configuration and the relative position of the Val 70 residue; nevertheless, the HC is still formed. This result suggests that ubiquitin can interact with PtpA during the simulation, albeit with nonspecific interactions and with the HC anchoring the interaction. Furthermore, the RMSD along the trajectory for both proteins of the complex shows values of less than 3 Å (**Figure S14A**), similar to the values for the proteins alone in solution, implying that both keep their respective internal conformation along the trajectory, without additional stabilization.

To check whether ubiquitin could affect PtpA activity, we evaluated putative changes in the PtpA active site during the interaction. To this end, we performed a simple geometric approach, in which the surface of the triangle formed by three relevant heavy atoms of the PtpA active site was followed along the trajectory (S atom of Cys 11 and Cys 16 together with a C atom of the Asp 126 lateral chain) when PtpA is alone or interacting with ubiquitin (**Figure 5B**). This figure shows two possible and stable values for the calculated triangular surface, meaning two possible conformations of the PtpA active site: one with a higher area reflecting an open conformation and the other with a lower area indicating a closed conformation. In the case of the MD simulation of PtpA alone, and only one trajectory of the complex, we found an open conformation, while in two other MDs of the complex, a close conformation was found (**Figure S14B**). However, no correlation between the final position of ubiquitin in the complex and the conformation of the PtpA active site was found, explaining the reported PtpA activating effect of the ubiquitin. In addition, we experimentally tested the impact of ubiquitin on PtpA activity using good-quality recombinant ubiquitin and active PtpA proteins (**Figure S15A**). Contrary to what was previously reported (Wang et al., 2015), we did not detect activation of PtpA acting on the artificial substrate pNPP. We observed the same result, even varying the PtpA:ubiquitin molar ratio from 1:0 to 1:10 (**Figure S15B**). Considering that the authors used the PtpA linked to the GST-tag and we used PtpA linked to a His-tag, we evaluated whether the discrepancy could be due to the His-tag used. Even after removing the tag, no activating effect of ubiquitin on PtpA phosphatase activity was observed (**Figure S15C**). Thus, our discrepancy with the data reported by Wang et al., 2015 could be due to the distinct fusion protein used. Overall, our *in silico* and experimental results suggest that the interaction of ubiquitin with PtpA cannot explain the reported PtpA activation. However, we cannot discard that additional factors may be required to achieve this effect. In parallel, we also analyzed whether the interaction of PtpA with ubiquitin can occur in the context of the PtpA-*h*TFP_*α*_ complex. This analysis could be interesting because *h*TFP_*α*_ has several ubiquitylation sites noted in the database Phosphosite (Hornbeck et al., 2015), and we demonstrated that it co-purifies with the E3 ubiquitin ligase (TRIM21, **Figure S4A**), which could connect *h*TFP_α_ with the Ub-mediated proteasomal degradation. **Figure 5C** shows that PtpA could form the protein complex with ubiquitin without steric clashes. However, our study did not detect activation of PtpA by ubiquitin acting on *h*TFP_α_, after three independent assays (**Figure 5D**). As shown in the graph, we observed a significant decrease in P-Tyr/*h*TFP_α_ signal level after incubation with PtpA in the absence or presence of Ub. However, in the case of the assay with PtpA+Ub, this decrease was lower than without Ub. This suggests an inhibitory effect of ubiquitin on the phosphatase activity of PtpA acting on *h*TFP_α_ as a substrate, consistently with the detection of an alternation of the active site between an open (active) and closed (inactive) conformation during PtpA/ubiquitin MD simulation (**Figure S14B**). Further studies using the recombinant TFP_α/β_ heterotetramer as a substrate will be interesting to determine if the inhibitory effect of the ubiquitin is maintained.

## Conclusion

**Figure 6:**
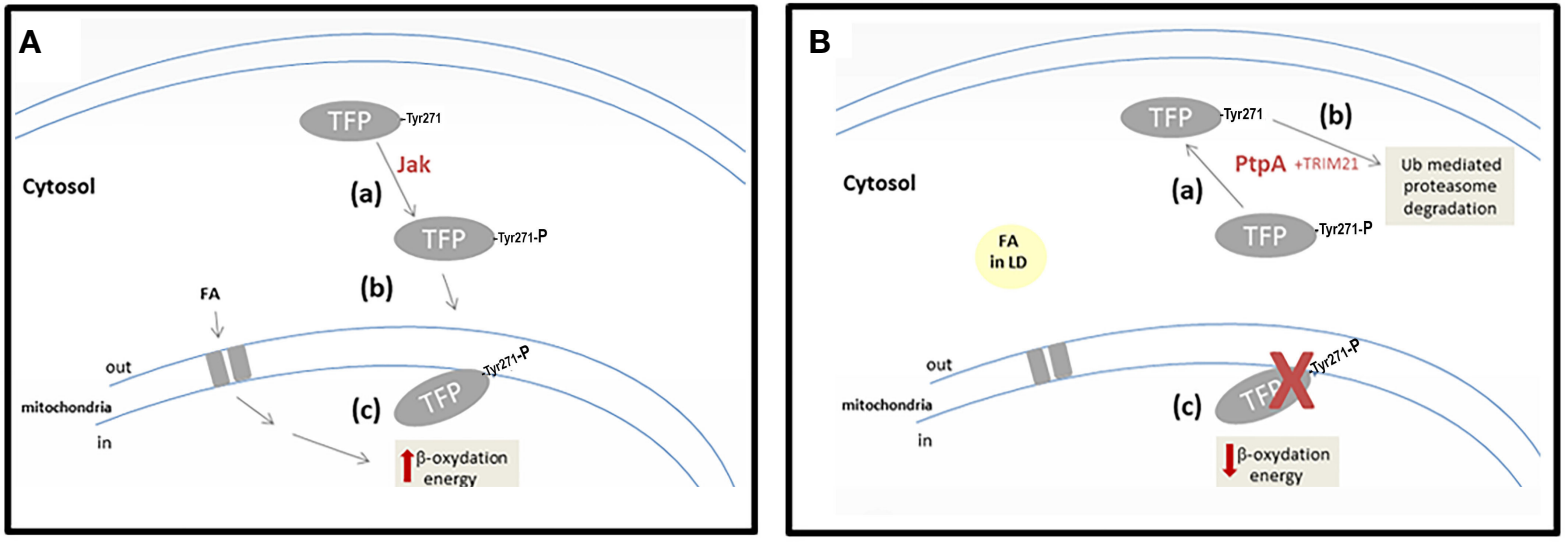
Scheme showing our working hypothesis. **(A)** A hypothetical scenario in the cells without *Mtb* infection. Step-a corresponds to the phosphorylation of *h*TFP_α_ by a cellular kinase (maybe Jak). Step-b corresponds to the correct localization of *h*TFP_α_, in the mitochondrial inner membrane. Step-c represents the activity of *h*TFP_*α*_, in the *β*-oxidation of the long-chain fatty acids transported to the mitochondria. **(B)** A hypothetical scenario in the cells infected with *Mtb*. Step-a corresponds to the dephosphorylation of TFP by mycobacterial PtpA. Step-b corresponds to the Ub-mediated proteasomal degradation stimulated by *h*TFP-TRIM21 interaction. Step-c represents *h*TFP_α_ decrease in mitochondria, with a concomitant accumulation of long-chain fatty acids into LD in the cytosol of the cells.

Our results allow advancement in the characterization of *Mtb* PtpA interaction and activity on the alpha subunit of *h*TFP, a key enzyme of lipid metabolism. These lead us to suggest that the p-Tyr271 of *h*TFP_α_ is the potential target of mycobacterial PtpA, located in a conserved helix relevant to its localization and activity. Our working hypothesis, integrating our results with those already published, was included in **Figure 6**.

In cells, *h*TFP_α_ is synthesized by cytosolic ribosomes, like most mitochondrial proteins, and then it is translocated to the inner mitochondrial membrane (Bykov et al., 2020). Despite *h*TFP_α_ being a central protein of the metabolism and having several PTMs, including phosphorylation (**Figure S7**), its role is unknown. Considering that PTMs have an important role in regulating cell functions (Ribet and Cossart, 2010), it is appropriate to think that specific phosphorylation of *h*TFP can modulate its cellular localization. In the present work, we demonstrated *in vitro* that *h*TFP_α_ could be phosphorylated by Jak in three residues, two of them (p-Tyr271 of helix-10 and p-Tyr43 of the predicted signal peptide) located in important sites of the protein that could define its cellular localization. The p-Tyr271 of helix-10 from the alpha subunit was described as relevant for *h*TFP_α,_ anchor to the mitochondrial membrane, and for its activity since it contributes to the formation of the channels that connect the different active sites of the enzyme (Xia et al., 2019). In this context, we suggest that the tyrosine phosphorylation of *h*TFP_α_ by a cellular kinase (possibly Jak) defines its correct localization in the mitochondrial membrane (**Figure 6A**, steps a and b). Once located in the mitochondria *h*TFP_α/β_ plays a central role in the β-oxidation of long-chain fatty acids, catalyzing three of the four stages of this pathway (Eaton et al., 2000). Indeed, oxidation of the long-chain fatty acids, transported from the cytosol to the mitochondria, contributes directly (by the generated NADH and FADH_2_) and indirectly (by the acetyl-CoA) to the mitochondrial ATP synthesis (**Figure 6A**, step c).

On the other hand, during *Mtb* infection, the phosphatase PtpA is introduced to the cytosol of the macrophages, as previously demonstrated (Bach et al., 2008). In this location, PtpA could dephosphorylate *h*TFP_α_, as demonstrated *in vitro* by our group. As a consequence, we suggest that the dephosphorylated *h*TFP_α_ will not be able to reach the mitochondria (**Figure 6B**, step a). This would explain why *h*TFP_α_ is no longer detected in the mitochondria of macrophages infected specifically with the virulent *Mtb* H37Rv (Jamwal et al., 2013). In this experiment the authors did not describe changes in the mitochondria levels of *h*TFP_β_ after infection with the virulent *Mtb* H37Rv, and in the levels of both *h*TFP subunits after infection with the avirulent *Mtb* strain. Despite these observations, in an *in vitro* PtpA activity assay, using immunoprecipitated TFP from macrophages, we observed a reduction in the phosphorylation levels of both subunits (Margenat et al., 2015). In addition, there is evidence that during *Mtb* infection PtpA also reaches the nucleus of host cells and regulates the expression of genes involved in host innate immunity or in cell proliferation and migration (Wang et al., 2017). The PtpA nuclear localization, could contribute to explain the observed 10-fold decrease in the *h*TFP_α_ mRNA observed during the infection with the virulent *Mtb* H37Rv (Jamwal et al., 2013). The relevance of this result needs to be evaluated in future studies of infected macrophages with *Mtb*.

Furthermore, *h*TFP_α_ has several ubiquitylation sites noted in the database Phosphosite (Hornbeck et al., 2015), and we demonstrated by MS that *h*TFP_α_ from macrophages co-purify with the E3 ubiquitin ligase TRIM21 (**Figure S4**). This enzyme could be responsible for *h*TFP_α_ ubiquitylation, connecting TFP with the Ub-mediated proteasomal degradation (**Figure 6B**, step b) and the metabolic modulation. Similarly, it has been observed that TRIM21 is involved in the downregulation of glycolysis, *via* proteasomal degradation of the rate-limiting metabolic enzyme phosphofructokinase (PFK) (Chen et al., 2021). This is an enzyme that catalyzes a key regulatory step of glycolytic metabolism, which was also identified by our group as another of the potential substrates of PtpA (Margenat et al., 2015). A drop in mitochondrial *h*TFP_*α*_ level (**Figure 6B**, step c) could contribute to explaining some of the metabolic changes described during mycobacterial infection (Cumming et al., 2018; Genoula et al., 2018) such as decreased mitochondrial activity and changes in lipid metabolism as a cytoplasmic accumulation of lipid droplets (**Figure 6B**, step d). The storage of lipids during infection can act as a mechanism that allows reestablishing a cellular balance, avoiding the death of the macrophage, and in turn, favoring the persistence of the pathogen (Laval et al., 2021). With respect to how PtpA activity is regulated during infection, we think that in view of our results and our hypothesis, more studies must be carried out to understand the potential regulatory role of ubiquitin and other relevant molecules as fatty acids.

Altogether, we suggest that the role of this virulence factor is to promote the survival of mycobacteria in infected cells, affecting not only the innate immunity (Bach et al., 2008; Wang et al., 2017, 2015; 2020) and apoptosis (Poirier et al., 2014), but also macrophage pathways involved in lipid metabolism. Future studies will be performed using cellular models to evaluate whether potential changes in macrophage metabolism can be correlated with PtpA activity on TFP.

## Materials and methods

### Molecular docking: PtpA/*h*TFP_α_

The following protocol was used to select which tyrosine may be phosphorylated in the *h*TFP_α_ protein, in addition to those reported in the PhosphoSitePlus^®^ (Hornbeck et al., 2015), and acts as putative interacting residue with mPtpA when *h*TFP_α_ is its substrate. The protocol was performed for each tyrosine in *h*TFP_α_ located at the surface of the protein, and it consists of three steps involving (i) Selection of the putative phosphotyrosine on the *h*TFP_α_, (ii) docking simulations, and (iii) electrostatics calculations. (i) Selection of putative phosphotyrosine on *h*TFP_α_. The selected pdb structure for *h*TFP_α_ was the only X-ray structure available in the PDB databank (Berman et al., 2000), pdb code 6DV2, with a resolution of 3.6 Å (Xia et al., 2019). The putative tyrosines from this structure that could be phosphorylated were selected based on their exposure to the protein’s surface. The selected tyrosines, with at least 40% of their surface exposed to solvent in the pdb structure, were Y43, Y239, Y271, Y435, Y637, Y639, Y724, and Y762. Each of these residues was phosphorylated *in silico* by hand one by one, adding the necessary atoms to the tyrosine residue. Energy minimization of the complete protein structure was then performed in implicit solvent using the Amber package of programs (Pearlman et al., 1995) with the amber ff14sb force field (Maier et al., 2015). The force field parameters for the phosphotyrosine were taken from the publication by Homeyer (Homeyer et al., 2006). The implicit solvent was taken into account using the modified Generalized Born model developed by A. Onufriev et al. (Onufriev et al., 2004, 2000) as implemented in the amber program (Case et al., 2005). Finally, 8 different *h*TFP_α_ structures were obtained, each with one phosphotyrosine. (ii) *Docking simulations:* The HADDOCK program (Dominguez et al., 2003; Van Zundert et al., 2016) was used for the molecular docking simulations between PtpA and *h*TFP_α_ with one phosphotyrosine. The conformation of the PtpA protein was taken from the X-ray structure with a resolution of 1.9 Å with the pdb code 1U2P (Madhurantakam et al., 2005). The active residues at the protein interface for docking simulation were defined as the catalytic residues Cys 11, Cys 16, and Asp 126. For *h*TFP_α_, each phosphorylated tyrosine was treated as an active residue. The passive residues for both proteins were defined as all surface residues within 6.5 Å from the active residues on each protein. Different numbers of clusters were obtained for each docking simulation. Results, around 200 different complexes for each docking simulation were clustered according to the HADDOCK protocol (Dominguez et al., 2003; Van Zundert et al., 2016). (iii) *Electrostatic calculations*: Since the HADDOCK score cannot be used to compare the results of the *h*TFP_α_ protein with different phosphorylated tyrosines, but only to compare different solutions with the same phosphorylated tyrosines for a given complex (Kastritis and Bonvin, 2010), the electrostatic contribution to the free energy of binding of the interaction between PtpA and *h*TFP_α_ was used as a criterion for selecting the best representative complex. Therefore, the complex with the lowest electrostatic energy of binding was selected as the best representative. The electrostatic energy was calculated by solving the nonlinear Poisson-Boltzmann equation for the complex and each protein using APBS 3.2.0 software (Jurrus et al., 2018), using a dielectric constant for points within and outside the protein of 2.0 and 78.5 D respectively. The electrostatic energy of binding (E) was then calculated as the difference between the electrostatic energy of the complex, composed of the *h*TFP_α_ and PtpA proteins, and the electrostatic energy calculated for both *h*TFP_α_ and PtpA proteins individually, E=E*h*TFP_α_+PtpA-(EPtpA+E*h*TFP_α_). We consider a negative value of E as an indicator that the complex is formed and may be stable.

### Molecular dynamics simulations

All MD simulations were performed using the Amber package version 2018 (Case et al., 2005). Parameters from the Amber ff14SB force field were used for all protein residues (Maier et al., 2015); while the parameters for the phosphotyrosine were taken from the literature (Homeyer et al., 2006). Periodic boundary conditions were taken into account in all simulations. A weak-coupling algorithm (Berendsen et al., 1984) was used to couple the simulation boxes with an isotropic pressure of 1 atm and a reference temperature of 300 K. Relaxation times were chosen to be 5 ps and 2 ps for pressure and temperature coupling, respectively. All bonds involving hydrogen were constrained using the SHAKE algorithm (Hess et al., 1997). The time step for all simulations was set to 2 fs. All systems were immersed into a truncated octahedron of TIP3P water molecules (Price and Brooks, 2004) with a minimum distance between the edges of the simulation box and the protein surface atom of 11 Å. The bonds of the water molecules were constrained using the SETTLE algorithm (Miyamoto and Kollman, 1992). The complete systems were neutralized with counter-ions. A direct cutoff for nonbonded interactions of 10 Å and the particle mesh Ewald for long-range electrostatics were applied (Essmann et al., 1995). A standard protocol was used for all MD simulations, and it begins with two initial energy minimizations: the first one only for the solvent molecules, constraining the position of all non-solvent atoms, and a second minimization for the entire system. In order to equilibrate the systems at 300 K, each of them was slowly heated from 100 K to 300 K over a period of 1 ns under NVT conditions, followed by MD simulations at 300 K up to 100 ns. Subsequently, a production run of 400 ns or 500 ns was performed for each complex under NPT conditions. For each simulated system, different numbers of parallel runs were performed with different random initial velocities in order to have more statistics.

### Phylogenetic analyses

The human trifunctional enzyme (NCBI accession: NP_000173.2) was used as a query for a blastp search against the NCBI RefSeq database (e-value: 1e-5, number of hits: 5000). Hits were manually filtered to those containing “trifunctional” in their descriptions. When more than one isoform was available, the longest one was maintained. Hits were further filtered to those with a query coverage greater than 50%. Sequences were submitted to NGPhylogeny.fr (Lemoine et al., 2019) for alignment with MAFFT (Katoh and Standley, 2013), alignment curation with BMGE (Criscuolo and Gribaldo, 2010), evolutionary model selection, and phylogenetic analysis with SMS (Lefort et al., 2017) and PhyML (Guindon et al., 2010), respectively. Trees were visualized and annotated using ITOL (Letunic and Bork, 2021).

### Production of recombinant PtpA-wt and PtpA-C11S

The plasmid pET28 containing the sequence of PtpA was obtained as described previously (Margenat et al., 2015). The construct expressing the single PtpA mutant C11S was obtained by site-directed mutagenesis (Quikchange Site-Directed Mutagenesis kit, Stratagene) using the following primers forward and reverse, respectively: 5´ -TTC GTT TCT ACG GGC AAC ATC TG-3, -5´- CA GAT GTT GCC CGT AGA AAC GAA - 3´. The sequence was verified by DNA sequencing (Macrogen). Expression was done as described previously (Margenat et al., 2015). Briefly, Transformed *E. coli* BL21 (DE3) cells were grown at 15°C in LB medium with 50 *μ*g ml^-1^ kanamycin, and protein synthesis induced with 0.5 mM isopropyl β-D-thiogalactoside (IPTG). The recombinant proteins were purified to homogeneity by metal-affinity (Cu-column) and size exclusion chromatography (SEC).

### Immunoprecipitation of *h*TFP from macrophages

The *h*TFP_*α*/*β*_ was obtained from macrophage extracts by immunoprecipitation with an anti-TFP monoclonal antibody. Briefly, the anti-TFP MAb (8*μ*g, ab110302, MitoSciences) was first covalently cross-linked to anti-mouse IgG Ab on the beads (100 *μ*l, 11201D, Life technologies) using BS3 (Sigma). Then, beads were washed and incubated with 500 *μ*g of macrophage extract, prepared as previously described (Margenat et al., 2015). Beads were washed, and bound proteins were eluted with 50 mM citrate pH 2.6 (2x100 *μ*l), and neutralized immediately. Samples were resolved by SDS-PAGE, stained with colloidal Coomassie, or transferred to PVDF membranes for 1 h at 100 V. Membranes were blocked for 16 hs at 4°C, washed twice with TBS-T, and subsequently incubated with anti-*h*TFP_α_ MAb (ab200652) at 1/1000 dilution in TBS-T, 1.5h at RT. After washing, membranes were incubated (1 h at RT) with an anti-rabbit antibody conjugated with horseradish peroxidase (HRP) (1/50000, Sigma-Aldrich 0545). After four washes with TBS-T and one wash with TBS, the reaction was developed with Pierce ECL western blotting substrate (Thermo Scientific). Immunoreactive bands were visualized using the GBOX ChemiSystem tool (SynGene). The presence of the TFP was confirmed by mass spectrometry (MS).

### Production of recombinant *h*TFP_α_ and the mutant *h*TFP_α_ -Y271F

The *h*TFP_α_-wt (ECHA sequence) was sub-cloned by RF-cloning (Unger et al., 2010), in a modified pET32a vector (named pT7), carrying an ampicillin-resistance cassette, an N-terminus His-tag sequence, and the tobacco etch virus (TEV) protease recognition site between this tag and the target gene insertion site (Correa et al., 2014). As a template the ECHA sequence already cloned in the pMyr-plasmid was used. The following forward and reverse primers were used, respectively: 5’-GGATCGGAAAAC CTGTATTTTCAGGGATCCACCAGAACCCATATTAACTATGGAG-3’ and 5’-GAACTGCGGGTGGCTCCAGCTGCCGGATCCTCACTGGTAGAACTTCTTGTTAGG-3’. PCR were done using Phusion Polymerase (Thermo), and the PCR conditions were: a denaturing step at 98°C for 30 s and 35 amplification cycles of 98°C for 10 s, 65°C for 30 sec and 72°C for 1 min 20 sec, with a final extension step at 72°C for 10 min. The obtained products (megaprimer) were analyzed by agarose gel electrophoresis, purified with GeneJET Extraction Kit (Thermo) and quantified in a nanodrop spectrophotometer. The generated megaprimers contained 30 bp in both ends that overlaps with the insertion site in the destination vector T7. The integration into the vector was done by performing a second PCR reaction using 200 ng megaprimer, 30 ng pT7 vector, and the PCR conditions as follows: a denaturing step at 98°C for 30 s, 30 amplification cycles of 98°C for 10 s, 60°C for 1 min and 72°C for 5 min, with a final extension step at 72°C for 7 min. 20 μl of the PCR product was the treated with 20 U of DpnI (Thermo) for 2 hours at 37°C to selectively degrade the methylated parental vector, and 20 min at 80°C to inactivate the enzyme. Then, 1μl was used to transform 50 μl of electrocompetent XL-1 *E. coli* cells or *E. coli* BL21 (DE3). Plasmids were purified using the GeneJET Plasmid Miniprep (Thermo) and the sequence verified by DNA sequencing (Macrogen).

Transformed *E. coli* BL21 (DE3) were grown overnight in 10 ml of Lysogeny broth (LB) containing 100 *μ*g ml^-1^ ampicillin at 37°C. For protein expression, 4 ml of overnight culture was transferred into 800 ml of LB with ampicillin and grown at 37°C to an OD_600_ of 0.6-0.7. Protein synthesis was induced by adding 0.5 mM IPTG at 19°C for 24 h. Cells were then harvested by centrifugation and lysed by sonication on ice in the lysis buffer (100 mM Tris, pH 8.0, 200 mM NaCl, 10% glycerol, containing EDTA-free protease inhibitor cocktail (Amersham Biosciences). The suspension was supplemented with 1.5% Tween 20 and incubated for 90 min at 4°C with gentle agitation. Cell debris was removed by centrifugation at 15000 xg for 40 min, and the supernatant was diluted with 100 mM Tris, pH 8.0, 200 mM NaCl, and 10% glycerol to obtain a final concentration of 0.5% Tween 20. Then, imidazole was added to the supernatant to a final concentration of 10 mM and incubated with the Ni-NTA resin (GE Healthcare) equilibrated in binding buffer (100 mM Tris pH 8, 200 mM NaCl, 10% glycerol, 10 mM imidazole, and 0,5% Tween 20), for 1 hour with gentle shaking. The mixture was then packed into a column, washed with binding buffer, and the recombinant protein was eluted with elution buffer (100 mM Tris pH 8, 200 mM NaCl, 10% glycerol, 300 mM imidazole, and 0.5% Tween 20). Imidazole was rapidly extracted by using a PD-10 column equilibrated in 50 mM Tris pH 8, 100 mM NaCl, 5% glycerol, and 0.5% Tween 20. The protein was further purified by SEC using a Superdex 200 16/60 preparative grade column (GE Healthcare) previously equilibrated in SEC-buffer (50 mM Tris-HCl pH 8.0, 100 mM NaCl, 5% glycerol, 0.1% Tween 20). Fractions containing purified *h*TFP_*α*_ were pooled and concentrated using an *Amicon Ultra-15 Millipore* concentrator to 1 mg/mL. The 6xHis-tag was removed with TEV protease in a 1:20 ratio overnight at 18°C, and then the TEV was removed using a Ni-NTA resin. The identity of the recombinant *h*TFP_α_ was confirmed by MS. Two batches of recombinant protein were produced and used in the present study.

For the production of the *h*TFP_α_-Y271F mutant, we used as a template the plasmid containing the sequence of the *h*TFP_α_-wt, and the mutation introduced by PCR site-directed mutagenesis using the following forward and reverse primers, respectively: 5’ - GAA AAA TTG ACA GCG TTT GCC ATG ACT ATT C -3’, and 5’ - G AAT AGT CAT GGC AAA CGC TGT CAA TTT TTC -3’. The presence of the mutation was verified by DNA sequencing (Macrogen). Recombinant *h*TFP_α_-Y271F was expressed in *E. coli* BL21 (DE3), following the same procedure described for the wt protein. The only difference was that the 6xHis-tag was removed with TEV protease before SEC purification, and not after this chromatography as described for the WT.

### Phosphorylation of *h*TFP_α_ and OH1-C112S by Jak

The recombinant *h*TFP_α_-wt, *h*TFP_α_-Y271F, and OH1-C112S produced as reported previously (Segovia et al., 2017), were phosphorylated with commercial Jak-1 kinase (Thermo Fisher Scientific) in the SEC-buffer supplemented with 2.5 mM MnCl_2_, 7.5 mM MgCl_2_, 1 mM DTT, and 1 mM ATP as substrate for 2 hours at 30°C. An enzyme:substrate (E:S) molar ratio of 1:3000 was used. Then, Jak-1 was removed by purification with glutathione agarose (LifeTech) and ATP was removed by SEC (PD-10, GE). The phosphorylated proteins were then used in PtpA activity and MS assays.

### PtpA activity assays on the recombinant *h*TFP_α_

To evaluate PtpA activity in solution using the recombinant proteins (*h*TFP_α_-wt or *h*TFP_α_-Y271F, OH1-C112S) we first phosphorylated them with Jak-1 as described above. The buffer of the proteins was changed by SEC to the activity buffer (50 mM Tris-HCl pH 8.0, 50 mM NaCl, 5% glycerol, 3 mM EDTA, 1 mM DTT and 0.1% Tween 20). To avoid PtpA inactivation and interference of the detergent during MS identification of the phospho-peptides (Feist & Hummon, 2015) it was not possible to increase the concentration of detergent more than 0.1% Tween 20. To preserve both proteins in solution the maximal incubation time at 37°C was 30 min. The E:S molar ratios used were 1:5000, 1:500 and 1:50. After 30 min, spots of 5 μL of the reaction were applied in a nitrocellulose membrane by triplicates and then the membrane was dried and blocked with membrane blocking solution (Invitrogen #00-0105). Afterward, membranes were washed in TBS-T and probed for p-Tyr levels with anti-p-Tyr antibody (Cell Signalling #9411) at 1/2000 dilution in TBS-T, ON at 4°C. Blots were then incubated with horseradish peroxidase (HRP)-linked anti-mouse (Sigma-Aldrich A4416, 1/10000) secondary antibody for 1 h at RT. After four washes with TBS-T, and one wash with TBS, the reaction was developed with Pierce ECL western blotting substrate (Thermo Scientific). The chemiluminescent signals of the bands were visualized using the GBOX ChemiSystem tool (SynGene). For the assays using *h*TFP_α_-wt or *h*TFP_α_-Y271F as substrate, the membrane was reprobed with anti *h*TFP_α_ antibody (Abcam ab200652) to determine the ratio between p-Tyr and *h*TFP_α_ chemiluminescent signals, quantified using ImageJ (Schneider et al., 2012).

### Mass spectrometry identification

The samples were separated 1.5 cm by SDS-PAGE and stained with colloidal Coomassie Brilliant Blue G-250. Each lane was excised into 4 slices that were destained by incubation with 0.2 M ammonium bicarbonate/ACN (1:1) for 1 h at room temperature with agitation. After this, proteins were reduced with 10 mM DTT at 56°C for 60 min and then alkylated with 55 mM iodoacetamide at room temperature for 45 min and in darkness. Then, in-gel proteolytic digestion and peptide extraction were performed as described earlier (Gil et al., 2019). To verify the identity of the *h*TFP_α_ immunopurified or produced as recombinant protein and for p-Tyr identification, online MS detection/analysis was carried out in a nano-HPLC (UltiMate 3000, Thermo) coupled to a hybrid quadrupole-orbitrap mass spectrometer (QExactive Plus, Thermo). Peptide mixtures were loaded on C18 columns and separated using a two-solvent system (solvent A: 0.1% formic acid in water and solvent B: 0.1% formic acid in acetonitrile) with a gradient from 0% to 90% of B) and a flow rate of 0.2 mL/min over 100 min. Peptide analysis was performed in a Q-exactive Plus (Q-Orbitrap, Thermo) associated with a Nano HPLC. Xcalibur 2.1 was used for data acquisition of a full MS scan in the positive ion mode with m/z between 200 and 2000 m/z. Sequential fragmentation of the ten most intense ions with a normalized collision energy of 35, an isolation width of 2 m/z. The activation Q was set at 0.25, the activation time at 15 ms, and a dynamic exclusion time of 30 s. MS source parameters were set as follows: 2.3 kV electrospray voltage and 260°C capillary temperature. PatternLab V (Version 5.0.0.109) (Santos et al., 2022) was employed to generate a target-decoy database using sequences from *E. coli* and *h*TFP_α_, PtpA, Jak, downloaded from the UniProt. In addition, 127 common mass spectrometry contaminants were included (Carvalho et al., 2016). The Comet search engine was operated using the following parameters: trypsin as a proteolytic enzyme with full specificity; oxidation of Met and phosphorylation on Tyr as variable modifications, carbamidomethylation of Cys as fixed modification; and 35 ppm of tolerance from the measured precursor m/z. XCorr and Z-Score were used as the primary and secondary search engine scores, respectively. Peptide spectrum matches were filtered using the Search Engine Processor (SEPro), and acceptable false discovery rate (FDR) criteria were set at ≤1% at the protein level and ≤2% at the peptide level.

To improve the identification of the phospho-peptides by MS, the phosphoproteins contained in one mg of recombinant *h*TFP_α_ were purified using the Pierce Phosphoprotein Enrichment kit (Thermo). This sample was used in the phosphatase assay (1 h at 37°C) without or with PtpA. Then the proteins were treated with sequencing-grade trypsin (0.25 μg, 3h or ON at 25°C), and the detergent was removed using a specific resin (Pirce#87780). Prior to MS analysis samples were desalted using C18 reverse phase micro-columns (Omix^®^Tips, Varian), dried by vacuum, and resuspended at 1 µg/ul in 0.1% formic acid (v/v) in water. Samples were injected into a nano-HPLC (UltiMate 3000, Thermo) coupled to a hybrid quadrupole-orbitrap mass spectrometer (QExactive Plus, Thermo), and separated and analyzed as described above.

## Data availability statement

The data presented in this study are deposited in the ProteomeXChange and accession number PXD038119.

## Author contributions

AV participated in the conception and supervision of the work. AV and. FH contributed to the experimental design and analyzed the data. MM, and GB, performed the main *in vitro* experiments of PtpA activity on *h*TFPα protein substrate; TG-C and VI produced the recombinant PtpA proteins and performed the PtpA activity assay using the artificial substrate pNPP, and evaluating the effect of ubiquitin. MM and MP prepared samples and analyzed the data of MS. MM and FC performed and analyzed the SPR experiments. AC performed the phylogenetic sequences analysis. FH performed all in silico studies, with the participation of V. Irving. AV and FH wrote the paper, and MM participated in manuscript writing and revision. All authors contributed to the article and approved the submitted version.
