# Supplementary material for: Characteristics of Mycobacterium tuberculosis PtpA interaction and activity on the alpha subunit of human mitochondrial trifunctional protein, a key enzyme of lipid metabolism
Source: Front Cell Infect Microbiol. 2023 Jun 22;13:1095060. doi: 10.3389/fcimb.2023.1095060 (PMC10325834; doi:10.3389/fcimb.2023.1095060)
Supplement: Supplementary file 1 [file DataSheet_1.pdf]

ECHA_HUMAN

```
                                                    1        10        20        30        40
ECHA_HUMAN                        ..................MVACRAIGILSRFSAFRILRSRGYICRNFTGSSALLTRTHIN
tr|A0A4R6XLA8|A0A4R6XLA8_9GAMM    ...........................................................MAYYT
tr|A0A6A0IJT2|A0A6A0IJT2_9BACT    ...............................................MSEPN..AGKSLR
tr|A0A7V3QZU7|A0A7V3QZU7_9BACT    ...........................................................MGAIR
tr|A0A7V4SYJ6|A0A7V4SYJ6_9BACT    ...........................................................MNPVR
tr|A0A2A8D2N6|A0A2A8D2N6_9BACT    .......................................MQTTTAATRSIDL..ETDLLT
tr|A0A2N1TGP0|A0A2N1TGP0_9SPIR    ...............................................MNSTI..KKKYIE
tr|A0A1G3QGW4|A0A1G3QGW4_9SPIR    ...........................................................MKHIA
tr|A0A1G3QTY3|A0A1G3QTY3_9SPIR    ...........................................................MKNIT
tr|A0A7X9L359|A0A7X9L359_9DELT    ...........................................................MKNLS
tr|A0A522CJY0|A0A522CJY0_9SPIR    ...........................................................MKHIS
tr|A0A2N1RRJ3|A0A2N1RRJ3_9SPIR    ...........................................................MKYLT
tr|A0A2N2KFN7|A0A2N2KFN7_9DELT    ...........................................................MNNLA
tr|A0A1V6AXX9|A0A1V6AXX9_9DELT    ...........................................................MKYLA
tr|A0A2N2HZL0|A0A2N2HZL0_9DELT    ...........................................................MKYLT
tr|A0A5E8ARP3|A0A5E8ARP3_9BACT    ...............................................MASKA..KNSFLT
tr|A0A662A7B1|A0A662A7B1_9BACT    ............................................................
tr|D7CV16|D7CV16_TRURR            .....................................................MELRFFR
tr|A0A3C2AKX4|A0A3C2AKX4_9FLAO    .....................................................MKNKYVE
tr|A0A3B8ZLN3|A0A3B8ZLN3_9PLAN    .....................................................MKNEYFE
tr|A0A345UGV8|A0A345UGV8_9BACT    .........................................................MLT
tr|A0A6C1P6A2|A0A6C1P6A2_9BACT    .................................................MDT..KTNYLR
tr|A0A3M1X9W8|A0A3M1X9W8_9BACT    ...........................................................MKFTE
tr|A0A354C796|A0A354C796_9DELT    .................................................MEA..VMPYGQ
tr|A0A3M1NUM8|A0A3M1NUM8_9BACT    ...........................................................MPYTT
tr|A0A3M2KRC8|A0A3M2KRC8_9BACT    ...........................................................MAYTR
tr|A0A5S9IHR0|A0A5S9IHR0_9BACT    ................................................MSNA..ESKDVI
tr|A0A6M1ST81|A0A6M1ST81_9BACT    ...........................................................MSYLD
tr|A0A521AAE5|A0A521AAE5_9BACT    ...........................................................MSYLS
tr|A0A1M4ZMD6|A0A1M4ZMD6_9BACT    ...........................................................MSYLS
tr|A0A2A2GCS2|A0A2A2GCS2_9BACT    ...........................................................MSYLN
tr|A0A5D3YIH7|A0A5D3YIH7_9BACT    ...........................................................MSYLS
tr|A0A6M1T9G0|A0A6M1T9G0_9BACT    ...........................................................MSYLS
tr|A0A6A8Q1Y8|A0A6A8Q1Y8_9BACT    ...........................................................MSYLN
tr|A0A521BVJ3|A0A521BVJ3_9BACT    ..........MITRHDKSPTYQGEDLDEVEITGSKYCTKPISTFRIQHLKFQ..NMSYLN
tr|A0A359E0Y8|A0A359E0Y8_9BACT    ...........................................................MSYLN
tr|A0A2D8CIM6|A0A2D8CIM6_9BACT    ...........................................................MSYLN
tr|A0A2D9FWF5|A0A2D9FWF5_9BACT    ...........................................................MSYLN
tr|A0A1B6YB70|A0A1B6YB70_9BACT    ...........................................................MSYLN
tr|A0A2D4ZTC0|A0A2D4ZTC0_9BACT    ...........................................................MSYLE
tr|A0A3D4UVW7|A0A3D4UVW7_9BACT    ...........................................................MSYLE
tr|A0A3F3I3M2|A0A3F3I3M2_9BACT    ...........................................................MSYLE
tr|A0A3D1G7L9|A0A3D1G7L9_9BACT    ...........................................................MSYLQ
tr|A0A3M8G1Y8|A0A3M8G1Y8_9BACT    ...........................................................MSYLT
tr|A0A5Q4F391|A0A5Q4F391_9BACT    .................................................MKN..RSNILT
tr|A0A6I7NPV0|A0A6I7NPV0_9BACT    ..................................................MK..TKKILN
tr|A0A651G1J2|A0A651G1J2_9BACT    ..................................................MS..KKNILQ
tr|A0A371QRK5|A0A371QRK5_9BACT    .................................................MAK..HKSILN
tr|A0A2N0VGJ5|A0A2N0VGJ5_9BACT    ..................................................MS..SQKIFE
tr|A0A316TYJ0|A0A316TYJ0_9BACT    ...............................................MTTEA..KTNILH
tr|A0A651GLZ8|A0A651GLZ8_9BACT    ...............................................MTTTT..KPKILQ
tr|A0A7Y5V3S5|A0A7Y5V3S5_9BACT    ...........................................................MNALT
tr|A0A7W1SHC0|A0A7W1SHC0_9BACT    .............................................MNTAPQP..PGGAFT
tr|A0A7Y5PBT7|A0A7Y5PBT7_9BACT    ......................................................MTAALT
tr|A0A7Y5TXL8|A0A7Y5TXL8_9BACT    ......................................................MTAALT
tr|A0A423PQ96|A0A423PQ96_9GAMM    ........................MSNASAEKTSPSSAAAPRTSGAPTHTSA..DADLFA
tr|U2G066|U2G066_9GAMM            ....................................MSNNTTTARPAAPTQG..RDELFQ
tr|A0A2E0J1F4|A0A2E0J1F4_9GAMM    ........................................MSTATATTQAPA..GDDIFK
tr|A0A2D4SCM8|A0A2D4SCM8_9GAMM    .................................................MPK..KQTAFE
tr|L0WJH1|L0WJH1_9GAMM            ..................................................MT..QSRAFT
tr|A0A1H5XF61|A0A1H5XF61_9GAMM    .................................................MSK..TQRAFT
tr|A0A2E9TS48|A0A2E9TS48_9GAMM    .................................................MSN..TQRAFT
tr|A0A095UE19|A0A095UE19_9GAMM    .................................................MSK..TQRAFT
tr|A0A7G2S9A0|A0A7G2S9A0_9GAMM    .................................................MSK..TQRAFT
tr|B5JU27|B5JU27_9GAMM            MSEETKLNRANASKAASKKASKKVTKKKVSSKKSAAKKATKKVASKATQPAR..QQSAFQ
tr|A0A1Y0IHP1|A0A1Y0IHP1_9GAMM    .............................................MNAKVEL..PNSAFS
tr|A0A316FZ14|A0A316FZ14_9GAMM    .................................................MSH..SKSAFQ
tr|A0A498C231|A0A498C231_9GAMM    ......................................MNTHPETAQENPPE..RGTAFD
tr|Q0A6T4|Q0A6T4_ALKEH            ......................................MTTHPKTAQENRPE..QETAFH
tr|A0A3E0WL10|A0A3E0WL10_9GAMM    .......................................MAKQADTQTAQQD..VASMFS
tr|A0A3S1BU16|A0A3S1BU16_9GAMM    ...............................................MTNEL..NLTVFQ
tr|A0A7V8QFN9|A0A7V8QFN9_9GAMM    ........................................MATQQEKGTATD..SAEPMT
tr|A0A1H8PPF9|A0A1H8PPF9_9GAMM    ..........................................MATAKEQPGN..ETTIFH
tr|A0A6H0J0V7|A0A6H0J0V7_9GAMM    ...........................................................MTIFS
tr|V5EZX6|V5EZX6_9VIBR            ..................................................MT..ELKTFN
tr|A0A6I1QBM2|A0A6I1QBM2_9VIBR    ..................................................MT..ELKTFN
tr|A0A511QRL5|A0A511QRL5_9VIBR    ..................................................MS..ETKTFN
tr|A0A7Y0JZP4|A0A7Y0JZP4_9VIBR    ..................................................MS..ETKTFN
tr|A0A193KD24|A0A193KD24_9VIBR    ..................................................MS..ETKTFN
tr|A0A1S1HM72|A0A1S1HM72_PROST    .......................................MTDYSPREMQGEI..AKGAFR
tr|A0A7T8I613|A0A7T8I613_9GAMM    .......................................MTDYSPREMQGEI..AKGAFR
tr|A0A140NIF0|A0A140NIF0_PROSM    .......................................MTHHSETGVQAGL..VHEAFR
tr|A0A379GMB7|A0A379GMB7_PROST    .......................................MTHHSETGVQAGL..VHEAFR
tr|A0A379H393|A0A379H393_PROST    .......................................MTHHSETGVQAGL..VHEAFR
tr|B2Q0L5|B2Q0L5_PROST            .........................................MTHHSETGVQAGLVHEAFR
tr|A0A6I3JUE9|A0A6I3JUE9_9GAMM    .......................................MTHHSETEVQAEC..IQDAFR
tr|A0A7D4P594|A0A7D4P594_YERMW    ...............................MSKENGLNPADEPVVVDSVAV..TPSVFS
tr|A0A7U7IVK6|A0A7U7IVK6_YEREN    ...............................MSQENTLNSGDETTQVEAVAP..AHSVFT
tr|A0A447RIF5|A0A447RIF5_YEREN    ...............................MSQENTLNSGDETTQVEAVAP..AHSVFT
tr|A0A7H4ZLV9|A0A7H4ZLV9_YERP4    ...............................MSQENTLNSGDETTQVEAVAP..AHSVFT
tr|A0A0H3NX46|A0A0H3NX46_YERE1    ...............................MSQENTLNSGDETTQVEAVAP..AHSVFT
tr|A0A7T9XUV7|A0A7T9XUV7_YEREN    ...............................MSQENTLNSGDETTQVEAVAP..AHSVFT
tr|A0A7U7IYV8|A0A7U7IYV8_YEREN    ...............................MSQENTLNSGDETTQVEAVAP..AHSVFT
tr|A0A0H5G7G2|A0A0H5G7G2_YEREN    ...............................MSQENTLNSGDETTQVEAVAP..AHSVFT
tr|A0A2A7TDI2|A0A2A7TDI2_YERKR    ...............................................MLGSTAPAHSVFT
tr|A0A0T9M261|A0A0T9M261_YERKR    .................................MSQENTLNSGTETVMLGSTAPAHSVFT
tr|A0A0A0CMP1|A0A0A0CMP1_PHOLU    ...................................MTQAQHDAASITTGTDQ..TASVFN
tr|A0A329VG55|A0A329VG55_9GAMM    ...................................MTQAQHDAAPITTGTNQ..TASVFS
sp|Q7N288|FADJ_PHOLL              ...................................MTQAQHDAAPITTGTNQ..TASVFS
tr|A0A6L9JMX5|A0A6L9JMX5_PHOLM    ...................................MTQAQHDAAPITTGTNQ..TASVFS
tr|A0A7X5HQ29|A0A7X5HQ29_PHOLM    ...................................MTQAQHDAAPITTGTNQ..TASVFS
tr|A0A1C0U051|A0A1C0U051_9GAMM    ...................................MTQSQNDAVAVTADIKQ..AASVFN
tr|A0A7X5QF62|A0A7X5QF62_9GAMM    ...................................MTQSQHDAATITMDTKQ..AVSVFN
```

```
tr|A0A2D0KWM9|A0A2D0KWM9_9GAMM   .........................................MALDETIMSA..EQSVFS
tr|A0A2D0LAI1|A0A2D0LAI1_9GAMM   .........................................MALDETIMSA..EQSVFS
tr|A0A2D0IXC8|A0A2D0IXC8_XENBU   .......................................MAQAEKEIIMSE..TQSVFS
tr|W1JAM5|W1JAM5_9GAMM           .......................................MAQAEKETIMSE..TQSVFS
tr|A0A3D9UED3|A0A3D9UED3_9GAMM   .......................................MAQAEKETIMSE..TQSVFS
tr|A0A1I3JCA9|A0A1I3JCA9_9GAMM   ....................................MAQAEKETIMSTAQP..TQSVFS
tr|A0A068QUL9|A0A068QUL9_9GAMM   ...................................MVQAEKATVISAAMQP..EQSVFS
tr|A0A0M0TCH6|A0A0M0TCH6_9GAMM   .....................................MAQAEKETVMSAAMQPEQSVFR
tr|A0A1Q5U854|A0A1Q5U854_9GAMM   .....................................MVQAEKETVMSAAMQPDQSVFS
tr|A0A2D0ISP1|A0A2D0ISP1_9GAMM   .....................................MVQAEKETVMSAVMQPEQSVFS
tr|A0A1Q5TUI8|A0A1Q5TUI8_9GAMM   .....................................MAQTEKETVMSAAMQPEQSVFR
tr|A0A2D0KJI7|A0A2D0KJI7_9GAMM   .....................................MAQTEKETVISAAVQPEQSVFR
tr|A0A1Y2SB97|A0A1Y2SB97_9GAMM   ....................................MTQAEKETAVPAMQA..EQSVFS
tr|A0A1I5DWI7|A0A1I5DWI7_9GAMM   ......................................MTQAEKETIVSAMQPEQSVFS
tr|A0A1I7GWY6|A0A1I7GWY6_9GAMM   ....................................MALAEKETVISTTQS..NQSVFN
tr|D3VKY8|D3VKY8_XENNA           ......................................MAQAEKEVVILTKQSDRSVFN
tr|A0A2G0Q6Q9|A0A2G0Q6Q9_9GAMM   ....................................MAQAEKETVMSTTQP..EQSVFS
tr|A0A2D0JU24|A0A2D0JU24_9GAMM   ....................................MAQSEQEAVMSTTQP..KQSVFN
tr|A0A0J5FN38|A0A0J5FN38_9GAMM   ......................................MAQAEQETVMSTTQPKQSVFN
tr|A0A432XLD4|A0A432XLD4_9GAMM   .................................................MS..EQKAFT
tr|A0A2D8HU84|A0A2D8HU84_9GAMM   .................................................MS..QDKAFT
tr|A0A656X1Q2|A0A656X1Q2_9GAMM   .................................................MS..QDKAFT
tr|A0A4Q1QH79|A0A4Q1QH79_9GAMM   .................................................MS..QDKAFT
tr|A0A1G7LQE6|A0A1G7LQE6_9GAMM   .................................................MS..QDKAFT
tr|A0A432YVG7|A0A432YVG7_9GAMM   .................................................MS..QDKAFT
tr|A0A1J5N0P5|A0A1J5N0P5_9GAMM   ......................................................MSTFT
tr|A0A0D8D548|A0A0D8D548_9GAMM   ...........................................MTEETTSS..SASAFT
tr|A0A3E0TNM2|A0A3E0TNM2_9GAMM   ..............................MTQVTTQTSKQAAEQAAEQAKNADNSVFT
tr|A0A3E0UD67|A0A3E0UD67_9GAMM   ..........................................MTETTTQAG..ENSVFS
tr|A0A3E0U0P4|A0A3E0U0P4_9GAMM   ..........................................MTETTTQAG..ENSVFS
tr|A0A0M2V8B1|A0A0M2V8B1_9GAMM   ...............................................MTKQ..QASSFS
tr|A0A285ITY5|A0A285ITY5_9GAMM   .................MTNK................................QQGSFS
tr|A0A486XTB9|A0A486XTB9_9GAMM   .................................................MT..SSNSFS
tr|I1E1G1|I1E1G1_9GAMM           .................................................MT..TTNSFS
tr|A0A1H6KJ68|A0A1H6KJ68_9GAMM   .................................................MT..TTNSFS
tr|A0A0X3Y764|A0A0X3Y764_9GAMM   .................................................MT..TTTSFS
tr|A0A2N1YEV4|A0A2N1YEV4_9GAMM   .................................................MT..TTNSFS
tr|A0A3P3QCM3|A0A3P3QCM3_9GAMM   ....................................................MTQPTFT
tr|F7NT72|F7NT72_9GAMM           ............................................MSQPTLD..TKSTFS
tr|A0A3S2TW63|A0A3S2TW63_9GAMM   ....................................................MSQPTFS
tr|A0A3D5BGW7|A0A3D5BGW7_9GAMM   ...................................MTEQTVIEQTGTEQIA..SAKTFS
tr|A0A5C7TI17|A0A5C7TI17_9GAMM   .....................................................MQPTFT
tr|A0A0U4W982|A0A0U4W982_9GAMM   .....................................................MQPTFS
tr|A0A2I0FCM2|A0A2I0FCM2_9GAMM   ................................................MSQ..QDKTFS
tr|A0A2G2IVL1|A0A2G2IVL1_9GAMM   ................................................MSQ..QXKTFS
tr|A6FI21|A6FI21_9GAMM           ................................................MSQ..QEKTFS
tr|A0A4U1BNR2|A0A4U1BNR2_9GAMM   ....................................................MKTDSFL
tr|E1SQ60|E1SQ60_FERBD           ....................................................MSDKTFS
tr|A0A4Y6IZ09|A0A4Y6IZ09_9GAMM   .....................................................MEKSFT
tr|A0A0C3QSZ7|A0A0C3QSZ7_9GAMM   .....................................................MEKSFT
tr|A0A6L7HXW0|A0A6L7HXW0_9GAMM   .....................................................MEKSFT
sp|A3QFP3|FADJ_SHELP             .....................................................MDKSFT
tr|A0A1E5IXH7|A0A1E5IXH7_SHECO   .....................................................MDKSFT
tr|A0A411PKQ0|A0A411PKQ0_9GAMM   .....................................................MEKTFE
tr|A0A6G9QKM3|A0A6G9QKM3_9GAMM   .....................................................MEKTFN
tr|A0A6P1UL63|A0A6P1UL63_9GAMM   .....................................................MEKTFN
tr|A0A2N1ERI9|A0A2N1ERI9_9GAMM   .....................................................MEKTFN
tr|A0A7W4FU55|A0A7W4FU55_9GAMM   .....................................................MEKTFN
sp|Q8ECP7|FADJ_SHEON             .....................................................MGKTFN
tr|A0A501XZY8|A0A501XZY8_9GAMM   .....................................................MEKTFN
tr|A0A2W5DCZ0|A0A2W5DCZ0_SHEOE   .....................................................MEKTFN
tr|A0A1E3V3C8|A0A1E3V3C8_9GAMM   .....................................................MEKTFN
tr|A0A1Z4AI20|A0A1Z4AI20_9GAMM   .....................................................MEKTFN
tr|A0A7X9LJL9|A0A7X9LJL9_9GAMM   .....................................................MEKTFN
tr|A0A073KMY5|A0A073KMY5_9GAMM   .................................................MA..MEKTFN
sp|A0KV76|FADJ_SHESA             .....................................................MEKTFN
tr|A0A220UTH7|A0A220UTH7_9GAMM   .....................................................MEKTFN
tr|A0A5B8R6W1|A0A5B8R6W1_9GAMM   .....................................................MEKTFN
tr|V1DAI4|V1DAI4_9GAMM           .................................................MA..MEKTFN
tr|A0A448CPQ4|A0A448CPQ4_SHEPU   .....................................................MEKTFY
tr|A0A252ERQ3|A0A252ERQ3_SHEPU   .....................................................MEKTFN
sp|Q0HKD1|FADJ_SHESM             .....................................................MEKTFN
sp|Q0HWN3|FADJ_SHESR             .....................................................MEKTFN
tr|F7RQE3|F7RQE3_9GAMM           .....................................................MEKTFN
tr|B8EE98|B8EE98_SHEB2           .....................................................MEKTFN
sp|A6WQ25|FADJ_SHEB8             .....................................................MEKTFN
tr|A0A448EK41|A0A448EK41_9GAMM   .....................................................MEKTFN
tr|A0A553JHX1|A0A553JHX1_SHEHA   .....................................................MDKTFN
tr|B8CPY6|B8CPY6_SHEPW           .....................................................MEKTFN
tr|A0A431WFC4|A0A431WFC4_9GAMM   .....................................................MEKTFN
tr|A8FTR7|A8FTR7_SHESH           .....................................................MEKTFN
tr|A0A431WNL0|A0A431WNL0_9GAMM   .....................................................MEKTFN
tr|A0A550AEC5|A0A550AEC5_9GAMM   .....................................................MEKTFN
tr|A0A7L4WW90|A0A7L4WW90_9GAMM   .....................................................MEKTFN
tr|A9DDU3|A9DDU3_9GAMM           .....................................................MEKTFN
tr|A0A330M2B9|A0A330M2B9_9GAMM   .....................................................MEKTFN
tr|A0A5N8UFC7|A0A5N8UFC7_9GAMM   .....................................................MEKTFN
tr|A0A1S6HN57|A0A1S6HN57_9GAMM   .....................................................MEKTFN
tr|D4ZMH7|D4ZMH7_SHEVD           .....................................................MEKTFN
tr|A0A3L8Q213|A0A3L8Q213_9GAMM   .....................................................MERTFN
tr|A0A3A6U4N9|A0A3A6U4N9_9GAMM   .....................................................MEKTFN
tr|A0A4Q5MA37|A0A4Q5MA37_9GAMM   .....................................................MEKTFN
tr|A0A1L6LSX5|A0A1L6LSX5_9DELT   ............................................MNNGKAESAARQALS
tr|A0A2W4L9B9|A0A2W4L9B9_9PROT   .............................................MEVAVKEPEFRLLR
tr|A0A2W4M4E6|A0A2W4M4E6_9PROT   .................MNDR................................STTAFR
tr|A0A6I2GRX9|A0A6I2GRX9_9DELT   ...................................................MAAPADFQ
tr|A0A0H4WMK2|A0A0H4WMK2_9DELT   ..........................................MATKVEELE..AKQGFS
tr|F8CJ36|F8CJ36_MYXFH           ..........................................MATKAEELE..VKQGFS
tr|A0A250K0F1|A0A250K0F1_9DELT   ..........................................MATKAEELE..VKQGFS
tr|A0A7Y6WFZ2|A0A7Y6WFZ2_9DELT   ..........................................MATKAEELE..VKQGFS
tr|A0A7Y7C660|A0A7Y7C660_9DELT   ..........................................MATKAEELE..VKQGFS
tr|A0A7Y4JFH2|A0A7Y4JFH2_MYXXA   ..........................................MATKAEELE..VKQGFS
tr|A0A4Y6CZQ9|A0A4Y6CZQ9_MYXXA   ..........................................MATKAEELE..VKQGFS
```

```
tr|A0A7Y4IKV5|A0A7Y4IKV5_MYXXA  ...........................................MATKAEELE..VKQGFS
tr|A0A511HHB0|A0A511HHB0_9DELT  ...........................................MATKAEELE..VKQGFS
tr|A0A4Y6CKY7|A0A4Y6CKY7_MYXXA  ...........................................MATKAEELE..VKQGFS
tr|Q1D1F2|Q1D1F2_MYXXD          ...........................................MATKAEELE..VKQGFS
tr|A0A7Y4MA14|A0A7Y4MA14_MYXXA  ...........................................MATKAEELE..VKQGFS
tr|A0A7T8Y4N9|A0A7T8Y4N9_MYXXA  ...........................................MATKAEELE..VKQGFA
tr|L7UE67|L7UE67_MYXSD          ...........................................MAIKLEELE..AKQGFS
tr|A0A511T9X1|A0A511T9X1_MYXFU  ...........................................MAIQLEELQ..AKQGFS
tr|A0A7Y7C9C4|A0A7Y7C9C4_9DELT  ...........................................MAIKLEELE..AKQGFS
tr|A0A540X7W8|A0A540X7W8_9DELT  ...........................................MAIKIEELE..AKQGFS
tr|A0A3A5FK19|A0A3A5FK19_9DELT  ...........................................MAIKLDEVE..AKQGFR
tr|A0A3A8JQL9|A0A3A8JQL9_9DELT  ...........................................MATKQEAVE..AKQGLS
tr|A0A7Y4NFA4|A0A7Y4NFA4_9DELT  ...........................................MATKQEAVE..AKQGLS
tr|A0A3A8HDQ7|A0A3A8HDQ7_9DELT  ...........................................MATKQEAVE..AKQGLS
tr|A0A3A8GR90|A0A3A8GR90_9DELT  ...........................................MATKQEAVE..AKQGLS
tr|A0A3A8SBD7|A0A3A8SBD7_9DELT  ...........................................MATKQEAVE..AKQGLS
tr|A0A3A8T0I8|A0A3A8T0I8_9DELT  ...........................................MATKQEAVE..AKQGLS
tr|A0A7X5BU07|A0A7X5BU07_9DELT  ...........................................MATKQEAVE..AKQGLS
tr|A0A7Y1RVL2|A0A7Y1RVL2_9DELT  ...........................................MATKQETVE..AKQGLS
tr|A0A3A8THN2|A0A3A8THN2_9DELT  ...........................................MATKQEAVE..AKQGLS
tr|A0A7Y1RX76|A0A7Y1RX76_9DELT  ...........................................MATKQEAVE..AKQGLS
tr|A0A3A8RA58|A0A3A8RA58_9DELT  ...........................................MATKQEAVE..AKQGLS
tr|A0A410RPB6|A0A410RPB6_CORCK  ...........................................MATKQEAVE..AKQGLS
tr|A0A7Y4J474|A0A7Y4J474_CORCK  ...........................................MATKQEAVE..AKQGLS
tr|A0A3A8I9Z6|A0A3A8I9Z6_9DELT  ...........................................MATKQEAVE..AKQGLS
tr|H8MKE9|H8MKE9_CORCM          ...........................................MATKQEAVE..AKQGLS
tr|A0A3A8H102|A0A3A8H102_9DELT  ...........................................MATKQEAVE..AKQGLS
tr|A0A554FW33|A0A554FW33_9DELT  ...........................................MATKQEAVE..AKQGLS
tr|A0A3A8NPC0|A0A3A8NPC0_9DELT  ...........................................MATKHEEVE..AKQGLS
tr|A0A3A8JUV3|A0A3A8JUV3_9DELT  ...........................................MATKHEEVE..AKQGLS
tr|A0A3A8NEX6|A0A3A8NEX6_9DELT  ...........................................MATKHEEVE..AKQGLS
tr|A0A3A8JEU8|A0A3A8JEU8_9DELT  ...........................................MATKHEEVE..AKQGLS
tr|A0A3A8LI76|A0A3A8LI76_9DELT  ...........................................MATKHEEVE..AKQGLS
tr|A0A085WXN8|A0A085WXN8_9DELT  ...........................................MAIKLEELE..AQQGFT
tr|A0A2T4V0M5|A0A2T4V0M5_9DELT  ..........................................MAAMIAQELE..AKQGFT
tr|A0A0G2ZSW5|A0A0G2ZSW5_9DELT  ..........................................MAAMIAQELE..AKQGFT
tr|A0A3M2DKY0|A0A3M2DKY0_9DELT  .................................................MTS..TPRALT
tr|A0A661NQ58|A0A661NQ58_9DELT  ..........................................MTTKNENSGT..KTESFR
tr|A0A520YD99|A0A520YD99_9DELT  .....................................................MANEGLS
tr|A0A7Y3BRE4|A0A7Y3BRE4_9DELT  .....................................................MANEGLS
tr|A0A2D9TF90|A0A2D9TF90_9DELT  ....................................................MSEEKTFR
tr|A0A2E0TP32|A0A2E0TP32_9DELT  ................................................MSDE..KKSALS
tr|A0A2E4Y3V1|A0A2E4Y3V1_9PROT  ................................................MSLP..NLKYFN
tr|A0A2E6VRH4|A0A2E6VRH4_9DELT  .......................................................MKNIH
tr|A0A1F9FB59|A0A1F9FB59_9DELT  ............................................................
```

ECHA_HUMAN

```
ECHA_HUMAN                                      T T
                                                         50           60           70           80           90          100
ECHA_HUMAN                              YGV.KGDVAVVRINSPNSKVNTLSKELHSEFSEVMNEIWA.SDQIRSAVLISSKPGCFIA
tr|A0A4R6XLA8|A0A4R6XLA8_9GAMM          LIK.ENNTATIWMDQPGSEVNTLSVKMLDDFSSLLDQIEQ.DDEIKAAVLISKKDNCFIA
tr|A0A6A0IJT2|A0A6A0IJT2_9BACT          YELRLDGVAVLTFDTPGSSVNVLSKELLGEVAELLEVVET.DPDVKAVVLSSGKADTFIA
tr|A0A7V3QZU7|A0A7V3QZU7_9BACT          VTKRPDGVAVLTFDNPKGKVNILSPEVVEEFRAVIDDIER.DPGVKACVLVSAKPDNFIA
tr|A0A7V4SYJ6|A0A7V4SYJ6_9BACT          LEKRLDGVAILTFDNPSGPQNILSRSLFGHFRSAIEDVLA.DPHVKAAVLASGKKD.FLA
tr|A0A2A8D2N6|A0A2A8D2N6_9BACT          LTIDDDGIATVMIDDPDASVNKISEDMLAAFEEVIGIAEK.NSEIRGAVFISGKDDTFIA
tr|A0A2N1TGP0|A0A2N1TGP0_9SPIR          VKV.KDHVAVITVNCPGTKVNAVSSGLLDEITALMPEFEN.DQEIEGLVIVSGKKDSFIV
tr|A0A1G3QGW4|A0A1G3QGW4_9SPIR          VDVTKDRVAVVTIDCKDSTVNKVSSELLDEIAGMLDKINL..KELIGLVIVSGKEDNFVV
tr|A0A1G3QTY3|A0A1G3QTY3_9SPIR          MDITKEKVAVVTIDCRDSKVNKVSGELLDEISGMLDHISS..QEIKGLVIASGKEDNFVV
tr|A0A7X9L359|A0A7X9L359_9DELT          FEKKDNGVAVVTIDCPDSRVNKVSSGLLAEIAGLLDEVGR.DGTIRGMVILSGKDDNFVV
tr|A0A522CJY0|A0A522CJY0_9SPIR          FEVTPDQVAVITINCQGTKVNKVSSELLTEIDSMLTEIES.NKALRGVVVMSGKSDNFVV
tr|A0A2N1RRJ3|A0A2N1RRJ3_9SPIR          FEKTEDGIAVVTIDCPGQKVNTISSGLMGDVEAVLDRIEK.DSSVRGVAVLSAKDNNFVA
tr|A0A2N2KFN7|A0A2N2KFN7_9DELT          LEITKEGIAVVTVDCPDGKVNKISSGLLREVAANLQSISN.DPKVAGMVIVSGKQDNFVV
tr|A0A1V6AXX9|A0A1V6AXX9_9DELT          MEKTKDGIAIITIDCPESKVNKVSSGLLDEVAGVLDDIKN.DKSIKGMVIASGKEDNFVV
tr|A0A2N2HZL0|A0A2N2HZL0_9DELT          LEKANDGIAIVTIDCPESKVNKISSGLLDEIAGILPDISN.DQSIKGMVITSGKEDNFVV
tr|A0A5E8ARP3|A0A5E8ARP3_9BACT          LEK.HGDVGVIWMDLPGEEWNKISIDTEAQFREVMQQIEA.DSGLKASVLISRKKG.FMA
tr|A0A662A7B1|A0A662A7B1_9BACT          ............................................................
tr|D7CV16|D7CV16_TRURR                  LDR.DDDVAVIVFDQPDSRVNTLSQEAMREFATVLDELER.DEALRGAVLCSGKRDSFVV
tr|A0A3C2AKX4|A0A3C2AKX4_9FLAO          IEK.RDGIAIIWMDEQDSKINKIGPDMIDTFNPMMDELEN.DSEVRAAVMISRKKD.FIA
tr|A0A3B8ZLN3|A0A3B8ZLN3_9PLAN          LEN.RNGIGIIWMDQQGSKVNKIGPDMIGMFNPLLDQLER.DANIKAVVMISRKKD.FIA
tr|A0A345UGV8|A0A345UGV8_9BACT          LTR.ENAIAWLTLDQPGDKVNTLGKEMISGFGKLLDEVQQ.DAGIKAVVLISGKENNFIA
tr|A0A6C1P6A2|A0A6C1P6A2_9BACT          KEI.EGGILFMILDQPGERMNVLGREMIPEMHTLLDGIEA.DPAIQAVVLISGKPDNFIA
tr|A0A3M1X9W8|A0A3M1X9W8_9BACT          LEL.REGIAILWLDYPGEKINKISPELVDEFDALLDELEG.RSDVEGVVLASRKPDNFIA
tr|A0A354C796|A0A354C796_9DELT          IEK.ENGVAVIWLDQPGEKVNTISLDLVGTFTAILDSLEK.DETVKGIVLISRKPDNFIA
tr|A0A3M1NUM8|A0A3M1NUM8_9BACT          IEK.ENGVAVVWLDQPGEKVNKLAADLVDEFHALLDQLQQ.DSEVQAIVLISKKKDTFIA
tr|A0A3M2KRC8|A0A3M2KRC8_9BACT          VEK.EDGVAVVWLDQPGEKVNKLGPYMLEEFRTLLDDLES.HDEVKAVVLISGKPDTFIA
tr|A0A5S9IHR0|A0A5S9IHR0_9BACT          LQK.DDGVALLWLDRQGEKVNTLSVKTLDSFSKLLDELEK.DPEVKAVVLISKKKDNFIV
tr|A0A6M1ST81|A0A6M1ST81_9BACT          IRT.EDNIAVVTLDQPDSKVNKLDEELIDAFEELMDKYEN.DSELRGIVIISGKEGNFIA
tr|A0A521AAE5|A0A521AAE5_9BACT          TEE.QDHILVVTLDQPDEKVNKLDEQLISEFQELLPRAAQ..QDIEGIVLTSGKENNFIA
tr|A0A1M4ZMD6|A0A1M4ZMD6_9BACT          TEK.QDDILVVTLDQPGEKVNKLDEQLMTEFRDLLEKAGQ..ETITGLVLISGKENNFIA
tr|A0A2A2GCS2|A0A2A2GCS2_9BACT          TEI.QDNILVVTLDQPDSKVNKLTEELISEVKELLENTNP..DDIDGIVMVSGKKNNFIA
tr|A0A5D3YIH7|A0A5D3YIH7_9BACT          IEE.QENILVVTLNQPDEKVNKLNEALISDFENLLNDVN..DNEIDGIVLQSGKEGNFIA
tr|A0A6M1T9G0|A0A6M1T9G0_9BACT          TEN.EDGVLVVTLDQPGEKVNKLDEQLIGEFQDLLQNTDP..DSIEGIVLVSGKESNFIA
tr|A0A6A8Q1Y8|A0A6A8Q1Y8_9BACT          ITH.KNSVAIITLDLPGEKVNKLNESLMDEFSDFLDKLES.NDDLIGAVLISGKEDNFIA
tr|A0A521BVJ3|A0A521BVJ3_9BACT          ITP.KDSVAVVTLDMPDEKVNKLNEILIEEFSEFLDTLES.DDSLKGAILISGKENNFIA
tr|A0A359E0Y8|A0A359E0Y8_9BACT          ITH.KDSVAIITLDLPGEKVNKLNESMMDEFSTFLDVLEA.NDELIGAVLISGKKDTFIA
tr|A0A2D8CIM6|A0A2D8CIM6_9BACT          ITH.KDSVAIITLDLPGEKVNKLNESMMDEFSTFLDVLEA.NDELIGAVLISGKKDTFIA
tr|A0A2D9FWF5|A0A2D9FWF5_9BACT          ISQ.KESVAILTLDLPGEKVNKLNEDLIEEFSAFLDKLES.DNDLKGAVLISGKKDNFIA
tr|A0A1B6YB70|A0A1B6YB70_9BACT          ITK.KENVAIITLDQPGEKVNTLNEAMMDQFASFLDDLDS.DPSLKGAVLISGKENNFIA
tr|A0A2D4ZTC0|A0A2D4ZTC0_9BACT          ITK.TENVAVLTLDQPGEKVNTLNESMMEQFASFLTDLDS.DPSLIGAVLISGKENNFIA
tr|A0A3D4UVW7|A0A3D4UVW7_9BACT          ITK.TENVAVLTLDQPGEKVNTLNESMMEQFASFLTDLDS.DPSLIGAVLISGKENNFIA
tr|A0A3F3I3M2|A0A3F3I3M2_9BACT          ITK.TENVAVLTLDQPGEKVNTLNESMMEQFASFLTDLDS.DPSLIGAVLISGKENNFIA
tr|A0A3D1G7L9|A0A3D1G7L9_9BACT          KEI.KNSVAVLTLDKPGEKVNTLDESMMVQFSEFLDELNT.NDELRGAVLISGKKDNFIA
tr|A0A3M8G1Y8|A0A3M8G1Y8_9BACT          ITH.KNSVAVLSLDKPGEKVNTLDESMMEQFDVFLDELHQ.NDELNGAVLISGKENNFIA
tr|A0A5Q4F391|A0A5Q4F391_9BACT          IDQ.EQGIAIVTLNNPGEKVNKLSEELIEEFSVTCDSLEQ.DPRVEGVLLMSGKEENFIA
tr|A0A6I7NPV0|A0A6I7NPV0_9BACT          TEI.IDNVAIITLDTPGDKVNKLNDGLINEFSAILDKLES.DNSIEGVVLVSGKEENFIA
tr|A0A651G1J2|A0A651G1J2_9BACT          TEI.INSVAVVTLDTPDEKVNKLDERLIKEFKEVLDQVKS.DHSILGAVLISGKEENFIA
tr|A0A371QRK5|A0A371QRK5_9BACT          TEI.ISGVAIVTLDTPDEKVNKLNEALIEEFSGFLDQLES.DEELKGAVLLSGKDENFIA
tr|A0A2N0VGJ5|A0A2N0VGJ5_9BACT          IEN.KEDVAIITMDTPGEKVNKLNEALIDEISDILDQLES.DDSLKGAVLVSGKDGNFIA
tr|A0A316TYJ0|A0A316TYJ0_9BACT          TEI.IDGVAVVTLDTPDEKVNKLNEELIEEFSELLDRLES.DDSLNGALLVSGKDENFIA
tr|A0A651GLZ8|A0A651GLZ8_9BACT          TET.VDSVLIVRLDTPGEKVNKLNEPMIEEFSALLDRLET.DDTLEGALLISGKESNFIA
tr|A0A7Y5V3S5|A0A7Y5V3S5_9BACT          LTV.DDGVGIVTFDAPGQALNIISRSVRNEFIALFQQLDA.DPSIRAAVLISGKPDAFIA
tr|A0A7W1SHC0|A0A7W1SHC0_9BACT          VTR.DVDIAVVTLDIPGQPLNTLSTSLAASFGTALDEITR..SGARAIVLVSGKPDSFIA
tr|A0A7Y5PBT7|A0A7Y5PBT7_9BACT          LEH.VDGIAVVTIDLPGEPVNKVTAALRAEFAATFDRLSA.DSTVRGVVLASGKPDTWLA
tr|A0A7Y5TXL8|A0A7Y5TXL8_9BACT          LEH.VDGIAVVTIDLPGEPVNKVTAALRAEFAATFDRLSA.DSAVRGVVLASGKPDTWLA
tr|A0A423PQ96|A0A423PQ96_9GAMM          VERRDDGIAVVRFAVPGATQNTLRADFAPAFETLLESLEH.DTEVIGVVLVSARPGSFMA
tr|U2G066|U2G066_9GAMM                  LEKREDGIVVLRFGVPGARQNTIQADFADAFDHVLETIEH.DTEVIGVVLISNKPGSFLA
tr|A0A2E0J1F4|A0A2E0J1F4_9GAMM          LEKHADGIVVLRFGVPGASQNTIKADFADAFDDVLEKLEH.DTEVIGVVLISDKPGSFMA
tr|A0A2D4SCM8|A0A2D4SCM8_9GAMM          LEIRDDNVAVVRMNVPGEAHNVLKAEFTDEFEDLFRQLKG.NGQLQAVVFGSSKPGSFLA
tr|L0WJH1|L0WJH1_9GAMM                  VKPLDNGVAVLEINVPGDAQNTLKAEFADDFNAAFSDIES..ARPKALVLYSTKPGSFMA
tr|A0A1H5XF61|A0A1H5XF61_9GAMM          LKPLDNGVAVLEINVPGDTQNTLKAEFADDFNAAFADIEK..AAPKALVLYSTKAGSFMA
tr|A0A2E9TS48|A0A2E9TS48_9GAMM          LKPLDNGVAVLEINVPGDAQNTLKAEFADDFNAAFSAIEK..TKPKALVLYSTKAGSFMA
tr|A0A095UE19|A0A095UE19_9GAMM          LKPLDNGVAVLEINVPNDAQNTLKAEFADDFNSAFAAIEK..AKPKALVLYSTKAGSFMA
tr|A0A7G2S9A0|A0A7G2S9A0_9GAMM          LKPLDNGVAVLEINVPNDAQNTLKAEFADDFNSAFAAIEK..AKPKALVLYSTKAGSFMA
tr|B5JU27|B5JU27_9GAMM                  LRVRDDKVGVIEINVPGEAQNTLKAEFAEQMDDVMDQVRR.QAGLKGLVLISTKPGSFVA
tr|A0A1Y0IHP1|A0A1Y0IHP1_9GAMM          LKLREDAIGVIEINVPGDAQNTLKAEFADEFEVLFSELQA.QTGLQGLVLISTKPGSFVA
tr|A0A316FZ14|A0A316FZ14_9GAMM          FELKDNGVGVITIDVPGESQNTLKKEFVAEINDLLNSLEQ..LDVKGLVLISGKASSFVA
tr|A0A498C231|A0A498C231_9GAMM          LVLGDDGIAVITVDVPGERMNVLKGEMVREANALLDTLDE.QPALRGVIFTSGKADNFIA
tr|Q0A6T4|Q0A6T4_ALKEH                  LALGDDGIAVITVDVPGERMNVLKGEMVREANALLDTLDE.QPALRGVIFTSGKADNFIA
tr|A0A3E0WL10|A0A3E0WL10_9GAMM          LDIRNDGVAIVTFDVPGESQNTLKIEAVDEANRLFDELEQ.NSTVKAVVFKSGKPGSFIA
tr|A0A3S1BU16|A0A3S1BU16_9GAMM          LALREDGIAVITIDLPGESQNTLKAAFVTEANALLDRLEQ.DPAIRGVVFISGKPGSFIA
tr|A0A7V8QFN9|A0A7V8QFN9_9GAMM          LEIRDDGIAVITIDCPGEAQNTLGEAVMESARKHLADVTR.RKDIKGLIFRSAKPGSFIA
tr|A0A1H8PPF9|A0A1H8PPF9_9GAMM          VNRREDGIAVVTIDLPGESQNVLSRALMEEATASMDALES.DQSVKGIVFISGKPGSFIA
tr|A0A6H0J0V7|A0A6H0J0V7_9GAMM          INRRDDGIAVVTIDLPGESQNVLNRALMEEAVQCADTLDK.DDGLKGVIFISGKPGSFIA
tr|V5EZX6|V5EZX6_9VIBR                  LSIDEEQLAWLSIDVQGEKMNTLQAAFADEMSEILSQLEQKKSEIKGLIIHSLKPDNFIA
tr|A0A6I1QBM2|A0A6I1QBM2_9VIBR          LSIDEEQLAWLSIDVQGEKMNTLQAAFADEMNEILSQLEQKKSEIKGLIIHSLKPDNFIA
tr|A0A511QRL5|A0A511QRL5_9VIBR          LSIDSEQLAWLSIDVQGEKMNTLQAAFADEMNEILGQLEAKKSEITGLIVYSLKPDNFVA
tr|A0A7Y0JZP4|A0A7Y0JZP4_9VIBR          LTIDSEQLAWLSIDVQGEKMNTLQAAFADEMNDILEQLEAKKSEIKGLIVHSLKPDNFVA
tr|A0A193KD24|A0A193KD24_9VIBR          LTIDSEQLAWLSIDVQGEKMNTLQAAFADEMNDILEQLEAKKSEIKGLIVHSLKPDNFVA
tr|A0A1S1HM72|A0A1S1HM72_PROST          LEVHSQTVGVIYIDVPNEKVNTLKAEFAQQFLSILQQAQS.TSGLKGLVITSGKKDSFIA
tr|A0A7T8I613|A0A7T8I613_9GAMM          LEVHSQTVGVIYIDVPNEKVNTLKAEFAQQFLSILQQAQS.TSGLKGLVITSGKKDSFIA
tr|A0A140NIF0|A0A140NIF0_PROSM          LEVHAQTVGVIFIDVPNEKVNTLKAEFAQQFLSILQQAQS.TPGLKGLVITSGKKDSFIA
tr|A0A379GMB7|A0A379GMB7_PROST          LEVHAQTVGVIFIDVPNEKVNTLKAEFAQQFLSILQQAQS.TPGLKGLVITSGKKDSFIA
tr|A0A379H393|A0A379H393_PROST          LEVHAQTVGVIFIDVPNEKVNTLKAEFAQQFLSILQQAQS.TPGLKGLVITSGKKDSFIA
tr|B2Q0L5|B2Q0L5_PROST                  LEVHAQTVGVIFIDVPNEKVNTLKAEFAQQFLSILQQAQS.TPGLKGLVITSGKKDSFIA
tr|A0A6I3JUE9|A0A6I3JUE9_9GAMM          LEVHAQTVGVIFIDVPNEKVNTLKAEFAQQFLAILQQAQS.TSGLKGLVITSGKKDSFIA
tr|A0A7D4P594|A0A7D4P594_YERMW          LDVRPDNIGVITIDVIGDKVNTLKAEFAEQIAEILQQAQA.LPQLQGLVIISGKPDSFIA
tr|A0A7U7IVK6|A0A7U7IVK6_YEREN          LKVRHDNIGIITVDVVGDKVNTLKAEFAEQIAEILQQAQA.LSQLQGLVIISGKPDSFIA
tr|A0A447RIF5|A0A447RIF5_YEREN          LKVRHDNIGIITVDVVGDKVNTLKAEFAEQIAEILQQAQA.LSQLQGLVIISGKPDSFIA
tr|A0A7H4ZLV9|A0A7H4ZLV9_YERP4          LKVRHDNIGIITVDVVGDKVNTLKAEFAEQIAEILQQAQA.LSQLQGLVIISGKPDSFIA
tr|A0A0H3NX46|A0A0H3NX46_YERE1          LKVRHDNIGIITVDVVGDKVNTLKAEFAEQIAEILQQAQA.LSQLQGLVIISGKPDSFIA
tr|A0A7T9XUV7|A0A7T9XUV7_YEREN          LKVRHDNIGIITVDVVGDKVNTLKAEFAEQIAEILQQAQA.LSQLQGLVIISGKPDSFIA
tr|A0A7U7IYV8|A0A7U7IYV8_YEREN          LKVRHDNIGIITVDVVGDKVNTLKAEFAEQIAEILQQAQA.LSQLQGLVIISGKPDSFIA
tr|A0A0H5G7G2|A0A0H5G7G2_YEREN          LKVRHDNIGIITVDVVGDKVNTLKAEFAEQIAEILQQAQA.LSQLQGLVIISGKPDSFIA
tr|A0A2A7TDI2|A0A2A7TDI2_YERKR          LDVRPDNIGIITVDVVGDKVNTLKAEFAEQIAEILQQAQA.LSQLQGLVIISGKPGSFIA
tr|A0A0T9M261|A0A0T9M261_YERKR          LDVRPDNIGIITVDVVGDKVNTLKAEFAEQIAEILQQAQA.LSQLQGLVIISGKPGSFIA
tr|A0A0A0CMP1|A0A0A0CMP1_PHOLU          FDVRPDKIGVITINVPGEKVNTLKAEFVDQFLNVFKQAQQ.SSGLKGLILISGKPDTFIA
tr|A0A329VG55|A0A329VG55_9GAMM          FDVRPDKIGIITINVPGEKVNTLKAEFVDQFLNVFKQAQQ.SSGLKGLILISGKPDTFIA
sp|Q7N288|FADJ_PHOLL                    FDVRPDKIGIITINVPGEKVNTLKAEFVDQFLNVFKQAQQ.SSGLKGLILISGKPDTFIA
tr|A0A6L9JMX5|A0A6L9JMX5_PHOLM          FDVRPDKIGIITINVPGEKVNTLKAEFVDQFLNVFKQAQQ.SSGLKGLILISGKPDTFIA
tr|A0A7X5HQ29|A0A7X5HQ29_PHOLM          FDVRPDKIGIITINVPGEKVNTLKAEFVDQFLNVFKQAQQ.SSGLKGLILISGKPDTFIA
tr|A0A1C0U051|A0A1C0U051_9GAMM          FDIRPDKIGVITIDVPGEKVNTLKAEFVDQFLNVFKQAQQ.VSGLKGLILISGKPDTFIA
tr|A0A7X5QF62|A0A7X5QF62_9GAMM          FDVRPDKIGVITIDVPGEKVNTLKAEFVDQFLNVFKQAQQ.SSGLKGLILISGKPDTFIA
```

```
tr|A0A2D0KWM9|A0A2D0KWM9_9GAMM  FSVRDDRVGVITIDVPGEKVNTLKAEFVEQFLAVFEQAQQ.VSGLKGLVIISGKPDTFIA
tr|A0A2D0LAI1|A0A2D0LAI1_9GAMM  FSVRDDRVGVITIDVPGEKVNTLKAEFVEQFLAVFEQAQQ.VSGLKGLVIISGKPDTFIA
tr|A0A2D0IXC8|A0A2D0IXC8_XENBU  FSVRDDKIGVITIDVPGEKVNTLKAEFVEQFLTVFEKAQQ.VSGLKGLVIVSGKPDTFIA
tr|W1JAM5|W1JAM5_9GAMM          FSVRDDKIGVITIDVPGEKVNTLKAEFVEQFLTVFEKAQQ.VSGLKGLVIVSGKPDTFIA
tr|A0A3D9UED3|A0A3D9UED3_9GAMM  FSVRDDKIGVITIDVPGEKVNTLKAEFVEQFLTVFEKAQQ.VSGLKGLVIVSGKPDTFIA
tr|A0A1I3JCA9|A0A1I3JCA9_9GAMM  FSVRDDKVGIVTIDVPDEKVNTLKAEFVEQFLVIFDKAKQ.VSGLKGLVIVSGKPDTFIA
tr|A0A068QUL9|A0A068QUL9_9GAMM  FSVRDDKVGIITIDVPGEKVNTLKAEFVDQFLVIFEKAQQ.VSDLKGLVIISGKPDTFIA
tr|A0A0M0TCH6|A0A0M0TCH6_9GAMM  FSVRDDKVGIITIDVPGEKVNTLKAEFVEQFLIMFEKAQQ.VSNLKGLVIVSGKADTFIA
tr|A0A1Q5U854|A0A1Q5U854_9GAMM  FSVRDDKVGIITIDVPGEKVNTLKAEFVEQFLIMFEKAQQ.VSGLKGLVIVSGKPDTFIA
tr|A0A2D0ISP1|A0A2D0ISP1_9GAMM  FSVRDDKVGIITIDVPGEKVNTLKAEFVEQFLIMFEKAQQ.VSGLKGLVIVSGKPDTFIA
tr|A0A1Q5TUI8|A0A1Q5TUI8_9GAMM  FSVRDDKVGIITIDVPGEKVNTLKAEFVEQFLKVFEKAQQ.VSDLKGLVIVSGKPDTFIA
tr|A0A2D0KJI7|A0A2D0KJI7_9GAMM  FSVRDDKVGIITIDVAGEKVNTLKAEFVEQFLTVFEKAQQ.VSGLKGLVIVSGKPDTFIA
tr|A0A1Y2SB97|A0A1Y2SB97_9GAMM  FSVRDDKVGIITIDVPGEKVNTLKAEFVEQFLTMFEKAQQ.VSGLKGLIIISGKPDTFIA
tr|A0A1I5DWI7|A0A1I5DWI7_9GAMM  FSVRDDKVGIITIDVPGEKVNTLKAEFVEQFLMMFEKAQQ.VSGLKGLVIISGKPDTFIA
tr|A0A1I7GWY6|A0A1I7GWY6_9GAMM  FSVRDDNVGVITIDVPGEKVNTLKAEFVEQFQTTFEKAQQ.VSGLKGLVIISGKPDTFIA
tr|D3VKY8|D3VKY8_XENNA          FAVREDKVGVITIDVPGEKVNTLKAEFVEQFLTMFEKAQQ.VSGLKGLVIISGKPDTFIA
tr|A0A2G0Q6Q9|A0A2G0Q6Q9_9GAMM  FSIRDDKVGVITIDVPDEKVNTLKAEFIDQFLTMFEKAQQ.ASGLKGLVIVSGKPDTFIA
tr|A0A2D0JU24|A0A2D0JU24_9GAMM  FSVRDDKVGIITIDVPGEKVNTLKAEFVEQFLTMFEKVQQ.VSGLKGLVIVSGKPDTFIA
tr|A0A0J5FN38|A0A0J5FN38_9GAMM  FSVREDKVGIITIDVPGEKVNTLKAEFVEQFLTMFEKAQQ.VSGLKGLVIVSGKPDSFIA
tr|A0A432XLD4|A0A432XLD4_9GAMM  LELRDDKVAVIHIDVPGESMNTLKASFAEEVAQVMNKIEQ.HNDLKGVVVISDKPGSFVA
tr|A0A2D8HU84|A0A2D8HU84_9GAMM  MEVRDDGVAVVTIDVPGESMNTLKDSFAEEVGSLMNRLES.DDSVKGVVFISGKPGSFIA
tr|A0A656X1Q2|A0A656X1Q2_9GAMM  MEVRDDGVAVVTIDVPGESMNTLKDSFAEEVGSLMNRLES.DDSVKGVVFISGKPGSFIA
tr|A0A4Q1QH79|A0A4Q1QH79_9GAMM  MDIRDDGVAVVTIDVPGESMNTLKDSFADEVGSLLNKLES.DSSIKGVVFISGKPGSFIA
tr|A0A1G7LQE6|A0A1G7LQE6_9GAMM  MDIRDDGVAVVTIDVPGESMNTLKDSFADEVGSLLNKLES.DNSIKGVVFISGKPGSFIA
tr|A0A432YVG7|A0A432YVG7_9GAMM  MDIRDDGVAVVTIDVPGESMNTLKDSFADEVGSLLNKLES.DSSIKGVVFISGKPGSFIA
tr|A0A1J5N0P5|A0A1J5N0P5_9GAMM  TTINDDNIAIISMDVAGEKMNTLRAEFGDEMTSVLDELDA.NSALKGVVVISGKPDNFIA
tr|A0A0D8D548|A0A0D8D548_9GAMM  LVRQENGLAHLVMDVVGDTMNTLKAEFAEQVGEVLQEIKG.DKDIKGIVLLSGKKDSFVA
tr|A0A3E0TNM2|A0A3E0TNM2_9GAMM  LTRQENGIAHLIMDVPGESMNTLKAEFTEQVTEMLKAIKD.DSSITGIVVVSGKKDSFVA
tr|A0A3E0UD67|A0A3E0UD67_9GAMM  LVRQENGIAHLIMDVPGETMNTLKAEFSEQVTEMLKEIKD.DSSITGVVIVSGKADSFVA
tr|A0A3E0U0P4|A0A3E0U0P4_9GAMM  LVRQENGIAHLIMDVPGETMNTLKAEFSEQVTEMLKEIKD.DSSITGVVIVSGKADSFVA
tr|A0A0M2V8B1|A0A0M2V8B1_9GAMM  LAIRDDKVAVITMDIAGESMNVLKASFADEIDAILNAIQS.DSSVKGVVIISGKKDSFIA
tr|A0A285ITY5|A0A285ITY5_9GAMM  LAIRDDQVAVITMDIPGESMNVLKASFADEIDAILNTLQG.NSDVKGVVIVSGKKDSFIA
tr|A0A486XTB9|A0A486XTB9_9GAMM  LQIRDDLVGVITMDIAGESMNVLKAAFADEISALLSEIKA.NSQLKGLVFISGKPDSFIA
tr|I1E1G1|I1E1G1_9GAMM          LQIRDDNVGVITMDIAGESMNVLKAAFADEISQLLSDIKA.NSQLKGLVFISGKPDSFIA
tr|A0A1H6KJ68|A0A1H6KJ68_9GAMM  LQIRDDQIGVITMDIAGESMNVLKAEFADEISSLLADIKA.NSQLKGLVFISGKPDSFIA
tr|A0A0X3Y764|A0A0X3Y764_9GAMM  LQIRDDQIGVITMDIAGESMNVLKAAFADEISSLLADIKA.NSQLKGLVFISGKPDSFIA
tr|A0A2N1YEV4|A0A2N1YEV4_9GAMM  LQIRDDQIGVITMDIAGESMNVLKAAFADEISALLAQIKA.NNQLKGLVFISGKPDSFIA
tr|A0A3P3QCM3|A0A3P3QCM3_9GAMM  LTVRPDQVGVISMDVAGESMNVLKAAFADEIKSLLSEIKN.NKDIKGLVIISGKADSFIA
tr|F7NT72|F7NT72_9GAMM          LSIRPDQIGVISMDVAGESMNVLKAAFADEIKSLLSEIKN.NKDIKGLVIISGKPDSFIA
tr|A0A3S2TW63|A0A3S2TW63_9GAMM  LSIRPDQIGVISMDVAGESMNVLKAAFADEIKSLLSEIKN.NKDIKGLVIISGKPDSFIA
tr|A0A3D5BGW7|A0A3D5BGW7_9GAMM  LNLRDDGVGVITMDVAGESMNVLKAAFADEIKALLDEIRS.NKNIKGLVIISGKADSFIA
tr|A0A5C7TI17|A0A5C7TI17_9GAMM  LSQRDDGVAVITMDIAGESMNVLKAEFADEISAVLEQVRS.NKSIKGVVIISGKPDSFIA
tr|A0A0U4W982|A0A0U4W982_9GAMM  LSQRDDGVGVITMDIFGESMNVLKAEFADEIAAVLDEVRS.NKNIKGLVIISGKADSFIA
tr|A0A2I0FCM2|A0A2I0FCM2_9GAMM  LEIREDKIGVLTMDVPGETMNTLRAEFADEISDLMKEIKS.NSDLQGLVLISGKKDSFVA
tr|A0A2G2IVL1|A0A2G2IVL1_9GAMM  LEIREDKIGVLTMDVPGETMNTLRAEFADEISDLMKEISA.NSDLQGLVLISGKKDSFVA
tr|A6FI21|A6FI21_9GAMM          LEIREDKIGVLTMDVPGETMNTLRAEFADEISDLMKEISA.NSDLQGLVLISGKKDSFVA
tr|A0A4U1BNR2|A0A4U1BNR2_9GAMM  VEVHEDNIAIITMDVPGETMNTLRSEFGDEVRQVLDFLKS.RNDLKGVVFTSGKPDSFIA
tr|E1SQ60|E1SQ60_FERBD          LDVGNDGVAIITMDVPGESMNTLRDSFAAEVRDLLGEIVQ.DSSIKGVVFTSGKPGSFIA
tr|A0A4Y6IZ09|A0A4Y6IZ09_9GAMM  LSRLDSGIAVLTMDVPGETMNTLRAEFGPEICEMLAEIKS.DASIRGVVIISGKKDSFVA
tr|A0A0C3QSZ7|A0A0C3QSZ7_9GAMM  LNRQDNGIAVLTMDVPGETMNTLRAEFGPEICEMLAEIKS.DASIRGVVIISGKKDSFVA
tr|A0A6L7HXW0|A0A6L7HXW0_9GAMM  LNRLDNGIAVLTMDVPGETMNTLKAEFGPEICEMLAEIKS.DASIRGVVLISGKQDSFVA
sp|A3QFP3|FADJ_SHELP            LNRLDNGIAVLTMDVPGETMNTLRAEFGPEICEMLAEIKA.DAGIRGVVIISGKKDSFVA
tr|A0A1E5IXH7|A0A1E5IXH7_SHECO  LSRNENGIAVLTMDVPGETMNTLRAEFGPEISEVLTEIRS.DHQIKGLVIISGKKDSFVA
tr|A0A411PKQ0|A0A411PKQ0_9GAMM  LTRREDGIAILTMDVPGETMNTLKAEFGPEISEILSEVKQ.DTSIKGLVIISGKSDSFVA
tr|A0A6G9QKM3|A0A6G9QKM3_9GAMM  LARREDGIAVLTMDVPGETMNTLKAEFGPEITEILTEIKA.DSSIKGLVVVSGKADSFVA
tr|A0A6P1UL63|A0A6P1UL63_9GAMM  LARRDDGIAILTMDVPGETMNTLKEQFGPEISEILTEIKA.DNSIKGLVVISGKADSFVA
tr|A0A2N1ERI9|A0A2N1ERI9_9GAMM  LARRDDGIAILTMDVPGETMNTLKEQFGPEISEILTEIKA.DNSIKGLVVISGKADSFVA
tr|A0A7W4FU55|A0A7W4FU55_9GAMM  LARRDDGIAILTMDVPGETMNTLKEQFGPEISEILTEIKA.DNSIKGLVVISGKADSFVA
sp|Q8ECP7|FADJ_SHEON            LTRREDGIAILTMDVPGETMNTLKAEFGPEISEILSEIKR.DSSIRGLVLISGKKDSFVA
tr|A0A501XZY8|A0A501XZY8_9GAMM  LTRREDGIAILTMDVPGETMNTLKAEFGPEISEILSEIKR.DSSIRGLVLISGKKDSFVA
tr|A0A2W5DCZ0|A0A2W5DCZ0_SHEOE  LTRREDGIAILTMDVPGETMNTLKAEFGPEISEILSEIKR.DSNIRGLVLISGKKDSFVA
tr|A0A1E3V3C8|A0A1E3V3C8_9GAMM  LTRREDGIAILTMDVPGETMNTLKAEFGPEISEILSEIKR.DSSIRGLVLISGKKDSFVA
tr|A0A1Z4AI20|A0A1Z4AI20_9GAMM  LTRREDGIAILTMDVPGETMNTLKAEFGPEISEILSEIKR.DSSIRGLVLISGKKDSFVA
tr|A0A7X9LJL9|A0A7X9LJL9_9GAMM  LTRREDGIAILTMDVPGETMNTLKAEFGPEISEILSEIKR.DSSIRGLVLISGKKDSFVA
tr|A0A073KMY5|A0A073KMY5_9GAMM  LTRREDGIAILTMDVPGETMNTLKAEFGPEISEILSEIKR.DSSIRGLVLISGKKDSFVA
sp|A0KV76|FADJ_SHESA            LTRREDGIAILMMDVPGETMNTLKAEFGPEISEILSEIKR.DSSIRGLVLISGKKDSFVA
tr|A0A220UTH7|A0A220UTH7_9GAMM  LTRREDGIAILMMDVPGETMNTLKAEFGPEISEILSEIKR.DSSIRGLVLISGKKDSFVA
tr|A0A5B8R6W1|A0A5B8R6W1_9GAMM  LTRREDGIAILTMDVPGETMNTLKAEFGPEISEILSEIKG.DNSIRGLVLISGKKDSFVA
tr|V1DAI4|V1DAI4_9GAMM          LTRREDGIAILTMDVPGETMNTLKAEFGPEISEILSEIKG.DNSIRGLVLISGKKDSFVA
tr|A0A448CPQ4|A0A448CPQ4_SHEPU  LTRREDGIAILTMDVPGETMNTLKAEFGPEISEILSEIKR.DSSIRGLVLISGKKDSFVA
tr|A0A252ERQ3|A0A252ERQ3_SHEPU  LTRREDGIAILTMDVPGETMNTLKAEFGPEISEILSEIKR.DSSIRGLVLISGKKDSFVA
sp|Q0HKD1|FADJ_SHESM            LTRREDGIAILTMDVPGETMNTLKAEFGPEISEILSEIKR.DSSIRGLVLISGKKDSFVA
sp|Q0HWN3|FADJ_SHESR            LTRREDGIAILTMDVPGETMNTLKAEFGPEISEILSEIKR.DSSIRGLVLISGKKDSFVA
tr|F7RQE3|F7RQE3_9GAMM          LTRRDDGIAILTMDVPGETMNTLKAQFGPEISEILAEIKR.DSSIRGLVLISGKKDSFVA
tr|B8EE98|B8EE98_SHEB2          LTRRDDGIAILTMDVPGESMNTLKAQFGPEISEILAEIKS.DSSIRGLVLISGKKDSFVA
sp|A6WQ25|FADJ_SHEB8            LTRRDDGIAILTMDVPGETMNTLKAQFGPEISEILAEIKS.DSSIRGLVLISGKKDSFVA
tr|A0A448EK41|A0A448EK41_9GAMM  LTRRDDGIAILTMDVPGESMNTLKAQFGPEISEILAEIKS.DSSIRGLVLISGKKDSFVA
tr|A0A553JHX1|A0A553JHX1_SHEHA  LTKREDGIAVLTMDVPGETMNTLRAEFGPEISQVLAEIKA.DSSIKGLVLISGKKDSFVA
tr|B8CPY6|B8CPY6_SHEPW          LARREDGIAILTMDVPGETMNTLRSEFGPEISEMLAEIKQ.DSSIKGLVLVSGKKDSFVA
tr|A0A431WFC4|A0A431WFC4_9GAMM  LARREDGIAILTMDVPGETMNTLRAEFGPEINQVLAEIKA.DNSIKGLVLISGKKDSFVA
tr|A8FTR7|A8FTR7_SHESH          LARREDGIAILTMDVPGETMNTLRAEFGPEINQVLAEIKA.DSSIKGLVLISGKKDSFVA
tr|A0A431WNL0|A0A431WNL0_9GAMM  LARREDGIAILTMDVPGETMNTLRAEFGPEINQVLAEIKA.DNSIKGLILISGKRDSFVA
tr|A0A550AEC5|A0A550AEC5_9GAMM  LSRREDGIAVLTIDVPGESMNTLRAEFGPEITDVLAEIKA.DSSIKGLVIASGKKDCFVA
tr|A0A7L4WW90|A0A7L4WW90_9GAMM  LSRREDGIAVLTIDVPGESMNTLRAEFGPEITDVLAEIKA.DSSIKGLVIASGKKDCFVA
tr|A9DDU3|A9DDU3_9GAMM          LVRREDGIAVLTIDVPGETMNTLRSEFGPEITEILAEIKA.DNSIKGLVLASAKKDCFVA
tr|A0A330M2B9|A0A330M2B9_9GAMM  LVRREDGIAVLTIDVPGETMNTLRTEFGPEITEILAEIKA.DNSIKGLVLASAKKDCFVA
tr|A0A5N8UFC7|A0A5N8UFC7_9GAMM  LVRREDGIAVLTIDVPGETMNTLRTEFGPEITEILAEIKA.DKSIKGLVLASGKKDCFVA
tr|A0A1S6HN57|A0A1S6HN57_9GAMM  LVRREDGIAVLTIDVPGETMNTLRTEFGPEITEVLAEIKA.DTSIKGLVLASGKKDCFVA
tr|D4ZMH7|D4ZMH7_SHEVD          LVRREDGIAVLTIDVPGETMNTLRTEFAAEITEVLAEIKA.DTSIKGLVLASGKKDCFVA
tr|A0A3L8Q213|A0A3L8Q213_9GAMM  LERRDDGIAVVTMDVPGDTMNTLRAEFANEISELLSEIKR.DNSIKGLVLISGKKDSFIA
tr|A0A3A6U4N9|A0A3A6U4N9_9GAMM  LTRRDDGIAVLTMDVPGESVNTLRAEFSAEINELLSEIKS.DSSIKGLVIISGKKDSFIA
tr|A0A4Q5MA37|A0A4Q5MA37_9GAMM  LSRRDDGIAVLTMDVPGDSVNTLQAKFAEEITDLLKEIKA.DRNIKGMVVISGKKNSFIA
tr|A0A1L6LSX5|A0A1L6LSX5_9DELT  VKVRPDGVAVITYDVAGEPVNTLKATFGAEFERVFGEIAR.DPAIKAAILVSGKPDGFIA
tr|A0A2W4L9B9|A0A2W4L9B9_9PROT  VDVASDGVAHVVLDDPSEPVNTISEELGAEMRRLWARLKA.DPAVRSILISSGKKGNFVA
tr|A0A2W4M4E6|A0A2W4M4E6_9PROT  LDRRADGFALLVVDVPGEPTNVLRAEFASELERVLEEVER.DDTIRAVILISGKADGFIA
tr|A0A6I2GRX9|A0A6I2GRX9_9DELT  YAV.EDGVAVITLDQRESPVNTLSPETGEAFAQLLTRAME.DAAVRAVVFTSGKKDGFVA
tr|A0A0H4WMK2|A0A0H4WMK2_9DELT  YQV.EGGVAVITFDLPDSPVNTLSPETGEAFLRVMSRAER.EPEVKAVVFTSGKKDSFVA
tr|F8CJ36|F8CJ36_MYXFH          YQV.EDGVAVITFDLPDSPVNTLSPETGEAFLRVMSRAER.EPEVKAVVFTSGKKDSFVA
tr|A0A250K0F1|A0A250K0F1_9DELT  YQV.EDGVAVITFDLPDSPVNTLSPETGEAFLRVMSRAER.EPEVKAVVFTSGKKDSFVA
tr|A0A7Y6WFZ2|A0A7Y6WFZ2_9DELT  YQV.EDGVAVITFDLPDSPVNTLSPETGEAFLRVMTRAER.EPEVKAVVFTSGKKDSFVA
tr|A0A7Y7C660|A0A7Y7C660_9DELT  YQV.EDGVAVITFDLPDSPVNTLSPETGEAFLRVMTRAER.EPEVKAVVFTSGKKDSFVA
tr|A0A7Y4JFH2|A0A7Y4JFH2_MYXXA  YQV.EDGVAVITFDLPDSPVNTLSPETGEAFLRVMMRAER.EPEVKAVVFTSGKKDSFVA
tr|A0A4Y6CZQ9|A0A4Y6CZQ9_MYXXA  YQV.EDGVAVITFDLPDSPVNTLSPETGEAFLRVMMRAER.EPEVKAVVFTSGKRDSFVA
```

```
tr|A0A7Y4IKV5|A0A7Y4IKV5_MYXXA  YRV.EDGVAVITFDLPDSPVNTLSPETGEAFLRVMARAER.EPEVKAVVFTSGKKDSFVA
tr|A0A511HHB0|A0A511HHB0_9DELT  YRV.EDGVAVITFDLPDSPVNTLSPETGEAFLRVMARAER.EPEVKAVVFTSGKKDSFVA
tr|A0A4Y6CKY7|A0A4Y6CKY7_MYXXA  YRV.EDGVAVITFDLPDSPVNTLSPETGEAFLRVMARAER.EPEVKAVVFTSGKKDSFVA
tr|Q1D1F2|Q1D1F2_MYXXD          YQV.EGGVAVITFDLPDSPVNTLSPETGEAFLRVMMRAER.EPEVKAVVFTSGKKDSFVA
tr|A0A7Y4MA14|A0A7Y4MA14_MYXXA  YQV.EGGVAVITFDLPDSPVNTLSPETGEAFLRVMMRAER.EPEVKAVVFTSGKKDSFVA
tr|A0A7T8Y4N9|A0A7T8Y4N9_MYXXA  YQV.EGGVAVITFDLPDSPVNTLSPETGEAFLRVMMRAER.EPEVKAVVFTSGKKDSFVA
tr|L7UE67|L7UE67_MYXSD          LQV.EDGVAVITFDLPDSAVNTLSPDVGTAFTRILEEVER.EPSVKAVVFISGKKDSFVA
tr|A0A511T9X1|A0A511T9X1_MYXFU  LQV.EDGVAVVTFDLPDSAVNTLSPEVGAAFSRLLEEAER.EAEVKALVFISGKKDSFVA
tr|A0A7Y7C9C4|A0A7Y7C9C4_9DELT  LSV.EDGIAVVSFDLPDSAVNTLSPEVGAAFTLLLEEVER.ESHVKAVVLISGKKDSFVA
tr|A0A540X7W8|A0A540X7W8_9DELT  LTV.EDGVAVVSFDLPDSAVNTLSPEVGAAFTLLLEEVER.EAQVKAVVFISGKKDNFVA
tr|A0A3A5FK19|A0A3A5FK19_9DELT  YEV.EGGVAVITFDLPDSAVNTLSPETGEAFTALMRHAEG.DTAVKAVVFISGKKDNFVA
tr|A0A3A8JQL9|A0A3A8JQL9_9DELT  YDV.TNGVAVITVDQPGAPVNTLSPETGTAFTTLLQQAER.DPEVKAVVFISGKKDNFVA
tr|A0A7Y4NFA4|A0A7Y4NFA4_9DELT  YDV.TNGVAVITVDQPGAPVNTLSPEVGTAFTTLLQQAER.DPEVKAVVFISGKKDNFVA
tr|A0A3A8HDQ7|A0A3A8HDQ7_9DELT  YDV.TNGVAVITVDQPGAPVNTLSPEVGTAFTTLLQQAER.DPEVKAVVFISGKKDNFVA
tr|A0A3A8GR90|A0A3A8GR90_9DELT  YDV.TNGVAVITVDQPGAPVNTLSPEVGAAFSDLLLQAER.DPEVRAVVFISGKKDNFVA
tr|A0A3A8SBD7|A0A3A8SBD7_9DELT  YDV.TNGVAVITVDQPGSPVNTLSPDVGAAFSDLLLQAER.DPEVKAVVFISGKKDNFVA
tr|A0A3A8T0I8|A0A3A8T0I8_9DELT  YDV.TNGVAVITVDQPGAPVNTLSPDVGAAFSDLLLQAER.DPEVKAVVFISGKKDNFVA
tr|A0A7X5BU07|A0A7X5BU07_9DELT  YDV.TNGVAVITVDQPGAPVNTLSPDVGAAFSDLLLQAER.DPEVKAVVFISGKKDNFVA
tr|A0A7Y1RVL2|A0A7Y1RVL2_9DELT  YDV.TNGVAVITVDQPGAPVNTLSPDVGAAFSDLLLQAER.DPEVKAVVFISGKKDNFVA
tr|A0A3A8THN2|A0A3A8THN2_9DELT  YDV.TNGVAVITVDQPGSPVNTLSPDVGAAFSDLLLQAER.DPEVKAVVFISGKKDNFVA
tr|A0A7Y1RX76|A0A7Y1RX76_9DELT  YDV.TNGVAVITVDQPGAPVNTLSPDVGAAFSDLLLQAER.DPEVKAVVFISGKKDNFVA
tr|A0A3A8RA58|A0A3A8RA58_9DELT  YDV.TNGVAVITVDQPGAPVNTLSPEVGTAFSDLLLQAER.DPEVKAVVFISGKKDNFVA
tr|A0A410RPB6|A0A410RPB6_CORCK  YDV.TNGVAVITVDQPGAPVNTLSPDVGAAFSDLLLQAER.DPEVRAVVFISGKKDNFVA
tr|A0A7Y4J474|A0A7Y4J474_CORCK  YDV.TNSVAVITVDQPGAPVNTLSPDVGAAFSDLLLQAER.DPEVRAVVFISGKKDNFVA
tr|A0A3A8I9Z6|A0A3A8I9Z6_9DELT  YDV.TNGVAVITVDQPGAPVNTLSPDVGAAFSDLLLQAER.DPEVRAVVFISGKKDNFVA
tr|H8MKE9|H8MKE9_CORCM          YDV.TNGVAVITVDQPGAPVNTLSPEVGTAFSDLLVQAER.DPEVKAVVFISGKKDNFVA
tr|A0A3A8H102|A0A3A8H102_9DELT  YDV.TNGVAVITVDQPGAPVNTLSPDVGTAFSELLVQAER.DPEVKAVVFISGKKDNFVA
tr|A0A554FW33|A0A554FW33_9DELT  YGV.TDGIAVITVDLPGAPVNTLSPEVGTASTELLLQAER.DPEVKAVVFISGKKDSFVV
tr|A0A3A8NPC0|A0A3A8NPC0_9DELT  YDV.TDGVAVITVDQPGAPVNTLSPEVGTAFTQLLEQAER.DPAVKAVVFISGKKDNFVA
tr|A0A3A8JUV3|A0A3A8JUV3_9DELT  YDV.TDGVAVITVDLPGAPVNTLSPEVGTAFTHLLEQAER.ALDVKAVVFISGKKDNFVA
tr|A0A3A8NEX6|A0A3A8NEX6_9DELT  YDV.TDGVAVITVDQPGAPVNTLSPEVGTAFTQLLEQAER.ALDVKAVVFISGKKDNFVA
tr|A0A3A8JEU8|A0A3A8JEU8_9DELT  YGV.TDGVAVITVDLPGAAVNTLSPEVGTEFTQLLLQAER.DPEVKAVVFISGKKDNFVA
tr|A0A3A8LI76|A0A3A8LI76_9DELT  YDV.TDGVAVITVDLPGAAVNTLSPEVGTAFTQLLEQAER.ALDVKAVVFISGKKDNFVA
tr|A0A085WXN8|A0A085WXN8_9DELT  YSV.EDGVAVITIDQQGEAVNTLSPETGTAFEKLLGHAEA.DPNVKAVVFTSGKKDTFVA
tr|A0A2T4V0M5|A0A2T4V0M5_9DELT  YHV.EDGVALVVIDQPGEPVNTLSPETGEVFASLLNRAEK.DPAVKAVVFTSGKKDNFVA
tr|A0A0G2ZSW5|A0A0G2ZSW5_9DELT  YQV.EDGVALLVLDLPGEAVNTLSPETGEVFASLLTRAEK.DPAVKAVVFISGKKDSFVA
tr|A0A3M2DKY0|A0A3M2DKY0_9DELT  LDKRADGIAVVTFDVPGDSVNTLQATFADDFAEVFAKIEA.DDAIRAVVLRSGKKDNFVA
tr|A0A661NQ58|A0A661NQ58_9DELT  VERRDDGVAVVLMDVPGVTMNTLNARFADEFDALFGELSA.ADDVRAIVFASGKADSFIA
tr|A0A520YD99|A0A520YD99_9DELT  IEKRSDGVAIVRMDIPGEAMNTLKSDFIETFTEAFSSIEN.DPDIKAVVFTSGKKDSFIA
tr|A0A7Y3BRE4|A0A7Y3BRE4_9DELT  IEKRSDGVAIVRMDIPGEAMNTLKSDFIETFTEAFSSIEN.DPDIKAVVFTSGKKDSFIA
tr|A0A2D9TF90|A0A2D9TF90_9DELT  VEHREGGVAVLIMDVPGASMNTLRGDFNAEFAGAFDALET.DPTVKAVVFASGKPGSFIA
tr|A0A2E0TP32|A0A2E0TP32_9DELT  IDERDDGVAILWMDVPGESMNTLKEDFAQEFAEAFAHLET.NANVKAVVFASGKKGSFIA
tr|A0A2E4Y3V1|A0A2E4Y3V1_9PROT  LSV.KDNVLIIAFDDPEEKVNTLNLKMTDEFHKIMDVIDN.DDNIKAIVFTSGKPDNFIV
tr|A0A2E6VRH4|A0A2E6VRH4_9DELT  SEV.IENILVVTLDLQGEKVNKLTSTLSGEFEEAFKQAE..DNAVEAVVIISGKEDCFVA
tr|A0A1F9FB59|A0A1F9FB59_9DELT  ..........MTIDVPGEKQNTLSEKTKHGFKVCFERVAA.DPAIEGAVLISGKPD.FIA
```

```
ECHA_HUMAN
                                                    110          120          130          140          150
ECHA_HUMAN                           GADIN.MLAACKTLQEVTQLSQEAQRIVEKLEKS..TKPIVAAINGSCLGGGLEVAISCQ
tr|A0A4R6XLA8|A0A4R6XLA8_9GAMM       GADIK.DLMSIKDSKEVENLSREGNKILSRLAQL..KKPVIAAINGACLGGGLEVAMACH
tr|A0A6A0IJT2|A0A6A0IJT2_9BACT       GANLD.QVLAMETAEEAAAFSRNGHALLDRIERS..AKPVVAAIHGAALGGGLEVAMACR
tr|A0A7V3QZU7|A0A7V3QZU7_9BACT       GADLK.FFLSIRDPVVGERFSREGHALLDRVAGS..SKPTVAAIAGLALGGGLEVALACH
tr|A0A7V4SYJ6|A0A7V4SYJ6_9BACT       GADLK.DFLACRTPEEGAALSAEGHVLMDLVAKS..PKPFVAAIHGQVLGGGVELVLCCT
tr|A0A2A8D2N6|A0A2A8D2N6_9BACT       GADID.MLSAFEMPADVRALSRRAHRLLARMQNL..RMPTVAAINGAAMGGGLEMTLGAT
tr|A0A2N1TGP0|A0A2N1TGP0_9SPIR       GADID.ELNRKTTREEVIDYISKAHVVLNKLESL..SIPVVCAIHGSCLGGGMELTLTAR
tr|A0A1G3QGW4|A0A1G3QGW4_9SPIR       GADID.EVLAMKSDGEIRNYISRANDIINRIDDL..PIPVVSCIHGNCLGGGLEVALASD
tr|A0A1G3QTY3|A0A1G3QTY3_9SPIR       GADVD.EVLSMKTDLEIRDYISRANQILARIDAL..PIPVVSCIHGNCLGGGLEIALASD
tr|A0A7X9L359|A0A7X9L359_9DELT       GADVD.ELNAMKGKDEVIAYITKAHDVLKRIEAL..PFPVVCGIHGNCLGGGLELALVAN
tr|A0A522CJY0|A0A522CJY0_9SPIR       GADID.EVKAASTKEAAEDYLSRGHAVLNRLAAL..RIPKVAAIHGNCLGGGLELALIAD
tr|A0A2N1RRJ3|A0A2N1RRJ3_9SPIR       GADLF.ELKKMKTSEEVLSYMSKGHHILNRIENM..KIPFVACINGTCLGGGLEIALACH
tr|A0A2N2KFN7|A0A2N2KFN7_9DELT       GAAID.ELQAMRTKEEVVAYIALGHSILNDLENM..KIPVVACIAGNCLGGGLELALACN
tr|A0A1V6AXX9|A0A1V6AXX9_9DELT       GADID.ELKGMRTTEEVIAYISKGHAILNSLEKM..KIPVVACIHGNCLGGGLELALVCN
tr|A0A2N2HZL0|A0A2N2HZL0_9DELT       GADID.ELKGMRTSEEVIAYISKAHAILNGLESM..QIPVVACIHGNCLGGGLELALACN
tr|A0A5E8ARP3|A0A5E8ARP3_9BACT       GADIE.KFLQMK.PGESKATAQAGHAMLRDLEDS..KKPCVAAIHGACMGGGLEISLACA
tr|A0A662A7B1|A0A662A7B1_9BACT       .............GDEPKQAALTGHKLLLRIENS..EKPFVVAIHGACMGGGTEISLACA
tr|D7CV16|D7CV16_TRURR               GADIK.AFRTFESPEQVTALIHEGHALFNRLDAL..NKPVVAAVHGAAVGGGLELILACH
tr|A0A3C2AKX4|A0A3C2AKX4_9FLAO       GADIE.AFANVKKEGDWQPIAKKGHEILDRIQNS..KKPIVAAINGACMGAGLEIALACS
tr|A0A3B8ZLN3|A0A3B8ZLN3_9PLAN       GADIE.AFANVQKPGDWLPIGKQGHAILNRIESS..RKPIVAAIHGACMGAGLEIALACS
tr|A0A345UGV8|A0A345UGV8_9BACT       GADIK.MFDTFTTPEEFGAFIKTGTDLLDRIEKF..PKPVIAAINGGCIGGGLEVVMACH
tr|A0A6C1P6A2|A0A6C1P6A2_9BACT       GADIK.LFQSLDTPEAMEKFNREGNTLLDRIEAF..PKPVIAAMHGTTMGGGMEVALACH
tr|A0A3M1X9W8|A0A3M1X9W8_9BACT       GADLD.VFLKMNEPGQAKQLSRRGHAVLNRLAAF..PRPVVAAIHGAALGGGLEVALACH
tr|A0A354C796|A0A354C796_9DELT       GADLD.KFKDMTSPEEAEALSRKGHTLLNRMANF..PKPIVAALHGATLGGGLEVALACH
tr|A0A3M1NUM8|A0A3M1NUM8_9BACT       GADLE.RLLALKEPGQVEALSRSGQAVLDRVAAF..PKPIVAAIHGAALGGGLEVALACH
tr|A0A3M2KRC8|A0A3M2KRC8_9BACT       GADLDKLLELRHTPGAVEDLSRQGHQLLNRLASS..RKPVVAAIHGAALGGGLEVALACH
tr|A0A5S9IHR0|A0A5S9IHR0_9BACT       GADLD.VLQTFDKAEDAQKASQDGNDLLTRVSKF..PKPFVAAINGTTMGGGLEVALACH
tr|A0A6M1ST81|A0A6M1ST81_9BACT       GADVE.MLKNKQAPEGIEELSRRGNELLLRLENY..NKPVVAAIHGACIGGGNEVAMACH
tr|A0A521AAE5|A0A521AAE5_9BACT       GADVE.MLKNKKAPKEVEDLSRRGNDLLLELEHF..DKPVVAAIHGSCMGGGLEVAMACH
tr|A0A1M4ZMD6|A0A1M4ZMD6_9BACT       GADVA.MLKNKKAPEEIEDLSRRGNDLLLDLEHF..EKPIVAAIHGSCMGGGLEVAMACH
tr|A0A2A2GCS2|A0A2A2GCS2_9BACT       GADVE.MLKNKKAPEEIEDLSQRGNRLLLQLENY..PKPVVAAIHGSCLGGGLEVAMACN
tr|A0A5D3YIH7|A0A5D3YIH7_9BACT       GADVE.MLKNKSAPEGIEELSRRGNELLLKLEKH..PIPVVAAIHGSCIGGGMEVAMACN
tr|A0A6M1T9G0|A0A6M1T9G0_9BACT       GADVE.MLKNKSAPEGIEELSRRGNKLLLKLENY..PKPVVAAIHGSCIGGGMEVAMACN
tr|A0A6A8Q1Y8|A0A6A8Q1Y8_9BACT       GADID.MFQERDTVEEIEQLSKDGHQILNRIADF..PKPIAVAVHGSCMGGGLELLLACH
tr|A0A521BVJ3|A0A521BVJ3_9BACT       GADID.MFQERDTAEEIEQLSKTGHQILNRIADF..EKPIAVAVHGSCMGGGLELSLACH
tr|A0A359E0Y8|A0A359E0Y8_9BACT       GADID.MFQARDTAEEIEQLSKDGHKILNRIADF..KKPIAVAVHGSCMGGGLELSLACH
tr|A0A2D8CIM6|A0A2D8CIM6_9BACT       GADID.MFQARDTAEEIEQLSKDGHKILNRIADF..KKPIAVAVHGSCMGGGLELSLACH
tr|A0A2D9FWF5|A0A2D9FWF5_9BACT       GADID.MFQERDTAEEIEQLSKKGHEILNRIADF..KKPIAVAVHGSCMGGGLELSLACH
tr|A0A1B6YB70|A0A1B6YB70_9BACT       GADIE.MFKARETAEEIEQLSLDGHKILNRVESS..SKPIVVAIHGSCMGGGLELSLACH
tr|A0A2D4ZTC0|A0A2D4ZTC0_9BACT       GADIE.MFKSRETAQEIEQLSLDGHKILNRVESS..SKPIVVAIHGSCMGGGLELSLACQ
tr|A0A3D4UVW7|A0A3D4UVW7_9BACT       GADIE.MFKSRETAQEIEQLSLDGHKILNRVESS..SKPIVVAIHGSCMGGGLELSLACQ
tr|A0A3F3I3M2|A0A3F3I3M2_9BACT       GADIE.MFKSRETAQEIEQLSLDGHKILNRVESS..SKPIVVAIHGSCMGGGLELSLACQ
tr|A0A3D1G7L9|A0A3D1G7L9_9BACT       GADID.MFKARDTAEELEELSLNGHEILNKIEAS..AKPVVVAIHGSCMGGGLELSLACD
tr|A0A3M8G1Y8|A0A3M8G1Y8_9BACT       GADIE.MFKVRDTAEAMQQLSTDGNAILNKITAS..KKPFVVAIHGSCMGGGLELSLACH
tr|A0A5Q4F391|A0A5Q4F391_9BACT       GADIG.MFKARETAHELAELSRVGHQILLRVEHF..AKPFVVAIHGSCMGGGTELALACH
tr|A0A6I7NPV0|A0A6I7NPV0_9BACT       GADIG.MFKSRETAEELEGLSRTGHNILLRIERF..TKPVVVAIHGSCMGGGTELALACH
tr|A0A651G1J2|A0A651G1J2_9BACT       GADIE.MFKTRDTAEELAALSWTGHEILLEIEHS..TKPIVVAINGSCMGGGTELALACH
tr|A0A371QRK5|A0A371QRK5_9BACT       GADIE.MFKTRETSEELAELSWTGHEILLRVEQC..KKPIVAGIHGSCMGGGTELALACH
tr|A0A2N0VGJ5|A0A2N0VGJ5_9BACT       GADIE.MFKTRETAEELSELSWTGHEILLRIENL..KKPFVVGINGSCMGGGTELALASH
tr|A0A316TYJ0|A0A316TYJ0_9BACT       GADIE.MFKTRDTAEEMAKLSWTGHEILLRVEKC..KKPIVAGIHGSCMGGGTELALACH
tr|A0A651GLZ8|A0A651GLZ8_9BACT       GADIE.MFKTRETADELSELSWTGHEILLRVEQF..KKPIVVGIHGSCMGGGTELALACH
tr|A0A7Y5V3S5|A0A7Y5V3S5_9BACT       GADIE.EFLDWKTAAQAESASREGHALLDRLERL..RTPVVAAIHGACLGGGLEASLACP
tr|A0A7W1SHC0|A0A7W1SHC0_9BACT       GADIE.QLLGARTAQEAERMSRDGQAFFDRLEQS..KLPVVAAIHGACLGGGLELALACA
tr|A0A7Y5PBT7|A0A7Y5PBT7_9BACT       GADID.EFLTLQTAGEAEALSRSGQALLARLESL..DIPVVAAIHGACLGGGLETALACR
tr|A0A7Y5TXL8|A0A7Y5TXL8_9BACT       GADID.EFLTLQTAGEAEALSRSGQALLARLESL..DIPVVAAIHGACLGGGLETALACR
tr|A0A423PQ96|A0A423PQ96_9GAMM       GADID.MLDAQKSAADVEALSKQGQAGFRRLSKL..HVPVVAAIEGACLGGGLELALACD
tr|U2G066|U2G066_9GAMM               GADIG.LFEQVKSAGEVEALSLRGQAGFSRLSKL..HVPVVAAIEGSCLGGGLEMALACD
tr|A0A2E0J1F4|A0A2E0J1F4_9GAMM       GADIG.LFNDVKSANEVEALSLAAQAGFKRLSRL..HVPVVAAIAGSCLGGGLEMALACD
tr|A0A2D4SCM8|A0A2D4SCM8_9GAMM       GADIK.LFENVSTAKEIEDLSRQLHAALQEMEDL..PVPVVAAIDGACLGGGLELALACD
tr|L0WJH1|L0WJH1_9GAMM               GADIN.LFEKAENAQQVSDLSTLCQQTFGKLETL..GIPVVAAIKGACLGGGLELALACS
tr|A0A1H5XF61|A0A1H5XF61_9GAMM       GADIK.LFENAKTAKEVSDLSILCQQTFQKLEDL..DIPVVAAIKGACLGGGMELALACS
tr|A0A2E9TS48|A0A2E9TS48_9GAMM       GADIK.LFENAKTAKEVSDLSTQCQQTFQKLEDL..DIPVVAAIKGACLGGGMELALACS
tr|A0A095UE19|A0A095UE19_9GAMM       GADIK.LFESAKTAKEVSDLSTQCQQTFQKLEDL..DIPVVAAIKGACLGGGMELALACS
tr|A0A7G2S9A0|A0A7G2S9A0_9GAMM       GADIK.LFESAKTAKEVSDLSTQCQQTFQKLEDL..DIPVVAAIKGACLGGGMELALACS
tr|B5JU27|B5JU27_9GAMM               GADIN.LFDSIGSKQEVVELSKTCHRGFKAIEDL..NIPVVAAIEGACLGGGLELAMACH
tr|A0A1Y0IHP1|A0A1Y0IHP1_9GAMM       GADIN.LFDHAQSADEVSDLSRLCQKTFQRLEDL..QIPVVAAIDGACLGGGLELAMACH
tr|A0A316FZ14|A0A316FZ14_9GAMM       GADIS.MLSDINEASDAEDLAKQGQAIFNRLEKL..NIPSVAAINGSCLGGGLELAMACH
tr|A0A498C231|A0A498C231_9GAMM       GADVH.MLAACETAEAARELARAGQRLFDRLEAF..AKPVVAAIHGTCLGGGLELALACH
tr|Q0A6T4|Q0A6T4_ALKEH               GADVH.MLAACETAEAARELAHAGQRLFDRLEAF..PKPVVAAIHGTCLGGGLELALACH
tr|A0A3E0WL10|A0A3E0WL10_9GAMM       GADIN.MLVRCQTADEATELARGGQNFFNRIERF..RAPVVAAIDGACLGGGFELAMACH
tr|A0A3S1BU16|A0A3S1BU16_9GAMM       GADIH.MLHACQSAAEATALSQAGQRFFDRLEQF..KAPVVAAIDGACLGGGLELALACR
tr|A0A7V8QFN9|A0A7V8QFN9_9GAMM       GADIR.MLQRCGSAQEVSELSRAGQRAFDQIEDA..GIPVVAAVDGACLGGGLELAMACH
tr|A0A1H8PPF9|A0A1H8PPF9_9GAMM       GADIE.MLQACRSAEEVTELSRAGQAFFDRIANL..NVPVVAAINGMCLGGGLELALACH
tr|A0A6H0J0V7|A0A6H0J0V7_9GAMM       GADID.MLRSCTSAAEVTELSRAGQAFMDRIANF..RVPVVAAIDGSCLGGGLELALACH
tr|V5EZX6|V5EZX6_9VIBR               GADVR.MLDACKTATQATELATTGQKMFAKLGEF..PFSVVAAIHGPCLGGGLELALACD
tr|A0A6I1QBM2|A0A6I1QBM2_9VIBR       GADVR.MLDACKTATQATELATTGQKMFAKLGEF..PFSVVAAIHGPCLGGGLELALACD
tr|A0A511QRL5|A0A511QRL5_9VIBR       GADVR.MLDACGTAQQATELATTGQQMFARLGEF..PFSVVSAIHGPCLGGGLELALACD
tr|A0A7Y0JZP4|A0A7Y0JZP4_9VIBR       GADVR.MLDACSTAEQATELATTGQKMFARLGEF..PFSVVSAIHGPCLGGGLELALACD
tr|A0A193KD24|A0A193KD24_9VIBR       GADVR.MLDACSTAEQATELATTGQKMFARLGEF..PFSVVSAIHGPCLGGGLELALACD
tr|A0A1S1HM72|A0A1S1HM72_PROST       GADIS.MIANCTSKEEASELSREGHILFDKLENY..PLPIIAAIHGACLGGGLELALACH
tr|A0A7T8I613|A0A7T8I613_9GAMM       GADIS.MIANCTSKEEASELSREGHILFDKLENY..PLPIIAAIHGACLGGGLELALACH
tr|A0A140NIF0|A0A140NIF0_PROSM       GADIS.MIANCQSKEQASELSREGHLLFEKLENY..PLPIVAAIHGACLGGGLELALACH
tr|A0A379GMB7|A0A379GMB7_PROST       GADIS.MIANCQSKEQASELSREGHLLFEKLENY..PLPIVAAIHGACLGGGLELALACH
tr|A0A379H393|A0A379H393_PROST       GADIS.MIANCQSKEQASELSREGHLLFEKLENY..PLPIVAAIHGACLGGGLELALACH
tr|B2Q0L5|B2Q0L5_PROST               GADIS.MIANCQSKEQASELSREGHLLFEKLENY..PLPIVAAIHGACLGGGLELALACH
tr|A0A6I3JUE9|A0A6I3JUE9_9GAMM       GADIS.MIADCKSKEEASELSREGHKLFDKLENY..PLPIVAAIHGACLGGGLELALACH
tr|A0A7D4P594|A0A7D4P594_YERMW       GADIT.MIAACRTAQDARKLAQKGQSILAQIAAF..PVPVVAAIHGACLGGGLELALACH
tr|A0A7U7IVK6|A0A7U7IVK6_YEREN       GADIT.MIAACHTAQDARILAQKGQSILAQIAAF..PVPVVAAIHGACLGGGLELALACH
tr|A0A447RIF5|A0A447RIF5_YEREN       GADIT.MIAACHTAQDARILAQKGQSILAQIAAF..PVPVVAAIHGACLGGGLELALACH
tr|A0A7H4ZLV9|A0A7H4ZLV9_YERP4       GADIT.MIAACHTAQDARILAQKGQSILAQIAAF..PVPVVAAIHGACLGGGLELALACH
tr|A0A0H3NX46|A0A0H3NX46_YERE1       GADIT.MIAACHTAQDARILAQKGQSILAQIAAF..PVPVVAAIHGACLGGGLELALACH
tr|A0A7T9XUV7|A0A7T9XUV7_YEREN       GADIT.MIAACHTAQDARILAQKGQSILAQIAAF..PVPVVAAIHGACLGGGLELALACH
tr|A0A7U7IYV8|A0A7U7IYV8_YEREN       GADIT.MIAACHTAQDARILAQKGQSILAQIAAF..PVPVVAAIHGACLGGGLELALACH
tr|A0A0H5G7G2|A0A0H5G7G2_YEREN       GADIT.MIAACHTAQDARILAQKGQSILAQIAAF..PVPVVAAIHGACLGGGLELALACH
tr|A0A2A7TDI2|A0A2A7TDI2_YERKR       GADIS.MIAACRTAQDARVLAQKGQSILAQIAAF..PVPVVAAIHGACLGGGLELALACH
tr|A0A0T9M261|A0A0T9M261_YERKR       GADIS.MIAACRTAQDARVLAQKGQSILAQIAAF..PVPVVAAIHGACLGGGLELALACH
tr|A0A0A0CMP1|A0A0A0CMP1_PHOLU       GADIS.MIAGCKTKEDARDLAEKGQKLFSQIANY..PLPVVAAIHGACLGGGLELALACH
tr|A0A329VG55|A0A329VG55_9GAMM       GADIS.MIAGCKTKEDARDLAEKGQKLFSQIANY..PLPVVAAIHGACLGGGLELALACH
sp|Q7N288|FADJ_PHOLL                 GADIS.MIAGCKTKEDARDLAEKGQKLFSQIANY..PLPVVAAIHGACLGGGLELALACH
tr|A0A6L9JMX5|A0A6L9JMX5_PHOLM       GADIS.MIAGCKTKEDARDLAEKGQKLFSQIANY..PLPVVAAIHGACLGGGLELALACH
tr|A0A7X5HQ29|A0A7X5HQ29_PHOLM       GADIS.MIAGCKTKEDARDLAEKGQKLFSQIANY..PLPVVAAIHGACLGGGLELALACH
tr|A0A1C0U051|A0A1C0U051_9GAMM       GADIS.MIAGCKTKEEAQDLAEKGQKLFSQIANY..PLPVVAAIHGACLGGGLELALACH
tr|A0A7X5QF62|A0A7X5QF62_9GAMM       GADIS.MIAGCKTKEEAQDLAEKGQKLFSQIANY..PLPVVAAIHGACLGGGLELALACH
```

```
tr|A0A2D0KWM9|A0A2D0KWM9_9GAMM  GADIT.MIAGCQTQQEATALAEKGQELFDKIASY..SLPIVAAIHGACLGGGLEFALACH
tr|A0A2D0LAI1|A0A2D0LAI1_9GAMM  GADIT.MIAGCQTQQEATALAEKGQELFDKIASY..SLPIVAAIHGACLGGGLEFALACH
tr|A0A2D0IXC8|A0A2D0IXC8_XENBU  GADIT.MIAGCQTQQEATKLAEKGQELFARIANY..SLPIVAAIHGACLGGGLELALACH
tr|W1JAM5|W1JAM5_9GAMM          GADIT.MIAGCQTQQEATKLAEKGQELFARIANY..SLPIVAAIHGACLGGGLELALACH
tr|A0A3D9UED3|A0A3D9UED3_9GAMM  GADIT.MIAGCQTQQEATKLAEKGQELFARIANY..SLPIVAAIHGACLGGGLELALACH
tr|A0A1I3JCA9|A0A1I3JCA9_9GAMM  GADIS.MIANSQTQEEASALAEKGQKLFTQIANY..SLPIVAAIHGACLGGGLEFALACH
tr|A0A068QUL9|A0A068QUL9_9GAMM  GADIS.MIAHCQSPQEATELAEKGQKLFAQIADY..PLPIVAAIHGACLGGGLELALACH
tr|A0A0M0TCH6|A0A0M0TCH6_9GAMM  GADIS.MIAHCQSQEEATALAEKGQKLFAQIASY..PLPIVAAIHGACLGGGLELALACH
tr|A0A1Q5U854|A0A1Q5U854_9GAMM  GADIS.MIANCQTQEEATKLAEKGQKLFAQIANY..PLPVVAAIHGACLGGGLELALACH
tr|A0A2D0ISP1|A0A2D0ISP1_9GAMM  GADIS.MIANCQTQEEATALAEKGQKLFAQIANY..PLPVVAAIHGACLGGGLELALACH
tr|A0A1Q5TUI8|A0A1Q5TUI8_9GAMM  GADIS.MIANCQTQEEATKLAEKGQKLFAQIASY..SLPVVAAIHGACLGGGLELALACH
tr|A0A2D0KJI7|A0A2D0KJI7_9GAMM  GADIS.MIANCQTQEEATKLAEKGQKLFAQLANY..PLPVVAAIHGACLGGGLELALACH
tr|A0A1Y2SB97|A0A1Y2SB97_9GAMM  GADIS.MIAGCQTQAEATELAEKGQKLFAQIANY..PLPVVAAIHGACLGGGLELALACH
tr|A0A1I5DWI7|A0A1I5DWI7_9GAMM  GADIS.MIAGCQTQEEATELAEKGQKLFAQIANY..PLPVVAAIHGACLGGGLELALACH
tr|A0A1I7GWY6|A0A1I7GWY6_9GAMM  GADIS.MIAGCQTQEEATELAEKGQKLFATIANY..PLPIVAAIHGACLGGGLELALACH
tr|D3VKY8|D3VKY8_XENNA          GADIS.MIAGCQTQEEATELAEKGQKLFAQIANY..PLPIVAAVHGACLGGGLELALACH
tr|A0A2G0Q6Q9|A0A2G0Q6Q9_9GAMM  GADIS.MIASCQTQKEATELAEKGQKLFAQIANY..PLPVVSAIHGACLGGGLELALACH
tr|A0A2D0JU24|A0A2D0JU24_9GAMM  GADIS.MIAGCQSQEEATELAEKGQKLFAQIANY..PLPVVAAIHGACLGGGLELALACH
tr|A0A0J5FN38|A0A0J5FN38_9GAMM  GADIS.MIAGCQTQEEATELAEKGQKLFAQIANY..PLPVVAAIHGACLGGGLELALACH
tr|A0A432XLD4|A0A432XLD4_9GAMM  GADIS.MIDACETAADTESLARQGQAMFDRIEQL..NVPVVAAIDGACLGGGLELALACH
tr|A0A2D8HU84|A0A2D8HU84_9GAMM  GADIN.MIDGCENAVDAESLARKGQAMFDRIEQL..NVPVVAAINGACLGGGLELAMACH
tr|A0A656X1Q2|A0A656X1Q2_9GAMM  GADIN.MIDGCENAVDAESLARKGQAMFDRIEQL..NVPVVAAINGACLGGGLELAMACH
tr|A0A4Q1QH79|A0A4Q1QH79_9GAMM  GADIN.MINDCETAADTESLARKGQAMFDRIEQL..DVPVVAAINGACLGGGLELAMACH
tr|A0A1G7LQE6|A0A1G7LQE6_9GAMM  GADIN.MINDCETAADTESLARKGQAMFDRIEQL..DVPVVAAINGACLGGGLELAMACH
tr|A0A432YVG7|A0A432YVG7_9GAMM  GADIN.MINDCETAADTESLARKGQAMFDRIEQL..DVPVVAAINGACLGGGLELAMACH
tr|A0A1J5N0P5|A0A1J5N0P5_9GAMM  GADIT.MLDSCQSRDDVLAISTMGQRMFDRIENM..KVPVVAAINGPCLGGGLELAMACH
tr|A0A0D8D548|A0A0D8D548_9GAMM  GADIN.MLNACQTAEEATALSRQGQMLFDQLEKM..DIPVIAAIHGPCLGGGLELAMACH
tr|A0A3E0TNM2|A0A3E0TNM2_9GAMM  GADIN.MLASCKSAAEVTSLSRHGHMVFDQMEKF..PKPIIAAINGPCLGGGLELAMACH
tr|A0A3E0UD67|A0A3E0UD67_9GAMM  GADIN.MLASCTTAEEATTLSRQGQMVFDQMEKF..PKPIIAAINGPCLGGGLELAMACH
tr|A0A3E0U0P4|A0A3E0U0P4_9GAMM  GADIN.MLASCTTAEEATTLSRQGQMVFDQMEKF..PKPIIAAINGPCLGGGLELAMACH
tr|A0A0M2V8B1|A0A0M2V8B1_9GAMM  GADIS.MLDSCQSAEQAQDIARMGQQMFDRLEQL..TVPLVAAIHGPCLGGGLELAMACH
tr|A0A285ITY5|A0A285ITY5_9GAMM  GADIS.MLDSCETAEQAEDIARMGQQMFDRIEQL..KIPVVAAINGPCLGGGLELAMACH
tr|A0A486XTB9|A0A486XTB9_9GAMM  GADIS.MLDGCKTAEEAQAIGRMGQQMFDQLEQL..AIPLVAAIHGPCLGGGLELAMACH
tr|I1E1G1|I1E1G1_9GAMM          GADIS.MLDGCKTAEEAQAIGRMGQQMFDQLEQL..PIPLVAAIHGPCLGGGLELAMACH
tr|A0A1H6KJ68|A0A1H6KJ68_9GAMM  GADIS.MLDGCKTAEEAQAIGRMGQQMFDQLEQL..PIPLVAAIHGPCLGGGLELAMACH
tr|A0A0X3Y764|A0A0X3Y764_9GAMM  GADIS.MLDGCKTAEEAQAIGRMGQQMFDQLEQL..SIPLVAAIHGPCLGGGLELAMACH
tr|A0A2N1YEV4|A0A2N1YEV4_9GAMM  GADIS.MLDGCKTAEEAQAIGRMGQQMFDQLEQL..PIPLVAAIHGPCLGGGLELAMACH
tr|A0A3P3QCM3|A0A3P3QCM3_9GAMM  GADIS.MLDGCTTAQQAEDIGRMGQLVFNQLEAL..SIPLVAAIHGPCLGGGLELALACH
tr|F7NT72|F7NT72_9GAMM          GADIS.MLDGCTTAQQAEDIGRMGQLVFNQLEAL..SIPLVAAIHGPCLGGGLELALACH
tr|A0A3S2TW63|A0A3S2TW63_9GAMM  GADIS.MLDGCTTAQQAEDIGRMGQLVFNQLEAL..AIPLVAAIHGPCLGGGLELALACH
tr|A0A3D5BGW7|A0A3D5BGW7_9GAMM  GADIT.MLDGCKTAQEATDIAAMGQQMFSQLEAL..NIPLIAAIHGPCLGGGLELAMACH
tr|A0A5C7TI17|A0A5C7TI17_9GAMM  GADIS.MLDKCKTAAEATAIASMGQQMFNELEKL..HIPLIAAIHGPCLGGGLELAMACH
tr|A0A0U4W982|A0A0U4W982_9GAMM  GADIS.MLDKCKTAAEATAIASMGQRMFNELEKL..HIPLIAAIHGPCLGGGLELAMACH
tr|A0A2I0FCM2|A0A2I0FCM2_9GAMM  GADVT.MIDACETAADAEQLSLEGHRVMGELEAL..NIPVVAAIHGPCLGGGLELALACH
tr|A0A2G2IVL1|A0A2G2IVL1_9GAMM  GADVT.MIDACETAADAEKLSLEGHRVMGELEAL..NIPVVAAIHGPCLGGGLELALACH
tr|A6FI21|A6FI21_9GAMM          GADVT.MIDACETAADAEKLSLEGHRVMGELEAL..NIPVVAAIHGPCLGGGLELALACH
tr|A0A4U1BNR2|A0A4U1BNR2_9GAMM  GADIN.MLASCETAEDVYKLSRDGHKLLNELASQ..RLPVVAAIHGPCLGGGLELALACD
tr|E1SQ60|E1SQ60_FERBD          GADIA.MLAACKTTDDAYQISRGAQQLFNELAAL..KVPVVAAIDGPCLGGGLELALACD
tr|A0A4Y6IZ09|A0A4Y6IZ09_9GAMM  GADIS.MLDACSTAGDARELSKQGHEVFFALESL..TIPVVAAINGACLGGGLELALACH
tr|A0A0C3QSZ7|A0A0C3QSZ7_9GAMM  GADIS.MLDACATAEDARELSKQGHEVFLALESL..SIPVVAAIHGACLGGGLELALACH
tr|A0A6L7HXW0|A0A6L7HXW0_9GAMM  GADIS.MLDACATAEDARELSKQGHEVFLALESL..SIPVVAAINGACLGGGLELALACH
sp|A3QFP3|FADJ_SHELP            GADIS.MLDACATAEDARELSKQGHEVFFALESL..SIPVVAAINGACLGGGLELALACH
tr|A0A1E5IXH7|A0A1E5IXH7_SHECO  GADIS.MLDACTSADDAKQLSQQGHEVFLALEAL..TIPVVAAINGPCLGGGLELALACH
tr|A0A411PKQ0|A0A411PKQ0_9GAMM  GADIT.MLDACTSVEAAKELSQQGHVVFNELENL..KIPVVAAINGACLGGGLELALACH
tr|A0A6G9QKM3|A0A6G9QKM3_9GAMM  GADIS.MLAACKTEQDAKDLSQQGHVVFNDLEGL..TIPVVAAINGACLGGGLELALACH
tr|A0A6P1UL63|A0A6P1UL63_9GAMM  GADIT.MLAACKSEADAKALSQQGHVVFSELEGL..SIPVVAAINGACLGGGLELALACH
tr|A0A2N1ERI9|A0A2N1ERI9_9GAMM  GADIT.MLAACKSEADAKALSQQGHVVFSELEGL..SIPVVAAINGACLGGGLELALACH
tr|A0A7W4FU55|A0A7W4FU55_9GAMM  GADIT.MLAACKSEADAKALSQQGHVVFSELEGL..SIPVVAAINGACLGGGLELALACH
sp|Q8ECP7|FADJ_SHEON            GADIS.MLDACQTAGDAKALSQQGHVVFNELEAL..NIPVVAAIHGACLGGGLELALACH
tr|A0A501XZY8|A0A501XZY8_9GAMM  GADIS.MLDACQTAGDAKALSQQGHVVFNELEAL..NIPVVAAIHGACLGGGLELALACH
tr|A0A2W5DCZ0|A0A2W5DCZ0_SHEOE  GADIS.MLDACQTAGDAKALSLQGHVVFNELEAL..NIPVVAAIHGACLGGGLELALACH
tr|A0A1E3V3C8|A0A1E3V3C8_9GAMM  GADIS.MLDACQTAGDAKALSQQGHVVFNELEAL..NIPVVAAIHGACLGGGLELALACH
tr|A0A1Z4AI20|A0A1Z4AI20_9GAMM  GADIS.MLDACQTAGDAKALSQQGHVVFNELEAL..NIPVVAAIHGACLGGGLELALACH
tr|A0A7X9LJL9|A0A7X9LJL9_9GAMM  GADIS.MLDACQTAGDAKALSQQGHVVFNELEAL..NIPVVAAIHGACLGGGLELALACH
tr|A0A073KMY5|A0A073KMY5_9GAMM  GADIS.MLDACQTAGDAKALSQQGHVVFNELEAL..NIPVVAAIHGACLGGGLELALACH
sp|A0KV76|FADJ_SHESA            GADIS.MLDACQTAGDAKALSQQGHVVFNELEAL..NIPVVAAIHGACLGGGLELALACH
tr|A0A220UTH7|A0A220UTH7_9GAMM  GADIS.MLDACQTAGDAKALSQQGHVVFNELEAL..NIPVVAAIHGACLGGGLELALACH
tr|A0A5B8R6W1|A0A5B8R6W1_9GAMM  GADIS.MLDACQTAGDAKALSQQGHVVFNELEAL..NIPVVAAIHGACLGGGLELALACH
tr|V1DAI4|V1DAI4_9GAMM          GADIS.MLDACQTAGDAKALSQQGHVVFNELEAL..NIPVVAAIHGACLGGGLELALACH
tr|A0A448CPQ4|A0A448CPQ4_SHEPU  GADIS.MLDACQTAGDAKALSQQGHVVFNELEAL..NIPVVAAIHGACLGGGLELALACH
tr|A0A252ERQ3|A0A252ERQ3_SHEPU  GADIS.MLDACQTAGDAKALSQQGHVVFNELEAL..NIPVVAAIHGACLGGGLELALACH
sp|Q0HKD1|FADJ_SHESM            GADIS.MLDACQTAGDAKALSQQGHVVFNELEAL..NIPVVAAIHGACLGGGLELALACH
sp|Q0HWN3|FADJ_SHESR            GADIS.MLDACQTAGDAKALSQQGHVVFNELEAL..NIPVVAAIHGACLGGGLELALACH
tr|F7RQE3|F7RQE3_9GAMM          GADIS.MLDACKTASDAKALSQQGHVVFNELEAL..TIPVVAAIHGACLGGGLELALACH
tr|B8EE98|B8EE98_SHEB2          GADIS.MLDACETAGDAKALSQQGHVVFNELEAL..KIPVVAAIHGACLGGGLELALACH
sp|A6WQ25|FADJ_SHEB8            GADIS.MLDACKTAGDAKALSQQGHVVFNELEAL..TIPVVAAIHGACLGGGLELALACH
tr|A0A448EK41|A0A448EK41_9GAMM  GADIS.MLDACKTAGDAKALSQQGHVVFNELEAL..TIPVVAAIHGACLGGGLELALACH
tr|A0A553JHX1|A0A553JHX1_SHEHA  GADIS.MLDACETAADAKQLSQQGHVVFNELESL..TIPVVAAINGACLGGGLELAIACH
tr|B8CPY6|B8CPY6_SHEPW          GADIS.MLDACETAMDAKLLSQQGHVVFNELENL..SIPVVAAINGACLGGGLELALACH
tr|A0A431WFC4|A0A431WFC4_9GAMM  GADIS.MLDACKTAADAKLLSQQGHVVFNELEGL..KIPVIAAINGACLGGGLELALACH
tr|A8FTR7|A8FTR7_SHESH          GADIS.MLDACKTAADAKQLSQQGHVVFNELESL..KIPVIAAINGACLGGGLELALACH
tr|A0A431WNL0|A0A431WNL0_9GAMM  GADIS.MLDACKTAADAKLLSQQGHLVFNELESL..KIPVIAAINGACLGGGLELALACH
tr|A0A550AEC5|A0A550AEC5_9GAMM  GADIS.MLDACKSAADAKALSQQGHVVFNELEAL..NIPVVAAIDGVCLGGGLELALACH
tr|A0A7L4WW90|A0A7L4WW90_9GAMM  GADIS.MLDACKSAADAKALSQQGHVVFNELEAL..NIPVVAAIDGVCLGGGLELALACH
tr|A9DDU3|A9DDU3_9GAMM          GADIS.MLDACTSVEDAKALSQQGHRVFNELEAM..SIPVVAAIDGVCLGGGLELALACH
tr|A0A330M2B9|A0A330M2B9_9GAMM  GADIS.MLDACTSVEDAKALSQQGHVVFNELEAL..SIPVVAAIDGVCLGGGLELALACH
tr|A0A5N8UFC7|A0A5N8UFC7_9GAMM  GADIS.MLDACTSVEDAKALSQQGHIVFNELEAM..SIPVVAAIDGVCLGGGLELALACH
tr|A0A1S6HN57|A0A1S6HN57_9GAMM  GADIS.MLDACTSVADAKALSQQGHIVFNELEAL..SIPVVAAIDGVCLGGGLELALACH
tr|D4ZMH7|D4ZMH7_SHEVD          GADIS.MLDACTSVEDAKALSQQGHIVFNELEAL..SIPVVAAIDGVCLGGGLELALACH
tr|A0A3L8Q213|A0A3L8Q213_9GAMM  GADVK.MLDACKTKDDVTAITKQGYEVFAELEGL..SFPVVAAIHGVCLGGGLELAMACH
tr|A0A3A6U4N9|A0A3A6U4N9_9GAMM  GADIS.MLDKCKTSEDAKTLSQQGHRVFFDLENL..PFPVIAAIHGSCLGGGLELAMACH
tr|A0A4Q5MA37|A0A4Q5MA37_9GAMM  GADIK.MLDACATAEDARTLSKQGHEVFAELESL..PFPVVAAIHGNCLGGGLELAMACH
tr|A0A1L6LSX5|A0A1L6LSX5_9DELT  GADIE.MLKALGTAAEATEMCRAGHRSIEKLVSS..PKPVVAAVHGAALGGGFEVALACH
tr|A0A2W4L9B9|A0A2W4L9B9_9PROT  GAKLE.MIQRVQDAKEAEALSRTAQDMFDEIAAS..EKPVVVAIDGAALGGGLELALACH
tr|A0A2W4M4E6|A0A2W4M4E6_9PROT  GADLK.LLRVISSREKAEELSRAGQRALERLASS..KKPFVAAIHGACLGGGLEVALACH
tr|A0A6I2GRX9|A0A6I2GRX9_9DELT  GAKVD.YLQTLTTAAEAEAVSRRAQEGFEQLEAAARTKPVVAAIHGAALGGGLEWALACS
tr|A0A0H4WMK2|A0A0H4WMK2_9DELT  GAKID.FLQTIKTAEEATAISRNGQEGFDKLDAF..PKPVVAAIHGACLGGGLEWALACD
tr|F8CJ36|F8CJ36_MYXFH          GAKID.FLHTIKTAEEATAISRNGQEGFDKLDAF..PKPVVAAIHGACLGGGLEWALACD
tr|A0A250K0F1|A0A250K0F1_9DELT  GAKID.FLQTIKTAEEATAISRNGQEGFDKLDAF..PKPVVAAIHGACLGGGLEWALACD
tr|A0A7Y6WFZ2|A0A7Y6WFZ2_9DELT  GAKID.FLQTIKTAEEATAISRNGQEGFDRLDAF..PKPVVAAIHGACLGGGLEWALACD
tr|A0A7Y7C660|A0A7Y7C660_9DELT  GAKID.FLQTIKTAEEATAISRNGQEGFDKLDAF..PKPVVAAIHGACLGGGLEWALACD
tr|A0A7Y4JFH2|A0A7Y4JFH2_MYXXA  GAKID.FLQTIKTAEEATAISRNGQEGFDKLAAF..PKPVVAAIHGACLGGGLEWALACD
tr|A0A4Y6CZQ9|A0A4Y6CZQ9_MYXXA  GAKID.FLQTIKTAEEATAISRNGQEGFDKLAAF..PKPVVAAIHGACLGGGLEWALACD
```

```
tr|A0A7Y4IKV5|A0A7Y4IKV5_MYXXA  GAKID.FLQTIKTAEEATAISRNGQEGFDKLADF..PKPVVAAIHGACLGGGLEWALACD
tr|A0A511HHB0|A0A511HHB0_9DELT  GAKID.FLQTIKTAEEATAISRNGQEGFDKLADF..PKPVVAAIHGACLGGGLEWALACD
tr|A0A4Y6CKY7|A0A4Y6CKY7_MYXXA  GAKID.FLQTIKTAEEATAISRNGQEGFDKLADF..PKPVVAAIHGACLGGGLEWALACD
tr|Q1D1F2|Q1D1F2_MYXXD          GAKID.FLQTIKTAEEATAISRNGQEGFDKLADF..PKPVVAAIHGACLGGGLEWALACD
tr|A0A7Y4MA14|A0A7Y4MA14_MYXXA  GAKID.FLQTIKTAEEATAISRNGQEGFDKLADF..PKPVVAAIHGACLGGGLEWALACD
tr|A0A7T8Y4N9|A0A7T8Y4N9_MYXXA  GAKID.FLQTIKTAEEATAISRNGQEGFDKLADF..PKPVVAAIHGACLGGGLEWALACD
tr|L7UE67|L7UE67_MYXSD          GAKLD.LLQSLKTAEEATAISLQGQASFDRMEAF..PKPILAAIHGACLGGGLEWALACH
tr|A0A511T9X1|A0A511T9X1_MYXFU  GAKID.YLQTLKTAEEATAMSQQGQAGFDRLEAF..PKPVVAAIHGACLGGGLEWALACH
tr|A0A7Y7C9C4|A0A7Y7C9C4_9DELT  GAKID.YLQTLKTAEEATLMSQQGQAGFDRLEAF..PKPVVAAIHGACLGGGLEWALACH
tr|A0A540X7W8|A0A540X7W8_9DELT  GAKID.YLQTLKTAEDATAMSQQGQVGFDRLEAF..PKPVVAAIHGACLGGGLEWALACH
tr|A0A3A5FK19|A0A3A5FK19_9DELT  GAKID.FLQTLRSAEEATAISRNGQQGFEQLDAF..PKPVVAAIHGSCLGGGLEWALACD
tr|A0A3A8JQL9|A0A3A8JQL9_9DELT  GANID.FLQTIKTPADVEAISRGAHAQFDQLEAF..SKPVVAAIHGACLGGGLEWVLACH
tr|A0A7Y4NFA4|A0A7Y4NFA4_9DELT  GANID.FLQTLKTPAEVEAISRGAHEQFDRLESF..SKPVVAAIHGACLGGGLEWVLACH
tr|A0A3A8HDQ7|A0A3A8HDQ7_9DELT  GANID.FLQTIKTPADVEAISRGAHAQFDQLEAF..SKPVVAAIHGACLGGGLEWVLACH
tr|A0A3A8GR90|A0A3A8GR90_9DELT  GANID.FLQTIKSPADVEAISRAAHAEFDRLEAY..SKPVVAAIHGACLGGGLEWVLACH
tr|A0A3A8SBD7|A0A3A8SBD7_9DELT  GANID.FLQTIKSPADVEAISRAAHAEFDRLEAF..SKPVVAAIHGACLGGGLEWVLACH
tr|A0A3A8T0I8|A0A3A8T0I8_9DELT  GANID.FLQTIKSPADVEAISRAAHAEFDRLEAF..SKPVVAAIHGACLGGGLEWALACH
tr|A0A7X5BU07|A0A7X5BU07_9DELT  GANID.FLQTLKSPADVEAISRAAHAEFDRLEAF..SKPVVAAIHGACLGGGLEWALACH
tr|A0A7Y1RVL2|A0A7Y1RVL2_9DELT  GANID.FLQTIKSPADVEAISRAAHAEFDRLEAF..SKPVVAAIHGACLGGGLEWALACH
tr|A0A3A8THN2|A0A3A8THN2_9DELT  GANID.FLQTIKSPADVEAISRAAHAEFDRLEAF..SKPVVAAIHGACLGGGLEWALACH
tr|A0A7Y1RX76|A0A7Y1RX76_9DELT  GANID.FLQTIKSPADVEAISRAAHAEFDRLEAF..SKPVVAAIHGACLGGGLEWALACH
tr|A0A3A8RA58|A0A3A8RA58_9DELT  GANID.FLQTLKSPADVEAISRGAHEQFDRLESF..SKPVVAAIHGACLGGGLEWVLACH
tr|A0A410RPB6|A0A410RPB6_CORCK  GANID.FLQTLKSPADVEAISRAAHAEFDRLESF..SKPVVAAIHGACLGGGLEWVLACH
tr|A0A7Y4J474|A0A7Y4J474_CORCK  GANID.FLRTLKTPADVEAISRGAHEQFDRLEAF..SKPVVAAIHGACLGGGLEWVLACH
tr|A0A3A8I9Z6|A0A3A8I9Z6_9DELT  GANID.FLQTLKTPADVEAISRGAHEQFDRLESF..SKPVVAAIHGACLGGGLEWVLACH
tr|H8MKE9|H8MKE9_CORCM          GANID.FLQTLKTPADVEAISRGAHEQFDRLESF..SKPVVAAIHGACLGGGLEWVLACH
tr|A0A3A8H102|A0A3A8H102_9DELT  GANID.FLQTLKTPADVEAISRGAHEQFDRLEAF..SKPVVAAIHGACLGGGLEWVLACH
tr|A0A554FW33|A0A554FW33_9DELT  GANID.FLQTLKSPDEVMAISYAAYTEFDRLESF..SKPVVAAIHGACLGGGLEWALACH
tr|A0A3A8NPC0|A0A3A8NPC0_9DELT  GANID.FLQTIKSPDEVVAISRAAHAQFDRLEAF..PKPVVAAIHGACLGGGLEWALACH
tr|A0A3A8JUV3|A0A3A8JUV3_9DELT  GANID.FLQTIKSADEVVAISRGAHAQFDRLEAF..PKPVVAAIHGACLGGGLEWALACH
tr|A0A3A8NEX6|A0A3A8NEX6_9DELT  GANID.FLQTIKSADEVVAISRTAHAQFDRLEAF..PKPVVAAIHGACLGGGLEWALACH
tr|A0A3A8JEU8|A0A3A8JEU8_9DELT  GANID.FLQTIKSPDDVVAISRAAHAEFERLEEF..SKPVVAAIHGACLGGGLEWALACH
tr|A0A3A8LI76|A0A3A8LI76_9DELT  GANID.FLQTIKSPDEVVAISRAAHAEFDRLEAF..SKPVVAAIHGACLGGGLEWALACH
tr|A0A085WXN8|A0A085WXN8_9DELT  GAKID.FLQTIKTAAEATEISRKGQEGFDRLDAF..PKPVVAAIHGACLGGGLEWALACD
tr|A0A2T4V0M5|A0A2T4V0M5_9DELT  GAKID.FLQTIKSAAEATAVSRQGQQGFDRLDAF..PKPVVAAIHGACLGGGLEWALACD
tr|A0A0G2ZSW5|A0A0G2ZSW5_9DELT  GAKID.FLQTLKSAAEATAASRQGQQGFDRLEAF..SKPVVAAIHGACLGGGLEWALACD
tr|A0A3M2DKY0|A0A3M2DKY0_9DELT  GANID.MLRRAATPADAEALSRSGQRAMQRIEDL..RVPVVAAIHGACLGGGLELAMACH
tr|A0A661NQ58|A0A661NQ58_9DELT  GADIS.MLEAAKTAADATEISQRGQGALNRVEAL..GKPVVAAIHGPCLGGGLEVALACH
tr|A0A520YD99|A0A520YD99_9DELT  GADIT.MLEAVTSAEEGERVSREGHKVMNQIENC..PKPVVAAIHGVALGGGLEVALACH
tr|A0A7Y3BRE4|A0A7Y3BRE4_9DELT  GADIT.MLEAVTSAEEGERVSREGHKVMNQIENC..PKPVVAAIHGVALGGGLEVALACH
tr|A0A2D9TF90|A0A2D9TF90_9DELT  GADIG.MLDAIDTAAGAAAVSRAGQAAMDRVAAF..PKPVVAAIDGACLGGGLEVALACH
tr|A0A2E0TP32|A0A2E0TP32_9DELT  GANID.MLARAKSAAEASELSRQGQKAMDRIEAF..PKPVVAAIDGACLGGGLEVALACH
tr|A0A2E4Y3V1|A0A2E4Y3V1_9PROT  GADIK.MIKEFKTKEDVVKTSREGHELFKRIEKH..KKPSVAAINGPCLGGGLEVALSCH
tr|A0A2E6VRH4|A0A2E6VRH4_9DELT  GADID.ELSNAANAEAIKTLSQNGQAFFDRLESS..PKPIIAAIHGACMGGGLELALACH
tr|A0A1F9FB59|A0A1F9FB59_9DELT  GADIS.MIQTRKSAAEVERLSRELQQQLFLLEQS..SKPIVAAISGACMGGGLEIALACH
```

```
ECHA_HUMAN
                                     160       170       180       190       200       210
ECHA_HUMAN                           YRIATKDRKTVLGTPEVLLGALPGAGGTQRLPKMVGVPAALDMMLTGRSIRADRAKKMGL
tr|A0A4R6XLA8|A0A4R6XLA8_9GAMM       YRIASTDKKTVFGLPEMKLGLLPGGGGTQRLVKLTNLRYALDALLTGKNIYPFSAKKSGL
tr|A0A6A0IJT2|A0A6A0IJT2_9BACT       YRLATDDPKTALALPEVMLGLLPAGGGTQRLPRLVGLPAALPMLLTGRRLRARQALRCGL
tr|A0A7V3QZU7|A0A7V3QZU7_9BACT       YRLAADDPKVVLGLPEVQLGLLPAGGGTVRLPRLVGLPAALPMMLTGRRVRARKALKMGL
tr|A0A7V4SYJ6|A0A7V4SYJ6_9BACT       YRLASDDPATVLALPEVTLGLLPAAGGTQRLPPLVGLPAALPMMLTGQRVRSRKALRMGL
tr|A0A2A8D2N6|A0A2A8D2N6_9BACT       YRICSTHDKTKMALPEVQLGLLPGGGGTQYLPRLVGVQQALTMMLTGKNIYPRKAKRIGL
tr|A0A2N1TGP0|A0A2N1TGP0_9SPIR       YRIASDSPKTQMGLPEVKLGLIPAGGGTQRLPRLIGIQKALSAMLQGTSFRPKQAKKIGL
tr|A0A1G3QGW4|A0A1G3QGW4_9SPIR       YRIAADSTKTVMGLPEVMLGLLPAGGGTQRLPRLIGLKEALPLMLTGKNIRVRKAKKLGL
tr|A0A1G3QTY3|A0A1G3QTY3_9SPIR       YRIAADSTGTVMGFPEVMLGLLPAGGGTQRLPRLIGLIQALPLMLTGKNVRIRTAKKLGL
tr|A0A7X9L359|A0A7X9L359_9DELT       YRIATDSPKTTLGLPEVQLGLIPAAGGTQRLPRLIGLQNALPLMLTGRNLRPKKALKMGL
tr|A0A522CJY0|A0A522CJY0_9SPIR       YRIASDSTQTQMGLPEVQIGLLPAAGGTHRLPRLIGLRAAMPLMLTGKSIRVKKAKQLGL
tr|A0A2N1RRJ3|A0A2N1RRJ3_9SPIR       YRIAVNSPDVVIGFPEVQLGLLPAGGGCQRLTRLIGLTSALPMMLTAKTLRAKKARKLGI
tr|A0A2N2KFN7|A0A2N2KFN7_9DELT       YRMAVNSPQTVLGFPEVQLGLLPAGGGTQRLPRLIGLTAALPMLLTAKNLRAKKAKKAGL
tr|A0A1V6AXX9|A0A1V6AXX9_9DELT       YRMAVNGPQTVLGFPEVQLGLLPAAGGTQRLPRLIGLTAALPMLLTARNLRVKKAKRAGL
tr|A0A2N2HZL0|A0A2N2HZL0_9DELT       YRMAVNSPKTVLGFPEVQLGLLPAAGGTQRLPRLIGLTAALPLVLTARNLRVKQASRAGL
tr|A0A5E8ARP3|A0A5E8ARP3_9BACT       GRVASNHSSTMMALPEVKLGLLPGLGGTYRLPKLVGVQNALSMILTGRNVYAFPALKMGL
tr|A0A662A7B1|A0A662A7B1_9BACT       GRIAAKDPKTMMGLPEVKLGLLPGMGGTQRLPKLVGVQAALDVMLTGKNIFASKARKIGL
tr|D7CV16|D7CV16_TRURR               YRVASQHPKTKFALPEVTLGLLPGLGGTQRLPRLVGVEQALDMALTGRSVYAKPALKMGL
tr|A0A3C2AKX4|A0A3C2AKX4_9FLAO       ARVASDNPKTIMALPEVKLGLLPGGGGTQRLPRLVGIQKALDMMLTGKNVFAYPAKKMGL
tr|A0A3B8ZLN3|A0A3B8ZLN3_9PLAN       ARVAADEG.TVLSLPEVKLGLLPGGGGTQRLTRLVGIQKALDMMLTGKNIYAHPAKKMGL
tr|A0A345UGV8|A0A345UGV8_9BACT       YRIATDDPATKFAVPEVKLGLLPGAGGTQRLPRLVGVPTALDMMLTGKNVYPRQAKRMGL
tr|A0A6C1P6A2|A0A6C1P6A2_9BACT       YRIGTDHKDTRFALPEVKLGLLPGAGGTQRLPRLVGVPKALDIMLTGKNVYPRQALKMGL
tr|A0A3M1X9W8|A0A3M1X9W8_9BACT       YRVASDDPATVLALPEVRLGLLPGGGGTQRLPRLIGIQRALNMMLTGKNVYPRQALRMGL
tr|A0A354C796|A0A354C796_9DELT       YRVASDDPKTILALPEVKLGLLPGGGGTQRLPHLIGLQAALDMMLTGKNIYPRQAMKMGL
tr|A0A3M1NUM8|A0A3M1NUM8_9BACT       ARIATDDPKTILGQPEVKLGLLPAAGGTQRLPRLVGLQKALDIMLTGKNVYPRQAKKMGL
tr|A0A3M2KRC8|A0A3M2KRC8_9BACT       YRIITDSPKTVLGQPEVRLGLLPAGGATQRLPRLVGLQRALDMMLTGKNIYPYPARKMGL
tr|A0A5S9IHR0|A0A5S9IHR0_9BACT       YRIVTDHPKTVLALPEVKLGLLPGGGGTQRLPRLIGIQKALGLMLTGRNVYPHKAKKIGL
tr|A0A6M1ST81|A0A6M1ST81_9BACT       YRVASKDSATTFGQPEVKLGLLPGGGGTQRLPRLIGLQNALTYELTGKNIYPRQAYKMGL
tr|A0A521AAE5|A0A521AAE5_9BACT       YRVASDHDKTTMGQPEVQLGLLPGGGGTQRLPRLIGIQNALTYLLTGKNIYPGQAYKMGL
tr|A0A1M4ZMD6|A0A1M4ZMD6_9BACT       YRVASDHDQTVMAQPEVQLGLLPGGGGTQRLPRLIGIQNAFTYLLTGKNIYPRKAYKMGL
tr|A0A2A2GCS2|A0A2A2GCS2_9BACT       YRVASKHSDTVMGQPEVQLGLLPGGGGTQRLPRLIGVQNALSYMLTGKNIYPRKAYKLGL
tr|A0A5D3YIH7|A0A5D3YIH7_9BACT       YRVASEHSDTVMGQPEVNLGLLPGGGGTQRLPRLIGIQNALTYMLTGKNIYPRKAYKLGL
tr|A0A6M1T9G0|A0A6M1T9G0_9BACT       YRVASEHSDTTMGQPEVQLGLLPGGGGTQRLPRLIGVQNALTYMLTGKTIYPRKAYKLGL
tr|A0A6A8Q1Y8|A0A6A8Q1Y8_9BACT       YRVCSDSSKTVFALPEVKLGLLPGTGGTQRLPRTISLQKALTYMLTGKNIYTRQAKRMGL
tr|A0A521BVJ3|A0A521BVJ3_9BACT       YRVCSDSSDTVFSLPEVKLGLLPGTGGTQRLPRRIGIQKALTYMLTGKNIYVRQARNTGL
tr|A0A359E0Y8|A0A359E0Y8_9BACT       YRVCSDSSKTIFALPEVKLGILPGTGGTQRLPRLIGLQNSLPYMLAGKNIYTRQAKRMGL
tr|A0A2D8CIM6|A0A2D8CIM6_9BACT       YRVCSDSSKTIFALPEVKLGILPGTGGTQRLPRLIGLQNSLPYMLAGKNIYTRQAKRMGL
tr|A0A2D9FWF5|A0A2D9FWF5_9BACT       YRACSDSSDTVFALPEVKLGLLPGTGGTQRLPRLIGIQNALPYMLTGKNMYVRQARKMGL
tr|A0A1B6YB70|A0A1B6YB70_9BACT       YRIASGHSKTIMALPEVKLGILPGTGGTQRLPRLIGIQKSLNYMLTGKNIYGRQAYKMGL
tr|A0A2D4ZTC0|A0A2D4ZTC0_9BACT       YRIVSDHSKTILGLPEVKLGILPGTGGTQRLPRLIGIQKSLNFMLTGKNIYARQAYKIGL
tr|A0A3D4UVW7|A0A3D4UVW7_9BACT       YRIVSDHSKTILGLPEVKLGILPGTGGTQRLPRLIGIQKSLNFMLTGKNIYARQAYKIGL
tr|A0A3F3I3M2|A0A3F3I3M2_9BACT       YRIVSDHSKTILGLPEVKLGILPGTGGTQRLPRLIGIQKSLNFMLTGKNIYARQAYKIGL
tr|A0A3D1G7L9|A0A3D1G7L9_9BACT       YRLASKHPKTVLALPEVKLGVIPGTGGTQRLPRLIGIQKALGYMLPGKNIYAHSAKKIGL
tr|A0A3M8G1Y8|A0A3M8G1Y8_9BACT       YRIATNHPKTVLALPEVKLGVIPGTGGTQRLPRTIGIQKALGYMLTGGNIYARTAKRIGL
tr|A0A5Q4F391|A0A5Q4F391_9BACT       YRIVSDHPSTKIALPEVKLGLLPGMGGTQRLPALIGIQKSLPYLLTGRNIYPRQAKRNGF
tr|A0A6I7NPV0|A0A6I7NPV0_9BACT       YRIVSDHPSTKIALPEVKLGLLPGMGGTQRLPRLIGIQKALTYMLTGKNMYSYQAHKMGF
tr|A0A651G1J2|A0A651G1J2_9BACT       SRIVSDDSSTKIGLPEVKLGLLPGMGGTQRLPRLIGIQKALGYMLPGKNMYSYQAKKTGF
tr|A0A371QRK5|A0A371QRK5_9BACT       YRIVSDHSDTKIGLPEVKLGLLPGMGGTQRLPRLIGLQKALPYLLTGKNMYPRHARRTGF
tr|A0A2N0VGJ5|A0A2N0VGJ5_9BACT       YRIVSDDSSTKIGLPEVQLGLLPGMGGTQRLPRLIGIQKSLTYMLTGKNMYTHQAKKTGF
tr|A0A316TYJ0|A0A316TYJ0_9BACT       YRIVSDHNATKIGLPEVKLGLLPGMGGTQRLPRLIGIQKALTYMLTGKNMYSYQARKTGF
tr|A0A651GLZ8|A0A651GLZ8_9BACT       YRIVSDHNSTKIGLPEVKLGLLPGMGGTQRLPRLIGIQKALTYMLTGKNMYSYQARKTGF
tr|A0A7Y5V3S5|A0A7Y5V3S5_9BACT       WRIATDHPKTVLGLPEVQIGLFPGAGGTQRLPRTIGLQAALDMILTGRNVRAKKALQIGL
tr|A0A7W1SHC0|A0A7W1SHC0_9BACT       WRVATDHPKTVLALPEVQLGLIPGSGGTQRLPRLVGLQRALDMILTGRNIRAKKALGMGL
tr|A0A7Y5PBT7|A0A7Y5PBT7_9BACT       YRIASDHPKTVLALPEVQLGLIPGAGGTQRLPRLIGLQRALDMILSSRNVRARKAYQMGL
tr|A0A7Y5TXL8|A0A7Y5TXL8_9BACT       YRIASDHPKTVLALPEVQLGLIPGAGGTQRLPRLIGLQRALDMILSSRNVRARKAYQMGL
tr|A0A423PQ96|A0A423PQ96_9GAMM       VRIAADTAATTLGLPEVMLGLLPAAGGTQRLPRLVGIQAALDLMLTGRKLRPAQAKACGI
tr|U2G066|U2G066_9GAMM               VRIAAETPATALGLPEVMLGLLPAAGGTQRLPRLVGIATGLDLMLTGRKLRPAQAKKIGL
tr|A0A2E0J1F4|A0A2E0J1F4_9GAMM       VRIAADTPATTLGVPEVMLGLLPAAGGTQRLPRLVGIQSALDLMLTGRKLRPAQAKKLGL
tr|A0A2D4SCM8|A0A2D4SCM8_9GAMM       ARVASDSPKTVLGLPEVMLGLLPAGGGTQRLPNLIGIRAALDLMLTGKQVRPVKAKKLGI
tr|L0WJH1|L0WJH1_9GAMM               YRIAANSGDTVLGLPEVMLGLLPAGGGTQRLPRLVGIASALDMMLTGRQLKARPARKMGL
tr|A0A1H5XF61|A0A1H5XF61_9GAMM       YRIAASSSDTVLGLPEVMLGLLPAGGGTQRMPRLVGIASALDLMLTGRQLKAKPAKKIGL
tr|A0A2E9TS48|A0A2E9TS48_9GAMM       YRIAASSSDTVLGLPEVMLGLLPAGGGTQRMPRQVGIATALDLMLTGRQLKAKPAKKIGL
tr|A0A095UE19|A0A095UE19_9GAMM       YRIAASSSDTVLGLPEVMLGLLPAGGGTQRLPRQVGIATALDLMLTGRQLKAKPAKKIGL
tr|A0A7G2S9A0|A0A7G2S9A0_9GAMM       YRIAASSSDTVLGLPEVMLGLLPAGGGTQRLPRQVGIATALDLMLTGRQLKAKPAKKIGL
tr|B5JU27|B5JU27_9GAMM               YRVASSRSNTALGVPEVMLGLLPAGGGTQRLPRLVGIPAALDLMLTSRQLKPKQALKMGL
tr|A0A1Y0IHP1|A0A1Y0IHP1_9GAMM       YRIATSNSSTVLGLPEVMLGLLPAGGGTQRLPRLVGIAKALDMMLTGRQLKAKQALGMGL
tr|A0A316FZ14|A0A316FZ14_9GAMM       QRVCTDSSKTKLGLPEVQLGVLPGSGGTQRMPRLTGIATALDLMLTGKQLNAKRAQKAGL
tr|A0A498C231|A0A498C231_9GAMM       GRVATDHPATRLGLPEVQLGLLPGSGGTQRLPDRVGLPLALDLMLTGRQLRARQARKRGL
tr|Q0A6T4|Q0A6T4_ALKEH               GRVATDHPATRLGLPEVQLGLLPGSGGTQRLPDRVGLPLALDLMLTGRQLRARQARKRGL
tr|A0A3E0WL10|A0A3E0WL10_9GAMM       GRVATDNPKTSVGLPEVMLGLLPGSGGTQRLPRMIGVPAALDLMLTGKHVRASKALRMKL
tr|A0A3S1BU16|A0A3S1BU16_9GAMM       GRVCTDSPKTALGLPEVMLGVLPGSGGTQRLPRLIGIPAALDLMLTGRHLNARKAQRLGL
tr|A0A7V8QFN9|A0A7V8QFN9_9GAMM       ARVCTDSEKTVLGLPEVQLGLLPGSGGTQRLPRYVGIPAALDMMMTGKHVRPGKARRMGL
tr|A0A1H8PPF9|A0A1H8PPF9_9GAMM       GRVCTDADKTALGVPEVMLGLLPGSGGTQRLPRLVGIQAALGMALTGKQIRPAKAKRMGL
tr|A0A6H0J0V7|A0A6H0J0V7_9GAMM       GRVCTDSNKTALGVPEVMLGLLPGSGGTQRLPRQVGIQAALDMALTGKQIVPAKARRMGL
tr|V5EZX6|V5EZX6_9VIBR               YRVCSDDDITRLGLPEVQLGLLPGSGGTQRLPRLIGLMQSLDLILTGKQVRAKKAKKLGL
tr|A0A6I1QBM2|A0A6I1QBM2_9VIBR       YRVCSDDDITRLGLPEVQLGLLPGSGGTQRLPRLIGLMQSLDLILTGKQVRAKKAKKLGL
tr|A0A511QRL5|A0A511QRL5_9VIBR       YRVCSDDDITRLGLPEVQLGLLPGSGGTQRLPRLIGLMASLDLILTGKQVRAKKAKKLGL
tr|A0A7Y0JZP4|A0A7Y0JZP4_9VIBR       YRVCSDDDITRLGLPEVQLGLLPGSGGTQRLPRLIGLMASLDLILTGKQVRAKKAKKLGL
tr|A0A193KD24|A0A193KD24_9VIBR       YRVCSDDDITRLGLPEVQLGLLPGSGGTQRLPRLIGLMASLDLILTGKQVRAKKAKKLGL
tr|A0A1S1HM72|A0A1S1HM72_PROST       ARVCSNDDKTRLGLPEVQLGLLPGSGGTQRLPKLVGIPSALDMMLTGKQLRAKQAQKIGL
tr|A0A7T8I613|A0A7T8I613_9GAMM       ARVCSNDDKTRLGLPEVQLGLLPGSGGTQRLPKLVGIPSALDMMLTGKQLRAKQAQKIGL
tr|A0A140NIF0|A0A140NIF0_PROSM       VRVCSNDDKTRLGLPEVQLGLLPGSGGTQRLPKLIGIPNALDMMLTGKQLRAKQALKMGL
tr|A0A379GMB7|A0A379GMB7_PROST       VRVCSNDDKTRLGLPEVQLGLLPGSGGTQRLPKLIGIPNALDMMLTGKQLRAKQALKMGL
tr|A0A379H393|A0A379H393_PROST       VRVCSNDDKTRLGLPEVQLGLLPGSGGTQRLPKLIGIPNALDMMLTGKQLRAKQALKMGL
tr|B2Q0L5|B2Q0L5_PROST               VRVCSNDDKTRLGLPEVQLGLLPGSGGTQRLPKLIGIPNALDMMLTGKQLRAKQALKMGL
tr|A0A6I3JUE9|A0A6I3JUE9_9GAMM       VRVCSNDTKTRLGLPEVQLGLLPGSGGTQRLPKLIGIPNALDMMLTGRQLRAKQAQRMGL
tr|A0A7D4P594|A0A7D4P594_YERMW       SRICSLDDKTVLGLPEVQLGLLPGSGGTQRLPRLVGVSKALDMILTGRQVRARQALKMGL
tr|A0A7U7IVK6|A0A7U7IVK6_YEREN       SRICSQDDKTVLGLPEVQLGLLPGSGGTQRLPRLVGVSKALDMILTGRQVRARQALKMGL
tr|A0A447RIF5|A0A447RIF5_YEREN       SRICSQDDKTVLGLPEVQLGLLPGSGGTQRLPRLVGVSKALDMILTGRQVRARQALKMGL
tr|A0A7H4ZLV9|A0A7H4ZLV9_YERP4       SRICSQDDKTVLGLPEVQLGLLPGSGGTQRLPRLVGVSKALDMILTGRQVRARQALKMGL
tr|A0A0H3NX46|A0A0H3NX46_YERE1       SRICSQDDKTVLGLPEVQLGLLPGSGGTQRLPRLVGVSKALDMILTGRQVRARQALKMGL
tr|A0A7T9XUV7|A0A7T9XUV7_YEREN       SRICSQDDKTVLGLPEVQLGLLPGSGGTQRLPRLVGVSKALDMILTGRQVRARQALKMGL
tr|A0A7U7IYV8|A0A7U7IYV8_YEREN       SRICSQDDKTVLGLPEVQLGLLPGSGGTQRLPRLVGVSKALDMILTGRQVRARQALKMGL
tr|A0A0H5G7G2|A0A0H5G7G2_YEREN       SRICSQDDKTVLGLPEVQLGLLPGSGGTQRLPRLVGVSKALDMILTGRQVRARQALKMGL
tr|A0A2A7TDI2|A0A2A7TDI2_YERKR       SRICSQDDKTVLGLPEVQLGLLPGSGGTQRLPRLVGVSKALDMILTGRQVRARQALKMGL
tr|A0A0T9M261|A0A0T9M261_YERKR       SRICSQDDKTVLGLPEVQLGLLPGSGGTQRLPRLVGVSKALDMILTGRQVRARQALKMGL
tr|A0A0A0CMP1|A0A0A0CMP1_PHOLU       WRVCSLDDKTRLGLPEVQLGLLPGSGGTQRLPRLIGVSSALDIILTGKQLRAKQALRLGL
tr|A0A329VG55|A0A329VG55_9GAMM       WRVCSQDDKTRLGLPEVQLGLLPGSGGTQRLPRLIGVSSALDIMLTGKQLRAKQALRLGL
sp|Q7N288|FADJ_PHOLL                 WRVCSQDDKTRLGLPEVQLGLLPGSGGTQRLPRLIGVSSALDIMLTGKQLRAKQALRLGL
tr|A0A6L9JMX5|A0A6L9JMX5_PHOLM       WRVCSQDDKTRLGLPEVQLGLLPGSGGTQRLPRLIGVSSALDIMLTGKQLRAKQALRLGL
tr|A0A7X5HQ29|A0A7X5HQ29_PHOLM       WRVCSQDDKTRLGLPEVQLGLLPGSGGTQRLPRLIGVSSALDIMLTGKQLRAKQALRLGL
tr|A0A1C0U051|A0A1C0U051_9GAMM       WRICSLDDKTRLGLPEVQLGLLPGSGGTQRLPRLIGVSSALDIMLTGRQLSARQALRLGV
tr|A0A7X5QF62|A0A7X5QF62_9GAMM       WRICSLDDKTRLGLPEVQLGLLPGSGGTQRLPRLIGVSSALDIMLTGRQLRARQALRLGV
```

```
tr|A0A2D0KWM9|A0A2D0KWM9_9GAMM  ARICSLDDKTRLGLPEVQLGLLPGSGGTQRLPRLIGVSSALDMILTGRQLKAKQAKRLGV
tr|A0A2D0LAI1|A0A2D0LAI1_9GAMM  ARICSLDDKTRLGLPEVQLGLLPGSGGTQRLPRLIGVSSALDMILTGRQLKAKQAKRLGV
tr|A0A2D0IXC8|A0A2D0IXC8_XENBU  ARVCSLDDKTRLGLPEVQLGLLPGSGGTQRLPRLIGVSSALDMMLTGRQLKANQAKRLGV
tr|W1JAM5|W1JAM5_9GAMM          ARVCSLDDKTRLGLPEVQLGLLPGSGGTQRLPRLIGVSSALDMMLTGRQLKANQAKRLGV
tr|A0A3D9UED3|A0A3D9UED3_9GAMM  ARVCSLDDKTRLGLPEVQLGLLPGSGGTQRLPRLIGVSSALDMMLTGRQLKANQAKRLGV
tr|A0A1I3JCA9|A0A1I3JCA9_9GAMM  ARVCSLDEKTRLGLPEVQLGLLPGSGGTQRLPRLIGVSAALDMILTGRQLKAKQAQRLGV
tr|A0A068QUL9|A0A068QUL9_9GAMM  ARICSLDEKTRLGLPEVQLGLLPGSGGTQRLPRLIGVSAALDIILTGRQLKAKQAQRLGV
tr|A0A0M0TCH6|A0A0M0TCH6_9GAMM  ARICSLDEKTRLGLPEVQLGLLPGSGGTQRLPRLIGVSAALDIILTGRQLKAKQAQRLGV
tr|A0A1Q5U854|A0A1Q5U854_9GAMM  ARICSLDEKTRLGLPEVQLGLLPGSGGTQRLPRLIGVSTALDIILTGRQLKAKQAQRLGV
tr|A0A2D0ISP1|A0A2D0ISP1_9GAMM  ARICSLDEKTRLGLPEVQLGLLPGSGGTQRLPRLIGVSAALDIILTGRQLKVKQAQRLGV
tr|A0A1Q5TUI8|A0A1Q5TUI8_9GAMM  ARICSLDEKTRLGLPEVQLGLLPGSGGTQRLPRLIGVSAALDIILTGRQLKAKQAQRLGV
tr|A0A2D0KJI7|A0A2D0KJI7_9GAMM  ARICSLDEKTRLGLPEVQLGLLPGSGGTQRLPRLIGVSAALDIILTGRQLKAKQAQRLGV
tr|A0A1Y2SB97|A0A1Y2SB97_9GAMM  VRICSLDEKTRLGLPEVQLGLLPGSGGTQRLPRLIGVSAALDIILTGRQLRAKQAQRLGV
tr|A0A1I5DWI7|A0A1I5DWI7_9GAMM  ARICSLDEKTRLGLPEVQLGLLPGSGGTQRLPRLIGVSAALDIILTGRQLKAKQAQRLGV
tr|A0A1I7GWY6|A0A1I7GWY6_9GAMM  ARICSLDDKTRLGLPEVQLGLLPGSGGTQRLPRLVGVSAALDMILTGRQLKARQAKRLGV
tr|D3VKY8|D3VKY8_XENNA          ARICSLDERTRLGLPEVQLGLLPGSGGTQRLPRLIGVSAALDMILTGRQLKAKQAQRLGV
tr|A0A2G0Q6Q9|A0A2G0Q6Q9_9GAMM  ARICSLDEKTRLGLPEVQLGLLPGSGGTQRLPRLIGVSAALDMILTGRQLKAKQAQRLGV
tr|A0A2D0JU24|A0A2D0JU24_9GAMM  ARICSLDDKTRLGLPEVQLGLLPGSGGTQRLPRLIGVSAALDMILTGRQLKAKQAQRLGV
tr|A0A0J5FN38|A0A0J5FN38_9GAMM  ARICSLDDKTRLGLPEVQLGLLPGSGGTQRLPRLIGVSAALDMILTGRQLKAKQAQRLGV
tr|A0A432XLD4|A0A432XLD4_9GAMM  YRVATDSGKTVIGLPEVQLGLLPGSGGTQRLPRLIGVQKALPLILTGKQLRAKQAKKLGI
tr|A0A2D8HU84|A0A2D8HU84_9GAMM  VRICTDNSKTALGLPEVKLGLLPGSGGTQRLPELVGVQQGLTMILTGKELRAKQAKKAGL
tr|A0A656X1Q2|A0A656X1Q2_9GAMM  VRICTDNSKTALGLPEVKLGLLPGSGGTQRLPELVGVQQGLTMILTGKELRAKQAKKAGL
tr|A0A4Q1QH79|A0A4Q1QH79_9GAMM  VRVCTDNSKTVLGLPEVQLGLLPGSGGTQRLPRLVGIQQGLTMMLTGKQLRAKQAKKAGL
tr|A0A1G7LQE6|A0A1G7LQE6_9GAMM  VRVCTDNSKTVLGLPEVQLGLLPGSGGTQRLPRLVGIQQGLTMMLTGKQLRAKQAKKAGL
tr|A0A432YVG7|A0A432YVG7_9GAMM  VRVCTDNSKTVLGLPEVQLGLLPGSGGTQRLPRLVGIQQGLTMMLTGKQLRAKQAKKAGL
tr|A0A1J5N0P5|A0A1J5N0P5_9GAMM  ARVCTNDPKTVLGLPEVQLGLLPGSGGTQRLPALVGIAKALDMMLTGKQLRAKDAKKVGL
tr|A0A0D8D548|A0A0D8D548_9GAMM  ARVCTDSPKTALGLPEVQLGLLPGSGGTQRLPKLVGIQKALDMMLTGKQLRAKQALKAGL
tr|A0A3E0TNM2|A0A3E0TNM2_9GAMM  ARICSDSPKTALGVPEVQLGLLPGGGGTQRLPKLVGIQKALDMMLTGKQLRAKQALKIGL
tr|A0A3E0UD67|A0A3E0UD67_9GAMM  ARICSDSPKTALGVPEVQLGLLPGGGGTQRLPKLVGIQKSLDMMLTGKQLRAKQALKAGL
tr|A0A3E0U0P4|A0A3E0U0P4_9GAMM  ARICSDSPKTALGVPEVQLGLLPGGGGTQRLPKLVGIQKSLDMMLTGKQLRAKQALKAGL
tr|A0A0M2V8B1|A0A0M2V8B1_9GAMM  ARVATDSVKTVLGLPEVQLGLLPGSGGTQRLPRLVGVQKALDMMLTGKQLRAVPAKKAGL
tr|A0A285ITY5|A0A285ITY5_9GAMM  ARIATDSAKTILGLPEVQLGLLPGSGGTQRLPRLVGVQKALDMMLTGKQLRASQAKKAGL
tr|A0A486XTB9|A0A486XTB9_9GAMM  ARVATTDNKTVLGLPEVQLGLLPGSGGTQRLPKLVGLQKALDMILTGKQLRAAQAKKAGL
tr|I1E1G1|I1E1G1_9GAMM          ARVATSDSKTVLGLPEVQLGLLPGSGGTQRLPKLVGLQKALDMILTGKQLRAAQAKKAGL
tr|A0A1H6KJ68|A0A1H6KJ68_9GAMM  ARVASNDNKTVLGLPEVQLGLLPGSGGTQRLPRLVGLQKALDMILTGKQLRAAQAKKAGL
tr|A0A0X3Y764|A0A0X3Y764_9GAMM  ARVASNDSKTVLGLPEVQLGLLPGSGGTQRLPRLVGLQKALDMILTGKQLRAAQAKKAGL
tr|A0A2N1YEV4|A0A2N1YEV4_9GAMM  ARVASNDSKTVLGLPEVQLGLLPGSGGTQRLPRLVGLQKALDMILTGKQLRAAQAKKAGL
tr|A0A3P3QCM3|A0A3P3QCM3_9GAMM  ARVATTDNKTVLGLPEVQLGLLPGSGGTQRLPRLVGIQKALDMILTGKQLRAAQAKKIGL
tr|F7NT72|F7NT72_9GAMM          ARVATTDNKTVLGLPEVQLGLLPGSGGTQRLPRLVGIQKALDMILTGKQLRAAQAKKIGL
tr|A0A3S2TW63|A0A3S2TW63_9GAMM  ARIATTDNKTVLGLPEVQLGLLPGSGGTQRLPRLVGIQKALDMILTGKQLRAAQAKKIGL
tr|A0A3D5BGW7|A0A3D5BGW7_9GAMM  GRVATDDGKTVLGLPEVQLGLLPGSGGTQRLPKLVGLQKALDMMLTGKQLRAVQAKKAGL
tr|A0A5C7TI17|A0A5C7TI17_9GAMM  GRVATDDAKTVLGLPEVQLGLLPGSGGTQRLPKLVGLQKALDMMLTGKQLRAVQAKKTGL
tr|A0A0U4W982|A0A0U4W982_9GAMM  GRVATDDNKTVLGLPEVQLGLLPGSGGTQRLPKLVGLQKALDMMLTGKQLRAVQAKKAGL
tr|A0A2I0FCM2|A0A2I0FCM2_9GAMM  IRVCTESTKTVLGVPEVMLGLLPGSGGTQRLPRLIGVAKSLDLMLTGKQVRGKQALKMGL
tr|A0A2G2IVL1|A0A2G2IVL1_9GAMM  IRVCTESTKTVLGVPEVMLGLLPGSGGTQRLPRLIGVAKSLDLMLTGKQVRGKQALKMGL
tr|A6FI21|A6FI21_9GAMM          IRVCTESTKTVLGVPEVMLGLLPGSGGTQRLPRLIGVAKSLDLMLTGKQVRGKQALKMGL
tr|A0A4U1BNR2|A0A4U1BNR2_9GAMM  YRVISNSPKTVLGLPEVQLGILPGGGGTQRLPKLVGIAKSLDLMLTGKQLRPKQAVKLGV
tr|E1SQ60|E1SQ60_FERBD          YRICSDSAKTVLGVPEVQLGLLPGGGGTQRLPRLVGVARSLDMMLSGRQLRAKQAKKFGL
tr|A0A4Y6IZ09|A0A4Y6IZ09_9GAMM  QRVCSDSNKTMLGLPEVQLGLLPGGGGTQRLPRLVGIAKALDMMLTGKQLRPKQALKLGL
tr|A0A0C3QSZ7|A0A0C3QSZ7_9GAMM  QRVCTDSKKTMLGLPEVQLGLLPGGGGTQRLPRLVGIAKSLDMMLTGKQLRAKQALKLGL
tr|A0A6L7HXW0|A0A6L7HXW0_9GAMM  QRVCTDSKKTMLGLPEVQLGLLPGGGGTQRLPRLVGIAKSLDMMLTGKQLRAKQALKMGL
sp|A3QFP3|FADJ_SHELP            QRVCTDSNKTMLGLPEVQLGLLPGGGGTQRLPRLVGIAKSLDMMLTGKQLRAKQALKMGL
tr|A0A1E5IXH7|A0A1E5IXH7_SHECO  MRVCTDSAKTMMGLPEVQLGLLPGGGGTQRLPRLVGITTALDMMLTGKQLRPKQALKMGL
tr|A0A411PKQ0|A0A411PKQ0_9GAMM  LRVCTDDKKTVLGVPEVQLGLLPGGGGTQRLPRLVGVTTALDMMLTGKQIRPKKALKMGL
tr|A0A6G9QKM3|A0A6G9QKM3_9GAMM  LRVCSDDKKTVLGVPEVQLGLLPGGGGTQRLPRLVGVTTALDMMLTGKQIRPKQALKMGL
tr|A0A6P1UL63|A0A6P1UL63_9GAMM  LRVCSDDKKTMLGVPEVQLGLLPGGGGTQRLPRLVGIATALDMMLTGKQIRPKQALKMGL
tr|A0A2N1ERI9|A0A2N1ERI9_9GAMM  LRVCSDDKKTMLGVPEVQLGLLPGGGGTQRLPRLVGIATALDMMLTGKQIRPKQALKMGL
tr|A0A7W4FU55|A0A7W4FU55_9GAMM  LRVCSDDKKTMLGVPEVQLGLLPGGGGTQRLPRLVGIATALDMMLTGKQIRPKQALKMGL
sp|Q8ECP7|FADJ_SHEON            QRVCSDDGKTMLGVPEVQLGLLPGGGGTQRLPRLVGITTALDMMLTGKQIRPKQALKMGL
tr|A0A501XZY8|A0A501XZY8_9GAMM  QRVCSDDGKTMLGVPEVQLGLLPGGGGTQRLPRLVGITTALDMMLTGKQIRPKQALKMGL
tr|A0A2W5DCZ0|A0A2W5DCZ0_SHEOE  QRVCSDDGKTMLGVPEVQLGLLPGGGGTQRLPRLVGITTALDMMLTGKQIRPKQALKMGL
tr|A0A1E3V3C8|A0A1E3V3C8_9GAMM  QRVCSDDGKTMLGVPEVQLGLLPGGGGTQRLPRLVGITTALDMMLTGKQIRPKQALKMGL
tr|A0A1Z4AI20|A0A1Z4AI20_9GAMM  QRVCSDDGKTMLGVPEVQLGLLPGGGGTQRLPRLVGITTALDMMLTGKQIRPKQALKMGL
tr|A0A7X9LJL9|A0A7X9LJL9_9GAMM  QRVCSDDGKTMLGVPEVQLGLLPGGGGTQRLPRLVGITTALDMMLTGKQIRPKQALKMGL
tr|A0A073KMY5|A0A073KMY5_9GAMM  QRVCSDDGKTMLGVPEVQLGLLPGGGGTQRLPRLVGITTALDMMLTGKQIRPKQALKMGL
sp|A0KV76|FADJ_SHESA            QRVCSDDGKTMLGVPEVQLGLLPGGGGTQRLPRLVGITTALDMMLTGKQIRPKQALKMGL
tr|A0A220UTH7|A0A220UTH7_9GAMM  QRVCSDDGKTMLGVPEVQLGLLPGGGGTQRLPRLVGITTALDMMLTGKQIRPKQALKMGL
tr|A0A5B8R6W1|A0A5B8R6W1_9GAMM  QRVCSDDGKTMLGVPEVQLGLLPGGGGTQRLPRLVGITTALDMMLTGKQIRPKQALKMGL
tr|V1DAI4|V1DAI4_9GAMM          LRVCSDDGKTMLGVPEVQLGLLPGGGGTQRLPRLVGITTALDMMLTGKQIRPKQALKMGL
tr|A0A448CPQ4|A0A448CPQ4_SHEPU  QRVCSDDGKTMLGVPEVQLGLLPGGGGTQRLPRLVGITTALDMMLTGKQIRPKQALKMGL
tr|A0A252ERQ3|A0A252ERQ3_SHEPU  QRVCSDDGKTMLGVPEVQLGLLPGGGGTQRLPRLVGITTALDMMLTGKQIRPKQALKMGL
sp|Q0HKD1|FADJ_SHESM            QRVCSDDGKTMLGVPEVQLGLLPGGGGTQRLPRLVGITTALDMMLTGKQIRPKQALKMGL
sp|Q0HWN3|FADJ_SHESR            QRVCSDDGKTMLGVPEVQLGLLPGGGGTQRLPRLVGITTALDMMLTGKQIRPKQALKMGL
tr|F7RQE3|F7RQE3_9GAMM          QRVCSDDGKTMLGVPEVQLGLLPGGGGTQRLPRLVGITTALDMMLTGKQIRPKQALKMGL
tr|B8EE98|B8EE98_SHEB2          QRVCSDDGKTMLGVPEVQLGLLPGGGGTQRLPRLVGITTALDMMLTGKQIRPKQALKMGL
sp|A6WQ25|FADJ_SHEB8            QRVCSDDGKTMLGVPEVQLGLLPGGGGTQRLPRLVGITTALDMMLTGKKIRPKQALKMGL
tr|A0A448EK41|A0A448EK41_9GAMM  QRVCSDDGKTMLGVPEVQLGLLPGGGGTQRLPRLVGITTALDMMLTGKQIRPKQALKMGL
tr|A0A553JHX1|A0A553JHX1_SHEHA  QRVCSDNNKTVMGVPEVQLGLLPGGGGTQRLPRLVGITTALDMMLTGKQLRPKQALKMGL
tr|B8CPY6|B8CPY6_SHEPW          QRVCSLNSKTMMGVPEVQLGLLPGGGGTQRLPRLVGITTALDMMLTGKQIRPKQAIKMGL
tr|A0A431WFC4|A0A431WFC4_9GAMM  QRVCSLNSKTMMGVPEVQLGLLPGGGGTQRLPRLVGITTALDMMLTGKQLRPKQAVKMGL
tr|A8FTR7|A8FTR7_SHESH          QRVCSLNSKTMMGVPEVQLGLLPGGGGTQRLPRLVGITTALDMMLTGKQLRPKQALKMGL
tr|A0A431WNL0|A0A431WNL0_9GAMM  QRVCSLNSKTMMGVPEVQLGLLPGGGGTQRLPRLLGITTALDMMLTGKQLRPKQALKMGL
tr|A0A550AEC5|A0A550AEC5_9GAMM  LRVCSLNTKTMMGVPEVQLGLLPGGGGTQRLPRLVGITTALDMMLTGKQIRPKQALKMGL
tr|A0A7L4WW90|A0A7L4WW90_9GAMM  LRVCSLNTKTMMGVPEVQLGLLPGGGGTQRLPRLVGITTALDMMLTGKQIRPKQALKMGL
tr|A9DDU3|A9DDU3_9GAMM          QRVCSQNSKTTMGVPEVQLGLLPGGGGTQRLPRLVGITTALDMMLTGKQIRPKQALKMGL
tr|A0A330M2B9|A0A330M2B9_9GAMM  QRVCSQNSKTMMGVPEVQLGLLPGGGGTQRLPRLVGITTALDMMLTGKQIRPKQALKMGL
tr|A0A5N8UFC7|A0A5N8UFC7_9GAMM  QRVCSQNGKTMMGVPEVQLGLLPGGGGTQRLPRLVGITTALDMMLTGKQIRPKQALKMGL
tr|A0A1S6HN57|A0A1S6HN57_9GAMM  QRVCSQNSKTMMGVPEVQLGLLPGGGGTQRLPRLVGITTALDMMLTGKQIRPKQALKMGL
tr|D4ZMH7|D4ZMH7_SHEVD          LRVCSQNGKTMMGVPEVQLGLLPGGGGTQRLPRLVGITTALDMMLTGKQIRPKQALKMGL
tr|A0A3L8Q213|A0A3L8Q213_9GAMM  ARICTDDAKTMLGLPEVMLGLLPGGGGTQRLPKLVGITTALDMMLTGKQLRGKQALKAGL
tr|A0A3A6U4N9|A0A3A6U4N9_9GAMM  QRICSDDTKTALGLPEVQLGLLPGGGGTQRLPRLVGIASALDMMLTGKQLRPKQAKKLGI
tr|A0A4Q5MA37|A0A4Q5MA37_9GAMM  MRICSDSPKTMLGLPEVMLGLLPGGGGTQRLPRLVGIATALDMMLTGKQLRPKQALKNGL
tr|A0A1L6LSX5|A0A1L6LSX5_9DELT  GRVLSDDKKTALGLPEVQLGLLPGLNGLERLAALVGLQVALDYGLTGKNMRAAKAKSLGV
tr|A0A2W4L9B9|A0A2W4L9B9_9PROT  YRLASLARHTKLGLPETQLGLIPGAGGTQRLPRLIGIARALDLILTGRQVDARRALRMGL
tr|A0A2W4M4E6|A0A2W4M4E6_9PROT  GRVATNDPRTRLGLPEVQLGLLPGLGGTQRLPRLVGLQASLDLMLTGRQLDARRARKIGL
tr|A0A6I2GRX9|A0A6I2GRX9_9DELT  YRIATDSPKTQLGLPEVQLGLLPGAGGTQRLPALTGAQAALDLILAGKTVKAQKAKKLGI
tr|A0A0H4WMK2|A0A0H4WMK2_9DELT  YRIATDSPKTSLGLPEVQLGLIPGAGGTQRLPALIGVQAALDLILTGKSLKPAKAKKLGV
tr|F8CJ36|F8CJ36_MYXFH          YRVATDSPKTSLGLPEVQLGLIPGAGGTQRLPALIGVQAALDLILTGKSLKPGKAKKLGV
tr|A0A250K0F1|A0A250K0F1_9DELT  YRVATDSPKTSLGLPEVQLGLIPGAGGTQRLPALIGVQAALDLILTGKSLKPGKAKKLGV
tr|A0A7Y6WFZ2|A0A7Y6WFZ2_9DELT  YRIATDSPKTSLGLPEVQLGLIPGAGGTQRLPALIGVQAALDLILTGKSLKPAKAKKLGV
tr|A0A7Y7C660|A0A7Y7C660_9DELT  YRIATDSPKTSLGLPEVQLGLIPGAGGTQRLPALIGVQAALDLILTGKSLKPAKAKKLGV
tr|A0A7Y4JFH2|A0A7Y4JFH2_MYXXA  YRIATDSPKTSLGLPEVQLGLIPGAGGTQRLPALIGVQAALDLILTGKSLKPAKAKKLGV
tr|A0A4Y6CZQ9|A0A4Y6CZQ9_MYXXA  YRIATDSPKTSLGLPEVQLGLIPGAGGTQRLPALIGVQAALDLILTGKSLKPAKAKKLGV
```

```
tr|A0A7Y4IKV5|A0A7Y4IKV5_MYXXA  YRIATDSPKTSLGLPEVQLGLIPGAGGTQRLPALIGVQAALDLILTGKSLKPAKAKKLGV
tr|A0A511HHB0|A0A511HHB0_9DELT  YRIATDSPKTSLGLPEVQLGLIPGAGGTQRLPALIGVQAALDLILTGKSLKPAKAKKLGV
tr|A0A4Y6CKY7|A0A4Y6CKY7_MYXXA  YRIATDSPKTSLGLPEVQLGLIPGAGGTQRLPALIGVQAALDLILTGKSLKPAKAKKLGV
tr|Q1D1F2|Q1D1F2_MYXXD          YRIATDSPKTSLGLPEVQLGLIPGAGGTQRLPALIGVQAALDLILTGKSLKPAKAKKLGV
tr|A0A7Y4MA14|A0A7Y4MA14_MYXXA  YRIATDSPKTSLGLPEVQLGLIPGAGGTQRLPALIGVQAALDLILTGKSLKPAKAKKLGV
tr|A0A7T8Y4N9|A0A7T8Y4N9_MYXXA  YRIATDSPKTSLGLPEVQLGLIPGAGGTQRLPALIGVQAALDLILTGKSLKPAKAKKLGV
tr|L7UE67|L7UE67_MYXSD          YRIATDSPKTTLGLPEVQLGLLPGAGGTQRLPALIGAQTALDLILTGKSVKPSKARKLGI
tr|A0A511T9X1|A0A511T9X1_MYXFU  YRIATDSPKSTLGLPEVQLGLLPGAGGTQRLPALIGAQAALDLILTGKNVKPAKARKLGI
tr|A0A7Y7C9C4|A0A7Y7C9C4_9DELT  YRIATDSPKTTLGLPEVQLGLIPGAGGTQRLPALIGVQAALDLILTGKNVKPSKAKKLGI
tr|A0A540X7W8|A0A540X7W8_9DELT  YRIATDSPRTTLGLPEVQLGLIPGAGGTQRLPALIGVQAALDLILTGKSVKPSKAKKLGI
tr|A0A3A5FK19|A0A3A5FK19_9DELT  YRIATDSPKTTLGLPEVQLGLIPGAGGTQRLPALIGAQQALDLILTGKNVKPAKAKKLGL
tr|A0A3A8JQL9|A0A3A8JQL9_9DELT  YRIVTDSPKSVVGLPETQLGLIPGAGGTQRLPALIGAQAALDLILTGKNVKPSKAKKLGI
tr|A0A7Y4NFA4|A0A7Y4NFA4_9DELT  YRIVTDSPKSVVGLPETQLGLIPGAGGTQRLPALIGAQAALDLILTGKNVKPSKAKKLGI
tr|A0A3A8HDQ7|A0A3A8HDQ7_9DELT  YRIVTDSPKSVVGLPETQLGLIPGAGGTQRLPALIGAQAALDLILTGKNVKPSKAKKLGI
tr|A0A3A8GR90|A0A3A8GR90_9DELT  YRIVTDSPKSVVGLPETQLGLIPGAGGTQRLPALIGAQAALDLILTGKNVKPSKAKKLGI
tr|A0A3A8SBD7|A0A3A8SBD7_9DELT  YRIVTDSPKSVVGLPETQLGLIPGAGGTQRLPALIGAEAALDLILTGKNVKPSKAKKLGV
tr|A0A3A8T0I8|A0A3A8T0I8_9DELT  YRIVTDSPKSVVGLPETQLGLIPGAGGTQRLPALIGAEAALDLILTGKNVKPSKAKKLGL
tr|A0A7X5BU07|A0A7X5BU07_9DELT  YRIVTDSPKSVVGLPETQLGLIPGAGGTQRLPALIGAEAALDLILTGKNVKPSKAKKLGV
tr|A0A7Y1RVL2|A0A7Y1RVL2_9DELT  YRIVTDSPKSVVGLPETQLGLIPGAGGTQRLPALIGAEAALDLILTGKNVKPSKAKKLGV
tr|A0A3A8THN2|A0A3A8THN2_9DELT  YRIVTDSPKSVVGLPETQLGLIPGAGGTQRLPALIGAEAALDLILTGKNVKPSKAKKLGV
tr|A0A7Y1RX76|A0A7Y1RX76_9DELT  YRIVTDSPKSVVGLPETQLGLIPGAGGTQRLPALIGAEAALDLILTGKNVKPSKAKKLGV
tr|A0A3A8RA58|A0A3A8RA58_9DELT  YRIVTDSPKSVVGLPETQLGLIPGAGGTQRLPALIGAQAALDLILTGKNVKPSKAKKLGI
tr|A0A410RPB6|A0A410RPB6_CORCK  YRIVTDSPKSVVGLPETQLGLIPGAGGTQRLPALIGAEAALDLILTGKNVKPSKAKKLGI
tr|A0A7Y4J474|A0A7Y4J474_CORCK  YRIVTDSPKSVVGLPETQLGLIPGAGGTQRLPALIGAEAALDLILTGKNVKPSKAKKLGI
tr|A0A3A8I9Z6|A0A3A8I9Z6_9DELT  YRIVTDSPKSVVGLPETQLGLIPGAGGTQRLPALIGAEAALDLILTGKNVKPSKAKKLGI
tr|H8MKE9|H8MKE9_CORCM          YRIVTDSPKSVVGLPETQLGLIPGAGGTQRLPALIGAEAALDLILTGKNVKPSKAKKLGI
tr|A0A3A8H102|A0A3A8H102_9DELT  YRIVTDSPKSVVGLPETQLGLIPGAGGTQRLPALIGAEAALDLILTGKNVKPSKAKKLGI
tr|A0A554FW33|A0A554FW33_9DELT  YRIATDSPKTTLGLPETQLGLIPGAGGTQRLPALIGAQAALDLILTGKSVKPSKAKKLGI
tr|A0A3A8NPC0|A0A3A8NPC0_9DELT  YRIATDSPKTSLGLPETQLGLIPGAGGTQRLPALIGAQAALDLILTGKNVKASKAKKLGV
tr|A0A3A8JUV3|A0A3A8JUV3_9DELT  YRIATDSPKTSLGLPETQLGLIPGAGGTQRLPALIGAQAALDLILTGKNVKASKAKKLGI
tr|A0A3A8NEX6|A0A3A8NEX6_9DELT  YRIATDSPKTSLGLPETQLGLIPGAGGTQRLPALIGAQAALDLILTGKNVKASKAKKLGV
tr|A0A3A8JEU8|A0A3A8JEU8_9DELT  YRIATDSPKTSLGLPETQLGLIPGAGGTQRLPALIGAQAALDLILTGKNVKASKAKKLGI
tr|A0A3A8LI76|A0A3A8LI76_9DELT  YRIATDSPKTSLGLPETQLGLIPGAGGTQRLPELIGAQAALDLILTGKNVKASKAKKLGI
tr|A0A085WXN8|A0A085WXN8_9DELT  YRIATDSPKTQLGLPETQLGLLPGAGGTQRLPALIGIQAALDLILAGKTVKPSKAKRLGL
tr|A0A2T4V0M5|A0A2T4V0M5_9DELT  YRIATDSPKTTLGLPETQLGLIPGAGGTQRLSALIGVQAALDLILTGKNVKPSKAKKLGL
tr|A0A0G2ZSW5|A0A0G2ZSW5_9DELT  YRIATDSPKTTLGLPEVQLGLIPGAGGTQRLPALIGVQAALDLILTGKNVKPSKAKKLGL
tr|A0A3M2DKY0|A0A3M2DKY0_9DELT  ARVASDSRKTQLGQPEVKLGLLPGAGGTQRLPRLVGVQAALDLMLTGKSLRAGKARSLGL
tr|A0A661NQ58|A0A661NQ58_9DELT  ARVASDHRKTKLGLPEVQLGLLPGAGGTQRLPRLIGVQKALDLMLTGKQIDSKRAKRMGL
tr|A0A520YD99|A0A520YD99_9DELT  ARVVTDSKKTQLGLPEAQLGLLPGGGGTQRLPRQIGVQAALDLMLTGKNVNAKKALKLGL
tr|A0A7Y3BRE4|A0A7Y3BRE4_9DELT  ARVVTDSKKTQLGLPEAQLGLLPGGGGTQRLPRQIGVQAALDLMLTGKNVNAKKALKLGL
tr|A0A2D9TF90|A0A2D9TF90_9DELT  GRVCTERAK..LGLPESQIGLLPGAGGTQRLPRLIGLQDALDLMLTGKQVDARKAKKLGL
tr|A0A2E0TP32|A0A2E0TP32_9DELT  GRVASSSRKTKLGLPESQLGLLPGAGGTQRCPELIGVQAALDLMLTGKQVDARKAKKLGL
tr|A0A2E4Y3V1|A0A2E4Y3V1_9PROT  YRLATDSSKTILGLPEVMLGLLPGGGGTQRLPKLIGIQKALDMMLTGKNIKPAKAKKMGL
tr|A0A2E6VRH4|A0A2E6VRH4_9DELT  YRIATQHAKTVLALPEVMLGLLPGAGGTQRLPRLIGIQAALDMMLTGRNIRPAKAKKLGL
tr|A0A1F9FB59|A0A1F9FB59_9DELT  YRIATDHPKTVLSLPEVMLGLLPGGGGTQRLPALVGIQAALDMMLTGKNVRAKKAKRMGL
```

```
ECHA_HUMAN
                                    220       230       240       250
ECHA_HUMAN                          VDQLVEPLGPGLKPPEERTIEYLEEVAITFAKGL...........................
tr|A0A4R6XLA8|A0A4R6XLA8_9GAMM      VDSLVHKEG.............LYQAALKVASNPI..........................
tr|A0A6A0IJT2|A0A6A0IJT2_9BACT      VDALTSPGG.............LVETAAKAALGL..........................A
tr|A0A7V3QZU7|A0A7V3QZU7_9BACT      VDALTSPGG.............IEETAIRAALGL..........................L
tr|A0A7V4SYJ6|A0A7V4SYJ6_9BACT      VSALTTPGG.............LVETAALAARRL..........................A
tr|A0A2A8D2N6|A0A2A8D2N6_9BACT      VDATIHPPG.............LHRAAVEAAKAL..........................A
tr|A0A2N1TGP0|A0A2N1TGP0_9SPIR      IDEIVSPYG.............MEDIAIAKAKDL..........................A
tr|A0A1G3QGW4|A0A1G3QGW4_9SPIR      IDEIVVPYG.............LAEIGVTRALEL..........................A
tr|A0A1G3QTY3|A0A1G3QTY3_9SPIR      IDEVVVPYG.............LREIGVKKATEL..........................A
tr|A0A7X9L359|A0A7X9L359_9DELT      VDEVVMFHG.............MKDIAVKKALDL..........................S
tr|A0A522CJY0|A0A522CJY0_9SPIR      VDKVVIPYG.............MREVAITIALDL..........................A
tr|A0A2N1RRJ3|A0A2N1RRJ3_9SPIR      VDELVPLYG.............LKETALKITAGL...........................
tr|A0A2N2KFN7|A0A2N2KFN7_9DELT      VDELIAPYG.............AKETAVKKALEL.........................AR
tr|A0A1V6AXX9|A0A1V6AXX9_9DELT      VDELIPPYG.............IKETAAKKALEL.........................AG
tr|A0A2N2HZL0|A0A2N2HZL0_9DELT      VDELIPPYG.............AKETAVKKALEL...........................
tr|A0A5E8ARP3|A0A5E8ARP3_9BACT      VDELVYQPK.............LLDAAVKFARKL..........................A
tr|A0A662A7B1|A0A662A7B1_9BACT      VDMIVDRPK.............LLTAAKKHALKI..........................A
tr|D7CV16|D7CV16_TRURR              VDALSHPQG.............LVAAAKAAVRGLVAGEVTAR..................K
tr|A0A3C2AKX4|A0A3C2AKX4_9FLAO      VDRIATKEA.............LLDAAIVLANQI..........................A
tr|A0A3B8ZLN3|A0A3B8ZLN3_9PLAN      VDRLASPPA.............LLNAALFLANEL..........................V
tr|A0A345UGV8|A0A345UGV8_9BACT      VHELTHRHG.............LRQAAHYRAEKL......MK...................
tr|A0A6C1P6A2|A0A6C1P6A2_9BACT      LDEMTHKYA.............LRTAALNVAKKL..........................A
tr|A0A3M1X9W8|A0A3M1X9W8_9BACT      VDELFHPHG.............VVEAALAAVRDL..........................N
tr|A0A354C796|A0A354C796_9DELT      VDDLIHPYG.............LLPAAKKAALTL..........................A
tr|A0A3M1NUM8|A0A3M1NUM8_9BACT      VDALIHPYG.............LLNAARQMALEL..........................V
tr|A0A3M2KRC8|A0A3M2KRC8_9BACT      ADYVIHPYG.............LLSAAKQAALEL...........................
tr|A0A5S9IHR0|A0A5S9IHR0_9BACT      ADEIIHPHG.............LLQAAKNVALKL......TK...................
tr|A0A6M1ST81|A0A6M1ST81_9BACT      VDELTHKDA.............VLTAAKKAVEKI..........................N
tr|A0A521AAE5|A0A521AAE5_9BACT      VDELTHKDA.............ILKAAKKAVRTL..........................R
tr|A0A1M4ZMD6|A0A1M4ZMD6_9BACT      VDELTHKNA.............ILKAAKKAVHRL..........................G
tr|A0A2A2GCS2|A0A2A2GCS2_9BACT      VDELTHRDA.............VLTAAKKAVGKI..........................N
tr|A0A5D3YIH7|A0A5D3YIH7_9BACT      VDELTHKDA.............VLTAAKAAVKKI..........................N
tr|A0A6M1T9G0|A0A6M1T9G0_9BACT      VDELTHRDA.............VLTAAKKAVAKI..........................N
tr|A0A6A8Q1Y8|A0A6A8Q1Y8_9BACT      VDEVTHKDA.............IEKAAIKGVRKI..........................A
tr|A0A521BVJ3|A0A521BVJ3_9BACT      VDEVVHKDA.............LETAAIKAVHKI......TE...................
tr|A0A359E0Y8|A0A359E0Y8_9BACT      VDEVTHKDA.............IEKAAIKGVHKI..........................V
tr|A0A2D8CIM6|A0A2D8CIM6_9BACT      VDEVTHKDA.............IEKAAIKGVHKI..........................V
tr|A0A2D9FWF5|A0A2D9FWF5_9BACT      VDEVTHKDA.............IEKAAIKGVHKI..........................A
tr|A0A1B6YB70|A0A1B6YB70_9BACT      VDELVHKDA.............LEEAAIKAVHSV..........................S
tr|A0A2D4ZTC0|A0A2D4ZTC0_9BACT      ADEMVHKDA.............LKGAAIKAVHKL..........................S
tr|A0A3D4UVW7|A0A3D4UVW7_9BACT      ADEMVHKDA.............LKGAAIKAVHKL..........................S
tr|A0A3F3I3M2|A0A3F3I3M2_9BACT      ADEMVHKDA.............LKGAAIKAVHKL..........................S
tr|A0A3D1G7L9|A0A3D1G7L9_9BACT      VDELVHKDA.............LEEAAIKAVLKI.........................AD
tr|A0A3M8G1Y8|A0A3M8G1Y8_9BACT      VDELTHKDA.............LEEAAIKAVHKI......SE..................A
tr|A0A5Q4F391|A0A5Q4F391_9BACT      ADEVVHRHG.............LKEAGIRAAKKL..........................A
tr|A0A6I7NPV0|A0A6I7NPV0_9BACT      ANELVHRYA.............LTHAGIRAVKKL......KG...................
tr|A0A651G1J2|A0A651G1J2_9BACT      ANELVHKHA.............LKSAAIKAATKI......AK...................
tr|A0A371QRK5|A0A371QRK5_9BACT      ANEVVHRHA.............LKDAGISVVNKR..........................T
tr|A0A2N0VGJ5|A0A2N0VGJ5_9BACT      ANEVVHKHA.............LVKAGIKAVQKL..........................S
tr|A0A316TYJ0|A0A316TYJ0_9BACT      ADEVIHQHA.............IRDAGIKAVKKL..........................R
tr|A0A651GLZ8|A0A651GLZ8_9BACT      ANETVHQHA.............MKDAGIRAVKKL...........................
tr|A0A7Y5V3S5|A0A7Y5V3S5_9BACT      VHELVHPAI.............LRDVAILRAREL......AD..................G
tr|A0A7W1SHC0|A0A7W1SHC0_9BACT      IDDLVHPAI.............LRDVAVRRAREL......AD..................G
tr|A0A7Y5PBT7|A0A7Y5PBT7_9BACT      VDELVHPAI.............LADVAIDRARKL......AD..................K
tr|A0A7Y5TXL8|A0A7Y5TXL8_9BACT      VDELVHPAI.............LADVAIDRARKL......AD..................K
tr|A0A423PQ96|A0A423PQ96_9GAMM      VDTVVAPEA.............LRDEAIRQARFA...........................
tr|U2G066|U2G066_9GAMM              IDQVVAPEA.............LLMEAIRQARIA...........................
tr|A0A2E0J1F4|A0A2E0J1F4_9GAMM      VDGVVPPEA.............LLDEAIRQVRSA..........................G
tr|A0A2D4SCM8|A0A2D4SCM8_9GAMM      VDKVVAPEA.............IIDAAIAHAKKL.....................ASDKPG
tr|L0WJH1|L0WJH1_9GAMM              VDEVVDAVA.............VYDRAVARAAQL...........................
tr|A0A1H5XF61|A0A1H5XF61_9GAMM      VDEVVEAVA.............VYDRAVARALEL...........................
tr|A0A2E9TS48|A0A2E9TS48_9GAMM      VDEVVEAVA.............VYDRAVARALEL...........................
tr|A0A095UE19|A0A095UE19_9GAMM      VDEVVEGVA.............VYDRAVARALEL...........................
tr|A0A7G2S9A0|A0A7G2S9A0_9GAMM      VDEVVEGVA.............VYDRAVARALEL...........................
tr|B5JU27|B5JU27_9GAMM              IDQIVGSEA.............LEERAVALALK............................
tr|A0A1Y0IHP1|A0A1Y0IHP1_9GAMM      VDAVVEPEA.............LLVKAVAMAKPR...........................
tr|A0A316FZ14|A0A316FZ14_9GAMM      VDEVVPVAN.............LQLAAEKRVLNM..........................A
tr|A0A498C231|A0A498C231_9GAMM      VDDLVAPPI.............LLGAARELIHEL...........................
tr|Q0A6T4|Q0A6T4_ALKEH              VDDLVAPPI.............LLDAARKRIDEL..........................A
tr|A0A3E0WL10|A0A3E0WL10_9GAMM      VDDVVPAPI.............LEQAAIELAQKL..........................V
tr|A0A3S1BU16|A0A3S1BU16_9GAMM      VDDVVPATI.............LLQTALGLVQKL..........................Q
tr|A0A7V8QFN9|A0A7V8QFN9_9GAMM      VDDVVPASI.............VMDVAVEQINRL...........................
tr|A0A1H8PPF9|A0A1H8PPF9_9GAMM      VDDVVPEPI.............LEDAAIRMVQKL..........................Q
tr|A0A6H0J0V7|A0A6H0J0V7_9GAMM      VDDVVPAPI.............LEDAAVKRVKEL..........................Q
tr|V5EZX6|V5EZX6_9VIBR              VDASVPNSI.............FLDVAKSYAQKS...........................
tr|A0A6I1QBM2|A0A6I1QBM2_9VIBR      VDASVPNSI.............FLDVAKSYAQKS...........................
tr|A0A511QRL5|A0A511QRL5_9VIBR      VDASVPQSI.............LMDVAKTYAAKS...........................
tr|A0A7Y0JZP4|A0A7Y0JZP4_9VIBR      VDASVPQSI.............LMDVAKTYAAKS...........................
tr|A0A193KD24|A0A193KD24_9VIBR      VDASVPQSI.............LMDVAKTYAAKS...........................
tr|A0A1S1HM72|A0A1S1HM72_PROST      VDDVVPETI.............LLDVAIQMAKK............................
tr|A0A7T8I613|A0A7T8I613_9GAMM      VDDVVPETI.............LLDVAIQMAKK............................
tr|A0A140NIF0|A0A140NIF0_PROSM      VDDVVPETI.............LLDVAIAMAKK............................
tr|A0A379GMB7|A0A379GMB7_PROST      VDDVVPETI.............LLDVAIAMAKK............................
tr|A0A379H393|A0A379H393_PROST      VDDVVPETI.............LLDVAIAMAKK............................
tr|B2Q0L5|B2Q0L5_PROST              VDDVVPETI.............LLDVAIAMAKK............................
tr|A0A6I3JUE9|A0A6I3JUE9_9GAMM      VDDVVPDTI.............LLDVAIAMAKK............................
tr|A0A7D4P594|A0A7D4P594_YERMW      VDDAVPQDI.............LLDVAIKRAKA............................
tr|A0A7U7IVK6|A0A7U7IVK6_YEREN      VDDVVPQDI.............LLDVAIQRAKA............................
tr|A0A447RIF5|A0A447RIF5_YEREN      VDDVVPQDI.............LLDVAIQRAKA............................
tr|A0A7H4ZLV9|A0A7H4ZLV9_YERP4      VDDVVPQDI.............LLDVAIQRAKA............................
tr|A0A0H3NX46|A0A0H3NX46_YERE1      VDDVVPQDI.............LLDVAIQRAKA............................
tr|A0A7T9XUV7|A0A7T9XUV7_YEREN      VDDVVPQDI.............LLDVAIQRAKA............................
tr|A0A7U7IYV8|A0A7U7IYV8_YEREN      VDDVVPQDI.............LLDVAIQRAKA............................
tr|A0A0H5G7G2|A0A0H5G7G2_YEREN      VDDVVPQDI.............LLNVAIQRAKA............................
tr|A0A2A7TDI2|A0A2A7TDI2_YERKR      VDDVVPQDI.............LLDVAIQRAKA............................
tr|A0A0T9M261|A0A0T9M261_YERKR      VDDVVPQDI.............LLDVAIQRAKA............................
tr|A0A0A0CMP1|A0A0A0CMP1_PHOLU      VDDAVPLDI.............LLDIAIEKVKKG...........................
tr|A0A329VG55|A0A329VG55_9GAMM      VDDAVPLDI.............LLDIAIEKVKKG...........................
sp|Q7N288|FADJ_PHOLL                VDDAVPLDI.............LLDIAIEKVKKG...........................
tr|A0A6L9JMX5|A0A6L9JMX5_PHOLM      VDDAVPLDI.............LLDIAIEKVKKG...........................
tr|A0A7X5HQ29|A0A7X5HQ29_PHOLM      VDDAVPLDI.............LLDIAIEKVKKG...........................
tr|A0A1C0U051|A0A1C0U051_9GAMM      VDDAVPLDI.............LLEIAVEKVKKG...........................
tr|A0A7X5QF62|A0A7X5QF62_9GAMM      VDDAVPLDI.............LLEVAVEK..............................I
```

```
tr|A0A2D0KWM9|A0A2D0KWM9_9GAMM  VDDAVPLDI.............LLDVAVQYVKK...........................
tr|A0A2D0LAI1|A0A2D0LAI1_9GAMM  VDDAVPLDI.............LLDVAVQYVKK...........................
tr|A0A2D0IXC8|A0A2D0IXC8_XENBU  VDDAVPLDI.............LLNVAVQYVKK...........................
tr|W1JAM5|W1JAM5_9GAMM          VDDAVPLDI.............LLDVAVQYVKK...........................
tr|A0A3D9UED3|A0A3D9UED3_9GAMM  VDDAVPLDI.............LLDVAVQYVKK...........................
tr|A0A1I3JCA9|A0A1I3JCA9_9GAMM  VDDAVPLDI.............LLDVAVQYVKK...........................
tr|A0A068QUL9|A0A068QUL9_9GAMM  VDDAVPLDI.............LLDVAVQYIKKG.........................N
tr|A0A0M0TCH6|A0A0M0TCH6_9GAMM  VDDAVPLDI.............LLDVAVQYVKK...........................
tr|A0A1Q5U854|A0A1Q5U854_9GAMM  VDEAVPLDI.............LLDVAVQYVKK...........................
tr|A0A2D0ISP1|A0A2D0ISP1_9GAMM  VDEAVPLDI.............LLDVAVQYVKK...........................
tr|A0A1Q5TUI8|A0A1Q5TUI8_9GAMM  VDDAVPLDI.............LLDVAVQYVKK...........................
tr|A0A2D0KJI7|A0A2D0KJI7_9GAMM  VDDAVPLDI.............LLDVAVQYVKK...........................
tr|A0A1Y2SB97|A0A1Y2SB97_9GAMM  VDDAVPLDI.............LLDVAVQYVKK...........................
tr|A0A1I5DWI7|A0A1I5DWI7_9GAMM  VDDAVPLDI.............LLDVAVQYVKK...........................
tr|A0A1I7GWY6|A0A1I7GWY6_9GAMM  VDDAVPLDI.............LLDVAVQYAKK...........................
tr|D3VKY8|D3VKY8_XENNA          VDDAVPLEI.............LLDVAVQYAKK...........................
tr|A0A2G0Q6Q9|A0A2G0Q6Q9_9GAMM  VDDAVPLDI.............LLDVAVQYVKK...........................
tr|A0A2D0JU24|A0A2D0JU24_9GAMM  VDDAVPLDI.............LLDVAVQYVKK...........................
tr|A0A0J5FN38|A0A0J5FN38_9GAMM  VDDAVPLDI.............LLDVAVQYVKK...........................
tr|A0A432XLD4|A0A432XLD4_9GAMM  VDDVVPASI.............LLDAAIELALKS..........................
tr|A0A2D8HU84|A0A2D8HU84_9GAMM  VAEVVPQSI.............LLDVAVEHALK..........................R
tr|A0A656X1Q2|A0A656X1Q2_9GAMM  VAEVVPQSI.............LLDVAVEHALK..........................R
tr|A0A4Q1QH79|A0A4Q1QH79_9GAMM  VTEVVPQSI.............LLDVAVEHALK..........................R
tr|A0A1G7LQE6|A0A1G7LQE6_9GAMM  VTEVVPQSI.............LLDVAVEHALK..........................R
tr|A0A432YVG7|A0A432YVG7_9GAMM  VTDVVPQSI.............LLDVAVEHALK..........................R
tr|A0A1J5N0P5|A0A1J5N0P5_9GAMM  VSEVVPPVI.............LLDVALKLAAKP..........................
tr|A0A0D8D548|A0A0D8D548_9GAMM  VNDVVPESI.............LIRTAEEMALA..........................G
tr|A0A3E0TNM2|A0A3E0TNM2_9GAMM  VDDVVPNTI.............LLETAEKLALAG..........................
tr|A0A3E0UD67|A0A3E0UD67_9GAMM  VDDVVPNTI.............LVATAEKLALAG..........................
tr|A0A3E0U0P4|A0A3E0U0P4_9GAMM  VDDVVPNTI.............LVATAEKLALAG..........................
tr|A0A0M2V8B1|A0A0M2V8B1_9GAMM  VDEVVPVSI.............LLEAAVKRALQ..........................G
tr|A0A285ITY5|A0A285ITY5_9GAMM  VDDVVPATI.............LLDAAVKRALQ..........................G
tr|A0A486XTB9|A0A486XTB9_9GAMM  VDEVVPQSI.............LLEAAVKRALK...........................
tr|I1E1G1|I1E1G1_9GAMM          VDEVVPNSI.............LLDAAVKLALK..........................G
tr|A0A1H6KJ68|A0A1H6KJ68_9GAMM  VDEVVANSI.............LLDAAVKRALK..........................G
tr|A0A0X3Y764|A0A0X3Y764_9GAMM  VDEVVANSI.............LLEAAVKRALK..........................G
tr|A0A2N1YEV4|A0A2N1YEV4_9GAMM  VDEVVANSI.............LLEAAVKRALK..........................G
tr|A0A3P3QCM3|A0A3P3QCM3_9GAMM  VDAVVPKSI.............LLEAAVQLALKG..........................
tr|F7NT72|F7NT72_9GAMM          VDAVVPKSI.............LLEAAVQLALKG..........................
tr|A0A3S2TW63|A0A3S2TW63_9GAMM  VDAVVPKSI.............LLEAAVQLALKG..........................
tr|A0A3D5BGW7|A0A3D5BGW7_9GAMM  VDAVVPKSI.............LLEAAVKMALA..........................G
tr|A0A5C7TI17|A0A5C7TI17_9GAMM  VDVVVPRSI.............LLEAAVKMALA..........................G
tr|A0A0U4W982|A0A0U4W982_9GAMM  VDVVVPRSI.............LLEAAVKMALA..........................G
tr|A0A2I0FCM2|A0A2I0FCM2_9GAMM  VDEVVPETV.............LLEVAVKLAKK...........................
tr|A0A2G2IVL1|A0A2G2IVL1_9GAMM  VDEVVPETV.............LLEVAVKLAKK...........................
tr|A6FI21|A6FI21_9GAMM          VDEVVPETV.............LLEVAVKLAKK...........................
tr|A0A4U1BNR2|A0A4U1BNR2_9GAMM  ADEMVPNTV.............LIEVAKKHLAKG.........................K
tr|E1SQ60|E1SQ60_FERBD          VEETVPLSV.............LVDVAREYALK..........................G
tr|A0A4Y6IZ09|A0A4Y6IZ09_9GAMM  VDDVVPESI.............LLQTAVEMALAG..........................
tr|A0A0C3QSZ7|A0A0C3QSZ7_9GAMM  VDDVVPESI.............LLQTAIEMALAG..........................
tr|A0A6L7HXW0|A0A6L7HXW0_9GAMM  VDDVVPESI.............LLQTAIEMALAG..........................
sp|A3QFP3|FADJ_SHELP            VDDVVPESI.............LLQTAIEMALAG..........................
tr|A0A1E5IXH7|A0A1E5IXH7_SHECO  VNDVVPESI.............LLRTAIEMALSG..........................
tr|A0A411PKQ0|A0A411PKQ0_9GAMM  VDDMVPGSI.............LLEAAIKLAKKG..........................
tr|A0A6G9QKM3|A0A6G9QKM3_9GAMM  VNDVVPNTI.............LLETAIELAKKG..........................
tr|A0A6P1UL63|A0A6P1UL63_9GAMM  VNDVVPNSI.............LLETAIELAKKS..........................
tr|A0A2N1ERI9|A0A2N1ERI9_9GAMM  VNDVVPNSI.............LLETAIELAKKS..........................
tr|A0A7W4FU55|A0A7W4FU55_9GAMM  VNDVVPNSI.............LLETAIELAKKS..........................
sp|Q8ECP7|FADJ_SHEON            VNDVVPQTI.............LLQTAVEMALAG..........................
tr|A0A501XZY8|A0A501XZY8_9GAMM  VNDVVPQTI.............LLQTAVEMALAG..........................
tr|A0A2W5DCZ0|A0A2W5DCZ0_SHEOE  VNDVVPQTI.............LLQTAVEMALAG..........................
tr|A0A1E3V3C8|A0A1E3V3C8_9GAMM  VNDVVPQTI.............LLQTAVEMALAG..........................
tr|A0A1Z4AI20|A0A1Z4AI20_9GAMM  VNDVVPQTI.............LLQTAVEMALAG..........................
tr|A0A7X9LJL9|A0A7X9LJL9_9GAMM  VNDVVPQTI.............LLQTAVEMALAG..........................
tr|A0A073KMY5|A0A073KMY5_9GAMM  VNDVVPQTI.............LLQTAVEMALAG..........................
sp|A0KV76|FADJ_SHESA            VNDVVPQTI.............LLQTAVEMALAG..........................
tr|A0A220UTH7|A0A220UTH7_9GAMM  VNDVVPQTI.............LLQTAVEMALAG..........................
tr|A0A5B8R6W1|A0A5B8R6W1_9GAMM  VNDVVPQTI.............LLQTAVEMALAG..........................
tr|V1DAI4|V1DAI4_9GAMM          VNDVVPQTI.............LLQTAVEMALAG..........................
tr|A0A448CPQ4|A0A448CPQ4_SHEPU  VNDVVPQTI.............LLQTAVEMALAG..........................
tr|A0A252ERQ3|A0A252ERQ3_SHEPU  VNDVVPQTI.............LLQTAVEMALAG..........................
sp|Q0HKD1|FADJ_SHESM            VNDVVPQTI.............LLQTAVEMALAG..........................
sp|Q0HWN3|FADJ_SHESR            VNDVVPQTI.............LLQTAVEMALAG..........................
tr|F7RQE3|F7RQE3_9GAMM          VNDVVPQTI.............LLQTAVEMALAG..........................
tr|B8EE98|B8EE98_SHEB2          VNDVVPQTI.............LLQTAVEMALAG..........................
sp|A6WQ25|FADJ_SHEB8            VNDVVPQTI.............LLQTAVEMALAG..........................
tr|A0A448EK41|A0A448EK41_9GAMM  VNDVVPQTI.............LLQTAVEMALAG..........................
tr|A0A553JHX1|A0A553JHX1_SHEHA  VDDMVPDSI.............LLQTAVEMALKG..........................
tr|B8CPY6|B8CPY6_SHEPW          VDDAVPESI.............LMRTAIEMALAG..........................
tr|A0A431WFC4|A0A431WFC4_9GAMM  VDDAVPESI.............LLETAVAMALKG..........................
tr|A8FTR7|A8FTR7_SHESH          VDDVVPESI.............LLETAVEMALKG..........................
tr|A0A431WNL0|A0A431WNL0_9GAMM  VDDVVPESI.............LLETAVEMALKG..........................
tr|A0A550AEC5|A0A550AEC5_9GAMM  VDEVVPESI.............LLTTAVEMALKG..........................
tr|A0A7L4WW90|A0A7L4WW90_9GAMM  VDEVVPESI.............LLTTAVEMALKG..........................
tr|A9DDU3|A9DDU3_9GAMM          VDDVVPESI.............LLATAVKMALKG..........................
tr|A0A330M2B9|A0A330M2B9_9GAMM  VDDVVPESI.............LLATAVEMALKG..........................
tr|A0A5N8UFC7|A0A5N8UFC7_9GAMM  VDDVVPESI.............LLATAVEMALKG..........................
tr|A0A1S6HN57|A0A1S6HN57_9GAMM  VDDVVPESI.............LLATAVEMALKG..........................
tr|D4ZMH7|D4ZMH7_SHEVD          VDDVVPESI.............LLATAVKMALKG..........................
tr|A0A3L8Q213|A0A3L8Q213_9GAMM  VNDMVPQSI.............LLESAINLAKS..........................G
tr|A0A3A6U4N9|A0A3A6U4N9_9GAMM  VSEVVPKTI.............LLEAAIKLAKAG..........................
tr|A0A4Q5MA37|A0A4Q5MA37_9GAMM  VHDVVPNSI.............LLDAAIELAKGG..........................
tr|A0A1L6LSX5|A0A1L6LSX5_9DELT  ADEVVHVSI.............LHEVAAQHALKL.........................A
tr|A0A2W4L9B9|A0A2W4L9B9_9PROT  VDEAVPEPL.............LLQVARERARAL.........................G
tr|A0A2W4M4E6|A0A2W4M4E6_9PROT  VDEVVARPV.............LESAAIERALMLLAP.......................
tr|A0A6I2GRX9|A0A6I2GRX9_9DELT  VDEVVPAPI.............LRAVALQRAREL..........................
tr|A0A0H4WMK2|A0A0H4WMK2_9DELT  VDEVVPVPI.............LRDIAVRRAKEL......AEGKLKVERRHGQGFKGVAR
tr|F8CJ36|F8CJ36_MYXFH          VDEVVPAPI.............LRAIAVRRAKEL......AEGKLKVERRHGQGFKGVAA
tr|A0A250K0F1|A0A250K0F1_9DELT  VDEVVPAPI.............LRAIAVRRAKEL......AEGTLKVERRHGQGFKGVAA
tr|A0A7Y6WFZ2|A0A7Y6WFZ2_9DELT  VDEVVPAPI.............LRAIAVRRAKEL......AEGKLKVDRRHGQGFKGVAA
tr|A0A7Y7C660|A0A7Y7C660_9DELT  VDEVVPAPI.............LRAIAVRRAKEL......AEGKLKVDRRHGQGFKGVAA
tr|A0A7Y4JFH2|A0A7Y4JFH2_MYXXA  VDEVVPAPI.............LRAIAVRRAKEL......AAGTLKVERRHGQGFKGVAA
tr|A0A4Y6CZQ9|A0A4Y6CZQ9_MYXXA  VDEVVPAPI.............LRAIAVRRAKEL......AAGTLKVDRRHGQGFKGVAA
```

```
tr|A0A7Y4IKV5|A0A7Y4IKV5_MYXXA  VDEVVPAPI.............LRAIAVRRAKEL......AEGTLKVDRRHGQGFKGVAA
tr|A0A511HHB0|A0A511HHB0_9DELT  VDEVVPAPI.............LRAIAVRRAKEL......AEGTLKVDRRHGQGFKGVAA
tr|A0A4Y6CKY7|A0A4Y6CKY7_MYXXA  VDEVVPAPI.............LRAIAVRRAKEL......AEGTLKVDRRHGQGFKGVAA
tr|Q1D1F2|Q1D1F2_MYXXD          VDEVVPTPI.............LRAIAVLRAKEL......ADGKLKVDRRHGQGFKGVAA
tr|A0A7Y4MA14|A0A7Y4MA14_MYXXA  VDEVVPTPI.............LRAIAVLRAKEL......ADGKLKVDRRHGQGFKGVAA
tr|A0A7T8Y4N9|A0A7T8Y4N9_MYXXA  VDEVVPAPI.............LRAIAVRRAKEL......AEGKLKVDRRHGQGFKGVAA
tr|L7UE67|L7UE67_MYXSD          VDEVVPTPI.............LRAVALQRAREL......AEGSLKPERTRGQGLT....
tr|A0A511T9X1|A0A511T9X1_MYXFU  VDEVVPVPI.............LRAIAVQRAKEL......AAGSLKPVRAHGQGLKS...
tr|A0A7Y7C9C4|A0A7Y7C9C4_9DELT  VDEVVPTPI.............LRAVALQRAREL......AEGTLKPERTHGQGFKAV..
tr|A0A540X7W8|A0A540X7W8_9DELT  VDEVVPTPI.............LRAIALQRAREL......AAGTLTPERAHGQGFKAV..
tr|A0A3A5FK19|A0A3A5FK19_9DELT  VDEVVPVPL.............LRSLALQRAGEL......AAGTLKVTRAHGQGLKAVAS
tr|A0A3A8JQL9|A0A3A8JQL9_9DELT  VDEVVPVPM.............LKDIALKRAEEL.......AAGTLKVERAHQGFKAVAQ
tr|A0A7Y4NFA4|A0A7Y4NFA4_9DELT  VDEVVPVPM.............LKDIALKRAAEL......AAGTLKVERAH.QGFKAVAQ
tr|A0A3A8HDQ7|A0A3A8HDQ7_9DELT  VDEVVPVPM.............LKDIALKRAAEL......AAGTLKVERAH.QGFKAVAQ
tr|A0A3A8GR90|A0A3A8GR90_9DELT  VDEVVPVPM.............LKDIALKRAAEL......AAGTLKVERAH.QGFKAVAQ
tr|A0A3A8SBD7|A0A3A8SBD7_9DELT  VDEVVPVPM.............LKDLALKRAAEL.......AAGTLKVERSHQGFKAVAQ
tr|A0A3A8T0I8|A0A3A8T0I8_9DELT  VDEVVPVPM.............LKDLALKRAAEL.......AAGTLKVERSHQGFKAVAQ
tr|A0A7X5BU07|A0A7X5BU07_9DELT  VDEVVPVPM.............LKDLALKRAAEL.......AAGTLKVERSHQGFKAVAQ
tr|A0A7Y1RVL2|A0A7Y1RVL2_9DELT  VDEVVPVPM.............LKDLALKRAAEL.......AAGTLKVERSHQGFKAVAQ
tr|A0A3A8THN2|A0A3A8THN2_9DELT  VDEVVPVPM.............LKDLALKRAAEL.......AAGTLKVERSHQGFKAVAQ
tr|A0A7Y1RX76|A0A7Y1RX76_9DELT  VDEVVPVPM.............LKDLALKRAAEL.......AAGTLKVERSHQGFKAVAQ
tr|A0A3A8RA58|A0A3A8RA58_9DELT  VDEVVPVPM.............LKDIALKRAAEL.......AAGTLKVERSHQGFKAVAQ
tr|A0A410RPB6|A0A410RPB6_CORCK  VDEVVPVPM.............LKDLALKRAAEL.......AAGTLKVERSHQGFKAVAQ
tr|A0A7Y4J474|A0A7Y4J474_CORCK  VDEVVPVPM.............LKDLALKRAAEL.......AAGTLKVERSHQGFKAVAQ
tr|A0A3A8I9Z6|A0A3A8I9Z6_9DELT  VDEVVPVPM.............LKDLALKRAAEL.......AAGTLKVERSHQGFKAVAQ
tr|H8MKE9|H8MKE9_CORCM          VDEVVPVPM.............LKDIALKRAVEL.......AAGTLKVERSHQGFKAVAQ
tr|A0A3A8H102|A0A3A8H102_9DELT  VDEVVPVPM.............LKDIALKRAAEL.......AAGTLKVERSHQGFKAVAQ
tr|A0A554FW33|A0A554FW33_9DELT  VDEVVPVPM.............LKDLALKRAAEL.......AAGTLKVERAHQGFKAVAQ
tr|A0A3A8NPC0|A0A3A8NPC0_9DELT  VDEVVPVPM.............LKALAVRRAAEL.......AAGTLKVERAHQGFKAVAQ
tr|A0A3A8JUV3|A0A3A8JUV3_9DELT  VDEVVPVPM.............LKALAVRRAAEL.......AAGTLKVERAHQGFKAVAQ
tr|A0A3A8NEX6|A0A3A8NEX6_9DELT  VDEVVPVPM.............LKALAVRRAAEL......AEGTLKVERAH.QGFKAVAQ
tr|A0A3A8JEU8|A0A3A8JEU8_9DELT  VDEVVPVPM.............LKRIALQRAAEL.......AAGTLKVERAHQGFKAVAA
tr|A0A3A8LI76|A0A3A8LI76_9DELT  VDEVVPVPM.............LKRIALQRAAEL......AAGTLKVERAH.QGFKAVAA
tr|A0A085WXN8|A0A085WXN8_9DELT  VDEVVPVPI.............LRSIALQRAREL......SAGALKVERPHGQRLKAVAA
tr|A0A2T4V0M5|A0A2T4V0M5_9DELT  VDEVVPAPI.............LRAVAVRRAREL......AEGMLKVERSRGQGLKAVAQ
tr|A0A0G2ZSW5|A0A0G2ZSW5_9DELT  VDEVVPAPI.............LRTLAVRRAREL......AEG.LKVERPRGQGLKAVAQ
tr|A0A3M2DKY0|A0A3M2DKY0_9DELT  VDDVVPEPI.............LVDVAIDMARAL........................AD
tr|A0A661NQ58|A0A661NQ58_9DELT  VDEVVPNAI.............LLDVAIETAQKL....................AGREAG
tr|A0A520YD99|A0A520YD99_9DELT  VDVVVPPSI.............LLETAAALALER..........................
tr|A0A7Y3BRE4|A0A7Y3BRE4_9DELT  VDVVVPPSI.............LLETAAALALE..........................R
tr|A0A2D9TF90|A0A2D9TF90_9DELT  VDEVVEPSI.............LVDVAAQHAVRL..........................
tr|A0A2E0TP32|A0A2E0TP32_9DELT  VDEVVPSAI.............VVEVAAKHAKKY.........................A
tr|A0A2E4Y3V1|A0A2E4Y3V1_9PROT  VDDVVCPES.............LEAVAVQVAQKL.........................A
tr|A0A2E6VRH4|A0A2E6VRH4_9DELT  VDTVVSPIG.............LKHVAIQAAQKL.........................V
tr|A0A1F9FB59|A0A1F9FB59_9DELT  VDAVTVPYG.............LEDAAVTTCKRL.......................IMG
```

```
ECHA_HUMAN
                                                          260                  270       280       290
ECHA_HUMAN                         ...ADKKISPK..R.........DKGLVE.......KLTAYAMTIPFVRQQVYKKVEEKV
tr|A0A4R6XLA8|A0A4R6XLA8_9GAMM     ...KR...KPK............K..LG.......LVDKAL.NTTVGRNYVLKKARETV
tr|A0A6A0IJT2|A0A6A0IJT2_9BACT     N..GTLVPSPR............R..RR.......LLERLAAAPPL.RGVVLGKARDQV
tr|A0A7V3QZU7|A0A7V3QZU7_9BACT     D..GS...IRRSAR.........K..RP.......WTERLLE.SPPGRLLLFRQARKSV
tr|A0A7V4SYJ6|A0A7V4SYJ6_9BACT     E..GS...LRP..G........RRK..RS.......LVERLL.STPPGRALVLKKARDSV
tr|A0A2A8D2N6|A0A2A8D2N6_9BACT     N..GS...LTV...........ERDTMS.......FADKLLESNTVSRRVIYQKAGEKA
tr|A0A2N1TGP0|A0A2N1TGP0_9SPIR     S..GR...LKRNMR.........K..RS.......FMEKILEGNFLGRRIIFSQARKMV
tr|A0A1G3QGW4|A0A1G3QGW4_9SPIR     R..KG...FRR..K.........R..KR.......FLADMFLESPLGRGMVFKQARQMV
tr|A0A1G3QTY3|A0A1G3QTY3_9SPIR     Q..KK...IKR..K........RS..RS.......LMDAFLES.PFGRNRVFKQARQMV
tr|A0A7X9L359|A0A7X9L359_9DELT     SGTDP...ARK..KRQGLQQKVLDA..........TLKRFIDETPVGQRIVFNQARSQV
tr|A0A522CJY0|A0A522CJY0_9SPIR     K..KK...FKR..K........RK..RS.......FVDAFLE.SSFGRGVVFTQATKMV
tr|A0A2N1RRJ3|A0A2N1RRJ3_9SPIR     IKKGR...ILR..K........RK..RG.......LVSFLLDSTSPGRAIVFSQARKMV
tr|A0A2N2KFN7|A0A2N2KFN7_9DELT     .K.GS...SKR.KR.........Q..RS.......FVSFLLESNPVGRAVVFAQARKMI
tr|A0A1V6AXX9|A0A1V6AXX9_9DELT     .R.GN...IKR.KR.........K..RS.......FSSFLLESNPVGRSIVFSQARKMV
tr|A0A2N2HZL0|A0A2N2HZL0_9DELT     VNRGN...ITR.KR.........K..RS.......FVTFLMESNPVGRAIVFSQARKMV
tr|A0A5E8ARP3|A0A5E8ARP3_9BACT     T..GQ...YRR..K........SK..MP.......LASKVLESNPLTRGIIFSQATKMM
tr|A0A662A7B1|A0A662A7B1_9BACT     S..GK...FKR..K........DK..RP.......FLAKVLESNPITRNIIFKKAGEIV
tr|D7CV16|D7CV16_TRURR             A..GG...GAQ..G........GL..KG.......LRSALLERTPLNR.LIYRQALEGV
tr|A0A3C2AKX4|A0A3C2AKX4_9FLAO     K..AP...LKR..E........DK..RS.......GMEKFLEGNPITRNIIFKKAKEMV
tr|A0A3B8ZLN3|A0A3B8ZLN3_9PLAN     K..GP...LQR..D........DK..RS.......MTTKILEGTGFTRGIVFKKAKEMV
tr|A0A345UGV8|A0A345UGV8_9BACT     ....E...KPK..S........GK..LP.......LVAKLL.TNTAARNIAFSKARETV
tr|A0A6C1P6A2|A0A6C1P6A2_9BACT     T..GW...KPK..R.........K..IA.......LMDRALTQTFL.RGIALKKARETV
tr|A0A3M1X9W8|A0A3M1X9W8_9BACT     R..GS...KPR..R........PR..RT.......LTTWVLEANPLGRKLLFKKARQMV
tr|A0A354C796|A0A354C796_9DELT     D..QP...PER..K........KK..QP.......FVVKLLESNALSRSIVYKKAAELV
tr|A0A3M1NUM8|A0A3M1NUM8_9BACT     K..NP...KKH..S........KK..QN.......LMNTVLEGNPFTRKIIYKKAREMT
tr|A0A3M2KRC8|A0A3M2KRC8_9BACT     .AKKP...IKR..K........KR..IS.......PAERLLESNPLTRRLVYKKARQMV
tr|A0A5S9IHR0|A0A5S9IHR0_9BACT     ...KP...HKR..K........KR..MG.......FMEKALESTPLTKNIIYNTAKKQV
tr|A0A6M1ST81|A0A6M1ST81_9BACT     K..GK...FSR..K........DK..RG.......LGAKLLEGNPIGRKIIFSQARKRA
tr|A0A521AAE5|A0A521AAE5_9BACT     K..ER...FKR..K........DK..RS.......LLHQLMEGLSPLRKIIYSQARKRT
tr|A0A1M4ZMD6|A0A1M4ZMD6_9BACT     S..DG...FER..K........DK..RS.......LFHQLTEGLSPLRKIIYSQARKRT
tr|A0A2A2GCS2|A0A2A2GCS2_9BACT     S..GK...FER..E........DK..RS.......LVHQLMEGMSPLRNIIYSQARKRA
tr|A0A5D3YIH7|A0A5D3YIH7_9BACT     S..GK...FER..K........DK..RS.......LAHQLMEGLSPLRKIIYSQARKRA
tr|A0A6M1T9G0|A0A6M1T9G0_9BACT     K..DN...FER..K........DK..RS.......LLHQLMEGLSPLRKIIYSQARKRS
tr|A0A6A8Q1Y8|A0A6A8Q1Y8_9BACT     D..GK...FDR..K........DK..RS.......FVEKLLEGNPIGRKVIFSQARKKT
tr|A0A521BVJ3|A0A521BVJ3_9BACT     ...NG...ASH..K........VK..RS.......FFEKLLEGNPLGRKIIFSQARKKT
tr|A0A359E0Y8|A0A359E0Y8_9BACT     D..GK...FDR..K........DK..RP.......LMHKILEGNPLGRKIIFSQARKKT
tr|A0A2D8CIM6|A0A2D8CIM6_9BACT     D..GK...FDR..K........DK..RP.......LMHKILEGNPLGRKIIFSQARKKT
tr|A0A2D9FWF5|A0A2D9FWF5_9BACT     D..GK...FDR..K........DK..RP.......FLHKVLEGNPLGRTIIFSQARKKA
tr|A0A1B6YB70|A0A1B6YB70_9BACT     S..GK...FER..T........DK..RT.......SMEKMIEGNGLGRKIVFSQALKQT
tr|A0A2D4ZTC0|A0A2D4ZTC0_9BACT     S..GN...FTR..K........DK..RS.......SMEKMIEGNALGRKIVFSQALKQT
tr|A0A3D4UVW7|A0A3D4UVW7_9BACT     S..GN...FTR..K........DK..RS.......SMEKMIEGNALGRKIVFSQALKQT
tr|A0A3F3I3M2|A0A3F3I3M2_9BACT     S..GN...FTR..K........DK..RS.......SMEKMIEGNALGRKIVFSQALKQT
tr|A0A3D1G7L9|A0A3D1G7L9_9BACT     K..GP...HQR..K........DK..RS.......LVEKLLEGNPIGRSIIFSQARKKT
tr|A0A3M8G1Y8|A0A3M8G1Y8_9BACT     G..GA...VNR..K........DR..RS.......FVEKVLESNPIGRNIIFSQARKKT
tr|A0A5Q4F391|A0A5Q4F391_9BACT     G..GT...FVR..K........DR..RS.......LAEKALEGNPATRALIFAQARKQT
tr|A0A6I7NPV0|A0A6I7NPV0_9BACT     ...KK...PEN..P........DK..RS.......LGEKILEGNPAGRSIIYKQARKRT
tr|A0A651G1J2|A0A651G1J2_9BACT     ...DG...STK..K........GR..RS.......LLEKIVEGNPIGRKIIFNQARKQT
tr|A0A371QRK5|A0A371QRK5_9BACT     E..KT...AKH..P........DK..RS.......LAEKALESNPLTRKVIFSQARKQT
tr|A0A2N0VGJ5|A0A2N0VGJ5_9BACT     N..GK...AKQ..P........DK..RS.......FFEKILEGNPIGRKIIFSQARKRT
tr|A0A316TYJ0|A0A316TYJ0_9BACT     D..KK...ADH..P........DK..RS.......FAEKALESNPIGRSILFNQALKRT
tr|A0A651GLZ8|A0A651GLZ8_9BACT     .REKK...GEQ..P........DN..RS.......LAEKALEGNPLGRNIIFKQALQQT
tr|A0A7Y5V3S5|A0A7Y5V3S5_9BACT     S..RK....RN..A........RL..KP....GGLRNALLDDNPVGRAMVLRKAREQV
tr|A0A7W1SHC0|A0A7W1SHC0_9BACT     A..RK....PS..R........TA..KG....RGAAGVLLEDNPLGRRVVFRQAKAGV
tr|A0A7Y5PBT7|A0A7Y5PBT7_9BACT     TLVGR...RSP..K........DR..GA.......AGVLLEDNPLGRALVFRKAREGV
tr|A0A7Y5TXL8|A0A7Y5TXL8_9BACT     TLVGR...RSP..K........DR..GA.......AGVLLEDNPLGRALVFRKAREGV
tr|A0A423PQ96|A0A423PQ96_9GAMM     ...GK...RSL.TGSASLFGRARARIGAG...VNGLTHAALEDTTLGRRLLFAQARKRT
tr|U2G066|U2G066_9GAMM             ...GK...RSS.TASASLIQRAQGRMRAG...VGGLTNAALEDNPAGRRVLFDQARKTL
tr|A0A2E0J1F4|A0A2E0J1F4_9GAMM     K..RS...LTS..S.....ASVIGRARERVGEGVAGLTAAALEDNFAGRRVLFDQARKQL
tr|A0A2D4SCM8|A0A2D4SCM8_9GAMM     KNRGL...ASR.IGDLMALPGNLQD..KA.......MEVALEENFAGRKVLFSQARQQV
tr|L0WJH1|L0WJH1_9GAMM             ...AG...EKD..DAGISLGDYFSR..EG.......LTKAALEQNSLGRKVVFDQARKQA
tr|A0A1H5XF61|A0A1H5XF61_9GAMM     ...AD...AKE..ESGVNFASYFSK..DG.......ITKAALEQNSLGRKMVFDQARKQT
tr|A0A2E9TS48|A0A2E9TS48_9GAMM     ...VD...AKE..ESGVDLASYFSK..DG.......LTKAALEQNSLGRKMVFDQARKQT
tr|A0A095UE19|A0A095UE19_9GAMM     ...SD...ARE..ESSVNLASYFSK..DG.......LTKAALEQNSLGRKVVFDQARKQT
tr|A0A7G2S9A0|A0A7G2S9A0_9GAMM     ...AD...ARE..ESSVNLASYFSK..DG.......LTKAALEQNSLGRKVVFDQARKQT
tr|B5JU27|B5JU27_9GAMM             ...ST...APR..PAK...SMELSQ..EA.......LQELALEKNPVGRKVVFDQARKQA
tr|A0A1Y0IHP1|A0A1Y0IHP1_9GAMM     ...GK....RKVVNGGFSIPVSTDD.........LMALALEKNAMGRKVLFDQAGKKA
tr|A0A316FZ14|A0A316FZ14_9GAMM     KAGHW...RKSSDEPSVSWKGAMSA..SG.......AQKLALESNSFGRKIIFDKARKTV
tr|A0A498C231|A0A498C231_9GAMM     ...AG...QRR..Q.........RPKRP.......LATRLLENNPLGRRLVYRQSERTV
tr|Q0A6T4|Q0A6T4_ALKEH             E..PR...RHR..P.........K..RP.......LTTRLLENNPLGRRLVYRQSERTV
tr|A0A3E0WL10|A0A3E0WL10_9GAMM     KRRET...AQR............K..RS.......LMDWALEGNPVGRKVMFDQARKQM
tr|A0A3S1BU16|A0A3S1BU16_9GAMM     H..GR...ATL..RKKNASLLSVQR..........LTKLALEDNPLGRRVLFQKAREQV
tr|A0A7V8QFN9|A0A7V8QFN9_9GAMM     ...QGRHRRAR.EPGAGSTMERVTR..........W...ALEKNPLGRSVVFKQAEKQM
tr|A0A1H8PPF9|A0A1H8PPF9_9GAMM     GGKSG...SKR............R..RT.......WMESALEGNPLGRRIVFDQARKQA
tr|A0A6H0J0V7|A0A6H0J0V7_9GAMM     E..KQ...RGN..K........RR..RS.......WMDTLLEGNPIGRRIVFDQARKQA
tr|V5EZX6|V5EZX6_9VIBR             ........KPK...........RK..SS.......TKEKLISSVGLSRKVIFDQAAKKT
tr|A0A6I1QBM2|A0A6I1QBM2_9VIBR     ........KPK...........RK..SS.......TKEKLISSVGLSRKVIFDQAAKKT
tr|A0A511QRL5|A0A511QRL5_9VIBR     ........KPK...........RK..AP.......AKEKLISSIGLSRKVIFDQAAKKT
tr|A0A7Y0JZP4|A0A7Y0JZP4_9VIBR     ........KPK...........RK..AP.......AKEKLISSIGLSRKVIFDQAAKKT
tr|A0A193KD24|A0A193KD24_9VIBR     ........KPK...........RK..AP.......AKEKLISSIGLSRKVIFDQAAKKT
tr|A0A1S1HM72|A0A1S1HM72_PROST     ...GK...IQR...........AP..IH.......WQQRLLSSRLLRNKV.FSTAKQTV
tr|A0A7T8I613|A0A7T8I613_9GAMM     ...GK...IQR...........AP..IH.......WQQRLL.SSRLLRNKVFSTAKQTV
tr|A0A140NIF0|A0A140NIF0_PROSM     ...GK...IQR..A.........P..IH.......WQHRLL.SSNLLRNKVFSSAKQTV
tr|A0A379GMB7|A0A379GMB7_PROST     ...GK...IQR...........AP..IH.......WQHRLL.SSNLLRNKVFSSAKQTV
tr|A0A379H393|A0A379H393_PROST     ...GK...IQR...........AP..IH.......WQHRLL.SSNLLRNKVFSSAKQTV
tr|B2Q0L5|B2Q0L5_PROST             ...GK...IQR...........AP..IH.......WQHRLL.SSNLLRNKVFSSAKQTV
tr|A0A6I3JUE9|A0A6I3JUE9_9GAMM     ...GK...VQR..A.........P..LP.......WQQRLL.SSSLLRNKVFSSAKQTV
tr|A0A7D4P594|A0A7D4P594_YERMW     ...GW...LDK..P.........V..VP.......WQERLLSG.PLGKALLFNIVRKKT
tr|A0A7U7IVK6|A0A7U7IVK6_YEREN     ...GW...LDK..P.........A..LP.......WQERLLSG.PLGKALLFNIVRKKT
tr|A0A447RIF5|A0A447RIF5_YEREN     ...GW...LDK...........PA..LP.......WQERLL.SGPLGKALLFNIVRKKT
tr|A0A7H4ZLV9|A0A7H4ZLV9_YERP4     ...GW...LDK...........PA..LP.......WQERLL.SGPLGKALLFNIVRKKT
tr|A0A0H3NX46|A0A0H3NX46_YERE1     ...GW...LDK...........PA..LP.......WQERLL.SGPLGKALLFNIVRKKT
tr|A0A7T9XUV7|A0A7T9XUV7_YEREN     ...GW...LDK...........PA..LP.......WQERLL.SGPLGKALLFNIVRKKT
tr|A0A7U7IYV8|A0A7U7IYV8_YEREN     ...GW...LDK..P.........A..LP.......WQERLLSG.PLGKALLFKIVRKKT
tr|A0A0H5G7G2|A0A0H5G7G2_YEREN     ...GW...LDK...........PA..LP.......WQERLL.SGPLGKALLFKIVRKKT
tr|A0A2A7TDI2|A0A2A7TDI2_YERKR     ...GW...LDK..P.........V..LP.......WQERLLSG.PLGKALLFNIVRKKT
tr|A0A0T9M261|A0A0T9M261_YERKR     ...GW...LDK...........PV..LP.......WQERLL.SGPLGKALLFNIVRKKT
tr|A0A0A0CMP1|A0A0A0CMP1_PHOLU     ...IP...VRK..P............LP.......WQQRLLVG.PLGRYFLFNIVRKKT
tr|A0A329VG55|A0A329VG55_9GAMM     ...IP...VRK..P............LP.......WQQRLLVG.PVGRYFLFNIVRKKT
sp|Q7N288|FADJ_PHOLL               ...IP...VRK..P............LP.......WQQRLLVG.PVGRYFLFNIVRKKT
tr|A0A6L9JMX5|A0A6L9JMX5_PHOLM     ...IP...VRK..P............LP.......WQQRLL.VGPVGRYFLFNIVRKKT
tr|A0A7X5HQ29|A0A7X5HQ29_PHOLM     ...IP...VRK..P............LP.......WQQRLL.VGPVGRYFLFNIVRKKT
tr|A0A1C0U051|A0A1C0U051_9GAMM     ...IP...MRK............S..LP.......WQQRLLVG.PVGRYFLFNIVRKKT
tr|A0A7X5QF62|A0A7X5QF62_9GAMM     K..KG...IPV..R........KS..LP.......WQQRLLVG.PVGRYFLFNIVRKKT
```

```
tr|A0A2D0KWM9|A0A2D0KWM9_9GAMM  ...GV...AKR..K..........P..LA.......WSQRLL.ASALGRPLLFRMVQQKT
tr|A0A2D0LAI1|A0A2D0LAI1_9GAMM  ...GV...AKR..K..........P..LA.......WSQRLL.ASALGRPLLFRMVQQKT
tr|A0A2D0IXC8|A0A2D0IXC8_XENBU  ...GI...IKR..K..........P..LA.......WSQRLL.ASALGRPLLFRMVQQKT
tr|W1JAM5|W1JAM5_9GAMM          ...GI...IKR..K..........P..LA.......WSQRLL.ASALGRPLLFQMVQQKT
tr|A0A3D9UED3|A0A3D9UED3_9GAMM  ...GI...IKR..K..........P..LA.......WSQRLL.ASALGRPLLFQMVQQKT
tr|A0A1I3JCA9|A0A1I3JCA9_9GAMM  ...GA...VKR..K..........P..IA.......WSQRML.ASALGRPLLFHMVQKKT
tr|A0A068QUL9|A0A068QUL9_9GAMM  VKKGI...VKR..K..........P..LA.......WSQRVL.ASTLGRPLLFSMVHQKT
tr|A0A0M0TCH6|A0A0M0TCH6_9GAMM  ...GI...VKR..K..........P..LV.......WSQRIL.ASALGRPLLFQMVHKKT
tr|A0A1Q5U854|A0A1Q5U854_9GAMM  ...GI...VKR..K..........P..LA.......WPQRIL.ASALGRPLLFQMVHKKT
tr|A0A2D0ISP1|A0A2D0ISP1_9GAMM  ...GI...VKR............KP..LA.......WSQRIL.ASALGRPLLFQMVHKKT
tr|A0A1Q5TUI8|A0A1Q5TUI8_9GAMM  ...GS...VKR..K..........P..LA.......WSQRIL.ASALGRPLLFQMVHKKT
tr|A0A2D0KJI7|A0A2D0KJI7_9GAMM  ...GI...VKR..K..........P..LA.......WSQRIL.ASTLGRPLLFQMVHKKT
tr|A0A1Y2SB97|A0A1Y2SB97_9GAMM  ...GT...VKR..K..........P..LA.......WPQRML.ASPLSRPLLFRMVHKKT
tr|A0A1I5DWI7|A0A1I5DWI7_9GAMM  ...GT...VKR..K..........P..LA.......WSQRML.ASPLGRPLLFQMVHKKT
tr|A0A1I7GWY6|A0A1I7GWY6_9GAMM  ...GI...IKR..K..........P..LA.......WSQRIM.ASALGRPLLFHMVHQKT
tr|D3VKY8|D3VKY8_XENNA          ...GI...IKR..K..........P..LA.......WSQRIM.ASALGRPLLFHMVHQKT
tr|A0A2G0Q6Q9|A0A2G0Q6Q9_9GAMM  ...GV...VKR..K..........P..LA.......WSQRIL.ASALGRPLLFRMVHQKT
tr|A0A2D0JU24|A0A2D0JU24_9GAMM  ...GI...AKR..K..........P..LA.......WSQRIL.ASALGRPLLFHTVHQKT
tr|A0A0J5FN38|A0A0J5FN38_9GAMM  ...GI...AKR............KP..LA.......WSQRIL.ASALGRPLLFHTVHQKT
tr|A0A432XLD4|A0A432XLD4_9GAMM  ...KA...KVR..K.........VQ..HS.......TLNKMLEGTGVGRGIIFSKAREQA
tr|A0A2D8HU84|A0A2D8HU84_9GAMM  K..PK....ST..K.........PP..LK.......GMSKVLEATRFGRDIIFKKAGEQA
tr|A0A656X1Q2|A0A656X1Q2_9GAMM  K..PK....ST..K.........PP..LK.......GMSKVLEATRFGRDIIFKKAGEQA
tr|A0A4Q1QH79|A0A4Q1QH79_9GAMM  K..PK....AS..K.........PP..LK.......GFSKLLEATKFGRDIIFKKAGEQA
tr|A0A1G7LQE6|A0A1G7LQE6_9GAMM  K..PK....AS..K.........AP..LK.......GFSKVLEGTKFGRDIIFKKAGEQA
tr|A0A432YVG7|A0A432YVG7_9GAMM  K..PK....AS..K.........AP..LK.......GFSKILEGTKFGRDIIFKKAGEQA
tr|A0A1J5N0P5|A0A1J5N0P5_9GAMM  ...IK...KTK..P..........K..LD.......NVNALLQATSAGRNIMFSQAEKSV
tr|A0A0D8D548|A0A0D8D548_9GAMM  K..VK....AK..P.........RK..MS.......VMDKLLEGNSFGRNIVFKQAAKTV
tr|A0A3E0TNM2|A0A3E0TNM2_9GAMM  ...KV...KAK..K.........LK..RS.......LVDNLLEGNSIGRKIVFDQASKSV
tr|A0A3E0UD67|A0A3E0UD67_9GAMM  ...KV...KAK..Q.........LK..RS.......LMDKLLEGNSFGRKIVFDQASKSV
tr|A0A3E0U0P4|A0A3E0U0P4_9GAMM  ...KV...KAK..Q.........LK..RS.......LMDKLLEGNSFGRKVVFDQASKSV
tr|A0A0M2V8B1|A0A0M2V8B1_9GAMM  K..TK....AA..A.........KD..KG.......LLAKLLENTAPGRNLLFSQALKQT
tr|A0A285ITY5|A0A285ITY5_9GAMM  K..AK...SVS..K.........DK..GI........VAKLLENTGPGRNLLFSQALKQT
tr|A0A486XTB9|A0A486XTB9_9GAMM  ...GK...PQR.SA.........VK..LN.......LVGKVLERTSFGRNVLFNQARKQT
tr|I1E1G1|I1E1G1_9GAMM          K..PK....RE..P.........VK..LN.......LVGKVLERTPFGRNILFSQARKQT
tr|A0A1H6KJ68|A0A1H6KJ68_9GAMM  K..LE...REQ..P..........K..LN.......LVGKVLERTPFGRNILFSQARKQT
tr|A0A0X3Y764|A0A0X3Y764_9GAMM  K..PQ...REQ..P..........K..LN.......LVGKVLERTPFGRNILFSQARKQT
tr|A0A2N1YEV4|A0A2N1YEV4_9GAMM  K..PQ...REQ..P..........K..LN.......LVGKVLERTPFGRNILFSQARKQT
tr|A0A3P3QCM3|A0A3P3QCM3_9GAMM  ...KP...AAR..D.........VK..LN.......FVGKVLEKTPFGRAIVFSQAEKQT
tr|F7NT72|F7NT72_9GAMM          ...KP...AAR..D.........VK..LN.......FVGKVLEKTPFGRAIVFSQAEKQT
tr|A0A3S2TW63|A0A3S2TW63_9GAMM  ...KP...AAR..E.........VK..LN.......FVGKVLEKTPFGRAVVFSQAEKQT
tr|A0A3D5BGW7|A0A3D5BGW7_9GAMM  K..PD...RSK..A.........VK..LP.......FVGQLLEFTSIGRGILFSQATKQT
tr|A0A5C7TI17|A0A5C7TI17_9GAMM  K..PD...RSK..P.........VK..LP.......LVGQLLEFTSIGRGILFSQAQKQT
tr|A0A0U4W982|A0A0U4W982_9GAMM  K..PD...RSK..P.........VK..LP.......LVGQLLEFTSIGRGILFSQAEKQT
tr|A0A2I0FCM2|A0A2I0FCM2_9GAMM  ...GK...IER..K.........LK..RD.......LTSKLLETNKFGRNIMFDQATKQT
tr|A0A2G2IVL1|A0A2G2IVL1_9GAMM  ...GK...IQR..K.........LK..RD.......LTSKLLETNKFGRNIMFDQAKKQT
tr|A6FI21|A6FI21_9GAMM          ...GK...IQR..K.........LK..RD.......LTSKLLETNKFGRNIMFDQAKKQT
tr|A0A4U1BNR2|A0A4U1BNR2_9GAMM  R..GK...VAP.............K..LK.......GAEKLLEATSAGRNIIYKQALKTV
tr|E1SQ60|E1SQ60_FERBD          K..RK....AV..K.........PN..LK.......GAEKLLESTAFGRKVIFDQAGKKV
tr|A0A4Y6IZ09|A0A4Y6IZ09_9GAMM  ...AR...PAK..K.........RK..VS.......LVEKLLEGTPVGRNIIFDQALKQV
tr|A0A0C3QSZ7|A0A0C3QSZ7_9GAMM  ...AR...PAK..K.........RK..VP.......MVEKLLEGTPVGRNIIFDQALKQV
tr|A0A6L7HXW0|A0A6L7HXW0_9GAMM  ...AR...PVK..K.........PK..LS.......TVEKLLEGTPVGRNIIFEQALKQV
sp|A3QFP3|FADJ_SHELP            ...AR...PAK..K.........PK..LS.......TVEKLLEGTPVGRNIIFEQALKQV
tr|A0A1E5IXH7|A0A1E5IXH7_SHECO  ...KK...MPK..K.........AK..AS.......MVNRVLEGTSAGRNIIFDQALKQV
tr|A0A411PKQ0|A0A411PKQ0_9GAMM  ...KK...AAN..K.........VK..PT.......LVNKVLESTSFTRDVVFKQAGKQV
tr|A0A6G9QKM3|A0A6G9QKM3_9GAMM  ...KS...AAK..P.........VK..KS.......ALNKLLESTSFTRDIVFDQASKQV
tr|A0A6P1UL63|A0A6P1UL63_9GAMM  ...KQ...AAK..P.........VV..QS.......KINQFLESSSFTRDIMFDQARKQV
tr|A0A2N1ERI9|A0A2N1ERI9_9GAMM  ...KQ...AAK..P.........VV..QS.......KINQFLESSSFTRDIMFDQARKQV
tr|A0A7W4FU55|A0A7W4FU55_9GAMM  ...KQ...AAK..P.........VV..QS.......KLNQFLESSSFTRDIMFDQARKQV
sp|Q8ECP7|FADJ_SHEON            ...KQ...IAK..P.........VK..KS.......LVNQLLEGTGFGRNIIFDQAAKQV
tr|A0A501XZY8|A0A501XZY8_9GAMM  ...KK...TAK..P.........VK..KS.......LVNQLLEGTGFGRNIIFDQAAKQV
tr|A0A2W5DCZ0|A0A2W5DCZ0_SHEOE  ...KK...TAK..P.........VK..KS.......LVNQLLEGTGFGRNIIFDQAAKQV
tr|A0A1E3V3C8|A0A1E3V3C8_9GAMM  ...KK...TAK..P.........VK..KS.......LVNQLLEGTGFGRNIIFDQAAKQV
tr|A0A1Z4AI20|A0A1Z4AI20_9GAMM  ...KK...TAK..P.........VK..KS.......LVNQLLEGTGFGRNIIFDQAAKQV
tr|A0A7X9LJL9|A0A7X9LJL9_9GAMM  ...KK...TAK..P.........VK..KS.......LVNQLLEGTGFGRNIIFDQAAKQV
tr|A0A073KMY5|A0A073KMY5_9GAMM  ...KK...TAK..P.........VK..KS.......LVNQLLEGTGFGRNIIFDQAAKQV
sp|A0KV76|FADJ_SHESA            ...KR...TAK..P.........VK..KS.......LVNQLLEGTGFGRNIIFDQAAKQV
tr|A0A220UTH7|A0A220UTH7_9GAMM  ...KR...TAK..P.........VK..KS.......LVNQLLEGTGFGRNIIFDQAAKQV
tr|A0A5B8R6W1|A0A5B8R6W1_9GAMM  ...KR...TAK..P.........VK..KS.......LVNQLLEGTGFGRNIIFDQAAKQV
tr|V1DAI4|V1DAI4_9GAMM          ...KR...TAK..P.........VK..KS.......LVNQLLEGTGFGRNIIFDQAIKQV
tr|A0A448CPQ4|A0A448CPQ4_SHEPU  ...KR...TAK..P.........VK..KS.......LVNQLLEGTGFGRNIIFDQAAKQV
tr|A0A252ERQ3|A0A252ERQ3_SHEPU  ...KR...TAK..P.........VK..KS.......LVNQLLEGTGFGRNIIFDQAAKQV
sp|Q0HKD1|FADJ_SHESM            ...KR...TAK..P.........VK..KS.......LVNQLLEGTGFGRNIIFDQAAKQV
sp|Q0HWN3|FADJ_SHESR            ...KR...TAK..P.........VK..KS.......LVNQLLEGTGFGRNIIFDQAAKQV
tr|F7RQE3|F7RQE3_9GAMM          ...KR...ASK..P.........IK..KS.......LVNQVLEGTSFGRNIIFDQATKQV
tr|B8EE98|B8EE98_SHEB2          ...KR...APK..P.........VK..KS.......LVNQVLEGTSFGRNIIFDQATKQV
sp|A6WQ25|FADJ_SHEB8            ...KR...APK..P.........VK..KS.......LVNQVLEGTSFGRNIIFDQATKQV
tr|A0A448EK41|A0A448EK41_9GAMM  ...KR...APK..P.........VK..KS.......LVNQVLEGTSFGRNIIFDQATKQV
tr|A0A553JHX1|A0A553JHX1_SHEHA  ...KR...APK..K.........PK..LS.......LVNRLLEGSNIGRNIIFDQAGKQV
tr|B8CPY6|B8CPY6_SHEPW          ...KR...TAK..K.........KK..QS.......FINKMLEGTSAGRNIIFDQAGKQV
tr|A0A431WFC4|A0A431WFC4_9GAMM  ...KR...APK..K.........VK..KS.......LVTRLLEGTPPGRNIIFDQAGKQV
tr|A8FTR7|A8FTR7_SHESH          ...KR...APK..K.........VK..KS.......LVTRLLEGTPPGRNIIFDQAGKQV
tr|A0A431WNL0|A0A431WNL0_9GAMM  ...KR...APK..K.........VK..KS.......LVTRLLEGTPPGRNIIFDQASKQV
tr|A0A550AEC5|A0A550AEC5_9GAMM  ...KR...TAK..K.........VK..KS.......LVNKLLEGTPVGRNIIFDQASKQV
tr|A0A7L4WW90|A0A7L4WW90_9GAMM  ...KR...TAK..K.........VK..KS.......LVNKLLEGTPVGRNIIFDQASKQV
tr|A9DDU3|A9DDU3_9GAMM          ...KR...VAK..K.........VK..KS.......LLNKLLEGTSFGRNIIFDQAGKQV
tr|A0A330M2B9|A0A330M2B9_9GAMM  ...KR...VPK..K.........VK..KS.......LLNKLLEGTSFGRNIIFDQAGKQV
tr|A0A5N8UFC7|A0A5N8UFC7_9GAMM  ...KR...APK..K.........VN..KS.......LVNKLLEGTPVGRNIIFDQAGKQV
tr|A0A1S6HN57|A0A1S6HN57_9GAMM  ...KR...SPK..K.........VK..KS.......VVNKLLEGTSVGRNIIFDQAGKQV
tr|D4ZMH7|D4ZMH7_SHEVD          ...KV...APK..K.........EN..KS.......LVNKLLEGTAVGRNIIFDQAGKQV
tr|A0A3L8Q213|A0A3L8Q213_9GAMM  K..VA....PK..P.........RK..KS.......LINRLLEDTSMGRNIVFDQAAKKV
tr|A0A3A6U4N9|A0A3A6U4N9_9GAMM  ...HK...KAK..P.........VK..QS.......LVNRVLEATPIGRNIMFDQATKQV
tr|A0A4Q5MA37|A0A4Q5MA37_9GAMM  ...KK...KPA..K.........RK..TP.......LVNKILESNSFGRNIMFDQAAKQV
tr|A0A1L6LSX5|A0A1L6LSX5_9DELT  EKGAP...FGR..RAPKKGKGAAEQ...........LTTLALEKNPVGRRLLFKKAREEL
tr|A0A2W4L9B9|A0A2W4L9B9_9PROT  E..GR...PPRPGRGEGFRPPTAAE...........LRELALERNPIGRRILFSQARKQA
tr|A0A2W4M4E6|A0A2W4M4E6_9PROT  ...GD...RRRGAR........LDK..RA.......LQEAMLAKNPIGRKIVFEQARRQT
tr|A0A6I2GRX9|A0A6I2GRX9_9DELT  ...AD...GTR.VP.........ERGTAPAKDFGARLRELALEDNPVGRRVLFEQAKKQV
tr|A0A0H4WMK2|A0A0H4WMK2_9DELT  G..GK....AK..GLAGFLQGLANK..EL.......WAEVALEDNPLGRKVLFDQARKQL
tr|F8CJ36|F8CJ36_MYXFH          G..GK....SK..GLAGFIQGLANK..EL.......WAEVALEDNPLGRKVLFDQARKQL
tr|A0A250K0F1|A0A250K0F1_9DELT  G..GK....SK..GLAGFIQGLANK..EL.......WAEVALEDNPLGRKVLFDQARKQL
tr|A0A7Y6WFZ2|A0A7Y6WFZ2_9DELT  S..GK....AK..GLAGFIQGLANK..EL.......WAEVALEDNPLGRKVLFDQARKQL
tr|A0A7Y7C660|A0A7Y7C660_9DELT  S..GK....AK..GLAGFIQGLANK..EL.......WAEVALEDNPLGRKVLFDQARKQL
tr|A0A7Y4JFH2|A0A7Y4JFH2_MYXXA  N..GK....AK..GLAGFIQGLANK..EL.......WAEVALEDNPLGRKVLFDQARKQL
tr|A0A4Y6CZQ9|A0A4Y6CZQ9_MYXXA  N..GK....AK..GLAGFIQGLANK..EL.......WAEVALEDNPLGRKVLFDQARKQL
```

```
tr|A0A7Y4IKV5|A0A7Y4IKV5_MYXXA  N..GK....AK..GLAGFIQGLANK..EL.......WAEVALEDNPLGRKVLFDQARKQL
tr|A0A511HHB0|A0A511HHB0_9DELT  N..GK....AK..GLAGFIQGLANK..EL.......WAEVALEDNPLGRKVLFDQARKQL
tr|A0A4Y6CKY7|A0A4Y6CKY7_MYXXA  N..GK....AK..GLAGFIQGLANK..EL.......WAEVALEDNPLGRKVLFDQARKQL
tr|Q1D1F2|Q1D1F2_MYXXD          N..GK....AK..GLAGFIQGLANK..EL.......WAEVALEDNPLGRKVLFDQARKQL
tr|A0A7Y4MA14|A0A7Y4MA14_MYXXA  N..GK....AK..GLAGFIQGLANK..EL.......WAEVALEDNPLGRKVLFDQARKQL
tr|A0A7T8Y4N9|A0A7T8Y4N9_MYXXA  N..GK....VK..GLAGFIQGLANK..EL.......WAEVALEDNPLGRKVLFDQARKQL
tr|L7UE67|L7UE67_MYXSD          ...AG...GPK..GLAGFFQGLANK..EL.......WAEVALEDNPLGRKLLFDQARNQL
tr|A0A511T9X1|A0A511T9X1_MYXFU  ...GG...QKK..GLAGFFQGLANK..EL.......WAEVALEDNPLGRKVLFDAARKQL
tr|A0A7Y7C9C4|A0A7Y7C9C4_9DELT  ...GQ....KK..GVAGFFQGLANK..EL.......WAEVALEDNPLGRKVLFDQARKQL
tr|A0A540X7W8|A0A540X7W8_9DELT  ...GQ....KK..GVAGFFQGLANK..EL.......WAEVALEDNPLGRKVLFDQARKQL
tr|A0A3A5FK19|A0A3A5FK19_9DELT  N..GK....AK..GLAGFLKGLASK..DL.......WTEVALEDNPLGRKVLFDQARKQL
tr|A0A3A8JQL9|A0A3A8JQL9_9DELT  S..GK....KK..GLAGFFQGLISK..DL.......WAEAALEDNPLGRKVLFDQAKKAL
tr|A0A7Y4NFA4|A0A7Y4NFA4_9DELT  S..GK....KK..GLAGLFQGLISK..DL.......WAEAALEDNPLGRKVLFDQAKKAL
tr|A0A3A8HDQ7|A0A3A8HDQ7_9DELT  S..SK....KK..GLAGLFQGLISK..DL.......WAEAALEDNPLGRKVLFDQAKKAL
tr|A0A3A8GR90|A0A3A8GR90_9DELT  S..SK....KK..GLAGLFQGLISK.........DLWTEAALEDNPLGRKVMFDQAKKQL
tr|A0A3A8SBD7|A0A3A8SBD7_9DELT  S..GK....KK..GIAGLFQGLINK..DL.......WKEAALEDNPLGRKVMFDQAKKQL
tr|A0A3A8T0I8|A0A3A8T0I8_9DELT  S..GK....KK..GIAGLFQGLINK..DL.......WKEAALEDNPLGRKVMFDQAKKQL
tr|A0A7X5BU07|A0A7X5BU07_9DELT  S..SK....KK..GIAGLFQGLINK..DL.......WKEAALEDNPLGRKVMFDQAKKQL
tr|A0A7Y1RVL2|A0A7Y1RVL2_9DELT  S..SK....KK..GIAGLFQGLINK..DL.......WKEAALEDNPLGRKVMFDQAKKQL
tr|A0A3A8THN2|A0A3A8THN2_9DELT  S..SK....KK..GIAGLFQGLINK..DL.......WKEAALEDNPLGRKVMFDQAKKQL
tr|A0A7Y1RX76|A0A7Y1RX76_9DELT  S..SK....KK..GIAGLFQGLINK..DL.......WKEAALEDNPLGRKVMFDQAKKQL
tr|A0A3A8RA58|A0A3A8RA58_9DELT  S..GK....KK..GIAGIFQGLINK..DL.......WTEAALEDNPLGRKVMFDQAKKQL
tr|A0A410RPB6|A0A410RPB6_CORCK  S..GK....KK..GIAGLFQGLINK..DL.......WKEAALEDNPLGRKVMFDQAKKQL
tr|A0A7Y4J474|A0A7Y4J474_CORCK  S..GK....KK..GIAGLFQGLINK..DL.......WKEAALEDNPLGRKVMFDQAKKQL
tr|A0A3A8I9Z6|A0A3A8I9Z6_9DELT  S..GK....KK..GIAGLFQGLINK..DL.......WKEAALEDNPLGRKVMFDQAKKQL
tr|H8MKE9|H8MKE9_CORCM          S..GK....KK..GIAGIFQGLINK..DL.......WKEAALEDNPLGRKVMFDQAKKQL
tr|A0A3A8H102|A0A3A8H102_9DELT  S..GK....KK..GIAGIFQGLINK..DL.......WKEAALEDNPLGRKVMFDQAKKQL
tr|A0A554FW33|A0A554FW33_9DELT  S..GK....KR..GLAGFFQGLISK..EL.......WTEAALEDNPLGRKVLFDQAKKAL
tr|A0A3A8NPC0|A0A3A8NPC0_9DELT  S..GK....TK..GLAGFFQGLISK..DL.......WAEAALEDNPIGRKVLFDQAKKAL
tr|A0A3A8JUV3|A0A3A8JUV3_9DELT  S..GK....TK..GLAGFFQGLISK..DL.......WAEAALEDNPLGRKVLFDQAKKAL
tr|A0A3A8NEX6|A0A3A8NEX6_9DELT  S..GK....TK..GLAGFFQGLISK..DL.......WAEAALEDNPIGRKVLFDQAKKAL
tr|A0A3A8JEU8|A0A3A8JEU8_9DELT  S..GK....KK..GLAGIFQGLISK..DL.......WAEAALEDNPLGRKVLFDQAKKAL
tr|A0A3A8LI76|A0A3A8LI76_9DELT  S..GK....KK..GIAGLFQGLISK.........DLWAEAALEDNPLGRKVLFDQAKKAL
tr|A0A085WXN8|A0A085WXN8_9DELT  Q.......SKK..GLAGMLQRLADK..AL.......WSEVALEANPLGRKLLFDQARKLL
tr|A0A2T4V0M5|A0A2T4V0M5_9DELT  Q..GK.....K..GLGGLLLGLTNK..EM.......WTEVALEDNPVGRKILFDQARKQL
tr|A0A0G2ZSW5|A0A0G2ZSW5_9DELT  Q..GK.....K..GLGGLLLGLTNK..EM.......WTEVALEDNPVGRKILFDQARKQL
tr|A0A3M2DKY0|A0A3M2DKY0_9DELT  A..PE....PD..R..GALARVRDA..VRHLLSTEDLQALALEDNPVGRKLLFDQARKKV
tr|A0A661NQ58|A0A661NQ58_9DELT  DEQGGHMHLPD..A.........DE...........LQELALTKNPLGRKVLFDQAEKQL
tr|A0A520YD99|A0A520YD99_9DELT  ...VG...YVP..PRESFIEQLRDK..DS.......VTEFALSKNPVGRKILFDQARKQL
tr|A0A7Y3BRE4|A0A7Y3BRE4_9DELT  V..GY...VPP..R.ESFIEQLRDK..DS.......VTEFALSKNPVGRKILFDQARKQL
tr|A0A2D9TF90|A0A2D9TF90_9DELT  ...AG...EGH.LEEEGVFERFDGS..DA.......LKELAMARNPVGRKVVFDAARKQL
tr|A0A2E0TP32|A0A2E0TP32_9DELT  E..EG...VPK..REVGLLEQLQDS..SE.......LTELALTKNPLGRKVLFDQARKKL
tr|A0A2E4Y3V1|A0A2E4Y3V1_9PROT  SGTMK...LKR.WK.........AK...........GKDKVLEDNFAGRALVFNQAKSMV
tr|A0A2E6VRH4|A0A2E6VRH4_9DELT  S..GQLKTRKR..E.............PT.......AQQKALEDTPPGRALLFRQARSMV
tr|A0A1F9FB59|A0A1F9FB59_9DELT  ELSRQ...EPK..K..........KLPER.......AAEAALEDNPAGRAILFRQARQMV
```

```
ECHA_HUMAN
                                                    300       310       320       330       340       350
ECHA_HUMAN                              RKQTKGLYPAPLKIIDVVKTGIEQGSDAGYLCESQKFGELVMTKESKALMGLYHGQVLCK
tr|A0A4R6XLA8|A0A4R6XLA8_9GAMM          MRKTRGNYPAPLKILDCVETGLNHGMDAGLEAEAMGFGELHASQASKSLIGLFLAMQENK
tr|A0A6A0IJT2|A0A6A0IJT2_9BACT          LRKTRGLYPAPLHILSCVEEGLSRGNEAGQERESALFGELVASPGAKNLIRLFDAMTALK
tr|A0A7V3QZU7|A0A7V3QZU7_9BACT          LAKTRGLYPSPLSILDCVQTGYAKGPAAGYDRESVLFGQLVAGPECKALIGLFEATTELK
tr|A0A7V4SYJ6|A0A7V4SYJ6_9BACT          RAKTRGLYPAPFAILDCVEEGLKRGPEAGFRKEEALFGELLAGEAARSLLTLFLHMTELK
tr|A0A2A8D2N6|A0A2A8D2N6_9BACT          QKQARGNYPAPPKIIECVKTGMEQGLEEGLDTEARNFGELVFTPESRALVSLFFAKQKGD
tr|A0A2N1TGP0|A0A2N1TGP0_9SPIR          MRQTRGHYPAALAIIDSVAYGYRKGMKKGLLKEIQLLGDLVLSPVSGALRSLFFGMTALK
tr|A0A1G3QGW4|A0A1G3QGW4_9SPIR          MRQTRGLYPAPLEIIDSVEFGYKKGLGKGIERDIERFVRLVMSSEAKSLMNIFFGITNLK
tr|A0A1G3QTY3|A0A1G3QTY3_9SPIR          MRQSCGLYPAPLAIIDSVEYGYKKGLRKGIQADIERFVNLVISPEAKALMTLFFGTTDLK
tr|A0A7X9L359|A0A7X9L359_9DELT          LRQTMGLYPAALAIISSVEFGLKHGVAKGLENDIRIFGELVMDGKSKALRSLFDGMTALK
tr|A0A522CJY0|A0A522CJY0_9SPIR          MKQTHGLYPAPLSIIESVRRGYKVGVVKGMTEDVKRFGALAVSPQAKALMTLFFNMTDMK
tr|A0A2N1RRJ3|A0A2N1RRJ3_9SPIR          TRQTKGCYPAPYAVIDAVEYGLKRGKEAGLQREIELFGELVISPESRALQSLFTGMLDLK
tr|A0A2N2KFN7|A0A2N2KFN7_9DELT          ARQTYGCYPAPYAIIEAVEEGQKHGKAAGLKKEIELLAKLVVSSQSKALMSLFFGMADMK
tr|A0A1V6AXX9|A0A1V6AXX9_9DELT          TRQTYGCYPAPYAIIEAVEHGQKHGKTAGLKKEIELFGRLVTTAQSKALMSLFFGMTDLK
tr|A0A2N2HZL0|A0A2N2HZL0_9DELT          ERQTYGCYPAPFAIIEAVEHGQKHGKAAGLKKEIEIFSRLVTTSQSKALMSLFFGMSDLK
tr|A0A5E8ARP3|A0A5E8ARP3_9BACT          LRQTSGNYPAPYKILESVKYGLSHTREQAAEKETELFDQLLQSPEAFQLINIFFAMNALR
tr|A0A662A7B1|A0A662A7B1_9BACT          QRQTYGNYPAPIKIMESVKFGLSHTLDKGIENEVTLFQELLRTPQAKQLINIFLGMTSLK
tr|D7CV16|D7CV16_TRURR                  QKKTRGNLPAPVKIIETIRIGQEQGMQAGLEAEARNFAELVFSPESKALVHLFFAKNAAE
tr|A0A3C2AKX4|A0A3C2AKX4_9FLAO          DRQTNGNYPAPYEIMECVKVGMSSGLKKGYAEEVKRFEKLILTPESFQLRGIFFAMTEKK
tr|A0A3B8ZLN3|A0A3B8ZLN3_9PLAN          DRQTKGNYPAPYEILACVQTGSEKGAQAGYDEEVRRFEKLILTPESFQLRGIFFAMTEKK
tr|A0A345UGV8|A0A345UGV8_9BACT          MRQTRGNYPAPLRIIDSVKAGLTQSREKGFETERRLFSELAFTPESAALRSLFFGMQSVK
tr|A0A6C1P6A2|A0A6C1P6A2_9BACT          EAKTLGNYPAPPKIIEAVEKAVTASRRDGREFETRAFTSLVFTPQSRALVNLFFAMTDSK
tr|A0A3M1X9W8|A0A3M1X9W8_9BACT          ARQTMGNYPAPFKILECVEVGMSRGMKAGLEVEARNFDQLMRTPQCKALIQLFFEINSRK
tr|A0A354C796|A0A354C796_9DELT          RKQTWGNYPAPFKIIECVKAGLEKGLEAGIEEETKKFGELMVSPQSRELVQIFLSMTAMK
tr|A0A3M1NUM8|A0A3M1NUM8_9BACT          QKQTQGNYPAPFKIIDCVEIGMEQGMKAGLDAEARGFEELAHTPQAEELINLFFSMTHQK
tr|A0A3M2KRC8|A0A3M2KRC8_9BACT          ARQTQGNYPAPFKIIECVEIGLEKGFEAGLDAEARGFEELARTPQAEELIRLFFAVNDKR
tr|A0A5S9IHR0|A0A5S9IHR0_9BACT          MRQTYGNYPAPLKIIDCVKRGMGTNLKSGLQIESKNFGELVVSPESTQLVNLFFAMTDSK
tr|A0A6M1ST81|A0A6M1ST81_9BACT          QGNTKGNYPAPFKIIDCIEEGYKNGFKAGLEKESIGFGELAATKESKALVNLFFSMQGAK
tr|A0A521AAE5|A0A521AAE5_9BACT          RSKTRGNYPAPPKIIDCVEEGYENGMRGGLELESRLFGELAATPESKAMVNLFFAMQAAK
tr|A0A1M4ZMD6|A0A1M4ZMD6_9BACT          RSKTKGNYPAPPRIIDCVEEGYENGMRAGLNLESRNFGWLAATPESQAMVNLFFAMQGAK
tr|A0A2A2GCS2|A0A2A2GCS2_9BACT          KSESKGNYPAPEKIIDCVQEGYENGMQAGLQLESEYFGKLAATPESEAMVSLFFAMQNAK
tr|A0A5D3YIH7|A0A5D3YIH7_9BACT          QSNTKGNYPAPEKIIDSVEEGYENGFQAGLELESVNFGKLAATPESEALVNMFFAMQGAK
tr|A0A6M1T9G0|A0A6M1T9G0_9BACT          KSKTKGNYPAPDKIIDCVKEGYENGMEAGLELESVNFGKLAATPESEALVNLFFAMQGAK
tr|A0A6A8Q1Y8|A0A6A8Q1Y8_9BACT          AGQTKGNYPAPPKIIDAVEYGLKNGIEKGLENESRLFGELGATRESRNLVNLFFGMNASR
tr|A0A521BVJ3|A0A521BVJ3_9BACT          AGRTRGNYPAPPKIIDAVEYGYKHGLEKGLENETVLFGELGATQESRNLVNLFFGMNASK
tr|A0A359E0Y8|A0A359E0Y8_9BACT          AGQTKGNYPAPPKIIDAVEYGYKNGIDKGLENETVLFGELGATQESRNLVNLFFGMNASK
tr|A0A2D8CIM6|A0A2D8CIM6_9BACT          AGQTKGNYPAPPKIIDAVEYGYKNGIDKGLENETVLFGELGATQESRNLVNLFFGMNASK
tr|A0A2D9FWF5|A0A2D9FWF5_9BACT          LSKTRGNYPAHPKILDAVKYGYKNGLEKGLEKETVLFGELGATQESRNLVNLFFGMNASK
tr|A0A1B6YB70|A0A1B6YB70_9BACT          LAQTKGNYPAPEKILDAVEYGYKHGKEKGLLNEAKLFGELGATQESRALVNLFFAMNNAK
tr|A0A2D4ZTC0|A0A2D4ZTC0_9BACT          LAQTKGNYPAPEKILDTVEFGYKYGKEKGLINEAKLFGELGATKESRALVNLFFAMTNAK
tr|A0A3D4UVW7|A0A3D4UVW7_9BACT          LAQTKGNYPAPEKILDTVEFGYKYGKEKGLINEAKLFGELGATKESRALVNLFFAMTNAK
tr|A0A3F3I3M2|A0A3F3I3M2_9BACT          LAQTKGNYPAPEKILDTVEFGYKNGKEKGLINEAKLFGELGATKESRALVNLFFAMTNAK
tr|A0A3D1G7L9|A0A3D1G7L9_9BACT          MGQTKGNYPAPLYIIDSVEFGYKNGKKKGLEHEAVLFGKAGATSESRALVNLFFGITDAK
tr|A0A3M8G1Y8|A0A3M8G1Y8_9BACT          LAQTKGNYPAPLLIIDSVKYGYQKGLEKGLENEARLFGEAGATFESKALVNLFFAMTDAK
tr|A0A5Q4F391|A0A5Q4F391_9BACT          LAQTKGNYPAAMKIIDSVEYRYKHGFVKGMDNESKLFGELAVSPESRALVQLFFGMTHAK
tr|A0A6I7NPV0|A0A6I7NPV0_9BACT          LSETKGNYPAPLKIIESVKFGYDHGLEKGIENEAKLFGELAVTPESRALVQLFFGIMAAK
tr|A0A651G1J2|A0A651G1J2_9BACT          LGQTKGNYPAPLKIIESVDYGYKHGFEKGIKNESKLFGELAVSPESRELVNLFFGITAAK
tr|A0A371QRK5|A0A371QRK5_9BACT          IGKTRGNYPAPFKIIESVEYGYKNGFEKGLKNESKLFGELAVTPESRALVQLFFAMNRAK
tr|A0A2N0VGJ5|A0A2N0VGJ5_9BACT          HGQTKGNYPAPPKIIDSVEFGFKNGLEKGLKNESRLFGELAVTPESRALVQLFFAMNDAK
tr|A0A316TYJ0|A0A316TYJ0_9BACT          MKETRGNYPAPPKIIESVKYGFKNGLDAGLKNESLLFGELAVTPESRALVNLFFGMNAAK
tr|A0A651GLZ8|A0A651GLZ8_9BACT          MGRTKGNYPAPPKIIESVKYGYKHGIKKGLRNESKLFGELAVTPESRALVQLFFGMNGAK
tr|A0A7Y5V3S5|A0A7Y5V3S5_9BACT          LTRTKGNYPAPLAAIDAVAAGF.HGREHGFREEARRFGEMAMTPVCRELIFLFFATAALK
tr|A0A7W1SHC0|A0A7W1SHC0_9BACT          LAKTHGHYPAPLAALDVVRAGYDRGMRQGLEEEARQFGELTQTPEAKELMFLFFATTSLK
tr|A0A7Y5PBT7|A0A7Y5PBT7_9BACT          EEKTHGHYPAPFAALQAIQAGYEKGHDAGYQAEARAFGEMAVSEESKQLVFLFFATTALK
tr|A0A7Y5TXL8|A0A7Y5TXL8_9BACT          EEKTHGHYPAPFAALQAIQAGYEKGHDAGYQAEARAFGEMAVSEESKQLVFLFFATTALK
tr|A0A423PQ96|A0A423PQ96_9GAMM          RSKTRGNYPAPERIIDVVATGFARGVQAGFDAEAKAFGELAMTPESQSLRHVYHATEALK
tr|U2G066|U2G066_9GAMM                  RAKTHGHYPAPERIIGVVATGYAQGVKAGFAVEAKAFGELAMGPVSKALRHVYHATESLK
tr|A0A2E0J1F4|A0A2E0J1F4_9GAMM          QSKTKGHYPAPERIIDVVATGYAKGMKAGYAAEAKAFGDLAMSEVSKSLRHVYHATEALK
tr|A0A2D4SCM8|A0A2D4SCM8_9GAMM          YKQTRGNYPAPEKIIDCVAEGAARGMKAGLAMEAKAFGQLVFTPQCKALRHVYRATEDLK
tr|L0WJH1|L0WJH1_9GAMM                  AKKTHGLYPAPPAIIDAVEAGYENGLAQGYATEARLFGELAMSSQSKQLRGIFHATNTLK
tr|A0A1H5XF61|A0A1H5XF61_9GAMM          AKKTHGLYPAPPAIIDAVEAGFEDGIEQGYATEARLFGELAMSPQSKQLRGIFHATNTLK
tr|A0A2E9TS48|A0A2E9TS48_9GAMM          AKKTHGLYPAPPAIIDAVEAGFEEGIEQGYATEARLFGELAMSPQSKQLRGIFHATNTLK
tr|A0A095UE19|A0A095UE19_9GAMM          AKKTHGLYPAPPAIIDAVEAGFEEGIEQGYATEARLFGELAMSPQSKQLRGIFHATNTLK
tr|A0A7G2S9A0|A0A7G2S9A0_9GAMM          AKKTHGLYPAPPAIIDAVEAGFEEGIEQGYATEARLFGELAMSPQSKQLRGIFHATNTLK
tr|B5JU27|B5JU27_9GAMM                  AKKTYGNYPAPERIIGAVEKGFSHGIEAGYEEEAQAFGDLAMSDVSKQLRGIFHATNALK
tr|A0A1Y0IHP1|A0A1Y0IHP1_9GAMM          AGKTLGNYPAPEKIIRAVAAGYRSGLAAGYEAEAMAFGELAMSAVSGQLRNIFHATNELK
tr|A0A316FZ14|A0A316FZ14_9GAMM          KGKTKGNYPAPFKIIECIEMGMEHGFEKGLEIEARYFGELVVTPVARQLMNIFFATTELK
tr|A0A498C231|A0A498C231_9GAMM          QAKTHGNYPAPPRIIDAVRAGRERGRQVGLETEGRHFGELAMTPEAQALMGLYFATTALK
tr|Q0A6T4|Q0A6T4_ALKEH                  QAKTHGNYPAPPRIIDAVRAGRERGRQAGLETERRHFGELAMTPEARALMALYFATTALK
tr|A0A3E0WL10|A0A3E0WL10_9GAMM          LAKTKGNYPAPKRIIEVVEIGVNRGFEEGLKAEARSFGELAMTPEMRELTRLYFATTEMK
tr|A0A3S1BU16|A0A3S1BU16_9GAMM          LGKTRGHYPAPLRIIDCVETGVSQGFRKGLAAEALGFGELAVTPQARQLMNLYFATTAMK
tr|A0A7V8QFN9|A0A7V8QFN9_9GAMM          LAQTKGNYPAPKRILEVVEAGLSQGMRKGLELEAKAFGELAMTPEARQLMGLYFATTAMK
tr|A0A1H8PPF9|A0A1H8PPF9_9GAMM          QSKTRGNYPALPRIIECIETGINRGMKSGLELEAKRFGELAMTPQARQLMNIYFATTAMK
tr|A0A6H0J0V7|A0A6H0J0V7_9GAMM          QKRTRGNYPALPRIIECVEAGIHQGVEKGLELEAKRFGELAMTPQARQLMSIYFATTAMK
tr|V5EZX6|V5EZX6_9VIBR                  QQKTRGNYPATDKILEVMQFGLEKGLKRGLEKEALEFGHLVMTYESKALRSLFFATTEMK
tr|A0A6I1QBM2|A0A6I1QBM2_9VIBR          QQKTRGNYPATDKILEVMQFGLEKGLKRGLEKEALEFGHLVMTYESKALRSLFFATTEMK
tr|A0A511QRL5|A0A511QRL5_9VIBR          KQKTRGNYPAADKILEVMQFGLEKGFAKGLEKEALEFGKLVMTSESKALRSLFFATTEMK
tr|A0A7Y0JZP4|A0A7Y0JZP4_9VIBR          NQKTRGNYPAADKILEVMQFGLEKGFAKGLEKEALEFGKLVMTSESKALRSLFFATTEMK
tr|A0A193KD24|A0A193KD24_9VIBR          KQKTRGNYPAADKILEVMQFGLEKGFAKGLEKEALEFGKLVMTSESKALRSLFFATTEMK
tr|A0A1S1HM72|A0A1S1HM72_PROST          MRKTKGHYPAPEKIIQVVRTGVEKGAKAGYDAEAKAFGELVMTPQSAALRSLFFATTSLK
tr|A0A7T8I613|A0A7T8I613_9GAMM          MRKTKGHYPAPEKIIQVVRTGVEKGAKAGYDAEAKAFGELVMTPQSAALRSLFFATTSLK
tr|A0A140NIF0|A0A140NIF0_PROSM          MRKTKGHYPAPEKIIQVVKTGVEKGAKAGYEAEAKAFGELVMTPESAALRSLFFATTALK
tr|A0A379GMB7|A0A379GMB7_PROST          MRKTKGHYPAPEKIIQVVKTGVEKGAKAGYEAEAKAFGELVMTPESAALRSLFFATTALK
tr|A0A379H393|A0A379H393_PROST          MRKTKGHYPAPEKIIQVVKTGVEKGAKAGYEAEAKAFGELVMTPESAALRSLFFATTALK
tr|B2Q0L5|B2Q0L5_PROST                  MRKTKGHYPAPEKIIQVVKTGVEKGAKAGYEAEAKAFGELVMTPESAALRSLFFATTALK
tr|A0A6I3JUE9|A0A6I3JUE9_9GAMM          MRKTKGHYPAPEKIIQVVKTGVEKGAKAGYEAEAKAFGELVMTPESAALRSLFFATTSLK
tr|A0A7D4P594|A0A7D4P594_YERMW          LAKTKGHYPAALRIIDVVRKGLDHGSASGYEAEASAFGELAMSPQSAALRSLFFATTSLK
tr|A0A7U7IVK6|A0A7U7IVK6_YEREN          QAKTKGHYPATERIIDVVRKGLDHGGPAGYEAEAKAFGELAMTPESAALRSLFFATTSLK
tr|A0A447RIF5|A0A447RIF5_YEREN          QAKTKGHYPATERIIDVVRKGLDHGGPAGYEAEAKAFGELAMTPESAALRSLFFATTSLK
tr|A0A7H4ZLV9|A0A7H4ZLV9_YERP4          QAKTKGHYPATERIIDVVRKGLDHGGPAGYEAEAKAFGELAMTPESAALRSLFFATTSLK
tr|A0A0H3NX46|A0A0H3NX46_YERE1          QAKTKGHYPATERIIDVVRKGLDHGGPAGYEAEAKAFGELAMTPESAALRSLFFATTSLK
tr|A0A7T9XUV7|A0A7T9XUV7_YEREN          QAKTKGHYPATERIIDVVRKGLDHGGPAGYEAEAKAFGELAMTPESAALRSLFFATTSLK
tr|A0A7U7IYV8|A0A7U7IYV8_YEREN          QAKTKGHYPATERIIDVVRKGLDHGGPAGYEAEAKAFGELAMTPESAALRSLFFATTSLK
tr|A0A0H5G7G2|A0A0H5G7G2_YEREN          QAKTKGHYPATERIIDVVRKGLDLGGPAGYEAEAKAFGELAMTPESAALRSLFFATTSLK
tr|A0A2A7TDI2|A0A2A7TDI2_YERKR          LAKTKGHYPAAERIIDVVRKGLDHGGPAGYEAEAKAFGELAMSPESAALRSLFFATTALK
tr|A0A0T9M261|A0A0T9M261_YERKR          LAKTKGHYPAAERIIDVVRKGLDHGGPAGYEAEAKAFGELAMSPESAALRSLFFATTALK
tr|A0A0A0CMP1|A0A0A0CMP1_PHOLU          LAKTRGHYPAPERIIDVVKEGLEKGMSQGLRAEAVAFGELAMTRESAALRNLFFAATSLK
tr|A0A329VG55|A0A329VG55_9GAMM          LAKTRGHYPAPERIIEVVKEGLEKGMSQGLRAEAVAFGELAMTRESAALRNLFFAATSLK
sp|Q7N288|FADJ_PHOLL                    LAKTRGHYPAPERIIEVVKEGLEKGMSQGLRAEAVAFGELAMTRESAALRNLFFAATSLK
tr|A0A6L9JMX5|A0A6L9JMX5_PHOLM          LAKTRGHYPAPERIIEVVKEGLEKGMSQGLRAEAVAFGELAMTRESAALRNLFFAATSLK
tr|A0A7X5HQ29|A0A7X5HQ29_PHOLM          LAKTRGHYPAPERIIEVVKEGLEKGMSQGLRAEAVAFGELAMTRESAALRNLFFAATSLK
tr|A0A1C0U051|A0A1C0U051_9GAMM          LAKTRGHYPAPERIIDVVKEGLEKGMNQGLRAEAMAFGELAMTRESAALRNLFFAATSLK
tr|A0A7X5QF62|A0A7X5QF62_9GAMM          LAKTRGHYPAPERIIEVVKEGLEKGMNQGLRAEAMAFGELAMTRESAALRNLFFAATSLK
```

```
tr|A0A2D0KWM9|A0A2D0KWM9_9GAMM  RAKTHGHYPAPERIIDVIRLGLEKGQKAGLQAESKAFGELAMSSESSALRSLFFATTSLK
tr|A0A2D0LAI1|A0A2D0LAI1_9GAMM  RAKTHGHYPAPERIIDVIRLGLEKGQKAGLQAESKAFGELAMSSESSALRSLFFATTSLK
tr|A0A2D0IXC8|A0A2D0IXC8_XENBU  RAKTHGHYPAPERIIDVVRTGLEKGQKTGFQAEAKAFGELAMSSESEALRSLFFAATSLK
tr|W1JAM5|W1JAM5_9GAMM          RAKTHGHYPAPERIIDVVRTGLEKGQKTGFRAEAKAFGELAMSPESEALRSLFFAATSLK
tr|A0A3D9UED3|A0A3D9UED3_9GAMM  RAKTHGHYPAPERIIDVVRTGLEKGQKTGFRAEAKAFGELAMSPESEALRSLFFAATSLK
tr|A0A1I3JCA9|A0A1I3JCA9_9GAMM  REKTHGHYPAPEKIINVIRTGLEKGLDKGFQAEARAFGELVMTQESAALRSLFFASTALK
tr|A0A068QUL9|A0A068QUL9_9GAMM  REKTRGHYPAPEKIINVIRAGLEKGTEKGFQLEAKAFGELAMTPESEALRSLFFASTALK
tr|A0A0M0TCH6|A0A0M0TCH6_9GAMM  REKTRGHYPAPEKIINVIRAGLEKGSEKGFQLEARAFGELAMTQESEALRSLFFASTTLK
tr|A0A1Q5U854|A0A1Q5U854_9GAMM  REKTRGHYPAPEKIINVICAGLEKGSEKGFQLEAKAFGELAMTQESEALRNLFFASTTLK
tr|A0A2D0ISP1|A0A2D0ISP1_9GAMM  REKTRGHYPAPEKIINVICAGLEKGSEKGFQLEAKAFGELAMTQESEALRSLFFASTTLK
tr|A0A1Q5TUI8|A0A1Q5TUI8_9GAMM  REKTRGHYPAPEKIINVICAGLEKGSEKGFQLEAKAFGELAMTQESEALRSLFFASTTLK
tr|A0A2D0KJI7|A0A2D0KJI7_9GAMM  REKTRGHYPAPEKIINVICAGLEKGSEKGFQLEAKAFGELAMTQESEALRSLFFASTTLK
tr|A0A1Y2SB97|A0A1Y2SB97_9GAMM  REKTRGHYPAPEKIINVIRTGLEKGVERGFQLEAKAFGELAMSQESEALRSLFFASTTLK
tr|A0A1I5DWI7|A0A1I5DWI7_9GAMM  REKTRGHYPAPEKIINVIRTGLEKGAERGFQLEAKAFGELAMSQESEALRSLFFASTTLK
tr|A0A1I7GWY6|A0A1I7GWY6_9GAMM  QAKTHGHYPAPEKIINVIRTGLEKGAIHGFKAEARVFGELAMSPESAALRSLFFASTTLK
tr|D3VKY8|D3VKY8_XENNA          QAKTHGHYPAPEKIINVIRTGLEKGAIHGFKTEAKAFGELAMSKESEALRSLFFASTTLK
tr|A0A2G0Q6Q9|A0A2G0Q6Q9_9GAMM  RAKTHGHYPAPEKIINVIRTGLEKGLDKGFQAEARAFGELAMSQESEALRGLFFASTALK
tr|A0A2D0JU24|A0A2D0JU24_9GAMM  RAKTHGHYPAPEKIINVIRTGLEKGPDKGFKAEAKAFGELAMSPESAALRSLFFASTALK
tr|A0A0J5FN38|A0A0J5FN38_9GAMM  RAKTHGHYPAPEKIINVIRTGLEKGLDKGFKAEAKAFGELAMSPESAALRSLFFASTTLK
tr|A0A432XLD4|A0A432XLD4_9GAMM  QAKAKGNYPALDKIIDTIAYGADKGFKAGLEFEARSFGELAMTPESKQLRNIFFATTAMK
tr|A0A2D8HU84|A0A2D8HU84_9GAMM  QKKAHGNYPAIDKIIQTVREGVERGREAGLDKEARSFGELAMTPESYQLRQIFFATTEMK
tr|A0A656X1Q2|A0A656X1Q2_9GAMM  QKKAHGNYPAIDKIIQTVREGVERGREAGLDKEARSFGELAMTPESYQLRQIFFATTEMK
tr|A0A4Q1QH79|A0A4Q1QH79_9GAMM  QKKAQGNYPAIDKIIQTVREGIERGQEAGLDKEARSFGELAMTPESYQLRQIFFATTEMK
tr|A0A1G7LQE6|A0A1G7LQE6_9GAMM  QKKAHGNYPAIDKIIQTVREGVERGHEAGLDKEARSFGELAMTPESYQLRQIFFATTEMK
tr|A0A432YVG7|A0A432YVG7_9GAMM  QKKAHGNYPAIDKIIQTVREGVERGHEAGLDKEARSFGELAMTPESYQLRQIFFATTEMK
tr|A0A1J5N0P5|A0A1J5N0P5_9GAMM  LAKTKGHYPAPLKIIDCVRKGLNESRQAGLAIEASHFADLVMTDESKALRQLFFATTEMK
tr|A0A0D8D548|A0A0D8D548_9GAMM  LAKTKGKYPAPLKIIDCVKTGSEQSPAQGYQVEANHFGELVKTPESAQLRNIFFATTDMK
tr|A0A3E0TNM2|A0A3E0TNM2_9GAMM  LAKTKGNYPAPLKIIDCVRTGVERSPAMGYQVEADHFGHLVMTPESEQLRNLFFATTEMK
tr|A0A3E0UD67|A0A3E0UD67_9GAMM  LAKTKGNYPAPLKIIDCVRTGAENSPAMGYQVEADHFGHLVMTPESEQLRNLFFATTEMK
tr|A0A3E0U0P4|A0A3E0U0P4_9GAMM  LAKTKGNYPAPLKIIDCVRTGAENSPAMGYQVEADHFGHLVMTPESEQLRNLFFATTEMK
tr|A0A0M2V8B1|A0A0M2V8B1_9GAMM  LKKTQGNYPAPVRILEVVRKGLDSGYAAGLAAEAKAFGQLCMTKESAALRSLFFATTQMK
tr|A0A285ITY5|A0A285ITY5_9GAMM  LKKTQGNYPAPVRIIEVVRKGQDSGYAAGLAAEAKAFGQLCMTKESAALRSLFFATTQMK
tr|A0A486XTB9|A0A486XTB9_9GAMM  LKKTQGNYPAPLKILDVIKAGVDNGMQAGLAAEAKAFGELCMTKESAALRSLFFATTQMK
tr|I1E1G1|I1E1G1_9GAMM          LSKTQGNYPAPLKILDVIKTGVDSGMQAGLAAEAKAFGELCMTKESAALRSLFFATTQMK
tr|A0A1H6KJ68|A0A1H6KJ68_9GAMM  LQKTQGNYPAPLKILDVIKTGVDSGMQAGLAAEAKAFGELCMTKESAALRSLFFATTQMK
tr|A0A0X3Y764|A0A0X3Y764_9GAMM  LQKTQGNYPAPLKILDVIKTGVDSGMQAGLAAEAKAFGELCMTKESAALRSLFFATTQMK
tr|A0A2N1YEV4|A0A2N1YEV4_9GAMM  LQKTQGNYPAPLKILDVIKTGVDSGMQAGLAAEAKAFGELCMTKESAALRSLFFATTQMK
tr|A0A3P3QCM3|A0A3P3QCM3_9GAMM  LAKTHGNYPAPLKIIKAIKAGVDGGFNKGLDVEAKAFGELCMTSESKALRSLFFATTQMK
tr|F7NT72|F7NT72_9GAMM          LAKTHGNYPAPLKILKAIKAGVDGGFNKGLDVEAKAFGELCMTSESKALRSLFFATTQMK
tr|A0A3S2TW63|A0A3S2TW63_9GAMM  LAKTQGNYPAPLKILKAIKAGVDGGFNKGLDVEAKAFGELCMTSESKALRSLFFATTQMK
tr|A0A3D5BGW7|A0A3D5BGW7_9GAMM  LLKTGGHYPAPLKIIEAIKAGVDSGMARGLQVEAQAFGELCMTSVSKSLRGLFFATTQMK
tr|A0A5C7TI17|A0A5C7TI17_9GAMM  LAKTQGHYPAPLKIIDAIKTGVFSGMQKGLEVEAKHFGELCMTPVSANLRNLFFATTQMK
tr|A0A0U4W982|A0A0U4W982_9GAMM  LAKTQGHYPAPLKIIDAIKAGVHSGMQKGLEVEATHFGELCMTPVSANLRNLFFATTQMK
tr|A0A2I0FCM2|A0A2I0FCM2_9GAMM  LSKTRGNYPAPEAILEVVKVGQEKGFAAGLKLEAKRFAELAMTSESACLRGIFFATTEMK
tr|A0A2G2IVL1|A0A2G2IVL1_9GAMM  LSKTRGNYPAPEAILEVVKLGQEKGFEAGLKLEAKRFAELAMTSESAALRGIFFATTXMK
tr|A6FI21|A6FI21_9GAMM          LSKTRGNYPAPEAILEVVKLGQEKGFEAGLKLEAKRFSELAMTSESAALRGIFFATTAMK
tr|A0A4U1BNR2|A0A4U1BNR2_9GAMM  MSKTKGNYPAPEKIIDCVKAS.SGDLESGLKLEAKHFSELVMSSESEALRSIFFATTEMK
tr|E1SQ60|E1SQ60_FERBD          LAKTQGNYPAPLKIIDCVRTGLEKGLDAGYEVEARHFGELVMTPESEALRSIFFATTEMK
tr|A0A4Y6IZ09|A0A4Y6IZ09_9GAMM  NKKTQGNYPAPEKIIDCVRQGMTKDMVKGLEVEAQHFGDLVVSSESEALRSIFFATTDMK
tr|A0A0C3QSZ7|A0A0C3QSZ7_9GAMM  NKKTQGNYPAPEKIIDCVRQGVTKGMVKGLEVEAQHFGDLVVSSESEALRSIFFATTEMK
tr|A0A6L7HXW0|A0A6L7HXW0_9GAMM  NKKTQGNYPAPEKIIDCVRQGVTKGMVKGLEVEAQHFGDLVASSESEALRSIFFATTEMK
sp|A3QFP3|FADJ_SHELP            NKKTQGNYPAPEKIIDCVRQGVTKGIVKGLEVEAQHFGDLVVSNESEALRSIFFATTEMK
tr|A0A1E5IXH7|A0A1E5IXH7_SHECO  NKKTHGNYPAPEKIIDCVRQGMTKGLIKGLEVEATHFADLVMSPESEALRSIFFATTEMK
tr|A0A411PKQ0|A0A411PKQ0_9GAMM  ESKTHGNYPAPAKIIDCVRQGLNKGLTKGLEVEASHFAELVMSKESEALRSIFFATTEMK
tr|A0A6G9QKM3|A0A6G9QKM3_9GAMM  AKKTAGNYPAPAKIIDCVRQGLNKGMTKGLEVEATHFANLVMSKESAALRSIFFATTEMK
tr|A0A6P1UL63|A0A6P1UL63_9GAMM  MKKTQGNYPAPAKIIDCVRQGMNKGMVKGLEVEATHFANLVMSKESAALRSLFFATTEMK
tr|A0A2N1ERI9|A0A2N1ERI9_9GAMM  MKKTQGNYPAPAKIIDCVRQGMNKGMVKGLEVEATHFANLVMSKESAALRSLFFATTEMK
tr|A0A7W4FU55|A0A7W4FU55_9GAMM  MKKTQGNYPAPAKIIDCVRQGMNKGMVKGLEVEATHFANLVMSKESAALRSLFFATTEMK
sp|Q8ECP7|FADJ_SHEON            AKKTQGNYPAPAKIIDCVRQGIAKGMQKGLEVEASHFAELVVSKESEALRSIFFATTEMK
tr|A0A501XZY8|A0A501XZY8_9GAMM  AKKTQGNYPAPAKIIDCVRQGMAKGMQKGLEVEASHFAELVVSKESEALRSIFFATTEMK
tr|A0A2W5DCZ0|A0A2W5DCZ0_SHEOE  AKKTQGNYPAPAKIIDCVRQGMAKGMQKGLEVEASHFAELVVSKESEALRSIFFATTEMK
tr|A0A1E3V3C8|A0A1E3V3C8_9GAMM  AKKTQGNYPAPAKIIDCVRQGMAKGMQKGLEVEASHFAELVVSKESEALRSIFFATTEMK
tr|A0A1Z4AI20|A0A1Z4AI20_9GAMM  AKKTQGNYPAPAKIIDCVRQGMAKGMQKGLEVEASHFAELVVSKESEALRSIFFATTEMK
tr|A0A7X9LJL9|A0A7X9LJL9_9GAMM  AKKTQGNYPAPAKIIDCVRQGMAKGMQKGLEVEASHFAELVVSKESEALRSIFFATTEMK
tr|A0A073KMY5|A0A073KMY5_9GAMM  AKKTQGNYPAPAKIIDCVRQGMAKGMQKGLEVEASHFAELVVSKESEALRSIFFATTEMK
sp|A0KV76|FADJ_SHESA            VKKTQGNYPAPAKIIDCVRQGMAKGMQKGLEVEASHFAELVVSKESEALRSIFFATTEMK
tr|A0A220UTH7|A0A220UTH7_9GAMM  VKKTQGNYPAPAKIIDCVRQGMAKGMQKGLEVEASHFAELVVSKESEALRSIFFATTEMK
tr|A0A5B8R6W1|A0A5B8R6W1_9GAMM  AKKTQGNYPAPAKIIDCVRQGMAKGMQKGLEVEASHFAELVVSKESEALRSIFFATTEMK
tr|V1DAI4|V1DAI4_9GAMM          AKKTQGNYPAPAKIIDCVRQGMAKGMQKGLEVEASHFAELVVSKESEALRSIFFATTEMK
tr|A0A448CPQ4|A0A448CPQ4_SHEPU  AKKTQGNYPAPAKIIDCVRQGMAKGMQKGLEVEASHFAELVVSKESEALRSIFFATTEMK
tr|A0A252ERQ3|A0A252ERQ3_SHEPU  AKKTQGNYPAPAKIIDCVRQGMAKGMQKGLEVEASHFAELVVSKESEALRSIFFATTEMK
sp|Q0HKD1|FADJ_SHESM            AKKTQGNYPAPAKIIDCVRQGMAKGMQKGLEVEASHFAELVVSKESEALRSIFFATTEMK
sp|Q0HWN3|FADJ_SHESR            AKKTQGNYPAPAKIIDCVRQGMAKGMQKGLEVEASHFAELVVSKESEALRSIFFATTEMK
tr|F7RQE3|F7RQE3_9GAMM          EKKTQGNYPAPAKIIDCVRQGIAKGMQKGLEVEASHFAELVISKESEALRSIFFATTEMK
tr|B8EE98|B8EE98_SHEB2          EKKTQGNYPAPAKIIDCVRQGIAKGMQKGLEVEASHFAELVVSKESEALRSIFFATTEMK
sp|A6WQ25|FADJ_SHEB8            EKKTQGNYPAPAKIIDCVRQGIAKGMQKGLEVEASHFAELVVSKESEALRSIFFATTEMK
tr|A0A448EK41|A0A448EK41_9GAMM  EKKTQGNYPAPAKIIDCVRQGIAKGMQKGLEVEASHFAELVVSKESEALRSIFFATTEMK
tr|A0A553JHX1|A0A553JHX1_SHEHA  QKKTQGNFPAPDKIIDCVRQGMAKGMVKGLEVEASHFSELVLSKESAALRSIFFATTEMK
tr|B8CPY6|B8CPY6_SHEPW          AKKTQGNYPAPAKIIDCVRQGMTKGLIKGLEVEASHFSDLVISKESEALRSIFFATTEMK
tr|A0A431WFC4|A0A431WFC4_9GAMM  EKKTHGNYPAPAKIIDCVRQGMAKGLTKGLEVEANHFADLVVSKESEALRSIFFGTTEMK
tr|A8FTR7|A8FTR7_SHESH          EKKTHGNYPAPAKIIDCVRQGMAKGLTKGLEVEANHFADLVVSKESEALRSIFFGTTEMK
tr|A0A431WNL0|A0A431WNL0_9GAMM  EKKTHGNYPAPAKIIDCVRQGMARGLTKGLEVEANHFADLVVSKESEALRSIFFGTTEMK
tr|A0A550AEC5|A0A550AEC5_9GAMM  QKKTQGNYPAPAKIIDCVRQGMAKGKAKGLEVEASHFSELVMSTESAALRSIFFATTEMK
tr|A0A7L4WW90|A0A7L4WW90_9GAMM  QKKTQGNYPAPAKIIDCVRQGMAKGKAKGLEVEASHFSELVMSTESAALRSIFFATTEMK
tr|A9DDU3|A9DDU3_9GAMM          QQKTQGNYPAPAKIIDCVRQGMAKGKTAGLEVEASHFAELVMSTESAALRSIFFATTEMK
tr|A0A330M2B9|A0A330M2B9_9GAMM  QQKTQGNYPAPAKIIDCVRQGMAKGKTTGLEVEASHFAELVMSTESAALRSIFFATTEMK
tr|A0A5N8UFC7|A0A5N8UFC7_9GAMM  QKKTQGNYPAPAKIIDCVRQGMAKGKAKGLEVEASHFADLVMSTESAALRSIFFATTEMK
tr|A0A1S6HN57|A0A1S6HN57_9GAMM  QKKTQGNYPAPAKIIDSVRQGMAKGKTKGLEVEASHFAELVMSKESAALRSIFFATTEMK
tr|D4ZMH7|D4ZMH7_SHEVD          QKKTQGNYPAPAKIIDCVRQGMAKGKTKGLEVEASHFAELVMSKESAALRSIFFATTEMK
tr|A0A3L8Q213|A0A3L8Q213_9GAMM  HKKTQGNYPAPPKIIDCIKVGANKGMQAGLEAESRHFADLVLTKESAALRTIFFATTEMK
tr|A0A3A6U4N9|A0A3A6U4N9_9GAMM  QRKTQGNYPAPNKIIDCVRVGMSKGIHAGLEVEARHFSDLVMTKESAALRSIFFATTEMK
tr|A0A4Q5MA37|A0A4Q5MA37_9GAMM  NKKTQGNYPAPPKIIDCVKVGMSKGLVAGLDIEARHFSDLVMTKESAALRSIFFATTEMK
tr|A0A1L6LSX5|A0A1L6LSX5_9DELT  LKKTGGHYPAPERIVDVLQAFAEHGFEAAREVEAKHFGELVVSSVAHRLIEIFFAQTALK
tr|A0A2W4L9B9|A0A2W4L9B9_9PROT  LEKSRGHYPAIPAAIEAVETGIEQGYERGLAREAELFGELAVTDVSRNLVRLFFAQTALK
tr|A0A2W4M4E6|A0A2W4M4E6_9PROT  RKKTHGNYPAPERILDVVETGLAHGPMRGYDAETRAFGELAASPEARELINIFFATNELK
tr|A0A6I2GRX9|A0A6I2GRX9_9DELT  LQKTRGKYPAPLKALEAVRVGLESGREKGLAAEARLFGELVVSDVSRRLVEVFFATTALK
tr|A0A0H4WMK2|A0A0H4WMK2_9DELT  LKKTRGKFPAPEKALQVIRVGLESGHKAGQEAEAKAFGELVVSDVSKRLVEIFFATTALK
tr|F8CJ36|F8CJ36_MYXFH          LKKTRGKFPAPEKALQVVRVGLESGHKAGQEAEAKAFGELVVSDVSKRLVEIFFATTALK
tr|A0A250K0F1|A0A250K0F1_9DELT  LKKTRGKFPAPEKSLQVVRVGLESGHKAGQEAEAKAFGELVVSDVSRRLVEIFFATTALK
tr|A0A7Y6WFZ2|A0A7Y6WFZ2_9DELT  LKKTRGKFPAPEKALQVVRVGLESGHKAGQEAEAKAFGELVVSDVSKRLVEIFFATTALK
tr|A0A7Y7C660|A0A7Y7C660_9DELT  LKKTRGKFPAPEKALQVVRVGLESGHKAGQEAEAKAFGELVVSDVSKRLVEIFFATTALK
tr|A0A7Y4JFH2|A0A7Y4JFH2_MYXXA  LKKTRGKFPAPEKALQVVRVGLESGHKAGQEAEAKAFGELVVSDVSKRLVEIFFATTALK
tr|A0A4Y6CZQ9|A0A4Y6CZQ9_MYXXA  LKKTRGKFPAPEKALQVVRVGLESGHKAGQEAEAKAFGELVVSDVSRRLVEIFFATTALK
```

```
tr|A0A7Y4IKV5|A0A7Y4IKV5_MYXXA  LKKTRGKFPAPEKALQVVRVGLESGHKAGQEAEAKAFGELVVSDVSKRLVEIFFATTALK
tr|A0A511HHB0|A0A511HHB0_9DELT  LKKTRGKFPAPEKALQVVRVGLESGHKAGQEAEAKAFGELVVSDVSKRLVEIFFATTALK
tr|A0A4Y6CKY7|A0A4Y6CKY7_MYXXA  LKKTRGKFPAPEKALQVVRVGLESGHKAGQEAEAKAFGELVVSDVSKRLVEIFFATTALR
tr|Q1D1F2|Q1D1F2_MYXXD          LKKTRGKFPAPEKALQVVRVGLESGHKAGQEAEAKAFGELVVSDVSKRLVEIFFATTALK
tr|A0A7Y4MA14|A0A7Y4MA14_MYXXA  LKKTRGKFPAPEKALQVVRVGLESGHKAGQEAEAKAFGELVVSDVSKRLVEIFFATTALK
tr|A0A7T8Y4N9|A0A7T8Y4N9_MYXXA  LKKTRGKFPAPEKALQVVRVGLESGHKAGQEAEAKAFGELVVSDVSKRLVEIFFATTALK
tr|L7UE67|L7UE67_MYXSD          LKKTRGKYPAPEKALQVIRVGLESGRKAGLEAEARAFGELVMSDVSKRLVEIFFATTALK
tr|A0A511T9X1|A0A511T9X1_MYXFU  LKKTRGKYPAPEKALRVVRTGLESGRQAGLAAEAKAFGELVMSDVSKRLVEVFFATTALK
tr|A0A7Y7C9C4|A0A7Y7C9C4_9DELT  RKKTRGKYPAPEKALQVVRAGLESGRKAGLEAEAKAFGELVMSDVSKRLVEVFFATTALK
tr|A0A540X7W8|A0A540X7W8_9DELT  RKKTRGKYPAPEKALQVIRVGLESGRKAGLEAEAKAFGELVMSDVSKRLVEVFFATTALK
tr|A0A3A5FK19|A0A3A5FK19_9DELT  LKKTRGKYPAPEKALQVIRAGLESGRAAGLEAEAKAFGELVVSDVSRRLVEIFFATTALK
tr|A0A3A8JQL9|A0A3A8JQL9_9DELT  LKKTRGKYPAQEKALQVIRVGLESGRKAGLEAEAKAFGELVFTDVSRRLVEIFFATTALK
tr|A0A7Y4NFA4|A0A7Y4NFA4_9DELT  LKKTRGKYPAQEKVLQVIRVGLESGRKAGLEAEAKAFGELVFTDVSKRLVEIFFATTALK
tr|A0A3A8HDQ7|A0A3A8HDQ7_9DELT  LKKTRGKYPAQEKALQVIRVGLESGRKAGLEAEAKAFGELVFTDVSKRLVEIFFATTALK
tr|A0A3A8GR90|A0A3A8GR90_9DELT  LKKTRGKYPAQEKALQVIRVGLESGRKAGLEAEAKAFGELVFTDVSRRLVEIFFATTALK
tr|A0A3A8SBD7|A0A3A8SBD7_9DELT  LKKTRGKYPAQEKALQVIRVGLESGRKAGLEAEAKAFGELVFTDVSRRLVEIFFATTALK
tr|A0A3A8T0I8|A0A3A8T0I8_9DELT  LKKTRGKYPAQEKALQVIRVGLESGRKAGLEAEAKAFGELVFTDVSRRLVEIFFATTALK
tr|A0A7X5BU07|A0A7X5BU07_9DELT  LKKTRGKYPAQEKALQVIRVGLESGRKAGLEAEAKAFGELVFTDVSRRLVEIFFATTALK
tr|A0A7Y1RVL2|A0A7Y1RVL2_9DELT  LKKTRGKYPAQEKALQVIRVGLESGRKAGLEAEAKAFGELVFTDVSRRLVEIFFATTALK
tr|A0A3A8THN2|A0A3A8THN2_9DELT  LKKTRGKYPAQEKALQVIRVGLESGRKAGLEAEAKAFGELVFTDVSRRLVEIFFATTALK
tr|A0A7Y1RX76|A0A7Y1RX76_9DELT  LKKTRGKYPAQEKALQVIRVGLESGRKAGLEAEAKAFGELVFTDVSRRLVEIFFATTALK
tr|A0A3A8RA58|A0A3A8RA58_9DELT  LKKTRGKYPAQEKALQVIRVGLESGRKAGLEAEAKAFGELVFTDVSRRLVEIFFATTALK
tr|A0A410RPB6|A0A410RPB6_CORCK  LKKTRGKYPAQEKALQVIRVGLESGRKAGLEAEAKAFGELVFTDVSRRLVEIFFATTALK
tr|A0A7Y4J474|A0A7Y4J474_CORCK  LKKTRGKYPAQEKALQVIRVGLESGCKAGLEAEAKAFGELVFTDVSRRLVEIFFATTALK
tr|A0A3A8I9Z6|A0A3A8I9Z6_9DELT  LKKTRGKYPAQEKALQVIRVGLESGRKAGLEAEAKAFGELVFTDVSRRLVEIFFATTALK
tr|H8MKE9|H8MKE9_CORCM          LKKTRGKYPAQEKALQVIRVGLESGRKAGLDAEAKAFGELVFTDVSRRLVEIFFATTALK
tr|A0A3A8H102|A0A3A8H102_9DELT  LKKTRGKYPAQEKALQVIRVGLESGRKAGLDAEAKAFGELVFTDVSRRLVEIFFATTALK
tr|A0A554FW33|A0A554FW33_9DELT  LKKTRGKYPAQEKALQVIRVGLESGRKAGLEAEAKAFGELVFTDVSRRLVEIFFATTALK
tr|A0A3A8NPC0|A0A3A8NPC0_9DELT  LKKTRGKYPAQEKALQVIRVGLESGRKAGLEAEAKAFGELVFTDVSRRLVEIFFATTALK
tr|A0A3A8JUV3|A0A3A8JUV3_9DELT  LKKTRGKYPAQEKALQVIRVGLESGRKAGLEAEAKAFGELVFTDVSRRLVEIFFATTALK
tr|A0A3A8NEX6|A0A3A8NEX6_9DELT  LKKTRGKYPAQEKALQVIRVGLESGRKAGLEAEAKAFGELVFTDVSRRLVEIFFATTALK
tr|A0A3A8JEU8|A0A3A8JEU8_9DELT  LKKTRGKYPAQEKALQVIRVGLESGRKAGLEAEAKAFGELVFTDVSRRLVEIFFATTALK
tr|A0A3A8LI76|A0A3A8LI76_9DELT  LKKTRGKYPAQEKALQVIRVGLESGRKAGLEAEAKAFGELVFTDVSRRLVEIFFATTALK
tr|A0A085WXN8|A0A085WXN8_9DELT  LKKTRGKYPAPEKALEAIRVGVESGRKAGLETEARLFGELVVSDVSKRLVEIFFATTALK
tr|A0A2T4V0M5|A0A2T4V0M5_9DELT  RKKTRGKYPAPEKALEAIRIGVESGRAAGLEAEAKFFGELVVSDVSKQLVEIFFATTALK
tr|A0A0G2ZSW5|A0A0G2ZSW5_9DELT  RKKTRGKYPAPEKALEAIRVGVESGRAAGLEAEARFFGELVVSDVSKRLVEIFFATTALK
tr|A0A3M2DKY0|A0A3M2DKY0_9DELT  LAQTHGNYPAPLAILEVVKTGLDKGLDAGYEAEAKAFGRLVASPEAKQLMGIFFATRALE
tr|A0A661NQ58|A0A661NQ58_9DELT  RAKTRGNYPAPEKILEAVRTGLADGFDAGLEAERRLFGELTVSPEAAALMSIFFATTALK
tr|A0A520YD99|A0A520YD99_9DELT  HKQTRGNYPAQDLIIDIVKTGLEDGFEAGLAAEARGFGRLLTTPEAANLMSIFFATTALK
tr|A0A7Y3BRE4|A0A7Y3BRE4_9DELT  HKQTRGNYPAQDLIIDIVKTGLEDGFEAGLAAEARGFGRLLTTPEAANLMSIFFATTALK
tr|A0A2D9TF90|A0A2D9TF90_9DELT  HKKTRGNYPAQEKILDVVKVGLADGMRAGLEAEASAFGELVMSPQSEALRSIFFATQVMK
tr|A0A2E0TP32|A0A2E0TP32_9DELT  HEKTRGNYPAQDRILDVVKIGLEKGRAAGLEAEATHFGELVMSPESAALRSIYFATQAMK
tr|A0A2E4Y3V1|A0A2E4Y3V1_9PROT  LKKTGGLYPAPLKILEAVKAGVNSGPKKGYETEADLFGDLAMTRESKSLISLFEGQTSLK
tr|A0A2E6VRH4|A0A2E6VRH4_9DELT  LKQTQGLYPSPLAILDVVEHGYKRGIRAGFNMESEKFAELSQTPACGSLMSLFFGQQTCK
tr|A0A1F9FB59|A0A1F9FB59_9DELT  MEKTGGLYPAPLAIIDVVEAGTGKGPEAGYGEESRRFGELVMSKEAAALMTLFFAQTALK
```

```
ECHA_HUMAN                          T....T
                                                360       370       380       390       400
ECHA_HUMAN                          KNK....FGAPQKDVKHLAILGAGLMGAGIAQVSVDKG.LKTILKDATLTALDRGQQQVF
tr|A0A4R6XLA8|A0A4R6XLA8_9GAMM      KLK....NNNKTENVDSHAMIGAGFMGAGITEVSIKSG.YDVILKDISNEGLANAMKTIW
tr|A0A6A0IJT2|A0A6A0IJT2_9BACT      KRP....EGAEPRKVARLAVLGSGLMGEGIAAVSLPLN..PVVLKDVSERSLARAAGSLH
tr|A0A7V3QZU7|A0A7V3QZU7_9BACT      KPL....DATPPRPVERLAVLGGGFMGSGIAAVSLELA..PVVVRDLSQKALEGCARSVY
tr|A0A7V4SYJ6|A0A7V4SYJ6_9BACT      KPL....DDTAPRPVRRLAVLGGGFMGAGVASVSLKRS..AVVVKDVSDEVLSRCGKIVW
tr|A0A2A8D2N6|A0A2A8D2N6_9BACT      KNP....DADKARPINTLGVLGAGLMGAGIAEVSAENG.ADVVLKDQNIELAAGGKKHIY
tr|A0A2N1TGP0|A0A2N1TGP0_9SPIR      KNP....LKDKIVAVEKLAVLGAGLMGHGIAAVSTGLA.DTILLKDMTLDAAARGMKEIW
tr|A0A1G3QGW4|A0A1G3QGW4_9SPIR      KNP....YKSKARDVKKMAVLGAGLMGSGIASVSTQVC.DTILMKDMNLDAAARGMSEVW
tr|A0A1G3QTY3|A0A1G3QTY3_9SPIR      KNP....HKKKARSVKKLAIVGTGLMGSGIASVSAPVC.DTILMKDMNLDAAASGMSEVW
tr|A0A7X9L359|A0A7X9L359_9DELT      KNP....FEEMARPVRKIGVLGAGLMGHGIASVSTGAC.DTILIKDVSLEAVAKGMKEIY
tr|A0A522CJY0|A0A522CJY0_9SPIR      KNP....SSEKPRPVGKLAVMGAGLMGQGIAAVSLGLS.DTVLLKDVSVDAAAAGMKQIH
tr|A0A2N1RRJ3|A0A2N1RRJ3_9SPIR      KNP....KKNLARRTGKLAVIGAGLMGQGIASVSTPVC.DTILMKDMNLDASAKGMKEIW
tr|A0A2N2KFN7|A0A2N2KFN7_9DELT      KNP....QKELARKVGKLAIIGTGLMGQGIAAVSASVS.DTILLKDVSLDAAARGMKEIW
tr|A0A1V6AXX9|A0A1V6AXX9_9DELT      KNP....QKSLARKVGKMGVIGTGLMGQGIASVSTAIC.DTILMKDVKLDDAARGMKEMW
tr|A0A2N2HZL0|A0A2N2HZL0_9DELT      KNP....QKSLARKVRQMAVIGTGLMGQGIASVSTAIC.DTILMKDVKLDDAARGMKEIW
tr|A0A5E8ARP3|A0A5E8ARP3_9BACT      KNP....MKELARPVKTLGVLGAGLMGEGIAEVTVQNG.TDILLKDLQEPALAQAKKNIW
tr|A0A662A7B1|A0A662A7B1_9BACT      KNP....LQKQVKKVNSIAVLGAGLMGEGIAEISLQKG.WEIILKDISTEMLSKARNNIR
tr|D7CV16|D7CV16_TRURR              KNP....YAEAARAVRTVGILGAGLMGSGIAQVSAEAG.HQVILKDRNLSVAVKGKGSVW
tr|A0A3C2AKX4|A0A3C2AKX4_9FLAO      KNP....QAELARKTDTIAMVGAGFMGAGIAQVSAAKD.VRVLLKDIKQETLTQARQTVW
tr|A0A3B8ZLN3|A0A3B8ZLN3_9PLAN      KNP....LKELVRPVDTMAMMGAGFMGAGIAEVSAAKD.IRVLLKDIKQETLSQAKQTVW
tr|A0A345UGV8|A0A345UGV8_9BACT      KNP....WQGKDRKVERIGVLGSGLMGGGIADVSAANG.YRISLKDRDTASAVKGFEAIR
tr|A0A6C1P6A2|A0A6C1P6A2_9BACT      KRL....DKSLIGKAGTIGVLGAGLMGSGIADVSAG.GGYRVLLKDRDLESAAKGYAGIW
tr|A0A3M1X9W8|A0A3M1X9W8_9BACT      KNP....LADQVRPVKRVGILGAGLMGSGIAQVSATSG.LEVVLKDVSLEALGRAERQIY
tr|A0A354C796|A0A354C796_9DELT      KNP....RKDLVRPVSQIGILGAGLMGSGIANVSATSG.MKVLLKDINHEAVGWGEKSVW
tr|A0A3M1NUM8|A0A3M1NUM8_9BACT      KNP....LKSKVREVNTIGILGAGLMGSGIAEVSASKG.YEIFIKDISLQAIASGEKRIW
tr|A0A3M2KRC8|A0A3M2KRC8_9BACT      KHP....TPEKARPVQRIGILGAGLMGSGIAQVSVMNG.LDVVIKDISHEALAKAEKTIY
tr|A0A5S9IHR0|A0A5S9IHR0_9BACT      KNP....QQDKVKKVEKIGMLGAGLMGAGIANISATKD.IRVVLKDISMEAANNGFKNIS
tr|A0A6M1ST81|A0A6M1ST81_9BACT      KNP....DKEKAKEVNKIGVLGAGLMGSGIADVSVNND.YRVLLKDQDIEPAAKGEQAIW
tr|A0A521AAE5|A0A521AAE5_9BACT      KNP....NSEQVSDIHKIGVLGAGLMGSGIADVSATKGEYRILLKDQEIQQAAEGKKHIW
tr|A0A1M4ZMD6|A0A1M4ZMD6_9BACT      KNP....ASEKTEPIQKIGVLGAGLMGSGIADVSANKGDYRILLKDQNAEQAVEGKKHIW
tr|A0A2A2GCS2|A0A2A2GCS2_9BACT      KNP....NEEKAKEIQKIGVLGAGLMGSGIADVSANNE.YRVLLKDQEAEQAAKGKKSIW
tr|A0A5D3YIH7|A0A5D3YIH7_9BACT      KNP....QEDQAKGIQKMGVLGAGLMGSGIADVSVNND.FRVLLKDQSVEQASKGKQSIW
tr|A0A6M1T9G0|A0A6M1T9G0_9BACT      KNS....DEDKGREVEKIGVLGAGLMGSGIADVSANND.YRVLLKDQTVEQAGEGKKDIW
tr|A0A6A8Q1Y8|A0A6A8Q1Y8_9BACT      KVP....HKEKARDVSKICVLGAGLMGSGIADVSVNKGEYRVLLKDQTIEKAAQGEKEIW
tr|A0A521BVJ3|A0A521BVJ3_9BACT      KVP....KPDLVKPVNKIGVLGAGLMGSGIADVSINKGGYRVLLKDQDMESAAKGEKEIW
tr|A0A359E0Y8|A0A359E0Y8_9BACT      KVP....NSDLVKPVKKIGVLGAGLMGSGIAEVSVDKGDYRVLLKDQTIENAAQGEKEIW
tr|A0A2D8CIM6|A0A2D8CIM6_9BACT      KVP....NSDLVKPVKKIGVLGAGLMGSGIAEVSVDKGDYRVLLKDQTIENAAQGEKEIW
tr|A0A2D9FWF5|A0A2D9FWF5_9BACT      KVP....DPDMVKKVQRIGVLGAGLMGSGIADVSVNKGDYRVLLKDQDIDNAAQGEKEIW
tr|A0A1B6YB70|A0A1B6YB70_9BACT      KNP....QKDLVKPVNKIGVLGAGLMGSGIADVSINKGKYSVLLKDRDLESAAKGEQGIW
tr|A0A2D4ZTC0|A0A2D4ZTC0_9BACT      KNP....LKELVLPIDKIGVLGAGLMGSGIADVSINKGKYSVLLKDRDLESASKGEKSIW
tr|A0A3D4UVW7|A0A3D4UVW7_9BACT      KNP....LKELVLPIDKIGVLGAGLMGSGIADVSINKGKYSVLLKDRDLESASKGEKSIW
tr|A0A3F3I3M2|A0A3F3I3M2_9BACT      KNP....LKELVLPIDKIGVLGAGLMGSGIADVSINKGKYSVLLKDRDLESASKGEKSIW
tr|A0A3D1G7L9|A0A3D1G7L9_9BACT      KNP....LADQVKPVKTIGVLGAGLMGSGIADVSINKGKYSVLLKDRTLEDAAKGEKVIW
tr|A0A3M8G1Y8|A0A3M8G1Y8_9BACT      KNP....LEGKQVPIKTIGVLGAGLMGSGVADVSVNKGGYSVLLKDQTPEAAAKGEKVIW
tr|A0A5Q4F391|A0A5Q4F391_9BACT      NNP....MENRVRPVETIAVLGAGLMGAGIAEVSAEQG.YRVWLKDQTADRALKGKAQID
tr|A0A6I7NPV0|A0A6I7NPV0_9BACT      KNS....LDSEKRVVKNIGILGAGLMGAGIAEVSIEEG.YHVWLKDQSLDNAMRGQSGIR
tr|A0A651G1J2|A0A651G1J2_9BACT      KNP....HPEKIKSVSNIAVLGAGLMGSGITEVSIDQK.YHVWLKDQTIDNALKGSETIR
tr|A0A371QRK5|A0A371QRK5_9BACT      KNP....FEKKAKEVKNIGVLGAGLMGSGITEVSVEDG.IHVWLKDQSLENAAKGEADID
tr|A0A2N0VGJ5|A0A2N0VGJ5_9BACT      KNP....LKDQAKEVERIGVLGAGLMGSGIAEVSVDNG.YHVWLKDQTVENAAKGEAEIL
tr|A0A316TYJ0|A0A316TYJ0_9BACT      KNP....LDDKKREVKRIAVLGAGLMGSGIAEVSAEEG.YHVWLKDQTMENAMEGEAEIE
tr|A0A651GLZ8|A0A651GLZ8_9BACT      KNP....LDEKKRDVKRIGVLGAGLMGSGITEVSIEEG.YHVWLKDQTLENAVKGEAEIE
tr|A0A7Y5V3S5|A0A7Y5V3S5_9BACT      KDTGAPSAGAEAQPVERLGILGAGFMGAGIASVAVQQGAM.VRLKDADLGRVGKGIAAVS
tr|A0A7W1SHC0|A0A7W1SHC0_9BACT      KDAGVSDPAPKPRDIERLGILGAGFMGAGIASVAVQQGTI.VRLKDTDHARVGKGLASVR
tr|A0A7Y5PBT7|A0A7Y5PBT7_9BACT      KDPGVDEPAPEPHEVDTLGIIGAGFMGAGIASVAVQQG.TPVRLKDTDTTRVSRGIAAVS
tr|A0A7Y5TXL8|A0A7Y5TXL8_9BACT      KDPGVDEPAPEPHEVDTLGIIGAGFMGAGIASVAVQQG.TPVRLKDTDTTRVSRGIAAVS
tr|A0A423PQ96|A0A423PQ96_9GAMM      RTTFVA.RNVKARAVRRVGVLGAGLMGAGIATVTIDKAGLPVRLKDVSDDGLARGLDHVQ
tr|U2G066|U2G066_9GAMM              KATFVG.RNVKPRDVHQVGVLGAGLMGAGIATVTVDKAGLPVRLKDVSNEGLARGMQHVR
tr|A0A2E0J1F4|A0A2E0J1F4_9GAMM      KATFVA.KNVKPREVRQIGVLGAGLMGAGIATVTIDKAGLPVRLKDVSDDGLARGMQHVN
tr|A0A2D4SCM8|A0A2D4SCM8_9GAMM      RETFVA.EGVVPREIDHVGVLGAGLMGAGIAMVTINKAGLDVRLKDKDNEGLARGLSSVR
tr|L0WJH1|L0WJH1_9GAMM              KETFVA.DGVQARQIHRTGVLGAGLMGAGIALTTVNKAKLPVRLKDRDDKGLAHGVQYLR
tr|A0A1H5XF61|A0A1H5XF61_9GAMM      KETFVA.DGVKARNMEKVGVLGAGLMGAGIALTTINKAKLPVRLKDRDDKGLAHGVKYVS
tr|A0A2E9TS48|A0A2E9TS48_9GAMM      KETFVA.DGVKARDIEKVGVLGAGLMGAGIALTTINKAKLPVRLKDRDDKGLAHGVKYVS
tr|A0A095UE19|A0A095UE19_9GAMM      KETFVA.DGLKARNIEKVGVLGAGLMGAGIALTTINKAKLPVRLKDRDDNGLAHGVKYVS
tr|A0A7G2S9A0|A0A7G2S9A0_9GAMM      KETFVA.DGVKARNIEKVGVLGAGLMGAGIALTTINKAKLPVRLKDRDDNGLAHGVKYVS
tr|B5JU27|B5JU27_9GAMM              KETFVADESVETRTIERVGVLGAGLMGAGIALNTVAKAKLPVRLKDRDAKGLSHGVGYVA
tr|A0A1Y0IHP1|A0A1Y0IHP1_9GAMM      KETFVGDDRVVPRPVKQIAVLGAGLMGAGIALNTVSKTGLPVRLKDRDAKGLSHGVSYVR
tr|A0A316FZ14|A0A316FZ14_9GAMM      KDKGV.ESDVEPKPVKRVGVLGGGLMGAGIAYVSIDKAGKQVRLKDVSTDGVNNALKYSW
tr|A0A498C231|A0A498C231_9GAMM      KDSGT.DAGADPRPVRRVGVLGAGLMGAGIAYVTSAKAETPVRLKDIALEGLNSGMRHIN
tr|Q0A6T4|Q0A6T4_ALKEH              KDSGT.DAGVEPRPVRRVGVLGAGLMGAGIAYVTSAKAGTPVRLKDIALEGLNNGMRHID
tr|A0A3E0WL10|A0A3E0WL10_9GAMM      KDLGVADDKVKPREIHRIGVLGAGLMGAGISYVTIGKAKIPVRLKDKEPQGLSRGLKQIH
tr|A0A3S1BU16|A0A3S1BU16_9GAMM      KDNGVADAGIQPRPVHRIGVLGAGLMGAGIAYVTANKARLAVRLKDKEVAGLNRGLAYID
tr|A0A7V8QFN9|A0A7V8QFN9_9GAMM      KDTGVSDPKVTPREVHRVGVLGAGLMGAGISYVTADKAGIPVRLKDVKPEGLSAGMKHVH
tr|A0A1H8PPF9|A0A1H8PPF9_9GAMM      KDTGTDDASAAPRQVERTAVLGAGLMGAGIAFVTTNKAGLPVRLKDIAVEGLNKGMQHLN
tr|A0A6H0J0V7|A0A6H0J0V7_9GAMM      KDTGT.EADVEPRPVRRVAVLGAGLMGAGIAFVITNKAELPVRLKDVRVEGLNKGMQHIN
tr|V5EZX6|V5EZX6_9VIBR              KEHF...GNATPAKVNHVGVLGGGLMGAGIAHVSLAKGKFAVNLKDVAQDGVLNALSYNY
tr|A0A6I1QBM2|A0A6I1QBM2_9VIBR      KEHF...GNATPAKVNHVGVLGGGLMGAGIAHVSLAKGKFAVNLKDVAQDGVLNALSYNY
tr|A0A511QRL5|A0A511QRL5_9VIBR      KEHF...GNAVPAKVKHVGVLGGGLMGAGIAHVSLAKAKLPVNLKDVSQDGVLNALGYNY
tr|A0A7Y0JZP4|A0A7Y0JZP4_9VIBR      KENF...GNAVPAKVKHVGVLGGGLMGAGIAHVSLAKAKLPVNLKDVSQDGVLNALGYNY
tr|A0A193KD24|A0A193KD24_9VIBR      KEHF...GNATPAKVKHVGVLGGGLMGAGIAHVSLAKAKLPVNLKDVSQDGVLNALGYNY
tr|A0A1S1HM72|A0A1S1HM72_PROST      NETG...SAAVPAKFKQIGVLGGGLMGGGIAYVTAMRGDLAVRIKDINESGVAQALRYSW
tr|A0A7T8I613|A0A7T8I613_9GAMM      NETG...SAAVPAKFKQIGVLGGGLMGGGIAYVTAMRGDLAVRIKDINESGVAQALRYSW
tr|A0A140NIF0|A0A140NIF0_PROSM      NETG...SSAKPLAFKQIGVLGGGLMGGGIAFVTAMKGDLPVRIKDINEKGITHSLRYSW
tr|A0A379GMB7|A0A379GMB7_PROST      NETG...SSAKPLAFKQIGVLGGGLMGGGIAFVTAMKGDLPVRIKDINEKGITHSLRYSW
tr|A0A379H393|A0A379H393_PROST      NETG...SSAKPLAFKQIGVLGGGLMGGGIAFVTAMKGDLPVRIKDINEKGITHSLRYSW
tr|B2Q0L5|B2Q0L5_PROST              NETG...SSAKPLAFKQIGVLGGGLMGGGIAFVTAMKGDLPVRIKDINEKGITHSLRYSW
tr|A0A6I3JUE9|A0A6I3JUE9_9GAMM      NETG...SSAKPLKFNQIGILGGGLMGGGIAFVTAMKGNLPVRIKDINDKGVTHALGYSW
tr|A0A7D4P594|A0A7D4P594_YERMW      KETG...GDAQPRAIHRVGVLGGGLMGGGIANVTATRAGLPVRIKDINPQGINQALKYTW
tr|A0A7U7IVK6|A0A7U7IVK6_YEREN      KESG...GKAQPRAFHRVGVLGGGLMGGGIAHVTATRAGLPVRIKDINPTGINQALKYTW
tr|A0A447RIF5|A0A447RIF5_YEREN      KESG...GKAQPRAFHRVGVLGGGLMGGGIAHVTATRAGLPVRIKDINPTGINQALKYTW
tr|A0A7H4ZLV9|A0A7H4ZLV9_YERP4      KESG...GKAQPRAFHRVGVLGGGLMGGGIAHVTATRAGLPVRIKDINPTGINQALKYTW
tr|A0A0H3NX46|A0A0H3NX46_YERE1      KESG...GKAQPRAFHRVGVLGGGLMGGGIAHVTATRAGLPVRIKDINPTGINQALKYTW
tr|A0A7T9XUV7|A0A7T9XUV7_YEREN      KESG...GKAQPRAFHRVGVLGGGLMGGGIAHVTATRAGLPVRIKDINPTGINQALKYTW
tr|A0A7U7IYV8|A0A7U7IYV8_YEREN      KESG...GKAQPRAFHRVGVLGGGLMGGGIAHVTATRAGLPVRIKDINPTGINQALKYTW
tr|A0A0H5G7G2|A0A0H5G7G2_YEREN      KESG...GKAQPRAFHRVGVLGGGLMGGGIAHVTATRAGLPVRIKDINPTGINQALKYTW
tr|A0A2A7TDI2|A0A2A7TDI2_YERKR      KETG...GTAQPRDIHRVGVLGGGLMGGGIANVTATRAGLPVRIKDINPTGINQALKYTW
tr|A0A0T9M261|A0A0T9M261_YERKR      KETG...GTAQPRDIHRVGVLGGGLMGGGIANVTATRAGLPVRIKDINPTGINQALKYTW
tr|A0A0A0CMP1|A0A0A0CMP1_PHOLU      NETG...SSERPAEIKRVGILGGGLMGGGVANVTATRGKLPVRIKDINEKGISQVLKYTW
tr|A0A329VG55|A0A329VG55_9GAMM      NETG...SSEKPAKIKHVGILGGGLMGGGIANVTATRGKLPVRIKDINEKGISQVLKYTW
sp|Q7N288|FADJ_PHOLL                NETG...SSEKPAKIKHVGILGGGLMGGGIANVTATRGKLPVRIKDINEKGISQVLKYTW
tr|A0A6L9JMX5|A0A6L9JMX5_PHOLM      NETG...SSEKPAKIKHVGILGGGLMGGGIANVTATRGKLPVRIKDINEKGISQVLKYTW
tr|A0A7X5HQ29|A0A7X5HQ29_PHOLM      NETG...SSEKPAKIKHVGILGGGLMGGGIANVTATRGKLPARIKDINEKGISQVLKYTW
tr|A0A1C0U051|A0A1C0U051_9GAMM      NETG...SSEKPAKIERVGILGGGLMGGGIANVTATRGKLPARIKDINEKGISQALKYTW
tr|A0A7X5QF62|A0A7X5QF62_9GAMM      NETG...SSEKPAKIERVGILGGGLMGGGIANVTATRGKLPARIKDINEKGISQVLKYTW
```

```
tr|A0A2D0KWM9|A0A2D0KWM9_9GAMM  NETG...SSELPAQIRHVGILGGGLMGGGIANVTATRGHLPSRIKDINERGINQALKYTW
tr|A0A2D0LAI1|A0A2D0LAI1_9GAMM  NETG...SSELPAQIRHVGILGGGLMGGGIANVTATRGHLPSRIKDINERGINQALKYTW
tr|A0A2D0IXC8|A0A2D0IXC8_XENBU  NETG...SSEQPVKIKHVGILGGGLMGGGIANVTVTRGNLPVRIKDINEKGINQALKYTW
tr|W1JAM5|W1JAM5_9GAMM          NETG...SSEQPAKIKHVGILGGGLMGGGIASVTATRGNLPVRIKDINEKGINQALKYTW
tr|A0A3D9UED3|A0A3D9UED3_9GAMM  NETG...SSEQPAKIKHVGILGGGLMGGGIASVTATRGNLPVRIKDINEKGINQALKYTW
tr|A0A1I3JCA9|A0A1I3JCA9_9GAMM  NETG...SSEKPAKIKQVGILGGGLMGGGIANVTITRGKLPVRIKDISEKGINQALKYTW
tr|A0A068QUL9|A0A068QUL9_9GAMM  NETG...SSEQPAEIKQVGILGGGLMGGGIANVTATRGNLPVRIKDINEKGINQALKYTW
tr|A0A0M0TCH6|A0A0M0TCH6_9GAMM  NETG...SSEPPAEIRHVGILGGGLMGGGIANVTVTRGNLPARIKDINEKGINQALKYTW
tr|A0A1Q5U854|A0A1Q5U854_9GAMM  NETG...SLEQPAEIKHVGILGGGLMGGGIANVTVTRGNLPVRIKDINEKGINQALKYTW
tr|A0A2D0ISP1|A0A2D0ISP1_9GAMM  NETG...SSEQPAEIRHVGILGGGLMGGGIANVTVTRGNLPARIKDINEKGISQALKYTW
tr|A0A1Q5TUI8|A0A1Q5TUI8_9GAMM  NETG...SSEQPTEIKQVGILGGGLMGGGIANVTVTRGNLPARIKDISEKGINQALKYTW
tr|A0A2D0KJI7|A0A2D0KJI7_9GAMM  NETG...SSEQPAEIKHVGILGGGLMGGGIANVTVTRGNLPARIKDIHEKGINQALKYTW
tr|A0A1Y2SB97|A0A1Y2SB97_9GAMM  NETG...SSSQPAEIKHVGILGGGLMGGGIANVTMTRGNLPVRIKDINEKGINQALKYTW
tr|A0A1I5DWI7|A0A1I5DWI7_9GAMM  NETG...SSEQPAEIKYVGVLGGGLMGGGIANVTMTRGNLPVRIKDISEKGINQALKYTW
tr|A0A1I7GWY6|A0A1I7GWY6_9GAMM  NETG...SSEQPAEIKQVGILGGGLMGGGIANVTITRGNLSARIKDINEKGINQALEYTW
tr|D3VKY8|D3VKY8_XENNA          NETG...SSEQPAEIKQVGILGGGLMGGGIANVTMSRGNLPVRIKDINEKGINQALKYTW
tr|A0A2G0Q6Q9|A0A2G0Q6Q9_9GAMM  NETG...SSEQPSEIKHVGILGGGLMGGGIANVTMTRGNLPVRIKDINEKGINQALKYTW
tr|A0A2D0JU24|A0A2D0JU24_9GAMM  NETG...SSEQPADIKHVGILGGGLMGGGIANVTITRGNLPARIKDINEKGINQALKYTW
tr|A0A0J5FN38|A0A0J5FN38_9GAMM  NETG...SSEQPAEIKHVGILGGGLMGGGIANVTITRGNLPARIKDINEKGINQALKYTW
tr|A0A432XLD4|A0A432XLD4_9GAMM  KETGA..GDTKPRDIQRVGVLGGGLMGGGIAYVTAAKAGLPARIKDINENGIVHALKYSY
tr|A0A2D8HU84|A0A2D8HU84_9GAMM  KETGA..DGVEPDSVKRVGVLGGGLMGGGIAYVTAAKAGIPARIKDISEDGISNALHYSY
tr|A0A656X1Q2|A0A656X1Q2_9GAMM  KETGA..DGVEPDPVKRVGVLGGGLMGGGIAYVTAAKAGIPARIKDISEDGISNALHYSY
tr|A0A4Q1QH79|A0A4Q1QH79_9GAMM  KETGA..DGVEPDPIKRVGVLGGGLMGGGIAYVTAAKANIPARIKDISEDGVRHALHYSY
tr|A0A1G7LQE6|A0A1G7LQE6_9GAMM  KETGA..DGVEPDPIKRVGVLGGGLMGGGIAYVTAAKANIPARIKDIAEDGVRHALHYSY
tr|A0A432YVG7|A0A432YVG7_9GAMM  KETGA..DGVEPDPIKRVGVLGGGLMGGGIAYVTAAKANIPARIKDISEDGVRHALHYSY
tr|A0A1J5N0P5|A0A1J5N0P5_9GAMM  KETG...SDAQPEKVNKAMILGGGLMGGGIANVTAIKANIPTRIKDISHDGISQALKYSF
tr|A0A0D8D548|A0A0D8D548_9GAMM  KEQGV..AGVEPEKIAKAGVLGGGLMGGGIAFVTATKAKVPVRIKDISHKGINHALKYGF
tr|A0A3E0TNM2|A0A3E0TNM2_9GAMM  KEDGV..AGVAPEKISKAGVLGGGLMGGGIAFVTATKAKLPVRVKDIAHNGISAALKYSF
tr|A0A3E0UD67|A0A3E0UD67_9GAMM  KEDGV..EGVAPEKVAKAGVLGGGLMGGGIAFVTATKAKVPVRVKDIAHNGINAALKYSF
tr|A0A3E0U0P4|A0A3E0U0P4_9GAMM  KEDGV..EGVAPEKVAKAGVLGGGLMGGGIAFVTATKAKVPVRVKDIAHNGINAALKYSF
tr|A0A0M2V8B1|A0A0M2V8B1_9GAMM  KETGA..GDTQPKPVHKVAVLGGGLMGGGIANVSSTKAGVPVRIKDINEQGISNALKYSY
tr|A0A285ITY5|A0A285ITY5_9GAMM  KETGA..GETQPQQVRKAAVLGGGLMGGGIANVSSTKAGVPVRIKDINEQGISNALKYSY
tr|A0A486XTB9|A0A486XTB9_9GAMM  KETGA..GDTKPQKVRKAAVLGGGLMGGGIANVTATKAGIPVRIKDISEQGISNALKYSY
tr|I1E1G1|I1E1G1_9GAMM          KETGA..GETKPNKVRKAAVLGGGLMGGGIANVTATKAGIAVRIKDINEQGISNALKYSY
tr|A0A1H6KJ68|A0A1H6KJ68_9GAMM  KESGA..GDTKPNKVRKAAVLGGGLMGGGIANVTATKAGIPVRIKDINEQGISNALKYSY
tr|A0A0X3Y764|A0A0X3Y764_9GAMM  KESGA..GDTKPNKVRKAAVLGGGLMGGGIANVTATKAGIPVRIKDINEQGISNALKYSY
tr|A0A2N1YEV4|A0A2N1YEV4_9GAMM  KESGA..GDTKPNKVRKAAVLGGGLMGGGIANVTATKAAIPVRIKDINEQGISNALKYSY
tr|A0A3P3QCM3|A0A3P3QCM3_9GAMM  KETGA..GDVQPHKVHKAAILGGGLMGGGIANVTATKAGVPVRIKDINQQGISNAMKYSY
tr|F7NT72|F7NT72_9GAMM          KETGA..GDVVPRKVHKAAILGGGLMGGGIANVTATKAGVPVRIKDISQQGISNAMKYSY
tr|A0A3S2TW63|A0A3S2TW63_9GAMM  KETGA..GDVVPRKVRKAAILGGGLMGGGIANVTATKAGVPVRIKDISQQGISNAMKYSY
tr|A0A3D5BGW7|A0A3D5BGW7_9GAMM  KETGA..GDVKPSKVHKAAVLGGGLMGGGIANVTAGKAGIPVRIKDINQQGISNALKYSY
tr|A0A5C7TI17|A0A5C7TI17_9GAMM  KESGA..GDAKPRKVKKAAVLGGGLMGGGIANVTAGKAGVPVRIKDINQTGISNALKYSF
tr|A0A0U4W982|A0A0U4W982_9GAMM  KESGA..GDAKPRKVKKAAVLGGGLMGGGIANVTAGKAGVPVRIKDINQTGIGNALKYSF
tr|A0A2I0FCM2|A0A2I0FCM2_9GAMM  KEDGV..AGVAPKKVKKAIVLGGGLMGGGIANVTATKAKVPVRIKDIAPQGLLNAQKYTY
tr|A0A2G2IVL1|A0A2G2IVL1_9GAMM  KEDGV..EGVAPKKVKKAVVLGGGLMGGGIANVTATKAKVPVRIKDIAPQGLLNAQKYTY
tr|A6FI21|A6FI21_9GAMM          KEDGV..EGVAPKKVKKAVVLGGGLMGGGIANVTATKAKVPVRIKDIAPQGLLNAQKYTY
tr|A0A4U1BNR2|A0A4U1BNR2_9GAMM  KETGA..GDVEPKPVKRTMVLGGGLMGGGIANVTAVKAKVPVRIKDIAHEGISKAMSSTW
tr|E1SQ60|E1SQ60_FERBD          KESGA..GDAKPLPLNKVMVLGGGLMGGGIASVTALKAKLPARIKDIAEPGLQNALAYSY
tr|A0A4Y6IZ09|A0A4Y6IZ09_9GAMM  KESGA..GDVSPKAVNKVMVLGGGLMGGGIASVTTTKAKIPVRVKDISETGLSNALAYAY
tr|A0A0C3QSZ7|A0A0C3QSZ7_9GAMM  KETGA..GDVSPQAVNKVMVLGGGLMGGGIASVTTTKAKIPVRVKDISETGLSNALAYAY
tr|A0A6L7HXW0|A0A6L7HXW0_9GAMM  KETGA..GDVTPKAVNKVMVLGGGLMGGGIASVTTTKAKIPVRVKDISEIGLSNALAYAY
sp|A3QFP3|FADJ_SHELP            KESGA..GDVSPKAVNKVMVLGGGLMGGGIASVTTTKAKIPVRVKDISEIGLSNALAYAY
tr|A0A1E5IXH7|A0A1E5IXH7_SHECO  KESGA..GDVQAKPVKKAMILGGGLMGGGIASVTTTKAKIPARVKDINETGLSNALSYAY
tr|A0A411PKQ0|A0A411PKQ0_9GAMM  KETGA..EGAEPRDINKAMVLGGGLMGGGIASVTTTKAKVPVRVKDISEQGLSNALAYAY
tr|A0A6G9QKM3|A0A6G9QKM3_9GAMM  KETGA..AGAEPRKINKAMVLGGGLMGGGIASVTTTKAKVPVRVKDISEQGLSNALAYAY
tr|A0A6P1UL63|A0A6P1UL63_9GAMM  KETGA..EGAVPRKVKKVMVLGGGLMGGGIASVTTTKAKIPARVKDISEQGLSNALSYAY
tr|A0A2N1ERI9|A0A2N1ERI9_9GAMM  KETGA..EGAVPRKVKKVMVLGGGLMGGGIASVTTTKAKIPARVKDISEQGLSNALSYAY
tr|A0A7W4FU55|A0A7W4FU55_9GAMM  KETGA..EGAVPRKVKKVMVLGGGLMGGGIASVTTTKAKIPARVKDISEQGLSNALSYAY
sp|Q8ECP7|FADJ_SHEON            KETGA..EGATPRKVKKAVILGGGLMGGGIASVTTTKAKIPARVKDINEKGLSNALSYAY
tr|A0A501XZY8|A0A501XZY8_9GAMM  KETGA..EGATPRKVKKAVILGGGLMGGGIASVTTTKAKIPARVKDINEKGLSNALSYAY
tr|A0A2W5DCZ0|A0A2W5DCZ0_SHEOE  KETGA..EGATPRKVKKAVILGGGLMGGGIASVTTTKAKIPARVKDINEKGLSNALSYAY
tr|A0A1E3V3C8|A0A1E3V3C8_9GAMM  KETGA..EGATPRKVKKAVILGGGLMGGGIASVTTTKAKIPARVKDINEKGLSNALSYAY
tr|A0A1Z4AI20|A0A1Z4AI20_9GAMM  KETGA..EGATPRKVKKAVILGGGLMGGGIASVTTTKAKIPARVKDINEKGLSNALSYAY
tr|A0A7X9LJL9|A0A7X9LJL9_9GAMM  KETGA..EGATPRKVKKAVILGGGLMGGGIASVTTTKAKIPARVKDINEKGLSNALSYAY
tr|A0A073KMY5|A0A073KMY5_9GAMM  KETGA..EGATPRKVKKAVILGGGLMGGGIASVTTTKAKIPARVKDINEKGLSNALSYAY
sp|A0KV76|FADJ_SHESA            KETGA..EGATPRKVKKAVILGGGLMGGGIASVTTTKAKIPARVKDINEKGLSNALSYAY
tr|A0A220UTH7|A0A220UTH7_9GAMM  KETGA..EGATPRKVKKAVILGGGLMGGGIASVTTTKAKIPARVKDINEKGLSNALSYAY
tr|A0A5B8R6W1|A0A5B8R6W1_9GAMM  KETGA..EGATPRKVKKAVILGGGLMGGGIASVTTTKAKIPARVKDINEKGLSNALSYAY
tr|V1DAI4|V1DAI4_9GAMM          KETGA..EGATPRKVKKAVILGGGLMGGGIASVTTTKAKIPARVKDINEKGLSNALSYAY
tr|A0A448CPQ4|A0A448CPQ4_SHEPU  KETGA..EGATPRKVKKAVILGGGLMGGGIASVTTTKAKIPARVKDINEKGLSNALSYAY
tr|A0A252ERQ3|A0A252ERQ3_SHEPU  KETGA..EGATPRKVKKAVILGGGLMGGGIASVTTTKAKIPARVKDINEKGLSNALSYAY
sp|Q0HKD1|FADJ_SHESM            KETGA..EGATPRKVKKAVILGGGLMGGGIASVTTTKAKIPARVKDINEKGLSNALSYAY
sp|Q0HWN3|FADJ_SHESR            KETGA..EGATPRKVKKAVILGGGLMGGGIASVTTTKAKIPARVKDINEKGLSNALSYAY
tr|F7RQE3|F7RQE3_9GAMM          KETGA..EGATPRKVKKAVILGGGLMGGGIASVTTTKAKIPARVKDINEKGLSNALAYAY
tr|B8EE98|B8EE98_SHEB2          KETGA..EGASPRKVKKAVILGGGLMGGGIASVTTTKAKIPVRVKDISEKGLSNALAYAY
sp|A6WQ25|FADJ_SHEB8            KETGA..EGASPRKVKKAVILGGGLMGGGIASVTTTKAKIPVRVKDISEKGLSNALAYAY
tr|A0A448EK41|A0A448EK41_9GAMM  KETGA..EGASPRKVKKAVILGGGLMGGGIASVTTTKAKIPVRVKDISEKGLSNALAYAY
tr|A0A553JHX1|A0A553JHX1_SHEHA  KESGA..GDAKPRTIKKVMVLGGGLMGGGIASVTTTKAKIPARVKDISEQGLSNALSYAY
tr|B8CPY6|B8CPY6_SHEPW          KETGA..GDVEPRKVKKAMILGGGLMGGGIASVTTTKAKIPARVKDISETGLSNALSYAY
tr|A0A431WFC4|A0A431WFC4_9GAMM  KETGA..GDVEPREVHKAMVLGGGLMGGGIASVTTTKAKIPARVKDISEQGLSNALSYAY
tr|A8FTR7|A8FTR7_SHESH          KETGA..GDVEPRKVHKAMVLGGGLMGGGIASVTTTKAKIPARVKDISEQGLSNALSYAY
tr|A0A431WNL0|A0A431WNL0_9GAMM  KETGA..GDVEPREVHKAMVLGGGLMGGGIASVTTTKAKIPARVKDISEQGLSNALSYAY
tr|A0A550AEC5|A0A550AEC5_9GAMM  KETGA..GDVKPRKVSKAMILGGGLMGGGIASVTTTKAKIPARVKDISEQGLSNALSYAY
tr|A0A7L4WW90|A0A7L4WW90_9GAMM  KETGA..GDVKPRKVSKAMILGGGLMGGGIASVTTTKAKIPARVKDISEQGLSNALSYAY
tr|A9DDU3|A9DDU3_9GAMM          RETGA..GDVVPRKVNKVMVLGGGLMGGGIASVTTSKAKIPARVKDISEQGLSNALSYAY
tr|A0A330M2B9|A0A330M2B9_9GAMM  RETGA..GDVVPRKVNKVMVLGGGLMGGGIASVTTSKAKIPARVKDISEQGLSNALSYAY
tr|A0A5N8UFC7|A0A5N8UFC7_9GAMM  KETGA..GDVVPRKVNKAMVLGGGLMGGGIASVTTTKAKIPARVKDISEQGLSNALSYAY
tr|A0A1S6HN57|A0A1S6HN57_9GAMM  KETGA..GDVEPRKVNKAMVLGGGLMGGGIASVTTTKAKIPARVKDISEQGLSNALSYAY
tr|D4ZMH7|D4ZMH7_SHEVD          KETGA..GDVKPRKVNKAMILGGGLMGGGIASVTTSKAKIPARVKDISEQGLSNALSYAY
tr|A0A3L8Q213|A0A3L8Q213_9GAMM  KEA....GEAEIRDLNKAVVLGGGLMGGGIASVTATKAKMPVRVKDIAEQGLSNALAYAY
tr|A0A3A6U4N9|A0A3A6U4N9_9GAMM  KEA....GEANSKSVKKAVVLGGGLMGGGIASVTSTKAKVPVRVKDINEQGLSNALSYAY
tr|A0A4Q5MA37|A0A4Q5MA37_9GAMM  KEA....GEADAKKVNKAVVLGGGLMGGGIASVTTTKAGIPARVKDINENGLSNALSYAY
tr|A0A1L6LSX5|A0A1L6LSX5_9DELT  KDSGVSDPSVKPRRVEKVAMLGAGLMGGGIAYVTMNAG.IPVRLKDKDDASLGRGLKYVA
tr|A0A2W4L9B9|A0A2W4L9B9_9PROT  KATGVADSNVRPKEVRSVAMLGAGLMGGGIAYVTSAIAGIPVRFKEKDAEGLGRGFAYVR
tr|A0A2W4M4E6|A0A2W4M4E6_9PROT  KDPGIENPEVKPVEVHKVGVLGAGLMGAGVAYVTAAVAGLPVRLRDRDEAALGRGLAQIR
tr|A0A6I2GRX9|A0A6I2GRX9_9DELT  KENGTKNPEAKAREVKRVAVLGGGLMGGGIAYVASAVQGVPVRVKDKDLAGAGRALKQVQ
tr|A0A0H4WMK2|A0A0H4WMK2_9DELT  KENGTSNPDAKPREVKKVAVLGGGLMGGGIAYVASVLQGVPVRVKDKDDAGVGRAMKQVQ
tr|F8CJ36|F8CJ36_MYXFH          KENGTASPDAKPREVKKVAVLGGGLMGGGIAYVASSLQGVPVRVKDKDDAGVGRAMKQVQ
tr|A0A250K0F1|A0A250K0F1_9DELT  KENGTSNPDAKPREVKKVAVLGGGLMGGGIAYVASSLQGVPVRVKDKDDAGVGRAMKQVQ
tr|A0A7Y6WFZ2|A0A7Y6WFZ2_9DELT  KENGTSNPDAKPREVKKVAVLGGGLMGGGIAYVTSVLQGVPVRVKDKDDAGVGRAMKQVQ
tr|A0A7Y7C660|A0A7Y7C660_9DELT  KENGTANPDAKPREVKKVAVLGGGLMGGGIAYVTSVLQGVPVRVKDKDDAGVGRAMKQVQ
tr|A0A7Y4JFH2|A0A7Y4JFH2_MYXXA  KENGTSNPNAKPREVKKVAVLGGGLMGGGIAYVTSVLQGVPVRVKDKDDAGVGRAMKQVQ
tr|A0A4Y6CZQ9|A0A4Y6CZQ9_MYXXA  KENGTSNPDAKPREVKKVAVLGGGLMGGGIAYVTSVLQGVPVRVKDKDDAGVGRAMKQVQ
```

```
tr|A0A7Y4IKV5|A0A7Y4IKV5_MYXXA  KENGTSNPDAKPREVKKVAVLGGGLMGGGIAYVTSVLQGVPVRVKDKDDAGVGRAMKQVQ
tr|A0A511HHB0|A0A511HHB0_9DELT  KENGTSNPDAKPREVKKVAVLGGGLMGGGIAYVTSVLQGVPVRVKDKDDAGVGRAMKQVQ
tr|A0A4Y6CKY7|A0A4Y6CKY7_MYXXA  KENGTSNPDAKPREVKKVAVLGGGLMGGGIAYVTSVLQGVPVRVKDKDDAGVGRAMKQVQ
tr|Q1D1F2|Q1D1F2_MYXXD          KENGTSNPDAKPREVKKVAVLGGGLMGGGIAYVTSVLQGVPVRVKDKDDAGVGRAMKQVQ
tr|A0A7Y4MA14|A0A7Y4MA14_MYXXA  KENGTSNPDAKPREVKKVAVLGGGLMGGGIAYVTSVLQGVPVRVKDKDDAGVGRAMKQVQ
tr|A0A7T8Y4N9|A0A7T8Y4N9_MYXXA  KENGTSNPDAKPREVKKVAVLGGGLMGGGIAYVTSVLQGVPVRVKDKDDAGVGRAMKQVQ
tr|L7UE67|L7UE67_MYXSD          KENGTADASVKPREVKKVGVLGGGLMGGGIAYVAGVLQGAQVRVKDKDDAGAGRALKQVQ
tr|A0A511T9X1|A0A511T9X1_MYXFU  KENGTSDASVKPREVKKVAVLGGGLMGGGIAYVAGVLQGAQVRVKDKDDAGVARALKQVQ
tr|A0A7Y7C9C4|A0A7Y7C9C4_9DELT  KENGTADASVKPREVKKVAVLGGGLMGGGIAYVAGVLQGVQVRVKDKDDAGTSRALKQVQ
tr|A0A540X7W8|A0A540X7W8_9DELT  KENGTADASVKPREVKKVAVLGGGLMGGGIAYVAGVLQGAQVRVKDKDDAGASRAMKQVQ
tr|A0A3A5FK19|A0A3A5FK19_9DELT  KENGTANPSVKAREVKKVGVLGGGLMGGGIAYVSSALQGVHVRVKDKDDAGAGRALKQVQ
tr|A0A3A8JQL9|A0A3A8JQL9_9DELT  KENGTANASVKAREVKKVGVLGGGLMGGGIAYVAGVLQGVPVRVKDRDDAGAGRALKQVQ
tr|A0A7Y4NFA4|A0A7Y4NFA4_9DELT  KENGTANATVKPREVKKVGVLGGGLMGGGIAYVAGVLQGVPVRVKDRDDAGAGRALKQVQ
tr|A0A3A8HDQ7|A0A3A8HDQ7_9DELT  KENGTANASVKPREVKKVGVLGGGLMGGGIAYVAGVLQGVPVRVKDRDDAGAGRALKQVQ
tr|A0A3A8GR90|A0A3A8GR90_9DELT  KENGTSNPNLKPREVKKVGVLGGGLMGGGIAYVAGVLQGVPVRVKDRDDAGAGRALKQVQ
tr|A0A3A8SBD7|A0A3A8SBD7_9DELT  KENGTSNPNLKPREVKKVGVLGGGLMGGGIAYVAGVLQGVPVRVKDRDDAGAGRALKQVQ
tr|A0A3A8T0I8|A0A3A8T0I8_9DELT  KENGTSNPNLKPREVKKVGVLGGGLMGGGIAYVAGVLQGVPVRVKDRDDAGAGRALKQVQ
tr|A0A7X5BU07|A0A7X5BU07_9DELT  KENGTSNPNLKPREVKKVGVLGGGLMGGGIAYVAGVLQGVPVRVKDRDDAGAGRALKQVQ
tr|A0A7Y1RVL2|A0A7Y1RVL2_9DELT  KENGTSNPNLKPREVKKVGVLGGGLMGGGIAYVAGVLQGVPVRVKDRDDAGAGRALKQVQ
tr|A0A3A8THN2|A0A3A8THN2_9DELT  KENGTSNPNLKPREVKKVGVLGGGLMGGGIAYVAGVLQGVPVRVKDRDDAGAGRALKQVQ
tr|A0A7Y1RX76|A0A7Y1RX76_9DELT  KENGTSNPNLKPREVKKVGVLGGGLMGGGIAYVAGVLQGVPVRVKDRDDAGAGRALKQVQ
tr|A0A3A8RA58|A0A3A8RA58_9DELT  KENGTANPNLKPREVKKVGVLGGGLMGGGIAYVAGVLQGVPVRVKDKDDAGAGRALKQVQ
tr|A0A410RPB6|A0A410RPB6_CORCK  KENGTANPNLKPREVKKVGVLGGGLMGGGIAYVAGVLQGVPVRVKDRDDAGAGRALKQVQ
tr|A0A7Y4J474|A0A7Y4J474_CORCK  KENGTSNPNLKPREVKKVGVLGGGLMGGGIAYVAGVLQGVPVRVKDRDDAGAGRALKQVQ
tr|A0A3A8I9Z6|A0A3A8I9Z6_9DELT  KENGTSNPNLKPREVKKVGVLGGGLMGGGIAYVAGVLQGVPVRVKDRDDAGAGRALKQVQ
tr|H8MKE9|H8MKE9_CORCM          KENGTANPNLKPREVKKVGVLGGGLMGGGIAYVAGVLQGVPVRVKDRDDAGAGRALKQVQ
tr|A0A3A8H102|A0A3A8H102_9DELT  KENGTSNPNLKPREVKKVGVLGGGLMGGGIAYVAGVLQGVPVRVKDRDDAGAGRALKQVQ
tr|A0A554FW33|A0A554FW33_9DELT  KENGTANPSVKPREVKKVGVLGGGLMGGGIAYVAGVLQGVPVRVKDKDDAGAGRALKQVQ
tr|A0A3A8NPC0|A0A3A8NPC0_9DELT  KENGTANPSVKPREVKKVGVLGGGLMGGGIAYVAGVLQGVPVRVKDRDDAGAGRALKQVQ
tr|A0A3A8JUV3|A0A3A8JUV3_9DELT  KENGTANPSVKPREVKKVGVLGGGLMGGGIAYVAGVLQGVPVRVKDRDDAGAGRALKQVQ
tr|A0A3A8NEX6|A0A3A8NEX6_9DELT  KENGTANPSVKPREVKKVGVLGGGLMGGGIAYVAGVLQGVPVRVKDRDDAGAGRALKQVQ
tr|A0A3A8JEU8|A0A3A8JEU8_9DELT  KENGTANANVKPREVKKVGVLGGGLMGGGIAYVAGVLQGVPVRVKDKDDAGAGRALKQAQ
tr|A0A3A8LI76|A0A3A8LI76_9DELT  KENGTANASVKPREVKKVGVLGGGLMGGGIAYVAGVLQGVPVRVKDKDDAGAGRALKQVQ
tr|A0A085WXN8|A0A085WXN8_9DELT  KENGTSDPSVMPREVKKIGVLGGGLMGGGIAYVSAALQGVPVRVKDKDDAGVGRALKQVQ
tr|A0A2T4V0M5|A0A2T4V0M5_9DELT  KENGTANADVKPREVKKVGVLGGGLMGGGIAYVSSALQGVPVRVKDKDDAGVGRALKQVQ
tr|A0A0G2ZSW5|A0A0G2ZSW5_9DELT  KENGTASADVKPREVKKVGVLGGGLMGGGIAYVSSALQGVPVRVKDKDDAGVGRALKQVQ
tr|A0A3M2DKY0|A0A3M2DKY0_9DELT  KDSGVDDPSVEPRRVDKVGVLGAGLMGAGIAYVNSAVAGVRTRIKDKDDAGVGRGLAYIR
tr|A0A661NQ58|A0A661NQ58_9DELT  KDSGVDDEKVTARPVTKVGMLGAGLMGGGIAYVTSSIAKVPVRIKDRDHDGVLRGLRYVR
tr|A0A520YD99|A0A520YD99_9DELT  KDSGVDDPNVEPREVNKVGMLGAGLMGAGIAYVTTAVAKIPVRLKDRDADGVMAGLRYVR
tr|A0A7Y3BRE4|A0A7Y3BRE4_9DELT  KDSGVDDPNVEPREVNKVGMLGAGLMGAGIAYVTTAVAKIPVRLKDRDADGVMAGLRYVR
tr|A0A2D9TF90|A0A2D9TF90_9DELT  KDSGVDDPSVKPREVQRLGMLGAGLMGAGIAYVTATIAKKPVRLKDRDAKGVLWGLRYCR
tr|A0A2E0TP32|A0A2E0TP32_9DELT  KDSGVDDPEVKPREVSHVGMLGAGLMGAGIAYVTTALAKTPVRLKDRDADGVLSGLQYVR
tr|A0A2E4Y3V1|A0A2E4Y3V1_9PROT  KNR....YGSPENKPDWIGVIGGGLMGSGISLVNIQKG.IKTRLKDLSQESLGKSEKYVW
tr|A0A2E6VRH4|A0A2E6VRH4_9DELT  KNR....FGKPDHAVKTLGILGAGLMGAGIGLVSVQKG.TRVLLKDISQENLGKGKKQIY
tr|A0A1F9FB59|A0A1F9FB59_9DELT  KNR....FGKAATPLDTLGLLGAGLMGAGIAEVTVQKG.VRVLLKDVSAEALARGQKTIW
```

```
ECHA_HUMAN
                                         410          420          430          440          450          460
ECHA_HUMAN                               KGLNDKVKKKALTSFERDSIFSNLTGQLDYQGFEKADMVIEAVFEDLSLKHRVLKEVE..
tr|A0A4R6XLA8|A0A4R6XLA8_9GAMM           TDFNQLVKKKAMGVVERDRLMSKVKATTAAEDLNAADLVIEAVFEDIDLKQNILADVE..
tr|A0A6A0IJT2|A0A6A0IJT2_9BACT           SGLEKRRRSGALTRLARDRQWFGLLATTDPRDVAGCDLVVEAVFEDLALKRKVLAECE..
tr|A0A7V3QZU7|A0A7V3QZU7_9BACT           SGLSKRVQSGAISRFERDRLFSRLHLTQEAEALRRAGLVIEAVFEDLDLKRKVLAEVE..
tr|A0A7V4SYJ6|A0A7V4SYJ6_9BACT           EGLSKQVKSGAVTRFERDRLWARLTPSLDYEAIRHADLVVEAVFEDLALKRKVLAETE..
tr|A0A2A8D2N6|A0A2A8D2N6_9BACT           KAMNKKSDRRIISEFERDQIVERVTPTGDYNAFRHADVVIEAVPEDLDLKRSILSDTE..
tr|A0A2N1TGP0|A0A2N1TGP0_9SPIR           KGLDKRSRSGALTPFERDVQYGKIVPCDDYRNFRNTDLVIEAVFEDITLKRKVLREVE..
tr|A0A1G3QGW4|A0A1G3QGW4_9SPIR           KGIASQVKSGAVRKFDGDVMYGKLVPCDDYSRFRGTDIVIEAVFEDLSLKRKILKDVE..
tr|A0A1G3QTY3|A0A1G3QTY3_9SPIR           KGIVKQVQSGAVRRFDGDVMYGKLVPCDDYLRFKGADIVIEAVFEDINLKRTILKEAE..
tr|A0A7X9L359|A0A7X9L359_9DELT           KGLDKRSRSGGITNFDRDVQYGKVIACDDYSLFGRTDIVIEAVFEDLALKRRILAEVE..
tr|A0A522CJY0|A0A522CJY0_9SPIR           RGIQKRVKSGALRRFDGEALYGKVVPCDDYSMFKNTGMVIEAVFEDLGLKQRVLADVE..
tr|A0A2N1RRJ3|A0A2N1RRJ3_9SPIR           KGLSKRAESGAIFPFERDVQYGRLVPCDDYQYFKNTNIVIEAVFEDLALKRKILSDVE..
tr|A0A2N2KFN7|A0A2N2KFN7_9DELT           KGLDKKARSGAIVPFERDVQYGKLVPTDTYSLFKNTELVVEAVFEDLALKKRVLSDVE..
tr|A0A1V6AXX9|A0A1V6AXX9_9DELT           KGLEKKVKSGAILEFDSHVQYGKLVPCDDYSLFKNTDLVVEAVFEDLAVKQRVLADVE..
tr|A0A2N2HZL0|A0A2N2HZL0_9DELT           KGLEKKTKSGAIVGFDRDIQYGKLVPCDDYSLFKNTDLVVEAVFEDLAVKKRVLADVE..
tr|A0A5E8ARP3|A0A5E8ARP3_9BACT           SVLMKKMKTRAITKADAETIINRVHTTTTYEGFHKADVIIEAVFEDINIKHKVLKETE..
tr|A0A662A7B1|A0A662A7B1_9BACT           RALNKKVKQRVYLKTEADTFMNKLHSDIEYTGFNKPDLIIEAVFEDINLKHNIIADLE..
tr|D7CV16|D7CV16_TRURR                   KELDKRVGKGR.SAFERDVIDARITAVEDYAAFKRADLVIEAVLEDVELKRSVLKEVE..
tr|A0A3C2AKX4|A0A3C2AKX4_9FLAO           KDIGKKVKRKAMPQLDADRLMNRITGQLDYNNFEKVDVLIEAVFEDMKVKHMVMKECE..
tr|A0A3B8ZLN3|A0A3B8ZLN3_9PLAN           STIGTKVKRKAMSQLEADRMMSRIVGQLDYSHFEKAQLVIEAVFEDLDLKHKVLAECE..
tr|A0A345UGV8|A0A345UGV8_9BACT           RETDKKVKKRIMSSFEGDAHLARITPTGSYEHFRDIPIVIEAVFEDLKIKHAVLKEVE..
tr|A0A6C1P6A2|A0A6C1P6A2_9BACT           QELDKKRGKRIISPFERDRTSSLIHPTGDYRGFEKADLVIEAVFEDLDIKHKVLAEVE..
tr|A0A3M1X9W8|A0A3M1X9W8_9BACT           QALDRRVRQRALLPFQRDQILSRITPVTDYHGFDRVDLVIEAVFEDLELKRRVLAEVE..
tr|A0A354C796|A0A354C796_9DELT           QYLDGKVKKKALSPFQRDLTFSRITGCTDYRGFDKADLIIEAVFEDLDIKRKILAEAE..
tr|A0A3M1NUM8|A0A3M1NUM8_9BACT           DNLDGRVKKRALSAFERDKIMSRIHGVTDYHGFEKANLVIEAVFEDLQIKQQVLAEVE..
tr|A0A3M2KRC8|A0A3M2KRC8_9BACT           RELDRRVRRRALRPFERDRIMERLTPVLDYAPFRKVPLVIEAVFEDLAIKHQVLQEVE..
tr|A0A5S9IHR0|A0A5S9IHR0_9BACT           KDIGVKCKKRIYTAFQKDTILSKVFPTDSYKSLRNTDVVIEAVFEDLDLKRKVLAETE..
tr|A0A6M1ST81|A0A6M1ST81_9BACT           EDLDTKVSKRIISSFERDRISGLVTPTDSYKGFENVDLVIEAVFEDLDLKKKIVKEVE..
tr|A0A521AAE5|A0A521AAE5_9BACT           KGLEKKRKKGIISSFERDRLSSLITSTADYKGFEKADLVIEAVFEDLDLKRSILQEVE..
tr|A0A1M4ZMD6|A0A1M4ZMD6_9BACT           RDLEKKRKKKIISSFERDRISSLVTATDSYDGFANVGLVIEAVFEDLELKQSILREVE..
tr|A0A2A2GCS2|A0A2A2GCS2_9BACT           DNLDKKRKKRIISEFERDRIASHVTPTGTYDGFENVGLVIEAVFEDLNLKKSIIKEVE..
tr|A0A5D3YIH7|A0A5D3YIH7_9BACT           KSLEKNRKKHIISSFERDRISSLVTPTETYDGFENTDMVIEAVFEDLDLKQSILEEVE..
tr|A0A6M1T9G0|A0A6M1T9G0_9BACT           QRLEKKRKKHIISSFERDRISSLVTPTETYKGFKNTDLVIEAVFEDLDLKQSILQEVE..
tr|A0A6A8Q1Y8|A0A6A8Q1Y8_9BACT           KPLNKKVQKGIISEFERDRLASMVTGVDSYDGFEAVDLVIEAVFEDLELKKSIVREIE..
tr|A0A521BVJ3|A0A521BVJ3_9BACT           KALNEKVDKRIISEFERDKTAAKVTGTDSYEGFDDVELVIEAVFEDLDLKRNIIQEVE..
tr|A0A359E0Y8|A0A359E0Y8_9BACT           KSLNEKADKKIISEFERDQTASKVTGVDSYDGFNSVDVVIEAVFEDLDLKKNIVKQIE..
tr|A0A2D8CIM6|A0A2D8CIM6_9BACT           KSLNEKADKKIISEFERDQTASKVTGVDSYDGFNSVDVVIEAVFEDLDLKKNIVKQIE..
tr|A0A2D9FWF5|A0A2D9FWF5_9BACT           KSLNDKVNKKITSEFERDNTMSKVTGTDSYEGFGAVDLVIEAVFEDLELKQNIVQEIE..
tr|A0A1B6YB70|A0A1B6YB70_9BACT           EDLNKKSEKRIITKFERDEIASRVTGVDSYAGFDGLNLVIEAVFEDLNLKHKIIKEVE..
tr|A0A2D4ZTC0|A0A2D4ZTC0_9BACT           EDLNKKARKRIITKFERDEVASRVTGIDSYTGFDGVDLVIEAVFEDLDLKHKIIKEVE..
tr|A0A3D4UVW7|A0A3D4UVW7_9BACT           EDLNKKARKRIITKFERDEVASRVTGIDSYTGFDGVDLVIEAVFEDLDLKHKIIKEVE..
tr|A0A3F3I3M2|A0A3F3I3M2_9BACT           EDLNKKARKRIITKFERDEVASRVTGIDSYTGFDGVDLVIEAVFEDLDLKHKIIKEVE..
tr|A0A3D1G7L9|A0A3D1G7L9_9BACT           DELNKKSKKRIITPFERDEIASRLTGADSYDAFGKTDMVIEAVFEDLDLKRKIVAEVE..
tr|A0A3M8G1Y8|A0A3M8G1Y8_9BACT           DGLNKKASKRIITRFERDQIASRVTGTDSYNVFGKADLVIEAVFEDLDLKRRIVAEVE..
tr|A0A5Q4F391|A0A5Q4F391_9BACT           ENLLSKVEKRILNPMDRDRQLSRIHTTDSYDGFGRIDLVIEAVFEDLELKRSIVKEVQ..
tr|A0A6I7NPV0|A0A6I7NPV0_9BACT           NNLDQKVGKRILSSYERDEQMSRVFPTISYNGFNKMDLVIEAVFEDLELKRQIVKEVE..
tr|A0A651G1J2|A0A651G1J2_9BACT           ENLNQKVNKRILSRFERDEKLSLIHATDTYNGFDKIDVVIEAVFEDLSLKQSIVEDVE..
tr|A0A371QRK5|A0A371QRK5_9BACT           ENLQEKIDKKIISSFEKDELMSRIHPTTSYENFDQIDLVIEAVFEDLELKQKIVKEVE..
tr|A0A2N0VGJ5|A0A2N0VGJ5_9BACT           KNLDKKVSKHILSRFERDEIASLIHPTETYNGFEHIDLVIEAVFEDLDLKQSIVEEIE..
tr|A0A316TYJ0|A0A316TYJ0_9BACT           KNLEKKVSKRILSSFEKDEKMSLIHPTTTYNGFENIDLVIEAVFEDLDLKQKIVNEVE..
tr|A0A651GLZ8|A0A651GLZ8_9BACT           KNLNQKVGKRILSGFERDERMSLVHPTVSYDGFDKIDLVIEAVFEDLELKQNIVKDVE..
tr|A0A7Y5V3S5|A0A7Y5V3S5_9BACT           AVLQERVRKRQMTRQEMADRLSLAGGTADYSGFGAVDLVIEAVFEDLAVKHAVLREVE..
tr|A0A7W1SHC0|A0A7W1SHC0_9BACT           AVLQERLKKKQLTRGQVTDMMVLAGGSTDYSGFEQADLVIEAVFEDLALKHRVLRDVE..
tr|A0A7Y5PBT7|A0A7Y5PBT7_9BACT           EVLRGRVKKKQITRLQYDDQLSLAGGTVDYAGFGRATLVIEAVFEDLSLKHRVLREVE..
tr|A0A7Y5TXL8|A0A7Y5TXL8_9BACT           EVLRGRVKKKQITRLQYDDQLSLAGGTVDYAGFGRATLVIEAVFEDLSLKHRVLREVE..
tr|A0A423PQ96|A0A423PQ96_9GAMM           QFYARRVAKKAMTRAQADRCLHQVTGTTDYSGIGACELVVEAVFEDLDLKRAMLADVEAA
tr|U2G066|U2G066_9GAMM                   AFYDKRVHNKAMTRAAADRYLYQVTATTDYSGIGAADIVIEAVFEDLELKQAMLADVEAA
tr|A0A2E0J1F4|A0A2E0J1F4_9GAMM           AFYAKRVAKKAMTRAQADRCLHQVTGTTNYSGIGACDIVIEAVFEDLELKQQMLADVEAA
tr|A0A2D4SCM8|A0A2D4SCM8_9GAMM           GFYEKRVKRKAMSRYDAERDLRRMTGTTGYDGFKGADMVIEAVFEDLELKHRMLADVE..
tr|L0WJH1|L0WJH1_9GAMM                   DFYNKRVKKGAMSAADASAELGRASFTSDYSGFKGLDLVVEAVFEDVKLKHLMLKDVE..
tr|A0A1H5XF61|A0A1H5XF61_9GAMM           EFYNKRIKKGAMSAADASTEQGRISYTADYSGFKGLDLVVEAVFEDLGLKHRMLKDVE..
tr|A0A2E9TS48|A0A2E9TS48_9GAMM           EFYNKRAKKGAMTAADASAEQGRISYTADYSGFKGLDLVVEAVFEDLDLKHRMLKDVE..
tr|A0A095UE19|A0A095UE19_9GAMM           EFYNKRAKKGAMSAADASAEQGRISYTADYSGFKGLDLVVEAVFEDLGLKHKMLKDVE..
tr|A0A7G2S9A0|A0A7G2S9A0_9GAMM           EFYNKRAKKGAMSAADASAEQGRISYTADYSGFKGLDLVVEAVFEDLGLKHKMLKDVE..
tr|B5JU27|B5JU27_9GAMM                   KYYDKRAKLGAMSQFDAEAAKRQVTYTDQYTGFKRLDLVVEAVFEDLGLKHQMLKDTE..
tr|A0A1Y0IHP1|A0A1Y0IHP1_9GAMM           KFYDKRVKLGAMTAFDAQAKQAQMTVTEDYRGFAHADLVIEAVFEDLALKHQMLKDTE..
tr|A0A316FZ14|A0A316FZ14_9GAMM           SLIKKKMKRKQYSKIEAMKVQSRLTGGTDYKGFKQVDLVIEAVFEDLSLKHQMIKDVE..
tr|A0A498C231|A0A498C231_9GAMM           GEIHARKKRGALTDFEATRQRTRVTPALDYRGFTALDVVIEAVFEDLELKHRMLADVE..
tr|Q0A6T4|Q0A6T4_ALKEH                   GEIRARHKRGALTDFEATRQRTRVTPTLDYRGFGALDVVIEAVFEDPELKHRMLADVE..
tr|A0A3E0WL10|A0A3E0WL10_9GAMM           DTIAKRVERRSITKIEGQIEGNRVTPTLDYSGFKNADMVIEAVFEDLGLKHKMVQEIE..
tr|A0A3S1BU16|A0A3S1BU16_9GAMM           RLIQKRLQRRSVTAFEAAQEQGRVTPTLDFSGFHNLDLVIEAVFEDLELKHRMLADVE..
tr|A0A7V8QFN9|A0A7V8QFN9_9GAMM           DEVTKKVKRKSISAIEGDIIARRVTGTLDFSGFRNLDMVIEAVFEKLDLKHQMIRDIE..
tr|A0A1H8PPF9|A0A1H8PPF9_9GAMM           DQLQEKVKRRSMTAFEADVQRNRVTPTLDYSGFSGVDIAIEAVFEDLDLKHRMVQDIE..
tr|A0A6H0J0V7|A0A6H0J0V7_9GAMM           GEIQAKVKRRSLTPFEADVQLNRVTPTLDYSGFNGIDMAIEAVFEDLDLKHRMVRDVE..
tr|V5EZX6|V5EZX6_9VIBR                   KLLEKQRKRRILSKAQVQSKMSQLRGGTDYLGFNKADVVIEAVFEDLSLKQGMVQDIE..
tr|A0A6I1QBM2|A0A6I1QBM2_9VIBR           KLLEKQRKRRILSKAQVQSKMSQLRGGTDYLGFNKADVVIEAVFEDLSLKQGMVQDIE..
tr|A0A511QRL5|A0A511QRL5_9VIBR           KLLNKQRKRRILSKAQLQFKMSQLRGGTDYLGFNKAQVVIEAVFEDLALKQSMVKEIE..
tr|A0A7Y0JZP4|A0A7Y0JZP4_9VIBR           KLLNKQRKRRILSKAQLQSKMSQLRGGTDYLGFNKAQVVIEAVFEDLALKQSMVKEIE..
tr|A0A193KD24|A0A193KD24_9VIBR           KLLNKQRKRRILSKAQLQSKMSQLRGGTNYLGFNKAQVVIEAVFEDLALKQSMVKEIE..
tr|A0A1S1HM72|A0A1S1HM72_PROST           DRLTQRVNKRRMPARERDAVMAKISGTLDYQGFEHTDIVVEAVFEDLALKQKMVTEIQ..
tr|A0A7T8I613|A0A7T8I613_9GAMM           DRLTQRVNKRRMPARERDAVMAKISGTLDYQGFEHTDIVVEAVFEDLALKQKMVAEIQ..
tr|A0A140NIF0|A0A140NIF0_PROSM           DLLTQRVNKRRMLARERNAVMAKISGTLNYQGFEHADIVVEAVFEDLSLKQKMVAEVQ..
tr|A0A379GMB7|A0A379GMB7_PROST           DLLTQRVNKRRMLARERNAVMAKISGTLNYQGFEHADIVVEAVFEDLSLKQKMVAEVQ..
tr|A0A379H393|A0A379H393_PROST           DLLTQRVNKRRMLARERNAVMAKISGTLNYQGFEHADIVVEAVFEDLSLKQKMVAEVQ..
tr|B2Q0L5|B2Q0L5_PROST                   DLLTQRVNKRRMLARERNAVMAKISGTLNYQGFEHADIVVEAVFEDLSLKQKMVAEVQ..
tr|A0A6I3JUE9|A0A6I3JUE9_9GAMM           DLLTQRVNKRRMLARERSAVMAKISGTLTYQGFEHADIVVEAVFEDLALKRKMVTEVQ..
tr|A0A7D4P594|A0A7D4P594_YERMW           DALGKRVRSKRMRPAERQRQMMLISGSTDYSGFDTVDVVIEAVFEDLSLKQQMVADIE..
tr|A0A7U7IVK6|A0A7U7IVK6_YEREN           DTLGKRVRSKRMRPAERQRQMMLISGSTDYCGFGNVDIVVEAVFEDLSLKQQMVADIE..
tr|A0A447RIF5|A0A447RIF5_YEREN           DTLGKRVRSKRMRPAERQRQMMLISGSTDYCGFGNVDIVVEAVFEDLSLKQQMVADIE..
tr|A0A7H4ZLV9|A0A7H4ZLV9_YERP4           DTLGKRVRSKRMRPAERQRQMMLISGSTDYCGFGNVDIVVEAVFEDLSLKQQMVADIE..
tr|A0A0H3NX46|A0A0H3NX46_YERE1           DTLGKRVRSKRMRPAERQRQMMLISGSTDYCGFGNVDIVVEAVFEDLSLKQQMVADIE..
tr|A0A7T9XUV7|A0A7T9XUV7_YEREN           DTLGKRVRSKRMRPAERQRQMMLISGSTDYCGFGNVDIVVEAVFEDLSLKQQMVADIE..
tr|A0A7U7IYV8|A0A7U7IYV8_YEREN           DTLGKRVRSKRMRPAERQRQMMLISGSTDYCGFGNVDIVVEAVFEDLSLKQQMVADIE..
tr|A0A0H5G7G2|A0A0H5G7G2_YEREN           DTLGKRVRSKRMRPAERQRQMMLISGSTDYCGFGNVDIVVEAVFEDLSLKQQMVADIE..
tr|A0A2A7TDI2|A0A2A7TDI2_YERKR           DALGKRVRSKRMRPAERQRQMMLISGSTDYCGFSNVDIVIEAVFEDLTLKQQMVADIE..
tr|A0A0T9M261|A0A0T9M261_YERKR           DALGKRVRSKRMRPAERQRQMMLISGSTDYCGFSNVDIVIEAVFEDLTLKQQMVADIE..
tr|A0A0A0CMP1|A0A0A0CMP1_PHOLU           DLLSKRVKQKRLRPAERAQQMMLISGATDYRGFAQADIVVEAVFEDLSLKQQMVAEIE..
tr|A0A329VG55|A0A329VG55_9GAMM           DLLSKRVKQKRLRPAERAQQMMLISGTTDYRGFAQTDIVVEAVFEDLSLKQQMVAEIE..
sp|Q7N288|FADJ_PHOLL                     DLLSKRVKQKRLRPAERAQQMMLISGTTDYRGFAQTDIVVEAVFEDLSLKQKMVAEIE..
tr|A0A6L9JMX5|A0A6L9JMX5_PHOLM           DLLSKRVKQKRLRPAERAQQMMLISGTTDYRGFAQTDIVVEAVFEDLSLKQKMVAEIE..
tr|A0A7X5HQ29|A0A7X5HQ29_PHOLM           DLLSKRVKQKRLRPAERAQQMMLISGTTDYRGFAQTDIVVEAVFEDLSLKQKMVAEIE..
tr|A0A1C0U051|A0A1C0U051_9GAMM           DLLSRRVKQKRLRPVERDQQMMLISGTTGYRGFEQVDIVVEAVFEDLSLKQQMVAEIE..
tr|A0A7X5QF62|A0A7X5QF62_9GAMM           DLLSKRVKQKRLRPAERAKQMMLISGTTDYRGFEQVDIVVEAVFEDLSLKQQMVAEIE..
```

```
tr|A0A2D0KWM9|A0A2D0KWM9_9GAMM  DMLSKRVSQRRLTSSERSRQMSLLSGTTDYSGFKQADIVVEAVFEDLVLKRKMVAEVE..
tr|A0A2D0LAI1|A0A2D0LAI1_9GAMM  DMLSKRVSQRRLTSSERSRQMSLLSGTTDYSGFKQADIVVEAVFEDLVLKRKMVAEVE..
tr|A0A2D0IXC8|A0A2D0IXC8_XENBU  DLLSKRVKQRRLKPSERSRQMSLISGSTDYSGFKQVDIVVEAVFEDLVLKRKMVSEIE..
tr|W1JAM5|W1JAM5_9GAMM          DLLSKRVKQRRLKPSECSRQMSLLSGSTDYIGFKQADIVVEAVFEDLVLKRKMVSEIE..
tr|A0A3D9UED3|A0A3D9UED3_9GAMM  DLLSKRVKQRRLKPLECSRQMSLLSGSTDYIGFKQADIVVEAVFEDLVLKRKMVSEIE..
tr|A0A1I3JCA9|A0A1I3JCA9_9GAMM  DLLTKRVKQRRLSPGERSRQMSLLSGTTDYSGFKQADIVVEAVFEDLVLKRKMVSEIE..
tr|A0A068QUL9|A0A068QUL9_9GAMM  DRLSKRVKQRRLKSGERTRQMSLLSGSTDYSGFQQADIVVEAVFEDLVLKRKMVAEIE..
tr|A0A0M0TCH6|A0A0M0TCH6_9GAMM  DLLSKRVKQRRLQSSERSRQMSLLTGTTDYSGFKQADIVVEAVFEDLVLKRKMVAEIE..
tr|A0A1Q5U854|A0A1Q5U854_9GAMM  DLLSKRVKQRRLKSSERSRQMSLLTGTTDYSGFKQADIIVEAVFEDLVLKRKMVAEIE..
tr|A0A2D0ISP1|A0A2D0ISP1_9GAMM  DLLSKRVKQRRLKSSERSRQMSLLTGTTDYSGFKQADIIVEAVFEDLVLKRQMVAEIE..
tr|A0A1Q5TUI8|A0A1Q5TUI8_9GAMM  DLLSKRVKQRRLKSSERSHQMSLLTGTTDYSGFKQTDIVVEAVFEDLVLKRKMVAEIE..
tr|A0A2D0KJI7|A0A2D0KJI7_9GAMM  DLLSKRVKQRRLKSSERSHQMSLLTGTTDYSGFKQVDIVVEAVFEDLVLKRKMVAEIE..
tr|A0A1Y2SB97|A0A1Y2SB97_9GAMM  DLLSKRVKQRRLKSSERSRQMALLTGTMDYSGFKQTDIVVEAVFEDLVLKRKMVSEIE..
tr|A0A1I5DWI7|A0A1I5DWI7_9GAMM  DLLSKRVKQRRLKSSERSRQMSLLTGTIDYSGFEQADIVVEAVFEELVLKRKMVSEIE..
tr|A0A1I7GWY6|A0A1I7GWY6_9GAMM  DLLSKRVKQHRLKPSERSRQMSLLTGTTDYSGFKQADIVVEAVFEDLILKRKMVSEVE..
tr|D3VKY8|D3VKY8_XENNA          DLLSKRVKQRRLKPSERSRQMSLLTGTTDYSGFKQADIVVEAVFEDLILKRKMVSEVE..
tr|A0A2G0Q6Q9|A0A2G0Q6Q9_9GAMM  DLLSKRVKQRRLKSAERSRQMSLLTGTTDYSGFKQADIVVEAVFEDLVLKRKMVSEIE..
tr|A0A2D0JU24|A0A2D0JU24_9GAMM  DLLSKRVKQRRLKPSERSQQMSLLTGTTDYSGFKQADIVVEAVFEDLVLKRKMVSEIE..
tr|A0A0J5FN38|A0A0J5FN38_9GAMM  DLLSKRVKQRRLKPSERSQQMSLLTGTTDYSGFKQADIVVEAVFEDLVLKRKMVSEIE..
tr|A0A432XLD4|A0A432XLD4_9GAMM  DLLNKKVKRKHMARAELEATMLRLSGSLDYTGFDQADVVIEAVFEDLNLKQQMVADVE..
tr|A0A2D8HU84|A0A2D8HU84_9GAMM  ERLNKKVKRRHMRRAELEKTMLMLSGSLDYSGFERTDVVIEAVFEDLNLKQKMVADVE..
tr|A0A656X1Q2|A0A656X1Q2_9GAMM  ERLNKKVKRRHMRRAELEKTMLMLSGSLDYSGFERTDVVIEAVFEDLNLKQKMVADVE..
tr|A0A4Q1QH79|A0A4Q1QH79_9GAMM  ERLIKKVKRKHMRRAEYEKTMLMLSGTLDYSGFERTDVVIEAVFEDLELKQKMVADIE..
tr|A0A1G7LQE6|A0A1G7LQE6_9GAMM  ERLIKKVKRKHMRRAEYEKTMLMLSGTLDYSGFERTDVVIEAVFEDLDLKQKMVADIE..
tr|A0A432YVG7|A0A432YVG7_9GAMM  ERLIKKVKRKHMRRAEYEKTMLMLSGTLDYSGFERTDVVIEAVFEDLDLKQKMVADIE..
tr|A0A1J5N0P5|A0A1J5N0P5_9GAMM  DLLDKKLKRRFITRSELQSKMNNLTGTVAYRGVKDVDMVVEAVFEDLDLKHQMVKDVE..
tr|A0A0D8D548|A0A0D8D548_9GAMM  DLLNKKVKRRFMMHSEMQKQMSSITGTVDYSGFKELDIVVEAVFEDLALKQKMVADVE..
tr|A0A3E0TNM2|A0A3E0TNM2_9GAMM  DLLNKKVKRRFMRHSEMQKQMSAITGTLDYSGFNDADIVVEAVFEDLPLKQKMVADVE..
tr|A0A3E0UD67|A0A3E0UD67_9GAMM  DLLNKKVKRRFMRHSEMQKQMSAITGTLDYSGFNDTDIVVEAVFEDLSLKQKMVADVE..
tr|A0A3E0U0P4|A0A3E0U0P4_9GAMM  DLLNKKVKRRFMRHSEMQKQMSAITGTLDYSGFNDTDIVVEAVFEDLSLKQKMVADVE..
tr|A0A0M2V8B1|A0A0M2V8B1_9GAMM  KLLEKKFKRRFISKAEMQKQLLLLTGSTDYSGFHDVDLVIEAVFEDLDLKQKMVADIE..
tr|A0A285ITY5|A0A285ITY5_9GAMM  DLLQKKFKRRFISKAEMQKQLLLLTGSTDYSGFHDVDFVIEAVFEDLALKQQMVADIE..
tr|A0A486XTB9|A0A486XTB9_9GAMM  SLLEKKFKRRFITKAQMQQQLLLMTGSTDYSGFHDADIVVEAVFEDLALKQQMVADIE..
tr|I1E1G1|I1E1G1_9GAMM          SLLEKKFNRRFISKAEMQKQLLLITGTTDYSGFHDADIVVEAVFEDLALKQQMVADIE..
tr|A0A1H6KJ68|A0A1H6KJ68_9GAMM  SLLEKKFKRRFISKAEMQKQLLLITGTTDYSGFHDADIVVEAVFEDLALKQQMVADIE..
tr|A0A0X3Y764|A0A0X3Y764_9GAMM  SLLEKKFKRRFISKAEMQKQLLLITGTTDYSGFHDADIVVEAVFEDLALKQQMVADIE..
tr|A0A2N1YEV4|A0A2N1YEV4_9GAMM  SLLEKKFKRRFISKAEMQKQLLLITGTTDYSGFHDADIVVEAVFEDLALKQQMVADIE..
tr|A0A3P3QCM3|A0A3P3QCM3_9GAMM  SLLEKKLRKRFVTKTEVQKQMSLLTGTTDYSGFHDVDLVVEAVFEDLNLKQQMVADIE..
tr|F7NT72|F7NT72_9GAMM          SLLEKKLRKRFVTKTEVQKQMSLLTGTTDYSGFHDVDLVVEAVFEDLNLKQQMVADIE..
tr|A0A3S2TW63|A0A3S2TW63_9GAMM  SLLEKKLRKRFVTKTEVQKQMSLLTGTTDYSGFHDVDLVVEAVFEDLNLKQQMVADIE..
tr|A0A3D5BGW7|A0A3D5BGW7_9GAMM  SLLNKKFKRRYISRTEMQKQMALLTGTTDYSGFHDVDLVVEAVFEDLALKQQMVKDIE..
tr|A0A5C7TI17|A0A5C7TI17_9GAMM  SLLNKKYKKRFISKTELQKQMSLLTGTTDYSGFHDVDLVVEAVFEDLALKQQMVADIE..
tr|A0A0U4W982|A0A0U4W982_9GAMM  SLLNKKFKKRFISKTELQKQMALLTGTTDYSGFHDVDLVIEAVFEDLALKQQMVADIE..
tr|A0A2I0FCM2|A0A2I0FCM2_9GAMM  DILNKKVKRRFMSKAEMQSQLAMITGTTEYTGVKSADIVIEAVFEDLKLKHQMVADIE..
tr|A0A2G2IVL1|A0A2G2IVL1_9GAMM  DILNKKVKRRFMSKAEMQSQLAMITGTTEYTGVKNADIVIEAVFEDLKLKHQMVADIE..
tr|A6FI21|A6FI21_9GAMM          DILNKKVKRRFMSKAEMQSQLAMITGTTEYTGVKNADIVIEAVFEDLKLKHQMVADIE..
tr|A0A4U1BNR2|A0A4U1BNR2_9GAMM  KQLDKKVKRRHILESERDKTMSLLTGTLDYQGAQSIDMVIEAVFEDLKLKHQMVKDVE..
tr|E1SQ60|E1SQ60_FERBD          KQVDKRVKKRQMTPAQRDKAMSLLSTTTRYDGIKDVDIVVEAVFEDLNLKHQMVKDVE..
tr|A0A4Y6IZ09|A0A4Y6IZ09_9GAMM  KLLAKGVKRRHMTPAVRDNLMALMTTTTEYKGIKDADMVVEAVFEDLNLKHQMVKDVE..
tr|A0A0C3QSZ7|A0A0C3QSZ7_9GAMM  KLLAKGVKRRHMTPAVRDNLMALMTTTTEYKGIKDADMVVEAVFEDLKLKHQMVQDVE..
tr|A0A6L7HXW0|A0A6L7HXW0_9GAMM  KLLAKGVKRRHMTPAVRDNLMALMTTTTEYKGIKDADMVVEAVFEDLNLKHQMVQDVE..
sp|A3QFP3|FADJ_SHELP            KLLAKGVKRRHMTPAVRDNLMALMTTTTEYKGIKDADMVVEAVFEDLNLKHQMVKDVE..
tr|A0A1E5IXH7|A0A1E5IXH7_SHECO  KLLDKGVKRRHMTSAARDNLMALMTTTTEYKGIKDADIVVEAVFEDLGLKHQMVKDVE..
tr|A0A411PKQ0|A0A411PKQ0_9GAMM  KLLAKGVKRRHMTPAVRDNLMALMTTTTEYKGVKDADIVVEAVFEDLSLKHQMVKDVE..
tr|A0A6G9QKM3|A0A6G9QKM3_9GAMM  KLLDKGVKRRHMTPAVRDNIMSLMTTTTEYKGVKDADIVVEAVFEDLALKHQMVKDVE..
tr|A0A6P1UL63|A0A6P1UL63_9GAMM  KLLDKGVKRRHMTPIARDNIMALMTTTTEYTGIKDADIVVEAVFEDLALKHKMVQDVE..
tr|A0A2N1ERI9|A0A2N1ERI9_9GAMM  KLLDKGVKRRHMTPIARDNIMALMTTTTEYTGIKDADIVVEAVFEDLALKHKMVQDVE..
tr|A0A7W4FU55|A0A7W4FU55_9GAMM  KLLDKGVKRRHMTPIARDNIMALMTTTTEYTGIKDADIVVEAVFEDLALKHKMVQDVE..
sp|Q8ECP7|FADJ_SHEON            KLLDKGVKRRHMTPAARDNLMALMTTTTEYKGVKDADIVVEAVFEDLALKHQMVKDIE..
tr|A0A501XZY8|A0A501XZY8_9GAMM  KLLDKGVKRRHMTPAARDNLMALMTTTTEYKGVKDADIVVEAVFEDLALKHQMVKDIE..
tr|A0A2W5DCZ0|A0A2W5DCZ0_SHEOE  KLLDKGVKRRHMTPAARDNLMALMTTTTEYKGVKDADIVVEAVFEDLALKHQMVKDIE..
tr|A0A1E3V3C8|A0A1E3V3C8_9GAMM  KLLDKGVKRRHMTPAARDNLMALMTTTTEYKGVKDADIVVEAVFEDLALKHQMVKDIE..
tr|A0A1Z4AI20|A0A1Z4AI20_9GAMM  KLLDKGVKRRHMTPAARDNLMALMTTTTEYKGVKDADIVVEAVFEDLALKHQMVKDIE..
tr|A0A7X9LJL9|A0A7X9LJL9_9GAMM  KLLDKGVKRRHMTPAARDNLMALMTTTTEYKGVKDADIVVEAVFEDLALKHQMVKDIE..
tr|A0A073KMY5|A0A073KMY5_9GAMM  KLLDKGVKRRHMTPAARDNLMALMTTTTEYKGVKDADIVVEAVFEDLALKHQMVKDIE..
sp|A0KV76|FADJ_SHESA            KLLDKGVKRRHMTPAVRDNLMALMTTTTEYKGVKDADIVVEAVFEDLALKHQMVKDIE..
tr|A0A220UTH7|A0A220UTH7_9GAMM  KLLDKGVKRRHMTPAVRDNLMALMTTTTEYKGVKDADIVVEAVFEDLALKHQMVKDIE..
tr|A0A5B8R6W1|A0A5B8R6W1_9GAMM  KLLDKGVKRRHMTPAVRDNLMALMTTTTEYKGVKDADIVVEAVFEDLALKHQMVKDIE..
tr|V1DAI4|V1DAI4_9GAMM          KLLDKGVKRRHMTPAVRDNLMALMTTTTEYKGVKDADIVVEAVFEDLALKHQMVKDIE..
tr|A0A448CPQ4|A0A448CPQ4_SHEPU  KLLDKGVKRRHMTPAVRDNLMALMTTTTEYKGVKDADIVVEAVFEDLALKHQMVKDIE..
tr|A0A252ERQ3|A0A252ERQ3_SHEPU  KLLDKGVKRRHMTPAVRDNLMALMTTTTEYKGVKDADIVVEAVFEDLALKHQMVKDIE..
sp|Q0HKD1|FADJ_SHESM            KLLDKGVKRRHMTPAVRDNLMALMTTTTEYKGVKDADIVVEAVFEDLALKHQMVKDIE..
sp|Q0HWN3|FADJ_SHESR            KLLDKGVKRRHMTPAVRDNLMALMTTTTEYKGVKDADIVVEAVFEDLALKHQMVKDIE..
tr|F7RQE3|F7RQE3_9GAMM          KLLDKGVKRRHMTPAARDNLMALMTTTTEYKGVKDADIVVEAVFEDLALKHQMVKDIE..
tr|B8EE98|B8EE98_SHEB2          KLLDKGVKRRHMTPAARDNLMALMTTTTEYKGVKDADIVVEAVFEDLALKHQMVKDIE..
sp|A6WQ25|FADJ_SHEB8            KLLDKGVKRRHMTPAARDNLMALMTTTTEYKGVKDADIVVEAVFEDLALKHQMVKDIE..
tr|A0A448EK41|A0A448EK41_9GAMM  KLLDKGVKRRHMTPAARDNLMALMTTTTEYKGVKDADIVVEAVFEDLALKHQMVKDIE..
tr|A0A553JHX1|A0A553JHX1_SHEHA  KLLDKGVKRRHMTPAVRDNLMSLMTTTTEYKGIKDADIVVEAVFEDLDLKHQMVKDVE..
tr|B8CPY6|B8CPY6_SHEPW          KLLDKGVKRRHMTPAVRDNLMALMTTTTEYKGIKDADIVVEAVFEDLSLKHQMVKDVE..
tr|A0A431WFC4|A0A431WFC4_9GAMM  KLLDKGVKRRHMTPAVRDNLMSLMTTTTEYKGIKDADIVVEAVFEDLKLKHQMVKDVE..
tr|A8FTR7|A8FTR7_SHESH          KLLDKGVKRRHMTPAVRDNLMSLMTTTTEYKGIKDADIVVEAVFEDLKLKHQMVKDVE..
tr|A0A431WNL0|A0A431WNL0_9GAMM  KLLDKGVKRRHMTPAVRDNLMSLMTTTTEYKGIKDADIVVEAVFEDLKLKHQMVKDVE..
tr|A0A550AEC5|A0A550AEC5_9GAMM  KLLDKGVKRRHMTPAARDSLMSLMTTTTEYKGIKDADIVVEAVFEDLNLKHQMVKDVE..
tr|A0A7L4WW90|A0A7L4WW90_9GAMM  KLLDKGVKRRHMTPAARDSLMSLMTTTTEYKGIKDADIVVEAVFEDLNLKHQMVKDVE..
tr|A9DDU3|A9DDU3_9GAMM          KLLDKGVKRRHMTPSARDNIMSLMTTTTEYKGIKDADIVVEAVFEDLNLKHQMVKDVE..
tr|A0A330M2B9|A0A330M2B9_9GAMM  KLLDKGVKRRHMTPSARDNIMSLMTTTTEYKGIKDADIVVEAVFEDLNLKHQMVKDVE..
tr|A0A5N8UFC7|A0A5N8UFC7_9GAMM  KLLDKGVKRRHMTPSARDNIMSLMTTTTEYKGIKDADIVVEAVFEDLKLKHQMVKDVE..
tr|A0A1S6HN57|A0A1S6HN57_9GAMM  KLLDKGVKRRHMTPSARDNIMSLMTTTTEYKGIKDADIVVEAVFEDLNLKHQMVKDVE..
tr|D4ZMH7|D4ZMH7_SHEVD          KLLDKGVKRRHMTPSARDNIMSLMTTTTEYIGIKYADIVVEAVFEDLSLKHQMVKDVE..
tr|A0A3L8Q213|A0A3L8Q213_9GAMM  KQLDKKVKRRHITASARDNTMALMTTTTEYRGVKDADIVVEAVFEDLNLKHQMVKDIE..
tr|A0A3A6U4N9|A0A3A6U4N9_9GAMM  NLLDKKVKRRHMTANDRDNIMALMTTTTEYKGVQDADIVVEAVFEDLSLKHQMVKDIE..
tr|A0A4Q5MA37|A0A4Q5MA37_9GAMM  KLLDKKVKRRHMKSSERDATMALMTTTTEYKGVKDADIVVEAVFEDLNLKHQMVKDIE..
tr|A0A1L6LSX5|A0A1L6LSX5_9DELT  DILDEDVKKKKLTKLERDQKLALLSGTTDYAGMKSAYLVIEAVFEDLDVKHKVIREIE..
tr|A0A2W4L9B9|A0A2W4L9B9_9PROT  RVLDDRVRRKRITPVEREEVMARITGTTRYDGFSNVDLVIEAVFEDLELKHRVIREIE..
tr|A0A2W4M4E6|A0A2W4M4E6_9PROT  KILDGRVKSRRITPVERDRLMAQITPTTTYSGFREAELVIEAVFEDLELKQRVLSDVE..
tr|A0A6I2GRX9|A0A6I2GRX9_9DELT  GVLDERVKRRSLTWREAAAKSSLVTAGTDYAGFKGADLVIEAVFEDLALKHRVIAETE..
tr|A0A0H4WMK2|A0A0H4WMK2_9DELT  TILDERVKRRSLTRREATAKSAVVTAGTDYSGFKSADLVIEAVFEDLKLKHRVIAEVE..
tr|F8CJ36|F8CJ36_MYXFH          SILDERVKRRSLTRREATAKSALVTAGTDYAGFKSADLVIEAVFEDLKLKHRIIAEVE..
tr|A0A250K0F1|A0A250K0F1_9DELT  SILDERVKRRSLTRREATAKSALVTAGTDYAGFKSADLVIEAVFEDLKLKHRIIAEVE..
tr|A0A7Y6WFZ2|A0A7Y6WFZ2_9DELT  SILDERVKRRSLTRREATAKSALVTAGTDYSGFKSADLVIEAVFEDLKLKHRIIAEVE..
tr|A0A7Y7C660|A0A7Y7C660_9DELT  SILDERVKRRSLTRRDATAKSALVTAGTDYAGFKSADLVIEAVFEDLKLKHRIIAEVE..
tr|A0A7Y4JFH2|A0A7Y4JFH2_MYXXA  SILDERVKRRSLTRREATAKSALVTAGTDYSGFKSADLVIEAVFEDLKLKHRIIAEVE..
tr|A0A4Y6CZQ9|A0A4Y6CZQ9_MYXXA  SILDERVKRRSLTRREATAKSALVTAGTDYSGFKSADLVIEAVFEDLKLKHRIIAEVE..
```

```
tr|A0A7Y4IKV5|A0A7Y4IKV5_MYXXA  SILDERVKRRSLTRREATAKSALVTAGTDYSGFKSADLVIEAVFEDLKLKHRIIAEVE..
tr|A0A511HHB0|A0A511HHB0_9DELT  SILDERVKRRSLTRREATAKSALVTAGTDYSGFKSADLVIEAVFEDLKLKHRIIAEVE..
tr|A0A4Y6CKY7|A0A4Y6CKY7_MYXXA  SILDERVKRRSLTRREATAKSALVTAGTDYSGFKSADLVIEAVFEDLKLKHRIIAEVE..
tr|Q1D1F2|Q1D1F2_MYXXD          SILDERVKRRSLTRREATAKSALVTAGTDYSGFKSADLVIEAVFEDLKLKHRIIAEVE..
tr|A0A7Y4MA14|A0A7Y4MA14_MYXXA  SILDERVKRRSLTRREATAKSALVTAGTDYSGFKSADLVIEAVFEDLKLKHRIIAEVE..
tr|A0A7T8Y4N9|A0A7T8Y4N9_MYXXA  SILDERVKRRSLTRREATAKSALVTAGTDYSGFKSADLVIEAVFEDLKLKHRIIAEVE..
tr|L7UE67|L7UE67_MYXSD          GILEERVKRRSLTWREAAAKQAHITAGTDYSGFKSADLVIEAVFEDLKLKHRILAEVE..
tr|A0A511T9X1|A0A511T9X1_MYXFU  GILDERVKRRSLTWREAAAKQARITAGTDYSGFKSADLVIEAVFEDLKLKHRILAEVE..
tr|A0A7Y7C9C4|A0A7Y7C9C4_9DELT  GILDERVKRRSLTWREAAAKQARITAGTDYSGFKAADLVIEAVFEDLKLKHRILAEVE..
tr|A0A540X7W8|A0A540X7W8_9DELT  GILDERVKRRSLTWREAAAKQARITAGTDYSGFKAADLVIEAVFEDLKLKHRMLAEVE..
tr|A0A3A5FK19|A0A3A5FK19_9DELT  SVLDERVKRRSLTSREAVAKMAMVTAGTDYSGFKTADLVIEAVFEDLKLKHRIIAEVE..
tr|A0A3A8JQL9|A0A3A8JQL9_9DELT  TVLDERVKRRSLTPRESNAKLSNITAGTDYSGFKSVDLIIEAVFEDLKLKHQVIAEVE..
tr|A0A7Y4NFA4|A0A7Y4NFA4_9DELT  TVLDERVKRRSLTHREANAKLSNITAGTDYSGFKSVDLIIEAVFEDLKLKHQVIAEVE..
tr|A0A3A8HDQ7|A0A3A8HDQ7_9DELT  TVLDERVKRRSLTHREANAKLSNITAGTDYSGFKSVDLIIEAVFEDLKLKHQVIAEVE..
tr|A0A3A8GR90|A0A3A8GR90_9DELT  TVLDERVKRRSLTRLESNAKLSSITAGTDYSGFKSVDLVIEAVFEDLKLKHQIIAEVE..
tr|A0A3A8SBD7|A0A3A8SBD7_9DELT  TVLDERVKRRSLTRLESNAKLSNITAGTDYSGFKSVDLIIEAVFEDLKLKHQVIAEVE..
tr|A0A3A8T0I8|A0A3A8T0I8_9DELT  TVLDERVKRRSLTRLESNAKLSNITAGTDYSGFKSVDLIIEAVFEDLKLKHQVIAEVE..
tr|A0A7X5BU07|A0A7X5BU07_9DELT  TVLDERVKRRSLTRLESNAKLSNITAGTDYSGFKSVDLIIEAVFEDLKLKHQVIAEVE..
tr|A0A7Y1RVL2|A0A7Y1RVL2_9DELT  TVLDERVKRRSLTRLESNEKLSNITAGTDYSGFKSVDLIIEAVFEDLKLKHQVIAEVE..
tr|A0A3A8THN2|A0A3A8THN2_9DELT  TVLDERVKRRSLTRLESNAKLSNITAGTDYSGFKSVDLIIEAVFEDLKLKHQVIAEVE..
tr|A0A7Y1RX76|A0A7Y1RX76_9DELT  TVLDERVKRRSLTRLESNAKLSNITAGTDYSGFKSVDLIIEAVFEDLKLKHQVIAEVE..
tr|A0A3A8RA58|A0A3A8RA58_9DELT  TVMDERVKRRSLTRLESNAKLSNITAGTDYSGFKSVDLVIEAVFEDLKLKHKVIAEVE..
tr|A0A410RPB6|A0A410RPB6_CORCK  TVLDERVKRRSLTRLESNAKLSHITAGTDYSGFKSVDLVIEAVFEDLKLKHQVIAEVE..
tr|A0A7Y4J474|A0A7Y4J474_CORCK  TVLDERVKRRSLTRLESNAKLSHITAGTDYSGFKSVDLVIEAVFEDLKLKHQVIAEVE..
tr|A0A3A8I9Z6|A0A3A8I9Z6_9DELT  TVLDERVKRRSLTRLESNAKLSHITAGTDYSGFKSVDLVIEAVFEDLKLKHQVIAEVE..
tr|H8MKE9|H8MKE9_CORCM          TVLDERVKRRSLTRLESNAKLSNITAGTDYSGFKSVDLVIEAVFEDLKLKHQVIAEVE..
tr|A0A3A8H102|A0A3A8H102_9DELT  TVLDERVKRRSLTRLESNAKLSNITAGTDYSGFKSVDLVIEAVFEDLKLKHQVIAEVE..
tr|A0A554FW33|A0A554FW33_9DELT  TVLDERVKRRSLTHREASAKLANITAATDYSGFKSVDLIIEAVFEDLTLKHKVIAEVE..
tr|A0A3A8NPC0|A0A3A8NPC0_9DELT  TVLDERVKRRSLTHREASAKLSNISATTDYSGFKSVDLIIEAVFEDLKLKHQVIAEVE..
tr|A0A3A8JUV3|A0A3A8JUV3_9DELT  TVLDERVKRRSLTHREASAKFSNITAATDYSGFKSVDLVIEAVFEDLKLKHQVIAEVE..
tr|A0A3A8NEX6|A0A3A8NEX6_9DELT  TVLDERVKRRSLTHREASAKLSNITAATDYSGFKSVDLVIEAVFEDLKLKHQVIAEVE..
tr|A0A3A8JEU8|A0A3A8JEU8_9DELT  SVLDERVKRRSLTHRESSAKMSNITAGTDYSGFKSVDLVIEAVFEDLKLKHKVIAEVE..
tr|A0A3A8LI76|A0A3A8LI76_9DELT  TVLDERVKRRSLTHRESSAKMSNISAGTDYSGFKSVDLIIEAVFEDLKLKHKVIAEVE..
tr|A0A085WXN8|A0A085WXN8_9DELT  SVLDERVKRRSLTWREAAAKQALITAGTDYSGFKNVDLVIEAVFEDLKLKHRVIAETE..
tr|A0A2T4V0M5|A0A2T4V0M5_9DELT  SIYDERVKKRSLTWREAAAKMALVTGGTGYEGFKNVDVVIEAVFEDLALKHRIIGEVE..
tr|A0A0G2ZSW5|A0A0G2ZSW5_9DELT  GLYDERVKKRSLTWREAASKMALVTGGTGYEGFKSVDVVIEAVFEDLALKHRIMGEVE..
tr|A0A3M2DKY0|A0A3M2DKY0_9DELT  GIHDERVARRRSTPRERLEKMALITATTDYTGFADCDVVIEAVFEDLELKQRVLRDIE..
tr|A0A661NQ58|A0A661NQ58_9DELT  EIVDKRLERRRITKLDADRLMTMISGTTSYDGLGDAEIIIEAVFEDIELKHRVLRDTE..
tr|A0A520YD99|A0A520YD99_9DELT  GIIDGRVKRKRLTERQADALMLQTTGTTSYEGFKDIDVVIEAVFEDLSLKERMVRDVE..
tr|A0A7Y3BRE4|A0A7Y3BRE4_9DELT  GIIDGRVKRKRLTERQADALMLQTTGTTSYEGFKDIDVVIEAVFEDLSLKERMVRDVE..
tr|A0A2D9TF90|A0A2D9TF90_9DELT  DLLDGRVKRKKMTPMKADQLMMQISGATDYSGFADCNVVVEAVFEDLALKQRMVRDVE..
tr|A0A2E0TP32|A0A2E0TP32_9DELT  GIIDKRVQRKKMTPMDADELMLQTTGTVDYSGFHRCEVVIEAVFEDLELKRKMVKEVE..
tr|A0A2E4Y3V1|A0A2E4Y3V1_9PROT  GALNKRVKRRAMSSFERDKIFSSLTTQTDWTVFEKCPLVIEAVFEDLSLKQHILEEFE..
tr|A0A2E6VRH4|A0A2E6VRH4_9DELT  SVLNKKTKRKAISAFERDRLMSQVIGQVNYTGFENCELVVEAVFEDLALKHRVIQELE..
tr|A0A1F9FB59|A0A1F9FB59_9DELT  SDLDKRVRSHAMSPFERDRTFSGVVPKTDYRGFDKCELVIEAVFEDLALKHKIIREIE..
```

```
ECHA_HUMAN
                                      470       480       490       500       510       520
                                        .         .         .         .         .         .
ECHA_HUMAN                       ..AVIPDHCIFASNTSALPISEIAAVSKRPEKVIGMHYFSPVDKMQLLEIITTEKTSKDT
tr|A0A4R6XLA8|A0A4R6XLA8_9GAMM   ..KRCKKSTVFATNTSSLPLASIAAKAAHPQNVVGMHYFSPVPKMPLLELIVTDKTSKRA
tr|A0A6A0IJT2|A0A6A0IJT2_9BACT   ..EVLSPSAVFASNTSALPIREIAREAQMPERVVGMHYFSPVPKMPLLEVIAPEKASPEA
tr|A0A7V3QZU7|A0A7V3QZU7_9BACT   ..ALVPAECVFASNTSALPIASIAEKALHPERVLGMHYFSPVPKMPLLELVVAEKTAPWA
tr|A0A7V4SYJ6|A0A7V4SYJ6_9BACT   ..AVVSESCVFASNTSALPIRAIAEGARRPERVLGMHYFSPVPKMPLLEIVRAEKTSREA
tr|A0A2A8D2N6|A0A2A8D2N6_9BACT   ..AVISDECVYATNTSSIPISEIAEAADRPERVLGMHYFSPVGKMPLLEIVVTEDTSEEA
tr|A0A2N1TGP0|A0A2N1TGP0_9SPIR   ..ESTGDRTIFASNTSAIPITAIAAEASRRENVVGMHYFSPVPKMPLLEIITTKETAPWV
tr|A0A1G3QGW4|A0A1G3QGW4_9SPIR   ..EAADRDTIFASNTSALPITSIAKGCRRPQNVIGMHYFSPVPRMPLLEIIKTDKTAAWV
tr|A0A1G3QTY3|A0A1G3QTY3_9SPIR   ..AATGGDTIVASNTSALPIKSIARGCKRPQNVIGMHYFSPVPRMPLLEIIKTDTTADWV
tr|A0A7X9L359|A0A7X9L359_9DELT   ..AATGEDTIFASNTSALPITLIAEGSARPANVIGMHYFSPVPKMPLLEIITTEATADWV
tr|A0A522CJY0|A0A522CJY0_9SPIR   ..AATGDSCIFASNTSALPIGAIAEKAARPQNVIGMHYFSPVPQMPLLEIITTPKTAEWV
tr|A0A2N1RRJ3|A0A2N1RRJ3_9SPIR   ..SAAGDRTIFASNTSAIPITDIAKGCGRPANVIGMHYFSPVPKMPLLEIITTAKTAPWV
tr|A0A2N2KFN7|A0A2N2KFN7_9DELT   ..EAADENTIFASNTSAIPIADIAAGCRRPQNVIGMHYFSPVPRMPLLEIITTDKTAPWV
tr|A0A1V6AXX9|A0A1V6AXX9_9DELT   ..TATDERTIFASNTSAIPIHDIAEGCKRPENVIGMHYFSPVPKMPLLEIITTDKTAPWV
tr|A0A2N2HZL0|A0A2N2HZL0_9DELT   ..TATDERTIFASNTSAIPIQDIAAGCKRPENVIGMHYFSPVPKMPLLEIITTGKTAPWV
tr|A0A5E8ARP3|A0A5E8ARP3_9BACT   ..AAIRKDCVFASNTSALPITKIAEASSRPETVIGMHYFSPVPKMPLLEIVVTGKTADWV
tr|A0A662A7B1|A0A662A7B1_9BACT   ..KYIREDAIIATNTSALPIKAIAAVAKTPENVIGMHYFSPVPKMPLLEIIVHEKTADWV
tr|D7CV16|D7CV16_TRURR           ..AVAQEELIFATNTSSIPIREIAAASRRPEQVIGMHYFSPVPKLPLLEIIKTDATPDWV
tr|A0A3C2AKX4|A0A3C2AKX4_9FLAO   ..EHLSKDAIFASNTSALPITEIAKASKRPEQVIGMHYFSPVPKMPLLEIVVTDQTADWV
tr|A0A3B8ZLN3|A0A3B8ZLN3_9PLAN   ..QHMNDQAIFASNTSALPIKEIATASKRPEQVIDMHYFSPVPKMPLLEIIVTDQTADWV
tr|A0A345UGV8|A0A345UGV8_9BACT   ...AVSPDAIFASNTSSLPIADIAKGAKKPENVVGMHYFSPVQKMPLLEIIKTDQTSEEA
tr|A0A6C1P6A2|A0A6C1P6A2_9BACT   ..AVVPDHCIFASNTSSLPITDIAAGSKRPELVLGMHYFSPVQKMPLLEIIKTKKTTDRA
tr|A0A3M1X9W8|A0A3M1X9W8_9BACT   ..LETGEQTIFASNTSSLPIEKLAKIASRPTQVVGMHYFSPVPRMPLLEIVVTPKTEAWV
tr|A0A354C796|A0A354C796_9DELT   ..AHAKEEAIFASNTSSLPIRSIAAASLRPEQVIGMHYFSPVPKMPLLEIIITEKTADWV
tr|A0A3M1NUM8|A0A3M1NUM8_9BACT   ..AVTPKQCIFASNTSSIPISRIAEKAKRPKQVIGMHYFSPVPKMPLLEIIITPQTAQWV
tr|A0A3M2KRC8|A0A3M2KRC8_9BACT   ..AATEGKAIFASNTSALPIGDIAARARHPEQVIGMHYFSPVPKMPLLEVIVTPKTADWV
tr|A0A5S9IHR0|A0A5S9IHR0_9BACT   ..AVVRDDCIFASNTSSLPITDIAAEAKHPERVIGMHYFSPVPKMPLLEIITTDKTADWV
tr|A0A6M1ST81|A0A6M1ST81_9BACT   ..EYIPERCIFASNTSSLPIAKIAEASQRPEQVIGMHYFSPVPKMPLIEIIKTDETADWV
tr|A0A521AAE5|A0A521AAE5_9BACT   ..EVLPEHGIFASNTSSLPIRKIAEAAKRPGQVIGMHYFSPVPKMPLLEIITTDQTADWV
tr|A0A1M4ZMD6|A0A1M4ZMD6_9BACT   ..DRLPGKAIFASNTSSLPIRDIARAAQRPQQVIGMHYFSPVPKMPLLEIITTEQTADWV
tr|A0A2A2GCS2|A0A2A2GCS2_9BACT   ..EVTPDDYIFASNTSSLPITKIAKASRRPEQVVGMHYFSPVPKMPLLEIITTDQTADWV
tr|A0A5D3YIH7|A0A5D3YIH7_9BACT   ..QVTPDHCIFASNTSSLPINNIAEASGRPEQVVGMHYFSPVPKMPLLEIITTEKTADWV
tr|A0A6M1T9G0|A0A6M1T9G0_9BACT   ..EATSDHAIFASNTSSLPIEDIADAADRPEQVVGMHYFSPVPKMPLLEIITTDQTADWV
tr|A0A6A8Q1Y8|A0A6A8Q1Y8_9BACT   ..KKTADHCIFASNTSSLPISEIAKGAQNPENVIGMHYFSPVQKMPLLEIITTEQTADWV
tr|A0A521BVJ3|A0A521BVJ3_9BACT   ..AKTRDNCIFASNTSSLPISKIAEAANRPENVIGMHYFSPVQKMPLLEIIATEQTADWV
tr|A0A359E0Y8|A0A359E0Y8_9BACT   ..SSTPDHTIFASNTSSLPISKIAEGAKRPENIIGMHYFSPVQKMPLLEIITTEDTADWV
tr|A0A2D8CIM6|A0A2D8CIM6_9BACT   ..SSTPDHTIFASNTSSLPISKIAEGAKRPENIIGMHYFSPVQKMPLLEIITTEDTADWV
tr|A0A2D9FWF5|A0A2D9FWF5_9BACT   ..KNTGDDCIFASNTSSLPISKIAEGANRPENIIGMHYFSPVQKMPLLEIITTDKTADWV
tr|A0A1B6YB70|A0A1B6YB70_9BACT   ..EVTSDDTIFASNTSSLPISEIAKASSHPKNVIGMHYFSPVQKMPLLEIITTEQTADWV
tr|A0A2D4ZTC0|A0A2D4ZTC0_9BACT   ..DVTGENTIFASNTSSIPISEIAKASSRPQNVIGMHYFSPVQKMPLLEIITTSQTADWV
tr|A0A3D4UVW7|A0A3D4UVW7_9BACT   ..DVTGENTIFASNTSSIPISEIAKASSRPQNVIGMHYFSPVQKMPLLEIITTSQTADWV
tr|A0A3F3I3M2|A0A3F3I3M2_9BACT   ..DVTGENTIFASNTSSIPISEIAKASSRPQNVIGMHYFSPVQKMPLLEIITTSQTADWV
tr|A0A3D1G7L9|A0A3D1G7L9_9BACT   ..AATDDDCIFASNTSSLPISDIAQGAKRPENIIGMHYFSPVQKMPLLEIITTPQTADWV
tr|A0A3M8G1Y8|A0A3M8G1Y8_9BACT   ..AITSDRCIFATNTSSLPIVDIAEGAKRPENIIGMHYFSPVQKMPLLEIIITPQTADWV
tr|A0A5Q4F391|A0A5Q4F391_9BACT   ..RESGSSTIFASNTSSLPISEIAAGARYPGNIIGMHYFSPVQKMPLLEIIKTPKTKQWV
tr|A0A6I7NPV0|A0A6I7NPV0_9BACT   ..ESCSEDTIFASNTSSLPITEIAANAKRPENIIGMHYFSPVQKMPLLEIIKTKQTADWV
tr|A0A651G1J2|A0A651G1J2_9BACT   ..SAASETTIFASNTSSLPISDIAEKAKRPQNIIGMHYFSPVQKMPLLEIIKTDKTADWV
tr|A0A371QRK5|A0A371QRK5_9BACT   ..ENSSEHTIFASNTSSLPISDIAAKAKRPENILGMHYFSPVQKMPLLEIIKTEKTADWV
tr|A0A2N0VGJ5|A0A2N0VGJ5_9BACT   ..NHGSKDIIFASNTSSLPITDIAAKADRPENIIGMHYFSPVQKMPLLEIIKTEKTDDWV
tr|A0A316TYJ0|A0A316TYJ0_9BACT   ..SAGSENTIFASNTSSLPISDIAAKSERPEQIIGMHYFSPVQKMPLLEIIKTDKTADWV
tr|A0A651GLZ8|A0A651GLZ8_9BACT   ..QNGHEKTIFASNTSSLPITDIAAKAKRPENIIGMHYFSPVQKMPLLEIIKTEKTADWV
tr|A0A7Y5V3S5|A0A7Y5V3S5_9BACT   ..AVIPDHAIVASNTSTIPISRIADASHRPERVVGMHFFSPVHKMPLLEVITTPRTRPDV
tr|A0A7W1SHC0|A0A7W1SHC0_9BACT   ..PALRADAVYASNTSTIPIAEIAAAATHPERVLGMHFFSPVHKMPLLEVIATERTSKEA
tr|A0A7Y5PBT7|A0A7Y5PBT7_9BACT   ..PLLAPAAVYASNTSTIPITRIAEVASRPGRVLGMHFFSPVHKMPLLEVIVTPRTNSDA
tr|A0A7Y5TXL8|A0A7Y5TXL8_9BACT   ..PLLAPAAVYASNTSTIPITRIAEVASRPGRVLGMHFFSPVHKMPLLEVIVTPRTNSDA
tr|A0A423PQ96|A0A423PQ96_9GAMM   ADACANTDCIFATNTSSLPIAEIAAQAKRPENVIGLHYFSPVEKMPLLEIIATEQTSKKT
tr|U2G066|U2G066_9GAMM           ADAQGNPDCIFATNTSSLPISDIAARAKRPENVIGLHYFSPVEKMPLLEIIATDKTSKET
tr|A0A2E0J1F4|A0A2E0J1F4_9GAMM   ADARGNTDCIFATNTSSLPISEIAAKAKRPENVIGLHYFSPVEKMPLLEIIATEKTSKKT
tr|A0A2D4SCM8|A0A2D4SCM8_9GAMM   ..ALGNEQTIFASNTSSIPITDIAAKAQRPENVIGLHYFSPVEKMPLLEIIATEHTAPEV
tr|L0WJH1|L0WJH1_9GAMM           ..EHGNDQTIFASNTSSIPITEIARAAKRPQNVIGLHYFSPVEKMPLLEIITTDQTDPAV
tr|A0A1H5XF61|A0A1H5XF61_9GAMM   ..ANGNKHTVFASNTSSIPITEIASAAERPENVIGLHYFSPVEKMPLLEIITTDKTAPEV
tr|A0A2E9TS48|A0A2E9TS48_9GAMM   ..ANGNKHTVFASNTSSIPITDIASAAERPENVIGLHYFSPVEKMPLLEIITTDKTAPEV
tr|A0A095UE19|A0A095UE19_9GAMM   ..ANGNKHTVFASNTSSIPITDIASAAERPENVIGLHYFSPVEKMPLLEIITTDKTAPEV
tr|A0A7G2S9A0|A0A7G2S9A0_9GAMM   ..ANGNKHTVFASNTSSIPITDIASAAERPENVIGLHYFSPVEKMPLLEIITTDKTAPEV
tr|B5JU27|B5JU27_9GAMM           ..ANGNENTIFASNTSSIPISDIAKAAKRPENVIGLHYFSPVEKMPLLEIITTDKTAPEV
tr|A0A1Y0IHP1|A0A1Y0IHP1_9GAMM   ..SHGHQQTIFASNTSSIPISDIAKAAARPENVIGMHYFSPVEKMPLLEIITHEQTAPEV
tr|A0A316FZ14|A0A316FZ14_9GAMM   ..QHCDSDTVFATNTSSIPIADIAKASKRPEQVIGLHYFSPVEKMPLLEIIKTEKTSDEV
tr|A0A498C231|A0A498C231_9GAMM   ..AHGPEHVIFASNTSSLPISEIAAGAQRPERVIGMHYFSPVEKMPLLEVIATNRTAPEV
tr|Q0A6T4|Q0A6T4_ALKEH           ..AHGPEHVIFASNTSSLPISEIATGAQRPERVIGMHYFSPVEKMPLLEVIATDRTDPEV
tr|A0A3E0WL10|A0A3E0WL10_9GAMM   ..AHCPEHCIFATNTSSIPIGRIAEGAKRPELVIGMHYFSPVEKMPLLEVIATDKTRPDV
tr|A0A3S1BU16|A0A3S1BU16_9GAMM   ..AHCPAQTIFATNTSSIPIAHIAAAAQRPEQVIGMHYFSPVEKMPLLEIITTAHTAPEV
tr|A0A7V8QFN9|A0A7V8QFN9_9GAMM   ..ANCREDTIFATNTSSIPITDIAGGAKRPENVIGMHYFSPVEKMPLLEVIATDRTAPEV
tr|A0A1H8PPF9|A0A1H8PPF9_9GAMM   ..SNAGETTIFATNTSSIPITRIAQGAKRPQNVIGMHYFSPVEKMPLLEVIATDRTDPEV
tr|A0A6H0J0V7|A0A6H0J0V7_9GAMM   ..THGGERTIFATNTSSIPITRIAEGAERPHNVIGMHYFSPVEKMPLLEVIATEHTDPEV
tr|V5EZX6|V5EZX6_9VIBR           ..QQCPENTIFASNTSSLPIYQIAEGAKRPENIVGLHYFSPAEKMPLVEVIPHATTSAET
tr|A0A6I1QBM2|A0A6I1QBM2_9VIBR   ..QQCPENTIFASNTSSLPIYQIAEGAQRPENIVGLHYFSPAEKMPLVEVIPHATTSAET
tr|A0A511QRL5|A0A511QRL5_9VIBR   ..NECSDSTIFASNTSSLPIHQIAESAQRPENIVGLHYFSPAEKMPLVEVIPHATTSDET
tr|A0A7Y0JZP4|A0A7Y0JZP4_9VIBR   ..NECSDSTIFASNTSSLPIHQIAEGAQRPENIVGLHYFSPAEKMPLVEVIPHATTSDET
tr|A0A193KD24|A0A193KD24_9VIBR   ..SECSDNTIFASNTSSLPIHQIAEGAHRPENIVGLHYFSPAEKMPLVEVIPHATTSDET
tr|A0A1S1HM72|A0A1S1HM72_PROST   ..DVTKGHAIFASNTSSLPINQIAEKAKHPEKVIGLHYFSPVDKMPLVEVIPHQQTDAQT
tr|A0A7T8I613|A0A7T8I613_9GAMM   ..DVTKGHAIFASNTSSLPINQIAEKAKHPEKVIGLHYFSPVDKMPLVEVIPHQQTDAQT
tr|A0A140NIF0|A0A140NIF0_PROSM   ..EVTHGKAIFASNTSSLPIHQIAENAAHPEKVIGLHYFSPVDKMPLVEVIPHQQTDAET
tr|A0A379GMB7|A0A379GMB7_PROST   ..EVTHGKAIFASNTSSLPIHQIAENAAHPEKVIGLHYFSPVDKMPLVEVIPHQQTDAET
tr|A0A379H393|A0A379H393_PROST   ..EVTHGKAIFASNTSSLPIHQIAENAAHPEKVIGLHYFSPVDKMPLVEVIPHQQTDAET
tr|B2Q0L5|B2Q0L5_PROST           ..EVTHGKAIFASNTSSLPIHQIAENAAHPEKVIGLHYFSPVDKMPLVEVIPHQQTDAET
tr|A0A6I3JUE9|A0A6I3JUE9_9GAMM   ..NVIQGKAIFASNTSSLPIHQIAEPAAHPEKVIGLHYFSPVDKMPLVEVIPHQKTDDET
tr|A0A7D4P594|A0A7D4P594_YERMW   ..HFGAAHTIFASNTSSLPISQIAAQAQRPEQVIGLHYFSPVDKMPLVEVIPHAKTSEET
tr|A0A7U7IVK6|A0A7U7IVK6_YEREN   ..HFAAPHTIFASNTSSLPISDIAAQAQRPEQVIGLHYFSPVDKMPLVEVIPHAKTSKET
tr|A0A447RIF5|A0A447RIF5_YEREN   ..HFAAPHTIFASNTSSLPISDIAAQAQRPEQVIGLHYFSPVDKMPLVEVIPHAKTSKET
tr|A0A7H4ZLV9|A0A7H4ZLV9_YERP4   ..HFAAPHTIFASNTSSLPISDIAAQAQRPEQVIGLHYFSPVDKMPLVEVIPHAKTSKET
tr|A0A0H3NX46|A0A0H3NX46_YERE1   ..HFAAPHTIFASNTSSLPISDIAAQAQRPEQVIGLHYFSPVDKMPLVEVIPHAKTSKET
tr|A0A7T9XUV7|A0A7T9XUV7_YEREN   ..HFAAPHTIFASNTSSLPISDIAAQAQRPEQVIGLHYFSPVDKMPLVEVIPHAKTSKET
tr|A0A7U7IYV8|A0A7U7IYV8_YEREN   ..HFAAPHTIFASNTSSLPISDIAAQAQRPEQVIGLHYFSPVDKMPLVEVIPHAKTSKET
tr|A0A0H5G7G2|A0A0H5G7G2_YEREN   ..HFAAPHTIFASNTSSLPISDIAAQAQRPEQVIGLHYFSPVDKMPLVEVIPHAKTSKET
tr|A0A2A7TDI2|A0A2A7TDI2_YERKR   ..HFAAPHTIFASNTSSLPISDIAAQAQRPEQVIGLHYFSPVDKMPLVEVIPHAKTSEET
tr|A0A0T9M261|A0A0T9M261_YERKR   ..HFAAPHTIFASNTSSLPISDIAAQAQRPEQVIGLHYFSPVDKMPLVEVIPNAKTSEET
tr|A0A0A0CMP1|A0A0A0CMP1_PHOLU   ..TNAKPETIFASNTSSLPIHQIAEKAQRPEQVIGLHYFSPVDKMPLVEVIPHQGTSEKT
tr|A0A329VG55|A0A329VG55_9GAMM   ..TNAKPETIFASNTSSLPIHQIAEKAQRPEQVIGLHYFSPVDKMPLVEVIPHQGTSEKT
sp|Q7N288|FADJ_PHOLL             ..TNAKPETIFASNTSSLPIHQIAEKAQRPEQVIGLHYFSPVDKMPLVEVIPHQGTSEKT
tr|A0A6L9JMX5|A0A6L9JMX5_PHOLM   ..TNAKPETIFASNTSSLPIHQIAEKAQRPEQVIGLHYFSPVDKMPLVEVIPHQGTSEKT
tr|A0A7X5HQ29|A0A7X5HQ29_PHOLM   ..TNAKPETIFASNTSSLPIHQIAEKAQRPEQVIGLHYFSPVDKMPLVEVIPHQGTSEKT
tr|A0A1C0U051|A0A1C0U051_9GAMM   ..ASAKPETVFASNTSSLPIHQIAEKAQRPEQVIGLHYFSPVDKMPLVEVIPHKGTSEKT
tr|A0A7X5QF62|A0A7X5QF62_9GAMM   ..ANAKPETVFASNTSSLPIHQIAEKAQRPEQVIGLHYFSPVDKMPLVEVIPHQGTSEKT
```

tr|A0A2D0KWM9|A0A2D0KWM9_9GAMM	..ANTKAETIFASNTSSLPIHQIAEIAKRPEQVIGLHYFSPVDKMPLVEVIPHAGTSEKT
tr|A0A2D0LAI1|A0A2D0LAI1_9GAMM	..ANTKAETIFASNTSSLPIHQIAEIAKRPEQVIGLHYFSPVDKMPLVEVIPHAGTSEKT
tr|A0A2D0IXC8|A0A2D0IXC8_XENBU	..AHTKPETIFASNTSSLPIHQIAAVAKRPEHVIGLHYFSPVDKMPLVEVIPHAGTSEKT
tr|W1JAM5|W1JAM5_9GAMM	..AHTKPETIFASNTSSLPIHQIAEVAKRPEQVIGLHYFSPVDKMPLVEVIPHAGTSEKT
tr|A0A3D9UED3|A0A3D9UED3_9GAMM	..THTKPETIFASNTSSLPIHQIAEVAKRPEQVIGLHYFSPVDKMPLVEVIPHAGTSEKT
tr|A0A1I3JCA9|A0A1I3JCA9_9GAMM	..NNTNPDTIFASNTSSLPIHKIAEIAARPEQVIGLHYFSPVDKMPLVEVIPHAGTSEKT
tr|A0A068QUL9|A0A068QUL9_9GAMM	..EHTKPETIFASNTSSLPIHKIAEVAARPEQVIGLHYFSPVDKMPLVEVIPHAGTSEKT
tr|A0A0M0TCH6|A0A0M0TCH6_9GAMM	..ENTKPETIFASNTSSLPIHKIAETATRPAQVIGLHYFSPVDKMPLVEVIPHEGTSEKT
tr|A0A1Q5U854|A0A1Q5U854_9GAMM	..ENTKPETIFASNTSSLPIHKIAEAATRPEQVIGLHYFSPVDKMPLVEVIPHAGTSEKT
tr|A0A2D0ISP1|A0A2D0ISP1_9GAMM	..ENTKPETIFASNTSSLPIHKIAEAATRPEQVIGLHYFSPVDKMPLVEVIPHAGTSEKT
tr|A0A1Q5TUI8|A0A1Q5TUI8_9GAMM	..EHTKPETIFASNTSSLPIHKIAEAATRPEQVIGLHYFSPVDKMPLVEVIPHKGTSEKT
tr|A0A2D0KJI7|A0A2D0KJI7_9GAMM	..EHTKPETIFASNTSSLPIHKIAEAATRPEQVIGLHYFSPVDKMPLVEVIPHAGTSEKT
tr|A0A1Y2SB97|A0A1Y2SB97_9GAMM	..EHTKPETIFASNTSSLPIHKIAEIATRPEQVIGLHYFSPVDKMPLVEVIPHEGTSEKT
tr|A0A1I5DWI7|A0A1I5DWI7_9GAMM	..VHTKPETIFASNTSSLPIHKIAEVATRPERVIGLHYFSPVDKMPLVEVIPHEGTSEKT
tr|A0A1I7GWY6|A0A1I7GWY6_9GAMM	..DNTKPDTIFASNTSSLPIHKIAEVSTRPEQVIGLHYFSPVDKMPLVEVIPHAGTSEKT
tr|D3VKY8|D3VKY8_XENNA	..DNTKPDTIFASNTSSLPIHKIAEVSTRPEQVIGLHYFSPVDKMPLVEVIPHAGTSEKT
tr|A0A2G0Q6Q9|A0A2G0Q6Q9_9GAMM	..NRAKPETIFASNTSSLPIHKIAEISIRPEQIIGLHYFSPVDKMPLVEVIPHAGTSEKT
tr|A0A2D0JU24|A0A2D0JU24_9GAMM	..ENTKPKTIFASNTSSLPIHKIAEVATRPERIIGLHYFSPVDKMPLVEVIPHAGTSEKT
tr|A0A0J5FN38|A0A0J5FN38_9GAMM	..ENTKPETIFASNTSSLPIHKIAEVATRPEQIIGLHYFSPVDKMPLVEVIPHAGTSEKT
tr|A0A432XLD4|A0A432XLD4_9GAMM	..AAGHENTIFATNTSSLPITKIAANAKRPENVIGLHYFSPVDKMPLAEIITHEGTSDET
tr|A0A2D8HU84|A0A2D8HU84_9GAMM	..EHAAESTIFATNTSSLPITQIAATAKRPEQVIGLHYFSPVDKMPLAEIITHSGTSDKT
tr|A0A656X1Q2|A0A656X1Q2_9GAMM	..EHAAESTIFATNTSSLPITQIAATAKRPEQVIGLHYFSPVDKMPLAEIITHSGTSDKT
tr|A0A4Q1QH79|A0A4Q1QH79_9GAMM	..AHASESTIFATNTSSLPITQIAAKAKRPEQVIGLHYFSPVDKMPLAEIITHEGTSDKT
tr|A0A1G7LQE6|A0A1G7LQE6_9GAMM	..EHASESTIFATNTSSLPITQIAAKAKRPEQVIGLHYFSPVDKMPLAEIITHEGTSDKT
tr|A0A432YVG7|A0A432YVG7_9GAMM	..EHASESTIFATNTSSLPITQIAAKAKRPEQVIGLHYFSPVDKMPLAEIITHEGTSDKT
tr|A0A1J5N0P5|A0A1J5N0P5_9GAMM	..THCGEHTIFASNTSSLPIGKIAQHAERPENVIGLHYFSPVDKMPLVEVIAHEKTSAQT
tr|A0A0D8D548|A0A0D8D548_9GAMM	..EFCKPTTIFASNTSSLPIGQIAAKAARPENVIGLHYFSPVDKMPLAEIIAHEGTSDQT
tr|A0A3E0TNM2|A0A3E0TNM2_9GAMM	..QHCKPETIFASNTSSLPIGQIAEKAQRPENVIGLHYFSPVDKMPLAEIIAHEKTSDQT
tr|A0A3E0UD67|A0A3E0UD67_9GAMM	..QHCKPETIFASNTSSLPIGQIAEKAERPENVIGLHYFSPVDKMPLAEIIAHEKTSDQT
tr|A0A3E0U0P4|A0A3E0U0P4_9GAMM	..QHCKPETIFASNTSSLPIGQIAEKAERPENVIGLHYFSPVDKMPLAEIIAHEKTSDQT
tr|A0A0M2V8B1|A0A0M2V8B1_9GAMM	..QHCAEHTIFASNTSSLPISQIAAKALRPENVIGLHYFSPVDKMPLVEVIAHDKTSAQT
tr|A0A285ITY5|A0A285ITY5_9GAMM	..QHCGEHTIFASNTSSLPISQIAAKATRPENVIGLHYFSPVDKMPLVEVIAHDKTSAQT
tr|A0A486XTB9|A0A486XTB9_9GAMM	..QHCSERTIFASNTSSLPIKQIAAKAKRPENVIGLHYFSPVDKMPLVEVIAHDGTSAQT
tr|I1E1G1|I1E1G1_9GAMM	..QHCSENTIFASNTSSLPIKQIAAKAQRPENVIGLHYFSPVDKMPLVEVIAHEGTSAQT
tr|A0A1H6KJ68|A0A1H6KJ68_9GAMM	..QHCSEHTIFASNTSSLPIGQIAAKAARPENVIGLHYFSPVDKMPLVEVIAHETTSAQT
tr|A0A0X3Y764|A0A0X3Y764_9GAMM	..QHCADSTIFASNTSSLPIGQIAAKAARPENVIGLHYFSPVDKMPLVEVIAHEATSAQT
tr|A0A2N1YEV4|A0A2N1YEV4_9GAMM	..QHCADSTIFASNTSSLPIGQIAAKAARPENVIGLHYFSPVDKMPLVEVIAHETTSAQT
tr|A0A3P3QCM3|A0A3P3QCM3_9GAMM	..ANCSEQTIFASNTSSLPIGQIAAKAARPENVIGLHYFSPVDKMPLVEVIAHEKTSAET
tr|F7NT72|F7NT72_9GAMM	..TNCSEHTVFASNTSSLPIGQIAAKAARPENVIGLHYFSPVDKMPLVEVIAHDKTSAET
tr|A0A3S2TW63|A0A3S2TW63_9GAMM	..TNCSEHTVFASNTSSLPIGQIAAKAARPENVIGLHYFSPVDKMPLVEVIAHDKTSAET
tr|A0A3D5BGW7|A0A3D5BGW7_9GAMM	..SHCHENTIFASNTSSLPIGQIAAQAARPENVIGLHYFSPVDKMPLVEVIAHEKTSAQT
tr|A0A5C7TI17|A0A5C7TI17_9GAMM	..QHCSEHTVFASNTSSLPIGQIAAKAARPENVIGLHYFSPVDKMPLVEVIAHEKTSAET
tr|A0A0U4W982|A0A0U4W982_9GAMM	..QHCSEHTVFASNTSSLPIGQIAAKAARPENVIGLHYFSPVDKMPLVEVIAHDKTSAET
tr|A0A2I0FCM2|A0A2I0FCM2_9GAMM	..ANCNENTIFASNTSSLPITQIASEAKRPENVIGLHYFSPVDKMPLAEIITHEGTSDQT
tr|A0A2G2IVL1|A0A2G2IVL1_9GAMM	..ANCNENTIFASNTSSLPITQIASEAKRPENVIGLHYFSPVDKMPLAEIITHEGTSDQT
tr|A6FI21|A6FI21_9GAMM	..ANCNENTIFASNTSSLPITQIASEAKRPENVIGLHYFSPVDKMPLAEIITHEGTSDQT
tr|A0A4U1BNR2|A0A4U1BNR2_9GAMM	..ANCGPDTIFATNTSSLPIGQIAAVAERPEQVIGLHYFSPVEKMPLVEVISHEKTSAQT
tr|E1SQ60|E1SQ60_FERBD	..ANCGEHTIFASNTSSLPIGQIAEAASRPDKVIGLHYFSPVEKMPLVEVIAHKGTSAET
tr|A0A4Y6IZ09|A0A4Y6IZ09_9GAMM	..RECGEHTIFASNTSSLPIKQIAEAAERPENVIGLHYFSPVEKMPLVEVIAHEKTSPQT
tr|A0A0C3QSZ7|A0A0C3QSZ7_9GAMM	..RECGEHTIFASNTSSLPIKQIAEAAERPENVIGLHYFSPVEKMPLVEVIAHEKTSPET
tr|A0A6L7HXW0|A0A6L7HXW0_9GAMM	..RECGEHTIFASNTSSLPIKQIAEAAERPENVIGLHYFSPVEKMPLVEVIAHDKTSPQT
sp|A3QFP3|FADJ_SHELP	..RECGEHTIFASNTSSLPIKQIAEAAERPENVIGLHYFSPVEKMPLVEVIAHDKTSPQT
tr|A0A1E5IXH7|A0A1E5IXH7_SHECO	..RECGEHTIFASNTSSLPISQIAEAAQRPENVIGLHYFSPVEKMPLVEVIAHEKTSAET
tr|A0A411PKQ0|A0A411PKQ0_9GAMM	..RECGENTIFASNTSSLPIGQIAQAASRPENVIGLHYFSPVEKMPLVEVIAHKTTSPET
tr|A0A6G9QKM3|A0A6G9QKM3_9GAMM	..RECGENTIFASNTSSLPIGQIAQAATRPENVIGLHYFSPVEKMPLVEVIAHKTTSPET
tr|A0A6P1UL63|A0A6P1UL63_9GAMM	..RECGENTIFASNTSSLPIGQIAAAASRPENVIGLHYFSPVEKMPLVEVIAHKTTSPET
tr|A0A2N1ERI9|A0A2N1ERI9_9GAMM	..RECGENTIFASNTSSLPIGQIAAAASRPENVIGLHYFSPVEKMPLVEVIAHKTTSPET
tr|A0A7W4FU55|A0A7W4FU55_9GAMM	..RECGENTIFASNTSSLPIGQIAAAASRPENVIGLHYFSPVEKMPLVEVIAHKTTSPET
sp|Q8ECP7|FADJ_SHEON	..RECGEHTIFASNTSSLPIGQIAQAASRPENVIGLHYFSPVEKMPLVEVIAHAKTSPET
tr|A0A501XZY8|A0A501XZY8_9GAMM	..RECGEHTIFASNTSSLPIGQIAQAASRPENVIGLHYFSPVEKMPLVEVIAHAKTSPET
tr|A0A2W5DCZ0|A0A2W5DCZ0_SHEOE	..RECGEHTIFASNTSSLPIGQIAQAASRPENVIGLHYFSPVEKMPLVEVIAHAKTSPET
tr|A0A1E3V3C8|A0A1E3V3C8_9GAMM	..RECGEHTIFASNTSSLPIGQIAQAASRPENVIGLHYFSPVEKMPLVEVIAHAKTSPET
tr|A0A1Z4AI20|A0A1Z4AI20_9GAMM	..RECGEHTIFASNTSSLPIGQIAQAASRPENVIGLHYFSPVEKMPLVEVIAHAKTSPET
tr|A0A7X9LJL9|A0A7X9LJL9_9GAMM	..RECGEHTIFASNTSSLPIGQIAQAASRPENVIGLHYFSPVEKMPLVEVIAHAKTSPET
tr|A0A073KMY5|A0A073KMY5_9GAMM	..RECGEHTIFASNTSSLPIGQIAQAASRPENVIGLHYFSPVEKMPLVEVIAHAKTSPET
sp|A0KV76|FADJ_SHESA	..RECGEHTIFASNTSSLPIGQIAQAASRPENVIGLHYFSPVEKMPLVEVIAHAKTSPET
tr|A0A220UTH7|A0A220UTH7_9GAMM	..RECGEHTIFASNTSSLPIGQIAQAASRPENVIGLHYFSPVEKMPLVEVIAHAKTSPET
tr|A0A5B8R6W1|A0A5B8R6W1_9GAMM	..RECGEHTIFASNTSSLPIGQIAQAASRPENVIGLHYFSPVEKMPLVEVIAHAKTSPET
tr|V1DAI4|V1DAI4_9GAMM	..RECGEHTIFASNTSSLPIGQIAQAASRPENVIGLHYFSPVEKMPLVEVIAHAKTSPET
tr|A0A448CPQ4|A0A448CPQ4_SHEPU	..RECGEHTIFASNTSSLPIGQIAQAASRPENVIGLHYFSPVEKMPLVEVIAHAKTSPET
tr|A0A252ERQ3|A0A252ERQ3_SHEPU	..RECGEHTIFASNTSSLPIGQIAQAASRPENVIGLHYFSPVEKMPLVEVIAHAKTSPET
sp|Q0HKD1|FADJ_SHESM	..RECGEHTIFASNTSSLPIGQIAQAASRPENVIGLHYFSPVEKMPLVEVIAHAKTSPET
sp|Q0HWN3|FADJ_SHESR	..RECGEHTIFASNTSSLPIGQIAQAASRPENVIGLHYFSPVEKMPLVEVIAHAKTSPET
tr|F7RQE3|F7RQE3_9GAMM	..RECGEHTIFASNTSSLPISQIAEAATRPENVIGLHYFSPVEKMPLVEVIAHAKTSPET
tr|B8EE98|B8EE98_SHEB2	..RECGEHTIFASNTSSLPISQIAEAATRPENVIGLHYFSPVEKMPLVEVIAHAKTSPET
sp|A6WQ25|FADJ_SHEB8	..RECGEHTIFASNTSSLPISQIAEAATRPENVIGLHYFSPVEKMPLVEVIAHAKTSPET
tr|A0A448EK41|A0A448EK41_9GAMM	..RECGEHTIFASNTSSLPISQIAEAATRPENVIGLHYFSPVEKMPLVEVIAHAKTSPET
tr|A0A553JHX1|A0A553JHX1_SHEHA	..RECGEHTIFASNTSSLPISQIAEAASRPENVIGLHYFSPVEKMPLVEVIAHKKTSAET
tr|B8CPY6|B8CPY6_SHEPW	..RECGENTIFASNTSSLPISQIAEAATRPENVIGLHYFSPVEKMPLVEVIAHEKTSAET
tr|A0A431WFC4|A0A431WFC4_9GAMM	..RECGENTIFASNTSSLPISQIAEAASRPENVIGLHYFSPVEKMPLVEVIAHEKTSAET
tr|A8FTR7|A8FTR7_SHESH	..RECGENTIFASNTSSLPISQIAEAASRPENVIGLHYFSPVEKMPLVEVIAHEKTSAET
tr|A0A431WNL0|A0A431WNL0_9GAMM	..RECGENTIFASNTSSLPISQIAEAASRPENVIGLHYFSPVEKMPLVEVIAHEKTSAET
tr|A0A550AEC5|A0A550AEC5_9GAMM	..RECGEHTIFASNTSSLPISQIAEAASRPENVIGLHYFSPVEKMPLVEVIAHEKTSAET
tr|A0A7L4WW90|A0A7L4WW90_9GAMM	..RECGEHTIFASNTSSLPISQIAEAASRPENVIGLHYFSPVEKMPLVEVIAHEKTSAET
tr|A9DDU3|A9DDU3_9GAMM	..RECGENTIFASNTSSLPISQIAEAATRPENVIGLHYFSPVEKMPLVEVIAHKKTSPET
tr|A0A330M2B9|A0A330M2B9_9GAMM	..RECGENTIFASNTSSLPISQIAEAATRPENIIGLHYFSPVEKMPLVEVIAHKKTSPET
tr|A0A5N8UFC7|A0A5N8UFC7_9GAMM	..RECGENTIFASNTSSLPISQIAEAAARPENVIGLHYFSPVEKMPLVEVIAHKKTSPET
tr|A0A1S6HN57|A0A1S6HN57_9GAMM	..RECGENTIFASNTSSLPISQIAEAASRPENVIGLHYFSPVEKMPLVEVIAHKKTSPET
tr|D4ZMH7|D4ZMH7_SHEVD	..RECGENTIFASNTSSLPISQIAEAAARPENVIGLHYFSPVEKMPLVEVIAHKKTSPET
tr|A0A3L8Q213|A0A3L8Q213_9GAMM	..RECNENTIFASNTSSLPIGQIAEAAERPENVIGLHYFSPVEKMPLVEVIAHEKTSQQT
tr|A0A3A6U4N9|A0A3A6U4N9_9GAMM	..RECSKETIFASNTSSLPIGQIAEVAERPENVIGLHYFSPVDKMPLVEVIAHEKTSAET
tr|A0A4Q5MA37|A0A4Q5MA37_9GAMM	..RECSENTIFASNTSSLPIGQIAEAAERPENVIGLHYFSPVEKMPLVEVIAHKKTSQQT
tr|A0A1L6LSX5|A0A1L6LSX5_9DELT	..AAGKEGVIFASNTSSIPIAKIAAGSKHPENVIGMHYFSPVHKMPLLEVIQTKETAPEV
tr|A0A2W4L9B9|A0A2W4L9B9_9PROT	..EVIREDCILASNTSTIPITRLAEASRRPENVVGMHYFSPVEKMPLLEVIRGKKTGDSA
tr|A0A2W4M4E6|A0A2W4M4E6_9PROT	..QYGHPNAIFASNTSSLPITKIAERARAPERVIGMHYFSPVEKMPLLEIVVTDRTADWV
tr|A0A6I2GRX9|A0A6I2GRX9_9DELT	..AVTREDCIFASNTSSLPITELAKGAKRPQNVIGMHYFSPVHKMPLLEIITHAGTADWV
tr|A0A0H4WMK2|A0A0H4WMK2_9DELT	..AVTGEQTIFASNTSSLPITDLAKGSRRPGQVIGMHYFSPVHKMPLLEIITHAGTEDWV
tr|F8CJ36|F8CJ36_MYXFH	..AVTGDQTIFASNTSSIPITELAKGSRRPAQVIGMHYFSPVHKMPLLEVITHAGTADWV
tr|A0A250K0F1|A0A250K0F1_9DELT	..AVTGDQTIFASNTSSIPITELAKGSRRPAQVIGMHYFSPVHKMPLLEVITHAGTADWV
tr|A0A7Y6WFZ2|A0A7Y6WFZ2_9DELT	..AVTGDQTIFASNTSSIPITELAKGSRRPAQVIGMHYFSPVHKMPLLEVITHAGTADWV
tr|A0A7Y7C660|A0A7Y7C660_9DELT	..AVTGDQTIFASNTSSIPITELAKGSRRPAQVIGMHYFSPVHKMPLLEVITHAGTADWV
tr|A0A7Y4JFH2|A0A7Y4JFH2_MYXXA	..AVTGDQTIFASNTSSIPITELAKGSRRPAQVIGMHYFSPVHKMPLLEIITHAGTADWV
tr|A0A4Y6CZQ9|A0A4Y6CZQ9_MYXXA	..AVTGDQTIFASNTSSIPITELAKGSRRPAQVIGMHYFSPVHKMPLLEVITHAGTADWV

```
tr|A0A7Y4IKV5|A0A7Y4IKV5_MYXXA   ..AVTGDQTIFASNTSSIPITELAKGSRRPAQVIGMHYFSPVHKMPLLEIITHAGTADWV
tr|A0A511HHB0|A0A511HHB0_9DELT   ..AVTGDQTIFASNTSSIPITELAKGSRRPAQVIGMHYFSPVHKMPLLEIITHAGTADWV
tr|A0A4Y6CKY7|A0A4Y6CKY7_MYXXA   ..AVTGDQTIFASNTSSIPITELAKGSRRPAQVIGMHYFSPVHKMPLLEIITHAGTADWV
tr|Q1D1F2|Q1D1F2_MYXXD           ..AVTGDQTIFASNTSSIPITELAKGSRRPAQVIGMHYFSPVHKMPLLEIITHAGTADWV
tr|A0A7Y4MA14|A0A7Y4MA14_MYXXA   ..AVTGDQTIFASNTSSIPITELAKGSRRPAQVIGMHYFSPVHKMPLLEIITHAGTADWV
tr|A0A7T8Y4N9|A0A7T8Y4N9_MYXXA   ..AVTGDQTIFASNTSSIPITELAKGSRRPAQVIGMHYFSPVHKMPLLEIITHAGTADWV
tr|L7UE67|L7UE67_MYXSD           ..AVTRPDAIFASNTSSIPITELAKGSQRPAQVIGMHYFSPVNKMPLLEVITHAGTADWV
tr|A0A511T9X1|A0A511T9X1_MYXFU   ..AVTGPDAIFASNTSSIPITELAKGSKRPAQVIGMHYFSPVNKMPLLEIITHAGTADQV
tr|A0A7Y7C9C4|A0A7Y7C9C4_9DELT   ..AVTGPDAIFASNTSSIPITELAKGSQRPAQVIGMHYFSPVNKMPLLEIITHAGTADWV
tr|A0A540X7W8|A0A540X7W8_9DELT   ..AVTGPDAIFASNTSSIPITELAKGSQRPAQVIGMHYFSPVNKMPLLEIITHAGTADWV
tr|A0A3A5FK19|A0A3A5FK19_9DELT   ..AVTREDCIFASNTSSLPISELAKGSKRPAQVIGMHYFSPVHKMPLLEIITHPGTAEWV
tr|A0A3A8JQL9|A0A3A8JQL9_9DELT   ..AVTGENTIFASNTSSLPIGELAKGSKRPSQVIGMHYFSPVHKMPLLEIITHPGTAEWV
tr|A0A7Y4NFA4|A0A7Y4NFA4_9DELT   ..AVTGENTIFASNTSSLPIGELAKGSKRPSQVIGMHYFSPVHKMPLLEIITHPGTAEWV
tr|A0A3A8HDQ7|A0A3A8HDQ7_9DELT   ..AVTGENTIFASNTSSLPIGELAKGSKRPSQVIGMHYFSPVHKMPLLEIITHPGTAEWV
tr|A0A3A8GR90|A0A3A8GR90_9DELT   ..AVTGENTIFASNTSSLPIGELAKGSKRPSQVIGMHYFSPVHKMPLLEIITHPGTAEWV
tr|A0A3A8SBD7|A0A3A8SBD7_9DELT   ..AVTGENTIFASNTSSLPIGELAKGSKRPSQVIGMHYFSPVHKMPLLEIITHPGTAEWV
tr|A0A3A8T0I8|A0A3A8T0I8_9DELT   ..AVTGENTIFASNTSSLPIGELAKGSKRPSQVIGMHYFSPVHKMPLLEIITHPGTAEWV
tr|A0A7X5BU07|A0A7X5BU07_9DELT   ..AVTGENTIFASNTSSLPIGELAKGSNRPSQVIGMHYFSPVHKMPLLEIITHPGTAEWV
tr|A0A7Y1RVL2|A0A7Y1RVL2_9DELT   ..AVTGENTIFASNTSSLPIGELAKGSKRPSQVIGMHYFSPVHKMPLLEIITHPGTAEWV
tr|A0A3A8THN2|A0A3A8THN2_9DELT   ..AVTGENTIFASNTSSLPIGELAKGSKRPSQVIGMHYFSPVHKMPLLEIITHPGTAEWV
tr|A0A7Y1RX76|A0A7Y1RX76_9DELT   ..AVTGENTIFASNTSSLPIGELAKGSKRPSQVIGMHYFSPVHKMPLLEIITHPGTAEWV
tr|A0A3A8RA58|A0A3A8RA58_9DELT   ..AVTGENTIFASNTSSLPIGELAKGSKRPSQVIGMHYFSPVHKMPLLEIITHPGTAEWV
tr|A0A410RPB6|A0A410RPB6_CORCK   ..AVTGENTIFASNTSSLPIGELAKGSKRPSQVIGMHYFSPVHKMPLLEIITHPGTAEWV
tr|A0A7Y4J474|A0A7Y4J474_CORCK   ..AVTGENTIFASNTSSLPIGELAKGSKRPSQVIGMHYFSPVHKMPLLEIITHPGTAEWV
tr|A0A3A8I9Z6|A0A3A8I9Z6_9DELT   ..AVTGENTIFASNTSSLPIGELAKGSKRPSQVIGMHYFSPVHKMPLLEIITHPGTAEWV
tr|H8MKE9|H8MKE9_CORCM           ..AVTGENTIFASNTSSLPIGELAKGSKRPSQVIGMHYFSPVHKMPLLEIITHPGTAEWV
tr|A0A3A8H102|A0A3A8H102_9DELT   ..AVTGENTIFASNTSSLPIGELAKGSKRPSQVIGMHYFSPVHKMPLLEIITHPGTAEWV
tr|A0A554FW33|A0A554FW33_9DELT   ..AVTGADTIFASNTSSLPIGELAKGSKRPSQVIGMHYFSPVHKMPLLEIITHPGTAEWV
tr|A0A3A8NPC0|A0A3A8NPC0_9DELT   ..AVTGPDTIFASNTSSLPIGDLAKGSKRPAQVIGMHYFSPVHKMPLLEIITHPGTAEWV
tr|A0A3A8JUV3|A0A3A8JUV3_9DELT   ..AITGPDTIFASNTSSLPIGELAKGSKRPAQVIGMHYFSPVHKMPLLEIITHPGTAEWV
tr|A0A3A8NEX6|A0A3A8NEX6_9DELT   ..AVTGADTIFASNTSSLPIGELAKGSKRPSQVIGMHYFSPVHKMPLLEIITHPGTAEWV
tr|A0A3A8JEU8|A0A3A8JEU8_9DELT   ..AVTGPDTIFASNTSSLPITELAKGSKRPSQVIGMHYFSPVHKMPLLEIITHPGTAEWV
tr|A0A3A8LI76|A0A3A8LI76_9DELT   ..AVTGPDTIFASNTSSLPITELAKGSKRPSQVIGMHYFSPVHKMPLLEIITHPGTAEWV
tr|A0A085WXN8|A0A085WXN8_9DELT   ..AVTREDCIFASNTSSLPITELAKGSKRPEQVIGMHYFSPVHKMPLLEIITHKGTADWV
tr|A0A2T4V0M5|A0A2T4V0M5_9DELT   ..AVTREDCIFASNTSSIPITELAKGSKRPAQVIGMHYFSPVNKMPLLEIITHKGTADWV
tr|A0A0G2ZSW5|A0A0G2ZSW5_9DELT   ..LVTREDCIFASNTSSIPITELAKGSRRPAQVIGMHYFSPVNKMPLLEIITHKGTADWV
tr|A0A3M2DKY0|A0A3M2DKY0_9DELT   ..DIGGDDVIFASNTSSIPIARIAEASRHPETVIGMHYFSPVHKMPLLEVIVTDATADWV
tr|A0A661NQ58|A0A661NQ58_9DELT   ..AATGKETIFASNTSSLPITRIAEGSAHPETVIGMHYFSPVHKMPLLEIITTEATADWV
tr|A0A520YD99|A0A520YD99_9DELT   ..EAGRDDVIFASNTSSLPITMIAAASKHPETVIGMHYFSPVHKMPLLEIIVTDKTADWV
tr|A0A7Y3BRE4|A0A7Y3BRE4_9DELT   ..EAGRDDVIFASNTSSLPITMIAAASKHPETVIGMHYFSPVHKMPLLEIIVTDKTADWV
tr|A0A2D9TF90|A0A2D9TF90_9DELT   ..AHGPEGVIFASNTSSLPITQIADAAAKPENVIGMHYFSPVHKMPLLEIIVTEKTSDEV
tr|A0A2E0TP32|A0A2E0TP32_9DELT   ..AAGPEDVIFASNTSSLPIGEIAEASMHPETVIGMHYFSPVHKMPLLEIIVTEKTADWV
tr|A0A2E4Y3V1|A0A2E4Y3V1_9PROT   ..KIAPEDCVFASNTSALPIKQIAAVSKRPENVLGMHYFSPVEKMPLLEIIVTDKTSDRA
tr|A0A2E6VRH4|A0A2E6VRH4_9DELT   ..EHINEDCIFATNTSALPIADIAKGSKRPENVVGMHYFSPVEKMPLLEVIKTDETSENA
tr|A0A1F9FB59|A0A1F9FB59_9DELT   ..DHVRTDCVVASNTSALPITKLAEASKRPHNIIGMHYFSPVPKMPLLEIIVHKGTAKEA
```

```
ECHA_HUMAN                                    530       540       550       560       570       580
ECHA_HUMAN                        SASAVAVGLKQGKVIIVVKDGPGFYTTRCLAPMMSEVIRILQEGVDPKKLDSLTTSFGFP
tr|A0A4R6XLA8|A0A4R6XLA8_9GAMM    RQMATNVGIKQGKTVVVVKDGPGFYTTRVLSALTHEAMKVLQDGAKIEDVDNAMKDWGFP
tr|A0A6A0IJT2|A0A6A0IJT2_9BACT    VATARAYGVAQGKTVIVVKDGPGFYTTRILAPMMNEAILLLDERADVEAIEAALCDWGFP
tr|A0A7V3QZU7|A0A7V3QZU7_9BACT    VATARAFAVKQGKTVIVVEDGPGFYTTRILSPFLNEAVLLLEEGARMEALDSALKDFGYP
tr|A0A7V4SYJ6|A0A7V4SYJ6_9BACT    LATARAFGVSQGKTCIVVKDGPGFYTTRILSPFLNEAMVLLEEGARVEAVDRALKDFGYP
tr|A0A2A8D2N6|A0A2A8D2N6_9BACT    IATAFDAGLLQGKTVIVVNDGPGFYTTRILAIYMNEALLLLEEGGDVEEIDKIMKDFGFP
tr|A0A2N1TGP0|A0A2N1TGP0_9SPIR    TATVLEFGIRQGKTCIVVKDGPGFYTTRILAPLLFESGLLVYEGAEIPAIDGAMKQFGYP
tr|A0A1G3QGW4|A0A1G3QGW4_9SPIR    TATALELGIRQGKTCIIVRDGPGFYTTRILAPLLLESSRVISEGGEIPEVDRAMQLFGYP
tr|A0A1G3QTY3|A0A1G3QTY3_9SPIR    TATALDLGIRQGKTCIIVKDGPGFYTTRILAPLLLEGSRVIAEGGGLPEVDRAMQLFGYP
tr|A0A7X9L359|A0A7X9L359_9DELT    KATALEFGIQQGKTCIVVKDCPGFYTTRILIPMLNESMKLIEEGADIKAIDTAMKRFGYP
tr|A0A522CJY0|A0A522CJY0_9SPIR    VSTARDYGTKQGKTCIVVKDGPGFYTTRILAPLLNEAVVLVEEGADIHDIDRAMHLFGYP
tr|A0A2N1RRJ3|A0A2N1RRJ3_9SPIR    TASALDLGIAQGKTCIVVKDGPGFYTTRILAPMLNEVMLLIEEGAVPDEIDRAMRQFGYP
tr|A0A2N2KFN7|A0A2N2KFN7_9DELT    AATALEMGISQGKTCIVVKDGPGFYTTRILSPMLNEVVLLIEEGAIPNDIDLAMRRFGYP
tr|A0A1V6AXX9|A0A1V6AXX9_9DELT    TATALDMGIAQGKTCIVVKDGPGFYTTRILAPLLNEAVLLVEEGADSVDIDRAMRQFGYP
tr|A0A2N2HZL0|A0A2N2HZL0_9DELT    TSTALDMGIAQGKTCIVVKDGPGFYTTRILVPLLNEAVLLVEEGADSLDIDRAMRQFGYP
tr|A0A5E8ARP3|A0A5E8ARP3_9BACT    TATALEVGVKQGKTCIVVKDGPGFYTTRILAPFINEALLLMQEGADPLQMDKAMKKFGFP
tr|A0A662A7B1|A0A662A7B1_9BACT    TATALDVGIKQGKTCIVVKDGPGFYTTRILAPFMNEALLMQEGVDPLFLDKTMKSFGFP
tr|D7CV16|D7CV16_TRURR            LGTALQVGLEQGKTPIVVGDAPGFYTTRILALYMNEALVLLDEGARVQDIDRAIKDFGFP
tr|A0A3C2AKX4|A0A3C2AKX4_9FLAO    TSTCYDLGVRQGKTVIVVKDGPGFYTTRILAPLMGEALNMLDEGADALQIDKALKKFGFP
tr|A0A3B8ZLN3|A0A3B8ZLN3_9PLAN    TATCYDVGVRQGKTVIVVKDGPGFYTTRILAPFMGETLNLLDEGADALQIDRVIKKFGFP
tr|A0A345UGV8|A0A345UGV8_9BACT    ISTAYEIGIRQGKTVIVVHDGPGFYTTRILAPFINEALLLLEEGAAIEDIDRAMKDFGFP
tr|A0A6C1P6A2|A0A6C1P6A2_9BACT    LHTAIEVGMKQGKTVIVVNDGPGFYTTRILAPFMNEAMLLLEEGAAIDEIDKAMKKLGFP
tr|A0A3M1X9W8|A0A3M1X9W8_9BACT    TATAYQVGVQQGKTCIVVNDGPGFYTTRILAPLLNEALLLLEEGGDIPYIDRVMRQFGFP
tr|A0A354C796|A0A354C796_9DELT    RATALDVGIRQGKTVIIVRDGPGFYTTRILAPLLNEALEILDEGGDIEDIDRAMRRFGFP
tr|A0A3M1NUM8|A0A3M1NUM8_9BACT    TATAIDVGIRQGKTVVVVKDGPGFYTTRILSPMMNEALLLLEEGGDVLHIDRVMKKFGFP
tr|A0A3M2KRC8|A0A3M2KRC8_9BACT    TATAVDVGIRQGKTVIVVKDGPGFYTTRILAPMMNEALLLLEEGGEIRHIDQTMKRFGFP
tr|A0A5S9IHR0|A0A5S9IHR0_9BACT    TATAYDLGVKQGKTVIVVGDGPGFYTTRILAPLMNEAICLLEEGAKIEDIDRNMKLFGYP
tr|A0A6M1ST81|A0A6M1ST81_9BACT    TATAREVGIQQGKHVIVVNDGPGFYTTRILAPFMNEALMLLDEGVSIEQLDKDMKSFGFP
tr|A0A521AAE5|A0A521AAE5_9BACT    TATAREVGIRQGKHVIVVNDGPGFYTTRILAPYMNEALMLLEEGIAIKELDNHMKQFGFP
tr|A0A1M4ZMD6|A0A1M4ZMD6_9BACT    AATAREVGIRQGKHVIVVNDGPGFYTTRILAPYMNEALMLLEEGISIEELDKHMKQFGFP
tr|A0A2A2GCS2|A0A2A2GCS2_9BACT    TATAREVGIQQGKHVIVVNDGPGFYTTRILAPFMNEALMLLEEGISIKELDKHMKRFGFP
tr|A0A5D3YIH7|A0A5D3YIH7_9BACT    TATAREVGIQQGKHVIVVNDGPGFYTTRILSPFMNEALMLLEEGISIKQLDHDMKQFGFP
tr|A0A6M1T9G0|A0A6M1T9G0_9BACT    TATAREVGIQQGKHVIVVNDGPGFYTTRILSPFMNEALMLLEEGIAIKELDSHMKNFGFP
tr|A0A6A8Q1Y8|A0A6A8Q1Y8_9BACT    TATAFRVGVNQGKNVIVVKDGPGFYTTRILAPYMNEGLLLLEEGAEIEYMDTIMKDFGFP
tr|A0A521BVJ3|A0A521BVJ3_9BACT    IQTACQVGVNQGKNVIVVGDGPGFYTTRILAPYMNEALKLLEEGASIEFLDKIMKDFGFP
tr|A0A359E0Y8|A0A359E0Y8_9BACT    TQTAFQVGVKQGKNVIVVGDGPGFYTTRILAPYMNEALTLLEEGASIEFLDKIMKDFGFP
tr|A0A2D8CIM6|A0A2D8CIM6_9BACT    TQTAFQVGVKQGKNVIVVGDGPGFYTTRILAPYMNEALTLLEEGASIEFLDKIMKDFGYP
tr|A0A2D9FWF5|A0A2D9FWF5_9BACT    TQTAYQVGVNQGKNVIVVKDGPGFYTTRILAPYMNEALKLLEEGASIEFLDKIMKDFGFP
tr|A0A1B6YB70|A0A1B6YB70_9BACT    TATAYEVGVKQGKTVIVVNDGPGFYTTRILAPFMNEALLLLEEGASIEFLDKAMKQFGYP
tr|A0A2D4ZTC0|A0A2D4ZTC0_9BACT    TATAYEVGVKQGKTVIVVNDGPGFYTTRILAPFMNEALLLLEEGASIEFLDKAMKQFGFP
tr|A0A3D4UVW7|A0A3D4UVW7_9BACT    TATAYEVGVKQGKTVIVVNDGPGFYTTRILAPFMNEALLLLEEGASIEFLDKAMKQFGYP
tr|A0A3F3I3M2|A0A3F3I3M2_9BACT    TATAYEVGVKQGKTVIVVNDGPGFYTTRILAPFMNEALLLLEEGASIEFLDKAMKQFGYP
tr|A0A3D1G7L9|A0A3D1G7L9_9BACT    TATAYEVGVKQGKTVIVVNDGPGFYTTRILAPYMNEALLLLEEGASIEFLDKIMKQFGYP
tr|A0A3M8G1Y8|A0A3M8G1Y8_9BACT    TATAYEVGVKQGKTVIVVNDGPGFYTTRILAPYMNEALLLLEEGASIEFLDKVMKKFGYP
tr|A0A5Q4F391|A0A5Q4F391_9BACT    LATAYDVGVKQGKTVIVVNDGPGFYTTRILAPFMNEALLLLEEGASVEQLESTMKQFGFP
tr|A0A6I7NPV0|A0A6I7NPV0_9BACT    TATAYDIGLMQGKTVIVVNDGPGFYTTRILAPYMNEALLLLEEGASAEQLDDAMKQFGFP
tr|A0A651G1J2|A0A651G1J2_9BACT    VATAYDIGVKQGKTVIVVNDGPGFYTTRILAPYMNEALLLLEEGASIEQLDRAMKQFGFP
tr|A0A371QRK5|A0A371QRK5_9BACT    IATAYEVGLRQGKTVIVVEDGPGFYTTRILAPFINEALLMLEEGASIEQLDKAMKQFGFP
tr|A0A2N0VGJ5|A0A2N0VGJ5_9BACT    TATAYEVGLNQGKTVIVVNDGPGFYTTRILAPFMNEALLLLEEGAKIEDLDKAMKQFGFP
tr|A0A316TYJ0|A0A316TYJ0_9BACT    TATAFEVGLKQGKTVIVVNDGPGFYTTRILAPFMNEALLLLEEGASIEQLDSAMKDFGFP
tr|A0A651GLZ8|A0A651GLZ8_9BACT    IATAYDVGLKQDKTVIVVNDGPGFYTTRILAPFMNEALLLLEEGASIEQLDRAMKQFGFP
tr|A0A7Y5V3S5|A0A7Y5V3S5_9BACT    VATAVAFGRALGKTVIVVNDAPGFYVNRILAPYLNEAGALLDAGAPVEAIDAAMEQFGFP
tr|A0A7W1SHC0|A0A7W1SHC0_9BACT    IVDAVAYGRRLGKTVIVVNDGPGFYTTRALAAYLNEAGKLLDEGASIGSIDRALTDFGFP
tr|A0A7Y5PBT7|A0A7Y5PBT7_9BACT    TVTAVAYGKRLGKTVIVVNDAPGFYTTRTLSAYMNEAGRLLDEGAAIESVDAALTTFGFP
tr|A0A7Y5TXL8|A0A7Y5TXL8_9BACT    TVTAVAYGKRLGKTVIVVNDAPGFYTTRTLSAYMNEAGRLLDEGAAIESVDAALTTFGFP
tr|A0A423PQ96|A0A423PQ96_9GAMM    IATAVAFGKAQGKTVIVVGDGPGFYTTRVLSPYLNEAARLLSEGAGVRQIDQALTDHGFP
tr|U2G066|U2G066_9GAMM            IATAVAFGKAQGKTVIVVRDGPGFYTTRALSPYLNEAARLLAEGASVRAIDEALVDYGFP
tr|A0A2E0J1F4|A0A2E0J1F4_9GAMM    IATAVAFGKAQGKTVIVVADGPGFYTTRALSPYLNEAARLLSEGAGVRQIDEALVNHGFP
tr|A0A2D4SCM8|A0A2D4SCM8_9GAMM    IAASVKFGKAQGKTIIVVKDGPGFYTTRILAPFLNEATRILSEGVEIRAVDKALKDYGFP
tr|L0WJH1|L0WJH1_9GAMM            TAGCVAFGKAQGKTVIVVKDGPGFYTTRILAPYLNEATRLLTEGVDVKQVDQALVRFGFP
tr|A0A1H5XF61|A0A1H5XF61_9GAMM    TAGCVAFGKAQGKTVIVVKDGPGFYTTRILAPYLNESTRLLTEGVDVKAVDKALVRFGFP
tr|A0A2E9TS48|A0A2E9TS48_9GAMM    TAGCVAFGKAQGKTVIVVKDGPGFYTTRILAPYLNEATRLLTEGVDVKAVDKALVRFGFP
tr|A0A095UE19|A0A095UE19_9GAMM    TAGCVAFGKAQGKTVIVVKDGPGFYTTRILAPYLNEATRLLTEGVDVKAVDKALVRFGFP
tr|A0A7G2S9A0|A0A7G2S9A0_9GAMM    TAGCVAFGKAQGKTVIVVKDGPGFYTTRILAPYLNEATRLLTEGVDVKAVDKALVRFGFP
tr|B5JU27|B5JU27_9GAMM            TKGCVAFGKAQGKTVIVVKDGPGFYTTRILAPYLNEASRLLLEGVGPKELDKALQQFGFP
tr|A0A1Y0IHP1|A0A1Y0IHP1_9GAMM    TAACVEFGKAQGKTVIVVKDGPGFYTTRILAPYLNEASRLLLEGFGPKDLDKALMQLGYP
tr|A0A316FZ14|A0A316FZ14_9GAMM    IAQCVAFGKEQGKTVIVVNDGAGFYVNRILAPYMNEAGRMVAKGVPIDTIDKALVQAGFP
tr|A0A498C231|A0A498C231_9GAMM    IATTVALGRAQGKSVIVVRDGAGFYVNRILAPYLNEACRLLQEGVAIDHIDRTLVRFGFP
tr|Q0A6T4|Q0A6T4_ALKEH            IATTVALGRTQGKTVIVVRDGAGFYVNRILAPYLNEACRLLQEGVAIDHIDRTLVRFGFP
tr|A0A3E0WL10|A0A3E0WL10_9GAMM    IATTVDIGRKQGKTPIVVKDGAGFYVNRILAPYLNEASHLLSEGVAIDRIDKTLTQFGFP
tr|A0A3S1BU16|A0A3S1BU16_9GAMM    IATTVDFGRRQGKTVIVVQDGAGFYVNRILAPYMNEAGHLLNEGAAVDAIDQALVQFGFP
tr|A0A7V8QFN9|A0A7V8QFN9_9GAMM    IATVVDVGRKQGKTPIVVKDGAGFYVNRILSPYINEAMHLLGSGVAIDRIDKALVQFGFP
tr|A0A1H8PPF9|A0A1H8PPF9_9GAMM    IATTVDVGRKQGKTVIVVKDGAGFYVNRILAPYINEACHMLSEGVAIDRIDETMLNFGFP
tr|A0A6H0J0V7|A0A6H0J0V7_9GAMM    IATTVDVGRKQGKTVIVVKDGAGFYVNRILAPYINEACHMLSEGVPIDRIDETMLNFGFP
tr|V5EZX6|V5EZX6_9VIBR            VATVVALARKQGKTPIVVKDSAGFYVNRILAPYMNEAAQVLMAEQEIEHIDSALLNFGFP
tr|A0A6I1QBM2|A0A6I1QBM2_9VIBR    VATVVALARKQGKTPIVVKDSAGFYVNRILAPYMNEAAQVLMAEQEIEHIDSALLNFGFP
tr|A0A511QRL5|A0A511QRL5_9VIBR    VATVVALARKQGKTPIVVKDTAGFYVNRILAPYMNEAAQVLMAEQEIEHIDNALLNFGFP
tr|A0A7Y0JZP4|A0A7Y0JZP4_9VIBR    VATVVALARKQGKTPIVVKDTAGFYVNRILAPYMNEAAQVLMAEQEIEHIDNALLNFGFP
tr|A0A193KD24|A0A193KD24_9VIBR    VATVVALARKQGKTPIVVKDTAGFYVNRILAPYMNEAAQVLMAEQEIEHIDNALLNFGFP
tr|A0A1S1HM72|A0A1S1HM72_PROST    IATAVALAKKQGKTAIVVGDDAGFYVNRILAPYLCEAAECLMHGESIEHIDSALVDFGFP
tr|A0A7T8I613|A0A7T8I613_9GAMM    IATAVALAKKQGKTAIVVGDDAGFYVNRILAPYLCEAAECLMHGESIEHIDSALVDFGFP
tr|A0A140NIF0|A0A140NIF0_PROSM    IATVVAFAKRQGKTAIVVGDDAGFYVNRILAPYLCEAAECLMSGESIEHIDSALVDFGFP
tr|A0A379GMB7|A0A379GMB7_PROST    IATVVAFAKRQGKTAIVVGDDAGFYVNRILAPYLCEAAECLMSGESIEHIDSALVDFGFP
tr|A0A379H393|A0A379H393_PROST    IATVVAFAKRQGKTAIVVGDDAGFYVNRILAPYLCEAAECLMSGESIEHIDSALVDFGFP
tr|B2Q0L5|B2Q0L5_PROST            IATVVAFAKRQGKTAIVVGDDAGFYVNRILAPYLCEAAECLMSGESIEHIDSALVDFGFP
tr|A0A6I3JUE9|A0A6I3JUE9_9GAMM    IATVVAFAKRQGKTAIVVGDDAGFYVNRILAPYLCEAAECLMAGESIEHIDSALVDFGFP
tr|A0A7D4P594|A0A7D4P594_YERMW    IATTVALARKQGKTAIVVADRAGFYVNRILAPYINEAARCLLDGEPIDSIDSALVDFGFP
tr|A0A7U7IVK6|A0A7U7IVK6_YEREN    IATTVALACKQGKTAIVVADRAGFYVNRILAPYINEAARCLLDGEPIDSVDKALVDFGFP
tr|A0A447RIF5|A0A447RIF5_YEREN    IATTVALACKQGKTAIVVADRAGFYVNRILAPYINEAARCLLDGEPIDSVDKALVDFGFP
tr|A0A7H4ZLV9|A0A7H4ZLV9_YERP4    IATTVALACKQGKTAIVVADRAGFYVNRILAPYINEAARCLLDGEPIDSVDKALVDFGFP
tr|A0A0H3NX46|A0A0H3NX46_YERE1    IATTVALACKQGKTAIVVADRAGFYVNRTLAPYINEAARCLLDGEPIDSVDKALVDFGFP
tr|A0A7T9XUV7|A0A7T9XUV7_YEREN    IATTVALACKQGKTAIVVADRAGFYVNRILAPYINEAARCLLDGEPIDSVDKALVDFGFP
tr|A0A7U7IYV8|A0A7U7IYV8_YEREN    IATTVALARKQGKTAIVVADRAGFYVNRILAPYINEAARCLLDGEPIDSVDKALVDFGFP
tr|A0A0H5G7G2|A0A0H5G7G2_YEREN    IATTVALARKQGKTAIVVADRAGFYVNRILAPYINEAARCLLDGEPIDSVDKALVDFGFP
tr|A0A2A7TDI2|A0A2A7TDI2_YERKR    IATTVALARKQGKTAIVVADCAGFYVNRILAPYINEAARCILDGEPIDSIDKALVDFGFP
tr|A0A0T9M261|A0A0T9M261_YERKR    IATTVALARKQGKTAIVVADCAGFYVNRILAPYINEAARCILDGEPIDSIDKALVDFGFP
tr|A0A0A0CMP1|A0A0A0CMP1_PHOLU    IATAVALAKKQGKTAIVVGDKAGFYVNRILVPYISEAAHCLVAGEPIDHIDGALVNFGFP
tr|A0A329VG55|A0A329VG55_9GAMM    IATAVSLAKKQGKTAIVVGDKAGFYVNRILVPYISEAAHCLVAGEPIDHIDGALVNFGFP
sp|Q7N288|FADJ_PHOLL              IATAVSLAKKQGKTAIVVGDKAGFYVNRILVPYISEAAHCLVAGEPIDHIDGALVNFGFP
tr|A0A6L9JMX5|A0A6L9JMX5_PHOLM    IATAVSLAKKQGKTAIVVGDKAGFYVNRILVPYISEAAHCLVAGEPIDHIDGALVNFGFP
tr|A0A7X5HQ29|A0A7X5HQ29_PHOLM    IATAVSLAKKQGKTAIVVGDKAGFYVNRILVPYISEAAHCLVAGEPIDHIDGALVNFGFP
tr|A0A1C0U051|A0A1C0U051_9GAMM    IATVVALAKKQGKTAIVVGDKAGFYVNRILVPYISEASNCLIAGEPIDRIDKALVNFGFP
tr|A0A7X5QF62|A0A7X5QF62_9GAMM    IATAVALAKKQGKTAIVVGDKAGFYVNRILVPYISEASNCLVAGEPIDHIDKALVNFGFP
```

```
tr|A0A2D0KWM9|A0A2D0KWM9_9GAMM  IATAVALAKKQGKTAIVVGDKAGFYVNRILSPYINEAGYCLVEGEPIEHIDKALVDFGFP
tr|A0A2D0LAI1|A0A2D0LAI1_9GAMM  IATVVALAKKQGKTAIVVGDKAGFYVNRILSPYINEAGYCLVEGEPIEHIDKALVDFGFP
tr|A0A2D0IXC8|A0A2D0IXC8_XENBU  IATAVALAKKQGKTAIVVGDKAGFYVNRILSPYINEAGYCLVEGEPVNHIDKALVDFGFP
tr|W1JAM5|W1JAM5_9GAMM          IATAVALAKKQGKTAIVVGDKAGFYVNRILSPYINEAGYCLVEGEPVNHIDKALVDFGFP
tr|A0A3D9UED3|A0A3D9UED3_9GAMM  IATAVALAKKQGKTAIVVGDKAGFYVNRILSPYINEAGYCLVEGEPVNHIDKALVDFGFP
tr|A0A1I3JCA9|A0A1I3JCA9_9GAMM  VATAVALAKKQGKTAIVVGDKAGFYVNRILAPYINEAGYCLVEGEPIEHIDKALVDFGFP
tr|A0A068QUL9|A0A068QUL9_9GAMM  IATAVALAKKQGKTAIVVGDKAGFYVNRILTPYINEAGYCLVEGEPIEHIDSALTNFGFP
tr|A0A0M0TCH6|A0A0M0TCH6_9GAMM  IATTVALAKKQGKTAIVVGDKAGFYVNRILTPYINEAGYCLLEGEPVEHIDNALLNFGFP
tr|A0A1Q5U854|A0A1Q5U854_9GAMM  IATAVALAKKQGKTAIVVGDKAGFYVNRILTPYINEAGYCVLEGEPIEHIDNALMNFGFP
tr|A0A2D0ISP1|A0A2D0ISP1_9GAMM  IATVVALAKKQGKTAIVVGDKAGFYVNRILTPYINEAGYCVLEGEPIEHIDNALMNFGFP
tr|A0A1Q5TUI8|A0A1Q5TUI8_9GAMM  IATAVALAKKQGKTAIVVGDKAGFYVNRILTPYINEAGYCVLEGEPIEHIDNALMNFGFP
tr|A0A2D0KJI7|A0A2D0KJI7_9GAMM  IATAVALAKKQGKTAIVVGDKAGFYVNRILTPYINEAGYCVLEGEPIEHIDNALMNFGFP
tr|A0A1Y2SB97|A0A1Y2SB97_9GAMM  IATAVALAKKQGKTAIVVGDKAGFYVNRILTPYINEAGYCLVEGEPIEHIDNALTNFGFP
tr|A0A1I5DWI7|A0A1I5DWI7_9GAMM  IATAVALAKKQGKTAIVVGDKAGFYVNRILTPYINEAGYCLVEGEPIEHIDNALMNFGFP
tr|A0A1I7GWY6|A0A1I7GWY6_9GAMM  IATAVSLAKKQGKTAIVVGDKAGFYVNRILTPYINEAGYCLVEGEPVDHIDKALVNFGFP
tr|D3VKY8|D3VKY8_XENNA          IATAVALAKKQGKTAIVVGDKAGFYVNRILTPYINEAGYCLVAGEPIDHIDKALVNFGFP
tr|A0A2G0Q6Q9|A0A2G0Q6Q9_9GAMM  IATVVALAKKQGKTAIVVGDKAGFYVNRILTPYINEAGYCLVEGEPVEHIDKALVGFGFP
tr|A0A2D0JU24|A0A2D0JU24_9GAMM  IATAVALAKKQGKTAIVVGDNAGFYVNRILAPYINEAGYCLLEGEPVEHIDKALVDFGFP
tr|A0A0J5FN38|A0A0J5FN38_9GAMM  IATAVALAKKQGKTAIVVGDNAGFYVNRILAPYINEAGYCLLEGEPVDHIDNALVDFGFP
tr|A0A432XLD4|A0A432XLD4_9GAMM  IATTVALAKKQGKTPIVVKDGAGFYVNRILAPYMNEAARLVLEGEPIEVVDQALVKFGFP
tr|A0A2D8HU84|A0A2D8HU84_9GAMM  IATTVSLAKKQGKTPIVVKDGAGFYVNRILAPYMNEAARLLLAGEPIEHLDKTLVKFGFP
tr|A0A656X1Q2|A0A656X1Q2_9GAMM  IATTVSLAKKQGKTPIVVKDGAGFYVNRILAPYMNEAARLLLAGEPIEHLDKTLVKFGFP
tr|A0A4Q1QH79|A0A4Q1QH79_9GAMM  IATTVALAKKQGKTPIVVKDGAGFYVNRILAPYMNEAARLMLAGEPIEKLDKTLVKFGFP
tr|A0A1G7LQE6|A0A1G7LQE6_9GAMM  IATTVALAKKQGKTPIVVKDGAGFYVNRILAPYMNEAARLMLAGEPIEKLDKTLVKFGFP
tr|A0A432YVG7|A0A432YVG7_9GAMM  IATTVALAKKQGKTPIVVKDGAGFYVNRILAPYMNEAARLMLAGEPIEKLDKTLVKFGFP
tr|A0A1J5N0P5|A0A1J5N0P5_9GAMM  IATTVAFAKKQGKTPIVVKDGAGFYVNRILALYVNEAANVLLEGDSIESLDKALVEFGFP
tr|A0A0D8D548|A0A0D8D548_9GAMM  ISTTVAFAKKQGKTPIVVKDKAGFYVNRILAPYMNEAANLLLEGESIEKLDKTLVNFGFP
tr|A0A3E0TNM2|A0A3E0TNM2_9GAMM  ISTTVAFAKKQGKTPIVVKDKAGFFVNRILAPYMNEAARMLLAGEPIEKLDKALVKFGFP
tr|A0A3E0UD67|A0A3E0UD67_9GAMM  ISTTVAFAKKQGKTPIVVKDKAGFFVNRILAPYMNEAARMLLAGEPIEKLDKALVKFGFP
tr|A0A3E0U0P4|A0A3E0U0P4_9GAMM  ISTTVAFAKKQGKTPIVVKDKAGFFVNRILAPYMNEAARMLLAGEPIEKLDKALVKFGFP
tr|A0A0M2V8B1|A0A0M2V8B1_9GAMM  IATTVAFARKQGKTPIVVKDGAGFYVNRILALYMNEAATILLAGEPVEKIDRALVKFGFP
tr|A0A285ITY5|A0A285ITY5_9GAMM  IATTVAFARKQGKTPIVVKDGAGFYVNRILALYMNEAANLLLTGEPVEKIDKALVKFGFP
tr|A0A486XTB9|A0A486XTB9_9GAMM  IANTVAFARKQGKTPIVVKDGAGFYVNRILALYMNEAATILLEGEPVEKIDQALVKFGFP
tr|I1E1G1|I1E1G1_9GAMM          IATTVAFARKQGKTPIVVKDGAGFYVNRILALYMNEAANVLLEGEPVEKIDKALVKFGFP
tr|A0A1H6KJ68|A0A1H6KJ68_9GAMM  IATTVAFARKQGKTPIVVKDGAGFYVNRILALYMNEAANILLEGEPVEKIDKALVKFGFP
tr|A0A0X3Y764|A0A0X3Y764_9GAMM  IATTVAFARKQGKTPIVVKDGAGFYVNRILALYMNEAANILLEGEPVEKIDKALVKFGFP
tr|A0A2N1YEV4|A0A2N1YEV4_9GAMM  IATTVAFARKQGKTPIVVKDGAGFYVNRILALYMNEAANILLEGEPVEKIDKALVKFGFP
tr|A0A3P3QCM3|A0A3P3QCM3_9GAMM  IATTVAFARKQGKTPIVVKDGAGFYVNRILALYMNEAANILLEGEPVEALDKALVKFGFP
tr|F7NT72|F7NT72_9GAMM          IATTVAFARKQGKTPIVVKDGAGFYVNRILALYMNEAANILLEGEPVEALDKALVKFGFP
tr|A0A3S2TW63|A0A3S2TW63_9GAMM  IATTVAFARKQGKTPIVVKDGAGFYVNRILALYMNEAANILLEGEPVEALDKALVKFGFP
tr|A0A3D5BGW7|A0A3D5BGW7_9GAMM  IATTVAFARKQGKTPIVVKDGAGFYVNRILALYMNEAANLLLEGEPVEKLDKALTKFGLP
tr|A0A5C7TI17|A0A5C7TI17_9GAMM  IATTVAFARKQGKTPIVVKDGAGFYVNRILALYMNEAANLLLEGESVEKLDKALTRFGFP
tr|A0A0U4W982|A0A0U4W982_9GAMM  IATTVAFARKQGKTPIVVKDGAGFYVNRILALYMNEAANLLLEGESVEKLDKALTRFGFP
tr|A0A2I0FCM2|A0A2I0FCM2_9GAMM  ISTTVEFARKQGKTPIVVKDGAGFYVNRILAPYMNEAARLVLEQEPIEAVDKALVDFGFP
tr|A0A2G2IVL1|A0A2G2IVL1_9GAMM  ISTTVEFARKQGKTPIVVKDGAGFYVNRILAPYMNEAARLVLEQEPIEAVDKALVDFGFP
tr|A6FI21|A6FI21_9GAMM          ISTTVEFARKQGKTPIVVKDGAGFYVNRILAPYMNEAARLVLEQEPIEAVDKALVDFGFP
tr|A0A4U1BNR2|A0A4U1BNR2_9GAMM  IATTVKFAKKQGKTPIVVKDCAGFYVNRILAPYMNEAANLLLEGEAIEKLDNSLVNFGFP
tr|E1SQ60|E1SQ60_FERBD          IATTVAFARKQGKTPIVVQDGAGFYVNRILALYMNEAANLLLDGEPVEALDKALVKFGFP
tr|A0A4Y6IZ09|A0A4Y6IZ09_9GAMM  IATTVAFARKQGKTPIVVKDGAGFYVNRILALYMNEAANLLLEGQSVDHLDKALVKFGFP
tr|A0A0C3QSZ7|A0A0C3QSZ7_9GAMM  IATTVAFARKQGKTPIVVKDGAGFYVNRILALYMNEAANLLLEGQSVDHLDKALVKFGFP
tr|A0A6L7HXW0|A0A6L7HXW0_9GAMM  IATTVAFARKQGKTPIVVKDGAGFYVNRILALYMNEAANLLLEGQSVDHLDKALVKFGFP
sp|A3QFP3|FADJ_SHELP            IATTVAFARKQGKTPIVVKDGAGFYVNRILALYMNEAANLLLEGQSVDHLDKALVKFGFP
tr|A0A1E5IXH7|A0A1E5IXH7_SHECO  IATTVAFARKQGKTPIVVQDGAGFYVNRILALYMNEAANLLLQGESVEHLDKALVKFGFP
tr|A0A411PKQ0|A0A411PKQ0_9GAMM  IATTVAFARKQGKTPIVVQDGAGFYVNRILALYMNEAAQWLLEGQRVEHLDKALVKFGFP
tr|A0A6G9QKM3|A0A6G9QKM3_9GAMM  IATTVAFARKQGKTPIVVQDGAGFYVNRILALYMNEAAQLLLEGQRIEHLDRSLVKFGFP
tr|A0A6P1UL63|A0A6P1UL63_9GAMM  IATTVAFARKQGKTPIVVQDGAGFYVNRILALYMNEAAQLLLEGQRVEHLDRALVKFGFP
tr|A0A2N1ERI9|A0A2N1ERI9_9GAMM  IATTVAFARKQGKTPIVVQDGAGFYVNRILALYMNEAAQLLLEGQRVEHLDRALVKFGFP
tr|A0A7W4FU55|A0A7W4FU55_9GAMM  IATTVAFARKQGKTPIVVQDGAGFYVNRILALYMNEAAQLLLEGQRVEHLDRALVKFGFP
sp|Q8ECP7|FADJ_SHEON            IATTVAFARKQGKTPIVVQDGAGFYVNRILALYMNEAAQLLLEGQSVEHLDKALVKFGFP
tr|A0A501XZY8|A0A501XZY8_9GAMM  IATTVAFARKQGKTPIVVQDGAGFYVNRILALYMNEAAQLLLEGQSVEHLDKALVKFGFP
tr|A0A2W5DCZ0|A0A2W5DCZ0_SHEOE  IATTVAFARKQGKTPIVVQDGAGFYVNRILALYMNEAAQLLLEGQSVEHLDKALVKFGFP
tr|A0A1E3V3C8|A0A1E3V3C8_9GAMM  IATTVAFARKQGKTPIVVQDGAGFYVNRILALYMNEAAQLLLEGQSVEHLDKALVKFGFP
tr|A0A1Z4AI20|A0A1Z4AI20_9GAMM  IATTVAFARKQGKTPIVVQDGAGFYVNRILALYMNEAAQLLLEGQSVEHLDKALVKFGFP
tr|A0A7X9LJL9|A0A7X9LJL9_9GAMM  IATTVAFARKQGKTPIVVQDGAGFYVNRILALYMNEAAQLLLEGQSVEHLDKALVKFGFP
tr|A0A073KMY5|A0A073KMY5_9GAMM  IATTVAFARKQGKTPIVVQDGAGFYVNRILALYMNEAAQLLLEGQSVEHLDKALVKFGFP
sp|A0KV76|FADJ_SHESA            IATTVAFARKQGKTPIVVQDGAGFYVNRILALYMNEAAQLLLEGQSIEHLDKALVKFGFP
tr|A0A220UTH7|A0A220UTH7_9GAMM  IATTVAFARKQGKTPIVVQDGAGFYVNRILALYMNEAAQLLLEGQSIEHLDKALVKFGFP
tr|A0A5B8R6W1|A0A5B8R6W1_9GAMM  IATTVAFARKQGKTPIVVQDGAGFYVNRILALYMNEAAQLLLEGQSIEHLDKALVKFGFP
tr|V1DAI4|V1DAI4_9GAMM          IATTVAFARKQGKTPIVVQDGAGFYVNRILALYMNEAAQLLLEGQSIEHLDKALVKFGFP
tr|A0A448CPQ4|A0A448CPQ4_SHEPU  IATTVAFARKQGKTPIVVQDGAGFYVNRILALYMNEAAQLLLEGQSIEHLDKALVKFGFP
tr|A0A252ERQ3|A0A252ERQ3_SHEPU  IATTVAFARKQGKTPIVVQDGAGFYVNRILALYMNEAAQLLLEGQSIEHLDEALVKFGFP
sp|Q0HKD1|FADJ_SHESM            IATTVAFARKQGKTPIVVQDGAGFYVNRILALYMNEAAQLLLEGQSIEHLDKALVKFGFP
sp|Q0HWN3|FADJ_SHESR            IATTVAFARKQGKTPIVVQDGAGFYVNRILALYMNEAAQLLLEGQSIEHLDKALVKFGFP
tr|F7RQE3|F7RQE3_9GAMM          IATTVAFARKQGKTPIVVQDGAGFYVNRILALYMNEAAQLLLEGQSVEHLDKALVKFGFP
tr|B8EE98|B8EE98_SHEB2          IATTVAFARKQGKTPIVVQDGAGFYVNRILALYMNEAAQLLLEGQSVEHLDKALVKFGFP
sp|A6WQ25|FADJ_SHEB8            IATTVAFARKQGKTPIVVQDGAGFYVNRILALYMNEAAQLLLEGQSVEHLDKALVKFGFP
tr|A0A448EK41|A0A448EK41_9GAMM  IATTVAFARKQGKTPIVVQDGAGFYVNRILALYMNEAAQLLLEGQSVEHLDKALVKFGFP
tr|A0A553JHX1|A0A553JHX1_SHEHA  IATTVAFARKQGKTPIVVQDGAGFYVNRILALYMNEAAQLLLEGKSVEGLDKALVKFGFP
tr|B8CPY6|B8CPY6_SHEPW          IATTVAFARKQGKTPIVVQDGAGFYVNRILALYMNEAAQLLLEGQSVEHLDKALVKFGFP
tr|A0A431WFC4|A0A431WFC4_9GAMM  IATTVAFARKQGKTPIVVQDGAGFYVNRILALYMNEAAQLLLEGQSVEHLDKALVKFGFP
tr|A8FTR7|A8FTR7_SHESH          IATTVAFARKQGKTPIVVQDGAGFYVNRILALYMNEAAQLLLEGQSVEHLDKALVKFGFP
tr|A0A431WNL0|A0A431WNL0_9GAMM  IATTVAFARKQGKTPIVVQDGAGFYVNRILALYMNEAAQLLLEGQSVEHLDKALVKFGFP
tr|A0A550AEC5|A0A550AEC5_9GAMM  IATTVAFARKQGKTPIVVQDGAGFYVNRILALYMNEAAQLLLEGQSVEHLDKSLVKFGFP
tr|A0A7L4WW90|A0A7L4WW90_9GAMM  IATTVAFARKQGKTPIVVQDGAGFYVNRILALYMNEAAQLLLEGQSVEHLDKSLVKFGFP
tr|A9DDU3|A9DDU3_9GAMM          IATVVAFARKQGKTPIVVQDGAGFYVNRILALYMNEAAQLLLEGQSVDHLDKALVKFGFP
tr|A0A330M2B9|A0A330M2B9_9GAMM  IATVVAFARKQGKTPIVVQDGAGFYVNRILALYMNEAAQLLLEGQSVDHLDKALVKFGFP
tr|A0A5N8UFC7|A0A5N8UFC7_9GAMM  IATVVAFARKQGKTPIVVQDGAGFYVNRILALYMNEAAQLLLEGQSVEHLDKALVKFGFP
tr|A0A1S6HN57|A0A1S6HN57_9GAMM  IATTVAFARKQGKTPIVVQDGAGFYVNRILALYMNEAAQLLLEGQSVEHLDKALVKFGFP
tr|D4ZMH7|D4ZMH7_SHEVD          IATTVAFARKQGKTPIVVQDGAGFYVNRILALYMNEAAQLLLEGQSVEHLDKALVKFGFP
tr|A0A3L8Q213|A0A3L8Q213_9GAMM  IDTTVAFARKQGKTPIVVKDGAGFYVNRILVLYMLEAAQVLLEGQRVEHLDKALVKFGFP
tr|A0A3A6U4N9|A0A3A6U4N9_9GAMM  IATTVNFARKQGKTPIVVKDGAGFYVNRILALYMNEASQVLLEGQRIEHLDKSLVKFGFP
tr|A0A4Q5MA37|A0A4Q5MA37_9GAMM  IDTTVAFARKQGKTPIVVQDGAGFYVNRILALYMNEAASVLLEGQRIEHLDKSLVKFGFP
tr|A0A1L6LSX5|A0A1L6LSX5_9DELT  VATAVSIGKKQGKTVIVVNDGTGFYTSRILGPMMNEASYLLTEGLSVETIDKAMTAWGWP
tr|A0A2W4L9B9|A0A2W4L9B9_9PROT  VATAVALGKAQGKTVIVVNDGPGFYTSRILAPYMMEAAWLLAEGGDIAQIDEAMRDWGFP
tr|A0A2W4M4E6|A0A2W4M4E6_9PROT  TRTAVAIGKRQGKTVIVVRDGPGFYTSRILGPYLNEASYLLADGYAIDVIDDALVKWGFP
tr|A0A6I2GRX9|A0A6I2GRX9_9DELT  TATCVEVGRRQGKTVIVVNDGVGFYTSRILAPYMNEAAYLLAEGADIRELDRALTDFGFP
tr|A0A0H4WMK2|A0A0H4WMK2_9DELT  TATCVEVGRKQGKTVIVVNDGPGFYTSRILAPYMNEAAYLLGEGADIAELDKALVEFGFP
tr|F8CJ36|F8CJ36_MYXFH          TATCVEVGRKQGKTVIVVNDGPGFYTSRILAPYMNEAAYLLAEGADIAELDRALVEFGFP
tr|A0A250K0F1|A0A250K0F1_9DELT  TATCVEVGRKQGKTVIVVNDGPGFYTSRILAPYMNEAAYLLAEGADIAELDRALVEFGFP
tr|A0A7Y6WFZ2|A0A7Y6WFZ2_9DELT  TATCVEVGRKQGKTVIVVNDGPGFYTSRILAPYMNEAAYLLAEGADIAELDRALVEFGFP
tr|A0A7Y7C660|A0A7Y7C660_9DELT  TATCVEVGRKQGKTVIVVNDGPGFYTSRILAPYMNEAAYLLAEGADIAELDRALVEFGFP
tr|A0A7Y4JFH2|A0A7Y4JFH2_MYXXA  TATCVEVGRKQGKTVIVVNDGPGFYTSRILAPYMNEAAYLLAEGADIAELDRALVEFGFP
tr|A0A4Y6CZQ9|A0A4Y6CZQ9_MYXXA  TATCVEVGRKQGKTVIVVNDGPGFYTSRILAPYMNEAAYLLAEGADIAELDRALVEFGFP
```

```
tr|A0A7Y4IKV5|A0A7Y4IKV5_MYXXA  TATCVEVGRKQGKTVIVVNDGPGFYTSRILAPYMNEAAYLLAEGADIAELDRALVEFGFP
tr|A0A511HHB0|A0A511HHB0_9DELT  TATCVEVGRKQGKTVIVVNDGPGFYTSRILAPYLNEAAYLLAEGADIAELDRALVEFGFP
tr|A0A4Y6CKY7|A0A4Y6CKY7_MYXXA  TATCVEVGRKQGKTVIVVNDGPGFYTSRILAPYMNEAAYLLAEGADIAELDRALVEFGFP
tr|Q1D1F2|Q1D1F2_MYXXD          TATCVEVGRKQGKTVIVVNDGPGFYTSRILAPYMNEAAYLLAEGADIAELDRALVEFGFP
tr|A0A7Y4MA14|A0A7Y4MA14_MYXXA  TATCVEVGRKQGKTVIVVNDGPGFYTSRILAPYMNEAAYLLAEGADIAELDRALVEFGFP
tr|A0A7T8Y4N9|A0A7T8Y4N9_MYXXA  TATCVEVGRKQGKTVIVVNDGPGFYTSRILAPYMNEAAYLLAEGADIAELDRALVEFGFP
tr|L7UE67|L7UE67_MYXSD          TATCVDVGRRQGKTVIVVNDGPGFYTSRILAPYLNEAAYLLGEGADIAALDKALVEFGFP
tr|A0A511T9X1|A0A511T9X1_MYXFU  TATCVEVGRKQGKTVIVVNDGPGFYTSRILAPYLNEAAYLLAEGADIAALDKALVEFGFP
tr|A0A7Y7C9C4|A0A7Y7C9C4_9DELT  TATCVEVGRKQGKTVIVVNDGPGFYTSRILAPYMNEAAHLLAEGADIAALDKALVEFGFP
tr|A0A540X7W8|A0A540X7W8_9DELT  TATCVEVGRKQGKTVIVVNDGPGFYTSRILAPYMNEAAHLLAEGADIAALDKALVEFGFP
tr|A0A3A5FK19|A0A3A5FK19_9DELT  TATCVEVGRKQGKTVIVVNDGPGFYTSRILAPYMNEAAYLLADGADITELDKALVEFGFP
tr|A0A3A8JQL9|A0A3A8JQL9_9DELT  TATCVDVGKKQGKTVIVVNDGPGFYTSRILAPYMNEAAYLLAEGADIVQLDKALVDFGFP
tr|A0A7Y4NFA4|A0A7Y4NFA4_9DELT  TATCVDVGKKQGKTVIVVNDGPGFYTSRILAPYMNEAAYLLAEGADIVQLDKALVDFGFP
tr|A0A3A8HDQ7|A0A3A8HDQ7_9DELT  TATCVDVGKKQGKTVIVVNDGPGFYTSRILAPYMNEAAYLLAEGADIVQLDKALVDFGFP
tr|A0A3A8GR90|A0A3A8GR90_9DELT  TATCVDVGKKQGKTVIVVNDGPGFYTSRILAPYMNEAAYLLAEGADIVQLDKALVDFGFP
tr|A0A3A8SBD7|A0A3A8SBD7_9DELT  TATCVDVGKKQGKTVIVVNDGPGFYTSRILAPYMNEAAYLLAEGADIVQLDKALVDFGFP
tr|A0A3A8T0I8|A0A3A8T0I8_9DELT  TATCVDVGKKQGKTVIVVNDGPGFYTSRILAPYMNEAAYLLAEGADIVQLDKALVDFGFP
tr|A0A7X5BU07|A0A7X5BU07_9DELT  TATCVDVGKKQGKTVIVVNDGPGFYTSRILAPYMNEAAYLLAEGADIVQLDKALVDFGFP
tr|A0A7Y1RVL2|A0A7Y1RVL2_9DELT  TATCVDVGKKQGKTVIVVNDGPGFYTSRILAPYMNEAAYLLAEGADIVQLDKALVDFGFP
tr|A0A3A8THN2|A0A3A8THN2_9DELT  TATCVDVGKKQGKTVIVVNDGPGFYTSRILAPYMNEAAYLLAEGADIVQLDKALVDFGFP
tr|A0A7Y1RX76|A0A7Y1RX76_9DELT  TATCVDVGKKQGKTVIVVNDGPGFYTSRILAPYMNEAAYLLAEGADIVQLDKALVDFGFP
tr|A0A3A8RA58|A0A3A8RA58_9DELT  TATCVDVGKKQGKTVIVVNDGPGFYTSRILAPYMNEAAYLLAEGADIVQLDKALVDFGFP
tr|A0A410RPB6|A0A410RPB6_CORCK  TATCVDVGKKQGKTVIVVNDGPGFYTSRILAPYMNEAAYLLAEGADIVQLDKALVDFGFP
tr|A0A7Y4J474|A0A7Y4J474_CORCK  TATCVDVGKKQGKTVIVVNDGPGFYTSRILAPYMNEAAYLLAEGADIVQLDKALVDFGFP
tr|A0A3A8I9Z6|A0A3A8I9Z6_9DELT  TATCVDVGKKQGKTVIVVNDGPGFYTSRILAPYMNEAAYLLAEGADIVQLDKALVDFGFP
tr|H8MKE9|H8MKE9_CORCM          TATCVDVGKKQGKTVIVVNDGPGFYTSRILAPYMNEAAYLLAEGADIVQLDKALVDFGFP
tr|A0A3A8H102|A0A3A8H102_9DELT  TATCVDVGKKQGKTVIVVNDGPGFYTSRILAPYMNEAAYLLAEGADIVQLDKALVDFGFP
tr|A0A554FW33|A0A554FW33_9DELT  TATCVDVGRKQGKTVIVVNDGPGFYTSRILAPYMNEAAYLLAEGADILQLDKALVDFGFP
tr|A0A3A8NPC0|A0A3A8NPC0_9DELT  TATCVDVGRKQGKTVIVVNDGPGFYTSRVLAPYMNEAAYLLAEGADIVQLDKALVDFGFP
tr|A0A3A8JUV3|A0A3A8JUV3_9DELT  TATCVDVGRKQGKTVIVVNDGPGFYTSRVLAPYMNEAAYLLAEGADIVQLDKALVDFGFP
tr|A0A3A8NEX6|A0A3A8NEX6_9DELT  TATCVDVGRKQGKTVIVVNDGPGFYTSRVLAPYMNEAAYLLAEGADIVQLDKALVDFGFP
tr|A0A3A8JEU8|A0A3A8JEU8_9DELT  TATCVDVGRKQGKTVIVVNDGPGFYTSRILAPYMNEAAHLLAEGADIVQLDKALVDFGFP
tr|A0A3A8LI76|A0A3A8LI76_9DELT  TATCVDVGRKQGKTVIVVNDGPGFYTSRILAPYMNEAAYLLAEGADIVQLDKALVDFGFP
tr|A0A085WXN8|A0A085WXN8_9DELT  TATCVEVGRKQGKTVIVVNDGVGFYTSRILAPYMNEAAYLLAEGADIAELDKALVEFGFP
tr|A0A2T4V0M5|A0A2T4V0M5_9DELT  TATCVEVGKKQGKTVIVVNDGPGFYTSRILAPYMNEAAHLLADGADIAELDKALVEFGFP
tr|A0A0G2ZSW5|A0A0G2ZSW5_9DELT  TATCVEVGKKQGKTVIVVNDGPGFYTSRILAPYMNEAAHLLADGADIAELDKALVEFGFP
tr|A0A3M2DKY0|A0A3M2DKY0_9DELT  TATCVALGKRQGKTVIVVRDGTGFYTSRILGPYMAEAAQLLAAGVPIDDIDAALIQFGFP
tr|A0A661NQ58|A0A661NQ58_9DELT  TATCVALGKAQGKTVIVVRDGVGFYTTRILAPFMNEAAHLLSEGAAVDRIDDALMDFGYP
tr|A0A520YD99|A0A520YD99_9DELT  TATCVKLGKKQGKHVIVVKDGVGFYTSRVLAPMMNEAAHLVAEGVPIEKIDSAMLDWGFP
tr|A0A7Y3BRE4|A0A7Y3BRE4_9DELT  TATCVKLGKKQGKHVIVVKDGVGFYTSRVLAPMMNEAAHLVAEGVPIEKIDSAMLDWGFP
tr|A0A2D9TF90|A0A2D9TF90_9DELT  TATCVKLGKKQGKTVIVVKDGVGFYTTRILAPMMNEAAHILSEGVAIDRIDRALLDFGFP
tr|A0A2E0TP32|A0A2E0TP32_9DELT  TATCVELGKKQGKTVIVVGDGPGFYTTRILAPLMNEAAHILAEGAPIEEIDEAMLDFGFP
tr|A0A2E4Y3V1|A0A2E4Y3V1_9PROT  ASLAVEMGMKQGKTVIVVNDGPGFYTTRILAPLTDEAAIIALEGTDLHHINNIMKDFGFP
tr|A0A2E6VRH4|A0A2E6VRH4_9DELT  AATAVDYGIRQGKTVIVVKDGPGFYTTRILAPYMDESAILLAEGINPYDLDTIMKKYGFP
tr|A0A1F9FB59|A0A1F9FB59_9DELT  KALAVDVGIRQGKTVIVVNDGPGFYTSRILGPLMDEAAQCALEGVALHLIDHAMRQWGFP
```

```
ECHA_HUMAN                             590       600        610        620        630
ECHA_HUMAN                         VGAATLVDEVGVDVAKHV..AEDLGKVFGERFGGGNPELLTQMVSKGFLGRKSGKGFY..
tr|A0A4R6XLA8|A0A4R6XLA8_9GAMM     VGPLALMDEVGIDVGAHI.ARGALGEMFVKR.GIEQDDTVQKLADAKLFGRKVGKGFY..
tr|A0A6A0IJT2|A0A6A0IJT2_9BACT     VGPITLLDEVGIDVAAHV..AEDLGKAFASR.GAAASPTLRKIAEAGLLGRKAGKGFY..
tr|A0A7V3QZU7|A0A7V3QZU7_9BACT     VGPVTLLDEVGIDVGAHV..AKELGPVYAHR.GLGASDALPRLFEAGYQGRKNGRGFY..
tr|A0A7V4SYJ6|A0A7V4SYJ6_9BACT     VGPVALLDEVGIDVGAHV..AKDFGDTFKDR.GLSSSEALPKLFAAGFQGRKNGKGFY..
tr|A0A2A8D2N6|A0A2A8D2N6_9BACT     MGPFELFDLVGIDVAAKI..TEVMRKHLDDD.QTKISDSAATLVEAGLKGQKTEQGFY..
tr|A0A2N1TGP0|A0A2N1TGP0_9SPIR     VGPMALLDEVGIDVGTHV..TRELAVIFKDR.GIAAPYDLGKIVEKGFKGRKSGKGLY..
tr|A0A1G3QGW4|A0A1G3QGW4_9SPIR     VGPMTLLDEVGIDVGIHV..IEEVRPIFEPR.GITSPVDFSVLSDKGFLGRKSKKGLY..
tr|A0A1G3QTY3|A0A1G3QTY3_9SPIR     VGPMTLLDEVGIDVGIHV..IEEVLPIFEPR.GITAPIDFTILTQKGFLGRKSKKGLY..
tr|A0A7X9L359|A0A7X9L359_9DELT     VGPVTLVDELGIDVAAHVAKGEV.KKLFDAR.GLKASEGYTKLFEKGYKGRKNRKGFY..
tr|A0A522CJY0|A0A522CJY0_9SPIR     VGPITLIDEVGIDVGAHV..SKGLGEAFASR.GMKSSSALPALLEKGYKGKKNKKGFY..
tr|A0A2N1RRJ3|A0A2N1RRJ3_9SPIR     VGPAALIDEVGIDVGAHV..AGELGPMFIKR.GNPASEGIPKLYAEGYMGKKNRKGLY..
tr|A0A2N2KFN7|A0A2N2KFN7_9DELT     VGPVALLDEVGIDVGAHV..AEVLSPLFVQR.GMAGSDSLPQLFAHGYYGKKNRKGFY..
tr|A0A1V6AXX9|A0A1V6AXX9_9DELT     VGPITLIDEVGIDVGAHV..AMGLGKMFAAR.GMQSSDAFPKLTAKGYKGKKNKKGFY..
tr|A0A2N2HZL0|A0A2N2HZL0_9DELT     VGPITLIDEVGIDVGAHV..AKELGGMFAAR.GVQPSDTFPKLIAQDYKGRKNKKGFY..
tr|A0A5E8ARP3|A0A5E8ARP3_9BACT     VGPITLIDEVGIDVGAHI.NKGDLEAFFNKR.GAKSSDLLNKLAEAGFKGRKNRKGFL..
tr|A0A662A7B1|A0A662A7B1_9BACT     VGPITLIDEVGIDVGAHI.SSGDLATLFEKR.DAKSNDLLKRMEVKGFKGKKNKKGFL..
tr|D7CV16|D7CV16_TRURR             VGPITLLDEVGIDVGAKI..NGVLKAPFAER.GIALAEGGEALMNAGFLGRKNRKGFY..
tr|A0A3C2AKX4|A0A3C2AKX4_9FLAO     VGPITLMDEVGIDVGYHIMTGDLIQHFIATRKGVKTSDGITKMYEAKYYGRKNKKGFY..
tr|A0A3B8ZLN3|A0A3B8ZLN3_9PLAN     VGPITLMDEVGIDVGAHIMSGRLIQSFLATREGVKMSQGLKLMYEAGYHGRKNKKGFF..
tr|A0A345UGV8|A0A345UGV8_9BACT     VGPIVLIDEVGIDVAAHV..ADVLGPLFRER.GATPSDSARKLYDAGFRGRKNAKGFY..
tr|A0A6C1P6A2|A0A6C1P6A2_9BACT     VGPLVLMDEVGIDVAAHV..AGVMGAKFKDR.GVEGSKAAEKLSEAGYAGRKNDKGFY..
tr|A0A3M1X9W8|A0A3M1X9W8_9BACT     VGPFKLMDEVGIDVGAHV..SRVLAPLFAKR.GVTATDAMEKLVAQGFKGRKTGKGFY..
tr|A0A354C796|A0A354C796_9DELT     VGPIALIDEVGIDVGAHV..SNVMGPLFSIR.GAIPNTAMEKLFAAGYRGRKNNRGFY..
tr|A0A3M1NUM8|A0A3M1NUM8_9BACT     VGPFTLMDEVGMDVGAHV..AEILKEMFAER.GVQSTDAMEKLVEAGYKGRKSRKGFY..
tr|A0A3M2KRC8|A0A3M2KRC8_9BACT     VGPITLMDEVGLDVGAHV..SDFLGKKFADR.GVTPTDVLQKMVEEGYLGRKNGKGFY..
tr|A0A5S9IHR0|A0A5S9IHR0_9BACT     VGPITLLDEVGLDVAAHV..TDVMNDLFSKR.GVTGSNVAKQITDAGLKGRKNGKGFY..
tr|A0A6M1ST81|A0A6M1ST81_9BACT     VGPAALFDEVGIDVAAHI..TEVLNPMFSER.GVEPSAKPQELFDDGYKGKKNKKGFY..
tr|A0A521AAE5|A0A521AAE5_9BACT     VGPLALFDEVGIDVSAHI..TEVLGELFAQR.GFNPSKKPTTLLEEGYKGKKNKMGFY..
tr|A0A1M4ZMD6|A0A1M4ZMD6_9BACT     VGPAALFDEVGIDVAAHI..TEVLSDLFAER.DFHPSDKPTTLFEEGYKGKKNRQGFY..
tr|A0A2A2GCS2|A0A2A2GCS2_9BACT     VGPATLFDEVGIDVAAHI..TEVLSDLFADR.GVHPSKKPTELFEDGYKGKKNKKGFY..
tr|A0A5D3YIH7|A0A5D3YIH7_9BACT     VGPAALFDEVGIDVAAHI..TEVLSDLFAER.GIQSSQKPTELFEEGYKGRKNQKGFY..
tr|A0A6M1T9G0|A0A6M1T9G0_9BACT     VGPAALFDEVGIDVAAHI..TKVLNDLFAER.GVNPSTKPEELFEAGYKGRKNKKGFY..
tr|A0A6A8Q1Y8|A0A6A8Q1Y8_9BACT     VGPMALFDEVGIDVGAHV..AETMAPMFKER.GMKYNNKAEELLEDEYRGRKNKRGMY..
tr|A0A521BVJ3|A0A521BVJ3_9BACT     VGPMALFDEVGIDVGAHV..AETMSPMFAER.GIDTNNRAQELLDAGYKGRKNKKGLY..
tr|A0A359E0Y8|A0A359E0Y8_9BACT     VGPMALFDEVGIDVGAHV..AETMAPMFAER.GVESTNKADELLEAGFKGRKNKRGMY..
tr|A0A2D8CIM6|A0A2D8CIM6_9BACT     VGPMALFDEVGIDVGAHV..AETMAPMFAER.GVESTNKADELLEAGFKGRKNKRGMY..
tr|A0A2D9FWF5|A0A2D9FWF5_9BACT     VGPMALFDEVGIDVGAHV..AETMSPVFAER.GANTDNRAQELLDAGYKGRKNDKGMY..
tr|A0A1B6YB70|A0A1B6YB70_9BACT     VGPMALLDEVGFDVGAHV..GETMKPMFDKR.GARSSEKAQELVDEGYLGRKNKKGMY..
tr|A0A2D4ZTC0|A0A2D4ZTC0_9BACT     VGPMALLDEVGFDVGAHV..GETMKPMFDKR.GAKSSEKAKELVDEGYLGRKNKKGMY..
tr|A0A3D4UVW7|A0A3D4UVW7_9BACT     VGPMALLDEVGFDVGAHV..GETMKPMFDKR.GAKSSEKAKELVDEGYLGRKNKKGMY..
tr|A0A3F3I3M2|A0A3F3I3M2_9BACT     VGPMALLDEVGFDVGAHV..GETMKPMFDKR.GAKSSEKAKELVDEGYLGRKNKKGMY..
tr|A0A3D1G7L9|A0A3D1G7L9_9BACT     VGPMALLDEVGMDVGAHV..GETMAPMFDAR.GGKSSTKAKELMDAGFLGRKNKKGMY..
tr|A0A3M8G1Y8|A0A3M8G1Y8_9BACT     VGPMALLDEVGFDVGAHV..GETMSPVFNAR.GGKSSTKAKELMDAGFLGRKNKKGLY..
tr|A0A5Q4F391|A0A5Q4F391_9BACT     VGPIALFDEVGIDVGAHV..SEVLGALFRER.GAKTSSKAAELAEAGYLGRKNGKGFY..
tr|A0A6I7NPV0|A0A6I7NPV0_9BACT     VGPVALFDEVGIDVGAHV..AGVLGKLFKVR.GAKTSNKAKELMDAGFLGRKNRKGFY..
tr|A0A651G1J2|A0A651G1J2_9BACT     VGPVALFDEVGIDVGAHV..ADVLSDKFESR.GAKTSKKARELVDADFKGRKNGKGFY..
tr|A0A371QRK5|A0A371QRK5_9BACT     VGPVALLDEVGIDVGAHV..AGVLSDTFEER.GAKTSKKAAELMEAGYKGRKNNKGFY..
tr|A0A2N0VGJ5|A0A2N0VGJ5_9BACT     VGPVALFDEVGIDVGAHV..AEVLSDKFESR.GAKTSKKAKELVDAGYKGRKNKKGFY..
tr|A0A316TYJ0|A0A316TYJ0_9BACT     VGPVALFDEVGIDVGAHV..ADTLSDIFEER.GAKTSKKAAELVDKGYKGRKNGKGFY..
tr|A0A651GLZ8|A0A651GLZ8_9BACT     VGPVALLDEVGIDVAAHV..AETLSNKFEAR.GAKTSKKAKELVEAGYKGRKNRKGFY..
tr|A0A7Y5V3S5|A0A7Y5V3S5_9BACT     VGPLTLLDEVGLDIASKS..AAILHEAFGER..LAPAPAMRAIVSAGRTGRKGKSGFY..
tr|A0A7W1SHC0|A0A7W1SHC0_9BACT     VGPITLMDEVGIDVGGKV..GLVLSQAFGTR..MAPSDAMRKVVESGRTGRKGGKGFY..
tr|A0A7Y5PBT7|A0A7Y5PBT7_9BACT     VGPITLLDEVGIDVGGKV..GQVLYEAFGER..MASSESLRRVVESGRTGRKGKKGFY..
tr|A0A7Y5TXL8|A0A7Y5TXL8_9BACT     VGPITLLDEVGIDVGGKV..GQVLYEAFGER..MASSESLRRVVESGRTGRKGKKGFY..
tr|A0A423PQ96|A0A423PQ96_9GAMM     VGPMTLRDEVGVDVGVKV..GPILEAAFGER..MSAPDASQRMIEAGHLGRKSGKGFY..
tr|U2G066|U2G066_9GAMM             VGPLTLLDEVGVDVGVKV..GPILEAAFGPR..MAAPDASAKMIDAGYLGRKSGKGFY..
tr|A0A2E0J1F4|A0A2E0J1F4_9GAMM     VGPMTLLDEVGVDVGVKV..GPILEAAFGER..MAAPNASAKMIEAGHLGRKSGKGFY..
tr|A0A2D4SCM8|A0A2D4SCM8_9GAMM     VGPITLMDEVGIDVGTKI..GPILEKAFGER..MAVPGDANKMIEAGYAGRKTGKGFY..
tr|L0WJH1|L0WJH1_9GAMM             VGPITLLDEVGVDVGTKV..GPVLEKAFGER..MASPDAAGLMIENNYLGRKSGRGFY..
tr|A0A1H5XF61|A0A1H5XF61_9GAMM     VGPITLLDEVGVDVGTKV..GPVLEKAFGER..MASPDAAGLMIENDYLGRKSGRGFY..
tr|A0A2E9TS48|A0A2E9TS48_9GAMM     VGPITLLDEVGVDVGTKV..GPVLEKAFGER..MASPDAAGLMIENDYLGRKSGRGFY..
tr|A0A095UE19|A0A095UE19_9GAMM     VGPITLLDEVGVDVGTKV..GPVLEKAFGDR..MASPDAAGLMIENDYLGRKSGRGFY..
tr|A0A7G2S9A0|A0A7G2S9A0_9GAMM     VGPITLLDEVGVDVGTKV..GPVLEKAFGDR..MASPDAAGLMIENDYLGRKSGRGFY..
tr|B5JU27|B5JU27_9GAMM             VGPITLLDEVGIDVGTKV..GPVLEAAFGER..MQSPDAADRMIEKGFLGRKAGKGFY..
tr|A0A1Y0IHP1|A0A1Y0IHP1_9GAMM     VGPITLLDEVGIDVGTKV..GPILEAAFGER..MASPEAANLMIENGFLGRKAGKGFY..
tr|A0A316FZ14|A0A316FZ14_9GAMM     VGAMTLLDEVGIDVATKV..APILQDAFGER..MEPPAMFDKLKEDDRKGRKNGKGFY..
tr|A0A498C231|A0A498C231_9GAMM     VGPFALLDEVGLDVTEKV..APILHRAFGER..MTPVDVGERMLADGRKGRKSGKGFY..
tr|Q0A6T4|Q0A6T4_ALKEH             VGPFALLDEVGLDVSEKV..APILHRAFGER..MTPVDVGGRMLADGRKGRKSGKGFY..
tr|A0A3E0WL10|A0A3E0WL10_9GAMM     VGAFALLDEVGLDVGTKV..GPILHEAFGDR..MKPVESGDKMLKDGRYGKKSKKGFY..
tr|A0A3S1BU16|A0A3S1BU16_9GAMM     VGPFALLDEVGIDVGSKV..GPILHQAFGER..MKPAGVADKLLADGRYGRKSRKGFY..
tr|A0A7V8QFN9|A0A7V8QFN9_9GAMM     VGPFKLLDEVGIDISAHV..APILHEAFGER..MKPVSEAGKMIDDGRLGKKAGKGFY..
tr|A0A1H8PPF9|A0A1H8PPF9_9GAMM     VGPFALLDEVGLDVTAKV..APILHDAFGDR..MKPVAAADAMLSDGRYGKKSQKGFY..
tr|A0A6H0J0V7|A0A6H0J0V7_9GAMM     VGPFALLDEVGLDVTAKV..APILHEAFGDR..MKPVAEANRMIEDGRYGKKSEKGFY..
tr|V5EZX6|V5EZX6_9VIBR             VGPITLLDEVGVDIGAKI..MPILVSELGER..FQGPNLFDVLLNDGRIGRKAGKGFY..
tr|A0A6I1QBM2|A0A6I1QBM2_9VIBR     VGPITLLDEVGVDIGAKI..MPILVSELGER..FQGPNLFDVLLNDGRIGRKAGKGFY..
tr|A0A511QRL5|A0A511QRL5_9VIBR     VGPITLLDEVGVDIGAKI..MPILVKELGER..FQGPDLFDTLLNDGRKGRKVGKGFY..
tr|A0A7Y0JZP4|A0A7Y0JZP4_9VIBR     VGPITLLDEVGVDIGAKI..MPILVKELGER..FQGPDLFDTLLNDGRKGRKVGKGFY..
tr|A0A193KD24|A0A193KD24_9VIBR     VGPITLLDEVGVDIGAKI..MPILVKELGER..FQGPDLFDTLLNDGRKGRKVGKGFY..
tr|A0A1S1HM72|A0A1S1HM72_PROST     VGPFNLLDEVGIDVGTKI..LPILVERFGER..FMAPELLDKVNKDGRKGKKNGKGFY..
tr|A0A7T8I613|A0A7T8I613_9GAMM     VGPFNLLDEVGIDVGTKI..LPILVERFGER..FMAPELLDKVNKDGRKGKKNGKGFY..
tr|A0A140NIF0|A0A140NIF0_PROSM     VGPFNLLDEVGIDVGTKI..LPILVNRFGDR..FKAPELLDKVNKDDRKGKKNGKGFY..
tr|A0A379GMB7|A0A379GMB7_PROST     VGPFNLLDEVGIDVGTKI..LPILVNRFGDR..FKAPELLDKVNKDDRKGKKNGKGFY..
tr|A0A379H393|A0A379H393_PROST     VGPFNLLDEVGIDVGTKI..LPILVNRFGDR..FKAPELLDKVNKDDRKGKKNGKGFY..
tr|B2Q0L5|B2Q0L5_PROST             VGPFNLLDEVGIDVGTKI..LPILVNRFGDR..FKAPELLDKVNKDDRKGKKNGKGFY..
tr|A0A6I3JUE9|A0A6I3JUE9_9GAMM     VGPFNLLDEVGIDVGTKI..LPILVNRFGDR..FKAPDLLDKVNKDDRKGKKNGKGFY..
tr|A0A7D4P594|A0A7D4P594_YERMW     VGPITLLDEVGIDVGTKI..MPILVEQLGPR..FAAPASFDLLLQDGRKGRKNGRGFY..
tr|A0A7U7IVK6|A0A7U7IVK6_YEREN     VGPITLLDEVGIDVGTKI..IPILVEKLGAR..FAAPPSFDVILEDGRKGRKNGRGFY..
tr|A0A447RIF5|A0A447RIF5_YEREN     VGPITLLDEVGIDVGTKI..IPILVEKLGAR..FAAPPSFDVILEDGRKGRKNGRGFY..
tr|A0A7H4ZLV9|A0A7H4ZLV9_YERP4     VGPITLLDEVGIDVGTKI..IPILVEKLGAR..FAAPPSFDVILEDGRKGRKNGRGFY..
tr|A0A0H3NX46|A0A0H3NX46_YERE1     VGPITLLDEVGIDVGTKI..IPILVEKLGAR..FAAPPSFDVILEDGRKGRKNGRGFY..
tr|A0A7T9XUV7|A0A7T9XUV7_YEREN     VGPITLLDEVGIDVGTKI..IPILVEKLGAR..FAAPPSFDVILEDGRKGRKNGRGFY..
tr|A0A7U7IYV8|A0A7U7IYV8_YEREN     VGPITLLDEVGIDVGTQI..IPILVEKLGAR..FAAPPSFDVILEDGRKGRKNGRGFY..
tr|A0A0H5G7G2|A0A0H5G7G2_YEREN     VGPITLLDEVGIDVGTKI..IPILVEKLGAR..FAAPPSFDVILEDGRKGRKNGRGFY..
tr|A0A2A7TDI2|A0A2A7TDI2_YERKR     VGPITLLDEVGIDVGTKI..MPILVEKLGAR..FAAPPSFDVILKDGRKGRKNGRGFY..
tr|A0A0T9M261|A0A0T9M261_YERKR     VGPIALLDEVGIDVGTKI..MPILVEKLGAR..FAAPPSFDVILKDGRKGRKNGRGFY..
tr|A0A0A0CMP1|A0A0A0CMP1_PHOLU     VGPINLLDEVGIDVGTKI..MPILVEQLGPR..FAAPESLDAVLKDGRKGRKNGRGFY..
tr|A0A329VG55|A0A329VG55_9GAMM     VGPINLLDEVGIDVGTKI..MPILVEQLGPR..FAAPESLDAVLKDGRKGRKNGRGFY..
sp|Q7N288|FADJ_PHOLL               VGPINLLDEVGIDVGTKI..MPVLVEQLGPR..FAAPESLDAVLKDGRKGRKNGRGFY..
tr|A0A6L9JMX5|A0A6L9JMX5_PHOLM     VGPINLLDEVGIDVGTKI..MPVLVEQLGPR..FAAPESLDAVLKDGRKGRKNGRGFY..
tr|A0A7X5HQ29|A0A7X5HQ29_PHOLM     VGPINLLDEVGIDVGTKI..MPVLVEQLGPR..FAAPESLDAVLKDGRKGRKNGRGFY..
tr|A0A1C0U051|A0A1C0U051_9GAMM     VGPINLLDEVGIDVGTKI..MPILVEQLGPR..FAAPESLNVVLKDGRKGRKNGRGFY..
tr|A0A7X5QF62|A0A7X5QF62_9GAMM     VGPINLLDEVGIDVGTKI..MPILVEQLGAR..FAAPESLNAVLKDGRKGRKNGRGFY..
```

```
tr|A0A2D0KWM9|A0A2D0KWM9_9GAMM   IGPINLLDEVGIDVGTKI..IPVLVDQLGDR..FTPPAILDAVLSDDRKGKKNGRGFY..
tr|A0A2D0LAI1|A0A2D0LAI1_9GAMM   IGPINLLDEVGIDVGTKI..IPVLVDQLGDR..FTPPAILDAVLSDDRKGKKNGRGFY..
tr|A0A2D0IXC8|A0A2D0IXC8_XENBU   IGPINLLDEVGIDVGTKI..IPVLIEQLGSR..FTPSAILDAVLNDNRKGKKNGRGFY..
tr|W1JAM5|W1JAM5_9GAMM           IGPINLLDEVGIDVGTKI..IPVLVEQLGRR..FTPPAILDAVLNDNRKGKKNGRGFY..
tr|A0A3D9UED3|A0A3D9UED3_9GAMM   IGPINLLDEVGIDVGTKI..IPVLVEQLGRR..FTPPAILDAVLNDNRKGKKNGRGFY..
tr|A0A1I3JCA9|A0A1I3JCA9_9GAMM   IGPINLLDEVGIDVGTKI..IPILVEQLGDR..FVAPEILETILKDDRKGKKNGRGFY..
tr|A0A068QUL9|A0A068QUL9_9GAMM   IGPINLLDEVGIDVGTKI..IPILVEQLGSR..FAAPEMLAAILQDDRKGKKNGRGFY..
tr|A0A0M0TCH6|A0A0M0TCH6_9GAMM   IGPINLLDEVGIDVGTKI..IPVLVEELGSR..FAAPEILAAILQDDRKGRKNGRGFY..
tr|A0A1Q5U854|A0A1Q5U854_9GAMM   IGPINLLDEVGIDVGTKI..IPVLVEELGSR..FEAPEILDAILKDDRKGKKNGRGFY..
tr|A0A2D0ISP1|A0A2D0ISP1_9GAMM   IGPINLLDEVGIDVGTKI..IPVLVEELGSR..FEAPEILDAILRDDRKGKKNGRGFY..
tr|A0A1Q5TUI8|A0A1Q5TUI8_9GAMM   IGPINLLDEVGIDVGTKI..IPVLVEDLGSR..FAAPEILAAILQDDRKGKKNGRGFYLY
tr|A0A2D0KJI7|A0A2D0KJI7_9GAMM   IGPINLLDEVGIDVGTKI..IPVLVEELGSR..FEAPEILAAILKDDRKGKKNGRGFY..
tr|A0A1Y2SB97|A0A1Y2SB97_9GAMM   IGPINLLDEVGIDVGTKI..IPILVEQLGSR..FVAPEILDAILKDDRKGKKNGRGFY..
tr|A0A1I5DWI7|A0A1I5DWI7_9GAMM   IGPINLLDEVGIDVGTKI..IPILVEQLGSR..FTAPEILEAILKDDRKGKKNGRGFY..
tr|A0A1I7GWY6|A0A1I7GWY6_9GAMM   IGPINLLDEVGIDVGTKI..IPILVGQLGNR..FAAPEILEVILKNNRKGKKNGRGFY..
tr|D3VKY8|D3VKY8_XENNA           IGPINLLDEVGIDVGTKI..IPILVEQLGNR..FTAPDILAAILKDNRKGKKNRRGFY..
tr|A0A2G0Q6Q9|A0A2G0Q6Q9_9GAMM   IGPINLLDEVGIDVGTKI..IPILVEQLGNR..FAAPEILDAILKDDRKGKKNGRGFY..
tr|A0A2D0JU24|A0A2D0JU24_9GAMM   IGPINLLDEVGIDVGTKI..IPILVEQLGSR..FAAPEILEAVLKDNRKGKKNGRGFY..
tr|A0A0J5FN38|A0A0J5FN38_9GAMM   IGPINLLDEVGIDVGTKI..IPILVEQLGNR..FAAPEILEAVLKDNRKGKKNSRGFY..
tr|A0A432XLD4|A0A432XLD4_9GAMM   VGPMKLMDEVGIDVAAKV..APIMADELGER..FRAPDAFGKLLDDERKGKKNQKGFY..
tr|A0A2D8HU84|A0A2D8HU84_9GAMM   VGPITLLDEVGIDVAAKV..APVLVKELGDR..FEAPDAFDKLIDDDRKGKKNQKGFY..
tr|A0A656X1Q2|A0A656X1Q2_9GAMM   VGPITLLDEVGIDVAAKV..APVLVKELGDR..FEAPDAFEKLIDDDRKGKKNQKGFY..
tr|A0A4Q1QH79|A0A4Q1QH79_9GAMM   VGPMTLLDEVGIDVAAKV..APILVNELGDR..FEAPQAFEKLIDDDRKGKKNQKGFY..
tr|A0A1G7LQE6|A0A1G7LQE6_9GAMM   VGPMTLLDEVGIDVAAKV..APILVNELGDR..FEAPQAFEKLIDDDRKGKKNQKGFY..
tr|A0A432YVG7|A0A432YVG7_9GAMM   VGPMTLLDEVGIDVAAKV..APILVNELGDR..FEAPQAFEKLIDDDRKGKKNQKGFY..
tr|A0A1J5N0P5|A0A1J5N0P5_9GAMM   VGPVTLLDEVGIDVGSKI..SPILAEAELGDR..FKAPTAFDALLNDDRKGKKNRKGFY..
tr|A0A0D8D548|A0A0D8D548_9GAMM   VGPMQLLDEVGIDIGAKI..GPILQAELGDR..FATPPAFDKLLADGRLGKKASKGFY..
tr|A0A3E0TNM2|A0A3E0TNM2_9GAMM   VGPMQLLDEVGIDIGAKI..SPILTAELGER..FTAPEAFDSLINDGRLGKKSNKGFY..
tr|A0A3E0UD67|A0A3E0UD67_9GAMM   VGPMQLLDEVGIDIGAKI..SPILTAELGER..FTAPEAFDRLINDGRLGKKSNKGFY..
tr|A0A3E0U0P4|A0A3E0U0P4_9GAMM   VGPMQLLDEVGIDIGAKI..SPILTAELGER..FTAPEAFDRLINDGRLGKKSNKGFY..
tr|A0A0M2V8B1|A0A0M2V8B1_9GAMM   VGPVTLLDEVGIDVGAKI..SPILTEQLGER..FAAPAAFDKLLADGRKGKKSEKGFY..
tr|A0A285ITY5|A0A285ITY5_9GAMM   VGPMTLLDEVGIDVGAKI..SPILTEQLGER..FAPPPAFDKLLADGRKGKKSAKGFY..
tr|A0A486XTB9|A0A486XTB9_9GAMM   VGPVTLLDEVGIDVGAKI..SPILTKELGER..FAAPAFEKLLADGRKGKKTEKGFY..
tr|I1E1G1|I1E1G1_9GAMM           VGPVTLLDEVGIDVGAKI..SPILTQELGER..FSAPAAFDKLLADGRKGKKSEKGFY..
tr|A0A1H6KJ68|A0A1H6KJ68_9GAMM   VGPVTLLDEVGIDVGAKI..SPILTTELGER..FAAPAAFDKLLADGRKGKKTEKGFY..
tr|A0A0X3Y764|A0A0X3Y764_9GAMM   VGPVTLLDEVGIDVGAKI..SPILTTELGER..FAAPAAFDKLLADGRKGKKTEKGFY..
tr|A0A2N1YEV4|A0A2N1YEV4_9GAMM   VGPVTLLDEVGIDVGAKI..SPILTTELGER..FAAPAAFDKLLADGRKGKKTEKGFY..
tr|A0A3P3QCM3|A0A3P3QCM3_9GAMM   VGPITLLDEVGIDVGAKI..SPILLNELGER..FAAPAAFDRLLADGRKGRKTEKGFY..
tr|F7NT72|F7NT72_9GAMM           VGPITLLDEVGIDVGAKI..SPILLKELGER..FAAPAAFDRLLADGRKGRKAEKGFY..
tr|A0A3S2TW63|A0A3S2TW63_9GAMM   VGPITLLDEVGIDVGAKI..SPILLNELGER..FTAPAAFDRLLADGRKGRKTEKGFY..
tr|A0A3D5BGW7|A0A3D5BGW7_9GAMM   VGPITLLDEVGIDVGAKI..SPILERELGER..FKAPAAFDKLIADGRKGKKVEKGFY..
tr|A0A5C7TI17|A0A5C7TI17_9GAMM   VGPITLLDEVGIDVGAKI..SPILQKELGER..FQAPAAFDKLIADGRKGKKVEKGFY..
tr|A0A0U4W982|A0A0U4W982_9GAMM   VGPITLLDEVGIDVGAKI..SPILQKELGER..FQAPAAFDKLIADGRKGKKVEKGFY..
tr|A0A2I0FCM2|A0A2I0FCM2_9GAMM   VGPVTLLDEVGIDVGAKI..GPILTKELGSR..FEAPAAFDKLLADDRKGRKNEKGFY..
tr|A0A2G2IVL1|A0A2G2IVL1_9GAMM   VGPVTLLDEVGIDVGAKI..GPILTKELGSR..FEAPAAFDKLLADDRKGRKNEKGFY..
tr|A6FI21|A6FI21_9GAMM           VGPVTLLDEVGIDVGAKI..GPILTKELGSR..FEAPAAFDKLLADDRKGRKNEKGFY..
tr|A0A4U1BNR2|A0A4U1BNR2_9GAMM   VGPITLLDEVGIDVGAKI..SPILEAELGSR..FAAPAAFDKLLADGRMGRKNGKGFY..
tr|E1SQ60|E1SQ60_FERBD           VGPVTLLDEVGIDVGAKI..SPILESELGSR..FAAPAAFAKLLADDRKGRKNGKGFY..
tr|A0A4Y6IZ09|A0A4Y6IZ09_9GAMM   VGPMTLLDEVGIDVGAKI..SPILEKELGER..FKAPAAFDKLLADDRKGRKNGKGFY..
tr|A0A0C3QSZ7|A0A0C3QSZ7_9GAMM   VGPMTLLDEVGIDVGAKI..SPILEQELGER..FKAPAAFDKLLADERKGRKNGKGFY..
tr|A0A6L7HXW0|A0A6L7HXW0_9GAMM   VGPMTLLDEVGIDVGAKI..SPILEQELGER..FKAPAAFDKLLADDRKGRKNGKGFY..
sp|A3QFP3|FADJ_SHELP             VGPMTLLDEVGIDVGAKI..SPILEKELGER..FKAPAAFDKLLADDRKGRKNGKGFY..
tr|A0A1E5IXH7|A0A1E5IXH7_SHECO   VGPMTLLDEVGIDVGAKI..SPILEKELGAR..FQAPTAFDKLLGDDRKGRKNGKGFY..
tr|A0A411PKQ0|A0A411PKQ0_9GAMM   VGPMTLLDEVGIDVGAKI..SPILEKELGER..FSAPQAFDQLLADDRKGRKNGKGFY..
tr|A0A6G9QKM3|A0A6G9QKM3_9GAMM   VGPMTLLDEVGIDVGAKI..SPILEKELGAR..FSAPAAFDKLLADDRKGRKNGKGFY..
tr|A0A6P1UL63|A0A6P1UL63_9GAMM   VGPMTLLDEVGIDVGAKI..SPILEKELGDR..FKAPAAFDKLLADDRKGRKNGKGFY..
tr|A0A2N1ERI9|A0A2N1ERI9_9GAMM   VGPMTLLDEVGIDVGAKI..SPILEKELGDR..FKAPAAFDKLLADDRKGRKNGKGFY..
tr|A0A7W4FU55|A0A7W4FU55_9GAMM   VGPMTLLDEVGIDVGAKI..SPILEKELGDR..FKAPAAFDKLLADDRKGRKNGKGFY..
sp|Q8ECP7|FADJ_SHEON             VGPITLLDEVGIDVGAKI..SPILEKELGER..FKAPAAFDKLLSDDRKGRKNGKGFY..
tr|A0A501XZY8|A0A501XZY8_9GAMM   VGPITLLDEVGIDVGAKI..APILEKELGER..FKAPTAFDKLLNDDRKGRKNGKGFY..
tr|A0A2W5DCZ0|A0A2W5DCZ0_SHEOE   VGPITLLDEVGIDVGAKI..SPILEKELGER..FKAPAAFDKLLSDDRKGRKNGKGFY..
tr|A0A1E3V3C8|A0A1E3V3C8_9GAMM   VGPITLLDEVGIDVGAKI..SPILEKELGER..FKAPAAFDKLLSDDRKGRKNGKGFY..
tr|A0A1Z4AI20|A0A1Z4AI20_9GAMM   VGPITLLDEVGIDVGAKI..APILEKELGER..FKAPTAFDKLLSDDRKGRKNGKGFY..
tr|A0A7X9LJL9|A0A7X9LJL9_9GAMM   VGPITLLDEVGIDVGAKI..APILEKELGER..FKAPAAFDKLLSDDRKGRKNGKGFY..
tr|A0A073KMY5|A0A073KMY5_9GAMM   VGPITLLDEVGIDVGAKI..APILEKELGER..FKAPAAFDKLLSDDRKGRKNGKGFY..
sp|A0KV76|FADJ_SHESA             VGPITLLDEVGIDVGAKI..APILEKELGER..FKAPAAFDKLLSDDRKGRKNGKGFY..
tr|A0A220UTH7|A0A220UTH7_9GAMM   VGPITLLDEVGIDVGAKI..APILEKELGER..FKAPAAFDKLLSDDRKGRKNGKGFY..
tr|A0A5B8R6W1|A0A5B8R6W1_9GAMM   VGPITLLDEVGIDVGAKI..APILEKELGER..FKAPAAFDKLLSDDRKGRKNGKGFY..
tr|V1DAI4|V1DAI4_9GAMM           VGPITLLDEVGIDVGAKI..APILEKELGER..FKAPAAFDKLLSDDRKGRKNGKGFY..
tr|A0A448CPQ4|A0A448CPQ4_SHEPU   VGPITLLDEVGIDVGAKI..APILEKELGER..FKAPAAFDKLLSDDRKGRKNGKGFY..
tr|A0A252ERQ3|A0A252ERQ3_SHEPU   VGPITLLDEVGIDVGAKI..APILEKELGER..FKAPAAFDKLLSDDRKGRKNGKGFY..
sp|Q0HKD1|FADJ_SHESM             VGPITLLDEVGIDVGAKI..APILEKELGER..FKAPAAFDKLLSDDRKGRKNGKGFY..
sp|Q0HWN3|FADJ_SHESR             VGPITLLDEVGIDVGAKI..APILETELGER..FKAPAAFDKLLSDDRKGRKNGKGFY..
tr|F7RQE3|F7RQE3_9GAMM           VGPITLLDEVGIDVGAKI..SPILEKELGER..FKAPAAFDKLLGDDRKGRKNGKGFY..
tr|B8EE98|B8EE98_SHEB2           VGPITLLDEVGIDVGAKI..SPILEKELGER..FKAPAAFDKLLSDDRKGRKNGKGFY..
sp|A6WQ25|FADJ_SHEB8             VGPITLLDEVGIDVGAKI..SPILEKELGER..FKAPAAFDKLLSDDRKGRKNGKGFY..
tr|A0A448EK41|A0A448EK41_9GAMM   VGPITLLDEVGIDVGAKI..SPILEKELGER..FKAPAAFDKLLGDDRKGRKNGKGFY..
tr|A0A553JHX1|A0A553JHX1_SHEHA   VGPMTLLDEVGIDVGAKI..SPILERELGDR..FKAPAAFDKLLSDDRKGRKNNKGFY..
tr|B8CPY6|B8CPY6_SHEPW           VGPMTLLDEVGIDVGAKI..SPTLEAELGER..FKAPSAFDKLLADDRKGRKNGKGFY..
tr|A0A431WFC4|A0A431WFC4_9GAMM   VGPMTLLDEVGIDVGAKI..SPILESELGER..FKAPAAFDKLLADDRKGRKNGKGFY..
tr|A8FTR7|A8FTR7_SHESH           VGPMTLLDEVGIDVGAKI..SPILESELGER..FKAPAAFDKLLADDRKGRKNGKGFY..
tr|A0A431WNL0|A0A431WNL0_9GAMM   VGPMTLLDEVGIDVGAKI..SPILESELGER..FKAPAAFDKLLADDRKGRKNGKGFY..
tr|A0A550AEC5|A0A550AEC5_9GAMM   VGPMTLLDEVGIDVGAKI..SPILEAELGDR..FKAPEAFDKLLKDDRKGRKNGKGFY..
tr|A0A7L4WW90|A0A7L4WW90_9GAMM   VGPMTLLDEVGIDVGAKI..SPILEAELGDR..FKAPEAFDKLLKDDRKGRKNGKGFY..
tr|A9DDU3|A9DDU3_9GAMM           VGPMTLLDEVGIDVGAKI..SPILAAELGER..FKAPAAFDKLLGDDRKGRKNGKGFY..
tr|A0A330M2B9|A0A330M2B9_9GAMM   VGPMTLLDEVGIDVGAKI..SPILAAELGER..FKAPAAFDKLLGDDRKGRKNGKGFY..
tr|A0A5N8UFC7|A0A5N8UFC7_9GAMM   VGPMTLLDEVGIDVGAKI..SPILEAELGER..FKAPAAFDKLLSDDRKGRKNGKGFY..
tr|A0A1S6HN57|A0A1S6HN57_9GAMM   VGPMTLLDEVGIDVGAKI..SPILEAELGER..FKAPAAFDKLLRDDRKGRKNGKGFY..
tr|D4ZMH7|D4ZMH7_SHEVD           VGPMTLLDEVGIDVGAKI..SPILEAELGER..FKAPAAFDKLLSDDRKGRKNGKGFY..
tr|A0A3L8Q213|A0A3L8Q213_9GAMM   VGPINLLDEVGIDVGAKI..SPILEKELGSR..FKAPDVYEKLLADGRKGRKSEKGFY..
tr|A0A3A6U4N9|A0A3A6U4N9_9GAMM   VGPMTLLDEVGIDVGAKI..SPILEKELGER..FVAPQAFDKLLGDGRKGRKSDKGFY..
tr|A0A4Q5MA37|A0A4Q5MA37_9GAMM   VGPMTLLDEVGIDVGAKI..SPILEKELGER..FEAPKAFDKLLNDGRKGRKSEKGFY..
tr|A0A1L6LSX5|A0A1L6LSX5_9DELT   VGPVTLLDEVGIDVAAHV..GPIMVAAFGDR..MIPPPTMAKLVADDRKGRKNKRGFY..
tr|A0A2W4L9B9|A0A2W4L9B9_9PROT   VGPFTLLDEVGIDVGASV..TRTMVEAFGER..MLPPDAFERVVADGRKGRKNKKGFY..
tr|A0A2W4M4E6|A0A2W4M4E6_9PROT   VGPLALLDEVGIDVASKV..GKILHSAFGAR..MAPPPAVEQLLADERRGRKNERGFY..
tr|A0A6I2GRX9|A0A6I2GRX9_9DELT   VGPITLLDEVGIDVAHKV..GPIMQAAFGER..LAAPSGLDAVVKDGRLGRKNGRGFY..
tr|A0A0H4WMK2|A0A0H4WMK2_9DELT   VGPITLLDEVGIDVAQKV..GPIMEAAFGKR..MAAPKALEKVVADGRLGRKSEKGFY..
tr|F8CJ36|F8CJ36_MYXFH           VGPMTLLDEVGIDVAQKV..GPIMEAAFGKR..MAAPKALEKVVADGRLGRKTEKGFY..
tr|A0A250K0F1|A0A250K0F1_9DELT   VGPMTLLDEVGIDVAQKV..GPIMEAAFGKR..MAAPKALEKVVADGRLGRKTEKGFY..
tr|A0A7Y6WFZ2|A0A7Y6WFZ2_9DELT   VGPITLLDEVGIDVAQKV..GPIMEAAFGKR..MAAPKALEKVVADGRLGRKTQKGFY..
tr|A0A7Y7C660|A0A7Y7C660_9DELT   VGPITLLDEVGIDVAQKV..GPIMEAAFGKR..MAAPKALEKVVADGRLGRKTQKGFY..
tr|A0A7Y4JFH2|A0A7Y4JFH2_MYXXA   VGPITLLDEVGIDVAQKV..GPLMEAAFGKR..MAAPKALEKVVADGRLGRKTQKGFY..
tr|A0A4Y6CZQ9|A0A4Y6CZQ9_MYXXA   VGPITLLDEVGIDVAQKV..GPLMEAAFGKR..MAAPKALEKVVADGRLGRKTQKGFY..
```

```
tr|A0A7Y4IKV5|A0A7Y4IKV5_MYXXA  VGPITLLDEVGIDVAQKV..GPIMEAAFGKR..MAAPKALEKVVADGRLGRKTQKGFY..
tr|A0A511HHB0|A0A511HHB0_9DELT  VGPITLLDEVGIDVAQKV..GPIMEAAFGKR..MAAPKALEKVVADGRLGRKTQKGFY..
tr|A0A4Y6CKY7|A0A4Y6CKY7_MYXXA  VGPITLLDEVGIDVAQKV..GPIMEAAFGKR..MAAPKALEKVVTDGRLGRKTQKGFY..
tr|Q1D1F2|Q1D1F2_MYXXD          VGPITLLDEVGIDVAQKV..GPIMEAAFGKR..MAAPKALEKVVADGRLGRKTQKGFY..
tr|A0A7Y4MA14|A0A7Y4MA14_MYXXA  VGPITLLDEVGIDVAQKV..GPIMEAAFGKR..MAAPKALEKVVADGRLGRKTQKGFY..
tr|A0A7T8Y4N9|A0A7T8Y4N9_MYXXA  VGPITLLDEVGIDVAQKV..GPIMEAAFGKR..MAAPKALEKVVADGRLGRKTQKGFY..
tr|L7UE67|L7UE67_MYXSD          VGPMTLLDEVGIDVAHKV..GPLMEAAFGKR..MAAPKALDAVVAEGRLGRKSQKGFY..
tr|A0A511T9X1|A0A511T9X1_MYXFU  VGPMTLLDEVGIDVAHKV..SPMMEAAFGKR..MVAPKALDGVVADGRLGRKSQKGFY..
tr|A0A7Y7C9C4|A0A7Y7C9C4_9DELT  VGPMTLLDEVGIDVAHKV..GPIMEAAFGKR..MAAPKSLDGVVADGRLGRKSQKGFY..
tr|A0A540X7W8|A0A540X7W8_9DELT  VGPMTLLDEVGIDVAHKV..GPIMEAAFGKR..MSAPKSLDGVVADGRLGRKSQKGFY..
tr|A0A3A5FK19|A0A3A5FK19_9DELT  VGPITLLDEVGIDVAHKV..GPIMEGAFGKR..MAAPKALDGVVADGRLGRKTQKGFY..
tr|A0A3A8JQL9|A0A3A8JQL9_9DELT  VGPITLLDEVGIDVAQKV..GPIMEAAFGKR..MSAPKALEGVVSDGRLGRKTNKGFY..
tr|A0A7Y4NFA4|A0A7Y4NFA4_9DELT  VGPITLLDEVGIDVAQKV..GPIMEAAFGKR..MAAPKALEGVVSDGRLGRKTNKGFY..
tr|A0A3A8HDQ7|A0A3A8HDQ7_9DELT  VGPITLLDEVGIDVAQKV..GPIMEAAFGKR..MAAPKALEGVVSDGRLGRKTNKGFY..
tr|A0A3A8GR90|A0A3A8GR90_9DELT  VGPITLLDEVGIDVAQKV..GPIMEAAFGKR..MAAPKALEGVVSDGRLGRKTNKGFY..
tr|A0A3A8SBD7|A0A3A8SBD7_9DELT  VGPITLLDEVGIDVAQKV..GPIMEAAFGKR..MVAPKALAGVVSDGRLGRKTNKGFY..
tr|A0A3A8T0I8|A0A3A8T0I8_9DELT  VGPITLLDEVGIDVAQKV..GPIMEAAFGKR..MVAPKALAGVVSDGRLGRKTNKGFY..
tr|A0A7X5BU07|A0A7X5BU07_9DELT  VGPITLLDEVGIDVAQKV..GPIMEAAFGKR..MVAPKALAGVVSDGRLGRKTNKGFY..
tr|A0A7Y1RVL2|A0A7Y1RVL2_9DELT  VGPITLLDEVGIDVAQKV..GPIMEAAFGKR..MVAPKALAGVVSDGRLGRKTNKGFY..
tr|A0A3A8THN2|A0A3A8THN2_9DELT  VGPITLLDEVGIDVAQKV..GPIMEAAFGKR..MVAPKALAGVVSDGRLGRKTNKGFY..
tr|A0A7Y1RX76|A0A7Y1RX76_9DELT  VGPITLLDEVGIDVAQKV..GPIMEAAFGKR..MVAPKALAGVVSDGRLGRKTNKGFY..
tr|A0A3A8RA58|A0A3A8RA58_9DELT  VGPITLLDEVGIDVAQKV..GPIMEAAFGKR..MAAPKALEGVVSDGRLGRKTNKGFY..
tr|A0A410RPB6|A0A410RPB6_CORCK  VGPITLLDEVGIDVAQKV..GPIMEAAFGKR..MAAPKALEGVVSDGRLGRKTNKGFY..
tr|A0A7Y4J474|A0A7Y4J474_CORCK  VGPITLLDEVGIDVAQKV..GPIMEAAFGKR..MAAPKALEGVVSDGRLGRKTNKGFY..
tr|A0A3A8I9Z6|A0A3A8I9Z6_9DELT  VGPITLLDEVGIDVAQKV..GPIMEAAFGKR..MAAPKALEGVVSDGRLGRKTNKGFY..
tr|H8MKE9|H8MKE9_CORCM          VGPITLLDEVGIDVAQKV..GPIMEAAFGKR..MAAPKALEGVVSDGRLGRKTNKGFY..
tr|A0A3A8H102|A0A3A8H102_9DELT  VGPITLLDEVGIDVAQKV..GPIMEAAFGKR..MAAPKALEGVVSDGRLGRKTNKGFY..
tr|A0A554FW33|A0A554FW33_9DELT  VGPITLLDEVGIDVAQKV..GPVMEAAFGKR..MAAPKALEGVVSDGRLGRKTQKGFY..
tr|A0A3A8NPC0|A0A3A8NPC0_9DELT  VGPITLLDEVGIDVAQKV..GPIMEAAFGKR..MSAPKALEGVVSDGRLGRKTQKGFY..
tr|A0A3A8JUV3|A0A3A8JUV3_9DELT  VGPITLLDEVGIDVAQKV..GPIMEAAFGKR..MSAPKALEGVVSDGRLGRKTQKGFY..
tr|A0A3A8NEX6|A0A3A8NEX6_9DELT  VGPITLLDEVGIDVAQKV..GPIMEAAFGKR..MAAPKALEGVVSEGRLGRKTQKGFY..
tr|A0A3A8JEU8|A0A3A8JEU8_9DELT  VGPITLLDEVGIDVAQKV..GPIMEAAFGKR..MAAPKALDGVISDGRLGRKTQKGFY..
tr|A0A3A8LI76|A0A3A8LI76_9DELT  VGPITLLDEVGIDVAQKV..GPIMEAAFGKR..MAAPKALEGVISDGRLGRKTQKGFY..
tr|A0A085WXN8|A0A085WXN8_9DELT  VGPITLLDEVGIDVAYKV..GPIMEAAFGKR..LAAPKTLDKVIQDGRLGRKNQKGFY..
tr|A0A2T4V0M5|A0A2T4V0M5_9DELT  VGPITLLDEVGIDVAQKV..GPIMEAAFGKR..MSAPRALEKVVADGRLGRKNKKGFY..
tr|A0A0G2ZSW5|A0A0G2ZSW5_9DELT  VGPITLLDEVGIDVAQKV..GPIMETAFGKR..MAAPRALEKVVADGRLGRKNKKGFY..
tr|A0A3M2DKY0|A0A3M2DKY0_9DELT  VGPIALLDEVGIDVAHKV..GQIAADAFGDR..MPPMQGLDALIADGRHGRKNGKGFY..
tr|A0A661NQ58|A0A661NQ58_9DELT  VGPIKLTDEVGIDVGAKV..GKILLAEFGER..MQAPAGIDKLVQDDRKGRKNGRGFY..
tr|A0A520YD99|A0A520YD99_9DELT  VGPIKLTDEVGIDVGAKV..GKIMLEAFGER..MAAPEGMKKLIDDERFGRKNGRGFY..
tr|A0A7Y3BRE4|A0A7Y3BRE4_9DELT  VGPIKLTDEVGIDVGAKV..GKIMLEAFGER..MAAPEGMKKLIDDERFGRKNGRGFY..
tr|A0A2D9TF90|A0A2D9TF90_9DELT  VGPMKLTDEVGIDVGAKV..GKIMVQAFGDR..MVPPAGMDKLVADDRKGRKNGRGFY..
tr|A0A2E0TP32|A0A2E0TP32_9DELT  VGPIKLTDEVGIDVGAKV..GKVMQKAFGDR..MTPPAGMEKLVADDRKGRKNGRGFY..
tr|A0A2E4Y3V1|A0A2E4Y3V1_9PROT  VGPITLCDEVGLDVAMHI..AKDLKKALGERICSAPSELIDDLVKNNMNGRKSGKGFF..
tr|A0A2E6VRH4|A0A2E6VRH4_9DELT  VGPVTLLDEVGLDVGAHV..SKDLGAAFGERVVSADPRAVQALVETGAHGRKSQKGFF..
tr|A0A1F9FB59|A0A1F9FB59_9DELT  VGPITLLDEVGIDVAAHV..SKDMAPFFEPRFGPRDRSALEAMVKEGFTGRKGGKGFF..
```

```
ECHA_HUMAN                                                            TT
                             640                   650       660
ECHA_HUMAN                          ....IYQ..E...GVK........RK.......DLNSDMDSILASLKL....PP.KSEV.
tr|A0A4R6XLA8|A0A4R6XLA8_9GAMM      ....VYP.................KK.......GRKTVNQEIYKYFG.....GD.DRKD.
tr|A0A6A0IJT2|A0A6A0IJT2_9BACT      ....LYP.....KDKG........RH.......GKKPLNPEMLPLIG.....GP.DRRP.
tr|A0A7V3QZU7|A0A7V3QZU7_9BACT      ....LYP......PRG........KR.......GKKRPNAEVYGLLG.....GV.PRRP.
tr|A0A7V4SYJ6|A0A7V4SYJ6_9BACT      ....LYG......RR.........RR.......GAKPVNEAVYALLG.....GA.PRRE.
tr|A0A2A8D2N6|A0A2A8D2N6_9BACT      ....FYE..P...QPGK......PEK.......KRTKFNDAVYRHFG.....GK.NRTS.
tr|A0A2N1TGP0|A0A2N1TGP0_9SPIR      ....RYD......VPS........KK.......GKRPVNTEVYALFS.....GS.PRKN.
tr|A0A1G3QGW4|A0A1G3QGW4_9SPIR      ....RYD.............LPRKK........GEKPVNSEVYSLFSD....VP..RKK.
tr|A0A1G3QTY3|A0A1G3QTY3_9SPIR      ....RYD.LP..K.KK.................GKKPVNTAVYALFG.....DA.QRKK.
tr|A0A7X9L359|A0A7X9L359_9DELT      ....LYE.EP....GRKGLLALKRKK.......GGKPVNTEVYEILG.....GA..RRV.
tr|A0A522CJY0|A0A522CJY0_9SPIR      ....RYDVPK....KK.................GVRASDPDIYRLVGN....AP..RKK.
tr|A0A2N1RRJ3|A0A2N1RRJ3_9SPIR      ....RYD.............VKKKK........GVKIPDERVYAILG.....GA.PRKQ.
tr|A0A2N2KFN7|A0A2N2KFN7_9DELT      ....RYD.............GKKKK........GQKIPNAEVYALIG.....GA.PRRK.
tr|A0A1V6AXX9|A0A1V6AXX9_9DELT      ....RYDGKK....KK.................GKKIPNEEVYALLGA....AP..RKK.
tr|A0A2N2HZL0|A0A2N2HZL0_9DELT      ....LYD..A...KKKK................GQKLPNEEVYALLG.....GA.PRKK.
tr|A0A5E8ARP3|A0A5E8ARP3_9BACT      ....QYD.....GKTG........KK.......VRGKVNEEALRFIE.....SR.GSKA.
tr|A0A662A7B1|A0A662A7B1_9BACT      ....IYD..E....KTG.......KK.......VRGKINPDIKEFFPT.......KSNN.
tr|D7CV16|D7CV16_TRURR              ....DYS......SG.........KK.......GEKAVNEEVYRFFS.....AP..RKE.
tr|A0A3C2AKX4|A0A3C2AKX4_9FLAO      ....KYD......EN.........GK.......NMRELDDNVYSFFG.....GN.NRKK.
tr|A0A3B8ZLN3|A0A3B8ZLN3_9PLAN      ....QYD..S....KG........KK.......IRDKLDDNVYNYFG.....GS.QRKQ.
tr|A0A345UGV8|A0A345UGV8_9BACT      ....RYG.......EG.......KG........KKKEINPDIYAHFG.....GT.SRKK.
tr|A0A6C1P6A2|A0A6C1P6A2_9BACT      ....TYS.....GKGG.................KKKEVNQDVYSFFG.....GP.KRRP.
tr|A0A3M1X9W8|A0A3M1X9W8_9BACT      ....LYK................GKK.......GRREVNPAVYRLFG.....GR.KRKR.
tr|A0A354C796|A0A354C796_9DELT      ....RYG..D...TAGK................KKKEVNKEIYTFFG.....GP.GRKK.
tr|A0A3M1NUM8|A0A3M1NUM8_9BACT      ....LYD......QK.........GR.......KQKEVDQTVYRYFG.....GT.QRKK.
tr|A0A3M2KRC8|A0A3M2KRC8_9BACT      ....HYP......AGSPKLPVPGSK.......RKEVNQTVYKFFG.....GV.KKRR.
tr|A0A5S9IHR0|A0A5S9IHR0_9BACT      ....LYP.....KKKG.................KKKTINTDIYRFFG.....GN.SRKT.
tr|A0A6M1ST81|A0A6M1ST81_9BACT      ....NYE.....QQNS........KK.......KKKDPNEEIYKYFG.....GS.GRKS.
tr|A0A521AAE5|A0A521AAE5_9BACT      ....HYE.EE....DN........QT.......KKKDPNNEIYRFFG.....GP.KRTS.
tr|A0A1M4ZMD6|A0A1M4ZMD6_9BACT      ....HYEEEG....S.........KT.......KKKEPNKKIYQFFG.....GP.DRSS.
tr|A0A2A2GCS2|A0A2A2GCS2_9BACT      ....KYE.....QKNG........KM.......KKTDPNKEIYQYFG.....GS.DRKS.
tr|A0A5D3YIH7|A0A5D3YIH7_9BACT      ....TYE.EK....DG........DL.......KKGDPNKQMYQYFG.....GP.NRTS.
tr|A0A6M1T9G0|A0A6M1T9G0_9BACT      ....RYK.....QENG........KT.......KKDEPNEEIYEFFG.....GS.GRTS.
tr|A0A6A8Q1Y8|A0A6A8Q1Y8_9BACT      ....KYA.......DS.................KKKEVNTDIYAHFG.....GS.ERKN.
tr|A0A521BVJ3|A0A521BVJ3_9BACT      ....RYF.......DD.................KDKEVNTDIYKHFG.....GS.NRTN.
tr|A0A359E0Y8|A0A359E0Y8_9BACT      ....KYS.......SG.................KKKEVNTEIYSYFG.....GS.NRTN.
tr|A0A2D8CIM6|A0A2D8CIM6_9BACT      ....KYS.......SG.................KKKEVNTEIYSYFG.....GS.NRTN.
tr|A0A2D9FWF5|A0A2D9FWF5_9BACT      ....LYS.......ED.................DDKKVNTAIYDHFG.....GQ.DRTD.
tr|A0A1B6YB70|A0A1B6YB70_9BACT      ....SYS.......GG.................KKKEVNTGIYKYFG.....GT.NRTK.
tr|A0A2D4ZTC0|A0A2D4ZTC0_9BACT      ....SYA.......DG.................KKKEVNTEIYKYFG.....GT.NRTN.
tr|A0A3D4UVW7|A0A3D4UVW7_9BACT      ....SYA.......DG.................KKKEVNTEIYKYFG.....GT.NRTN.
tr|A0A3F3I3M2|A0A3F3I3M2_9BACT      ....SYA.......DG.................KKKEVNTEIYKYFG.....GT.NRTN.
tr|A0A3D1G7L9|A0A3D1G7L9_9BACT      ....TYE........S.................KKKQVNTGIYQYFG.....GS.ARSN.
tr|A0A3M8G1Y8|A0A3M8G1Y8_9BACT      ....NYD........G.................KKKSPNSDIYSFFG.....GS.SRNN.
tr|A0A5Q4F391|A0A5Q4F391_9BACT      ....LYP.......SG........KG.......KKKKVNRAIYSYFG.....GE.KRKK.
tr|A0A6I7NPV0|A0A6I7NPV0_9BACT      ....IYE.......EG........SK.......KKKKVNTEVYRFFG.....GE.DRQK.
tr|A0A651G1J2|A0A651G1J2_9BACT      ....RYE.......AG........SK.......KKKEVNKEIYSFFG.....DS.ARKD.
tr|A0A371QRK5|A0A371QRK5_9BACT      ....KYG.......DGS................KKKEINEEIYKFFG.....GT.TRKE.
tr|A0A2N0VGJ5|A0A2N0VGJ5_9BACT      ....TYE.......DGS................KKKEVNREIYGFFG.....GQ.DRKS.
tr|A0A316TYJ0|A0A316TYJ0_9BACT      ....VYE.......SG.....SGDKK.......KKKEVNTEIYSFFG.....GS.DRRS.
tr|A0A651GLZ8|A0A651GLZ8_9BACT      ....RYE.......ES........SK.......KKKEINPKIYEFFG.....GA.NRKS.
tr|A0A7Y5V3S5|A0A7Y5V3S5_9BACT      ....TYD......ATG.................KKGAVDASVYSLLA.....TP.NRSP.
tr|A0A7W1SHC0|A0A7W1SHC0_9BACT      ....LYG......SNG.................KKGDVDTSIYEVMG.....GR.ERRD.
tr|A0A7Y5PBT7|A0A7Y5PBT7_9BACT      ....QYD......DAG.................TKNGVDFSVYELLPN....GA.ARVE.
tr|A0A7Y5TXL8|A0A7Y5TXL8_9BACT      ....QYD......DAG.................TKNGVDFSVYELLPN....GA.ARVE.
tr|A0A423PQ96|A0A423PQ96_9GAMM      ....DYT...AKTKKG.................EKRPANADLERLLAQ....QRDGGRA.
tr|U2G066|U2G066_9GAMM              ....DYT.AK..RKKG.................AKRPVNPELATLLAD....DRQGVRA.
tr|A0A2E0J1F4|A0A2E0J1F4_9GAMM      ....DYT..A...PKK........KG.......EKRPINADLDTLLA.....GERDGGRA
tr|A0A2D4SCM8|A0A2D4SCM8_9GAMM      ....VYD..G...KKSK................GAKPVNTAVYKLMGV....SADNHKQ.
tr|L0WJH1|L0WJH1_9GAMM              ....IYE.....GVAK.................GEKPVNTALYELMNV....TT.SRDA.
tr|A0A1H5XF61|A0A1H5XF61_9GAMM      ....IYE..G....VAK.......AK.......GEKPVNTALYELMNV....SQ..EKD.
tr|A0A2E9TS48|A0A2E9TS48_9GAMM      ....IYE.....GVAK.................GEKPVNTALYELMNV....TS.EKDA.
tr|A0A095UE19|A0A095UE19_9GAMM      ....IYE.....GVAK.................GEKPVNTALYELMNV....TS.EKDA.
tr|A0A7G2S9A0|A0A7G2S9A0_9GAMM      ....IYE.....GVAK.................GEKPVNTALYELMNV....TS.EKDA.
tr|B5JU27|B5JU27_9GAMM              ....LYG.......DGV................KGKPVNQEVFDAMNV....TP..WKN.
tr|A0A1Y0IHP1|A0A1Y0IHP1_9GAMM      ....LYG......PEH.................KGKPVNDAVYNVMGV....SA..SRK.
tr|A0A316FZ14|A0A316FZ14_9GAMM      ....RYD..D....DS.........K.......GKREVDESIYQLLGI....TR..DHS.
tr|A0A498C231|A0A498C231_9GAMM      ....RYD..R....KR........GK.......GGRPVDEGVYRLLGV....TP..HAE.
tr|Q0A6T4|Q0A6T4_ALKEH              ....RYD..R....KR........GK.......GGRPVDEGVYRLLGL....AP..GRD.
tr|A0A3E0WL10|A0A3E0WL10_9GAMM      ....KYE.......GV........KK.......GKKEVDETVYDLLGV....KP.DNSM.
tr|A0A3S1BU16|A0A3S1BU16_9GAMM      ....LYE.......GV........RK.......GEKQVDKSVYAVLGV....SG.DKSI.
tr|A0A7V8QFN9|A0A7V8QFN9_9GAMM      ....DYS..G....KS.................SGKDVDEAVYKLLGV....EP..TGS.
tr|A0A1H8PPF9|A0A1H8PPF9_9GAMM      ....QYG..G....KK........KK.......GKKAVDESVYSLLSI....DP..SNK.
tr|A0A6H0J0V7|A0A6H0J0V7_9GAMM      ....KYG..G....KK........KK.....GGDKEVDESVYSLLGV....DP..SNQ.
tr|V5EZX6|V5EZX6_9VIBR              ....QYK........G.................KDKKVDKSVYKLLKL....TP.SRSI.
tr|A0A6I1QBM2|A0A6I1QBM2_9VIBR      ....QYK........G.................KDKKVDKSVYKLLKL....TP..SRS.
tr|A0A511QRL5|A0A511QRL5_9VIBR      ....QYK........G.................KDKKVDKSVYGLLKL....TP.SRSI.
tr|A0A7Y0JZP4|A0A7Y0JZP4_9VIBR      ....QYK........G.................KDKKVDKSVYGLLKL....TP..SRS.
tr|A0A193KD24|A0A193KD24_9VIBR      ....QYK........G.................KDKKVDKSVYGLLKL....TP..SRS.
tr|A0A1S1HM72|A0A1S1HM72_PROST      ....LYE..H....QASSKLFFWKKA.......HKRQVDKSLYSLVGI....HP..QNH.
tr|A0A7T8I613|A0A7T8I613_9GAMM      ....LYE..H....QASSKLFFWKKA.......HKRQVDKSLYSLVGI....HP..QNH.
tr|A0A140NIF0|A0A140NIF0_PROSM      ....LYG..H....QPTSKLMFWKKS.......KKRQVDSSIYSLIGI....QP..QNH.
tr|A0A379GMB7|A0A379GMB7_PROST      ....LYG..H....QPTSKLMFWKKS.......KKRQVDSSIYSLIGI....QP..QNH.
tr|A0A379H393|A0A379H393_PROST      ....LYG..H....QPASKLMFWKKS.......KKRQVDSSIYSLIGI....QP..QNH.
tr|B2Q0L5|B2Q0L5_PROST              ....LYG..H....QPASKLMFWKKS.......KKRQVDSSIYSLIGI....QP..QNH.
tr|A0A6I3JUE9|A0A6I3JUE9_9GAMM      ....LYG..H....QPSSKLRFWKKS.......KKRQVDKSIYSLINV....HP..KNH.
tr|A0A7D4P594|A0A7D4P594_YERMW      ....LYP..T..QSRG....IKWKRN.......SGKQVDASVYVLLGV....TP..KAH.
tr|A0A7U7IVK6|A0A7U7IVK6_YEREN      ....LYP..A....KN.SGF.KWKRS.......PGKQVDTSVYTLLGV....TP..KAH.
tr|A0A447RIF5|A0A447RIF5_YEREN      ....LYP..A....KN.SGF.KWKRS.......PGKQVDTSVYTLLGV....TP..KAH.
tr|A0A7H4ZLV9|A0A7H4ZLV9_YERP4      ....LYP..A....KN.SGF.KWKRS.......PGKQVDTSVYTLLGV....TP..KAH.
tr|A0A0H3NX46|A0A0H3NX46_YERE1      ....LYP..A....KN.SGF.KWKRS.......PGKQVDTSVYTLLGV....TP..KAH.
tr|A0A7T9XUV7|A0A7T9XUV7_YEREN      ....LYP..A....KN.SGF.KWKRS.......PGKQVDTSVYTLLGV....TP..KAH.
tr|A0A7U7IYV8|A0A7U7IYV8_YEREN      ....LYP..A....KN.SGF.KWKRS.......PGKQVDTSVYTLLGV....TP..KAH.
tr|A0A0H5G7G2|A0A0H5G7G2_YEREN      ....LYP..A....KN.SGF.KWKRS.......PGKQVDTSVYTLLGV....TP..KAH.
tr|A0A2A7TDI2|A0A2A7TDI2_YERKR      ....LYP..A....TS.RGF.KWKKS.......PGKQVDTSVYTLLGV....TP..KAH.
tr|A0A0T9M261|A0A0T9M261_YERKR      ....LYP..A....TS.RGF.KWKKS.......PGKQVDSSVYTLLGV....TP..KAH.
tr|A0A0A0CMP1|A0A0A0CMP1_PHOLU      ....LYA..S....EA.RKFWQFGKK.......G.KKVDSSIYTLLNI....TP..ESH.
tr|A0A329VG55|A0A329VG55_9GAMM      ....LYA..P....GP.RKFWQFGKR.......D.KKVDSSVYTLLNI....TP..ESH.
sp|Q7N288|FADJ_PHOLL                ....LYA.....PGP.RKFWQFGKR.......D.KKVDSSVYTLLNI....TP..ESH.
tr|A0A6L9JMX5|A0A6L9JMX5_PHOLM      ....LYA.......PGPRKFWQFGKR.......DKKVDSSVYTLLNI....TP.ESHM.
tr|A0A7X5HQ29|A0A7X5HQ29_PHOLM      ....LYA.......PGPRKFWQFGKR.......DKKVDSSIYTLLNI....TP.ESHM.
tr|A0A1C0U051|A0A1C0U051_9GAMM      ....LYT.......SETRKFWRIGKK.......GKKVDSSIYTLMNI....TP.ESRM.
tr|A0A7X5QF62|A0A7X5QF62_9GAMM      ....LYT.......SESRKSWRIGKK.......GKKVDSSVYALLNI....KP.ESRM.
```

```
tr|A0A2D0KWM9|A0A2D0KWM9_9GAMM   ....SYA..T....KK.SKFWSLRKN.......S.KEADSSIYSLLKV....QP..VAK.
tr|A0A2D0LAI1|A0A2D0LAI1_9GAMM   ....SYA.......TKKSKFWSLRKN........SKEADSSIYSLLKV....QP..VAK.
tr|A0A2D0IXC8|A0A2D0IXC8_XENBU   ....SYE..V....KK.TRFWSFRKS.......S.KEVDSSIYSLLKI....KP..NAK.
tr|W1JAM5|W1JAM5_9GAMM           ....SYE..V....KK.SRFWSFGKS.......S.KEVDSSIYSLLKI....KP..NAR.
tr|A0A3D9UED3|A0A3D9UED3_9GAMM   ....SYE.......VKKSRFWSFGKS........SKEVDSSIYSLLKI....KP..NAR.
tr|A0A1I3JCA9|A0A1I3JCA9_9GAMM   ....SYA........GKKGMFASFGK.......KSKQVDVSIYSLLKI....KP..AVN.
tr|A0A068QUL9|A0A068QUL9_9GAMM   ....LYA..N....KK.RAFWPFGKK.......SKKEADASVYALLNI....KP..AIK.
tr|A0A0M0TCH6|A0A0M0TCH6_9GAMM   ....LYT..........SRKLYTSRK.......KSKEVDASIYSLLKI....KP..AIK.
tr|A0A1Q5U854|A0A1Q5U854_9GAMM   ....LYA..S....KK.SSFWSFGKK.......SNKEVDASIYSLLKI....KP..AIK.
tr|A0A2D0ISP1|A0A2D0ISP1_9GAMM   ....LYA..S....KK.SSFWPFGKK.......PNKEVDASIYSLLKI....KP..AIK.
tr|A0A1Q5TUI8|A0A1Q5TUI8_9GAMM   ASNKLYA..N....KK.SSFWPFGKK.......SNKEVDASVYSLLKI....KP..AIK.
tr|A0A2D0KJI7|A0A2D0KJI7_9GAMM   ....LYA..N....KK.SSFWPFGKK.......SNKEVDTSIYSLLKI....KP..DIK.
tr|A0A1Y2SB97|A0A1Y2SB97_9GAMM   ....LYT..N....KK.GSFWSFGKK.......A.KQVDVSIYSLLKI....KP..AVK.
tr|A0A1I5DWI7|A0A1I5DWI7_9GAMM   ....SYA.......NKKGSFWSFGKK........AREVDASIYSLLKI....KP.AVKM.
tr|A0A1I7GWY6|A0A1I7GWY6_9GAMM   ....SYV..D....KK.GFFKSFGKT.......S.KEVDTSIYSLLKI....RP..AAK.
tr|D3VKY8|D3VKY8_XENNA           ....SYV..S....KK.GLLKFFSKG.......I.KEVDESIYSLLKI....KP..AAK.
tr|A0A2G0Q6Q9|A0A2G0Q6Q9_9GAMM   ....SYT..N....KK.SSFWSFGKQ.......A.KEVDSSIYSLLKI....KP..AVK.
tr|A0A2D0JU24|A0A2D0JU24_9GAMM   ....SYA..N....KK.NKFWSFGKK.......P.KEVDVSIYSLLKI....KP..AVK.
tr|A0A0J5FN38|A0A0J5FN38_9GAMM   ....SYA.......NKKNKFWSFGKK........SKEVDVSIYSLLKI....KP.TVKM.
tr|A0A432XLD4|A0A432XLD4_9GAMM   ....AYS..G..............KK.......PGKSVDESVYSLLGI....SP..SSK.
tr|A0A2D8HU84|A0A2D8HU84_9GAMM   ....QYG..K..............GV.......KGKPVDTSVYSLLDI....DP..KQS.
tr|A0A656X1Q2|A0A656X1Q2_9GAMM   ....QYG..K..............GV.......KGKPVDTSVYSLLDI....DP..KQS.
tr|A0A4Q1QH79|A0A4Q1QH79_9GAMM   ....KYG.......KGV................KGKPVDTSVYSLLSV....DP.NESK.
tr|A0A1G7LQE6|A0A1G7LQE6_9GAMM   ....KYG.......KGV................KGKPVDTSVYSLLGV....DP.NESK.
tr|A0A432YVG7|A0A432YVG7_9GAMM   ....KYG..K..............GV.......KGKPVDTSVYSLLGV....DP..NES.
tr|A0A1J5N0P5|A0A1J5N0P5_9GAMM   ....RYD.......KK........AR.......GKKLVDESVYKVLGV....SP.SKQT.
tr|A0A0D8D548|A0A0D8D548_9GAMM   ....LYG.......KKV................KKKQVDESIYKVLNI....TP.QGRL.
tr|A0A3E0TNM2|A0A3E0TNM2_9GAMM   ....LYG..K....AA........KK.......GKKQVDETIYGLLGI....TP..SGS.
tr|A0A3E0UD67|A0A3E0UD67_9GAMM   ....LYG..K....AA........KK.......GKKQVDETIYGLLGI....TP..SGS.
tr|A0A3E0U0P4|A0A3E0U0P4_9GAMM   ....LYG..K....AA........KK.......GKKQVDETIYGLLGI....TP..SGS.
tr|A0A0M2V8B1|A0A0M2V8B1_9GAMM   ....LYG..K....TA........KK.......GAKTVDNSVYNVLGV....TP..AGK.
tr|A0A285ITY5|A0A285ITY5_9GAMM   ....LYG..K....SA........KK.......GAKTVDNSVYSVLGV....TP..GGK.
tr|A0A486XTB9|A0A486XTB9_9GAMM   ....LYG..K....AV........KK.......GKKQVDASVYSVLGV....TA..APR.
tr|I1E1G1|I1E1G1_9GAMM           ....RYG..K....TA........KK.......GKKQVDDSVYSVLGV....TP..APR.
tr|A0A1H6KJ68|A0A1H6KJ68_9GAMM   ....LYG..K....NG........KK.......GKKRVDESVYSVLGV....TP..AAR.
tr|A0A0X3Y764|A0A0X3Y764_9GAMM   ....LYG..K....NG........KK.......GKKLVDESVYSVLGV....TP..APR.
tr|A0A2N1YEV4|A0A2N1YEV4_9GAMM   ....LYG..K....NG........KK.......GKKRVDESVYSVLGV....TP..APR.
tr|A0A3P3QCM3|A0A3P3QCM3_9GAMM   ....LYG..A....KA.........K.......GKKQVDASVYSVLGL....NP..AAK.
tr|F7NT72|F7NT72_9GAMM           ....LYG..A....KA.........K.......GKKQVDASVYTILGL....NP..AAK.
tr|A0A3S2TW63|A0A3S2TW63_9GAMM   ....LYG..A....KA.........K.......GKKQVDASVYTVLGL....NP..AAK.
tr|A0A3D5BGW7|A0A3D5BGW7_9GAMM   ....LYG..A....KA........KK.......GKKEVDTSVYALLGV....QP..GGK.
tr|A0A5C7TI17|A0A5C7TI17_9GAMM   ....LYG..K....NA........KK.......GRKDVDSSVYSLLGV....QP..NGR.
tr|A0A0U4W982|A0A0U4W982_9GAMM   ....LYG..K....NA........KK.......GRKEVDTSVYSLLGV....QP.NGRL.
tr|A0A2I0FCM2|A0A2I0FCM2_9GAMM   ....LYG..K....KA........KK.......GKKQVDESIYKLLNL....KP..QAS.
tr|A0A2G2IVL1|A0A2G2IVL1_9GAMM   ....LYG..K....KA........KK.......GKKQVDESIYKLLNL....KP..EAS.
tr|A6FI21|A6FI21_9GAMM           ....LYG..K....KA........KK.......GKKQVDESIYKLLNL....KP..EAS.
tr|A0A4U1BNR2|A0A4U1BNR2_9GAMM   ....RFD.......SD........KK.......GAKPVDESVYKVLGI....TP..SPR.
tr|E1SQ60|E1SQ60_FERBD           ....QYG.......GK........AK.......GKKPVDESVYQVLGI....QP.QGRL.
tr|A0A4Y6IZ09|A0A4Y6IZ09_9GAMM   ....QYG......NKA.................KKKLVDESVYGVLGL....TP.ATDK.
tr|A0A0C3QSZ7|A0A0C3QSZ7_9GAMM   ....QYG......GKS.................KKKLVDESVYGVLGL....TP.GNGG.
tr|A0A6L7HXW0|A0A6L7HXW0_9GAMM   ....QYG......AKS.................KKKLVDESVYGVLGL....TP.GTDS.
sp|A3QFP3|FADJ_SHELP             ....QYG..A..............KS.......KKKLVDESVYGVLGL....TP..GAD.
tr|A0A1E5IXH7|A0A1E5IXH7_SHECO   ....QYG......TKS.................KKKLVDESVYAVLGI....TP.TATS.
tr|A0A411PKQ0|A0A411PKQ0_9GAMM   ....LYG.......AK........AK.......KGKQVDESVYGVLGI....QA.GSDK.
tr|A0A6G9QKM3|A0A6G9QKM3_9GAMM   ....QYG.......PK........AK.......KAKLVDESVYGVLGI....AA.SSDK.
tr|A0A6P1UL63|A0A6P1UL63_9GAMM   ....QYG.......PK........AK.......KAKLVDESVYKVLDI....LI.ASDK.
tr|A0A2N1ERI9|A0A2N1ERI9_9GAMM   ....QYG.......PK........AK.......KAKLVDESVYKVLDI....LI.ASDK.
tr|A0A7W4FU55|A0A7W4FU55_9GAMM   ....QYG.......PK........AK.......KAKLVDESVYKVLDI....LI.ASDK.
sp|Q8ECP7|FADJ_SHEON             ....QYG......AAS.................KKKAVDETVYGVLGI....KP.GVDK.
tr|A0A501XZY8|A0A501XZY8_9GAMM   ....QYG......AAS.................KKKAVDESVYGVLGI....KP.GVDK.
tr|A0A2W5DCZ0|A0A2W5DCZ0_SHEOE   ....QYG......VAS.................KKKAVDESVYGVLGI....KP.GVDK.
tr|A0A1E3V3C8|A0A1E3V3C8_9GAMM   ....QYG......VAS.................KKKAVDESVYGVLGI....KP.GVDK.
tr|A0A1Z4AI20|A0A1Z4AI20_9GAMM   ....QYG......VAS.................KKKAVDESVYGVLGI....KP.GVDK.
tr|A0A7X9LJL9|A0A7X9LJL9_9GAMM   ....QYG......VAS.................KKKAVDESVYGVLGI....KP.GVDK.
tr|A0A073KMY5|A0A073KMY5_9GAMM   ....QYG......VAS.................KKKAVDESVYGVLGI....KP.GVDK.
sp|A0KV76|FADJ_SHESA             ....QYA..AGNKASS.................KKKAVDESVYAVLGI....KP.GMDK.
tr|A0A220UTH7|A0A220UTH7_9GAMM   ....QYA..AGNKASS.................KKKAVDESVYAVLGI....KP.GMDK.
tr|A0A5B8R6W1|A0A5B8R6W1_9GAMM   ....QYA..AGNKASS.................KKKAVDESVYAVLGI....KP.GMDK.
tr|V1DAI4|V1DAI4_9GAMM           ....QYA..AGNKASS.................KKKAVDESVYAVLGI....KP.GMDK.
tr|A0A448CPQ4|A0A448CPQ4_SHEPU   ....QYA..AGNKASS.................KKKAVDESVYGVLGI....KP.GIDK.
tr|A0A252ERQ3|A0A252ERQ3_SHEPU   ....QYA..AGNKASS.................KKKAVDESVYGVLGI....KP.GIDK.
sp|Q0HKD1|FADJ_SHESM             ....QYA..AGNKAPS.................KKKAVDESVYGVLGI....KP.GIDK.
sp|Q0HWN3|FADJ_SHESR             ....QYA..AGNKASS.................KKKVVDESVYGVLGI....KP.GIDK.
tr|F7RQE3|F7RQE3_9GAMM           ....QYG.....QKDS........SK.......KAKVVDESVYGVLGI....KP.GTNK.
tr|B8EE98|B8EE98_SHEB2           ....QYG.......AS........SK.......KAKAVDESVYGVLGI....KP.GTNK.
sp|A6WQ25|FADJ_SHEB8             ....QYG.......AS........SK.......KAKAVDESVYGVLGI....KP.GTNK.
tr|A0A448EK41|A0A448EK41_9GAMM   ....QYG.......AS........SK.......KAKAVDESVYGVLGI....KP.GTNK.
tr|A0A553JHX1|A0A553JHX1_SHEHA   ....LYG..S....KA.........K.......KAKQVDTSVYGILGL....ST.PVNN.
tr|B8CPY6|B8CPY6_SHEPW           ....QYG..S..............KK.......KAKEVDSSVYAVLGL....TP..GQN.
tr|A0A431WFC4|A0A431WFC4_9GAMM   ....LYG..S....KA....KQKFKK.......KAKLVDESVYAVLGL....TP.SSTD.
tr|A8FTR7|A8FTR7_SHESH           ....LYG..S....KA....KQRFKK.......KAKLVDESVYAVLGL....TP.TSTD.
tr|A0A431WNL0|A0A431WNL0_9GAMM   ....LYG..S....KA....KQRFKK.......KAKLVDESVYAVLGL....TP.SSTD.
tr|A0A550AEC5|A0A550AEC5_9GAMM   ....LYG..G....KA........NK.......KAKQVDESVYKVLGL....TP..GTD.
tr|A0A7L4WW90|A0A7L4WW90_9GAMM   ....LYG..G....KA........NK.......KAKQVDESVYKVLGL....TP..GTD.
tr|A9DDU3|A9DDU3_9GAMM           ....LYG..K....KS........NK.......KAKRVDESVYKLLGL....TP.GTSTP
tr|A0A330M2B9|A0A330M2B9_9GAMM   ....LYGKKS....NK.................KAKRVDESVYKLLGL....TPGTSTP.
tr|A0A5N8UFC7|A0A5N8UFC7_9GAMM   ....LYG..G....KA........KK.......KGKQVDESVYQVLGL....KP.GVNA.
tr|A0A1S6HN57|A0A1S6HN57_9GAMM   ....LYA..S....KA........KK.......KGKQVDESVYQVLGL....KP.GVNA.
tr|D4ZMH7|D4ZMH7_SHEVD           ....LYA..G....KA........KK.......KAKQVDESVYQVLGL....KP.GVNA.
tr|A0A3L8Q213|A0A3L8Q213_9GAMM   ....LYG..K....QA........KK.......GKKQVDESVYALFGI....TP..SHD.
tr|A0A3A6U4N9|A0A3A6U4N9_9GAMM   ....LYG..K....QA........KK.......GKKQVDESVYALFGL....TP..KPN.
tr|A0A4Q5MA37|A0A4Q5MA37_9GAMM   ....LYG..K....AA........KK.......GKKQVDESVYKLFNL....NP..TAN.
tr|A0A1L6LSX5|A0A1L6LSX5_9DELT   ....LYG..D....AK........KG.......KGKRVDESVYGVLGLPTPDPK.AKPK.
tr|A0A2W4L9B9|A0A2W4L9B9_9PROT   ....RYG........G.................KRKEVDESIYDLLG.....SR.TRKR.
tr|A0A2W4M4E6|A0A2W4M4E6_9PROT   ....VYG.......EK.......RRK.......GKKPVDTTVYQVLGI....QP.SRDA.
tr|A0A6I2GRX9|A0A6I2GRX9_9DELT   ....LYG......GKG.................KKKEVDPSVYALLPH....GE.KRVQ.
tr|A0A0H4WMK2|A0A0H4WMK2_9DELT   ....LYE.......DG.................KKQDVDPSIYALLPH....GT.ERRS.
tr|F8CJ36|F8CJ36_MYXFH           ....LYE.......GG.................KKQEVDPSIYALLPH....GT.ERRS.
tr|A0A250K0F1|A0A250K0F1_9DELT   ....LYE.......GG.................KKQEVDPSIYALLPH....GT.ERRS.
tr|A0A7Y6WFZ2|A0A7Y6WFZ2_9DELT   ....LYE.......DG.................KKQEVDSSIYALLPH....GT.ERRS.
tr|A0A7Y7C660|A0A7Y7C660_9DELT   ....LYE.......DG.................KKQEVDSSIYALLPH....GT.ERRS.
tr|A0A7Y4JFH2|A0A7Y4JFH2_MYXXA   ....LYE.......DG.................KKQEVDSSIYALLPH....GT.ERRS.
tr|A0A4Y6CZQ9|A0A4Y6CZQ9_MYXXA   ....LYE.......DG.................KKTEVDSSIYALLPH....GT.ERRS.
```

```
tr|A0A7Y4IKV5|A0A7Y4IKV5_MYXXA   ....LYE.......DG.................KKQEVDSSIYALLPH....GT.ERRS.
tr|A0A511HHB0|A0A511HHB0_9DELT   ....LYE.......DG.................KKQEVDSSIYALLPH....GT.ERRS.
tr|A0A4Y6CKY7|A0A4Y6CKY7_MYXXA   ....LYE.......DG.................KKQEVDSSIYALLPH....GT.ERRS.
tr|Q1D1F2|Q1D1F2_MYXXD           ....LYE.......DG.................KKQEVDSSIYALLPH....GT.ERRS.
tr|A0A7Y4MA14|A0A7Y4MA14_MYXXA   ....LYE.......DG.................KKQEVDSSIYALLPH....GT.ERRS.
tr|A0A7T8Y4N9|A0A7T8Y4N9_MYXXA   ....LYE.......DG.................KKQEVDSSIYALLPH....GT.ERRS.
tr|L7UE67|L7UE67_MYXSD           ....LYE.......NG.................KKREVDPSVYVLLPH....GK.ERKR.
tr|A0A511T9X1|A0A511T9X1_MYXFU   ....LYE.......NG.................KKKEVDPTVYGLLPH....GK.ERKG.
tr|A0A7Y7C9C4|A0A7Y7C9C4_9DELT   ....VYE.......NG.................KKKEVDPTVYALLPH....GK.ERKS.
tr|A0A540X7W8|A0A540X7W8_9DELT   ....LYE.......NG.................KKKEVDPTVYALLPH....GK.ERKS.
tr|A0A3A5FK19|A0A3A5FK19_9DELT   ....LYE.......NG.................KKKDVDPTVYALLPH....GK.DRKP.
tr|A0A3A8JQL9|A0A3A8JQL9_9DELT   ....LYE.......DG.................KKKEVDPQVYLLLPH....GK.DRKS.
tr|A0A7Y4NFA4|A0A7Y4NFA4_9DELT   ....LYE.......NG.................KKKEVDPQVYLLLPH....GK.DRKS.
tr|A0A3A8HDQ7|A0A3A8HDQ7_9DELT   ....LYE.......NG.................KKQDVDPQVYLLLPH....GK.DRKS.
tr|A0A3A8GR90|A0A3A8GR90_9DELT   ....LYE.......NG.................KKKEVDPQVYLLLPH....GK.DRKS.
tr|A0A3A8SBD7|A0A3A8SBD7_9DELT   ....LYE.......NG.................KKKEVDPQVYLLLPH....GK.DRKS.
tr|A0A3A8T0I8|A0A3A8T0I8_9DELT   ....LYE.......NG.................KKKEVDPQVYLLLPH....GK.DRKS.
tr|A0A7X5BU07|A0A7X5BU07_9DELT   ....LYE.......NG.................KKKEVDPQVYLLLPH....GK.DRKS.
tr|A0A7Y1RVL2|A0A7Y1RVL2_9DELT   ....LYE.......NG.................KKKEVDPQVYLLLPH....GK.DRKS.
tr|A0A3A8THN2|A0A3A8THN2_9DELT   ....LYE.......NG.................KKKEVDPQVYLLLPH....GK.DRKS.
tr|A0A7Y1RX76|A0A7Y1RX76_9DELT   ....LYE.......NG.................KKKEVDPQVYLLLPH....GK.DRKS.
tr|A0A3A8RA58|A0A3A8RA58_9DELT   ....LYE.......NG.................KKKEVDPQVYLLLPH....GK.DRKS.
tr|A0A410RPB6|A0A410RPB6_CORCK   ....LYE.......NG.................KKKEVDPQVYLLLPH....GK.DRKS.
tr|A0A7Y4J474|A0A7Y4J474_CORCK   ....LYE.......NG.................KKKEVDPQVYLLLPH....GK.DRKS.
tr|A0A3A8I9Z6|A0A3A8I9Z6_9DELT   ....LYE.......NG.................KKKEVDPQVYLLLPH....GK.DRKS.
tr|H8MKE9|H8MKE9_CORCM           ....LYE.......NG.................KKKEVDPQVYLLLPH....GK.DRKS.
tr|A0A3A8H102|A0A3A8H102_9DELT   ....LYE.......NG.................KKKEVDPQVYLLLPH....GK.DRKS.
tr|A0A554FW33|A0A554FW33_9DELT   ....LYE.......NG.................KKKEVDPQVYLLLPH....GK.DRKS.
tr|A0A3A8NPC0|A0A3A8NPC0_9DELT   ....LYE.......NG.................KKQDVDPAVYLLLPH....GK.DRKA.
tr|A0A3A8JUV3|A0A3A8JUV3_9DELT   ....LYE.......NG.................KKQDVDPAVYLLLPH....GK.DRKT.
tr|A0A3A8NEX6|A0A3A8NEX6_9DELT   ....LYE.......NG.................KKQDVDPAVYLLLPH....GK.DRKT.
tr|A0A3A8JEU8|A0A3A8JEU8_9DELT   ....LYE.......DG.................KKKEVDPAVYLLLPH....GK.DRKS.
tr|A0A3A8LI76|A0A3A8LI76_9DELT   ....LYE.......DG.................KKKEVDPSVYLLLPH....GK.DRKS.
tr|A0A085WXN8|A0A085WXN8_9DELT   ....TYT........G.................KKKEVDASVYGLLPG....GA.DRKK.
tr|A0A2T4V0M5|A0A2T4V0M5_9DELT   ....TYD.......GK.................KKKEVDASVYELLPH....GK.NRKN.
tr|A0A0G2ZSW5|A0A0G2ZSW5_9DELT   ....TYD........G.................KKKEVDPTVYELLPH....GK.NRKS.
tr|A0A3M2DKY0|A0A3M2DKY0_9DELT   ....RYD..V..PKKK.................GKRPVDDSVYAVLGV....EP..RTR.
tr|A0A661NQ58|A0A661NQ58_9DELT   ....LYG.......DD........KK.......GERVVDESVYTLLGV....KP.NNKV.
tr|A0A520YD99|A0A520YD99_9DELT   ....LYG..D.......................KKKGVDESVYEVLGV....TP.NNKS.
tr|A0A7Y3BRE4|A0A7Y3BRE4_9DELT   ....LYG..D.......................KKKGVDESVYEVLGV....TP.NNKS.
tr|A0A2D9TF90|A0A2D9TF90_9DELT   ....AYE.......GG.................KSKGVDTSVYEVLGV....TP.TNET.
tr|A0A2E0TP32|A0A2E0TP32_9DELT   ....LYG..D.......................KKKGVDESVYGVLGV....KP.KPGA.
tr|A0A2E4Y3V1|A0A2E4Y3V1_9PROT   ....LYN......EKKSSNPLSKIFQ......GSGKEINPAALKIVRENRIELR.GPQS.
tr|A0A2E6VRH4|A0A2E6VRH4_9DELT   ....LYG.AQ..QNKGMPLEKIKSTLGKPFGVKNRKPINPEAMKVVNQYRIEGK..HNH.
tr|A0A1F9FB59|A0A1F9FB59_9DELT   ....MYE.KH..KQSPVEKARAIVEKAMGAGK.KDKPYNPGALAILERHGV.KPGSKKD.
```

```
ECHA_HUMAN                                      670          680          690          700          710          720
                                                  .            .            .            .            .            .
ECHA_HUMAN                           S....SDEDIQFRLVTRFVNEAVMCLQEGILATPAEGDIGAVFGLGFPPCLGGPFRFVDL
tr|A0A4R6XLA8|A0A4R6XLA8_9GAMM       M....AKQAIQNRIGLIFVNECVHCLEEGILFAAKDGDLAAILGLGFPPFTGGPFSYIDA
tr|A0A6A0IJT2|A0A6A0IJT2_9BACT       F....PAEEIVDRLALLMVSEAVRCLEEGVLASPRDGDVGAILGLGFPPFRGGPFRHVDA
tr|A0A7V3QZU7|A0A7V3QZU7_9BACT       M....DRKAMAERLALLMANEAVHCLQEGVLSSPRDGDVGAVFGLGFPPFRGGPFRYLDA
tr|A0A7V4SYJ6|A0A7V4SYJ6_9BACT       F....PALEMAERLAFLMVNEAVWCLQEGVLACPRDGDVGGILGLGFPPFRGGPFRYLDT
tr|A0A2A8D2N6|A0A2A8D2N6_9BACT       I....PADEVEERLGLMMVNEAIRCLEEGILKDPVDGDLGAVFGLGFPPFRGGPFRYVDQ
tr|A0A2N1TGP0|A0A2N1TGP0_9SPIR       F....TAEEIQQRVSLMMINEAALCLQEGIIADPRDGDIGAVFGLGFPPFTGGPFRYMDS
tr|A0A1G3QGW4|A0A1G3QGW4_9SPIR       I....NVEEVQHRISLMMVNEAVTCLEEGIISSPRDGDMGAVLGLGFPPFRGGPFRYIDG
tr|A0A1G3QTY3|A0A1G3QTY3_9SPIR       I....DVEEVQLRISLMMVNEAITCLEEGIISSPRDGDVGAVLGLGFPPFRGGPFRYVDG
tr|A0A7X9L359|A0A7X9L359_9DELT       F....AEEEIQDRLSLAMINEAALCLQEGILSCARDGDIGAVFGLGFPPFTGGPFRYVDA
tr|A0A522CJY0|A0A522CJY0_9SPIR       F....DFAEIQQRVSLMMVNEAALCLQEGIIAGPRDGDVGAILGLGFPPFRGGPFRHVDS
tr|A0A2N1RRJ3|A0A2N1RRJ3_9SPIR       F....DPAVIQQRVSLMMVNEALLCLEEGIIASPRDGDIGAVFGLGFPPFHGGPFRYIDH
tr|A0A2N2KFN7|A0A2N2KFN7_9DELT       F....DAELIQQRVSLMMINEALICLQEGIISCPRDGDIGAVFGLGFPPFEGGPFRYIDR
tr|A0A1V6AXX9|A0A1V6AXX9_9DELT       F....DAKLIQQRVSMMMINEALICLQEGIISCPRDGDIGAVFGLGFPPFEGGPFRYIDR
tr|A0A2N2HZL0|A0A2N2HZL0_9DELT       F....DAKLIQQRVSMMMINESLLCLQEGIISCPRDGDIGAVFGLGFPPFEGGPFRYIDH
tr|A0A5E8ARP3|A0A5E8ARP3_9BACT       F....KDSEIQDRLSLIMINEAIYCLQEGILKEPRDGDIGAVFGLGFPPFLGGPFRFVDA
tr|A0A662A7B1|A0A662A7B1_9BACT       I....TAKEIQQRMAMMMVNEAVLCLEEGIIEKPLDGDIGAIFGLGFPPFRGGPFRYVDY
tr|D7CV16|D7CV16_TRURR               V....ARERIQERLPLIMMNEAFRCLEEGVLSSPRDGDLGAVFGLGFPPFLGGPFWYADR
tr|A0A3C2AKX4|A0A3C2AKX4_9FLAO       F....DDKEIYERIGMSMVSEAALCLQEGIIANPLDGDVGAVFGLGFPPFRGGPFRYMDT
tr|A0A3B8ZLN3|A0A3B8ZLN3_9PLAN       F....DDQEIVQRIALTMVGEAALCLQEGIVANPLDGDVGAVFGLGFSPFRGGPFRYIDA
tr|A0A345UGV8|A0A345UGV8_9BACT       I....SAEVIQKRMSTVMLNEALLCLQENILDSATDGDLGAVLGLGFPPFHGGPFRYIDL
tr|A0A6C1P6A2|A0A6C1P6A2_9BACT       V....TPAIIQERLLMAMVNEALLCLQDEILLSPADGDLGAILGLGFPPFLGGPFRYIDR
tr|A0A3M1X9W8|A0A3M1X9W8_9BACT       H....PVEEIQNRLVLVLVNEAIHCLQDGILLSPSDGDVGAVLGLGFPPFRGGPFRYVDA
tr|A0A354C796|A0A354C796_9DELT       F....DTLEIQNRLSLVMVNEAALCLQEGILQSPRDGDIGAVFGLGFPPFLGGPFRHIDN
tr|A0A3M1NUM8|A0A3M1NUM8_9BACT       H....APQEIQERLVMVMVNEAALCLQEEILSSPRDGDLGAVLGLGFPPFLGGPFRYIDR
tr|A0A3M2KRC8|A0A3M2KRC8_9BACT       L....PEEEIQNRLSLVMINEAARCLEEGIIANPTDGDLGAVLGLGFPPFLGGPFRYIDR
tr|A0A5S9IHR0|A0A5S9IHR0_9BACT       F....DREEMQLRVALMMVNEAAVCLQDGILKSPRDGDLGAILGLGFPPFRGGPFRYIDT
tr|A0A6M1ST81|A0A6M1ST81_9BACT       I....DTEDLQMRMALVMINEAALCLQEDILESATDGDLGAILGLGFPPFLGGPFRYIDR
tr|A0A521AAE5|A0A521AAE5_9BACT       M....DAQTVQQRMTLMMINEAAWCLQDDILRNPADGDLGAVLGLGFPPFLGGPFRYIDR
tr|A0A1M4ZMD6|A0A1M4ZMD6_9BACT       I....EAQTVQQRMTLMMVNEAAWCLQEDVLRNPTDGDLGAVLGLGFPPFLGGPFRYIDR
tr|A0A2A2GCS2|A0A2A2GCS2_9BACT       I....DEQTVQQRMTLTMVNEAAWCLQEDILHNPTDGDLGAVLGLGFPPFLGGPFRYIDR
tr|A0A5D3YIH7|A0A5D3YIH7_9BACT       M....DANTVQQRMTLTMVNEAAWCLQEDILHNATDGDLGAVMGLGFPPFLGGPFRYIDR
tr|A0A6M1T9G0|A0A6M1T9G0_9BACT       M....DADTVQQRMTLTMVNEAAWCLQEDILHNPVDGDLGAVLGLGFPPFLGGPFRYIDR
tr|A0A6A8Q1Y8|A0A6A8Q1Y8_9BACT       P....DKETAQQRMALMMINEAAWCLQDEIIKNPADGDLGAILGLGFPPFLGGPFRYIDQ
tr|A0A521BVJ3|A0A521BVJ3_9BACT       S....DKETAQQRMALTMINEAAWCLQEDILKSPNDGDLGAILGLGFPPFLGGPFRYIDQ
tr|A0A359E0Y8|A0A359E0Y8_9BACT       P....DKETAQLRMALTMVNEAAWCLEEGILKYPTDGNLGAILGLGFPPFLGGPFRYIDQ
tr|A0A2D8CIM6|A0A2D8CIM6_9BACT       P....DKETAQLRMALTMVNEAAWCLEEGILKYPTDGNLGAILGLGFPPFLGGPFRYIDQ
tr|A0A2D9FWF5|A0A2D9FWF5_9BACT       P....DTKTAQQRIALTMINEAAWCLEEGILKSPDDGDLGAILGLGFPPFLGGPFRYIDQ
tr|A0A1B6YB70|A0A1B6YB70_9BACT       P....DMETAQFRMALTMINEAAYCLEEGILKSATDGDLGAILGLGFPPFLGGPFRYIDR
tr|A0A2D4ZTC0|A0A2D4ZTC0_9BACT       P....DMKTAQFRMALTMINEAAYCLEEGVLKSATDGDLGAILGLGFPPFLGGPFRYIDQ
tr|A0A3D4UVW7|A0A3D4UVW7_9BACT       P....DMKTAQFRMALTMINEAAYCLEEGVLKSATDGDLGAILGLGFPPFLGGPFRYIDQ
tr|A0A3F3I3M2|A0A3F3I3M2_9BACT       P....DMKTAQFRMALTMINEAAYCLEEGVLKSATDGDLGAILGLGFPPFLGGPFRYIDQ
tr|A0A3D1G7L9|A0A3D1G7L9_9BACT       P....DAETAQLRMALMMVNEAVYCLQEDILKSPTDGDLGAILGLGFPPFTGGPFRYIDL
tr|A0A3M8G1Y8|A0A3M8G1Y8_9BACT       I....DAETAQLRMALMMVNEAAYCLQEEILKSPTDGDLGAILGLGFPPFTGGPFRYIDQ
tr|A0A5Q4F391|A0A5Q4F391_9BACT       F....TPEEIQSRMALMMVNEAVLCLQEEILLSAGDGDLGAILGLGFPPFLGGPFRYIDR
tr|A0A6I7NPV0|A0A6I7NPV0_9BACT       F....KVEEIQQRLAMIMVKEAVLCLNEGILEKPEDGDLGAILGLGFPPFLGGPFRYLES
tr|A0A651G1J2|A0A651G1J2_9BACT       F....SDEEIQQRLILMMVNEAVYCLQDGILQSPVDGDLGAIMGLGFPPFLGGPFRYAEG
tr|A0A371QRK5|A0A371QRK5_9BACT       F....KEIEIQQRLALVMVNEAVHCLGEGILDNATDGDLGAILGLGFPPFLGGPFRYIDR
tr|A0A2N0VGJ5|A0A2N0VGJ5_9BACT       I....DQNEIQKRLALVMVNEAVLCLQEGILESPTDGDLGAIMGLGFPPFLGGPFRYIDR
tr|A0A316TYJ0|A0A316TYJ0_9BACT       F....PENEIQKRLTLMMVNEAVYCLQENILQSATDGDLGAILGLGFPPFLGGPFRYIDH
tr|A0A651GLZ8|A0A651GLZ8_9BACT       F....KDDEIQQRLTLMMVNEAVYCLQENILLSPVDGDLGAILGLGFPPFLGGPFRYIDR
tr|A0A7Y5V3S5|A0A7Y5V3S5_9BACT       I....PPEEIRQRCALALANEAVRALEEGVVRSPRDGDIGAVFGIGFPPFRGGPFRWIDA
tr|A0A7W1SHC0|A0A7W1SHC0_9BACT       I....EASEVVERCVLAMVNEAARCLEGGILRSARDGDIGAVFGIGFPPFRGGPFRYIDS
tr|A0A7Y5PBT7|A0A7Y5PBT7_9BACT       L....DPVEIQQRCVLAMVNEALRCLEEGVLRSPRDGDVGAVFGIGFPPFRGGPFRYVDS
tr|A0A7Y5TXL8|A0A7Y5TXL8_9BACT       L....DPVEIQQRCVLAMVNEALRCLEEGVLRSPRDGDVGAVFGIGFPPFRGGPFRYVDS
tr|A0A423PQ96|A0A423PQ96_9GAMM       P....DAAEIVERCTLAFANEAAHCLSERVIREPMHGDIGAVFGLGFLPFTGGPFRYIDA
tr|U2G066|U2G066_9GAMM               P....SAAEIVERCTLLFVNEAAHCLSEQIIDDPMQGDIGAIFGLGFLPFTGGPFRYVDT
tr|A0A2E0J1F4|A0A2E0J1F4_9GAMM       P....DDAEIVERCTLLFANEAAHCLSEKIIREPMHGDIGAIFGLGFLPFTGGPFRYIDT
tr|A0A2D4SCM8|A0A2D4SCM8_9GAMM       M....DATEIAERCTLMMLNEAAYCLGDDIITDPMHGDIGAIFGLGFPPFTGGPFRFADQ
tr|L0WJH1|L0WJH1_9GAMM               .....DDEEIVSRCLWMMINEAAYCLQEGIISEPMHGDIGAIFGLGFPPFRGGPFRYMDS
tr|A0A1H5XF61|A0A1H5XF61_9GAMM       ....ADDEEIVSRCVWLMINEAAYCLQDGIISDPMHGDIGAIFGLGFPPFRGGPFRYMDS
tr|A0A2E9TS48|A0A2E9TS48_9GAMM       .....DDEEIVSRCAWLMINEAAYCLQDGIISDPMHGDIGAIFGLGFPPFRGGPFRYMDS
tr|A0A095UE19|A0A095UE19_9GAMM       .....DDEEIVSRCAWLMINEAAYCLQDGIISDPMHGDIGAIFGLGFPPFRGGPFRYMDS
tr|A0A7G2S9A0|A0A7G2S9A0_9GAMM       .....DDEEIVSRCAWLMINEAAYCLQDGIISDPMHGDIGAIFGLGFPPFRGGPFRYMDS
tr|B5JU27|B5JU27_9GAMM               P....DLVAAAERCTWMMINEAAFCLQEGIIQQPMHGDIGAIFGLGFPPFLGGPFRYMDS
tr|A0A1Y0IHP1|A0A1Y0IHP1_9GAMM       NNTAAELQEAAERCNWMLINEAAFCLQEGILQQPMHGDIGAIFGLGFPPFLGGPFRYMDT
tr|A0A316FZ14|A0A316FZ14_9GAMM       V....KADEIVERCLLMMVNEAARCLDEGIIRSARDGDIGAIFGIGFPPFEGGPFRYMDA
tr|A0A498C231|A0A498C231_9GAMM       L....SAEEIIDRTVLPMINEAVRCRDEGIIRCERDGDIGAVFGIGFPPFRGGPFHYLHR
tr|Q0A6T4|Q0A6T4_ALKEH               M....APEAIIDRTVLPMINEAVRCRDEGIIRCERDGDIGAVFGIGFPPFRGGPFHYLRQ
tr|A0A3E0WL10|A0A3E0WL10_9GAMM       .....NEAEIVDRCVLMMINEAARCYEEDVIRSLRDGDIGAIFGIGFPPFLGGPFRYADN
tr|A0A3S1BU16|A0A3S1BU16_9GAMM       .....AAGEIVERCLLMMLNEAARCLDERIIRSARDGDIGAVFGIGFPPFLGGPFRYADS
tr|A0A7V8QFN9|A0A7V8QFN9_9GAMM       M....KPEEIVDRTVLMMLNEAAICYQEGLIRSARDGDIGAVFGIGFPPFRGGPFRYMDS
tr|A0A1H8PPF9|A0A1H8PPF9_9GAMM       A....RESDIIDRTLLMMLNEAARCYDEGVIRSLRDGDIGAVYGIGFPPFRGGPFRYMDE
tr|A0A6H0J0V7|A0A6H0J0V7_9GAMM       A....PADDIIDRTLLLMVNEAARCWDEGVIRTLRDGDIGAVFGIGFPPFRGGPFRYMDA
tr|V5EZX6|V5EZX6_9VIBR               .....AEEDIALRCVIPLLNEAVRCLDEGIIASARDGDIGAIFGIGFPPFSGGPFRFMDT
tr|A0A6I1QBM2|A0A6I1QBM2_9VIBR       I....AEEDIALRCVIPLLNEAVRCLDEGIIASARDGDIGAIFGIGFPPFSGGPFRFMDT
tr|A0A511QRL5|A0A511QRL5_9VIBR       .....SEEDIALRCTIPLLNEAVRCLDEGIIASARDGDIGAIFGIGFPPFTGGPFRFIDT
tr|A0A7Y0JZP4|A0A7Y0JZP4_9VIBR       L....SEEDIALRCTIPLLNEAVRCLDEGIIASARDGDIGAIFGIGFPPFTGGPFRFIDT
tr|A0A193KD24|A0A193KD24_9VIBR       L....SEDDIALRCTIPLLNEAVRCLDEGIIASARDGDIGAIFGIGFPPFTGGPFRFIDT
tr|A0A1S1HM72|A0A1S1HM72_PROST       L....SKIDIAERCVMLMLNEAVRCLDEKIIQNARDGDIGAVFGIGFPPFFGGPFRYMDS
tr|A0A7T8I613|A0A7T8I613_9GAMM       L....SKIDIAERCVLLMLNEAVRCLDEKIIQNARDGDIGAVFGIGFPPFFGGPFRYMDS
tr|A0A140NIF0|A0A140NIF0_PROSM       L....SKIDIAERCVMLMLNEAVRCLDENIIQNARDGDIGAVFGIGFPPFFGGPFRYMDN
tr|A0A379GMB7|A0A379GMB7_PROST       L....SKIDIAERCVMLMLNEAVRCLDENIIQNARDGDIGAVFGIGFPPFFGGPFRYMDN
tr|A0A379H393|A0A379H393_PROST       L....SKIDIAERCVMLMLNEAVRCLDENIIQNARDGDIGAVFGIGFPPFFGGPFRYMDN
tr|B2Q0L5|B2Q0L5_PROST               L....SKIDIAERCVMLMLNEAVRCLDENIIQNARDGDIGAVFGIGFPPFFGGPFRYMDN
tr|A0A6I3JUE9|A0A6I3JUE9_9GAMM       L....SKIDIAERCVMLMLNEAVRCLDEKIIQNARDGDIGAVFGIGFPPFFGGPFRYMDS
tr|A0A7D4P594|A0A7D4P594_YERMW       L....GSAVIAQRCTMMMLNEAARCLDESIIRSPRDGDIGAVFGIGFPPFLGGPFRYLDS
tr|A0A7U7IVK6|A0A7U7IVK6_YEREN       L....ESAVIAQRCTMMLLNEAARCLDESIIRNPRDGDIGAVFGIGFPPFLGGPFRYMDS
tr|A0A447RIF5|A0A447RIF5_YEREN       L....ESAVIAQRCTMMLLNEAARCLDESIIRNPRDGDIGAVFGIGFPPFLGGPFRYMDS
tr|A0A7H4ZLV9|A0A7H4ZLV9_YERP4       L....ESAVIAQRCTMMLLNEAARCLDESIIRNPRDGDIGAVFGIGFPPFLGGPFRYMDS
tr|A0A0H3NX46|A0A0H3NX46_YERE1       L....ESAVIAQRCTMMLLNEAARCLDESIIRNPRDGDIGAVFGIGFPPFLGGPFRYMDS
tr|A0A7T9XUV7|A0A7T9XUV7_YEREN       L....ESAVIAQRCTMMLLNEAARCLDESIIRNPRDGDIGAVFGIGFPPFLGGPFRYMDS
tr|A0A7U7IYV8|A0A7U7IYV8_YEREN       L....ESAVIAQRCTMMLLNEAARCLDESIIRNPRDGDIGAVFGIGFPPFLGGPFRYMDS
tr|A0A0H5G7G2|A0A0H5G7G2_YEREN       L....ESAVIAQRCTMMLLNEAARCLDESIIRNPRDGDIGAVFGIGFPPFLGGPFRYMDS
tr|A0A2A7TDI2|A0A2A7TDI2_YERKR       L....ESAVIAQRCVMMMLNEAARCLDESIIRNPRDGDIGAVFGIGFPPFLGGPFRYMDS
tr|A0A0T9M261|A0A0T9M261_YERKR       L....ESAVIAQRCVMMMLNEAARCLDESIIRNPRDGDIGAVFGIGFPPFLGGPFRYMDS
tr|A0A0A0CMP1|A0A0A0CMP1_PHOLU       M....LSSEIAQRCVMLMLNEAVRCLDEGIIRSPRDGDIGAVFGIGFPPFFGGPFRYIDS
tr|A0A329VG55|A0A329VG55_9GAMM       M....LSSEIAQRCVMLMLNEAVRCLDEGIIRNPRDGDIGAVFGIGFPPFFGGPFRYIDS
sp|Q7N288|FADJ_PHOLL                 M....LSSEIAQRCVMLMLNEAVRCLDEGIIRSPRDGDIGAVFGIGFPPFFGGPFRYIDS
tr|A0A6L9JMX5|A0A6L9JMX5_PHOLM       L.....SSEIAQRCVMLMLNEAVRCLDEGIIRSPRDGDIGAVFGIGFPPFFGGPFRYIDS
tr|A0A7X5HQ29|A0A7X5HQ29_PHOLM       L.....SSEIAQRCVMLMLNEAVRCLDEGIIRSPRDGDIGAVFGIGFPPFFGGPFRYIDS
tr|A0A1C0U051|A0A1C0U051_9GAMM       .....LSSEIAQRCVMLMLNEAVRCLDEGIIRSPRDGDIGAVFGIGFPPFLGGPFRYIDS
tr|A0A7X5QF62|A0A7X5QF62_9GAMM       .....LSSEIAQRCVMLMLNEAVRCLDERVIRSPRDGDIGAVFGIGFPPFFGGPFRYIDS
```

```
tr|A0A2D0KWM9|A0A2D0KWM9_9GAMM  M....SRPEIAQRCLMLMLNEAVRCLDEGVIRSPRDGDIGAVFGIGFPPFLGGPFRYMDK
tr|A0A2D0LAI1|A0A2D0LAI1_9GAMM  M....SRPDIAQRCLMLMLNEAVRCLDEAIIRSPRDGDIGAVFGIGFPPFLGGPFRYMDK
tr|A0A2D0IXC8|A0A2D0IXC8_XENBU  M....SPSDVAQRCLMPMLNEAVRCLDEGIIRSPRDGDIGAVFGIGFPPFLGGPFRYMDK
tr|W1JAM5|W1JAM5_9GAMM          I....SPSDIAQRCLMPMLNEAVRCLDEGIIRSPRDGDIGAVFGIGFPPFLGGPFRYMDK
tr|A0A3D9UED3|A0A3D9UED3_9GAMM  I....SPSDIAQRCLMPMLNEAVRCLDEGIIRSPRDGDIGAVFGIGFPPFLGGPFRYMDK
tr|A0A1I3JCA9|A0A1I3JCA9_9GAMM  V....LPAEIAQRCVMLMLNEAVRCLDEGIVRSPRDGDIGAVFGIGFPPFLGGPFRYMDQ
tr|A0A068QUL9|A0A068QUL9_9GAMM  M....LPADIAQRCVILMLNEAVRCLEEGIIRSPRDGDIGAVFGIGFPPFLGGPFRYMDK
tr|A0A0M0TCH6|A0A0M0TCH6_9GAMM  M....LPADIAQRCVMLMLNEAVRCLDEGIIRSPRDGDIGAVFGIGFPPFLGGPFRYIDK
tr|A0A1Q5U854|A0A1Q5U854_9GAMM  M....LPADIAQRCVMLMLNEAVRCLDEGIIRSPRDGDIGAVFGIGFPPFLGGPFRYIDK
tr|A0A2D0ISP1|A0A2D0ISP1_9GAMM  M....LPADIAQRCVMLMLNEAVRCLDEGIIRSPRDGDIGAVFGIGFPPFLGGPFRYIDR
tr|A0A1Q5TUI8|A0A1Q5TUI8_9GAMM  M....LPADIAQRCVMLMLNEAVRCLDEGIIRSPRDGDIGAVFGIGFPPFLGGPFRYIDK
tr|A0A2D0KJI7|A0A2D0KJI7_9GAMM  M....LPADIAQRCVILMLNEAVRCLDEGIIRNPRDGDIGAVFGIGFPPFLGGPFRYIDK
tr|A0A1Y2SB97|A0A1Y2SB97_9GAMM  M....LPADIVQRCVMLMLNEAVRCLDEGIIRSPRDGDIGAVFGIGFPPFLGGPFRYIDK
tr|A0A1I5DWI7|A0A1I5DWI7_9GAMM  .....LPADIAQRCVMLMLNEAVRCLAEGIIRSPRDGDIGAVFGIGFPPFLGGPFRYIDK
tr|A0A1I7GWY6|A0A1I7GWY6_9GAMM  M....SPVDIAERCVMLMLNEAVHCLDEGIIRSPRDGDIGAVFGIGFPPFLGGPFRYIDQ
tr|D3VKY8|D3VKY8_XENNA          M....SPADIAQRCVMLMLNEAVRCLDENIIRSPRDGDIGAVFGIGFPPFLGGPFRYIDH
tr|A0A2G0Q6Q9|A0A2G0Q6Q9_9GAMM  M....LPVDIAQRCVMLMLNEAARCLEEGVIRSPRDGDIGAVFGIGFPPFLGGPFRYIDK
tr|A0A2D0JU24|A0A2D0JU24_9GAMM  M....LPADIAQRCVMLMLNEAVRCLDEGIIRSPRDGDIGAVFGIGFPPFLGGPFRYIDK
tr|A0A0J5FN38|A0A0J5FN38_9GAMM  L.....PADIAQRCVMLMLNEAVRCLDEGIIRSPRDGDIGAVFGIGFPPFLGGPFRYIDK
tr|A0A432XLD4|A0A432XLD4_9GAMM  L....SGEEIAQRTALLMLNEAAYCLAEGIIRSPRDGDIGAIFGIGFPPFRGGPFRYMDA
tr|A0A2D8HU84|A0A2D8HU84_9GAMM  K....SADEIIDICLLPMLNEAAYCLQEEIIRSPRDGDIGAIFGIGFPPFLGGPFRYMDS
tr|A0A656X1Q2|A0A656X1Q2_9GAMM  K....SADEIIDICLLPMLNEAAYCLQEEIIRSPRDGDIGAIFGIGFPPFLGGPFRYMDS
tr|A0A4Q1QH79|A0A4Q1QH79_9GAMM  .....SAEDIVDICLLPMLNEAVYCLQEGIIRSPRDGDIGAIFGIGFPPFLGGPFRYMDS
tr|A0A1G7LQE6|A0A1G7LQE6_9GAMM  .....SAEDIVDICLLPMLNEAVYCLQEGIIRSPRDGDIGAIFGIGFPPFLGGPFRYMDS
tr|A0A432YVG7|A0A432YVG7_9GAMM  K....SAEDIVDICLLPMLNEAVYCLQEGIIRSPRDGDIGAIFGIGFPPFLGGPFRYMDS
tr|A0A1J5N0P5|A0A1J5N0P5_9GAMM  .....SHQEIAQRCVIQMLNEATRCLEEGIINNVRDGDIGAIFGIGFPPFLAGPFSYIDK
tr|A0A0D8D548|A0A0D8D548_9GAMM  .....SDDEISQRCVYMMLNEAVRCLDEGIVRNARDGDIGAIFGIGFPPFLGGPFRYLDQ
tr|A0A3E0TNM2|A0A3E0TNM2_9GAMM  L....TEQEIAKRSVYMMLNEAMRCLDEGVVRNARDGDIGAIFGIGFPPFLGGPYRYIDK
tr|A0A3E0UD67|A0A3E0UD67_9GAMM  L....TEQEIAKRSVYMMLNEAMRCLDEGVVRNARDGDIGAIFGIGFPPFLGGPFRYIDK
tr|A0A3E0U0P4|A0A3E0U0P4_9GAMM  L....TEQEIAKRSVYMMLNEAMRCLGEGVVRNARDGDIGAIFGIGFPPFLGGPFRYIDK
tr|A0A0M2V8B1|A0A0M2V8B1_9GAMM  L....SSEAIAARCTVQMLNEAVRCLDEGVIACARDGDIGAIFGIGFPPFLGGPFRYIDT
tr|A0A285ITY5|A0A285ITY5_9GAMM  L....SSDAIANRCTAQMLNEAVRCLDEGVIASARDGDIGAIFGIGFPPFLGGPFRYIDT
tr|A0A486XTB9|A0A486XTB9_9GAMM  L....IRDEISSRCVVQMLNEAVRCLEEGIIASARDGDIGAIFGIGFPPFLGGPFRYIDQ
tr|I1E1G1|I1E1G1_9GAMM          L....NSDEISSRCVVQMLNEAVRCLEEGIIASARDGDIGAIFGIGFPPFLGGPFRYIDQ
tr|A0A1H6KJ68|A0A1H6KJ68_9GAMM  L....NSDEISNRCVVQMLNEAVRCLDEGIIASARDGDIGAIFGIGFPPFLGGPFRYIDQ
tr|A0A0X3Y764|A0A0X3Y764_9GAMM  L....NSDEISNRCVVQMLNEAVRCLDEGIIASARDGDIGAIFGIGFPPFLGGPFRYIDQ
tr|A0A2N1YEV4|A0A2N1YEV4_9GAMM  L....NSDEISNRCVVQMLNEAVRCLDEGIIASARDGDIGAIFGIGFPPFLGGPFRYIDQ
tr|A0A3P3QCM3|A0A3P3QCM3_9GAMM  L....SGDQIALRCVVQMLNEAVRCLEEGIISSARDGDIGAIFGIGFPPFLGGPFRYIDQ
tr|F7NT72|F7NT72_9GAMM          L....GGDQIALRCVVQMLNEAVRCLEEGIISSARDGDIGAIFGIGFPPFLGGPFRYIDQ
tr|A0A3S2TW63|A0A3S2TW63_9GAMM  L....SGDQIALRCVVQMLNEAVRCLEEGIISSARDGDIGAIFGIGFPPFLGGPFRYIDQ
tr|A0A3D5BGW7|A0A3D5BGW7_9GAMM  L....SAEQISNRCLVQMLNEAVRCLEEGIIASARDGDIGAIFGIGFPPFIGGPFRYIDA
tr|A0A5C7TI17|A0A5C7TI17_9GAMM  L....SSDQISKRCMVQMLNEAVRCLEEGIIASARDGDIGAIFGIGFPPFLGGPFRYIDS
tr|A0A0U4W982|A0A0U4W982_9GAMM  .....STEQISKRCMVQMLNEAVRCLEEGIIASARDGDIGAIFGIGFPPFLGGPFRYIDS
tr|A0A2I0FCM2|A0A2I0FCM2_9GAMM  M....APKALAERAVLLMLNEAARCLDEGILRSARDGDIGAIFGIGFPPFLGGPFHYMDS
tr|A0A2G2IVL1|A0A2G2IVL1_9GAMM  M....APKALAERAVLLMLNEAARCLDEGILRSARDGDIGAIFGIGFPPFLGGPFHYMDS
tr|A6FI21|A6FI21_9GAMM          M....APKALAERAVLLMLNEAARCLDEGILRSARDGDIGAIFGIGFPPFLGGPFHYMDS
tr|A0A4U1BNR2|A0A4U1BNR2_9GAMM  L....SDSEIAERCVVQMLNEAARCLDEGIIASARDGDIGAIFGIGFPPFLGGPFRYMDK
tr|E1SQ60|E1SQ60_FERBD          .....SQSELAERCVVQMLNEAVRCLEEGIIASPRDGDIGAIFGIGFPPFLGGPFRYIDR
tr|A0A4Y6IZ09|A0A4Y6IZ09_9GAMM  .....EPRAVAERCVMQMLNEAVRCLEEGIISSPRDGDIGAIFGIGFPPFLGGPFRYMDS
tr|A0A0C3QSZ7|A0A0C3QSZ7_9GAMM  .....EPRALAERCVVQMLNEAVRCLEEGIIASPRDGDIGAIFGIGFPPFLGGPFRYMDS
tr|A0A6L7HXW0|A0A6L7HXW0_9GAMM  .....EPRALAERCVVQMLNEAVRCLEEGIIASPRDGDIGAIFGIGFPPFLGGPFRYMDS
sp|A3QFP3|FADJ_SHELP            G....EPIALAERCVVQMLNEAVRCLEEGIIASPRDGDIGAIFGIGFPPFLGGPFRYMDS
tr|A0A1E5IXH7|A0A1E5IXH7_SHECO  .....DAAEVIQRCVVQMLNEAVRCLEEGIIASARDGDIGAIFGIGFPPFLGGPFRYIDT
tr|A0A411PKQ0|A0A411PKQ0_9GAMM  .....DIKQVAERCVVQMLNEAVRCLEEKIIASPRDGDIGAIFGIGFPPFLGGPFRYIDS
tr|A0A6G9QKM3|A0A6G9QKM3_9GAMM  .....EAKEVAERCTIQMLNEAVRCLEEGIIASARDGDIGAIFGIGFPPFLGGPFRYIDS
tr|A0A6P1UL63|A0A6P1UL63_9GAMM  .....EAKDVAERCTIQMLNEAVRCLEEGIIASARDGDIGAIFGIGFPPFLGGPFRYIDT
tr|A0A2N1ERI9|A0A2N1ERI9_9GAMM  .....EAKDVAERCTIQMLNEAVRCLEEGIIASARDGDIGAIFGIGFPPFLGGPFRYIDT
tr|A0A7W4FU55|A0A7W4FU55_9GAMM  .....EAKDVAERCTIQMLNEAVRCLEEGIIASARDGDIGAIFGIGFPPFLGGPFRYIDT
sp|Q8ECP7|FADJ_SHEON            .....EMSAVAERCVVQMLNEAVRCLDAGIIASPRDGDIGAIFGIGFPPFLGGPFHYIDT
tr|A0A501XZY8|A0A501XZY8_9GAMM  .....EMSAVAERCVVQMLNEAVRCLDEGIIASPRDGDIGAIFGIGFPPFLGGPFHYIDT
tr|A0A2W5DCZ0|A0A2W5DCZ0_SHEOE  .....EMSAVAERCVVQMLNEAVRCLDEGIIASPRDGDIGAIFGIGFPPFLGGPFHYIDT
tr|A0A1E3V3C8|A0A1E3V3C8_9GAMM  .....EMSAVAERCVVQMLNEAVRCLDEGIIASPRDGDIGAIFGIGFPPFLGGPFHYIDT
tr|A0A1Z4AI20|A0A1Z4AI20_9GAMM  .....EMSAVAERCVVQMLNEAVRCLDEGIIASPRDGDIGAIFGIGFPPFLGGPFHYIDT
tr|A0A7X9LJL9|A0A7X9LJL9_9GAMM  .....EMSAVAERCVVQMLNEAVRCLDEGIIASPRDGDIGAIFGIGFPPFLGGPFHYIDT
tr|A0A073KMY5|A0A073KMY5_9GAMM  .....EMSAVAERCVVQMLNEAVRCLDEGIIASPRDGDIGAIFGIGFPPFLGGPFHYIDT
sp|A0KV76|FADJ_SHESA            .....DLSAVAERCVVQMLNEAVRCLDDGIIASPRDGDIGAIFGIGFPPFLGGPFHYIDT
tr|A0A220UTH7|A0A220UTH7_9GAMM  .....DLSAVAERCVVQMLNEAVRCLDDGIIASPRDGDIGAIFGIGFPPFLGGPFHYIDT
tr|A0A5B8R6W1|A0A5B8R6W1_9GAMM  .....DLSAVAERCVVQMLNEAVRCLDDGIIASPRDGDIGAIFGIGFPPFLGGPFHYIDT
tr|V1DAI4|V1DAI4_9GAMM          .....DLSAVAERCVVQMLNEAVRCLDDGIIASSRDGDIGAIFGIGFPPFLGGPFHYIDT
tr|A0A448CPQ4|A0A448CPQ4_SHEPU  .....EMSSVAERCVVQMLNEAVRCLDDGIIASARDGDIGAIFGIGFPPFLGGPFHYIDT
tr|A0A252ERQ3|A0A252ERQ3_SHEPU  .....EMSAVAERCVVQMLNEAVRCLDDSIIASPRDGDIGAIFGIGFPPFLGGPFHYIDT
sp|Q0HKD1|FADJ_SHESM            .....EMSAVAERCVVQMLNEAVRCLDDGIIASPRDGDIGAIFGIGFPPFLGGPFHYIDT
sp|Q0HWN3|FADJ_SHESR            .....EMSAVAERCVVQMLNEAVRCLDDGIIASPRDGDIGAIFGIGFPPFLGGPFHYIDT
tr|F7RQE3|F7RQE3_9GAMM          .....DAKAVAERCVVQMLNEAVRCLDEGIIASPRDGDIGAIFGIGFPPFLGGPFHYIDT
tr|B8EE98|B8EE98_SHEB2          .....DAKAVAERCVVQMLNEAVRCLEDGIIASPRDGDIGAIFGIGFPPFLGGPFHYIDT
sp|A6WQ25|FADJ_SHEB8            .....DAKAVAERCVVQMLNEAVRCLEDGIIASPRDGDIGAIFGIGFPPFLGGPFHYIDT
tr|A0A448EK41|A0A448EK41_9GAMM  .....DAKAVAERCVVQMLNEAVRCLEDGIIASPRDGDIGAIFGIGFPPFLGGPFHYIDT
tr|A0A553JHX1|A0A553JHX1_SHEHA  GANSSELAEVSQRCVVQMLNEAVRCLEEGIIASPRDGDIGAIFGIGFPPFLGGPFHYIDT
tr|B8CPY6|B8CPY6_SHEPW          S....DANELALRCVVQMLNEAVRCLEEGIIASARDGDIGAIFGIGFPPFLGGPFHYIDT
tr|A0A431WFC4|A0A431WFC4_9GAMM  SNK..DSQDVAKRCVVQMLNEAVRCLEEGIIASPRDGDIGAIFGIGFPPFLGGPFHYIDS
tr|A8FTR7|A8FTR7_SHESH          SNN..DSQDVAQRCVVQMLNEAVRCLEEGIIASPRDGDIGAIFGIGFPPFLGGPFHYIDS
tr|A0A431WNL0|A0A431WNL0_9GAMM  SNN..DSQDVAQRCVVQMLNEAVRCLEEGIIASPRDGDIGAIFGIGFPPFLGGPFHYIDS
tr|A0A550AEC5|A0A550AEC5_9GAMM  G....EQSEVAQRCVVQMLNEAVRCLEEGIIASPRDGDIGAIFGIGFPPFLGGPFHYIDS
tr|A0A7L4WW90|A0A7L4WW90_9GAMM  G....EQSEVAQRCVVQMLNEAVRCLEEGIIASPRDGDIGAIFGIGFPPFLGGPFHYIDS
tr|A9DDU3|A9DDU3_9GAMM          G....ELADVAERCVVQMLNEAVRCLEEGIIASPRDGDIGAIFGIGFPPFLGGPFHYIDT
tr|A0A330M2B9|A0A330M2B9_9GAMM  G....ELADIAERCVVQMLNEAVRCLEEGIIASPRDGDIGAIFGIGFPPFLGGPFHYIDT
tr|A0A5N8UFC7|A0A5N8UFC7_9GAMM  .....EVSEVAQRCVVQMLNEAVRCLEEGIIASPRDGDIGAIFGIGFPPFLGGPFHYIDT
tr|A0A1S6HN57|A0A1S6HN57_9GAMM  .....EVSEVAQRCVVQMLNEAVRCLEEGIIASPRDGDIGAIFGIGFPPFLGGPFHYIDT
tr|D4ZMH7|D4ZMH7_SHEVD          .....EVSEVAQRCVVQMLNEAVRCLEEGIIASPRDGDIGAIFGIGFPPFLGGPFHYIDT
tr|A0A3L8Q213|A0A3L8Q213_9GAMM  H....DPKKTVQRCVVQMLNEAARCLDEGIIASPRDGDIGAIFGIGFPPFLGGPFRYMDE
tr|A0A3A6U4N9|A0A3A6U4N9_9GAMM  K....EASKLSQRCVVQMLNEAARCLDEGIIACPRDGDIGAIFGIGFPPFLGGPFRYMDE
tr|A0A4Q5MA37|A0A4Q5MA37_9GAMM  K....DPKMVAERCVVQMLNEAARCLEDGIIASPRDGDIGAIFGIGFPPFLGGPFKYMDQ
tr|A0A1L6LSX5|A0A1L6LSX5_9DELT  V....AVEEIQMRCSLQFVNEALHCFGEGILRSPRDGDIGAIFGLGFPPFRGGPFRYVDA
tr|A0A2W4L9B9|A0A2W4L9B9_9PROT  F....SREEIQERLYLQMCNEAALCLQEGILQEPRDGDVGAVFGLGFPPFRGGPFRWMDA
tr|A0A2W4M4E6|A0A2W4M4E6_9PROT  .....PVADVAQRCVLMMVNEAVRCLDEGILRSARDGDIGAVFGLGFPPFRGGPFRFVDA
tr|A0A6I2GRX9|A0A6I2GRX9_9DELT  L....DRQEMAERCSLQMVNEAIRCLGEGVLRSARDGDVGAIFGLGFPPFLGGPFRYADS
tr|A0A0H4WMK2|A0A0H4WMK2_9DELT  F....DRAEMAERVVLQMVNEAIRCLGEGILRSARDGDVGAIFGLGFPPFLGGPFHYVDS
tr|F8CJ36|F8CJ36_MYXFH          F....DRAEMAERVVLQMVNEAIRCLGEGILRSARDGDVGAIFGLGFPPFLGGPFHYVDS
tr|A0A250K0F1|A0A250K0F1_9DELT  F....DRAEMAERVVLQMVNEAIRCLGEGILRSARDGDVGAIFGLGFPPFLGGPFHYVDS
tr|A0A7Y6WFZ2|A0A7Y6WFZ2_9DELT  F....DRAEMAERVVLQMVNEAIRCLGEGILRSARDGDVGAIFGLGFPPFLGGPFHYVDS
tr|A0A7Y7C660|A0A7Y7C660_9DELT  F....DRAEMAERVVLQMVNEAIRCLGEGILRSARDGDVGAIFGLGFPPFLGGPFHYVDS
tr|A0A7Y4JFH2|A0A7Y4JFH2_MYXXA  F....DRAEMAERVVLQMVNEAIRCLGEGILRSARDGDVGAIFGLGFPPFLGGPFHYVDS
tr|A0A4Y6CZQ9|A0A4Y6CZQ9_MYXXA  F....DRAEMAERVVLQMVNEAIRCLGEGILRSARDGDVGAIFGLGFPPFLGGPFHYVDS
```

```
tr|A0A7Y4IKV5|A0A7Y4IKV5_MYXXA  F....DRAEMAERVVLQMVNEAIRCLGEGVLRSARDGDVGAIFGLGFPPFLGGPFHYVDS
tr|A0A511HHB0|A0A511HHB0_9DELT  F....DRAEMAERVVLQMVNEAIRCLGEGILRSARDGDVGAIFGLGFPPFLGGPFHYVDS
tr|A0A4Y6CKY7|A0A4Y6CKY7_MYXXA  F....DRAEMAERVVLQMVNEAIRCLGEGILRSARDGDVGAIFGLGFPPFLGGPFHYVDS
tr|Q1D1F2|Q1D1F2_MYXXD          F....DRAEMAERVVLQMVNEAIRCLGEGILRSARDGDVGAIFGLGFPPFLGGPFHYVDS
tr|A0A7Y4MA14|A0A7Y4MA14_MYXXA  F....DRAEMAERVVLQMVNEAIRCLGEGILRSARDGDVGAIFGLGFPPFLGGPFHYVDS
tr|A0A7T8Y4N9|A0A7T8Y4N9_MYXXA  F....DRAEMAERVVLQMVNEAIRCLGEGILRSARDGDVGAIFGLGFPPFLGGPFHYVDS
tr|L7UE67|L7UE67_MYXSD          F....DAAEMAERLVLQMVNEAIRCLGEGILRSARDGDVGAIFGLGFPPFLGGPFHYADS
tr|A0A511T9X1|A0A511T9X1_MYXFU  F....DASEMAERLVLQMVNEAIRCLGEGILRSARDGDVGAIFGLGFPPFLGGPFHYADA
tr|A0A7Y7C9C4|A0A7Y7C9C4_9DELT  F....DASEMAERLVLQMVNEAIRCLGEGILRSARDGDVGAIFGLGFPPFLGGPFHYADS
tr|A0A540X7W8|A0A540X7W8_9DELT  F....DASEMAERLVFQMVNEAIRCLGEGILRSARDGDVGAIFGLGFPPFLGGPFHFADS
tr|A0A3A5FK19|A0A3A5FK19_9DELT  M....ERAEMAERCALQMVNEAIRCLGENILRSPRDGDVGAIFGLGFPPFLGGPFRYADS
tr|A0A3A8JQL9|A0A3A8JQL9_9DELT  L....DAAEMAERVALQMVNEAIRCLGEGILRSPRDGDVGAIFGLGFPPFLGGPFRYADA
tr|A0A7Y4NFA4|A0A7Y4NFA4_9DELT  L....DASEMAERVALQMVNEAIRCLGEGILRSPRDGDVGAIFGLGFPPFLGGPFRYADA
tr|A0A3A8HDQ7|A0A3A8HDQ7_9DELT  L....DASEMAERVALQMVNEAIRCLGEGILRSPRDGDVGAIFGLGFPPFLGGPFRYADA
tr|A0A3A8GR90|A0A3A8GR90_9DELT  L....DASEMAERVALQMVNEAIRCLGEGILRSPRDGDVGAIFGLGFPPFLGGPFRYADA
tr|A0A3A8SBD7|A0A3A8SBD7_9DELT  L....DPAEMAERVALQMVNEAIRCLGEGILRSPRDGDVGAIFGLGFPPFLGGPFRYADA
tr|A0A3A8T0I8|A0A3A8T0I8_9DELT  L....DPAEMAERVALQMVNEAIRCLGEGILRSPRDGDVGAIFGLGFPPFLGGPFRYADA
tr|A0A7X5BU07|A0A7X5BU07_9DELT  L....DPAEMAERVALQMVNEAIRCLGEGILRSPRDGDVGAIFGLGFPPFLGGPFRYADA
tr|A0A7Y1RVL2|A0A7Y1RVL2_9DELT  L....DPAEMAERVALQMVNEAIRCLGEGILRSPRDGDVGAIFGLGFPPFLGGPFRYADA
tr|A0A3A8THN2|A0A3A8THN2_9DELT  L....DPAEMAERVALQMVNEAIRCLGEGILRSPRDGDVGAIFGLGFPPFLGGPFRYADA
tr|A0A7Y1RX76|A0A7Y1RX76_9DELT  L....DPAEMAERVALQMVNEAIRCLGEGILRSPRDGDVGAIFGLGFPPFLGGPFRYADA
tr|A0A3A8RA58|A0A3A8RA58_9DELT  L....DASEMAERVALQMVNEAIRCLGEGILRSARDGDVGAIFGLGFPPFLGGPFRYADA
tr|A0A410RPB6|A0A410RPB6_CORCK  L....DAAEMAERVALQMVNEAIRCLGEGILRSARDGDVGAIFGLGFPPFLGGPFRYADA
tr|A0A7Y4J474|A0A7Y4J474_CORCK  L....DAAEMAERVALQMVNEAIRCLGEGILRSARDGDVGAIFGLGFPPFLGGPFRYADA
tr|A0A3A8I9Z6|A0A3A8I9Z6_9DELT  L....DAAEMAERVALQMVNEAIRCLGEGILRSARDGDVGAIFGLGFPPFLGGPFRYADA
tr|H8MKE9|H8MKE9_CORCM          L....EASEMAERVALQMVNEAIRCLGEGILRNARDGDVGAIFGLGFPPFLGGPFRYADA
tr|A0A3A8H102|A0A3A8H102_9DELT  L....DAAEMAERVALQMVNEAIRCLGEGVLRSARDGDVGAIFGLGFPPFLGGPFRYADA
tr|A0A554FW33|A0A554FW33_9DELT  M....DPTEMAERCALQMVNEAIRCLGEGILRSPRDGDVGAIFGLGFPPFLGGPLRYADS
tr|A0A3A8NPC0|A0A3A8NPC0_9DELT  M....DPTEMAERCALQMVNEAIRCLGEGILRSPRDGDVGAIFGLGFPPFLGGPLRYADS
tr|A0A3A8JUV3|A0A3A8JUV3_9DELT  M....DPTEMAERCALQMVNEAIRCLGEGILRSPRDGDVGAIFGLGFPPFLGGPLRYADS
tr|A0A3A8NEX6|A0A3A8NEX6_9DELT  M....DPTEMAERCALQMVNEAIRCLGEGILRSPRDGDVGAIFGLGFPPFLGGPLRYADS
tr|A0A3A8JEU8|A0A3A8JEU8_9DELT  L....DAAEMAERCALQMVNEAIRCLGEGILRSPRDGDVGAIFGLGFPPFLGGPLRYADS
tr|A0A3A8LI76|A0A3A8LI76_9DELT  L....DPAEMAERCALQMVNEAIRCLGEGILRSPRDGDVGAIFGLGFPPFLGGPLRYADS
tr|A0A085WXN8|A0A085WXN8_9DELT  F....DRTEMAERCALQMVNEAIRCLGEGILRSPRDGDVGAIFGLGFPPFLGGPFRYADS
tr|A0A2T4V0M5|A0A2T4V0M5_9DELT  I....DAREMAERCALQMVNEAVRCLGEGILRSARDGDVGAIFGLGFPPFLGGPFRYADS
tr|A0A0G2ZSW5|A0A0G2ZSW5_9DELT  L....DAREMAERCALQMVNEAVRCLGEGILRSARDGDVGAIFGLGFPPFLGGPFRYADS
tr|A0A3M2DKY0|A0A3M2DKY0_9DELT  M....APEEIARRCALQMVNEAAYCFGDGILRCARDGDIGAVFGLGFPPFRGGPFRYADA
tr|A0A661NQ58|A0A661NQ58_9DELT  .....PSAEIAERCTLQMVNEAIHCLGEGILRSPRDGDIGAIFGLGFPPFRGGPFRYVDT
tr|A0A520YD99|A0A520YD99_9DELT  V....SNEDIAWRCALQFVNEACRCFGDGILRSARDGDIGAVFGLGFPPFRGGPFRFVDQ
tr|A0A7Y3BRE4|A0A7Y3BRE4_9DELT  V....SNEDIAWRCALQFVNEACRCFGDGILRSARDGDIGAVFGLGFPPFRGGPFRFVDQ
tr|A0A2D9TF90|A0A2D9TF90_9DELT  L....SNDDIAWRCALMMVNEAVLCFQEGILRSARDGDIGAIFGLGFPPFTGGPFRFVDL
tr|A0A2E0TP32|A0A2E0TP32_9DELT  L....SAEEIAWRCTLQMVNEACLCFGQGILRSARDGDIGAIFGLGFPPFRGGPFRFVDA
tr|A0A2E4Y3V1|A0A2E4Y3V1_9PROT  S....DE.ELQKRIAYRMINESALCLQENIIKNPIDGDIGAVFGLGFPPTFGGPFRYLDL
tr|A0A2E6VRH4|A0A2E6VRH4_9DELT  .....DPARVQQRMALRFVNEAVLCLQEGILFSPVEGDIGAVFGLGFPPMRGGPFRHVDS
tr|A0A1F9FB59|A0A1F9FB59_9DELT  V....DERELQERIGLRMVNEAVQCLQDGILEGPVDGDAGAVFGLGFPPMTGGPFRYVDA
```

```
ECHA_HUMAN
                                       730       740       750       760
ECHA_HUMAN                      YGAQKIVDRLKKYEAAYGKQFTPCQLLADHANSP...NKKFYQ.................
tr|A0A4R6XLA8|A0A4R6XLA8_9GAMM  NGADQIVSTLSELEATHGTRFTPAKLLLKHAKNN....QAFVN.................
tr|A0A6A0IJT2|A0A6A0IJT2_9BACT  VGADRVVARLGELAEKCGPRFAPPDLLASMAREG....KKFH..................
tr|A0A7V3QZU7|A0A7V3QZU7_9BACT  LGARQAVNQYEALAKLHGQRFTPAPLLVEMAKSG....KKFYEG................
tr|A0A7V4SYJ6|A0A7V4SYJ6_9BACT  LGAGEAVRRMEGYAERLGPRFRPAPLLEEAARKG....TKFYPPG...............
tr|A0A2A8D2N6|A0A2A8D2N6_9BACT  ERPGAVRTRLERLERDHGSRFKPSSLLVQHAEKD....TTFHND................
tr|A0A2N1TGP0|A0A2N1TGP0_9SPIR  QGIGSVTARMEQCVEKYGERFRPAQILKDMAVAG....KKFYPNN...............
tr|A0A1G3QGW4|A0A1G3QGW4_9SPIR  MGAGAIVALMEKLAVTYGIRFTPAGMLKQMASRG....KRFYQ.................
tr|A0A1G3QTY3|A0A1G3QTY3_9SPIR  MGAGAIVKIMEQLAAKYGKRFIPAGMLQDMASKG....GKFYK.................
tr|A0A7X9L359|A0A7X9L359_9DELT  VGAKTLVEKMKRMEQGFGALYKPADILVDMAAGG....AKFHRS................
tr|A0A522CJY0|A0A522CJY0_9SPIR  AGAGEIVRIMNGYQERLGARFAPAKILEDMAGQN....KKFYP.................
tr|A0A2N1RRJ3|A0A2N1RRJ3_9SPIR  AGGDSIIRIMNTLEKEYGGRFRPAKILSDMVKKG....KRFYTV................
tr|A0A2N2KFN7|A0A2N2KFN7_9DELT  IGPARILEMLESLQKTYGGRFHPPQILQDIVRSG....RRFHDE................
tr|A0A1V6AXX9|A0A1V6AXX9_9DELT  VGASSVKATLESLEKTYGNRFAPPQILKDIVQSG....RKFYGE................
tr|A0A2N2HZL0|A0A2N2HZL0_9DELT  LGASSIMATLESLEKNYGKRFTSPQILKDIVQSG....KKFYE.................
tr|A0A5E8ARP3|A0A5E8ARP3_9BACT  RGAKTIVEKMTQLEKEIGARFKPAELLVEQAGNG....KNFY..................
tr|A0A662A7B1|A0A662A7B1_9BACT  EGFENILSLLKSLEEKHGARFKPAGLLEKYINEN....KKFYED................
tr|D7CV16|D7CV16_TRURR          LGPRTVLERLEKLERDHGPRFKPAELLREHAAAD....RPFYADAASEG...........
tr|A0A3C2AKX4|A0A3C2AKX4_9FLAO  LGLQATVDKFNELAKNHGDRFLPPQILLDKAKAG....ETFYS.................
tr|A0A3B8ZLN3|A0A3B8ZLN3_9PLAN  TGANKVVDMFKQLTDKHGPRFKAPQILLDRAAQG....SKFYS.................
tr|A0A345UGV8|A0A345UGV8_9BACT  MGAEVILNQLKTLEQEHGARFTPAKLLEEHAKAG....RKFREG................
tr|A0A6C1P6A2|A0A6C1P6A2_9BACT  EGASNIFTRLDKLEGQHGIRFKPAEILAFMAKKS....GQFYG.................
tr|A0A3M1X9W8|A0A3M1X9W8_9BACT  EGGEAIFNRLRELEQRFGNRFTPAELLAEKVRRG....ESFHRR................
tr|A0A354C796|A0A354C796_9DELT  LGLPKFLSLMEQLAEKLGPRFTPAQILRDGEANN....QRFYT.................
tr|A0A3M1NUM8|A0A3M1NUM8_9BACT  LGVEKVVTILRKLEEKHGNRFTPAPILLDYANNQ....KRFYEEK...............
tr|A0A3M2KRC8|A0A3M2KRC8_9BACT  LGAGAVLSMLEELEQRFGARFTPAQIIREYAAKE....KRFYSE................
tr|A0A5S9IHR0|A0A5S9IHR0_9BACT  LGASKVVATLESLQEKHGVRFTPAPILQEYAKNN....KKFYN.................
tr|A0A6M1ST81|A0A6M1ST81_9BACT  LGADEVVKRMERFEDEIGVRFKPAPILKEYASSN....KKFHND................
tr|A0A521AAE5|A0A521AAE5_9BACT  VGADLIVERLENYQQGLGPRFKPADILKSYASAR....KKFHPDQA..............
tr|A0A1M4ZMD6|A0A1M4ZMD6_9BACT  QGAATIVERLETFRRGLGPRFKPADILRNHASAD....KKFHPD................
tr|A0A2A2GCS2|A0A2A2GCS2_9BACT  EGADTIVGRLEKFEQEHGPRFTPADILKEYAKDD....KKFHQD................
tr|A0A5D3YIH7|A0A5D3YIH7_9BACT  EGADKIVQRLEKFEQEHGPRFTPADILKDYAKSG....KKFHQD................
tr|A0A6M1T9G0|A0A6M1T9G0_9BACT  EGADTIVERLQKYEQKHGPRFTPADILKEHAQSG....EKFHQD................
tr|A0A6A8Q1Y8|A0A6A8Q1Y8_9BACT  EGVKHITDRLKAFAGELGPRFEPAGILVEYAKKG....KKFYNE................
tr|A0A521BVJ3|A0A521BVJ3_9BACT  TGVQKVVDRLEKFEGEFGPRFKSAEILIDYAKDK....RKFYED................
tr|A0A359E0Y8|A0A359E0Y8_9BACT  QGVQNVVDRLNSYVDKFGPRFKPAQILVDYAKEG....KKFHG.................
tr|A0A2D8CIM6|A0A2D8CIM6_9BACT  QGVQNVVDRLNSYVDKFGPRFKPAQILVDYAKEG....KKFHG.................
tr|A0A2D9FWF5|A0A2D9FWF5_9BACT  TGVQNVVDQLNDFTEKFGPRFKPAQILIDHAKEG....KKFYSEDQ..............
tr|A0A1B6YB70|A0A1B6YB70_9BACT  LGADVIVKKLNGFAEEFGPRFKPASILMDKAKKG....ETFHKD................
tr|A0A2D4ZTC0|A0A2D4ZTC0_9BACT  IGADTIVTKLTAFSEEFGPRFKPSEILIQKAESK....DSFH..................
tr|A0A3D4UVW7|A0A3D4UVW7_9BACT  IGADTIVTKLTAFSEEFGPRFKPSEILIQKAESK....DSFH..................
tr|A0A3F3I3M2|A0A3F3I3M2_9BACT  IGADTIVKKLTAFSEEFGPRFKPSDILIQKAESK....DSFH..................
tr|A0A3D1G7L9|A0A3D1G7L9_9BACT  KGAKWVTEKLNEFTDTFGPRFKPAELLADMAKSR....KDFYE.................
tr|A0A3M8G1Y8|A0A3M8G1Y8_9BACT  LGAQQVVDHLNGFAEKFGPRFKPAPILEDYIKNG....KRFHHS................
tr|A0A5Q4F391|A0A5Q4F391_9BACT  EGAASVCSALEKLQSKHGDRFRPAEMLQEMASSG....KAFYD.................
tr|A0A6I7NPV0|A0A6I7NPV0_9BACT  MGIEVAADLLDTLESDHGPRFNPPDRLEDMADNN....ERFYN.................
tr|A0A651G1J2|A0A651G1J2_9BACT  VGLAELTDRMLKLQKTHGDRFRPAALLGEYISDK....KSFYETYTPV............
tr|A0A371QRK5|A0A371QRK5_9BACT  EGSGAIVERMEILQTELGDRFKPAGLLTKHAKNN....TPFHD.................
tr|A0A2N0VGJ5|A0A2N0VGJ5_9BACT  ETPAKMVERMEKLQSKHGDRFKPADLL..RDKIG....NRFHS.................
tr|A0A316TYJ0|A0A316TYJ0_9BACT  EGPDKIIEWMSTLQNDHGDRFKPAGLLIDKNKSG....RRFHPE................
tr|A0A651GLZ8|A0A651GLZ8_9BACT  EGAAAVVKRMEKLQKEHGPRFKPADLLLEKSRKN....QGFH..................
tr|A0A7Y5V3S5|A0A7Y5V3S5_9BACT  TGAARVVSELEDLAARFAPRFSPAELLLSMARRG....ERFYPAEGRPVG..........
tr|A0A7W1SHC0|A0A7W1SHC0_9BACT  QGADEIVERLEGLNGRFPPRFEPAELLVEMARAR....RRFYPASGKPVE..........
tr|A0A7Y5PBT7|A0A7Y5PBT7_9BACT  LGAGRVVDELQALNDRFPGRFEPAGVLVAKARAR....ERFYPAHGHPLP..........
tr|A0A7Y5TXL8|A0A7Y5TXL8_9BACT  LGAGRVVDELQALNDRFPGRFEPAGVLVAKARAR....ERFYPAHGHPLP..........
tr|A0A423PQ96|A0A423PQ96_9GAMM  VGVARFVDRLHALVERHGPRFEPAPILKTMASARSASHGRFYS.................
tr|U2G066|U2G066_9GAMM          FGVGRVVDRLQALAERHGARFEPAPLLKTMAKAQAESHRRFYP.................
tr|A0A2E0J1F4|A0A2E0J1F4_9GAMM  MGVALFVDRLNALAEKHGPRFEPAPILKTMAKATSESHRRFYP.................
tr|A0A2D4SCM8|A0A2D4SCM8_9GAMM  LGAQAVVDKLDKYAGRYGERFKPAPALVEAAKKG....KPFYPEKKATTRRRKAEAS...
tr|L0WJH1|L0WJH1_9GAMM          LGLVNAVRTLERLTEQYGERFKPAPILIDMAKKK....ARFYQD................
tr|A0A1H5XF61|A0A1H5XF61_9GAMM  LGLTNSVRTLERLAEQFGDRFTPAPLLIDMAKKK....QRFYP.................
tr|A0A2E9TS48|A0A2E9TS48_9GAMM  LGLTNSVRTLERLAEQFGERFTPAPLLIEMAKKK....QRFYP.................
tr|A0A095UE19|A0A095UE19_9GAMM  LGLTNSVRTLERLAEQFGERFTPAPLLVEMAKKK....QRFYE.................
tr|A0A7G2S9A0|A0A7G2S9A0_9GAMM  LGLTNSVRTLERLAEQFGERFTPAPLLVEMAKKK....QRFYE.................
tr|B5JU27|B5JU27_9GAMM          LGLQASVQKLETLAERHGEHFQPAQILVDMAKNN....DTFYKN................
tr|A0A1Y0IHP1|A0A1Y0IHP1_9GAMM  LGLDKTLTRLEQLAATYGKRFEPAPIIRDMAAKK....QTFYPAR...............
tr|A0A316FZ14|A0A316FZ14_9GAMM  RGIQTIVERLSFYEKQEGERFKPAEILTRMSEQE....KSFYE.................
tr|A0A498C231|A0A498C231_9GAMM  CGEGNVAARLQALAEAHGPRFTPAPSLQG...QG..........................
tr|Q0A6T4|Q0A6T4_ALKEH          QGAAGVTERLQALAAEHGPRFAPAPSL.....QG..........................
tr|A0A3E0WL10|A0A3E0WL10_9GAMM  RGIGEIVARLQELEKRCGMRFQPAPILVKLAEEK....RGFYQE................
tr|A0A3S1BU16|A0A3S1BU16_9GAMM  LGIASVVDKLQQYQQRLGERFAPAESLLRMAQEK....RTFHPVD...............
tr|A0A7V8QFN9|A0A7V8QFN9_9GAMM  LGLDTVLKKLETHHQNQGERYAPARIISEMAEKG....ERFHKED...............
tr|A0A1H8PPF9|A0A1H8PPF9_9GAMM  RGIPEIVQRLKELQQQHGERFAPAEILERMAAGG....ERFHGD................
tr|A0A6H0J0V7|A0A6H0J0V7_9GAMM  RGIKETVERLRELQGKHGERFAPAPILERMASNG....ETFHTD................
tr|V5EZX6|V5EZX6_9VIBR          YGLDKLIEKMRQFELTYGERFEPCQGLIDRAESG....KTFYG.................
tr|A0A6I1QBM2|A0A6I1QBM2_9VIBR  YGLDKLIEKMRQFELTYGERFEPCQGLIDRAESG....KTFYG.................
tr|A0A511QRL5|A0A511QRL5_9VIBR  YGLENLIERMNQFELKYGERFKPCQGLLDRLESG....EKFYS.................
tr|A0A7Y0JZP4|A0A7Y0JZP4_9VIBR  YGLENLIEKMNQFELKYGERFKPCQGLLDRLESG....EKFYS.................
tr|A0A193KD24|A0A193KD24_9VIBR  YGLENLIEKMNQFELKYGERFKPCQGLLDRLESG....EKFYS.................
tr|A0A1S1HM72|A0A1S1HM72_PROST  LGIQKTVDTMNNLAEKYGEKFRPCDRLCQMAQNN....EQFFSE................
tr|A0A7T8I613|A0A7T8I613_9GAMM  LGIQKTVDTMNNLAEKYDEKFRPCDRLCQMAQNN....EQFFSE................
tr|A0A140NIF0|A0A140NIF0_PROSM  LGIQKTVDTMNQLAERYGDKFRPCERLCQMAQTN....ETFFPANKS.............
tr|A0A379GMB7|A0A379GMB7_PROST  LGIQKTVDTMNQLAERYGDKFRPCERLCQMAQTN....ETFFPANKS.............
tr|A0A379H393|A0A379H393_PROST  LGIQKTVDTMNQLAERYGDKFRPCERLCQMAQTN....ETFFPANKS.............
tr|B2Q0L5|B2Q0L5_PROST          LGIQKTVDTMNQLAERYGDKFRPCERLCQMAQTN....ETFFPANKS.............
tr|A0A6I3JUE9|A0A6I3JUE9_9GAMM  LGIQKTVDTMNNLAERYGDKFRPCELLCKMAEVN....KTFFQ.................
tr|A0A7D4P594|A0A7D4P594_YERMW  LGADNVVKTLRLLAQQYGERFEPCPLLVTMAEQQ....RQFYLPENSENEVSSHPPIESV
tr|A0A7U7IVK6|A0A7U7IVK6_YEREN  LGADKVVKTLNLLAQQYGERFEPCPLLVTMAEQQ....KRFYPPENSLDEAAITAHN...
tr|A0A447RIF5|A0A447RIF5_YEREN  LGADKVVKTLNLLAQQYGERFEPCPLLVTMAEQQ....KRFYPPENSLDEAAITAHN...
tr|A0A7H4ZLV9|A0A7H4ZLV9_YERP4  LGADKVVKTLNLLAQQYGERFEPCPLLVTMAEQQ....KRFYPPENSLDEAAITAHN...
tr|A0A0H3NX46|A0A0H3NX46_YERE1  LGADKVVKTLNLLAQQYGERFEPCPLLVTMAEQQ....KRFYPPENSLDEAAITAHN...
tr|A0A7T9XUV7|A0A7T9XUV7_YEREN  LGADKVVKTLNLLAQQYGERFEPCPLLVTMAEQQ....KRFYPPENSLDEAAITAHN...
tr|A0A7U7IYV8|A0A7U7IYV8_YEREN  LGADKVVKTLNLLAQQYGERFEPCPLLVTMAEQQ....KRFYPPENSLDEAAITAHN...
tr|A0A0H5G7G2|A0A0H5G7G2_YEREN  LGADKVVKTLNLLAQQYGERFEPCPLLVTMAEQQ....KRFYPPENSLDEAAITAHN...
tr|A0A2A7TDI2|A0A2A7TDI2_YERKR  LGAEKVVKTLNLLAQQYGERFEPCPLLVTMAEQQ....QRFYPLEESMTERAVNPDN...
tr|A0A0T9M261|A0A0T9M261_YERKR  LGAEKVVKTLNLLAQQYGERFEPCPLLVTMAEQQ....QRFYPPEESMNERAVNPDN...
tr|A0A0A0CMP1|A0A0A0CMP1_PHOLU  LGCARVVEILRRLESQYGDRFVPCECLVKMAEQN....KSFYP.................
tr|A0A329VG55|A0A329VG55_9GAMM  LGCARVVEILRRLESQYGDRFVPCECLVNMAEQN....KSFYP.................
sp|Q7N288|FADJ_PHOLL            LGCARVVEILRRLESQYGDRFVPCECLVNMAEQN....KSFYP.................
tr|A0A6L9JMX5|A0A6L9JMX5_PHOLM  LGCARVVEILRRLESQYGDRFVPCECLVNMAEQN....KSFYP.................
tr|A0A7X5HQ29|A0A7X5HQ29_PHOLM  LGCARVVEILRRLESQYGDRFVPCECLVNMAEQN....KSFYP.................
tr|A0A1C0U051|A0A1C0U051_9GAMM  LGCARVVEILRKLESQYGDRFVPCERLVKMAEQN....KNFYP.................
tr|A0A7X5QF62|A0A7X5QF62_9GAMM  LGCARVVEILRKLESQYGDRFVPCDRLVKMAEQN....KNFYP.................
```

```
tr|A0A2D0KWM9|A0A2D0KWM9_9GAMM  LGSRHVVETLQRLAQQYGDRFAPCERLIQMAEQD....KRFYE.................
tr|A0A2D0LAI1|A0A2D0LAI1_9GAMM  LGSRHVVETLQRLAQQYGDRFAPCERLIQMAEQD....KRFYE.................
tr|A0A2D0IXC8|A0A2D0IXC8_XENBU  LGSNKVVEILRRLEQQYGERFAPCERLVQMAEQN....KKFYE.................
tr|W1JAM5|W1JAM5_9GAMM          LGSNKVVEILRRLEQQHGERFAPCERLVQMAEQN....EKFYE.................
tr|A0A3D9UED3|A0A3D9UED3_9GAMM  LGSNKVVEILRRLEQQHGERFAPCERLVQMAEQN....EKFYE.................
tr|A0A1I3JCA9|A0A1I3JCA9_9GAMM  LGSDKVVEILRRLEAKHGERFAPCELLVQLAEQK....KKFYD.................
tr|A0A068QUL9|A0A068QUL9_9GAMM  LGSDKVVEILRRLEAQYGDRFTPCEYLVQMAEQK....KKFYA.................
tr|A0A0M0TCH6|A0A0M0TCH6_9GAMM  LGSDKVVEILRRLETQYGERFTPCEGLIRMAEQK....KKFHV.................
tr|A0A1Q5U854|A0A1Q5U854_9GAMM  LGSDKVVEILRRLEGQYGERFAPCGRLVQMAEQK....KKFHA.................
tr|A0A2D0ISP1|A0A2D0ISP1_9GAMM  LGSDKVVEILRRLEAQYGERFAPCERLIQMAEQK....NKFHA.................
tr|A0A1Q5TUI8|A0A1Q5TUI8_9GAMM  LGSDKVVEVLRRLEAQHGERFAPCERLVQMAEQK....KKFHA.................
tr|A0A2D0KJI7|A0A2D0KJI7_9GAMM  LGSDKVVETLRRLVPQYGERFTPCERLVQMAEQK....KKFYA.................
tr|A0A1Y2SB97|A0A1Y2SB97_9GAMM  LGSDKVVEILRRLEQQYGERFAPCERLIQMAEQK....KKFHV.................
tr|A0A1I5DWI7|A0A1I5DWI7_9GAMM  LGSDKIVEILRRLESQYGERFAPCEHLVQMVEQK....KKFHA.................
tr|A0A1I7GWY6|A0A1I7GWY6_9GAMM  LGSDKVVGVLRRLESKYGEKFTPCERLIQMAEQK....KKFHT.................
tr|D3VKY8|D3VKY8_XENNA          LGSDKVVGILRHLESQYGERFTPCEGLIRMAEQK....KKFYS.................
tr|A0A2G0Q6Q9|A0A2G0Q6Q9_9GAMM  LGSDKVVEILRRLESLHGEKFAPCERLIQMAEKK....EKFYE.................
tr|A0A2D0JU24|A0A2D0JU24_9GAMM  LGIDKVIETLRRLEQQHGERFTPCEGLIQMAEQK....KKFHA.................
tr|A0A0J5FN38|A0A0J5FN38_9GAMM  LGIDKVVETLCRLEQQHGERFTPCEGLIQMAEQK....KKFHS.................
tr|A0A432XLD4|A0A432XLD4_9GAMM  VGVTEIVKRLQGYQQQHGERYTPAPLLLKMAENN....ESFY..................
tr|A0A2D8HU84|A0A2D8HU84_9GAMM  QGLETIVNKLEKLASERGERYTPAPLLKQMVENG....WSFYQ.................
tr|A0A656X1Q2|A0A656X1Q2_9GAMM  QGLETIVNKLEKLASERGERYTPAPLLKQMVENG....WSFYQ.................
tr|A0A4Q1QH79|A0A4Q1QH79_9GAMM  QGIGSIVAKLEALAGERGERYTPAPLLKEMAKNG....ERFYS.................
tr|A0A1G7LQE6|A0A1G7LQE6_9GAMM  QGIGSIVAKLEALASERGERYTPAPLLKEMAKNG....ERFYS.................
tr|A0A432YVG7|A0A432YVG7_9GAMM  QGIGSIVAKLEALASERGERYTPAPLLKEMAKNG....ERFYS.................
tr|A0A1J5N0P5|A0A1J5N0P5_9GAMM  LGVSTVVSLLQGYEKQHGERFTPSNLLVEMSENN....KTFY..................
tr|A0A0D8D548|A0A0D8D548_9GAMM  VGATNVVSQLNRWAAQHGERFKPCDALVKMAEEG....RTCYE.................
tr|A0A3E0TNM2|A0A3E0TNM2_9GAMM  VGAVNIVNQLNQWAQQHGERYKPCQALVDMAEQG....TTCY..................
tr|A0A3E0UD67|A0A3E0UD67_9GAMM  VGAVNIVNQLNQWAQQHGERYKPCQALVDMAEQG....TTCY..................
tr|A0A3E0U0P4|A0A3E0U0P4_9GAMM  VGAVNIVNQLNQWAQQHGDRYKPCQALVDMAEQG....TTCY..................
tr|A0A0M2V8B1|A0A0M2V8B1_9GAMM  LGVTNLVATLRQLQAEHGERFAPCQRLLTMAEHN....SRFYTE................
tr|A0A285ITY5|A0A285ITY5_9GAMM  LGVTNLVASLRQLQAEHGERFAPCPRLLAMAEQN....SRFYQ.................
tr|A0A486XTB9|A0A486XTB9_9GAMM  LGADNLVAQLRNLQSRLGDRFTPAALLVSMAEQQ....QRFYP.................
tr|I1E1G1|I1E1G1_9GAMM          LGADKLVAQLRNLESRFGSRFAPAHLLVQMAEQQ....QRFYP.................
tr|A0A1H6KJ68|A0A1H6KJ68_9GAMM  LGAANLVAQLRNLESRFGSRFAPAPLLVTMAEQQ....QRFYN.................
tr|A0A0X3Y764|A0A0X3Y764_9GAMM  LGAADLVAQLRNLESRFGSRFAPAPLLVTMAEQQ....QRFYN.................
tr|A0A2N1YEV4|A0A2N1YEV4_9GAMM  LGAANLVAQLRNLESRFGSRFAPAPLLVTMAEQQ....QRFYN.................
tr|A0A3P3QCM3|A0A3P3QCM3_9GAMM  MGAATLVAHLRSFESRFGARFAPSKLLLEKAEQN....ASFY..................
tr|F7NT72|F7NT72_9GAMM          MGAATLVAHLRSFESRFGARFAPSKLLLEKAEQN....TTFY..................
tr|A0A3S2TW63|A0A3S2TW63_9GAMM  MGAATLVAHLRSFESRFGARFAPSKLLLEKAEQN....TTFY..................
tr|A0A3D5BGW7|A0A3D5BGW7_9GAMM  LGATTLVADLRRYEAQFGSRFAPSALLLQMAEQG....KTFY..................
tr|A0A5C7TI17|A0A5C7TI17_9GAMM  LGAGNLVADLRRFEAQFGARFAPAQLLLSMAEQD....KTFY..................
tr|A0A0U4W982|A0A0U4W982_9GAMM  LGAGNLVADLRCYEAQFGSRFAPAKMLLELAEQE....KTFY..................
tr|A0A2I0FCM2|A0A2I0FCM2_9GAMM  IGIAELVDKLERHQDKYGERFAPCDLLKAMAKEG....QKFYN.................
tr|A0A2G2IVL1|A0A2G2IVL1_9GAMM  IGIAELVDKLERHQDKYGERFAPCESLKAMAKEG....KKFYN.................
tr|A6FI21|A6FI21_9GAMM          IGIAELVDKLERHQDKYGERFAPCESLKAMAKEG....KKFYN.................
tr|A0A4U1BNR2|A0A4U1BNR2_9GAMM  LGAKQLVAILERYQSLHGDRFEPCERLKAMAAED....LRFYE.................
tr|E1SQ60|E1SQ60_FERBD          LGAKSLVERLRSYQERFGDRFAPADKLVAMAEQD....ERFYP.................
tr|A0A4Y6IZ09|A0A4Y6IZ09_9GAMM  LGAKHLVETLKRYQSEFGDRFTPCDRLVEMAESG....AKFYD.................
tr|A0A0C3QSZ7|A0A0C3QSZ7_9GAMM  LGAKHLVETLKRYQDQFGERFTPCDRLVEMAESG....SKFYD.................
tr|A0A6L7HXW0|A0A6L7HXW0_9GAMM  LGAKHLVETLKRYQDQFGERFAPCDRLVEMAESG....SKFYE.................
sp|A3QFP3|FADJ_SHELP            LGAKHLVETLKRYQDQFGDRFAPCDRLVEMAESG....SKFYE.................
tr|A0A1E5IXH7|A0A1E5IXH7_SHECO  MGPAKLVEILNQYAQDHGERFKPCAMLVEMAETG....SRFHS.................
tr|A0A411PKQ0|A0A411PKQ0_9GAMM  LGAANLVATLERYQSMYGERFAPAQMLKDMAAEG....KSFYS.................
tr|A0A6G9QKM3|A0A6G9QKM3_9GAMM  LGAKNLVDTLTRYQKLYGDRFTPCEMLKTMAADG....SSFHAK................
tr|A0A6P1UL63|A0A6P1UL63_9GAMM  LGASNLVATLQGYQSLYGDRFAPCDTLVKMASDG....SQFYKK................
tr|A0A2N1ERI9|A0A2N1ERI9_9GAMM  LGASNLVATLQGYQSLYGDRFAPCDTLVKMASDG....SQFYKK................
tr|A0A7W4FU55|A0A7W4FU55_9GAMM  LGASNLVATLQGYQSLYGDRFAPCDTLVKMASDG....SQFYKK................
sp|Q8ECP7|FADJ_SHEON            LGADNLVKILERYQTQYGDRFEPCQRLKAMAAEK....ARFF..................
tr|A0A501XZY8|A0A501XZY8_9GAMM  LGADNLVKILERYQTQYGDRFEPCPRLKAMAAEK....ARFF..................
tr|A0A2W5DCZ0|A0A2W5DCZ0_SHEOE  LGADNLVKILERYQTQYGDRFEPCPRLKAMAAEK....ARFF..................
tr|A0A1E3V3C8|A0A1E3V3C8_9GAMM  LGADNLVKILERYQTQYGDRFEPCPRLKGMAAEK....ARFF..................
tr|A0A1Z4AI20|A0A1Z4AI20_9GAMM  LGADNLVKILERYQTQYGDRFEPCPRLKAMAAEK....ARFF..................
tr|A0A7X9LJL9|A0A7X9LJL9_9GAMM  LGADNLVKILERYQTQYGDRFEPCPRLKAMAAEK....ARFF..................
tr|A0A073KMY5|A0A073KMY5_9GAMM  LGADNLVKILERYQTQYGDRFEPCPRLKAMAAEK....ARFF..................
sp|A0KV76|FADJ_SHESA            LGADNLVNILERYQAQYGDRFEPCPRLKAMAAEK....ARFF..................
tr|A0A220UTH7|A0A220UTH7_9GAMM  LGADNLVNILERYQAQYGDRFEPCPRLKAMAAEK....ARFF..................
tr|A0A5B8R6W1|A0A5B8R6W1_9GAMM  LGADNLVNILERYQAQYGDRFEPCPRLKAMAAEK....TRFF..................
tr|V1DAI4|V1DAI4_9GAMM          LGADNLVNILERYQAQYGDRFEPCPRLKAMAAEK....TRFF..................
tr|A0A448CPQ4|A0A448CPQ4_SHEPU  LGADNLVSILVRYQAQYGDRFEPCPRLKAMAAEK....TRFF..................
tr|A0A252ERQ3|A0A252ERQ3_SHEPU  LGADNLVSILERYQAQYGDRFEPCPRLKAMAAEK....TRFF..................
sp|Q0HKD1|FADJ_SHESM            LGADNLVKILERYQAQYGDRFEPCPRLKEMAAEK....TRFF..................
sp|Q0HWN3|FADJ_SHESR            LGADNLVKILERYQAQYGDRFEPCPRLKEMAAEK....TRFF..................
tr|F7RQE3|F7RQE3_9GAMM          LGAANLVRILEGYQSQLGDRFEPCERLKAMAQEN....RRFF..................
tr|B8EE98|B8EE98_SHEB2          LGAANLVNILESYQSQFGNRFEPCERLKTMAQEN....AHFF..................
sp|A6WQ25|FADJ_SHEB8            LGAANLVKILESYQSQFGNRFEPCERLKTMAQEN....AHFF..................
tr|A0A448EK41|A0A448EK41_9GAMM  LGAANLVQILEGYQSQFGNRFEPCERLKTMAQEN....AHFF..................
tr|A0A553JHX1|A0A553JHX1_SHEHA  LGAKDLVSKLEAYQGVYGDRFKPCNQLVEMASSG....AKFFS.................
tr|B8CPY6|B8CPY6_SHEPW          LGPAKLVEILSGYQSQFGDRFAPAELLKQMANEQ....SYFFK.................
tr|A0A431WFC4|A0A431WFC4_9GAMM  MGVAELVAELESYQAQYGDRFMPCARLKEMAENG....QTFFE.................
tr|A8FTR7|A8FTR7_SHESH          MGAAELVAVLETYQAKFGDRFTPCDRLKEMAENG....LSFFE.................
tr|A0A431WNL0|A0A431WNL0_9GAMM  MGAAELVAVLETYQAKYGDRFTPCDRLKEMAEKG....LSFFE.................
tr|A0A550AEC5|A0A550AEC5_9GAMM  LGAGALVEKLEKYQSRFGDRFVPCSKLKEMAENN....QRFFD.................
tr|A0A7L4WW90|A0A7L4WW90_9GAMM  LGAGALVEKLEKYQSRFGDRFVPCSKLKEMAENN....QRFFD.................
tr|A9DDU3|A9DDU3_9GAMM          LGAGVLVAKLEKYQAKHGDRFTPCDRLQEMAATG....RRFF..................
tr|A0A330M2B9|A0A330M2B9_9GAMM  LGAGVLVAKLETYQAKHGDRFAACDKLKQMAETN....QRFFD.................
tr|A0A5N8UFC7|A0A5N8UFC7_9GAMM  LGAGVLVAKLEKYQAKHGNRFAPCDKLREMAETN....QRFFD.................
tr|A0A1S6HN57|A0A1S6HN57_9GAMM  LGAGVLVAKLEKYQAKHGDRFAPCEKLKEMAESN....QHFFD.................
tr|D4ZMH7|D4ZMH7_SHEVD          LGAGVLVAKLEKYQAKHGDRFTPCNKLIEMAESN....QRFFD.................
tr|A0A3L8Q213|A0A3L8Q213_9GAMM  MGADKLVTLLKGYQATHGDRFEPCELLVKMAEEG....KKFYS.................
tr|A0A3A6U4N9|A0A3A6U4N9_9GAMM  LGAKALVETLQKYQNEFGDRFTPCDLLVKMAQEE....RSFYE.................
tr|A0A4Q5MA37|A0A4Q5MA37_9GAMM  LGVTNLVDMLKGYQSKFGDRFAPCDLLLKMAEEQ....KSFY..................
tr|A0A1L6LSX5|A0A1L6LSX5_9DELT  IGAAEILKRIQGYEQQHGKRWTPAPVLVDMAKSG....KKFYP.................
tr|A0A2W4L9B9|A0A2W4L9B9_9PROT  LGPQEVVRRLRIYEERIGHRFRPAQLLVDMAREG....RRFFSD................
tr|A0A2W4M4E6|A0A2W4M4E6_9PROT  VGAREVVERLETYAAQFGSRFEPAPLLTDMARRG....TTFYGMRRTETVNAQATATL..
tr|A0A6I2GRX9|A0A6I2GRX9_9DELT  FGADKLLQRLEHLQEKHGVRFTPAPLLVELARAG....GRFYPAGEG.............
tr|A0A0H4WMK2|A0A0H4WMK2_9DELT  RGPAEVLRKLEHFHDKLGERFIPAPLLVEMVKAG....KTFYPR................
tr|F8CJ36|F8CJ36_MYXFH          RGPADVLRKLEHFHDKLGERFVPAPHLVEMVKAG....KTFYPR................
tr|A0A250K0F1|A0A250K0F1_9DELT  RGPADVLRKLEHFHDKLGERFVPAPHLVEMVKAG....KTFYPR................
tr|A0A7Y6WFZ2|A0A7Y6WFZ2_9DELT  RGPAEVLRKLEHYHDKLGERFAPAPHLVEMVKAG....KTFYPR................
tr|A0A7Y7C660|A0A7Y7C660_9DELT  RGPAEVLRKLEHYHDKLGERFAPAPHLVELVKAG....KTFYPR................
tr|A0A7Y4JFH2|A0A7Y4JFH2_MYXXA  RGPAEVLRKLEHYHDKLGERFAPAPHLVEMVKAG....KTFYPR................
tr|A0A4Y6CZQ9|A0A4Y6CZQ9_MYXXA  RGPAEVLRKLEHYHDKLGERFAPAPHLVEMVKAG....KTFYPR................
```

```
tr|A0A7Y4IKV5|A0A7Y4IKV5_MYXXA  RGPAEVLRKLEHYHDKLGERFAPAPHLVEMVKAG....KTFYPR................
tr|A0A511HHB0|A0A511HHB0_9DELT  RGPAEVLRKLEHYHDKLGERFAPAPHLVEMVKAG....KTFYPR................
tr|A0A4Y6CKY7|A0A4Y6CKY7_MYXXA  RGPAEVLRKLEHYHDKLGERFAPAPHLVEMVKAG....KTFYPR................
tr|Q1D1F2|Q1D1F2_MYXXD          RGPAEVLRKLEHYHDKLGERFAPAPHLVEMVKAG....KTFYPR................
tr|A0A7Y4MA14|A0A7Y4MA14_MYXXA  RGPAEVLRKLEHYHDKLGERFAPAPHLVEMVKAG....KTFYPR................
tr|A0A7T8Y4N9|A0A7T8Y4N9_MYXXA  RGPAEVLRKLEHYHDKLGERFAPAPHLVEMVKAG....KTFYPR................
tr|L7UE67|L7UE67_MYXSD          VGAAELLRKLEHYQDKLGERFTPAPLLEEHVKAG....KTFYPR................
tr|A0A511T9X1|A0A511T9X1_MYXFU  RGLSEVLRKLEHYQDKLGERFTPAPLLVEMVKEG....KTFYPR................
tr|A0A7Y7C9C4|A0A7Y7C9C4_9DELT  RGPAEVLRKLEHYQDKLGERFTPAPLLVEMVKAG....KTFYPR................
tr|A0A540X7W8|A0A540X7W8_9DELT  MGPAELLRKLEHYQDKLGERFTPAPLLVEMVKAG....KTFYPR................
tr|A0A3A5FK19|A0A3A5FK19_9DELT  LGPAQLLRKLEHYQDKFGERFTPAPFLIEQVRAG....KSFHGG................
tr|A0A3A8JQL9|A0A3A8JQL9_9DELT  LGPSNLLRKLEHYQDKYGERFTPAPLLVEKVRAG....KGFYEA................
tr|A0A7Y4NFA4|A0A7Y4NFA4_9DELT  LGPANLLRKLEHYQDKYGERFTPAPLLVEKVRAG....KGFHEA................
tr|A0A3A8HDQ7|A0A3A8HDQ7_9DELT  LGPANLLRKLEHYQDKYGERFTPAPLLVEKVRAG....KGFYEA................
tr|A0A3A8GR90|A0A3A8GR90_9DELT  LGPANLLRKLEHYQDKYGERFTPAPLLVEKVRAS....KGFYEA................
tr|A0A3A8SBD7|A0A3A8SBD7_9DELT  LGPANLLRKLEHYQDKYGERFTPAPLLVEKVRAN....KGFYES................
tr|A0A3A8T0I8|A0A3A8T0I8_9DELT  LGPANLLRKLEHYQDKYGERFTPAPLLVEKVRAN....KGFYES................
tr|A0A7X5BU07|A0A7X5BU07_9DELT  LGPANLLRKLEHYQDKYGERFTPAPLLVEKVRAN....KGFYES................
tr|A0A7Y1RVL2|A0A7Y1RVL2_9DELT  LGPANLLRKLEHYQDKYGERFTPAPLLVEKVRAN....KGFYES................
tr|A0A3A8THN2|A0A3A8THN2_9DELT  LGPANLLRKLEHYQDKYGERFTPAPLLVEKVRAN....KGFYES................
tr|A0A7Y1RX76|A0A7Y1RX76_9DELT  LGPANLLRKLEHYQDKYGERFTPAPLLVEKVRAN....KGFYES................
tr|A0A3A8RA58|A0A3A8RA58_9DELT  LGPANLLRKLEHYQDKYGERFTPAPLLVEKVRAN....KGFYES................
tr|A0A410RPB6|A0A410RPB6_CORCK  LGPANLLRKLEHYHDKYGERFTPAPLLVEKVRAG....KGFYES................
tr|A0A7Y4J474|A0A7Y4J474_CORCK  LGPANLLRKLEHYQDKYGERFTPAPLLVEKVRDG....KGFYEA................
tr|A0A3A8I9Z6|A0A3A8I9Z6_9DELT  LGPANLLRKLEHYQDKYGERFTPAPLLVEKVRDG....KGFYES................
tr|H8MKE9|H8MKE9_CORCM          LGPANLLRKLEHYQDKYGERFTPAPLLVEKVRAG....KGFYES................
tr|A0A3A8H102|A0A3A8H102_9DELT  LGPANLLRKLEHYQDKYGERFTPAPLLVEKVRAG....KGFYES................
tr|A0A554FW33|A0A554FW33_9DELT  LGPANLLRKLEHYQDKYGERFTPAPLLVEKVKAG....KGFYEA................
tr|A0A3A8NPC0|A0A3A8NPC0_9DELT  LGPANLLRKLEHYQDKYGERFTPAPLLVEKVKAG....KGFYEA................
tr|A0A3A8JUV3|A0A3A8JUV3_9DELT  LGLDNLLRKLEHYQDKYGERFTPAPLLVEKVKAG....KGFYEA................
tr|A0A3A8NEX6|A0A3A8NEX6_9DELT  LGPANLLRKLEHYQDKYGERFTPAPLLLEKVKAG....KGFYEA................
tr|A0A3A8JEU8|A0A3A8JEU8_9DELT  LGPAHLLRKLEHYQDKYGERFTPAPLLVEKVRTG....KGFYDA................
tr|A0A3A8LI76|A0A3A8LI76_9DELT  LGPAHLLRKLEHYQDKYGERFTPAPLLLEKVRAG....KGFYDA................
tr|A0A085WXN8|A0A085WXN8_9DELT  LTPAVLLKRLEHYQDKYGERFTPAPHLVDVVNAG....KTFHSR................
tr|A0A2T4V0M5|A0A2T4V0M5_9DELT  IGPAELLRRLEHYQDKYGERFTPAPSLVEMVKAG....KTFYPR................
tr|A0A0G2ZSW5|A0A0G2ZSW5_9DELT  LGPAELLRRLEHFQDKYGERFTPAPSLVDMVKAG....KTFYPR................
tr|A0A3M2DKY0|A0A3M2DKY0_9DELT  LGIAALVDQLDRYAGEYGPRWAPAPVLRDMAAAG....QTFHGPSAAPPRAATRRKSA..
tr|A0A661NQ58|A0A661NQ58_9DELT  VGAKQVLERVEHYRDRYGARFEPAPLLVEKARGG....ATFY..................
tr|A0A520YD99|A0A520YD99_9DELT  VGAKEILGRLRELEKQLGPRFSPAPVLEEIARSG....QTFHGSDPVQPGQSRGNQAPSS
tr|A0A7Y3BRE4|A0A7Y3BRE4_9DELT  VGAKEILGRLRELEKQLGPRFSPAPVLEEIARSG....QTFHGSDPVQPGQSRGNQAPSS
tr|A0A2D9TF90|A0A2D9TF90_9DELT  HGPDAIVKRMQAFEKQYGSRFAPAPLLLAMAAQG....LTFHGAGAVQPGQDVPQTVIDT
tr|A0A2E0TP32|A0A2E0TP32_9DELT  VGAREVVRKLQNFEKRVGPRFAPAPILLAMAEKN....LTFHGENTVTPGEIPT......
tr|A0A2E4Y3V1|A0A2E4Y3V1_9PROT  VGAQSFLNDMKKFEDRYGERFKVATIIEDYAKEN....KKFHG.................
tr|A0A2E6VRH4|A0A2E6VRH4_9DELT  EGVSEVVRQLKTLEDEFGARYTPAQMLVDMAKKG....ERFHPA................
tr|A0A1F9FB59|A0A1F9FB59_9DELT  VGASTVVSTLERFAARYGKRFKPAELLVDQAKSG....KRFHAKG...............
```

ECHA_HUMAN

```
ECHA_HUMAN                                 . . . . .
tr|A0A4R6XLA8|A0A4R6XLA8_9GAMM             . . . . .
tr|A0A6A0IJT2|A0A6A0IJT2_9BACT             . . . . .
tr|A0A7V3QZU7|A0A7V3QZU7_9BACT             . . . . .
tr|A0A7V4SYJ6|A0A7V4SYJ6_9BACT             . . . . .
tr|A0A2A8D2N6|A0A2A8D2N6_9BACT             . . . . .
tr|A0A2N1TGP0|A0A2N1TGP0_9SPIR             . . . . .
tr|A0A1G3QGW4|A0A1G3QGW4_9SPIR             . . . . .
tr|A0A1G3QTY3|A0A1G3QTY3_9SPIR             . . . . .
tr|A0A7X9L359|A0A7X9L359_9DELT             . . . . .
tr|A0A522CJY0|A0A522CJY0_9SPIR             . . . . .
tr|A0A2N1RRJ3|A0A2N1RRJ3_9SPIR             . . . . .
tr|A0A2N2KFN7|A0A2N2KFN7_9DELT             . . . . .
tr|A0A1V6AXX9|A0A1V6AXX9_9DELT             . . . . .
tr|A0A2N2HZL0|A0A2N2HZL0_9DELT             . . . . .
tr|A0A5E8ARP3|A0A5E8ARP3_9BACT             . . . . .
tr|A0A662A7B1|A0A662A7B1_9BACT             . . . . .
tr|D7CV16|D7CV16_TRURR                     . . . . .
tr|A0A3C2AKX4|A0A3C2AKX4_9FLAO             . . . . .
tr|A0A3B8ZLN3|A0A3B8ZLN3_9PLAN             . . . . .
tr|A0A345UGV8|A0A345UGV8_9BACT             . . . . .
tr|A0A6C1P6A2|A0A6C1P6A2_9BACT             . . . . .
tr|A0A3M1X9W8|A0A3M1X9W8_9BACT             . . . . .
tr|A0A354C796|A0A354C796_9DELT             . . . . .
tr|A0A3M1NUM8|A0A3M1NUM8_9BACT             . . . . .
tr|A0A3M2KRC8|A0A3M2KRC8_9BACT             . . . . .
tr|A0A5S9IHR0|A0A5S9IHR0_9BACT             . . . . .
tr|A0A6M1ST81|A0A6M1ST81_9BACT             . . . . .
tr|A0A521AAE5|A0A521AAE5_9BACT             . . . . .
tr|A0A1M4ZMD6|A0A1M4ZMD6_9BACT             . . . . .
tr|A0A2A2GCS2|A0A2A2GCS2_9BACT             . . . . .
tr|A0A5D3YIH7|A0A5D3YIH7_9BACT             . . . . .
tr|A0A6M1T9G0|A0A6M1T9G0_9BACT             . . . . .
tr|A0A6A8Q1Y8|A0A6A8Q1Y8_9BACT             . . . . .
tr|A0A521BVJ3|A0A521BVJ3_9BACT             . . . . .
tr|A0A359E0Y8|A0A359E0Y8_9BACT             . . . . .
tr|A0A2D8CIM6|A0A2D8CIM6_9BACT             . . . . .
tr|A0A2D9FWF5|A0A2D9FWF5_9BACT             . . . . .
tr|A0A1B6YB70|A0A1B6YB70_9BACT             . . . . .
tr|A0A2D4ZTC0|A0A2D4ZTC0_9BACT             . . . . .
tr|A0A3D4UVW7|A0A3D4UVW7_9BACT             . . . . .
tr|A0A3F3I3M2|A0A3F3I3M2_9BACT             . . . . .
tr|A0A3D1G7L9|A0A3D1G7L9_9BACT             . . . . .
tr|A0A3M8G1Y8|A0A3M8G1Y8_9BACT             . . . . .
tr|A0A5Q4F391|A0A5Q4F391_9BACT             . . . . .
tr|A0A6I7NPV0|A0A6I7NPV0_9BACT             . . . . .
tr|A0A651G1J2|A0A651G1J2_9BACT             . . . . .
tr|A0A371QRK5|A0A371QRK5_9BACT             . . . . .
tr|A0A2N0VGJ5|A0A2N0VGJ5_9BACT             . . . . .
tr|A0A316TYJ0|A0A316TYJ0_9BACT             . . . . .
tr|A0A651GLZ8|A0A651GLZ8_9BACT             . . . . .
tr|A0A7Y5V3S5|A0A7Y5V3S5_9BACT             . . . . .
tr|A0A7W1SHC0|A0A7W1SHC0_9BACT             . . . . .
tr|A0A7Y5PBT7|A0A7Y5PBT7_9BACT             . . . . .
tr|A0A7Y5TXL8|A0A7Y5TXL8_9BACT             . . . . .
tr|A0A423PQ96|A0A423PQ96_9GAMM             . . . . .
tr|U2G066|U2G066_9GAMM                     . . . . .
tr|A0A2E0J1F4|A0A2E0J1F4_9GAMM             . . . . .
tr|A0A2D4SCM8|A0A2D4SCM8_9GAMM             . . . . .
tr|L0WJH1|L0WJH1_9GAMM                     . . . . .
tr|A0A1H5XF61|A0A1H5XF61_9GAMM             . . . . .
tr|A0A2E9TS48|A0A2E9TS48_9GAMM             . . . . .
tr|A0A095UE19|A0A095UE19_9GAMM             . . . . .
tr|A0A7G2S9A0|A0A7G2S9A0_9GAMM             . . . . .
tr|B5JU27|B5JU27_9GAMM                     . . . . .
tr|A0A1Y0IHP1|A0A1Y0IHP1_9GAMM             . . . . .
tr|A0A316FZ14|A0A316FZ14_9GAMM             . . . . .
tr|A0A498C231|A0A498C231_9GAMM             . . . . .
tr|Q0A6T4|Q0A6T4_ALKEH                     . . . . .
tr|A0A3E0WL10|A0A3E0WL10_9GAMM             . . . . .
tr|A0A3S1BU16|A0A3S1BU16_9GAMM             . . . . .
tr|A0A7V8QFN9|A0A7V8QFN9_9GAMM             . . . . .
tr|A0A1H8PPF9|A0A1H8PPF9_9GAMM             . . . . .
tr|A0A6H0J0V7|A0A6H0J0V7_9GAMM             . . . . .
tr|V5EZX6|V5EZX6_9VIBR                     . . . . .
tr|A0A6I1QBM2|A0A6I1QBM2_9VIBR             . . . . .
tr|A0A511QRL5|A0A511QRL5_9VIBR             . . . . .
tr|A0A7Y0JZP4|A0A7Y0JZP4_9VIBR             . . . . .
tr|A0A193KD24|A0A193KD24_9VIBR             . . . . .
tr|A0A1S1HM72|A0A1S1HM72_PROST             . . . . .
tr|A0A7T8I613|A0A7T8I613_9GAMM             . . . . .
tr|A0A140NIF0|A0A140NIF0_PROSM             . . . . .
tr|A0A379GMB7|A0A379GMB7_PROST             . . . . .
tr|A0A379H393|A0A379H393_PROST             . . . . .
tr|B2Q0L5|B2Q0L5_PROST                     . . . . .
tr|A0A6I3JUE9|A0A6I3JUE9_9GAMM             . . . . .
tr|A0A7D4P594|A0A7D4P594_YERMW             S R I T T
tr|A0A7U7IVK6|A0A7U7IVK6_YEREN             . . . . .
tr|A0A447RIF5|A0A447RIF5_YEREN             . . . . .
tr|A0A7H4ZLV9|A0A7H4ZLV9_YERP4             . . . . .
tr|A0A0H3NX46|A0A0H3NX46_YERE1             . . . . .
tr|A0A7T9XUV7|A0A7T9XUV7_YEREN             . . . . .
tr|A0A7U7IYV8|A0A7U7IYV8_YEREN             . . . . .
tr|A0A0H5G7G2|A0A0H5G7G2_YEREN             . . . . .
tr|A0A2A7TDI2|A0A2A7TDI2_YERKR             . . . . .
tr|A0A0T9M261|A0A0T9M261_YERKR             . . . . .
tr|A0A0A0CMP1|A0A0A0CMP1_PHOLU             . . . . .
tr|A0A329VG55|A0A329VG55_9GAMM             . . . . .
sp|Q7N288|FADJ_PHOLL                       . . . . .
tr|A0A6L9JMX5|A0A6L9JMX5_PHOLM             . . . . .
tr|A0A7X5HQ29|A0A7X5HQ29_PHOLM             . . . . .
tr|A0A1C0U051|A0A1C0U051_9GAMM             . . . . .
tr|A0A7X5QF62|A0A7X5QF62_9GAMM             . . . . .
```

```
tr|A0A2D0KWM9|A0A2D0KWM9_9GAMM  .....
tr|A0A2D0LAI1|A0A2D0LAI1_9GAMM  .....
tr|A0A2D0IXC8|A0A2D0IXC8_XENBU  .....
tr|W1JAM5|W1JAM5_9GAMM          .....
tr|A0A3D9UED3|A0A3D9UED3_9GAMM  .....
tr|A0A1I3JCA9|A0A1I3JCA9_9GAMM  .....
tr|A0A068QUL9|A0A068QUL9_9GAMM  .....
tr|A0A0M0TCH6|A0A0M0TCH6_9GAMM  .....
tr|A0A1Q5U854|A0A1Q5U854_9GAMM  .....
tr|A0A2D0ISP1|A0A2D0ISP1_9GAMM  .....
tr|A0A1Q5TUI8|A0A1Q5TUI8_9GAMM  .....
tr|A0A2D0KJI7|A0A2D0KJI7_9GAMM  .....
tr|A0A1Y2SB97|A0A1Y2SB97_9GAMM  .....
tr|A0A1I5DWI7|A0A1I5DWI7_9GAMM  .....
tr|A0A1I7GWY6|A0A1I7GWY6_9GAMM  .....
tr|D3VKY8|D3VKY8_XENNA          .....
tr|A0A2G0Q6Q9|A0A2G0Q6Q9_9GAMM  .....
tr|A0A2D0JU24|A0A2D0JU24_9GAMM  .....
tr|A0A0J5FN38|A0A0J5FN38_9GAMM  .....
tr|A0A432XLD4|A0A432XLD4_9GAMM  .....
tr|A0A2D8HU84|A0A2D8HU84_9GAMM  .....
tr|A0A656X1Q2|A0A656X1Q2_9GAMM  .....
tr|A0A4Q1QH79|A0A4Q1QH79_9GAMM  .....
tr|A0A1G7LQE6|A0A1G7LQE6_9GAMM  .....
tr|A0A432YVG7|A0A432YVG7_9GAMM  .....
tr|A0A1J5N0P5|A0A1J5N0P5_9GAMM  .....
tr|A0A0D8D548|A0A0D8D548_9GAMM  .....
tr|A0A3E0TNM2|A0A3E0TNM2_9GAMM  .....
tr|A0A3E0UD67|A0A3E0UD67_9GAMM  .....
tr|A0A3E0U0P4|A0A3E0U0P4_9GAMM  .....
tr|A0A0M2V8B1|A0A0M2V8B1_9GAMM  .....
tr|A0A285ITY5|A0A285ITY5_9GAMM  .....
tr|A0A486XTB9|A0A486XTB9_9GAMM  .....
tr|I1E1G1|I1E1G1_9GAMM          .....
tr|A0A1H6KJ68|A0A1H6KJ68_9GAMM  .....
tr|A0A0X3Y764|A0A0X3Y764_9GAMM  .....
tr|A0A2N1YEV4|A0A2N1YEV4_9GAMM  .....
tr|A0A3P3QCM3|A0A3P3QCM3_9GAMM  .....
tr|F7NT72|F7NT72_9GAMM          .....
tr|A0A3S2TW63|A0A3S2TW63_9GAMM  .....
tr|A0A3D5BGW7|A0A3D5BGW7_9GAMM  .....
tr|A0A5C7TI17|A0A5C7TI17_9GAMM  .....
tr|A0A0U4W982|A0A0U4W982_9GAMM  .....
tr|A0A2I0FCM2|A0A2I0FCM2_9GAMM  .....
tr|A0A2G2IVL1|A0A2G2IVL1_9GAMM  .....
tr|A6FI21|A6FI21_9GAMM          .....
tr|A0A4U1BNR2|A0A4U1BNR2_9GAMM  .....
tr|E1SQ60|E1SQ60_FERBD          .....
tr|A0A4Y6IZ09|A0A4Y6IZ09_9GAMM  .....
tr|A0A0C3QSZ7|A0A0C3QSZ7_9GAMM  .....
tr|A0A6L7HXW0|A0A6L7HXW0_9GAMM  .....
sp|A3QFP3|FADJ_SHELP            .....
tr|A0A1E5IXH7|A0A1E5IXH7_SHECO  .....
tr|A0A411PKQ0|A0A411PKQ0_9GAMM  .....
tr|A0A6G9QKM3|A0A6G9QKM3_9GAMM  .....
tr|A0A6P1UL63|A0A6P1UL63_9GAMM  .....
tr|A0A2N1ERI9|A0A2N1ERI9_9GAMM  .....
tr|A0A7W4FU55|A0A7W4FU55_9GAMM  .....
sp|Q8ECP7|FADJ_SHEON            .....
tr|A0A501XZY8|A0A501XZY8_9GAMM  .....
tr|A0A2W5DCZ0|A0A2W5DCZ0_SHEOE  .....
tr|A0A1E3V3C8|A0A1E3V3C8_9GAMM  .....
tr|A0A1Z4AI20|A0A1Z4AI20_9GAMM  .....
tr|A0A7X9LJL9|A0A7X9LJL9_9GAMM  .....
tr|A0A073KMY5|A0A073KMY5_9GAMM  .....
sp|A0KV76|FADJ_SHESA            .....
tr|A0A220UTH7|A0A220UTH7_9GAMM  .....
tr|A0A5B8R6W1|A0A5B8R6W1_9GAMM  .....
tr|V1DAI4|V1DAI4_9GAMM          .....
tr|A0A448CPQ4|A0A448CPQ4_SHEPU  .....
tr|A0A252ERQ3|A0A252ERQ3_SHEPU  .....
sp|Q0HKD1|FADJ_SHESM            .....
sp|Q0HWN3|FADJ_SHESR            .....
tr|F7RQE3|F7RQE3_9GAMM          .....
tr|B8EE98|B8EE98_SHEB2          .....
sp|A6WQ25|FADJ_SHEB8            .....
tr|A0A448EK41|A0A448EK41_9GAMM  .....
tr|A0A553JHX1|A0A553JHX1_SHEHA  .....
tr|B8CPY6|B8CPY6_SHEPW          .....
tr|A0A431WFC4|A0A431WFC4_9GAMM  .....
tr|A8FTR7|A8FTR7_SHESH          .....
tr|A0A431WNL0|A0A431WNL0_9GAMM  .....
tr|A0A550AEC5|A0A550AEC5_9GAMM  .....
tr|A0A7L4WW90|A0A7L4WW90_9GAMM  .....
tr|A9DDU3|A9DDU3_9GAMM          .....
tr|A0A330M2B9|A0A330M2B9_9GAMM  .....
tr|A0A5N8UFC7|A0A5N8UFC7_9GAMM  .....
tr|A0A1S6HN57|A0A1S6HN57_9GAMM  .....
tr|D4ZMH7|D4ZMH7_SHEVD          .....
tr|A0A3L8Q213|A0A3L8Q213_9GAMM  .....
tr|A0A3A6U4N9|A0A3A6U4N9_9GAMM  .....
tr|A0A4Q5MA37|A0A4Q5MA37_9GAMM  .....
tr|A0A1L6LSX5|A0A1L6LSX5_9DELT  .....
tr|A0A2W4L9B9|A0A2W4L9B9_9PROT  .....
tr|A0A2W4M4E6|A0A2W4M4E6_9PROT  .....
tr|A0A6I2GRX9|A0A6I2GRX9_9DELT  .....
tr|A0A0H4WMK2|A0A0H4WMK2_9DELT  .....
tr|F8CJ36|F8CJ36_MYXFH          .....
tr|A0A250K0F1|A0A250K0F1_9DELT  .....
tr|A0A7Y6WFZ2|A0A7Y6WFZ2_9DELT  .....
tr|A0A7Y7C660|A0A7Y7C660_9DELT  .....
tr|A0A7Y4JFH2|A0A7Y4JFH2_MYXXA  .....
tr|A0A4Y6CZQ9|A0A4Y6CZQ9_MYXXA  .....
```

```
tr|A0A7Y4IKV5|A0A7Y4IKV5_MYXXA  .....
tr|A0A511HHB0|A0A511HHB0_9DELT  .....
tr|A0A4Y6CKY7|A0A4Y6CKY7_MYXXA  .....
tr|Q1D1F2|Q1D1F2_MYXXD          .....
tr|A0A7Y4MA14|A0A7Y4MA14_MYXXA  .....
tr|A0A7T8Y4N9|A0A7T8Y4N9_MYXXA  .....
tr|L7UE67|L7UE67_MYXSD          .....
tr|A0A511T9X1|A0A511T9X1_MYXFU  .....
tr|A0A7Y7C9C4|A0A7Y7C9C4_9DELT  .....
tr|A0A540X7W8|A0A540X7W8_9DELT  .....
tr|A0A3A5FK19|A0A3A5FK19_9DELT  .....
tr|A0A3A8JQL9|A0A3A8JQL9_9DELT  .....
tr|A0A7Y4NFA4|A0A7Y4NFA4_9DELT  .....
tr|A0A3A8HDQ7|A0A3A8HDQ7_9DELT  .....
tr|A0A3A8GR90|A0A3A8GR90_9DELT  .....
tr|A0A3A8SBD7|A0A3A8SBD7_9DELT  .....
tr|A0A3A8T0I8|A0A3A8T0I8_9DELT  .....
tr|A0A7X5BU07|A0A7X5BU07_9DELT  .....
tr|A0A7Y1RVL2|A0A7Y1RVL2_9DELT  .....
tr|A0A3A8THN2|A0A3A8THN2_9DELT  .....
tr|A0A7Y1RX76|A0A7Y1RX76_9DELT  .....
tr|A0A3A8RA58|A0A3A8RA58_9DELT  .....
tr|A0A410RPB6|A0A410RPB6_CORCK  .....
tr|A0A7Y4J474|A0A7Y4J474_CORCK  .....
tr|A0A3A8I9Z6|A0A3A8I9Z6_9DELT  .....
tr|H8MKE9|H8MKE9_CORCM          .....
tr|A0A3A8H102|A0A3A8H102_9DELT  .....
tr|A0A554FW33|A0A554FW33_9DELT  .....
tr|A0A3A8NPC0|A0A3A8NPC0_9DELT  .....
tr|A0A3A8JUV3|A0A3A8JUV3_9DELT  .....
tr|A0A3A8NEX6|A0A3A8NEX6_9DELT  .....
tr|A0A3A8JEU8|A0A3A8JEU8_9DELT  .....
tr|A0A3A8LI76|A0A3A8LI76_9DELT  .....
tr|A0A085WXN8|A0A085WXN8_9DELT  .....
tr|A0A2T4V0M5|A0A2T4V0M5_9DELT  .....
tr|A0A0G2ZSW5|A0A0G2ZSW5_9DELT  .....
tr|A0A3M2DKY0|A0A3M2DKY0_9DELT  .....
tr|A0A661NQ58|A0A661NQ58_9DELT  .....
tr|A0A520YD99|A0A520YD99_9DELT PRVSA
tr|A0A7Y3BRE4|A0A7Y3BRE4_9DELT PRVSA
tr|A0A2D9TF90|A0A2D9TF90_9DELT SARP.
tr|A0A2E0TP32|A0A2E0TP32_9DELT  .....
tr|A0A2E4Y3V1|A0A2E4Y3V1_9PROT  .....
tr|A0A2E6VRH4|A0A2E6VRH4_9DELT  .....
tr|A0A1F9FB59|A0A1F9FB59_9DELT  .....
```
