# Supplementary material for: Characteristics of Mycobacterium tuberculosis PtpA interaction and activity on the alpha subunit of human mitochondrial trifunctional protein, a key enzyme of lipid metabolism
Source: Front Cell Infect Microbiol. 2023 Jun 22;13:1095060. doi: 10.3389/fcimb.2023.1095060 (PMC10325834; doi:10.3389/fcimb.2023.1095060)
Supplement: Supplementary file 2 [file DataSheet_2.pdf]

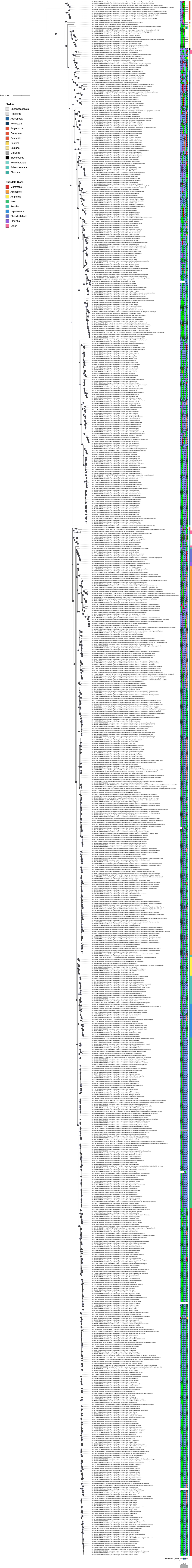

Tree scale: 1
Phylum
Choanoflagellata
Filasterea
Arthropoda
Nematoda
Euglenozoa
Oomycota
Priapulida
Porifera
Cnidaria
Mollusca
Brachiopoda
Hemichordata
Echinodermata
Chordata
Chordata Class
Mammalia
Actinopteri
Amphibia
Aves
Reptilia
Lepidosauria
Chondrichthyes
Cladistia
Other
