# Supplementary material for: Characteristics of Mycobacterium tuberculosis PtpA interaction and activity on the alpha subunit of human mitochondrial trifunctional protein, a key enzyme of lipid metabolism
Source: Front Cell Infect Microbiol. 2023 Jun 22;13:1095060. doi: 10.3389/fcimb.2023.1095060 (PMC10325834; doi:10.3389/fcimb.2023.1095060)
Supplement: Supplementary file 3 [file DataSheet_3.pdf]

Figure S1 (A).

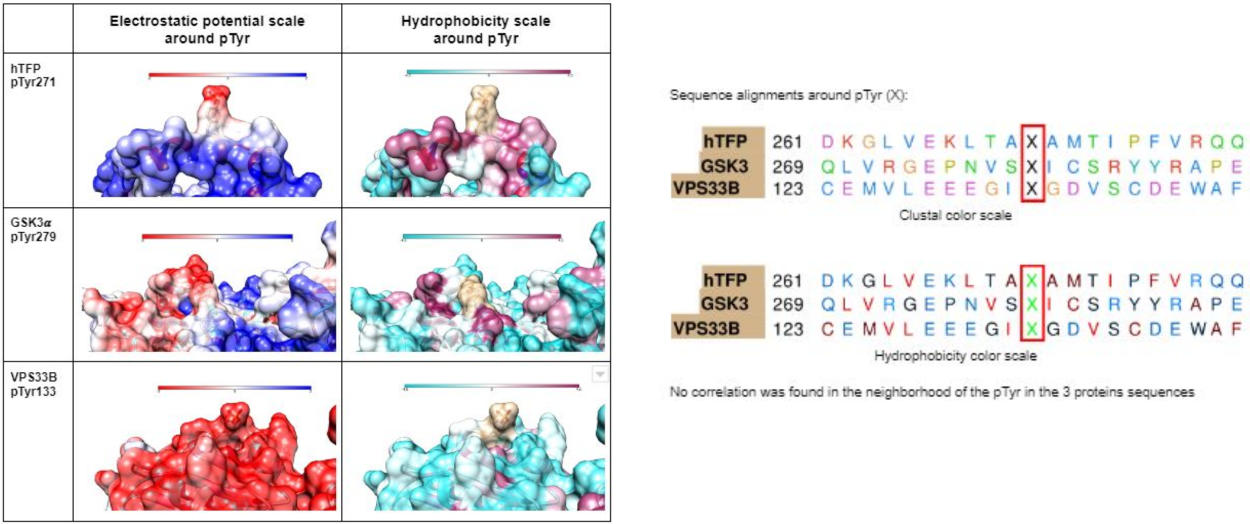

Figure S1 A. Protein surface around the p-Tyr residues for the reported substrates of PtpA: *h*TFP (in this paper and Margenat et al., 2015), GSK3$_{\alpha}$ (Bach et al., 2014) and VPS33B (Hmama et al., 2008), created by the MSMS package *(M.F. Sanner, A.J. Olson, and J.C. Spehner, "Reduced surface: an efficient way to compute molecular surfaces" Biopolymers 38:305 (1996)*). The first column shows their surface colored by the electrostatic potential calculated by the APBS program (from -2 Kcal/mol in red to 2 Kcal/mol in blue). The second column shows the surface colored by the hydrophobicity scale of Kyte and Doolittle (from -4.5 in cyan to 4.5 in maroon) (*Kyte & Doolittle, 1982, DOI: 10.1016/0022-2836(82)90515-0*). For the VSP33B protein, only the pTyr133 is shown since the other Tyr (Tyr382) is located with its side chain pointing towards the inner of the protein.

Figure S1 (B)

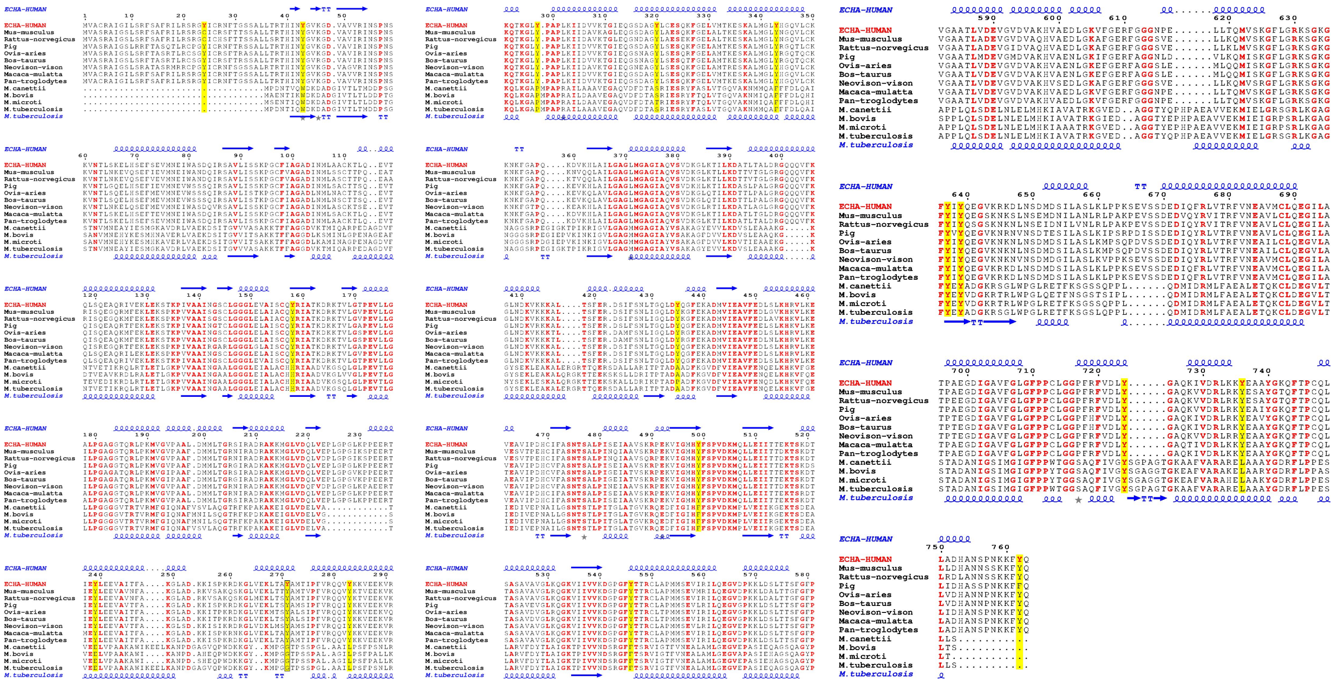

Figure S1 B. Complete multiple sequence alignment of protein homologs of *h*TFP$_\alpha$ representative of mammals and bacterial TB complex. Conserved residues are shown in red, and the tyrosines of *h*TFP$_\alpha$ are highlighted in yellow to visualize if they are conserved. The box indicates the Y271 residue in the helix-10 of the *h*TFP$_\alpha$. The alignment was obtained using the MUSCLE server (Edgar RC. 2004), and the figure was done using the ESPrit 3.0 server (Robert, X. and Gouet, P. 2014). The secondary protein structure of *h*TFP$_\alpha$ (human ECHA) and the *Mtb*FOM (*Mtb* ECHA) are also shown, based on the pdb structure 6DV2 (Xia et al, 2019) and 4B3H (Venkatesan et al, 2013), respectively.

Figure S2.

This document was added as an individual PDF for size reasons.

Figure S2. Multiple sequence alignment of bacterial protein homologs of *h*TFP$_{\alpha}$. The tyrosines of *h*TFP$_{\alpha}$ are highlighted in yellow; similar residues are shown in red and identical residues are highlighted in red boxes. The alignment was obtained using the MUSCLE server (Edgar RC. 2004), and the figure was done using the ESPrit 3.0 server (Robert, X. and Gouet, P. 2014).

Figure S3.

This document was added as an individual PDF for size reasons.

Figure S3. Phylogenetic analysis of the eukaryotes TFP trifunctional enzyme alpha subunit. Maximum likelihood tree represented as midpoint rooted. Node support is represented as circles sized according to SH-like aLRT value. Next to each leaf, the amino acids sequence alignment to human helix-10 is shown. Annotation: inner line: phylum colored according to the legend, outer line: Chordata class colored according to the legend.

Figure S4.

(A)

Protein name	Protein accession	Mass (Da)	Mascot Score	No of matched sequences	Peptide sequences	emPAI[(a)]
**Trifunctional enzyme subunit alpha, mitochondrial**	**ECHA_HUMAN**	83688	790	26 (26)	12 (12)	0,76
**Trifunctional enzyme subunit beta, mitochondrial**	**ECHB_HUMAN**	51547	480	14 (14)	6 (6)	0,52
E3 ubiquitin-protein ligase (TRIM21)	RO52_HUMAN	55162	599	28 (28)	9 (9)	0,92

[(a)] emPAI value is a parameter used as an estimation of the protein abundance in the sample.

(B)

Protein name	Protein accession	Mass (Da)	Mascot Score	No of matched sequences	No of unique peptide sequences
**Trifunctional enzyme subunit alpha, mitochondrial**	**ECHA_HUMAN**	81009	552	2(2)	2(2)
FKBP-type peptidyl-prolyl cis-trans isomerase	SLYD_ECOLI	20840	111	2(2)	2(2)

Figure S4. (A) Proteins identified by nano-LC-mass spectrometry after immunopurification of *h*TFP$_{\alpha,\beta}$ from macrophages. Proteins were identified by searching in the SwissProt database (taxonomy Homo sapiens) using the following parameters for the MS/MS ion search mode of the Mascot search engine: protein mass was unrestricted, peptide tolerance 1.5 Da, MS/MS tolerance 1 Da, cysteine carbamidomethylation, methionine oxidation as the allowed variable modifications, and one missed tryptic cleavage allowed. The significance limit for protein identification was set at $p<0.05$, and an ion cut-off 35. Only proteins identified with two or more peptides were considered positively identified.
(B) Proteins identified by MALDI-TOF-MS in the recombinant *h*TFP$_{\alpha}$ sample. MS analysis was carried out as described by Margenat et al, 2015, using the following parameters for the MS/MS ion search mode of the Mascot search engine: protein mass was unrestricted, peptide tolerance 0.1 Da, MS/MS tolerance 0.6 Da, cysteine carbamidomethylation, methionine oxidation as the allowed variable modifications, and one missed tryptic cleavage allowed. Sequence identity of *h*TFP$_{\alpha}$ (ECHA) was confirmed by MALDI-TOF (74% sequence coverage), and then also confirmed by Nano-LC-MS (97,9%).

Figure S5.

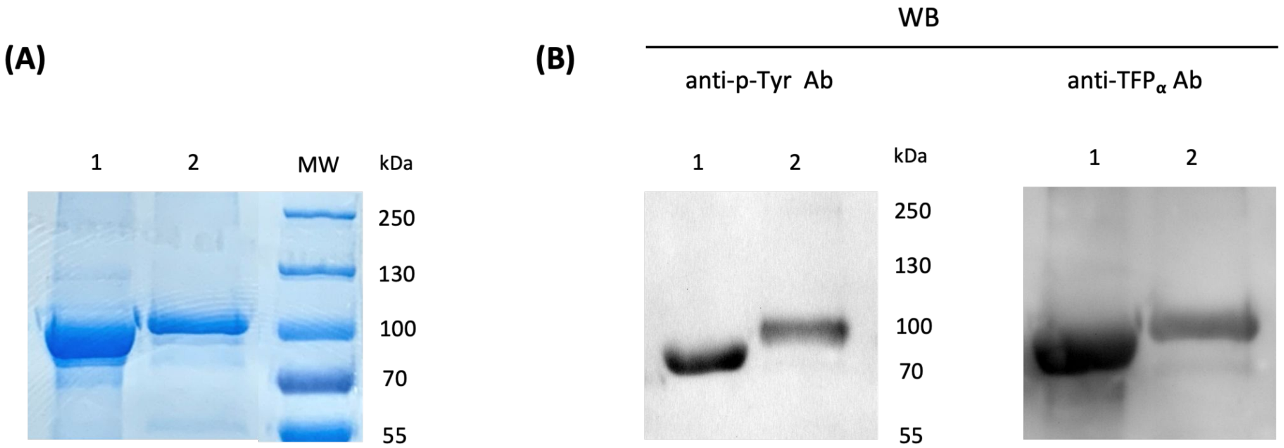

Figure S5. Phosphorylation of *h*TFP$_\alpha$ by Jak. (A) SDS-PAGE analysis (12% acrylamide) of recombinant *h*TFP$_\alpha$ untreated (lane 1) or treated (lane 2) with Jak. Consider that in line 1, two-times amount of sample was applied with respect to lane 2. The gel was stained with Coomassie Brilliant Blue G-250. **(B)** Images of the WB of the same samples analyzed in A. After SDS-PAGE, the proteins were transferred to PVDF membrane, blocked, and incubated with anti-p-Tyr antibody (Cell Signaling #9411, 1/2000) ON at 4°C. Then blots were washed and incubated with horseradish peroxidase (HRP)-linked anti-mouse (Sigma-Aldrich A4416, 1/10000) secondary antibody for 1 h at RT. The same membrane was reprobed with anti *h*TFPα rabbit antibody (Abcam, 1/1000) for 1.5 h at RT, washed, and incubated with (HRP)-linked anti-rabbit (Sigma-Aldrich, 1/50000) secondary antibody for 1 h at RT.

Figure S6.

	Detected p-Tyr	Peptide sequences	m/z	Measured MH	Theoretical MH	PPM	Primary Score	Secondary Score	Delta CN	Peaks Matched	Red Time	Classification Score
**Before treatment with Jak**	Tyr499	KVIGMH**Y[79.9663]**FSPVDKMQLLEIITTEK	968,155	2902,4504	2900,470121	-9,103	1,8879	9,772459	0,452	8	68,98	0,211666476
**After treatment with Jak**	Tyr435	ALTSFERDSIFSNLTGQLD**Y[79.9663]**QGFEKAD	1045,4754	3134,4115	3132,419737	-4,7655	2,0501	7,42192	0,4108	9	68,89	0,210116272
	Tyr271	LTA**Y[79.9663]**AMTIPFVR	731,867	1462,7267	1462,716369	7,0628	1,3658	7,420249	0,5141	5	71,87	0,64719594
	Tyr499	KVIGMH**Y[79.9663]**FSPVDKMQLLEIITTEK	968,489	2903,4525	2900,470121	-9,5304	2,0332	13,222184	0,5616	9	69,03	0,363273411
	Tyr43	THIN**Y[79.9663]**GVKGDVAVVR	569,9602	1707,8659	1707,857765	4,7633	3,1363	21,506338	0,6802	15	46,35	0,780111649

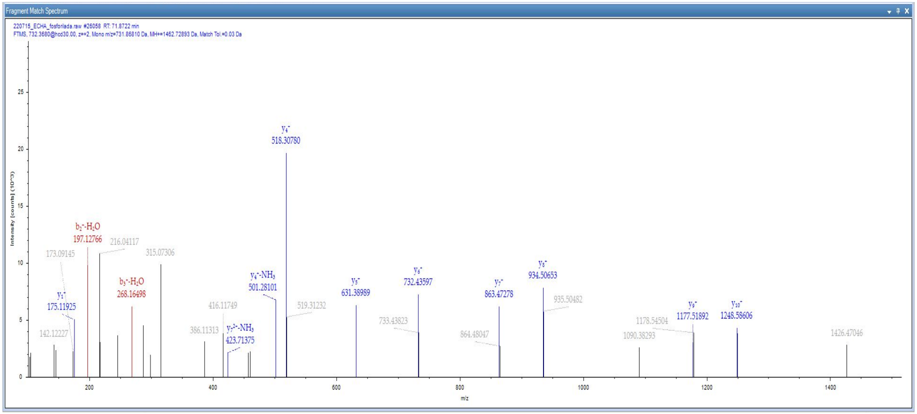

Figure S6. MS identification of p-Tyr residues of *h*TFP$_\alpha$ untreated or treated with the Jak kinase. In the table, the p-Tyr founds phosphorylated (+80 Da) are indicated. The graphic shows the fragmentation of the peptide containing the p-Tyr271. Internal fragment assignment: 315.07 Da = AY (phosphorylated), 386.11 Da = AYA (phosphorylated), 416.11 Da= TAY (phosphorylated), and 216.04 Da to the immonium ion phosphotyrosine. For detail, see the Methods section.

Figure S7. Table of PTMs of *h*TFP$_\alpha$. The table summarizes all the PTMs of hTFP$_\alpha$ reported up to now.

The PTMs of *h*TFP$_\alpha$ identified after large-scale MS studies and added to the PhosphoSitePlus, and the p-Tyr identified in the present study.

**Tyr Phosphorylation**	**Y43**#, **Y271**#, Y239#, Y298, Y435*,#, Y499*, Y546, Y637#, Y639#, Y724#, Y740
Thr/Ser Phosphorylation	T117, S120, T167, T171, T185,T203, S257, T294, S316, T387, T393, T395, T418, S419, S435, S453, S501, T521, S522, S524, S650, S665, S668, S669, T745, S756, T745, S756
Mono-methylation	R399, R549
Ubiquitylation	K60, K129, K214, K249, K303, K309, K350,K353, K359, K383, K390, K406, K415, K489, K519, K531, K605, K625, K646, K728, K735, K742
Succinylation	K46, K259, K334, K350, K359, K390, K406, K414, K415, K519,K531, K540, K569, K644, K728
Acetylation	K60, K129, K163, K214, K249, K259, K285, K289, K295, K303, K326, K334, K350, K351, K353, K359, K383, K390, K406, K411, K413, K460, K505, K519, K540, K569, K570, K597, K605, K644, K728, K735, K742, K759, K760

The new p-Tyr residues detected in the present study are highlighted in bold. (*) p-Tyr residues detected in the present study and also reported in PhosphoSitePlus. (#) Tyr residues exposed to the solvent in the *h*TFP structure.

Figure S8.

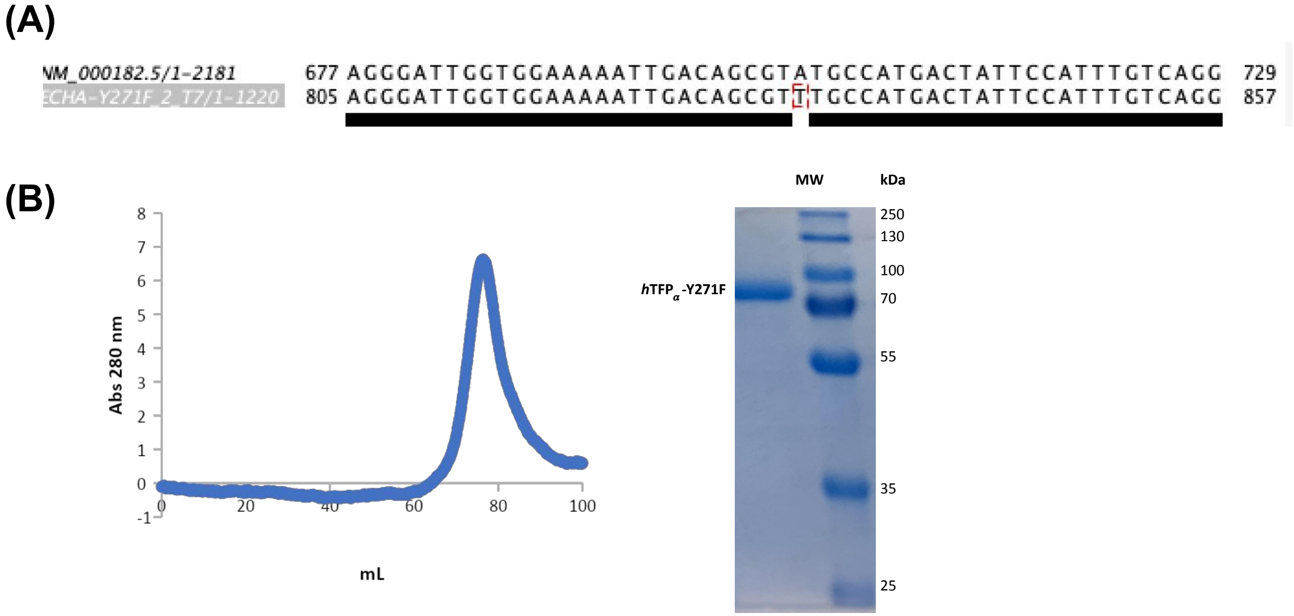

Figure S8. Production of the recombinant *h*TFP$_{\alpha}$-Y271F. (A) Sequence after the mutagenesis verifying the presence of the mutation corresponding to the Y271F aa change. **(B)** SEC profile of recombinant *h*TFP$_{\alpha}$-Y271F purification on Superdex 200 column. SEC was performed in an AKTA Basic system (GE Healthcare), injecting a sample volume of 4 mL in a Superdex 200 16/60 preparative grade column (GE Healthcare). Elution was carried out with two-bed column volumes of the equilibration buffer, and fractions containing the recombinant protein were pooled (elution volume of 74.5 mL). The apparent molecular weight was calculated using the following equation $Kav = -0.3872(\log Mw) + 2.2662$ corresponding to the previous calibration curve of the column using SEC molecular weight (SIGMA). A yield of 0.5 mg of soluble *h*TFP -Y271F /gr of *E.coli* pellet was obtained.

Figure S9.

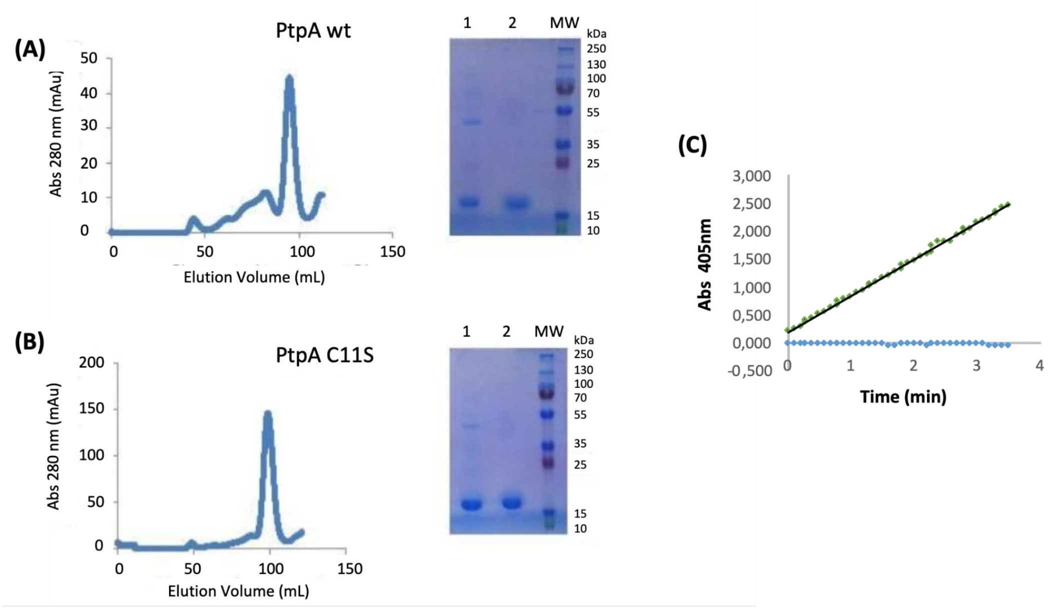

Figure S9. Production of the recombinant PtpA wt and the inactive mutant PtpA-C11S. Recombinant PtpA-wt **(A)** and PtpA-C11S **(B)** were purified by SEC onto a Superdex 200 16/60 preparative grade column (GE Healthcare). The major peak at 95 mL (21.5 kDa) corresponds to the monomeric native state of the protein PtpA wt (theoretical molecular weight 19.9 kDa). The major peak at 98 mL (16.4 kDa) corresponds to the monomeric native state of the protein PtpA-C11S. At the right, it shows the SDS-PAGE analysis (15% acrylamide) of the recombinant proteins purified by IMAC (lane 1) and after SEC (lane 2). Mw, molecular weight marker. All gels were stained with Coomassie Brilliant Blue R-250. Protein purification was carried out as described in Margenat et al. (Margenat et al., 2015). **(C)** The phosphatase activity of rPtpA-wt and rPtpA-C11S was evaluated using the artificial substrate p-nitrophenyl phosphate (*p*NPP) at 410 nm as described previously (Margenat et al., 2015). Results obtained with PtpA wt are shown as green dots, while sky blue dots show the results obtained with PtpA-C11S.

Figure S10 (A).

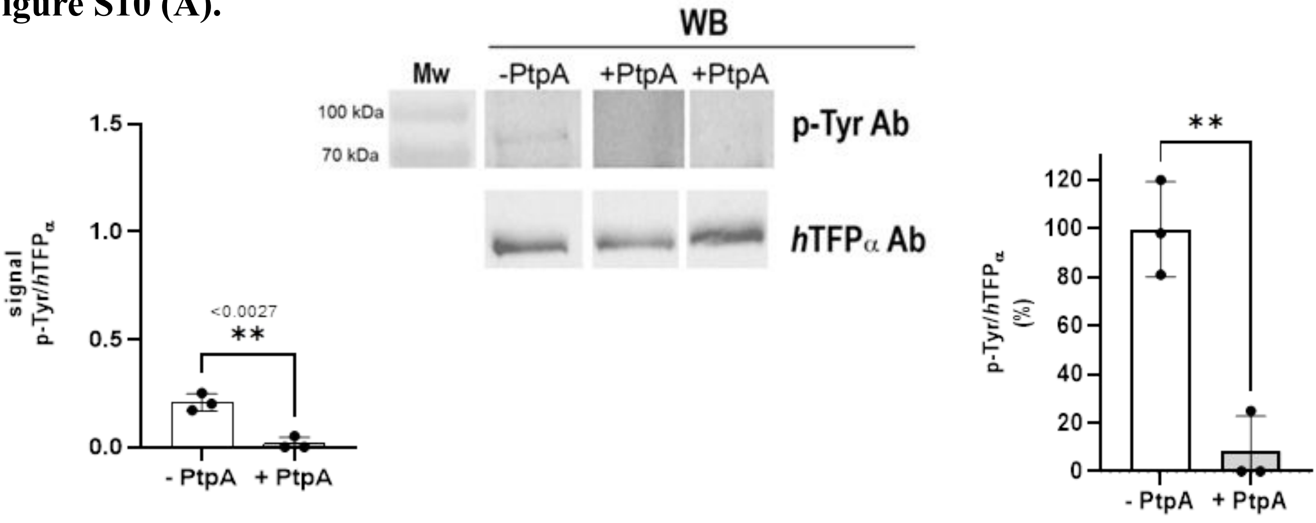

Figure S10 A. Dephosphorylation of the immobilized immunopurified *h*TFP$_\alpha$ by mycobacterial PtpA. The graph on the left shows p-Tyr/hTFP$_\alpha$ ratios, and the graph on the right shows the corresponding values expressed as %. The average p-Tyr/hTFP$_\alpha$ ratio before incubation with PtpA (-PtpA) was considered as 100%. Error bars represent experimental variability detected between replicates, and the asterisks are the p-value obtained after an unpaired t-test. The entire images of the WB are shown in the next slide. To evaluate PtpA activity on immobilized *h*TFP we used the protocol described by Najarro (Najarro et al., 2001). Equal amounts of the purified *h*TFP$_\alpha$ were resolved by SDS-PAGE, transferred to PVDF-membrane, blocked, and then incubated with 0μM (-PtpA) and 1.5 μM (+PtpA) in 20 mM Tris-HCl, pH 8.0, 50 mM NaCl, 3 mM EDTA, 1 mM DTT, 5% glycerol, 0.005% Tween 20, at 37°C for 30 min. Afterward, membranes were washed in TBS-T and probed for p-Tyr levels with anti-p-Tyr antibody (Cell Signalling #9411) at 1/2000 dilution in TBS-T, ON at 4° C. Blots were then incubated with horseradish peroxidase (HRP)-linked anti-mouse (Sigma-Aldrich A4416, 1/10000) secondary antibody for 1 h at RT. After four washes with TBS-T, and one wash with TBS, the reaction was developed with Pierce ECL western blotting substrate (Thermo Scientific). The chemiluminescent signals of the bands were visualized using the GBOX ChemiSystem tool (SynGene). The same membranes were reprobed with anti *h*TFP$_\alpha$ antibody to determine the ratio between p-Tyr and *h*TFP$_\alpha$ (Abcam ab200652) chemiluminescent signals, quantified using ImageJ (Schneider et al., 2012).

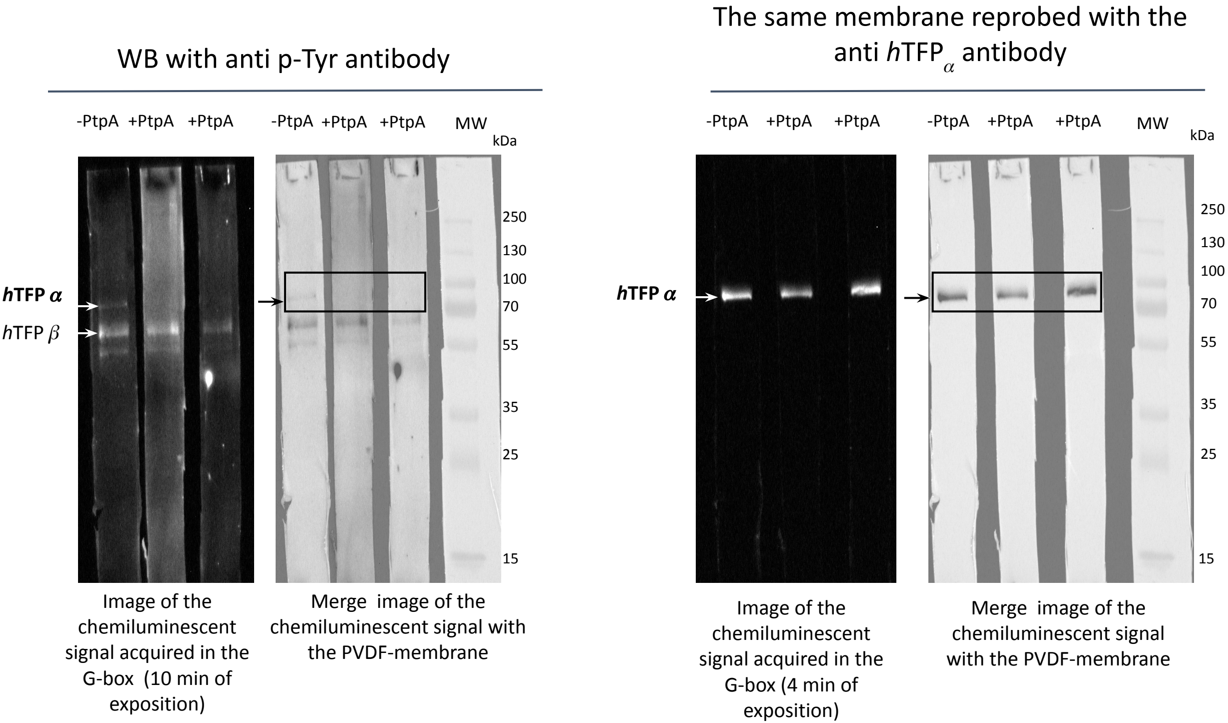

Dephosphorylation of the immobilized immunopurified *h*TFP$_{\alpha}$ by mycobacterial PtpA. The regions of these images used to compose Figure S10 (A) are indicated by black boxes.

Figure S10 (B).

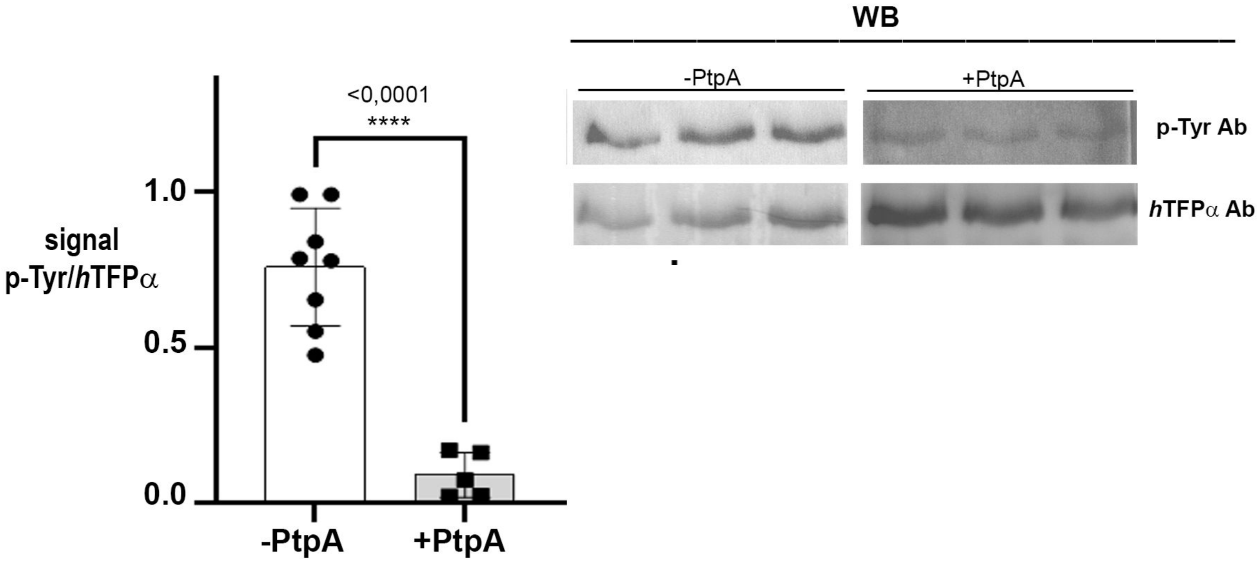

Figure S10 B. Dephosphorylation of the immobilized recombinant *h*TFP$_\alpha$ by mycobacterial PtpA. Equal amounts of the recombinant *h*TFP$_\alpha$ were resolved by SDS-PAGE, transferred to PVDF membrane, blocked, and then incubated without (-PtpA) and purified PtpA-wt 1.5 μM (+PtpA) at 37°C for 30 min. Dephosphorylation was evaluated with the anti-p-Tyr antibody and then the membranes was reprobed with anti *h*TFP$_\alpha$antibody to determine the ratio between p-Tyr and *h*TFP$_\alpha$ chemiluminescent signals. WB signals were quantified using ImageJ (Schneider et al., 2012). The statistical analysis was performed using an unpaired Student's t-test ($p < 0.05$). All data are presented as mean ± SD of two independent experiments. Calculations were done using GraphPad Prism. A representative WB membrane of this assay was included in the figure.

WB with anti p-Tyr antibody

-PtpA +PtpA

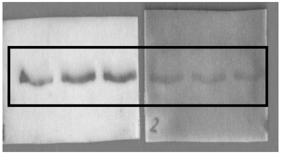

The same membrane reprobed with the anti *h*TFP$_{\alpha}$ antibody

-PtpA +PtpA

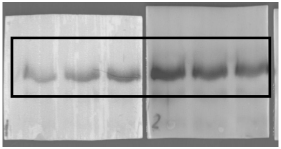

Merge image of the chemiluminescent signal with the PVDF-membrane acquired in the G-box (6 min of exposition)

Dephosphorylation of the immobilized recombinant *h*TFP$_{\alpha}$ by mycobacterial PtpA.
The regions of these images used to compose Figure 10 B are indicated by black boxes.

Figure S10 (C) and (D)

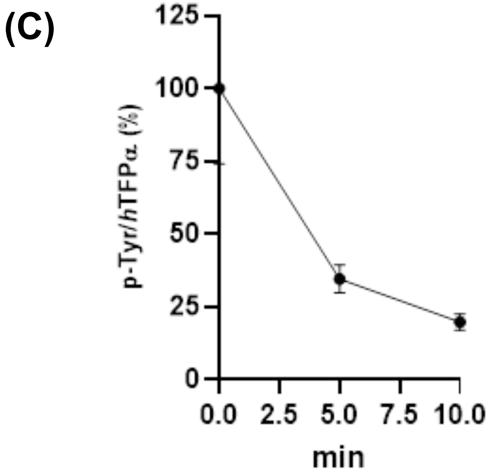

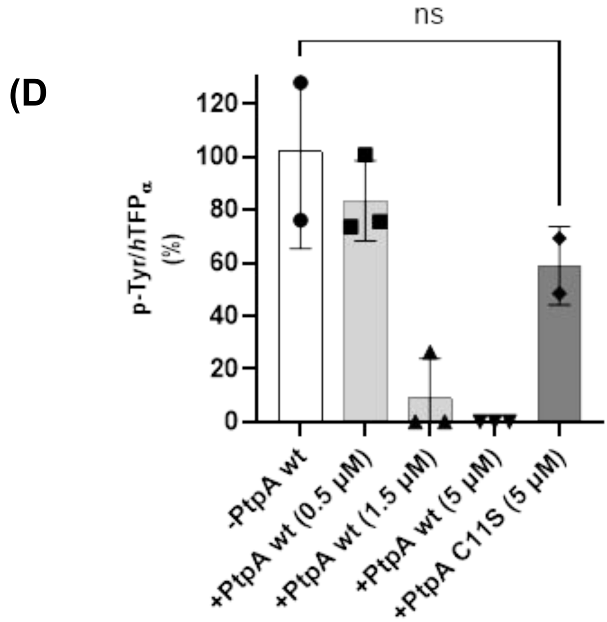

Figure S 10 (C). Dephosphorylation kinetics of immobilized recombinant *h*TFP$_\alpha$. Equal amounts of the recombinant hTFP$_\alpha$ were resolved by SDS-PAGE, transferred to PVDF membrane, blocked, and then incubated with 1.5 μM of PtpA at 37°C during different times. **(D). Dephosphorylation of the recombinant *h*TFP$_\alpha$ at different concentrations of PtpA.** Equal amounts of the recombinant *h*TFP$_\alpha$ were resolved by SDS-PAGE, transferred to PVDF membrane and blocked, and then incubated with or without purified PtpA-wt (0 μM, 0.5 μM, 1.5 μM, 5 μM) and the inactive mutant PtpA-C11S (5 μM) at 37°C for 30 min. Dephosphorylation was evaluated with the anti-p-Tyr antibody. The same membranes were reprobed with anti *h*TFP$_\alpha$ antibody to determine the ratio between p-Tyr and *h*TFP$_\alpha$ chemiluminescent signals. WB signals were quantified using ImageJ (Schneider et al., 2012). The average p-Tyr/*h*TFP$_\alpha$ ratio before incubation with PtpA (-PtpA) was considered 100%. Error bars represent experimental variability detected between replicates, and (ns) represents a non significant difference observed after an unpaired t-test.

Figure S10 (E)

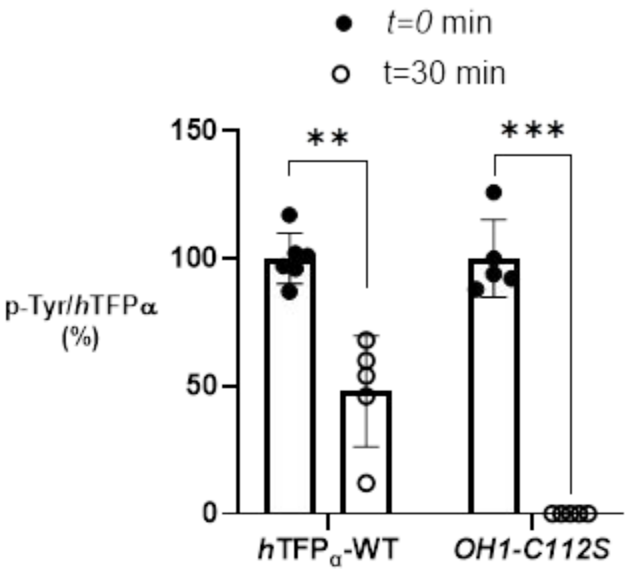

Figure S 10 (E). Dephosphorylation of the immobilized recombinant *h*TFP$_{\alpha}$ and OH1-C112S by PtpA. Equal amounts of the recombinant hTFP$_{\alpha}$ or rOH1-C112S, previously phosphorylated by Jak, were resolved by SDS-PAGE, transferred to PVDF membrane, blocked, and then incubated with 1.5 μM of PtpA for 30 min at 37° C. Dephosphorylation was evaluated with the anti-p-Tyr antibody. Chemiluminescent signals were quantified using ImageJ (Schneider et al, 2012). The average p-Tyr signal at t=0 was considered as 100%. The asterisks represent the p-value obtained after an unpaired t-test between t=0 and t=30 minutes.

Figure S11.

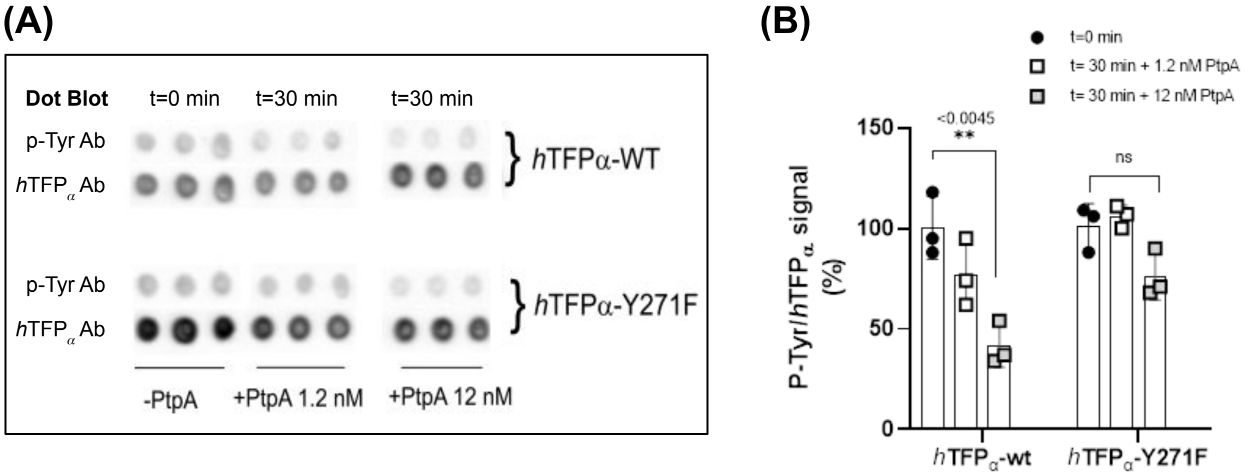

Figure S11. PtpA activity using *h*TFP$_\alpha$-wt and *h*TFP*α*-Y271F. (A) Dot blot showing the p-Tyr and *h*TFP signals after incubation of *h*TFP$_\alpha$ and *h*TFP$_\alpha$-Y271F with PtpA. Equal amounts of the recombinant *h*TFP$_\alpha$and *h*TFP*α*-Y271F were incubated with 1.2 nM and 12 nM of PtpA for 30 min at 37°C. Spots of 5 µL of the reaction at the corresponding time (t=0 min, t=30 min) were applied in a nitrocellulose membrane by triplicates. Dephosphorylation was evaluated with the anti-p-Tyr antibody. The same membranes were reprobed with anti *h*TFP$_\alpha$ antibody to determine the ratio between p-Tyr and *h*TFP$_\alpha$ chemiluminescent signals quantified using ImageJ (Schneider et al., 2012). **(B)** Graph showing the p-Tyr/*h*TFP$_\alpha$ ratios expressed as %. The average p-Tyr/hTFP*α* ratio at t=0 min was considered as 100%. Error bars represent experimental variability detected between replicates. After a two-way ANOVA a significant difference ($p<0.0001$) was detected between both groups (*h*TFP$_\alpha$ or *h*TFP*α*-Y271F). The asterisks in the graph represent the p-value obtained after an unpaired t-test between t=0 and t=30 minutes.

Figure S12.

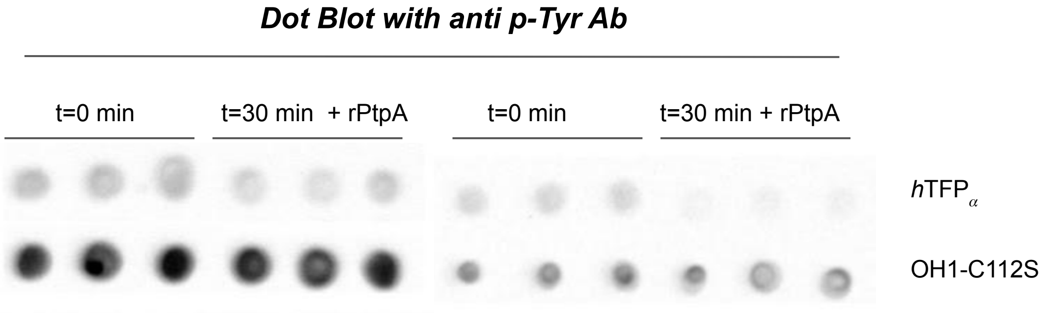

Figure S12. Evaluation of PtpA specificity. Equal amounts of *h*TFP$_\alpha$ or rOH1-C112S previously phosphorylated by Jak were incubated with 120 nM of PtpA (corresponding to a E:S molar ratio of 1:50) for 30 min at 37°C. Dephosphorylation was evaluated by a Dot Blot assay, with the anti-p-Tyr antibody. Spots of 5 µL of the reaction at the corresponding time (t=0 min, t=30 min) were applied in a nitrocellulose membrane by triplicates. Chemiluminescent signals were quantified using ImageJ (Schneider et al, 2012). The average p-Tyr signal at t=0 was considered as 100%. These dot-blot is representative of the replicates used to compose Figure 3 D.

Figure S13.

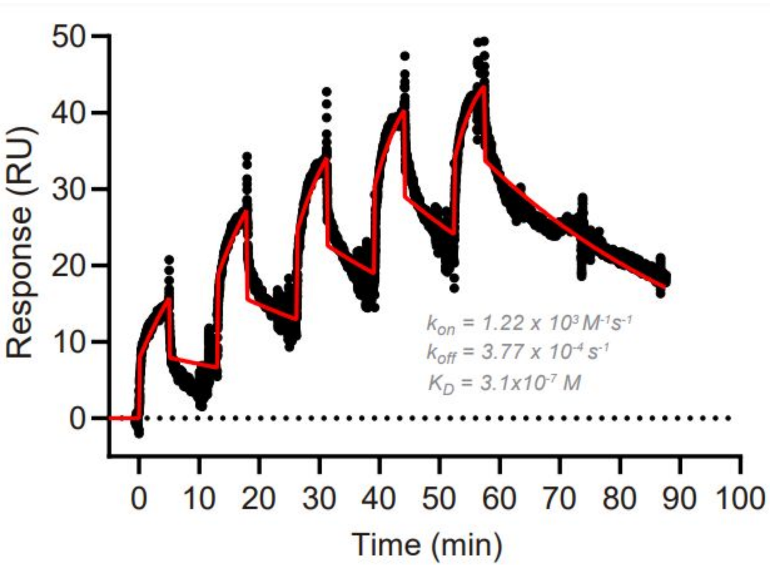

Figure S13. Real-time association-dissociation sensorgrams during the kinetic titration assay performed with the immunopurified *h*TFP$_{\alpha/\beta}$. Experiments were done on a Biacore 3000 GE Healthcare instrument by immobilizing *h*TFP$_{\alpha/\beta}$ on a CM5 sensor chip (GE Healthcare). After surface activation with 0.05 M NHS (N-hydroxysuccinimide) and 0.2 M EDC (1-ethyl-3-(3-dimethyl aminopropyl) carbodiimide), *h*TFP$_{\alpha/\beta}$ diluted in 10 mM sodium acetate pH 5.6 was immobilized to a final density of 360 RU (at a flow rate of 50 μl/min) and the remaining sites were blocked with 1.0 M ethanolamine pH 8.5. Successive injections of PtpA at different concentrations (3.5 µM, 4,72 µM, 7.09 µM, 10.63 µM, and 15.94 µM diluted in running buffer (20 mM Tris-HCl pH 8.0, 50 mM NaCl, 5 mM EDTA, 0.005% P20, 1 mM DTT) at 25°C, for 5 minutes and a flow rate of 40 µl/min were performed. Cycles of association and dissociation of 5 min were performed before each injection. All titrations were double referenced, and the curves corresponding to the injections with running buffer performed during the same time intervals and the signal on the control surface were subtracted. The black curve corresponds to the data obtained after double referencing, and the red curve represents the fit to the 1:1 interaction model using the Kinetic Titration modality (Karlsson et al. 2006) of the BIAevaluation software.

Figure S14.

(A)

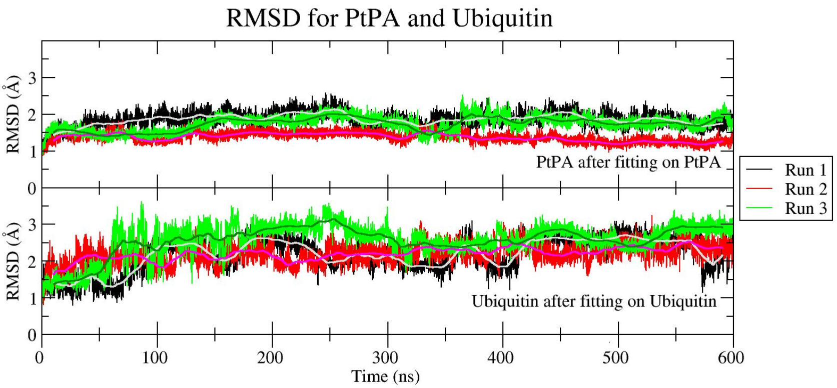

(B)

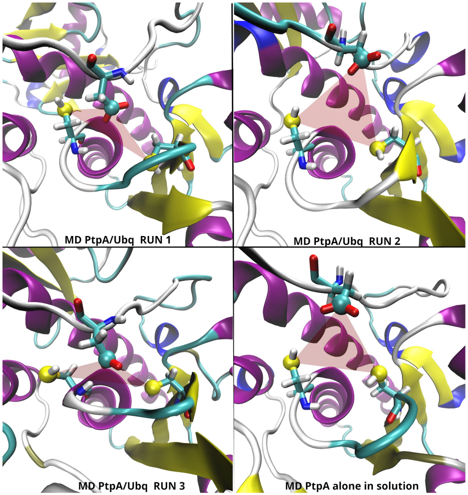

Figure S14. Molecular dynamics simulations of the PtpA/Ubq complex. (A) Root mean square deviation (RMSD) for the PtpA molecule in the complex (up) and Ubq molecule in the complex (down) in the three parallel MD runs. **(B)** Triangular surface defined for the PtpA binding site for the three MD runs of the complex along with the simulations of PtpA alone in solution.

Figure S15.

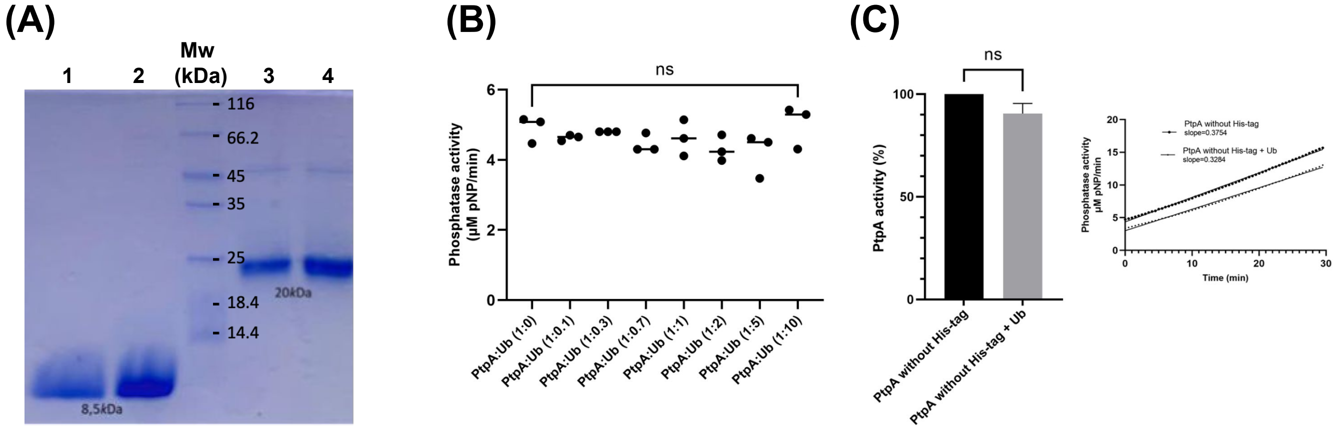

Figure S15 (A) SDS-PAGE analysis (15% acrylamide gel) of the purity of commercial ubiquitin (SIGMA U6253, 8.5 kDa, lanes 1 and 2) used in the kinetic assays of PtpA (20 kDa, lanes 3 and 4). The gel was stained with Coomassie Brilliant Blue R-250. **(B)** PtpA activity using pNPP as substrate after incubation with different concentrations of ubiquitin. The assays were performed with PtpA at 1.5 µM (final concentration) pre-incubated for 15 min at 37°C with or without ubiquitin before adding 20 mM pNPP artificial substrate. All data are presented as mean ± SD. No significant difference (ns) was observed after Kruskal-Wallis statistical analysis test using GraphPad Prism. **(C)** PtpA activity without His-tag in the absence or presence of ubiquitin. In the left, a graphical representation of the PtpA activity is expressed as %, considering as 100% the activity without the addition of ubiquitin. On the right is a graph of one of the kinetic assays used in the analysis. No-significant difference (ns) was observed after the unpaired t-test using GraphPad Prism.
